# RNA Sequencing of Murine Norovirus-Infected Cells Reveals Transcriptional Alteration of Genes Important to Viral Recognition and Antigen Presentation

**DOI:** 10.3389/fimmu.2017.00959

**Published:** 2017-08-11

**Authors:** Daniel Enosi Tuipulotu, Natalie E. Netzler, Jennifer H. Lun, Jason M. Mackenzie, Peter A. White

**Affiliations:** ^1^Faculty of Science, School of Biotechnology and Biomolecular Sciences, University of New South Wales, Sydney, NSW, Australia; ^2^Department of Microbiology and Immunology, Peter Doherty Institute for Infection and Immunity, University of Melbourne, Melbourne, VIC, Australia

**Keywords:** murine norovirus, norovirus infection, transcriptome, innate immunity, host response, antigen presentation, viral recognition

## Abstract

Viruses inherently exploit normal cellular functions to promote replication and survival. One mechanism involves transcriptional control of the host, and knowledge of the genes modified and their molecular function can aid in understanding viral-host interactions. Norovirus pathogenesis, despite the recent advances in cell cultivation, remains largely uncharacterized. Several studies have utilized the related murine norovirus (MNV) to identify innate response, antigen presentation, and cellular recognition components that are activated during infection. In this study, we have used next-generation sequencing to probe the transcriptomic changes of MNV-infected mouse macrophages. Our in-depth analysis has revealed that MNV is a potent stimulator of the innate response including genes involved in interferon and cytokine production pathways. We observed that genes involved in viral recognition, namely *IFIH1, DDX58*, and *DHX58* were significantly upregulated with infection, whereas we observed significant downregulation of cytokine receptors (*Il17rc, Il1rl1, Cxcr3*, and *Cxcr5*) and *TLR7*. Furthermore, we identified that pathways involved in protein degradation (including genes *Psmb3, Psmb4, Psmb5, Psmb9*, and *Psme2*), antigen presentation, and lymphocyte activation are downregulated by MNV infection. Thus, our findings illustrate that MNV induces perturbations in the innate immune transcriptome, particularly in MHC maturation and viral recognition that can contribute to disease pathogenesis.

## Introduction

Human norovirus (NoV) is a leading cause of acute gastroenteritis worldwide with an estimated 684 million cases of diarrhea annually resulting in 212,000 deaths (1). Transmission of NoV usually occurs from person-to-person, and most outbreaks occur in closed settings such as cruise ships, hospitals, schools, and aged- and child-care facilities (2–4). Infection with NoV typically results in a combination of projectile vomiting, non-bloody watery diarrhea, often associated with symptoms such as nausea, chills, headaches, fever, and muscle aches. The infection is usually self-limiting within 2 days of symptom onset (2). However, dehydration can occur in young children, the elderly and the immunocompromised, which can ultimately lead to death (5, 6). Moreover, the global costs associated with NoV infections are estimated to exceed $60 billion annually, which includes medical related expenses, reduction in productive work days, and loss of wages as a result of absence from employment or school (7). In addition, the absence of an approved vaccine or effective antiviral further exacerbates the difficulty in controlling human NoV infections and outbreaks.

NoV is a member of the *Caliciviridae* family within the *Norovirus* genus, which contains seven genogroups (GI-GVII) with each further divided into several genotypes (8). Specifically, GI, II, and IV can infect humans (8, 9), GV infects mice (10), and other genogroups infect porcine, ovine, bovine, and canine species (11–13) The discovery of murine norovirus (MNV) in 2003 (10) represented the first model for *in vitro* NoV replication and *in vivo* animal studies (14). This culture system has facilitated our understanding of the fundamental properties NoV biology, including replication (15, 16), receptor entry (17–19), pathogenesis (20–22), and the discovery of potential antivirals (23–25). Given the significant health and economic impact of NoV globally, there is a need to investigate all aspects of NoV biology to combat infections.

Innate immunity is an essential part of the host response to limit viral replication and prevent disease manifestation. The general innate pathway has the following steps: virus detection by cellular receptors, receptor activation, recruitment of adapter proteins, intracellular signaling cascades, nuclear translocation of transcription factors, and expression of genes important for host defense and adaptive immune stimulation [reviewed in Ref. (26)]. Viral detection is carried out by host pathogen recognition receptors (PRRs), which are activated through their interaction with components of the virus structure called PAMPs (pathogen associated molecular patterns) (27). PRRs are divided into three groups, which include the toll-like receptors (TLRs), retinoic acid-inducible gene I (RIG-I)-like receptors (RLRs), and nucleotide-binding domain, leucine-rich repeat-containing receptors (NLRs) (28). Members of each receptor family are involved in nucleic acid detection and are expressed either within the plasma membrane or within the endosome membranes (28).

Viral activation of PRRs causes a powerful stimulation of several signaling pathways (29) involved in the type I interferon (IFN) response, including the mitogen activating protein kinase (MAPK) (30), nuclear factor kappa B(NFκB) (31), interferon regulatory factor (IRF) (32), and Janus kinase-signal transducer and activator of transcription (JAK-STAT) pathways. These antiviral pathways can influence host gene expression, protein production, and post-translational modifications to generate an antiviral state within the infected cell. In this study, we aim to understand how MNV overcomes this antiviral state and continues with robust replication in the host cell.

Previous work on MNV has demonstrated that STAT1, RAG2 (recombination activating gene), type I and type II IFN receptors are used to limit MNV infection in mice (10). Other components of innate immunity shown to play a role in MNV infection include MDA5 for viral recognition (33), IRF3 and IRF7 for antiviral transcriptional control (34), interferon stimulated gene 15 (ISG15) (35), type I, II, and III IFNs (36, 37). These innate pathways are involved in cytokine production, stimulation of the adaptive immune system, cell proliferation, and apoptosis (38). Other aspects of innate biology during MNV infection have also been explored, including MHC class I expression (39–41) and the antiviral properties of IFN-γ in the context of persistent infection (37).

The overall aim of the current study was to characterize the biological processes subverted by MNV, particularly those of the innate immune response, to gain insights into NoV pathogenesis. Earlier transcriptomic analyses of MNV infection have demonstrated alterations in the immune response (42), chemokine production (43), regulation of apoptosis (21), cholesterol synthesis (44), and the cell cycle (45). However, the availability of next-generation sequencing (NGS) in recent years has provided an unparalleled technique to measure the global transcription changes in response to viral infection. In the current study, RNA sequencing was used to probe the cellular transcriptome of mouse macrophages following longitudinal MNV infection to identify changes that occur as viral infection progresses. Furthermore, we analyzed transcriptomic profiles of RAW264.7 cells treated with the TLR7 agonist loxoribine and compared these with MNV-infected cells to reveal the cellular responses induced solely by viral infection. Our findings highlight key components of the host cell response affected by MNV and provide plausible explanations into the mechanisms by which NoV causes disease. First, we characterized induction of a robust innate response with changes detected as early as 4 hpi that continued to develop with increased viral replication. We show global downregulation of genes that encode proteins important for the control of gene transcription and protein translation, which may be involved in the host protein shut-off, a characteristic of calicivirus infection [reviewed in Ref. (46)]. In addition, we propose a mechanism by which MNV could regulate the expression of MHC class I molecules in a bid to limit immune recognition. We discuss the complexities by which MNV modulates transcript levels of genes in several biological pathways and their impact on our understanding of NoV pathogenesis.

## Materials and Methods

### Cell Maintenance, Stimulation, and Virus Infections

Murine macrophage RAW264.7 cells (a kind gift from Hebert W. Virgin, Washington University, School of Medicine, St. Louis, MO, USA), were maintained in Dulbecco’s Modified Eagle’s Medium (Invitrogen, Carlsbad, CA, USA) supplemented with 10% (v/v) fetal bovine serum (Sigma-Aldrich, St. Louis, MO, USA), 2 mM Glutamax (Thermo Fisher, Waltham, MA, USA), 100 U/mL penicillin (Thermo Fisher), and 100 µg/mL streptomycin (Thermo Fisher). MNV-1 CW1 strain (a kind gift from Hebert W. Virgin, Washington University, School of Medicine, St. Louis, MO, USA) used in this study was purified from culture supernatant by ultracentrifugation, as previously described (33). MNV infections were carried out longitudinally from 4 to 20 h at a multiplicity of infection (MOI) of 5, and infections at 12 h (MOI 5) were performed in quadruplicate for comparison with loxoribine treatment (1 mM). All experiments were carried out on monolayers of 1 × 10^7^ RAW264.7 cells, and after the appropriate incubation, cells were collected for RNA extraction. Mock infections were performed for all experiments with complete media.

### RNA Extraction and Quality Control

Viral and cellular RNA was extracted from infected RAW264.7 cell monolayers using TRIzol LS (Invitrogen, Carlsbad, CA, USA), with phase separation carried out as per the manufacturer’s instructions. RNA was further purified from contaminants using the RNeasy Mini Kit (Qiagen, Hilden, Germany), which included DNA removal using RNase-free DNase (Qiagen). RNA was quantified using spectrophotometry, and RNA integrity was assessed on a Bioanalyzer (Agilent Technologies) prior to downstream analysis.

### Library Preparation and Sequencing

RNA extracted from loxoribine-treated, MNV-infected, and mock-infected RAW264.7 cells was depleted of cytoplasmic and mitochondrial ribosomal RNA using the Ribo-Zero Gold Kit (human/mouse/rat) (Illumina), and libraries were prepared using reagents and protocols supplied in the TruSeq Stranded Library kit (Illumina). Briefly, RNA was chemically fragmented and then reverse transcribed using random hexamers. Thereafter, unique adapter sequences were ligated to the newly synthesized cDNA products and PCR amplified. Libraries were validated by BioAnalyzer followed by 75-bp paired-end read sequencing on the NextSeq500 platform (Illumina), carried out at the Ramaciotti Center for Genomics at the University of New South Wales. All sequence data have been submitted to the gene expression omnibus data repository under series numbers GSE94821 and GSE94843.

### Sequence Analysis

Bioinformatic analysis of RNA sequencing was performed using the Cufflinks tool suite (47) on the Galaxy server at the University of Queensland, Australia (48–50). Following quality control (removal of adapter sequences and trimming), reads were mapped to the 10 mm genome (UCSC) using TopHat (v0.9) with default parameters (51). Mapped reads were then assembled into transcripts, normalized and quantified using Cufflinks (v2.2.1.0) (47). Assembled transcripts of all replicates, within all conditions, were merged into a single cataloged transcriptome. Thereafter, transcript abundance was compared between mock and treated or infected samples using Cuffdiff (v2.2.1.2) to identify differentially expressed genes (DEGs) (52). Genes with a fourfold or greater expression change with a FPKM value greater than 1 in at least one sample were considered differentially expressed (DE) for the longitudinal infection analysis. Genes with a *q*-value of <0.05, twofold or greater expression change, and a FPKM value of 1 in at least one sample were considered DE for the analysis of cells infected with MNV or treated with loxoribine for 12 h.

To confirm the presence or absence of viral replication, sequencing reads were mapped using Bowtie2 (53) to MNV-1 (GenBank accession number DQ285629), Abelson Murine Leukemia virus (Mu-LV) (GenBank accession number: NC_001499), and Moloney Mu-LV (GenBank accession number: NC_001501). The total number of reads at each position across the genome was used to quantify genomic coverage.

### Gene Enrichment Analysis

Enrichment analysis was performed to identify the functional role that the DEGs, identified from MNV infection and loxoribine treatment, play within the host. Gene lists were analyzed on the online servers DAVID (54) and GOrilla (55) for gene ontology and/or KEGG pathway analysis, and the most significant outputs were collated.

### Reverse Transcription-Quantitative Polymerase Chain Reaction (RT-qPCR)

Total cellular RNA (1 µg) was reverse transcribed in 10 µL reactions using the SuperScript VILO cDNA MasterMix synthesis kit (Thermo Fisher), following the manufacturer’s instructions. To quantitate viral genome copies and host gene expression levels following infection, qPCR was performed. Each reaction contained 2 µL of 10-fold diluted cDNA, 10 µL 2× iTaq universal SYBR Green supermix (Bio-Rad, Hercules, CA, USA) and 0.5 µM of both forward and reverse primers in a total volume of 20 µL (Table S8 in Supplementary Material). Amplification was performed on a RotorGeneQ (Qiagen) with 95°C denaturation for 1 min followed by 40 cycles of 95°C for 5 s, 55°C for 20 s, and 72°C for 20 s. Fold changes in mRNA abundance following MNV infection were calculated using the ΔΔ*C*_t_ method (56). Viral genomes were quantified using qPCR primers that amplify a 187 bp product within the MNV RdRp encoding region at the 3′ end of ORF1, as previously described (25).

### Protein Extraction and Immunoblotting

To extract proteins, MNV or mock infected cells were lysed in RIPA buffer (Sigma) supplemented with 100× protease/phosphatase inhibitor cocktail (Cell Signaling Technology, Danvers, MA, USA). Protein concentrations were measured using the BCA assay (Thermo Fisher). Protein (50 µg) from each sample was separated on an 10% Tris-glycine SDS-polyacrylamide gel (Bio-Rad) and transferred to a polyvinylidene difluoride membrane (Merck Millipore, Kenilworth, NJ, USA). Membranes were blocked with 5% skim milk in PBS with 0.1% Tween 20 and incubated with a primary antibody against the NoV capsid protein (Abcam, Cambridge, UK) (ab92976) at a 1:1,000 dilution for 90 min at room temperature. Thereafter, the membrane was incubated for 1 h with an anti-rabbit, HRP-linked secondary antibody (Santa Cruz Biotechnology) at a 1:10,000 dilution. Western blots were developed using a chemiluminescence HRP-substrate (Merck Millipore).

### Statistical Analyses

All bar and linear regression graphs were generated in PRISM v.6.0h and error bars are plotted with the SD of either triplicate or quadruplicate experiments of a condition. Correlation analysis was performed using the Pearson method on linear regression analysis.

## Results

### Productive MNV Infection in RAW264.7 Cells

**Figure 1:**
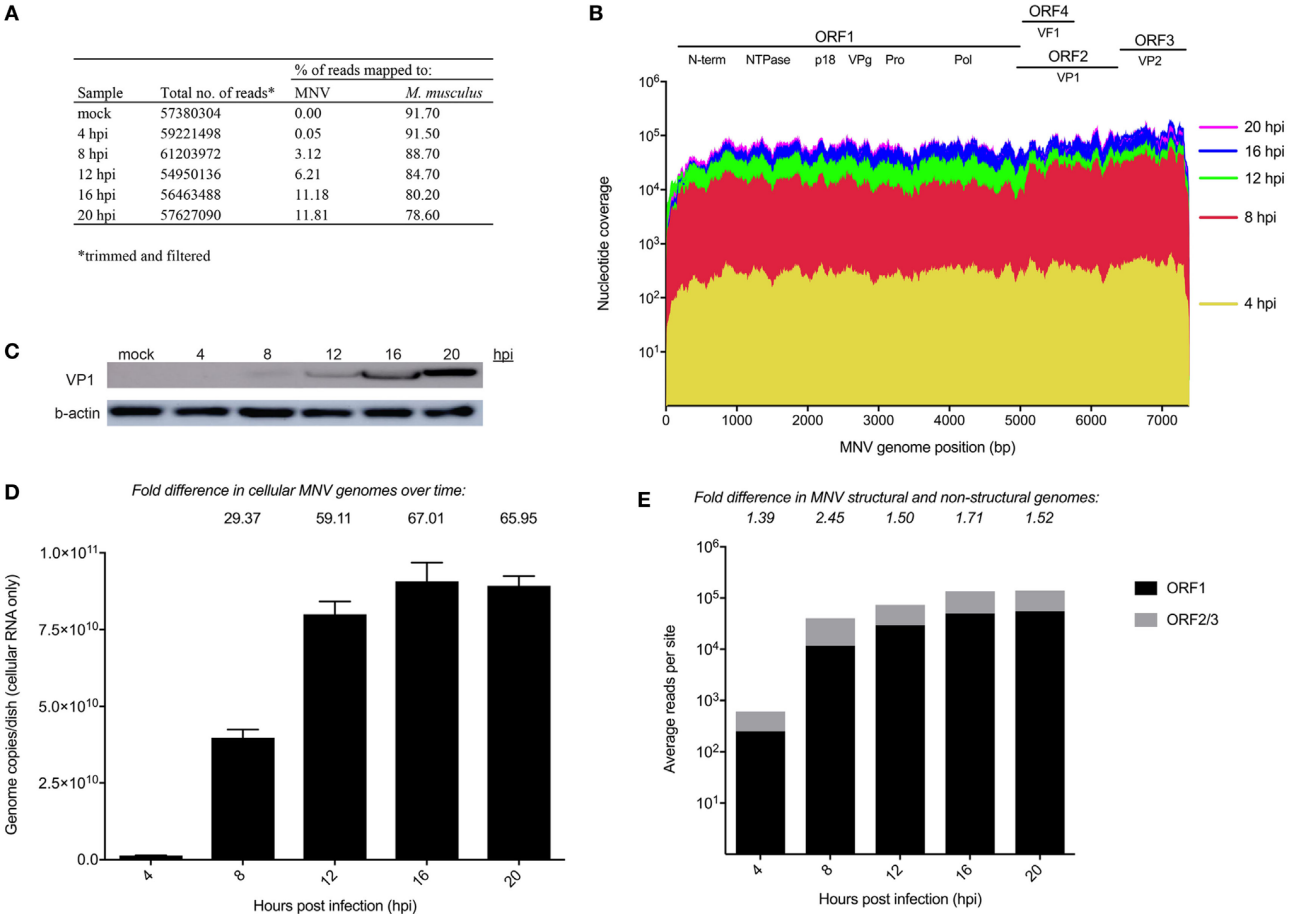
Longitudinal analysis of murine norovirus (MNV) replication. **(A)** Summary of sequencing reads mapped to the host (mm10) and viral (MNV-1.CW1/DQ285629) genomes. **(B)** Sequencing reads from each infection time point were mapped to the MNV genome and are represented in a coverage plot stratified by genome position. **(C)** Western blot for MNV viral protein 1 (VP1) (ORF2) structural protein was performed to quantify viral protein levels over time. **(D)** qRT-PCR for was performed quantify viral genomes over time. **(E)** Quantification of reads mapped to structural (gray) and non-structural regions (black) of the MNV genome. Fold differences between the average read coverage of ORF2-3 relative to ORF1 are listed above the graph.

The replication kinetics of MNV has been well studied (14, 15); however, little is known about the point at which MNV induces transcriptional changes within the host. We anticipated that cellular changes would mimic increases in viral replication, with more noticeable changes in gene expression occurring as infection progressed. RNA sequencing was used to quantify transcriptomic changes over time from 4 to 20 hpi and approximately 30 million 75-bp paired-end reads were generated for each sample (*n* = 12) with an average of 84% of reads mapped successfully to the mouse 10 mm (UCSC) reference genome (Figure 1A).

To have confidence that host expression changes were attributable to MNV infection, viral replication was assessed using several methods. Primarily, the RNA sequencing reads from each infection time point were mapped to the MNV CW1 strain reference sequence (Figure 1A) and nucleotide coverage at each position across the MNV genome was quantified (Figure 1B). This analysis revealed complete coverage of the MNV genome from sequencing reads with a >2 log increase in the abundance of MNV transcripts from 4 to 20 hpi. MNV replication was also confirmed by quantification of viral protein by Western blot targeting the major capsid protein, viral protein 1 (VP1) (Figure 1C) and RT-qPCR quantification of viral genomes (Figure 1D). Figure 1C shows increased signal intensity corresponding to MNV VP1 that correlates to increased viral replication. Similarly, viral genomes increased >60-fold over the 20 h infection period when measured by RT-qPCR (Figure 1D). Together, these gradual increases in viral genome and protein levels, detected by RNA sequencing, quantitative RT-qPCR, and Western blot, demonstrate robust MNV replication over time. Further to this, we also mapped reads to Abelson Mu-LV and Moloney Mu-LV genomes to rule out viral contamination. A negligible number of reads were mapped to either viral genome, with minimal coverage; >92% of either viral genome was missing. In addition, no significant increase in reads was detected over the time course (Table S9 in Supplementary Material).

Murine norovirus replication involves the production of both genomic and subgenomic RNA (14, 57, 58) both of which encode proteins that are critical to the production of infectious virions (59). To ascertain the relative abundance of genomic and subgenomic RNA, the average nucleotide coverage spanning ORF1 and ORF2–3 were individually quantified and compared. We found a greater level of nucleotide coverage at the ORF2-3 structural region compared to non-structural ORF1 region at each infection time point (1.39-, 2.45-, 1.5-, 1.71-, and 1.52-fold increase for 4, 8, 12, 16, and 20 hpi, respectively) (Figure 1E). The higher proportion of reads mapped to the ORF2-3 region (average of 1.7-fold) confirms the presence of a subgenomic RNA species.

### MNV Infection Induces Pronounced Innate Host Expression Changes with Time

**Figure 2:**
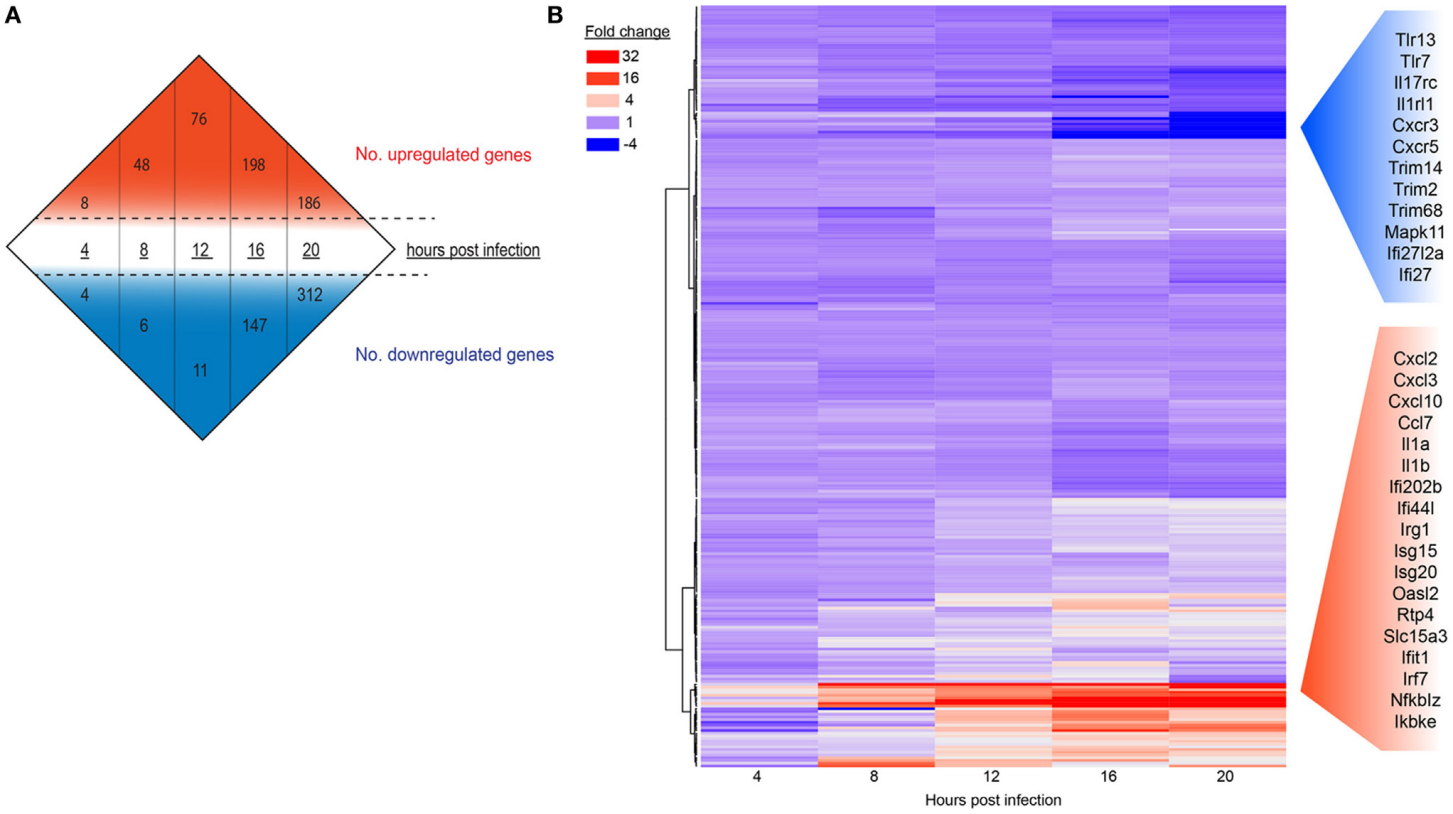
The host response of RAW264.7 cells infected with murine norovirus (MNV) over time. **(A)** Differentially expressed genes (fourfold or more change and FPKM value >1 in at least one sample) for each infection time are summarized numerically. Values above (red) and below (blue) the hashed lines represent upregulated and downregulated genes, respectively. **(B)** A total of 280 genes involved in the innate response were probed over time and are displayed in a heat map. Each panel represents a particular gene, and the color depicts the fold change at each time point. Red and blue side panels represent upregulated and downregulated genes, respectively, that show the greatest level of change with MNV infection.

To determine if gene expression changes would intensify as MNV replication proceeds and to measure the intensity of the host response to MNV infection, we analyzed the global expression changes over time (Figure 2A) (Table S1 in Supplementary Material), in addition to a more focused analysis of innate gene expression (Figure 2B). A simple enumeration of all genes altered within the mouse macrophages revealed a marked increase in the number of DEGs from 4 to 20 hpi (23- and 78-fold for upregulated and downregulated genes, respectively) (Figure 2A) that correlated with increased MNV replication (Figure 1). Furthermore, a heatmap of 280 innate immune-related genes (Figure 2B) exhibits that increased gene expression occurred as early as 4 hpi, whereas decreases in gene expression were less prominent at this time point and were generally detected in the later stages of the infection time course (12–20 hpi) (Figure 2B). Our longitudinal analysis demonstrated substantial increases in transcript abundance of innate genes, especially at 20 hpi for *Cxcl2* (174.8-fold), *Cxcl3* (30.9-fold), *Cxcl10* (16.5-fold), and *Ccl7* (7.0-fold) encoding chemokines (60, 61); *Il1a* (78.8-fold) and *Il1b* (48.3-fold) encoding interleukins (62, 63); as well as signaling molecules involved in induction of the IFN and genes induced by IFN (Figure 2B). Conversely, several genes involved in viral recognition and intracellular signaling were significantly downregulated over time including *TLR13* (5.3-fold), *TLR7* (3.4-fold) which encode TLRs (64, 65); *Il17rc* (4.2-fold), *Il1rl1* (2-fold), *Cxcr3* (13.6-fold), and *Cxcr5* (3.9-fold) encoding cytokine and chemokine receptors (66–68); and *Trim14* (2.3-fold), *Trim2* (2.6-fold), *Trim 68* (3.1-fold), *Trim47* (2.1-fold), and *Trim7* (3.0-fold) encoding members of the tripartite motif (Trim) family, which are important adaptor molecules of viral recognition receptors involved in activation and initiation of downstream intracellular signaling (Figure 2B) (69). Overall, we see that genes involved in the IFN response were highly upregulated with infection, while genes that encode proteins important for viral recognition displayed decreases in transcript abundance as infection progressed.

### qPCR Validates MNV-Induced Innate Gene Expression Changes

**Figure 3:**
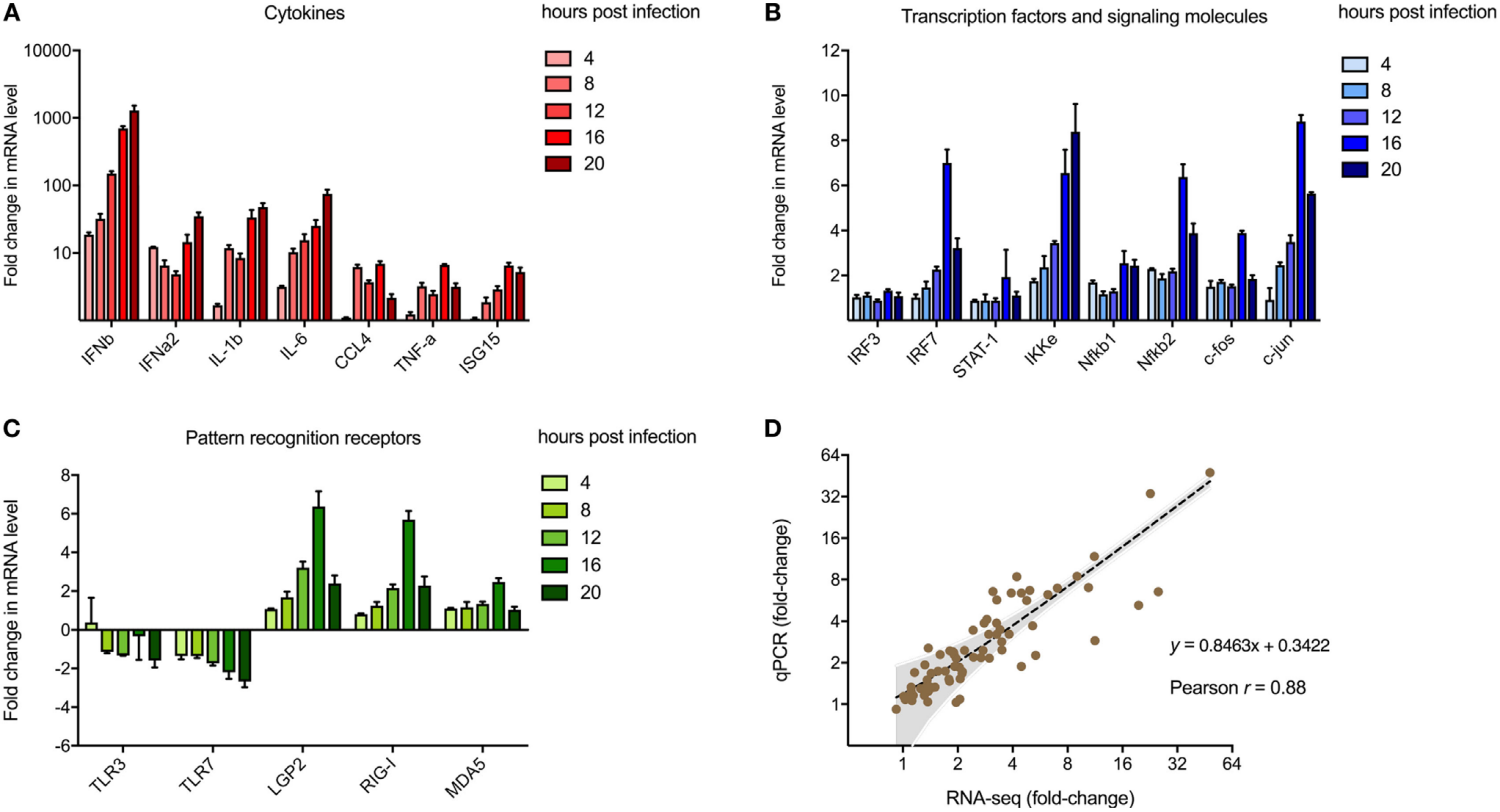
Validation of next-generation sequencing (NGS) analyses. Reverse transcription-quantitative polymerase chain reaction was performed to validate the use of NGS to quantify transcriptomic changes in murine norovirus (MNV)-infected cells. A total of 20 genes encoding **(A)** cytokines, **(B)** transcription factors, and **(C)** pathogen recognition receptors were screened for changes in expression with MNV infection over time. Fold changes (relative to mock) were calculated using the ΔΔ*C*_t_ method. **(D)** Changes in transcript abundance obtained from RNA-seq and qPCR were plotted for correlation analysis. The inverse fold changes for downregulated genes were used to generate a graph with positive correlation. Gray shading represents the 95% confidence interval for linear regression analysis (dotted black line, *r* = 0.88, *p*-value < 0.0001, Pearson correlation coefficient).

To validate transcriptomic changes detected from RNA sequencing during MNV infection over 20 h, 20 genes that encode key innate molecules of the antiviral response were probed for expression changes using qRT-PCR (Figures 3A–C). A steady increase in the level of gene expression was observed over time for genes encoding cytokines (Figure 3A), transcription factors and signaling molecules (Figure 3B) and PRRs, apart from TLR3 and TLR7 (Figure 3C). The most significant changes occurred in genes encoding cytokines (Figure 3A), with upward of 1,000-fold difference in expression recorded for IFN-β1 at 20 hpi with IFNα2, IL-1b, and IL-6 displaying >10-fold increase in transcript abundance at 20 hpi (Figure 3A). Increased expression changes for genes encoding transcription factors and PRRs were less prominent, although a gradual intensification of expression occurred over time for all genes analyzed (Figures 3A–C). A strong correlation existed between gene expression levels determined by RT-qPCR and NGS transcriptomic analysis (*r* = 0.88, *p*-value < 0.0001, Pearson coefficient) across all five time points of the longitudinal MNV infection, as demonstrated by linear regression analysis (Figure 3D).

### MNV Infection and Loxoribine Treatment Induce Distinct Expression Profiles in RAW264.7 Cells

**Figure 4:**
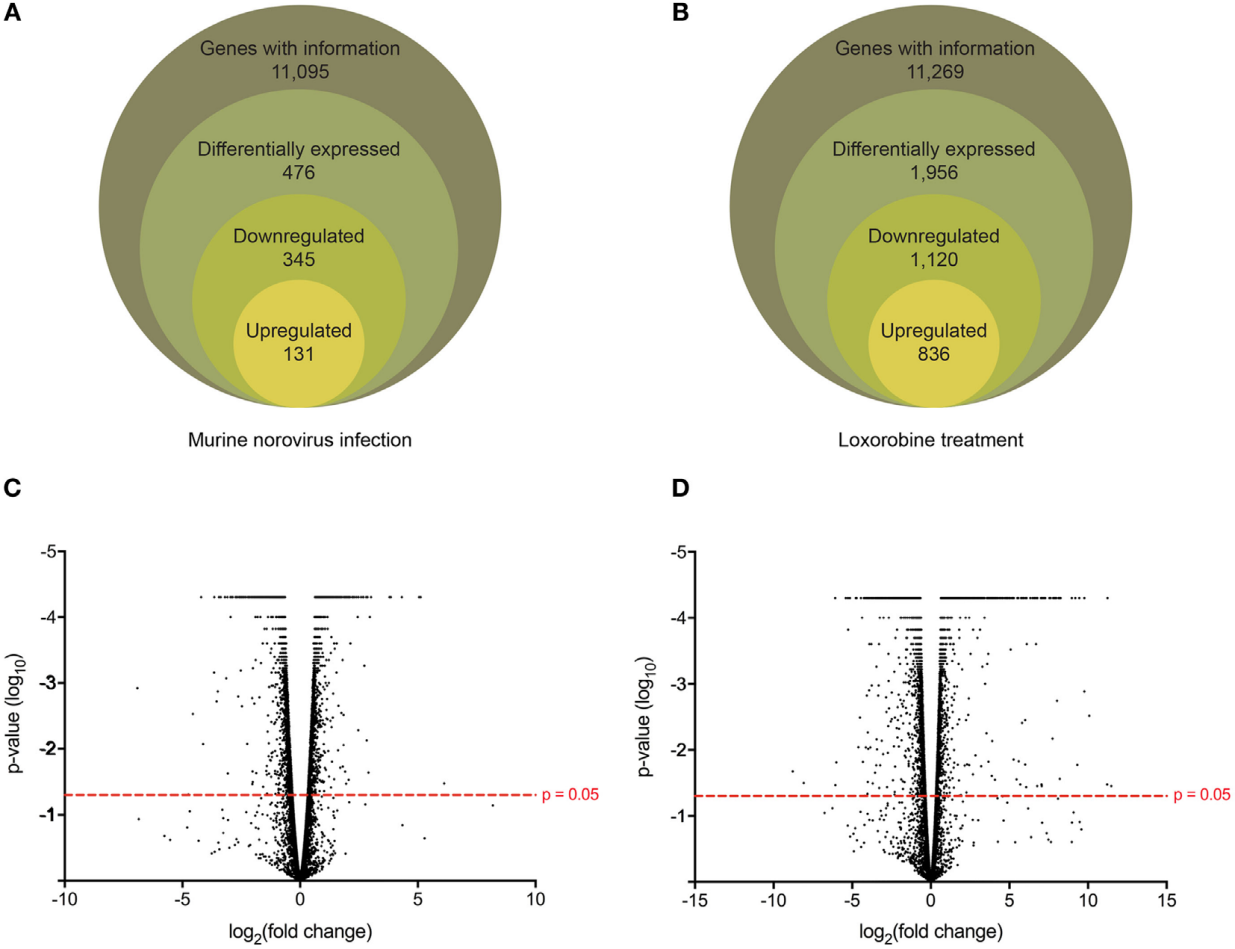
Gene expression profiling of murine norovirus (MNV)-infected and loxoribine-treated RAW264.7 cells. The global gene expression changes in RAW264.7 cells following 12 h of MNV infection **(A)** or loxoribine treatment **(B)** are illustrated schematically. Genes with expression information were further classified as differentially expressed based on the following stringencies: *q* value < 0.05, twofold or more change in transcript abundance and an FPKM value greater than 1 in at least one sample (mock or infection/treatment). The global distribution of gene expression in both MNV infection **(C)** and loxoribine treatment **(D)** is shown as a volcano plot with expression fold change plotted on the *x*-axis and significance on the *y*-axis. Genes significantly expressed are above the hashed red line (*p*-value < 0.05).

Following the initial time-course screen, we aimed to obtain a more detailed picture of host response changes induced by MNV infection, specifically at 12 hpi when infection is robust (Figure 1) (16). RNA sequencing of RAW264.7 cells infected with MNV or treated with loxoribine was performed and subsequent analysis yielded information on >11,000 genes (Figures 4A,B), and the expression profiles for both conditions, relative to mock, are presented in Figure 4. The transcriptome of macrophages infected with MNV had 476 genes significantly DE based on a *q*-value < 0.05 (FDR adjusted *p*-value), FPKM value >1 in at least one of the experimental conditions and a twofold or greater change in expression (Figure 4A) (Table S2 in Supplementary Material). Comparatively, loxoribine treatment yielded a greater increase in expression changes and a higher number of DEGs (*n* = 1,956) (Figure 4B) (Table S3 in Supplementary Material). Interestingly, both datasets contained significantly more downregulated genes than upregulated (Figures 4A,B). For MNV specifically, 345 genes were downregulated and 131 genes were upregulated (Figure 4A) and Tables 1 and 2 list the 25 genes with the greatest degree of change following infection for 12 h. A volcano plot distribution of altered expression induced by either MNV infection or loxoribine treatment is presented in Figures 4C,D. This illustrates the predominance of downregulated genes in both datasets, and a greater fold change in expression is noted for loxoribine treatment.

**Table 1:** Top 25 upregulated genes 12 hpi.

Gene ID	Description	Fold change
Lamc2	Laminin, gamma 2	34.77
Egr1	Early growth response 1	33.22
Cxcl2	Chemokine (C-X-C motif) ligand 2	19.94
Flrt3	Fibronectin leucine-rich transmembrane protein 3	14.47
Plk2	Polo-like kinase 2	13.90
Egr2	Early growth response 2	8.08
Il1a	Interleukin-1 alpha	7.78
Ccrl2	Chemokine (C–C motif) receptor-like 2	7.29
Kdm6b	KDM1 lysine (K)-specific demethylase 6B	7.00
Arc	Activity regulated cytoskeletal-associated protein	6.45
Mir155	microRNA 155	6.43
Irg1	Immunoresponsive gene 1	6.36
Phlda1	Pleckstrin homology like domain, family A, member 1	6.02
Rtp4	Receptor transporter protein 4	5.56
Bcl2a1a	B-cell leukemia/lymphoma 2 related protein A1a	5.55
Ccl7	Chemokine (C–C motif) ligand 7	5.46
Oas2	2′–5′ oligoadenylate synthetase 2	5.22
Olr1	Oxidized low density lipoprotein (lectin-like) receptor 1	4.94
Il7r	Interleukin-7 receptor	4.77
Arid5a	AT rich interactive domain 5A (MRF1-like)	4.68
Ifit1	Interferon-induced protein with tetratricopeptide repeats 1	4.67
Gem	GTP binding protein overexpressed in skeletal muscle	4.63
Ppp1r15a	Protein phosphatase 1, regulatory (inhibitor) subunit 15A	4.39
Tnfaip3	Tumor necrosis factor, alpha-induced protein 3	4.35
Myc	Myelocytomatosis oncogene	4.33

**Table 2:** Top 25 downregulated genes 12 hpi.

Gene ID	Description	Fold change
Hist1h2br	Histone cluster 1, H2br	−120.20
H2-Q10	Histocompatibility 2, Q region locus 10	−23.55
Hist1h2ad	Histone cluster 1, H2aD	−18.48
Dancr	Differentiation antagonizing non-protein coding RNA	−17.41
Ppia	Peptidylprolyl isomerase A	−12.62
Rps23	Ribosomal protein S23	−12.51
Snord89	Small nucleolar RNA, C/D box 89	−11.66
Snord22	Small nucleolar RNA, C/D box 22	−11.26
Hist1h3h	Histone cluster 1, H23h	−10.92
Cacng8	Calcium channel, voltage-dependent, gamma subunit 8	−10.88
Hist1h4n	Histone cluster 1, H4n	−10.37
Snora78	Small nucleolar RNA, H/ACA box 78	−9.17
Rps15a-ps4	Ribosomal protein 15a, pseudogene 4	−9.13
Rpl9	Ribosomal protein L9	−8.80
Hist2h2aa2	Histone cluster 2, H2aa2	−8.51
Dnaja1	DnaJ heat shock protein family (Hsp40) member A1	−7.79
Snora74a	Small nucleolar RNA, H/ACA box 74A	−7.75
Mir682	MicroRNA 682	−7.48
Rps10	Ribosomal protein S10	−7.26
Rps14	Ribosomal protein S14	−6.69
Oaz1	Ornithine decarboxylase antizyme 1	−6.65
Rpl36	Ribosomal protein L36	−6.51
Hist1h2bp	Histone cluster 1, H2bp	−6.38
Rps28	Ribosomal protein 28	−6.19
Tufm	Tu translation elongation factor, mitochondrial	−6.03

### A Robust Innate Response Is Mounted following MNV Infection for 12 h

**Figure 5:**
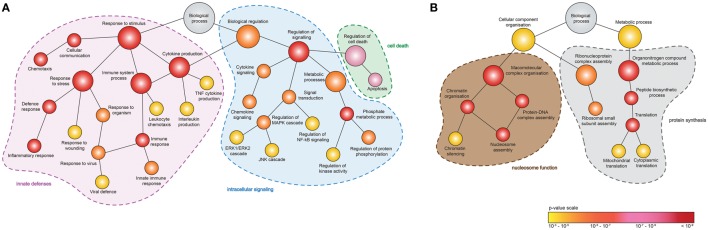
Enrichment analysis of genes differentially expressed following murine norovirus (MNV) infection. A hierarchical tree diagram of derived GOterms overrepresented by **(A)** upregulated and **(B)** downregulated differentially expressed genes (DEGs) (132 and 344 genes, respectively) is presented. Each circle node represents a biological process, and the colors depict the statistical significance of each process represented by MNV-induced DEGs.

To identify the function of DEGs induced by MNV 12 hpi (Figure 4A), ontology analyses were performed using GOrilla (Figures 5A,B) (Table S4 in Supplementary Material). Although despite the higher number of downregulated DEGs (*n* = 345) compared to upregulated DEGs (*n* = 131) (Figure 4A), fewer GOterms (*p*-value < 0.001) were represented by the downregulated DEGs (*n* = 90) relative to the upregulated DEGs (*n* = 392). Of the upregulated DEGs (*n* = 131), several biological functions were significantly overrepresented including the response to stimulus (GO:0050896) (*n* = 65/131), immune system process (GO:0002376) (*n* = 41/131), regulation of cytokine production (GO:0001817) (*n* = 22/131), regulation of apoptotic process (GO:0042981) (*n* = 25/131), and regulation of signaling (GO:0023051) (*n* = 42/131) (Figure 5A). Downregulated DEGs (345) were represented by GOterms related to ribonucleoprotein assembly (GO:0022618) (*n* = 11/344), nucleosome assembly (GO:0006334) (*n* = 22/344), and translation (GO:0006412) (*n* = 35/344), which were some of the most significant (Figure 5B) (Table S4 in Supplementary Material).

**Figure 6:**
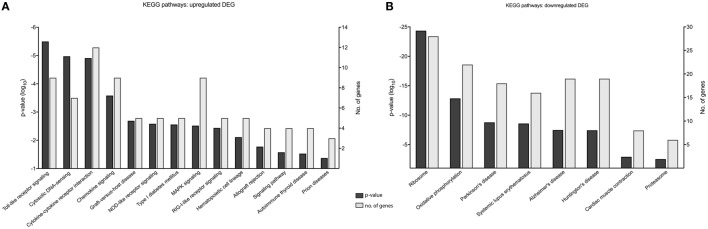
KEGG pathway analysis of murine norovirus (MNV)-induced differentially expressed genes (DEGs). KEGG pathways overrepresented by DEG in response to MNV infection were determined by submitting gene lists to the online bioinformatics database server, DAVID. **(A)** Upregulated and **(B)** downregulated gene lists were submitted individually to identify biological pathways altered by MNV and are listed the *x*-axes, *p*-value significance on the left *y*-axes and number of genes represented for each category on the right *y*-axes.

Gene enrichment analysis provided useful information on the function of DEGs, and to further our primary GOrilla analysis (Figure 5) (*n* = 476), we also performed KEGG pathway enrichment using DAVID to identify cellular pathways modified by MNV infection (Figure 6) (Table S5 in Supplementary Material). Consistent with our GOrilla analysis, genes upregulated by MNV infection (*n* = 131) (Figure 4A) had predominant involvement in immune signaling pathways including viral detection by TLRs (*n* = 9/131), NLRs (*n* = 5/131), RLRs (*n* = 5/131), MAPK signaling (*n* = 9/131), and cytokine–cytokine receptor interactions (*n* = 12/131) (Figure 6A). Analysis of downregulated genes (*n* = 345) (Figure 4A) revealed pathways involved in the function of the ribosome (*n* = 28/345), proteasome (*n* = 6/345), and oxidative phosphorylation (*n* = 22/345) as well as pathways known to be involved in several human pathologies including Alzheimer’s disease (*n* = 19/345), Huntington’s disease (*n* = 19/345), Parkinson’s disease (*n* = 18/345), and systemic lupus erythematosus (*n* = 16/345) (Figure 6B).

An essential feature of the innate response are the transmembrane receptors, such as TLRs and RLRs, that play an integral role in the detection of viruses and initiation of intracellular pathways (70). Members of the RLR family, including *DHX58* (LGP2) (1.89-fold), *DDX58* (RIG-I) (2.85-fold), and *IFIH1* (MDA5) (2.07-fold), had significantly increased gene expression at 12 hpi (Table S2 in Supplementary Material) (Figure 3C). Conversely, *TLR8* encoding a receptor for ssRNA recognition (71) and *TLR9* encoding the receptor for CpG sequences in DNA (72) were the only TLR members with a reportable increase in gene expression at 12 hpi (1.84- and 1.95-fold, respectively). Furthermore, both qPCR and RNA-seq read analysis of longitudinal infection (4–20 hpi) show that *TLR7*, which also encodes a receptor important for recognition of ssRNA (64), had modest downregulation (approximately threefold) as infection progressed (Figures 2B and 3C). Overall, we show that all RLR genes are upregulated with MNV infection; however, we report mixed expression of genes that encode TLRs.

### MNV-Specific Effects on the Host Response

**Figure 7:**
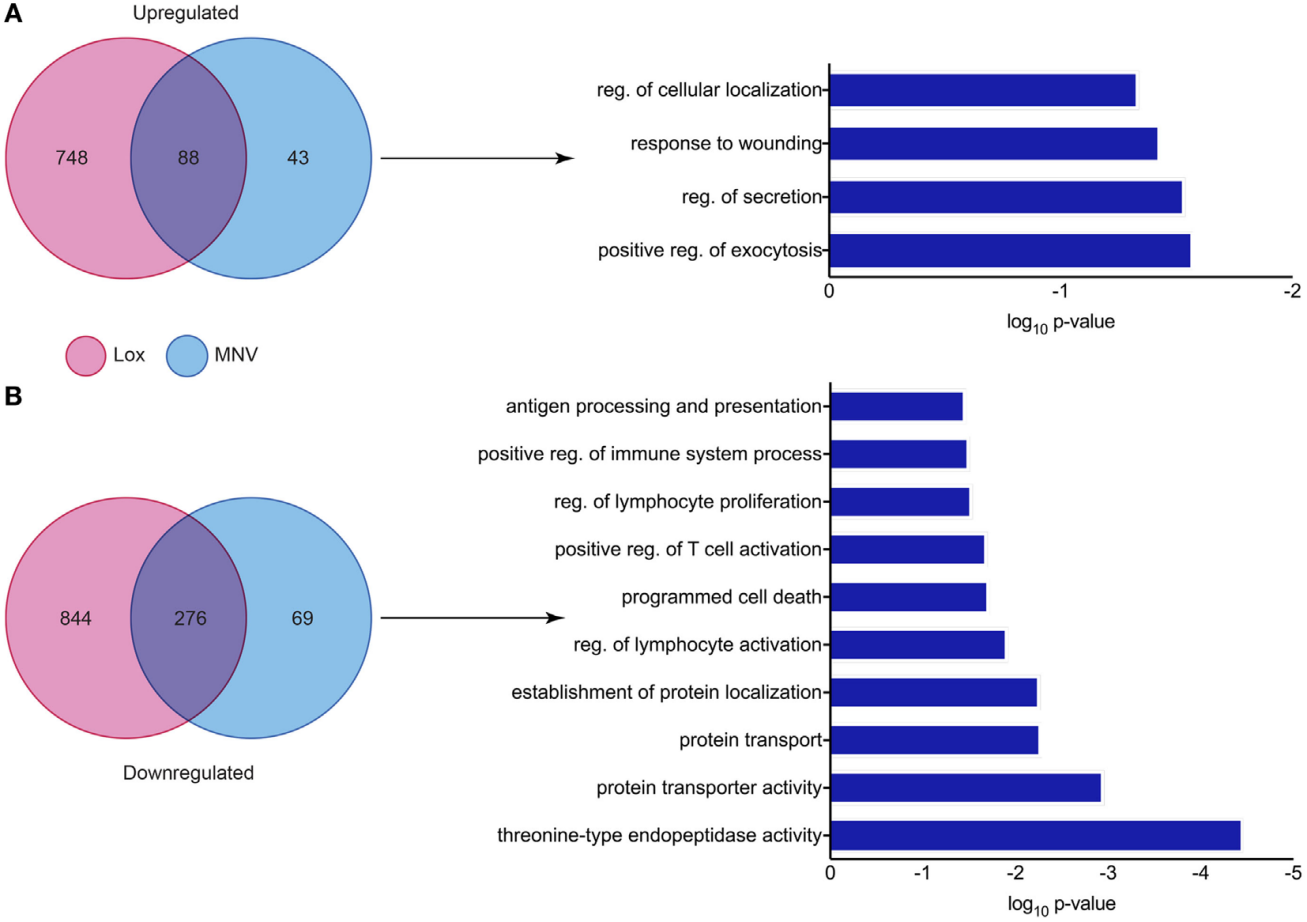
Comparative analysis of murine norovirus (MNV)- and loxoribine-induced differentially expressed genes (DEGs). Upregulated and downregulated DEGs from both MNV and loxoribine datasets were compared and are represented as Venn diagrams in panels **(A,B)**, respectively. DEGs induced by MNV only were further characterized by enrichment analysis using DAVID and GOterms (significance *p*-value < 0.05) and are represented as bar graphs.

Since MNV infection and TLR7 activation have been shown to induce an IFN response, a differential expression analysis of the two conditions revealed the genes that were less likely to be altered by innate stimulation. It is known that IFN can induce transcriptional changes in genes that are involved in innate antiviral defense (73–76) and our differential analyses aimed to identify transcriptional changes that are directly induced by viral infection. We performed a comparison of DEGs found in both the loxoribine treatment (TLR7 activation) and MNV infection datasets to delineate genes altered by the virus directly (Figure 7). Forty-three DEGs were upregulated (Figure 7A) and 69 DEGs were downregulated (Figure 7B) (Table S6 in Supplementary Material) solely by MNV infection. These revised gene lists (Table S6 in Supplementary Material) were submitted to DAVID for enrichment analysis to identify their biological functions (GOterms) (Table S7 in Supplementary Material). The most significant GOterms (*p*-value < 0.05) are depicted in Figure 7A for upregulated (*n* = 17) and Figure 7B for downregulated (*n* = 39) DEGs. Upregulated MNV-specific DEGs were enriched for functions including regulation of exocytosis (GO:0017157), regulation of cellular localization (GO:0060341), regulation of secretion (GO: 0051046), and GOterms related to the immune and defense responses. Conversely, GOterms generated from the MNV-specific downregulated DEGs were representative of protease activity (GO:0000502), protein transport (GO:0015031), and protein localization (GO:0034613). These biological functions align with GOterms also represented by the downregulated MNV-specific DEGs, involved in antigen presentation (GO:0019882) and immune cell activation (GO:0050863, GO:0002694, GO:0032944, and GO:0050670).

### MNV Affects Genes Involved in Immune Recognition

Closer examination of the 69 downregulated MNV-specific DEGs (Figure 7B) (Table S6 in Supplementary Material) revealed genes involved in several steps of MHC class I molecule maturation, including proteolysis and vesicular trafficking (Table S7 in Supplementary Material). Several examples include genes that encode beta catalytic subunits of the 26S proteasome including *Psmb3* (2.3-fold), *Psmb4* (2.5-fold), *Psmb5* (2.4-fold), and *Psmb9* (twofold) (Table S6 in Supplementary Material) that are important for the degradation of viral proteins to generate antigenic peptides (77, 78). *Psme2*, which was downregulated 2.5-fold, encodes the proteasome activator 28 subunit (Table S6 in Supplementary Material), an IFN-γ inducible protein that is involved in antigen processing by the immunoproteasome (i-proteasome) (79–82). Expression of *Ap1s1* was also decreased (2.2-fold) and encodes a subunit of adaptor protein 1 (Table S6 in Supplementary Material), a complex that mediates vesicular transport between the endoplasmic reticulum and the Golgi (83, 84). Furthermore, we observed a 24-fold reduction in the abundance of *H2-Q10* (Table S6 in Supplementary Material) mRNA, which encodes a secreted MHC class 1b molecule (Qa-10) involved in immune cell activation (85–87).

## Discussion

**Figure 8:**
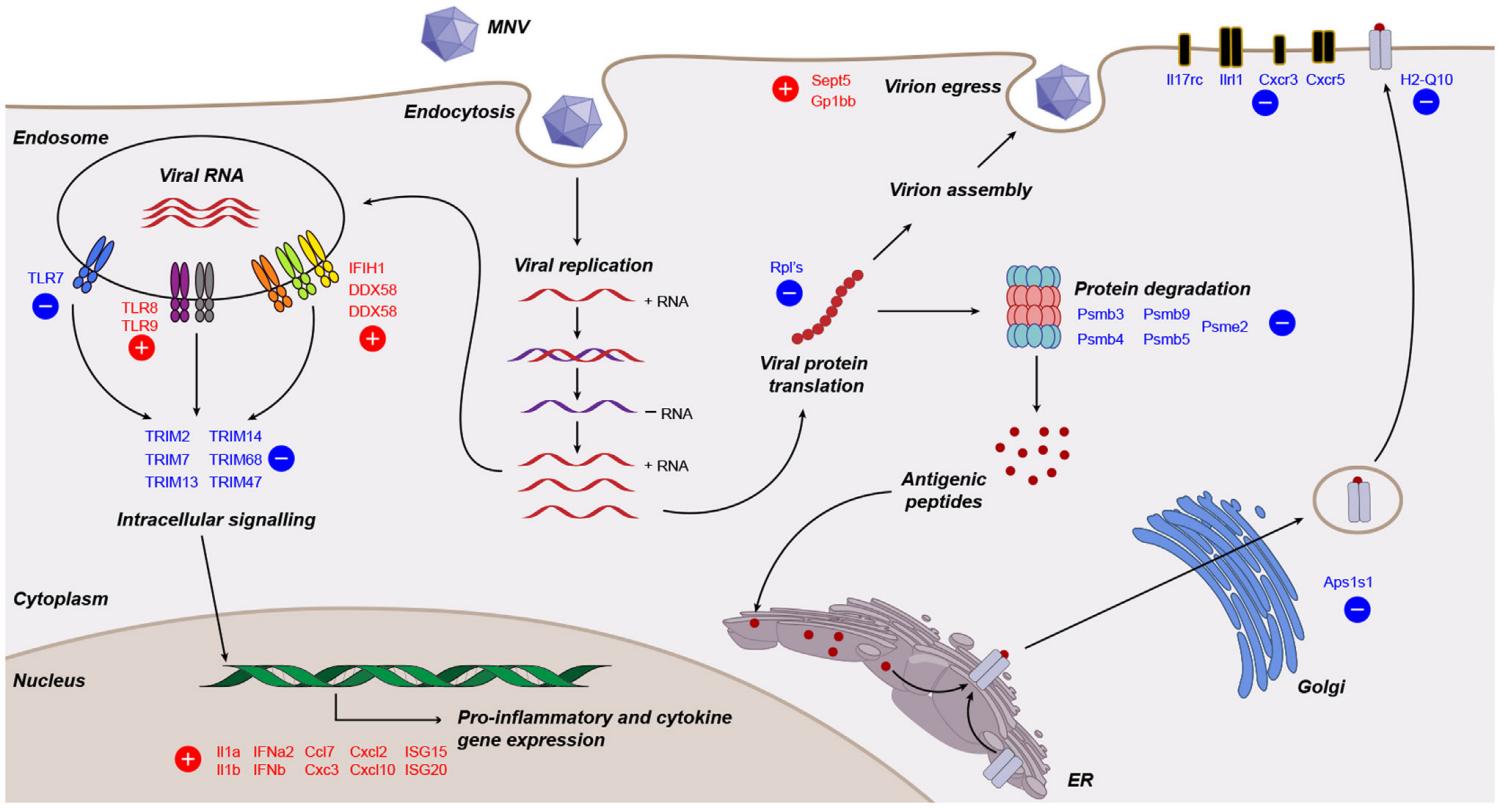
Working model of murine norovirus (MNV)-induced changes in the infected host. A summary of transcriptional changes characterized by RNA sequencing of MNV-infected cells is presented diagrammatically. Genes colored in red and blue are upregulated and downregulated, respectively.

Viruses need to overcome the innate response for continued replication, survival, and transmission. Knowledge of viral manipulation of the host can elucidate how immune evasion occurs, indicating potential pathways that contribute to pathogenesis. Previous studies investigating MNV infection have focused on individual receptors, signaling molecules, transcription factors or subsets of cytokines involved in innate immunity (32–36, 38), showing them to be important for MNV clearance. In the present study, we conducted a non-biased, broad transcriptomic analysis of the host response within MNV-infected macrophage cells and compared this with the response from TLR7 induction by loxoribine. This differential analysis allowed us to identify the subset of genes, whose expression is altered by MNV infection alone. We demonstrate that MNV perturbs the transcriptional profile of IFN signaling, viral recognition, cytokine stimulation, protein degradation, antigen presentation, and lymphocyte activation pathways (Figure 8).

Murine norovirus replicates within RAW264.7 macrophages to generate high titers of infectious virions (~1 × 10^7^ pfu/mL) (14), and the effectiveness of this system has made MNV a useful tool to understand the interplay between NoV and host biology. To confirm MNV replication over time (4–20 h), increases in genome levels were measured by qPCR (~60-fold) and NGS read mapping (>2 log-fold) (Figure 1). Several studies have previously shown the presence and increased quantity of the subgenomic species within MNV-infected cells, compared to full-length genomes (14, 88); however, the proportions of the different genomic species have not been characterized. Using NGS, we detected an average of 1.7-fold higher nucleotide coverage spanning ORF2-3 when compared to ORF1 (across all infection time points) (Figure 1E), which provided reliable confirmation of the existence of the MNV subgenomic RNA species. The increased abundance of the subgenomic RNA species likely plays an integral role in MNV replication and infection by providing more template for capsid production.

We also aimed to rule out the presence and replication of Abelson Mu-LV and Moloney Mu-LV, since both viruses have previously been shown to contaminate the RAW264.7 cell line (89). In contrast to the complete genome coverage of MNV (Figure 1E), there was no full-length genome coverage detected for Mu-LVs; over 92% of the genome was missing. Furthermore, the absence of increments in the small number of reads that did map to each genome over time (Table S9 in Supplementary Material) indicated no viral replication. These findings confirm the absence of both viruses and provide confidence that our analysis is based solely on MNV-induced cellular changes.

One of our main goals was to evaluate the transcript changes of innate immune genes (*n* = 280) in response to MNV infection (Figure 2B). Our longitudinal analyses illustrated that many of these innate genes had increased expression changes as infection advanced. Specifically, we found that genes encoding cytokines, interleukins, cellular transporters, and transcription factors were some of the most highly upregulated in early infection (Figure 2B). Conversely, downregulation of innate genes was also observed later in infection from 12 hpi onward. Several of these downregulated genes encoded TLRs, interleukin, and chemokine receptors (Figures 2B and 8), which are important for pathogen recognition and stimulation of the immune response (26, 27). Together these findings indicate that there is immediate viral recognition by the host and an early induction of the antiviral response. However, these data also suggest that MNV employs a strategy to reduce the available innate receptors, including those encoded by *TLR13, TLR7, Il17rc, Il1rl1, Cxcr3*, and *Cxcr5* (Figures 2B and 8) (66, 68, 69, 90), thereby reducing viral recognition and innate stimulation. We hypothesize that MNV induces these changes later in infection to dampen the host defenses for continued replication and cell-to-cell spread.

One limitation of our study is the use of the 10 mm reference genome, which is based on a mouse strain that differs from the origin of the RAW264.7 cell line. Previous work has revealed that this discrepancy may induce bias in read mapping and thus affect transcript quantitation (91). To confirm that our analyses were accurate, we validated transcript abundance by qRT-PCR of genes (*n* = 20) commonly involved in the innate response to viral infection (92–95) (Figures 3A–C). The robust correlation between both datasets (Figure 3D) demonstrated that our conclusions about the host response to MNV infection were not based on sequencing or read mapping bias. This qPCR analyses also allowed us to further investigate expression changes of PRRs with infection. We observed an increase (more than twofold) in expression of the RLR genes *IFIH1* (MDA5), *DDX58* (RIG-I) and *DHX58* (LGP2) (Figures 3C and 8), which corroborates previous work by McCartney et al. who showed MNV is recognized by the MDA5 receptor (33). These findings suggest that the RLRs play a predominant role in MNV recognition and are likely responsible for induction of the antiviral response seen in early infection (Figure 2B).

An important objective of our study was to interrogate the biological function of DEGs identified following MNV infection (12 h) to better understand the viral-host interaction. GOterm and KEGG pathway enrichment analysis (Figures 5 and 6) demonstrated that upon MNV infection, the host reacts by increasing the transcription of genes involved in the innate immune response, particularly those related to viral defense (Figure 5A). Obviously, this is not a surprise as most of the PRRs in the cytoplasm and endosome will encounter and engage with viral PAMPs (early in infection) to stimulate downstream signaling. However, what is apparent from our study is that MNV decreases the expression of the PRRs as the infection cycle continues (Figures 2B and 3C) and more importantly transcriptionally controls host cell translation machinery (Figures 5B and 8) and pathways involved in antigen presentation (Figures 7 and 8). Combined, these data imply a molecular mechanism to reduce the ability of an infected cell to present MNV antigens and thus provide ample opportunity for MNV to replicate and disseminate to neighboring cells.

To gain better resolution of the host biology modulated directly by MNV, we carried out a differential analysis of the transcriptomes generated by loxoribine (TLR7 agonist) treatment (Figure 4B) (Table S3 in Supplementary Material) and MNV infection (Figure 7). Since MNV infection and TLR7 activation induce an IFN response (34, 96), the resulting cellular changes likely alter the expression of many of the same genes. We detected a large proportion of genes common to both conditions (Figure 7), which emphasize that many gene expression changes observed in the initial analysis (Table S2 in Supplementary Material) are induced by IFN production (97, 98). Herein, we focused on the MNV-induced genes (Figure 7) with a view to characterize aspects of the host response modulated directly by the virus itself.

One compelling finding from this comparative analysis was the downregulation of several *Psmb* genes (*Psmb3, 4, 5*, and *9*) which encode components of the 26S proteasome catalytic core (99) (Figure 8). *Psbm9* encodes LMP2, one of the three proteins that replace the constitutive 26S proteasome catalytic subunits following immune stimulation to form the i-proteasome (79). The i-proteasome plays an essential role in the degradation of viral proteins for MHC class I antigen presentation and is induced by inflammatory mediators including IFN-γ (81, 100). In addition, the downregulation of *Psme2*, which encodes an activator of the i-proteasome, implies that MNV modulates the i-proteasome in a multifaceted manner to limit protein degradation for antigen presentation (Figure 8). Simply put, despite the generation of a robust innate immune response with MNV infection, the downregulation of genes involved in i-proteasome regulation hints toward immunological modulation by MNV to prevent MHC class I maturation. Further to this, we report the downregulation of *Ap1s1* (2.2-fold), encoding a protein, which has been shown to have a role in the trafficking and processing at the trans-Golgi network, a key step in the pathway taken by MHC class I molecules prior to integration into the plasma membrane (Figure 8). Previous work has demonstrated that RAW264.7 cells infected with MNV-1 show no increase in surface expression of MHC class I molecules up to 16 hpi (39), which is consistent with our hypothesis that MHC class I maturation is interfered through the downregulation of genes involved in proteasome function. Taken together, we can speculate that defects in protein trafficking and proteasome function (Figures 7B and 8) would be major drivers in MNV pathogenesis as an infected cell would have a limited capacity to communicate (*via* cytokines) and respond (*via* antiviral effectors such as ISGs) to the infection itself.

In summary, we believe that the described changes at the transcript level demonstrate the intricate nature by which MNV can regulate viral recognition (Figure 8) and it is reasonable to infer that such changes enable MNV to evade or dampen the immunological onset of the host response.

## Conclusion

We present a summary of the host gene expression changes induced by MNV infection in mouse macrophage cells. We show that a robust innate immune response is induced by MNV, which coincides with the disease manifestation known to accompany infection. Moreover, we have discovered that several elements of the host biology important to innate stimulation and immune recognition are directly affected by MNV infection. Overall, this study provides a novel source of global expression changes following MNV infection in an *in vitro* setting. However, given the richness of NGS, our findings represent only a small proportion of the possible analyses with significant potential for further bioinformatic mining. Our findings will likely benefit subsequent research into host–pathogen interactions, not just for MNV, but NoV in general.

## Author Contributions

DET, JM, and PW and drafted the experimental design. DET, NN, and JL performed the experiments and analyzed the data. DET, PW, and JM conceived the idea and shaped the structure of manuscript. DET, NN, JL, JM, and PW wrote the manuscript.

## Conflict of Interest Statement

The authors declare that the research was conducted in the absence of any commercial or financial relationships that could be construed as a potential conflict of interest.
