# Supplementary material for: RNA Sequencing of Murine Norovirus-Infected Cells Reveals Transcriptional Alteration of Genes Important to Viral Recognition and Antigen Presentation
Source: Front Immunol. 2017 Aug 11;8:959. doi: 10.3389/fimmu.2017.00959 (PMC5554501; doi:10.3389/fimmu.2017.00959)
Supplement: Supplementary file 1 [file Table_1.PDF]

TABLE S1 Differentially expressed genes following MNV infection over time. Genes were ranked by fold-change and considered differentially expressed if they had a 4-fold or greater change in transcript abundance, and FPKM value>1 in at least one sample.

		4 hpi		
**Gene ID**	**Chromosome**	**FPKM mock**	**FPKM mnv**	**log2(fold change)**
Hist1h2br	chr13:21806411-21810199	13.6614	0.143928	-6.56862
Cd27	chr6:125224211-125239934	11.3278	0.262633	-5.43068
Rpl13a	chr7:45125562-45128745	2506.43	603.607	-2.05395
Hist1h4j	chr13:21735095-21735407	1023.84	250.623	-2.0304
H2-Q10	chr17:35470088-35474563	0.113416	2.22707	4.29545
Rpl35	chr2:38998308-39005131	1.42745	12.7583	3.15992
Rpl11	chr4:136049947-136053371	0.159322	1.16725	2.87309
Rgs16	chr1:153740352-153745468	2.39008	13.6046	2.50896
Klhl35	chr7:99466003-99474020	0.228772	1.26154	2.4632
Irg1	chr14:103047011-103056573	1.29073	5.37163	2.05717
Fbxo15	chr18:84935024-84981392	0.28163	1.16987	2.05448
Serpine1	chr5:137061505-137072272	2.47684	10.0221	2.01661

		8 hpi		
**Gene ID**	**Chromosome**	**FPKM mock**	**FPKM mnv**	**log2(fold change)**
Hist1h2br	chr13:21806411-21810199	13.6614	0.117251	-6.86436
Hist1h2ao	chr13:21833023-21833575	23.3974	1.15272	-4.34323
Gm6525	chr3:84160438-84304775	1.61045	0.177856	-3.17868
Hist2h3c1	chr3:96246684-96247348	2.13215	0.309206	-2.78567
Rps28	chr17:33823036-33824498	8.43931	1.43158	-2.55952
Snord32a	chr7:45125562-45128745	1765.19	352.093	-2.32579
Cxcl2	chr5:90903898-90905938	0.48828	23.1196	5.56527
Egr1	chr18:34861206-34864956	0.612599	16.8859	4.78473
Il1a	chr2:129297369-129309972	0.0674848	1.10486	4.03316
Egr3	chr14:70077444-70082613	0.270464	3.9582	3.87133
Ccl7	chr11:82045711-82047523	0.651802	8.61858	3.72495
Chac1	chr2:119351241-119354327	1.96967	23.2081	3.5586
Rpl35	chr2:38998308-39005131	1.42745	16.3812	3.52053
Ccl2	chr11:82035576-82037452	14.375	163.729	3.50967
Il1b	chr2:129364579-129375733	1.67739	18.8109	3.48728
Egr2	chr10:67537868-67542188	1.65276	17.7885	3.42799
Myc	chr15:61985340-61990361	0.358124	3.69931	3.36872
Arc	chr15:74669080-74672570	0.809876	8.21605	3.34267
Rpl11	chr4:136049947-136053371	0.159322	1.10277	2.79112
Ptgs2	chr1:150100123-150108012	2.28432	15.7079	2.78166
Irg1	chr14:103047011-103056573	1.29073	8.73583	2.75876
Ccl4	chr11:83662583-83664683	18.4889	115.116	2.63836
Rgs16	chr1:153740352-153745468	2.39008	14.2241	2.57321
Phlda1	chr10:111506285-111508649	0.470448	2.79076	2.56855
Rgs1	chr1:144244668-144249104	15.8682	92.9858	2.55087
Snord55	chr4:117153835-117156132	417.638	2445.87	2.55002
1700012D01Rik	chr10:127667122-127668851	0.632236	3.65957	2.53314
Gm6377	chrX:109196755-109200445	0.607738	3.47858	2.51698
Plk2	chr13:110395043-110400843	2.8488	16.2797	2.51465
Ddit3	chr10:127290792-127311786	3.69599	20.7008	2.48565
Ndufa4l2	chr10:127514938-127522444	0.203786	1.13128	2.47282
Flrt3	chr2:140395429-142390050	0.338188	1.79902	2.41132
Trib3	chr2:152337424-152344060	2.08736	11.0559	2.40507
F3	chr3:121723536-121735052	0.209254	1.0788	2.3661
Gadd45a	chr6:67035095-67080652	0.374005	1.82653	2.28797
Pdcd1	chr1:94038304-94052553	3.70581	17.2606	2.21962
Maff	chr15:79346620-79359076	0.827744	3.83629	2.21245
Cxcl10	chr5:92331840-92414627	0.764427	3.53379	2.20877
Ccl3	chr11:83647842-83649378	61.429	282.984	2.20373
Gmfg	chr7:28437446-28446895	0.233483	1.06262	2.18624
Rcan1	chr16:92391950-92466169	16.5958	74.1784	2.16018
Isg15	chr4:156199423-156200818	0.260008	1.16091	2.15863

Ppp1r15a	chr7:45473562-45526268	3.24638	14.401	2.14927
Serpina3f,Serpina3g	chr12:104214543-104241934	0.424919	1.86661	2.13516
Tctex1d4	chr4:117126812-117133952	0.297724	1.2994	2.12579
Arid5a	chr1:36307732-36324029	0.43073	1.87407	2.12132
Ifi202b	chr1:173962568-173982844	0.24258	1.04827	2.11148
Gm4432	chr17:32196271-32284133	0.280095	1.20389	2.10371
6330407A03Rik	chr4:3678120-3791612	0.302821	1.29988	2.10184
Cth	chr3:157894247-157925063	1.29108	5.53889	2.10102
Magix	chrX:7673165-7681251	0.24085	1.02355	2.08737
Ier2	chr8:84661330-84662852	16.6966	69.6788	2.06117
Spry4	chr18:38586264-38601268	0.277842	1.14233	2.03964
Cdkn2b	chr4:89306288-89311032	1.34421	5.48301	2.02821

		12 hpi		
**Gene ID**	**Chromosome**	**FPKM mock**	**FPKM mnv**	**log2(fold change)**
Cd27	chr6:125224211-125239934	11.3278	0.196321	-5.85051
Hist1h2bq	chr13:21833742-21837530	2.41313	0.0440544	-5.77547
Hist1h2ao	chr13:21833023-21833575	23.3974	0.438163	-5.73874
Rhox5	chrX:37754607-37808878	13.0667	1.25123	-3.38448
Prss46	chr9:110844505-110856522	1.41269	0.21915	-2.68846
Atp5l	chr9:44913247-44920742	1.48823	0.241159	-2.62555
D330023K18Rik	chr2:31151048-31152291	3.156	0.516399	-2.61154
Kazald1	chr19:45076138-45079289	1.10654	0.192439	-2.52359
Rps13	chr7:116331506-116334190	0.909264	0.204624	-2.15172
Pacrg	chr17:10403011-10840191	1.80149	0.409655	-2.13671
Mir1843a,Scarna3b	chr12:80335846-80436601	64.9243	15.2278	-2.09205
Rab4a	chr8:123805995-123835291	3.32653	0.794179	-2.06648
Cxcl2	chr5:90903898-90905938	0.48828	58.786	6.91162
Dusp8	chr7:142079486-142095284	0.0194681	1.45394	6.22271
Egr1	chr18:34861206-34864956	0.612599	32.1811	5.71513
Rtp4	chr16:23609918-23614222	0.183699	4.40741	4.58451
Il1a	chr2:129297369-129309972	0.0674848	1.52491	4.49802
Wnt10a	chr1:74792018-74804175	0.057821	1.11504	4.26936
Rpl35	chr2:38998308-39005131	1.42745	21.7252	3.92785
Phlda1	chr10:111506285-111508649	0.470448	5.69722	3.59815
Isg15	chr4:156199423-156200818	0.260008	2.94054	3.49946
Maff	chr15:79346620-79359076	0.827744	9.30243	3.49035
Arc	chr15:74669080-74672570	0.809876	9.03905	3.4804
Plk2	chr13:110395043-110400843	2.8488	30.1252	3.40255
Upp1	chr11:9118007-9136170	0.135638	1.36096	3.32679
Gdf15	chr8:70629393-70631635	0.748633	7.33217	3.29191
Ppp1r15a	chr7:45473562-45526268	3.24638	31.0622	3.25826
Tnfrsf9	chr4:150920154-150946102	0.205692	1.9071	3.21283
Spaca4	chr7:45721219-45729492	0.147069	1.33365	3.18082
Gem	chr4:11704446-11714993	0.113238	1.02575	3.17925
Il1b	chr2:129364579-129375733	1.67739	15.1056	3.17079
Socs1	chr16:10783808-10785536	0.277162	2.45883	3.14917
Irg1	chr14:103047011-103056573	1.29073	10.5923	3.03677
Ifi202b	chr1:173962568-173982844	0.24258	1.95842	3.01316
Ccrl2	chr9:111054833-111057518	0.392938	3.132	2.99471
Cxcl10	chr5:92331840-92414627	0.764427	5.94824	2.96001
F3	chr3:121723536-121735052	0.209254	1.60943	2.94322
Gadd45a	chr6:67035095-67080652	0.374005	2.79625	2.90236
Gimap6	chr6:48701582-48708244	0.294112	2.17674	2.88773
Egr4	chr6:85511121-85513542	0.1544	1.13586	2.87904
Rpl11	chr4:136049947-136053371	0.159322	1.08308	2.76512
Myc	chr15:61985340-61990361	0.358124	2.4261	2.76011
Klk1b27	chr7:44052289-44056711	0.178711	1.20836	2.75735
Hbegf	chr18:36504926-36515805	0.325394	2.12581	2.70776
1700012D01Rik	chr10:127667122-127668851	0.632236	4.09355	2.69482
Arid5a	chr1:36307732-36324029	0.43073	2.72773	2.66284
Vgf	chr5:137030294-137033351	0.188166	1.11664	2.56908
Serpina3f,Serpina3g	chr12:104214543-104241934	0.424919	2.4847	2.54781
Mtag2	chr7:45339125-45370564	0.200797	1.15176	2.52003

S100a8	chr3:90669070-90670034	0.880675	4.98327	2.50041
Mcemp1	chr8:3665761-3668905	0.62542	3.52931	2.49649
Hist1h1t	chr13:23695810-23696545	0.257579	1.43999	2.48297
Saa3	chr7:46711997-46715676	61.0969	341.084	2.48096
Map1b	chr13:99421463-99516602	0.197237	1.06064	2.42693
Mir155,Mir155hg	chr16:84713022-84715244	0.452988	2.43083	2.4239
Tnfaip3	chr10:19000909-19015410	2.46602	13.2292	2.42347
Irf7	chr7:141263182-141266424	1.17635	6.29335	2.41952
Lppos	chr16:24392555-24992578	0.219257	1.16897	2.41454
Ccl4	chr11:83662583-83664683	18.4889	95.29	2.36567
Cd244	chr1:171559192-171585316	0.5371	2.76406	2.36353
Egr2	chr10:67537868-67542188	1.65276	8.46672	2.35693
C030037D09Rik	chr11:88718642-88728572	0.199591	1.01898	2.352
Oas2	chr5:120730332-120749848	0.740669	3.75776	2.34297
Kdm6b	chr11:69398517-69413675	0.753868	3.78822	2.32914
Rpl17	chr18:75000476-75003381	2.09626	10.4374	2.31587
Rgs1	chr1:144244668-144249104	15.8682	77.8824	2.29516
Cdc42ep2	chr19:5917555-5924816	1.33322	6.37846	2.25829
Ifi44	chr3:151730922-151749959	0.567002	2.68618	2.24413
Ccl2	chr11:82035576-82037452	14.375	67.9779	2.2415
Aqp9	chr9:71110658-71163289	0.395557	1.86689	2.23868
Atf3	chr1:191170296-191183333	12.2934	57.7457	2.23183
Cth	chr3:157894247-157925063	1.29108	5.899	2.19189
4930445N18Rik	chr18:82855896-82859162	0.285867	1.30037	2.1855
Ccl7	chr11:82045711-82047523	0.651802	2.94194	2.17426
Cd40	chr2:165055635-165071654	1.54388	6.95794	2.1721
Nfkbiz	chr16:55811376-55838641	2.83807	12.6782	2.15937
Cdkn2b	chr4:89306288-89311032	1.34421	5.93768	2.14314
Rps27a	chr11:29545841-29578352	4.6656	20.0734	2.10515
Clec4n	chr6:123229842-123247024	0.466675	1.99169	2.0935
Dusp1	chr17:26505590-26508472	3.50885	14.9172	2.0879
Ifi44l	chr3:151758736-151762891	0.254067	1.07857	2.08584
E230013L22Rik	chr8:11453976-11480241	0.539183	2.23636	2.05231
4930577N17Rik	chr3:51252719-51340644	0.480167	1.98819	2.04985
Fbxo15	chr18:84935024-84981392	0.28163	1.16396	2.04717
Ier3	chr17:35821712-35822911	39.0756	160.069	2.03435
Lcn2	chr2:32384636-32387739	0.978368	3.99525	2.02984
Xaf1	chr11:72301628-72313733	0.324502	1.31017	2.01345
Fjx1	chr2:102449365-102451792	0.668995	2.69965	2.01271

		**16 hpi**		
**Gene ID**	**Chromosome**	**FPKM mock**	**FPKM mnv**	**log2(fold change)**
Rhox5	chrX:37754607-37808878	13.0667	0.27223	-5.58492
Hist1h2ao	chr13:21833023-21833575	23.3974	0.49483	-5.56327
Cd27	chr6:125224211-125239934	11.3278	0.417642	-4.76145
Xlr	chrX:53777117-53797706	11.7172	0.539214	-4.44162
Pax6	chr2:105536079-105904564	4.64036	0.311374	-3.89752
Peg12	chr7:62461870-62464510	3.85542	0.271776	-3.8264
Fam13c	chr10:70440667-70599291	4.39255	0.354762	-3.63013
Slc16a12	chr19:34668405-34747111	36.3251	3.08517	-3.55754
4930461G14Rik	chr9:58455172-58469623	1.36487	0.12302	-3.4718
Pacrg	chr17:10403011-10840191	1.80149	0.164813	-3.45029
H2-Q1	chr17:35320557-35325099	1.11784	0.104934	-3.41315
S1pr1	chr3:115710432-115715055	11.0323	1.05836	-3.38182
Cd48	chr1:171682054-171705257	1.60238	0.1648	-3.28142
Ndrg2	chr14:51905270-51913488	80.0374	8.39711	-3.25271
Zfp229	chr17:21733723-21748969	1.62452	0.183488	-3.14626
Cldn11	chr3:31149919-31164326	17.9223	2.11879	-3.08045
Crip1	chr12:113152011-113153879	288.484	34.7047	-3.05529
Nudt7	chr8:114133573-114152312	4.9476	0.598649	-3.04695
Gm16617	chr14:51984832-52020733	1.42895	0.178425	-3.00156
Cd109	chr9:78615545-78716260	4.89268	0.612922	-2.99685
Zfp760	chr17:21707740-21725636	1.84899	0.239614	-2.94795
Ambp	chr4:63143278-63154142	2.59156	0.336968	-2.94314

Nudt12	chr17:59001400-59013322	1.46294	0.190404	-2.94173
Psma8	chr18:14706150-14762299	9.77988	1.27322	-2.94134
Atp6v0e2	chr6:48537568-48541800	12.8804	1.71895	-2.90557
Clu	chr14:65968482-65981545	10.2513	1.37965	-2.89343
2510009E07Rik	chr16:21649044-21694665	28.5107	3.895	-2.87181
Myom1	chr17:71019556-71126856	1.47385	0.204595	-2.84874
Odf3l1	chr9:56848658-56851963	2.11692	0.296439	-2.83616
Tcp11x2	chrX:135654697-135693790	1.6803	0.239066	-2.81324
Mettl21b	chr10:127032904-127041394	1.32557	0.197418	-2.74729
Ltc4s	chr11:50236471-50238471	7.58154	1.13127	-2.74455
Fut10	chr8:31187330-31261924	3.7876	0.56756	-2.73844
Cadm1	chr9:47530351-47853385	83.0574	12.5019	-2.73196
Slc16a7	chr10:125227484-125328535	4.52311	0.689406	-2.71389
Car5b	chrX:163976821-164028010	8.37571	1.2791	-2.71108
As3mt	chr19:46707442-46741095	16.4797	2.54096	-2.69724
Rarb	chr14:16430839-17082331	3.32886	0.515425	-2.69119
Tcaf1	chr6:42672546-42693059	3.46394	0.544134	-2.67038
Msr1	chr8:39467447-40227787	74.4606	11.7107	-2.66865
Rcn1	chr2:105385947-105399319	9.92673	1.5887	-2.64347
Acaa1b	chr9:119148042-119157093	4.41343	0.709557	-2.63691
Cntln	chr4:84884308-85131921	5.0988	0.839936	-2.60181
Car2	chr3:14886425-14900770	502.674	83.2253	-2.59453
Kitl	chr10:100015823-100100412	57.5157	9.52316	-2.59444
Alox5	chr6:116410070-116461178	11.2103	1.85976	-2.59164
Cbr1	chr16:93607836-93610349	6.93819	1.15147	-2.59108
Npl	chr1:153503015-153549714	15.4348	2.56447	-2.58945
Ndrg4	chr8:95703036-95715119	25.6425	4.2616	-2.58907
Mtus2	chr5:147957319-148316065	1.17939	0.197066	-2.58129
Rasgrp3	chr17:75435904-75529053	74.9304	12.6195	-2.5699
Tom1l1	chr11:90638183-90687601	12.5531	2.12852	-2.56012
Eif2s3y	chrY:1010611-1028598	22.3326	3.83041	-2.54358
Suz12	chr11:79993105-80034123	39.2403	6.80359	-2.52797
Dmrt2	chr19:25672410-25678991	2.35216	0.408937	-2.52404
Ly86	chr13:37345344-37419036	91.1121	15.8868	-2.51981
Rras2	chr7:114046781-114117781	17.9493	3.13204	-2.51875
3830403N18Rik	chrX:56136571-56153496	5.1413	0.901513	-2.51171
Prss50	chr9:110857966-110864628	26.9237	4.72255	-2.51124
Arhgap24	chr5:102481390-102897937	5.42646	0.961173	-2.49714
2610203C22Rik	chr1:9548045-9631092	18.8984	3.36307	-2.49041
Fads2	chr19:10064163-10101503	81.4266	14.7117	-2.46854
Atp6v0d2	chr4:19876837-19922566	7.76066	1.40641	-2.46416
Gtpbp10	chr5:5537456-5559501	2.42018	0.440591	-2.4576
Prss46	chr9:110844505-110856522	1.41269	0.257727	-2.45453
Cpne8	chr15:90487480-90679388	15.7201	2.89489	-2.44103
Plcb4	chr2:135741829-136013068	22.2428	4.09782	-2.44041
Car9	chr4:43507025-43513725	2.71111	0.499809	-2.43943
Zfp40	chr17:23173868-23193228	1.929	0.356214	-2.43704
Clec4a2	chr6:123122689-123143999	4.01376	0.743373	-2.43279
Cox7a1	chr7:30184170-30186030	4.98065	0.923019	-2.4319
Ddx3y	chrY:1260714-1286613	12.7649	2.39693	-2.41292
Ttc12	chr9:49436960-49486225	4.79696	0.909314	-2.39927
Serinc2	chr4:130253496-130275586	2.13064	0.404165	-2.39827
Lpin3	chr2:160880669-160906000	2.24199	0.429341	-2.38459
Bdh2	chr3:135281220-135304425	4.35832	0.841323	-2.37304
Rps19-ps3	chr4:147821776-147822202	3.37431	0.654589	-2.36593
Flot2	chr11:78037940-78060432	9.65509	1.88113	-2.35969
Havcr2	chr11:46193848-46589232	7.8381	1.52964	-2.35732
Syngr1	chr15:80091333-80119501	47.5282	9.31899	-2.35054
Abcb4	chr5:8893720-8959226	16.836	3.32184	-2.3415
Mgmt	chr7:136894610-137128188	5.10774	1.01159	-2.33606
Gpr176	chr2:118277097-118373419	4.71304	0.943557	-2.32048
Hmgn5	chrX:109004536-109013380	5.2753	1.06205	-2.3124
Cadps	chr14:12372562-12823079	2.98628	0.601575	-2.31153
Fam117a	chr11:95337017-95381872	3.50442	0.709087	-2.30514
Slc43a3	chr2:84936645-84958509	11.1774	2.28155	-2.2925

Anxa1	chr19:20373433-20390671	120.567	24.8583	-2.27804
Chchd10	chr10:75935572-75940672	20.7492	4.28706	-2.275
Emp1	chr6:135362930-135383173	52.6732	10.9102	-2.2714
Pls3	chrX:75785653-75875170	48.9877	10.1727	-2.26772
Chst10	chr1:38863872-38898160	16.5977	3.46442	-2.26029
Gpnmb	chr6:49036517-49058182	158.135	33.1198	-2.25539
Ank	chr15:27466676-27594907	226.284	47.518	-2.25159
Gm11127	chr17:36042960-36058645	4.99827	1.05549	-2.24351
Tmlhe	chrX_GL456233_random:15964	4.17281	0.882733	-2.24097
Dsel	chr1:111858701-111864918	2.99529	0.635312	-2.23716
Them6	chr15:74721233-74724373	29.8771	6.33831	-2.23687
Tmem141	chr2:25620065-25622005	22.3168	4.74	-2.23517
Trp53i11	chr2:93187583-93201757	2.17447	0.465033	-2.22526
Klf8	chrX:153238044-153396134	1.55877	0.335342	-2.2167
Panx1	chr9:15005784-15045478	9.81554	2.11594	-2.21377
Hpgds	chr6:65117292-65144730	14.8731	3.211	-2.21161
Uty	chrY:1097143-1245738	4.63176	1.00767	-2.20053
Olfr933	chr9:38975677-38976604	2.78754	0.607849	-2.19721
Ang,Rnase4	chr14:51091076-51106151	12.6633	2.77311	-2.19108
Sash3	chrX:48146526-48161563	2.76114	0.604705	-2.19096
Rpl3l	chr17:24727828-24736149	1.00937	0.224394	-2.16935
Cd24a	chr10:43579168-43584265	2.99431	0.665788	-2.16909
Zfp345	chr2:150470990-150485063	1.11668	0.248313	-2.16898
Fxyd2	chr9:45399708-45410278	254.811	56.6801	-2.16851
Sh3bgrl	chrX:109095406-109162467	116.536	26.0249	-2.16281
Fnbp1l	chr3:122538718-122619714	23.2034	5.1974	-2.15847
Akr1b8	chr6:34354163-34368454	62.5105	14.038	-2.15476
Serpinb6b	chr13:32965512-32979037	12.1603	2.73198	-2.15416
Tmsb15b1,Tmsb15b2	chrX:136954987-136976874	1.24476	0.280077	-2.15197
Hexb	chr13:97137936-97198357	133.125	29.9906	-2.1502
Zfp667	chr7:6286579-6307883	1.04739	0.237354	-2.14169
Kbtbd11	chr8:15011024-15033332	35.2639	8.01742	-2.13698
9330111N05Rik	chr13:80964073-81079857	1.29774	0.295123	-2.13662
G730013B05Rik	chr16:50526244-50559459	19.5495	4.45876	-2.13242
Cxcr3	chrX:101731534-101734147	9.04243	2.06631	-2.12965
Kdelc2	chr9:53384022-53401867	12.3325	2.84596	-2.11548
Tshz3	chr7:36698117-36773457	3.53769	0.817428	-2.11364
Sdsl	chr5:120458201-120472763	3.05635	0.708716	-2.10853
Hoxb6	chr11:96291036-96306910	1.44777	0.337941	-2.09899
Kif17	chr4:138262250-138301973	1.04616	0.245744	-2.08987
Klhl41	chr2:69670119-69684239	4.14137	0.977848	-2.08242
Hoxb7	chr11:96286645-96290163	8.86833	2.09803	-2.07963
Dhcr24	chr4:106561037-106589113	16.8875	3.99687	-2.07902
Nradd	chr9:110621134-110624393	4.91188	1.17719	-2.06093
AF251705	chr11:114996768-115001880	12.3051	2.95653	-2.05728
1300002E11Rik	chr16:21794346-21809039	1.91207	0.45977	-2.05615
Amica1	chr9:45079182-45135606	6.06545	1.46969	-2.0451
Mycn	chr12:12936092-12941836	197.501	47.9231	-2.04307
Tcf4	chr18:69344491-69687967	13.256	3.23116	-2.03652
2700099C18Rik	chr17:94750099-94834799	5.211	1.27142	-2.03512
Ak1	chr2:32621757-32635058	1.91714	0.471362	-2.02404
A930024E05Rik	chr5:122989353-122998341	1.22327	0.301069	-2.02258
Prtg	chr9:72807273-72917307	4.51026	1.11323	-2.01846
Angptl2	chr2:33133418-33371494	58.2747	14.3967	-2.01713
Ddx43	chr9:78395776-78423589	2.73469	0.675645	-2.01704
Tm7sf3	chr6:146602275-146634592	14.4978	3.59339	-2.01241
Fam198b	chr3:79885929-79946278	1.97699	0.491085	-2.00926
Clec12a	chr6:129350243-129365303	34.0471	8.49687	-2.00253
Apobec1	chr6:122577791-122602444	54.3264	13.5627	-2.00201
Agmo	chr12:37241638-37581932	10.2134	2.55034	-2.0017
Cxcl2	chr5:90903898-90905938	0.48828	87.8891	7.49183
Mx2	chr16:97536080-97560901	0.0280524	4.74158	7.4011
Rsad2	chr12:26442742-26456452	0.0191468	2.44853	6.99867
Dusp8	chr7:142079486-142095284	0.0194681	1.93978	6.63864
Egr1	chr18:34861206-34864956	0.612599	42.2239	6.10697

Rtp4	chr16:23609918-23614222	0.183699	8.23175	5.48578
Il1a	chr2:129297369-129309972	0.0674848	1.99905	4.88861
Isg15	chr4:156199423-156200818	0.260008	6.54845	4.65452
Kctd19	chr8:105375380-105413502	0.0913889	2.18151	4.57717
Il1b	chr2:129364579-129375733	1.67739	38.1888	4.50886
Tal2	chr4:53779704-53786885	0.0660032	1.33822	4.34164
Fpr2	chr17:17887823-17893952	0.0683414	1.22258	4.16102
Plk2	chr13:110395043-110400843	2.8488	50.8539	4.15793
Oasl2	chr5:114896933-114912245	0.0861845	1.48977	4.11152
Ccrl2	chr9:111054833-111057518	0.392938	6.62124	4.07473
Hist1h4n	chr13:21831792-21832158	11.4457	189.517	4.04945
9430065F17Rik	chr13:67553201-67570892	0.0735591	1.19486	4.02179
Phlda1	chr10:111506285-111508649	0.470448	7.38494	3.97248
Maff	chr15:79346620-79359076	0.827744	12.676	3.93677
Hspa1b	chr17:34956428-34959238	0.112875	1.71376	3.92437
Lcn2	chr2:32384636-32387739	0.978368	14.351	3.87463
Cxcl10	chr5:92331840-92414627	0.764427	10.8923	3.83279
Gdf15	chr8:70629393-70631635	0.748633	10.6391	3.82898
Cmpk2	chr12:26469214-26479837	0.105687	1.448	3.7762
1600002D24Rik	chr16:95831122-95929077	0.115717	1.50833	3.70427
Irg1	chr14:103047011-103056573	1.29073	16.8061	3.70273
Gem	chr4:11704446-11714993	0.113238	1.46008	3.68862
Socs1	chr16:10783808-10785536	0.277162	3.56142	3.68365
Arc	chr15:74669080-74672570	0.809876	9.79276	3.59594
Ppp1r15a	chr7:45473562-45526268	3.24638	38.3302	3.56158
Ifi202b	chr1:173962568-173982844	0.24258	2.58568	3.41401
1700056E22Rik	chr1:184033031-184033998	0.143452	1.49521	3.38171
Irf7	chr7:141263182-141266424	1.17635	12.2475	3.3801
Egr4	chr6:85511121-85513542	0.1544	1.58537	3.36007
Ifi44	chr3:151730922-151749959	0.567002	5.75565	3.34355
Ifit3	chr19:34583528-34588982	0.100577	1.00814	3.32532
1700012D01Rik	chr10:127667122-127668851	0.632236	6.30749	3.31853
A430078I02Rik	chr6:86438397-86480830	0.414559	4.11804	3.31231
Spry4	chr18:38586264-38601268	0.277842	2.75179	3.30803
C230035I16Rik	chr13:23427975-23431017	0.162119	1.53881	3.24669
Scarna8	chr4:86581284-86612022	17.992	169.99	3.24002
Slc39a2	chr14:51893609-51896745	0.178493	1.66768	3.22391
Serinc4	chr2:121449197-121456764	0.163277	1.50303	3.20249
Oas2	chr5:120730332-120749848	0.740669	6.7523	3.18848
Nfkbiz	chr16:55811376-55838641	2.83807	25.3572	3.15941
Olfr102	chr17:37313410-37314437	0.200911	1.77842	3.14596
Rasip1	chr7:45627536-45639092	0.150827	1.32079	3.13044
E230013L22Rik	chr8:11453976-11480241	0.539183	4.69092	3.12102
Mmp24	chr2:155775343-155819203	0.162965	1.41491	3.11808
Mmp9	chr2:164948218-164955849	56.3951	488.874	3.11582
Gm5129	chr5:29735333-29786478	0.912345	7.79878	3.0956
Snora62	chr9:120127765-120132369	18.2948	155.398	3.08646
Arid5a	chr1:36307732-36324029	0.43073	3.65097	3.08342
Hist2h2aa2	chr3:96239778-96240374	0.311449	2.6354	3.08095
Ifit1	chr19:34640888-34650009	0.389513	3.27087	3.06993
Atf3	chr1:191170296-191183333	12.2934	102.795	3.06381
Mcemp1	chr8:3665761-3668905	0.62542	5.21745	3.06045
Tnfaip3	chr10:19000909-19015410	2.46602	20.1127	3.02785
Clec2d	chr6:129180614-129186535	1.11757	9.04552	3.01683
Kdm6b	chr11:69398517-69413675	0.753868	6.05742	3.00632
Map1b	chr13:99421463-99516602	0.197237	1.57921	3.0012
Lbx2	chr6:83086364-83088241	0.153663	1.20618	2.97261
Upp1	chr11:9118007-9136170	0.135638	1.05999	2.96622
Hbegf	chr18:36504926-36515805	0.325394	2.47881	2.92939
Saa3	chr7:46711997-46715676	61.0969	457.371	2.90419
Chac1	chr2:119351241-119354327	1.96967	14.516	2.88162
Gm10638	chr8:86723937-86747060	0.291975	2.08554	2.8365
Tnfrsf9	chr4:150920154-150946102	0.205692	1.46872	2.83601
Serpina3f,Serpina3g	chr12:104214543-104241934	0.424919	3.00419	2.82172
Ccl4	chr11:83662583-83664683	18.4889	130.232	2.81636

1700066B19Rik	chr18:35726988-35730869	0.181752	1.26958	2.80431
Hp	chr8:109575127-109579172	14.05	96.1859	2.77525
Dusp1	chr17:26505590-26508472	3.50885	23.8746	2.7664
Hist1h1t	chr13:23695810-23696545	0.257579	1.75246	2.7663
Ephb4	chr5:137350108-137374522	0.182024	1.23654	2.76411
Gm9047	chr6:29471436-29473429	0.161821	1.08974	2.75151
Hist1h2ba	chr13:23933724-23934156	0.988551	6.6384	2.74745
BC021785,G630090E	chr10:39946911-39986646	0.187246	1.25327	2.74269
Gm10584	chr7:132236254-132318291	0.152342	1.01928	2.74216
F3	chr3:121723536-121735052	0.209254	1.38533	2.7269
Rpl10	chrX:74270815-74273135	1.596	10.5237	2.72112
Cdc42ep2	chr19:5917555-5924816	1.33322	8.69821	2.7058
Egr2	chr10:67537868-67542188	1.65276	10.735	2.69937
1810011O10Rik	chr8:24437615-24438946	0.791026	5.1351	2.6986
Aqp9	chr9:71110658-71163289	0.395557	2.56248	2.69558
Klhl35	chr7:99466003-99474020	0.228772	1.47894	2.69257
Xaf1	chr11:72301628-72313733	0.324502	2.07415	2.67623
Myc	chr15:61985340-61990361	0.358124	2.26776	2.66274
Hist1h2bq	chr13:21833742-21837530	2.41313	15.2646	2.66121
Cfb	chr17:34856373-34862514	0.546769	3.45083	2.65794
Sebox	chr11:78503512-78505081	0.175475	1.10181	2.65053
Gpr25	chr1:136258913-136260873	0.223787	1.39153	2.63647
Gadd45a	chr6:67035095-67080652	0.374005	2.30163	2.62153
Tmem132a	chr19:10842543-10869779	0.476604	2.91522	2.61274
Osm	chr11:4236784-4241026	1.79101	10.9254	2.60884
Lrfn3	chr7:30355513-30362772	0.167152	1.01956	2.60871
Snord49b	chr11:62602876-62604806	1972.02	12026.9	2.60851
Ksr1	chr11:79014800-79146354	0.692563	4.22289	2.60822
Gm6225	chr18:3266353-3366863	0.415011	2.445	2.55861
Oas1b	chr5:120812634-120824160	0.530984	3.12398	2.55664
Mirc35hg	chr6:30158640-30174125	0.550676	3.17365	2.52687
Slfn5	chr11:82911252-82964850	0.27581	1.56522	2.50462
Cd40	chr2:165055635-165071654	1.54388	8.73515	2.50027
Snora16a	chr4:132308677-132311024	24.6124	138.565	2.49311
Dusp5	chr19:53529317-53541322	2.58956	14.4578	2.48107
Slpi	chr2:164354069-164356507	33.5081	187.02	2.48061
2900060B14Rik	chr1:118389057-118609462	11.2113	62.4848	2.47855
Gstt1	chr10:75783812-75798584	1.32581	7.38533	2.47779
A430093F15Rik	chr19:10740946-10786043	0.402313	2.23106	2.47134
Hist1h4m	chr13:21811745-21812150	186.733	1034.01	2.46921
B930059L03Rik	chr12:110591373-110592679	2.34975	12.852	2.45142
Gpr84	chr15:103308234-103310438	17.1484	93.6795	2.44966
5730559C18Rik	chr1:136213521-136234280	0.277167	1.51071	2.4464
Mtag2	chr7:45339125-45370564	0.200797	1.08682	2.43631
Csf1	chr3:107741047-107760469	0.483595	2.60435	2.42905
Hcar2	chr5:123863569-123865516	1.34252	7.22947	2.42895
Ier2	chr8:84661330-84662852	16.6966	89.605	2.42403
Rgs1	chr1:144244668-144249104	15.8682	84.7052	2.41631
Dlk2	chr17:46297482-46328023	0.221903	1.1845	2.41628
Il7r	chr15:9506158-9529876	0.307416	1.63491	2.41094
Mir155,Mir155hg	chr16:84713022-84715244	0.452988	2.40724	2.40984
0610009L18Rik	chr11:120348677-120351190	0.375462	1.99351	2.40857
Lrrc29	chr8:105312339-105326276	0.263776	1.39677	2.40471
C3	chr17:57203966-57228136	7.95613	41.9075	2.39707
Ifi44l	chr3:151758736-151762891	0.254067	1.33372	2.39218
H2-T24	chr17:35994503-36038174	1.63621	8.58663	2.39173
Pilrb2	chr5:137865828-137871758	1.24452	6.52044	2.38938
Pilra	chr5:137787801-137836278	4.8438	25.3094	2.38546
Abhd11os	chr5:135009151-135013157	0.223995	1.16428	2.3779
Zfp36	chr7:28376783-28379228	12.0423	62.534	2.37653
Egr3	chr14:70077444-70082613	0.270464	1.39321	2.3649
Cd244	chr1:171559192-171585316	0.5371	2.76387	2.36343
Dpy19l3	chr7:35685499-35754454	0.200782	1.02991	2.35882
Snora68	chr8:70894721-70897443	265.538	1349.7	2.34565
Jun	chr4:95049035-95052222	27.331	138.773	2.34412

Trib3	chr2:152337424-152344060	2.08736	10.5535	2.33797
Ntn1	chr11:68209363-68386826	0.261014	1.31811	2.33627
Nod1	chr6:54923941-54972612	0.374596	1.88975	2.33479
Hist1h2ad	chr13:23574380-23574915	91.5951	461.725	2.33369
Isg20	chr7:78913423-78920396	0.432151	2.14625	2.31221
Tnf	chr17:35199366-35202007	42.5207	210.708	2.30901
Rhob	chr12:8497758-8499985	15.3372	75.7108	2.30347
Asb2	chr12:103321141-103356001	0.259866	1.2779	2.29794
Klra2	chr6:131219234-131247362	0.316639	1.54776	2.28927
Ppp1r27	chr11:120549974-120551132	0.969087	4.72648	2.28607
Cdkn2b	chr4:89306288-89311032	1.34421	6.53676	2.28181
Ddit3	chr10:127290792-127311786	3.69599	17.9477	2.27976
Ptgs2	chr1:150100123-150108012	2.28432	11.0608	2.27562
Hist1h2ae	chr13:23570662-23571220	132.593	638.365	2.26737
AW011738	chr4:156203283-156206028	0.258246	1.24128	2.26501
E130218I03Rik	chr4:134243305-134245873	0.444046	2.12771	2.26052
Fjx1	chr2:102449365-102451792	0.668995	3.15256	2.23645
Hist1h4d	chr13:23581601-23581969	1796.87	8437.9	2.23139
Ier5	chr1:155096366-155099636	24.8209	116.445	2.23003
Bcl2a1a	chr9:88956919-88962416	0.827674	3.86643	2.22387
4930445N18Rik	chr18:82855896-82859162	0.285867	1.32861	2.2165
Mcmdc2	chr1:9908637-9940954	0.260388	1.2064	2.21197
Oas3	chr5:120753097-120777659	2.80022	12.9309	2.2072
Cnbd2	chr2:156311845-156375638	0.232068	1.07026	2.20534
Hist1h4b	chr13:23756936-23757386	726.746	3329.21	2.19566
Ier3	chr17:35821712-35822911	39.0756	177.788	2.18582
Gmfg	chr7:28437446-28446895	0.233483	1.05359	2.17393
3830432H09Rik	chr1:119529479-119536165	0.657597	2.9642	2.17237
Fam43a	chr16:30599722-30602797	0.468883	2.11146	2.17094
Slc15a3	chr19:10842543-10869779	6.35309	28.6007	2.17052
Gimap6	chr6:48701582-48708244	0.294112	1.32221	2.16851
BC051226	chr17:33908181-33909178	0.981453	4.40436	2.16594
Dmwd	chr7:19076199-19082775	4.3284	19.3906	2.16345
Dhx58	chr11:100694883-100704271	1.16363	5.19486	2.15845
Usp18	chr6:121245905-121270917	1.56187	6.9727	2.15844
4930592I03Rik	chr18:82910878-83005314	0.627154	2.79511	2.15601
Ehd1	chr19:6276895-6300096	36.3948	161.685	2.15138
Irgm2	chr11:58214976-58222783	0.590423	2.6202	2.14986
Pomc	chr12:3954944-3960643	1.10494	4.89644	2.14776
Spry2	chr14:105891946-105896819	1.08764	4.81104	2.14514
Mgarp	chr3:51388412-51396547	0.53163	2.3451	2.14115
Hist1h2bg	chr13:23571399-23571863	583.293	2564.25	2.13624
Slc26a1	chr5:108629809-108685446	0.906717	3.98153	2.1346
Hist1h2bh	chr13:23542922-23543444	414.917	1821.36	2.13412
Tmem140	chr6:34863145-34878065	0.437149	1.91668	2.13241
Fbxo15	chr18:84935024-84981392	0.28163	1.23376	2.13118
AA543186	chr2:25327449-25332571	0.351548	1.5337	2.12523
Abtb2	chr2:103566309-103718423	2.53005	10.9751	2.117
Vwa1	chr4:155768494-155774561	0.352243	1.52689	2.11595
1700112J16Rik	chr7:67204081-67222536	0.32083	1.37579	2.10038
Pdlim4	chr11:54054927-54069017	0.242493	1.03725	2.09674
4833419F23Rik	chr18:4353546-4368945	0.679826	2.89557	2.09061
Pilrb1	chr5:137852146-137858049	4.17999	17.6011	2.0741
Slamf8	chr1:172581376-172590568	3.67714	15.2867	2.05562
3425401B19Rik	chr14:32659118-32685272	0.325057	1.34431	2.0481
Gm19710	chr3:89998759-90068347	0.331554	1.36967	2.04651
Snora33	chr10:23785182-23787209	224.654	924.225	2.04054
Dusp2	chr2:127336158-127338377	13.5598	55.5642	2.03482
Cd38	chr5:43868826-43912374	0.734765	3.00999	2.03441
Pacsin3	chr2:91256164-91264680	0.291448	1.18793	2.02714
Aoah	chr13:20794112-21024254	4.24147	17.2415	2.02325
Hist1h4h	chr13:23531043-23531478	1470.14	5913.07	2.00795
Fosl1	chr19:5447697-5457563	1.20512	4.83534	2.00444

		20 hpi		
Gene ID	Chromosome	FPKM mock	FPKM mnv	log2(fold change)
Nudt7	chr8:114133573-114152312	4.9476	0.0629547	-6.29627
Cd24a	chr10:43579168-43584265	2.99431	0.0499371	-5.90597
Trp53i11	chr2:93187583-93201757	2.17447	0.0364642	-5.89804
H2-Q2	chr17:35342332-35345722	5.62941	0.0951228	-5.88705
Slc22a17	chr14:54906726-54913132	3.19463	0.0610247	-5.71011
Zfp229	chr17:21733723-21748969	1.62452	0.0325518	-5.64113
Ndrg2	chr14:51905270-51913488	80.0374	1.69647	-5.56007
Slc16a12	chr19:34668405-34747111	36.3251	0.834846	-5.44331
Flot2	chr11:78037940-78060432	9.65509	0.229957	-5.39185
Fads2	chr19:10064163-10101503	81.4266	1.97811	-5.3633
Car5b	chrX:163976821-164028010	8.37571	0.207121	-5.33767
Slc16a7	chr10:125227484-125328535	4.52311	0.113452	-5.31716
Panx1	chr9:15005784-15045478	9.81554	0.258703	-5.2457
Nudt12	chr17:59001400-59013322	1.46294	0.0389242	-5.23206
Fam13c	chr10:70440667-70599291	4.39255	0.124493	-5.14093
Mtus2	chr5:147957319-148316065	1.17939	0.0337534	-5.12687
Alox5	chr6:116410070-116461178	11.2103	0.326645	-5.10096
Cd27	chr6:125224211-125239934	11.3278	0.330937	-5.09717
Atp6v0e2	chr6:48537568-48541800	12.8804	0.376978	-5.09455
Tln2	chr9:67217084-67559703	2.43207	0.0722176	-5.07369
Eif2s3y	chrY:1010611-1028598	22.3326	0.69064	-5.01508
Fut10	chr8:31187330-31261924	3.7876	0.11998	-4.98042
Chst10	chr1:38863872-38898160	16.5977	0.527577	-4.97545
Lpin3	chr2:160880669-160906000	2.24199	0.0738053	-4.92491
Cntln	chr4:84884308-85131921	5.0988	0.170106	-4.90565
Kdm5d	chrY:897787-943811	4.89983	0.1701	-4.84828
Tcaf1	chr6:42672546-42693059	3.46394	0.121107	-4.83806
Pax6	chr2:105536079-105904564	4.64036	0.164414	-4.81884
Nxf7	chrX:135579786-135593855	12.493	0.448203	-4.80082
Tmlhe	chrX_GL456233_random:15964	4.17281	0.150183	-4.79623
Peg12	chr7:62461870-62464510	3.85542	0.140399	-4.77928
Them6	chr15:74721233-74724373	29.8771	1.13961	-4.71243
Dhcr24	chr4:106561037-106589113	16.8875	0.648173	-4.70344
Car2	chr3:14886425-14900770	502.674	19.2936	-4.70343
As3mt	chr19:46707442-46741095	16.4797	0.638526	-4.6898
2510009E07Rik	chr16:21649044-21694665	28.5107	1.10622	-4.68779
Dsel	chr1:111858701-111864918	2.99529	0.116655	-4.68238
Gm11127	chr17:36042960-36058645	4.99827	0.194672	-4.68231
Uty	chrY:1097143-1245738	4.63176	0.181567	-4.67299
Prss50	chr9:110857966-110864628	26.9237	1.05744	-4.67023
H2-Q1	chr17:35320557-35325099	1.11784	0.0439582	-4.66843
Odf3l1	chr9:56848658-56851963	2.11692	0.0847599	-4.64244
Clu	chr14:65968482-65981545	10.2513	0.416089	-4.62277
Cldn11	chr3:31149919-31164326	17.9223	0.738437	-4.60114
3830403N18Rik	chrX:56136571-56153496	5.1413	0.221007	-4.53997
Eno1b	chr18:47922840-48107980	94.7439	4.11176	-4.5262
Rras2	chr7:114046781-114117781	17.9493	0.78681	-4.51176
Fat1	chr8:44950207-45052257	4.74948	0.209516	-4.50264
Sema5a	chr15:32244812-32696341	6.47251	0.287029	-4.49506
Rcn1	chr2:105385947-105399319	9.92673	0.442531	-4.48747
Ndrg4	chr8:95703036-95715119	25.6425	1.16351	-4.46198
Slc43a3	chr2:84936645-84958509	11.1774	0.542174	-4.36569
Ttc12	chr9:49436960-49486225	4.79696	0.233985	-4.35763
Arhgap24	chr5:102481390-102897937	5.42646	0.266281	-4.34899
Adcy2	chr13:68620042-68999541	5.32336	0.262173	-4.34375
Erbb3	chr10:128569367-128589501	1.30684	0.0645995	-4.33841
Serpinb6b	chr13:32965512-32979037	12.1603	0.614473	-4.30668
Npl	chr1:153503015-153549714	15.4348	0.785866	-4.29576
Jrk	chr15:74702411-74710374	3.20428	0.165207	-4.27765
Plcb4	chr2:135741829-136013068	22.2428	1.16311	-4.25728
Amz1	chr5:140724126-140753312	8.06053	0.423463	-4.25057
Abcb4	chr5:8893720-8959226	16.836	0.889347	-4.24266
Mycn	chr12:12936092-12941836	197.501	10.4334	-4.24258

Xlr	chrX:53777117-53797706	11.7172	0.637207	-4.20072
Cxx1b	chrX:53669176-53670408	1.78777	0.0986635	-4.1795
Angptl2	chr2:33133418-33371494	58.2747	3.23309	-4.17188
Aqp1	chr6:55336298-55348555	4.70706	0.26427	-4.15474
Kitl	chr10:100015823-100100412	57.5157	3.23084	-4.15397
Chst3	chr10:60181527-60219260	2.93112	0.164975	-4.15114
Capn1	chr19:5988544-6018459	6.14072	0.346521	-4.14739
Zfp760	chr17:21707740-21725636	1.84899	0.104698	-4.14243
Cd109	chr9:78615545-78716260	4.89268	0.28242	-4.11471
Mgmt	chr7:136894610-137128188	5.10774	0.294874	-4.11451
Myo7b	chr18:31959233-32036931	1.04666	0.0633426	-4.04648
Chchd10	chr10:75935572-75940672	20.7492	1.2862	-4.01187
Acaa1b	chr9:119148042-119157093	4.41343	0.274968	-4.00457
Fuz	chr7:44896078-44929490	3.7093	0.233185	-3.9916
Tshz3	chr7:36698117-36773457	3.53769	0.22383	-3.98233
Cadps	chr14:12372562-12823079	2.98628	0.189499	-3.97808
H2-Q8	chr17:35424846-35428361	3.64389	0.233959	-3.96115
Hist1h2ao	chr13:21833023-21833575	23.3974	1.53242	-3.93246
Rarb	chr14:16430839-17082331	3.32886	0.221059	-3.91252
Agmo	chr12:37241638-37581932	10.2134	0.680203	-3.90835
Ddx3y	chrY:1260714-1286613	12.7649	0.851928	-3.90531
Tom1l1	chr11:90638183-90687601	12.5531	0.840016	-3.90149
Plin3	chr17:56278961-56290511	16.8439	1.1335	-3.89337
Klhl41	chr2:69670119-69684239	4.14137	0.280399	-3.88455
Unc13d	chr11:116062095-116077961	1.40789	0.0956053	-3.8803
Ankrd34a	chr3:96596635-96599778	1.98459	0.135418	-3.87335
S1pr1	chr3:115710432-115715055	11.0323	0.7605	-3.85864
Psma8	chr18:14706150-14762299	9.77988	0.675399	-3.85601
Tcp11x2	chrX:135654697-135693790	1.6803	0.117196	-3.84172
Qsox1	chr1:155778154-155812899	9.44488	0.66041	-3.8381
Zfp599	chr9:22055459-22389206	1.09997	0.0769676	-3.83707
Sgsm1	chr5:113243219-113310786	8.31493	0.593323	-3.80881
Axl	chr7:25756499-25788733	22.4424	1.61972	-3.79241
Cxcr3	chrX:101731534-101734147	9.04243	0.663146	-3.76931
Pmepa1	chr2:173224464-173276533	4.19752	0.308052	-3.76829
Msr1	chr8:39467447-40227787	74.4606	5.50086	-3.75875
Fam117a	chr11:95337017-95381872	3.50442	0.261068	-3.74668
Fxyd2	chr9:45399708-45410278	254.811	19.0507	-3.74151
Pltp	chr2:164830729-164857708	29.7427	2.23408	-3.73478
Trip6	chr5:137309898-137314241	23.1368	1.74586	-3.72818
Gpr176	chr2:118277097-118373419	4.71304	0.360051	-3.71039
Cpne8	chr15:90487480-90679388	15.7201	1.20961	-3.7
Card11	chr5:140872998-141000596	7.28739	0.578466	-3.6551
Havcr2	chr11:46193848-46589232	7.8381	0.623756	-3.65145
Lat2	chr5:134600102-134615025	72.6702	5.83664	-3.63815
A930024E05Rik	chr5:122989353-122998341	1.22327	0.0999685	-3.61313
6430531B16Rik	chr7:139972302-139978755	2.95037	0.242372	-3.6056
Tmem40	chr6:115729136-115762466	1.55366	0.127641	-3.60551
Atp6v0d2	chr4:19876837-19922566	7.76066	0.644537	-3.58984
4930455G09Rik	chr4:142017897-142028995	2.63996	0.220369	-3.58252
Plscr1	chr9:92250193-92272561	2.04258	0.172412	-3.56646
Tmem138	chr19:10570477-10590041	5.97887	0.509472	-3.5528
Slc17a9	chr2:180725338-180742278	3.13805	0.268811	-3.5452
Mical2	chr7:112225835-112355194	4.98827	0.430618	-3.53406
C1qtnf6	chr15:78523345-78529651	1.55943	0.137429	-3.50426
Kazald1	chr19:45076138-45079289	1.10654	0.0985231	-3.48945
Zfhx4	chr3:5177823-5415855	2.48026	0.220915	-3.48893
Igf2r	chr17:12682405-12868143	10.1522	0.907933	-3.48307
Calml4	chr9:62838786-62875917	2.67907	0.240115	-3.47993
Ltc4s	chr11:50236471-50238471	7.58154	0.685989	-3.46623
Emp1	chr6:135362930-135383173	52.6732	4.7914	-3.45855
Atp2b4	chr1:133702673-133753747	5.71197	0.520445	-3.45617
Olfr933	chr9:38975677-38976604	2.78754	0.257316	-3.43738
Cd63	chr10:128908918-128912818	17.8374	1.64907	-3.43518
Rasgrp3	chr17:75435904-75529053	74.9304	6.9881	-3.42258

B230312C02Rik	chr2:180370857-180401802	1.9673	0.183558	-3.42191
9330111N05Rik	chr13:80964073-81079857	1.29774	0.123148	-3.39754
Hddc3	chr7:80343136-80346097	28.0613	2.6796	-3.38849
Gpr183	chr14:121876860-122021035	48.2503	4.62111	-3.38422
Hpgds	chr6:65117292-65144730	14.8731	1.45427	-3.35434
Ank	chr15:27466676-27594907	226.284	22.4735	-3.33184
Zbtb32	chr7:30589680-30592942	29.4291	2.93246	-3.32706
Gpnmb	chr6:49036517-49058182	158.135	15.8551	-3.31813
Zfp345	chr2:150470990-150485063	1.11668	0.11225	-3.31442
Plcb2	chr2:118707516-118728438	12.2116	1.22921	-3.31244
Ppapdc3	chr2:32095650-32110820	2.23824	0.225533	-3.31095
Clstn3	chr6:124430755-124464784	2.54863	0.258341	-3.30237
Tm7sf3	chr6:146602275-146634592	14.4978	1.47175	-3.30023
Amhr2	chr15:102445366-102454639	1.62249	0.166018	-3.28879
Hsd3b7	chr7:127800608-127803802	8.70526	0.900805	-3.2726
Gm16617	chr14:51984832-52020733	1.42895	0.149571	-3.25605
Irx2	chr13:72628977-72634194	13.2122	1.38314	-3.25585
Rasal3	chr17:32390660-32404808	2.66148	0.278826	-3.25479
Tmem26	chr10:68723745-68782654	1.56942	0.166103	-3.24008
Sgsh	chr11:119314786-119355510	6.74037	0.713894	-3.23905
Iqcg	chr16:32914099-33056186	1.80242	0.195805	-3.20245
Mfsd2a	chr4:122946850-122961188	2.71882	0.298636	-3.18652
Hoxb7	chr11:96286645-96290163	8.86833	0.976154	-3.18348
Serinc2	chr4:130253496-130275586	2.13064	0.235533	-3.17729
Hk3	chr13:54949431-55021385	16.2175	1.79481	-3.17565
Hist3h2ba	chr11:58948910-58949372	19.8548	2.20557	-3.17027
Gm20554	chr13:72623465-72628564	1.73935	0.194249	-3.16257
Hmga2	chr10:120361274-120476935	12.4602	1.41683	-3.13659
Nradd	chr9:110621134-110624393	4.91188	0.559557	-3.13392
Crip1	chr12:113152011-113153879	288.484	33.0811	-3.12442
Dnajc22	chr15:99099483-99104707	2.96176	0.342041	-3.11421
Kbtbd11	chr8:15011024-15033332	35.2639	4.12008	-3.09745
Rnf183	chr4:62427541-62434726	1.70381	0.202166	-3.07515
Clec12a	chr6:129350243-129365303	34.0471	4.06341	-3.06677
Efemp2	chr19:5474689-5481854	2.92788	0.353674	-3.04936
Gm6329	chr8:45160493-45165145	6.28652	0.760602	-3.04705
Fam167b	chr4:129576814-129578580	42.2133	5.26412	-3.00343
Layn	chr9:51056779-51077094	2.82964	0.35293	-3.00316
Speg	chr1:75375296-75432306	29.3323	3.67758	-2.99566
Tmsb15b1,Tmsb15b2	chrX:136954987-136976874	1.24476	0.156128	-2.99507
Akr1b8	chr6:34354163-34368454	62.5105	7.85146	-2.99307
Ang,Rnase4	chr14:51091076-51106151	12.6633	1.61149	-2.97419
Dmrt2	chr19:25672410-25678991	2.35216	0.306828	-2.93849
Hexb	chr13:97137936-97198357	133.125	17.3963	-2.93593
Gpr179	chr11:97332108-97352073	2.08725	0.273474	-2.93213
Olfr111	chr17:37529956-37530997	3.20849	0.42285	-2.92368
Mst1	chr9:108080435-108085027	1.28241	0.169413	-2.92025
Rwdd3	chr3:121155401-121171695	1.2739	0.168456	-2.91881
AF251705	chr11:114996768-115001880	12.3051	1.6305	-2.91587
Cadm1	chr9:47530351-47853385	83.0574	11.0885	-2.90504
Ddx43	chr9:78395776-78423589	2.73469	0.365881	-2.90193
2510002D24Rik	chr16:18836579-18840113	4.88687	0.665983	-2.87535
Ceacam1	chr7:25376818-25566417	1.44072	0.197641	-2.86584
Myom1	chr17:71019556-71126856	1.47385	0.202685	-2.86227
Syngr1	chr15:80091333-80119501	47.5282	6.54141	-2.86111
Pls3	chrX:75785653-75875170	48.9877	6.82982	-2.8425
Pip5k1b	chr19:24294795-24555827	16.5832	2.31933	-2.83794
St18	chr1:6487230-6860940	3.27718	0.459486	-2.83436
Cnksr1	chr4:134228041-134238399	1.02735	0.147733	-2.79786
Ly86	chr13:37345344-37419036	91.1121	13.1023	-2.79782
Prtg	chr9:72807273-72917307	4.51026	0.652659	-2.78881
Fuom	chr7:140097814-140102441	2.31215	0.339208	-2.769
Kif17	chr4:138262250-138301973	1.04616	0.153758	-2.76637
Yipf7	chr5:69516669-69542647	2.45308	0.364598	-2.75021
Gm6307	chr2:180370857-180401802	2.45976	0.368513	-2.73873

A430033K04Rik	chr5:138622858-138648905	1.752	0.263375	-2.73381
Arhgef10	chr8:14911662-15001085	4.43358	0.675555	-2.71433
Dcstamp	chr15:39745929-39760938	24.4216	3.76089	-2.69901
Cst7	chr2:150570414-150578944	37.0491	5.72189	-2.69488
Enpp4	chr17:44096309-44105808	1.99641	0.312453	-2.6757
Myo7a	chr7:98051053-98119493	19.8223	3.11068	-2.67182
Prss46	chr9:110844505-110856522	1.41269	0.222678	-2.66541
Pianp	chr6:124996719-125003097	5.68428	0.897745	-2.6626
Clec4a2	chr6:123122689-123143999	4.01376	0.636956	-2.65569
Fblim1	chr4:141576061-141606052	6.929	1.10516	-2.64839
Car9	chr4:43507025-43513725	2.71111	0.43309	-2.64614
Rprl3	chr8:3803124-3803361	8793.34	1437.84	-2.6125
L1cam	chrX:73853779-73880834	1.66436	0.274806	-2.59848
Trpm1	chr7:64153834-64269759	1.63473	0.270444	-2.59565
Trem2	chr17:48346400-48352276	155.761	25.7715	-2.59549
Hist2h3c1	chr3:96246684-96247348	2.13215	0.355124	-2.58592
Cd72	chr4:43442276-43454626	129.263	21.7574	-2.57073
Dusp14	chr11:84048044-84068357	2.97453	0.501343	-2.56879
Tmem181c-ps	chr17:6610102-6620925	1.91644	0.32451	-2.5621
Fcna	chr2:25624666-25627974	10.3107	1.75291	-2.55631
Acp5	chr9:22055459-22389206	10.581	1.80069	-2.55486
Olfm1	chr2:28193092-28230736	119.65	20.4632	-2.54771
Kif5a	chr10:127225694-127263363	3.02171	0.517421	-2.54596
G730013B05Rik	chr16:50526244-50559459	19.5495	3.37472	-2.53429
Cyp2s1	chr7:25802475-25816530	1.17065	0.207828	-2.49385
Olfr99	chr17:37279500-37280418	2.99695	0.532566	-2.49246
Gpr68	chr12:100876681-100908198	6.05156	1.08406	-2.48086
H60b	chr10:22158608-22374139	6.10192	1.09812	-2.47423
Amica1	chr9:45079182-45135606	6.06545	1.0964	-2.46785
Cbr1	chr16:93607836-93610349	6.93819	1.26576	-2.45456
Acsbg1	chr9:54604996-54661885	4.64986	0.858452	-2.43738
Ptchd1	chrX:155569735-155623327	6.71305	1.24025	-2.43634
Anxa1	chr19:20373433-20390671	120.567	22.4327	-2.42616
Spink5	chr18:43963240-44022487	2.3698	0.441754	-2.42345
Tlr13	chrX:106143274-106160493	11.4977	2.155	-2.41559
Sash3	chrX:48146526-48161563	2.76114	0.517608	-2.41533
Twsg1	chr17:65923064-65951187	37.4699	7.05658	-2.40869
Cyb561a3	chr19:10570477-10590041	3.36487	0.633786	-2.40848
F9	chrX:59999463-60030760	1.75175	0.330379	-2.40661
Tnni2	chr7:142442467-142444405	36.8955	6.98161	-2.40181
Slc40a1	chr1:45908069-45925594	23.0045	4.39103	-2.38928
Bcar3	chr3:122419779-122530182	12.6316	2.42167	-2.38296
Arsb	chr13:93771678-93943016	28.2646	5.44444	-2.37614
Tnfrsf11a	chr1:105780722-105847981	27.51	5.30555	-2.37438
Pacrg	chr17:10403011-10840191	1.80149	0.347737	-2.37313
Pla2g2e	chr4:138877941-138882814	1.45995	0.283201	-2.36602
Apobec1	chr6:122577791-122602444	54.3264	10.6065	-2.3567
Suz12	chr11:79993105-80034123	39.2403	7.74666	-2.34069
Tgfbr2	chr9:116087694-116175363	32.4401	6.46005	-2.32816
Fam102a	chr2:32535358-32569750	13.2813	2.65664	-2.32172
Endod1	chr9:14353989-14381242	16.0237	3.2069	-2.32096
Hacd4	chr4:88412929-88438926	2.50628	0.506622	-2.30657
Hmga2-ps1	chr1:176814659-177020432	18.9688	3.84065	-2.30421
Osbpl8	chr10:111164801-111297247	77.4057	15.8124	-2.29138
Srgap3	chr6:112717971-112947266	4.94733	1.0115	-2.29016
Fnbp1l	chr3:122538718-122619714	23.2034	4.77384	-2.28111
Rgs14	chr13:55369731-55384687	8.03225	1.65999	-2.27463
Slc45a3	chr1:131962914-131982972	1.54907	0.320272	-2.27403
Gm11545	chr11:94755135-94761182	2.6831	0.555894	-2.27102
Pdgfb	chr15:79995875-80014808	17.6895	3.6652	-2.27093
Fam198b	chr3:79885929-79946278	1.97699	0.412046	-2.26243
Dlg3	chrX:100767721-100818410	1.44963	0.302462	-2.26086
Dnmt3aos	chr12:3806979-3914443	1.17271	0.245034	-2.25879
Bdh2	chr3:135281220-135304425	4.35832	0.912391	-2.25605
Snord32a	chr7:45125562-45128745	1765.19	369.986	-2.25428

3110035E14Rik	chr1:9548045-9631092	1.85226	0.389164	-2.25083
Aim1	chr10:43950306-44004846	11.8372	2.51939	-2.23218
Cacna1a	chr8:84415363-84640249	4.12103	0.877328	-2.23182
Fam217b	chr2:178414533-178422161	2.54917	0.544176	-2.22788
3300005D01Rik	chr17:5798656-5803242	11.1993	2.39179	-2.22725
Capn2	chr1:182467258-182517483	36.556	7.80725	-2.22722
Zfp563	chr17:33089366-33110704	1.07537	0.229802	-2.22637
4933404O12Rik	chr5:136919145-136937109	8.58338	1.84029	-2.22161
Mgst3	chr1:167372383-167393797	99.1752	21.3889	-2.21312
Fhit	chr14:9550093-11162035	2.94902	0.636737	-2.21147
Dner	chr1:84369838-84696221	4.41487	0.955596	-2.2079
Psd4	chr2:24385396-24408729	6.19761	1.36511	-2.1827
Itpr2	chr6:146108298-146502223	7.75668	1.72244	-2.17098
Zfp709	chr8:71882067-71892565	1.72492	0.383429	-2.1695
Hist1h2bq	chr13:21833742-21837530	2.41313	0.53777	-2.16584
Slc6a8	chrX:73673132-73682500	10.2654	2.29676	-2.16012
C130050O18Rik	chr5:139359738-139460534	18.0786	4.04748	-2.15919
2610203C22Rik	chr1:9548045-9631092	18.8984	4.24337	-2.15498
Ambp	chr4:63143278-63154142	2.59156	0.582262	-2.15408
Slc41a2	chr10:83231138-83337817	7.70015	1.73515	-2.14983
Dab2	chr15:6299788-6440709	54.0799	12.2334	-2.14427
Arhgap22	chr14:33216822-33369936	4.42108	1.00339	-2.13952
Cd180	chr13:102693557-102706631	20.2743	4.60888	-2.13716
4930487H11Rik	chr1:62703316-62818692	4.77188	1.09137	-2.12842
Abhd1	chr5:30950065-30960327	3.97817	0.91312	-2.12323
Serpinb1c	chr13:32881396-32898140	2.51716	0.58308	-2.11003
Dap	chr15:31224384-31274338	64.5712	15.007	-2.10526
Dnaaf3	chr7:4522956-4532442	2.24729	0.522662	-2.10424
D830046C22Rik	chr5:139359738-139460534	6.34097	1.47609	-2.10292
Fgf11	chr11:69796067-69801716	6.08757	1.41761	-2.10241
Mir1843a,Scarna3b	chr12:80335846-80436601	64.9243	15.2277	-2.09206
Cx3cr1	chr9:120048682-120068296	33.2154	7.79075	-2.09202
Ggact	chr14:122890859-122913165	13.5805	3.19527	-2.08752
Sh3bgrl	chrX:109095406-109162467	116.536	27.421	-2.08743
Chst14	chr2:118926496-118928583	2.46131	0.580962	-2.08291
S100a6	chr3:90612893-90614414	414.653	98.3419	-2.07603
Pdcd1	chr1:94038304-94052553	3.70581	0.879649	-2.07479
Ptpn22	chr3:103859794-103912252	27.1645	6.45067	-2.0742
Il17rc	chr6:113471454-113483163	5.97618	1.42161	-2.0717
Tmem141	chr2:25620065-25622005	22.3168	5.36166	-2.05738
Pvrl1	chr9:43744575-43807461	12.3271	2.96858	-2.05399
Atp10d	chr5:72203328-72298771	5.64003	1.36662	-2.0451
Klc4	chr17:46630630-46645144	16.789	4.07309	-2.04332
Rftn2	chr1:55170159-55226782	1.20583	0.294566	-2.03336
Mras	chr9:99385419-99436712	1.46835	0.358825	-2.03284
Nceh1	chr3:27183003-27244911	15.0031	3.67888	-2.02792
Hist3h2bb-ps	chr11:58954047-58954512	15.2635	3.75826	-2.02195
Alcam	chr16:52248995-52452997	56.9954	14.0551	-2.01975
Snord16a	chr9:64173386-64178562	2538.78	626.616	-2.01848
Rab31	chr17:65651725-65772752	142.574	35.3391	-2.01238
Bahcc1	chr11:120232946-120292297	2.09982	0.52206	-2.00798
Stk38l	chr6:146724929-146778814	7.91164	1.96822	-2.00709
Lzts2	chr19:45015175-45045772	12.8189	3.19496	-2.0044
Ids	chrX:70343069-70365085	5.23758	1.30925	-2.00016
Cxcl2	chr5:90903898-90905938	0.48828	85.7147	7.45569
Rsad2	chr12:26442742-26456452	0.0191468	2.6865	7.13248
Dusp8	chr7:142079486-142095284	0.0194681	2.42144	6.95861
Mx2	chr16:97536080-97560901	0.0280524	2.45118	6.44921
Il1a	chr2:129297369-129309972	0.0674848	5.32088	6.30096
Rpl11	chr4:136049947-136053371	0.159322	10.0695	5.9819
Il1b	chr2:129364579-129375733	1.67739	81.0145	5.59389
Lcn2	chr2:32384636-32387739	0.978368	43.8683	5.48666
Fpr2	chr17:17887823-17893952	0.0683414	2.53752	5.21452
Egr4	chr6:85511121-85513542	0.1544	5.6421	5.19149
Egr1	chr18:34861206-34864956	0.612599	21.2413	5.11578

1810011O10Rik	chr8:24437615-24438946	0.791026	25.6239	5.01762
Cxcl3	chr5:90786100-90788093	0.092175	2.85075	4.95082
Hspa1b	chr17:34956428-34959238	0.112875	3.09028	4.77494
Rtp4	chr16:23609918-23614222	0.183699	4.93027	4.74625
Gadd45a	chr6:67035095-67080652	0.374005	8.46603	4.50056
Rpl9	chr5:65388363-65391431	0.239872	5.24441	4.45044
Kctd19	chr8:105375380-105413502	0.0913889	1.97074	4.43058
Isg15	chr4:156199423-156200818	0.260008	5.09268	4.2918
Ccrl2	chr9:111054833-111057518	0.392938	7.52219	4.25878
Saa3	chr7:46711997-46715676	61.0969	1168.74	4.25772
Csf3	chr11:98701312-98703629	0.133145	2.53234	4.2494
Ppm1h	chr10:122678761-122945793	9.08497	165.546	4.1876
Lif	chr11:4257567-4272514	0.102567	1.81313	4.14383
Cxcl10	chr5:92331840-92414627	0.764427	13.0588	4.0945
Gdf15	chr8:70629393-70631635	0.748633	12.5589	4.06831
Hist1h4n	chr13:21831792-21832158	11.4457	178.617	3.96399
Phlda1	chr10:111506285-111508649	0.470448	7.29271	3.95435
Tm4sf5	chr11:70505273-70511183	0.102272	1.52888	3.902
Irg1	chr14:103047011-103056573	1.29073	18.7184	3.8582
Plk2	chr13:110395043-110400843	2.8488	41.0092	3.84752
S100a8	chr3:90669070-90670034	0.880675	12.5305	3.83069
Mcemp1	chr8:3665761-3668905	0.62542	7.87654	3.65467
Art2a-ps	chr7:101552452-101560865	0.132943	1.66885	3.64998
Nfkbiz	chr16:55811376-55838641	2.83807	33.0922	3.54351
Rpl35	chr2:38998308-39005131	1.42745	16.4862	3.52974
Maff	chr15:79346620-79359076	0.827744	9.18773	3.47245
1700019G17Rik	chr6:85899049-85904938	0.0941514	1.0441	3.47113
Gimap6	chr6:48701582-48708244	0.294112	3.20792	3.4472
Klk1b27	chr7:44052289-44056711	0.178711	1.94128	3.44131
Gm11413	chr4:83378316-83390668	0.0959885	1.03677	3.43309
Ifit1	chr19:34640888-34650009	0.389513	4.03171	3.37165
1600002D24Rik	chr16:95831122-95929077	0.115717	1.14824	3.31075
Gmfg	chr7:28437446-28446895	0.233483	2.23474	3.25872
Ppp1r15a	chr7:45473562-45526268	3.24638	30.7253	3.24253
Gem	chr4:11704446-11714993	0.113238	1.03937	3.19828
Il1f9	chr2:24186475-24193567	0.173741	1.58687	3.19117
Acyp2	chr11:30505991-30649396	0.256063	2.33194	3.18696
Hcar2	chr5:123863569-123865516	1.34252	12.072	3.16865
Arg1	chr10:24915206-24927470	0.331048	2.91311	3.13745
Asb2	chr12:103321141-103356001	0.259866	2.28514	3.13645
Mmp13	chr9:7272513-7283333	0.337066	2.94669	3.128
Csf1	chr3:107741047-107760469	0.483595	4.19691	3.11746
Mmp9	chr2:164948218-164955849	56.3951	486.347	3.10835
Socs1	chr16:10783808-10785536	0.277162	2.32969	3.07134
Hp	chr8:109575127-109579172	14.05	115.592	3.0404
Cd38	chr5:43868826-43912374	0.734765	5.7263	2.96225
Tnfaip3	chr10:19000909-19015410	2.46602	19.042	2.94893
E230013L22Rik	chr8:11453976-11480241	0.539183	4.159	2.94739
Cd40	chr2:165055635-165071654	1.54388	11.6952	2.92128
C030037D09Rik	chr11:88718642-88728572	0.199591	1.50731	2.91686
Tnfrsf9	chr4:150920154-150946102	0.205692	1.54991	2.91363
Ptgs2	chr1:150100123-150108012	2.28432	17.1584	2.90908
Serpina3f,Serpina3g	chr12:104214543-104241934	0.424919	3.16815	2.89838
Fam221a	chr6:49367738-49389904	0.191895	1.42686	2.89446
Mir155,Mir155hg	chr16:84713022-84715244	0.452988	3.35764	2.88991
Cdc42ep2	chr19:5917555-5924816	1.33322	9.88158	2.88983
Aqp9	chr9:71110658-71163289	0.395557	2.92433	2.88615
Kdm6b	chr11:69398517-69413675	0.753868	5.55636	2.88175
Slpi	chr2:164354069-164356507	33.5081	244.709	2.86848
Tnfaip6	chr2:52038112-52056681	0.167295	1.21874	2.86492
Clec4a1	chr6:122921847-122934619	0.751439	5.46188	2.86167
Hbegf	chr18:36504926-36515805	0.325394	2.36307	2.8604
Spry4	chr18:38586264-38601268	0.277842	1.98469	2.83658
Arid5a	chr1:36307732-36324029	0.43073	3.04853	2.82326
Tmem132a	chr19:10842543-10869779	0.476604	3.36775	2.82092

Ccl7	chr11:82045711-82047523	0.651802	4.5983	2.8186
Pilrb2	chr5:137865828-137871758	1.24452	8.66317	2.79931
1700012D01Rik	chr10:127667122-127668851	0.632236	4.38575	2.79429
Hist1h1t	chr13:23695810-23696545	0.257579	1.77409	2.78399
Osm	chr11:4236784-4241026	1.79101	12.3027	2.78013
Bambi-ps1	chr2:122466582-122467797	0.207525	1.42287	2.77745
B230208H11Rik	chr10:12916645-12923127	0.187952	1.2877	2.77637
Fas	chr19:34290658-34327770	5.68479	38.4731	2.75867
Hotairm1	chr6:52158523-52162020	3.63169	24.4545	2.75139
Cdkn2b	chr4:89306288-89311032	1.34421	9.02255	2.74677
Cacnb3	chr15:98632327-98644536	0.162626	1.08586	2.73922
Cfb	chr17:34856373-34862514	0.546769	3.53491	2.69267
Clec2d	chr6:129180614-129186535	1.11757	7.22065	2.69176
Gm1673	chr5:33983473-33985006	2.37855	15.3673	2.69171
Ptges	chr2:30889470-30903297	0.205796	1.32148	2.68287
Ksr1	chr11:79014800-79146354	0.692563	4.44186	2.68115
Vgf	chr5:137030294-137033351	0.188166	1.20586	2.67998
Pilra	chr5:137787801-137836278	4.8438	30.8785	2.67239
Pilrb1	chr5:137852146-137858049	4.17999	26.6146	2.67064
Lrfn3	chr7:30355513-30362772	0.167152	1.04752	2.64776
Gpr25	chr1:136258913-136260873	0.223787	1.40041	2.64565
Pomc	chr12:3954944-3960643	1.10494	6.88455	2.63939
Egr2	chr10:67537868-67542188	1.65276	10.0248	2.60063
Ppap2b	chr4:105157346-105232767	0.185447	1.12313	2.59845
F3	chr3:121723536-121735052	0.209254	1.24686	2.57497
Mtag2	chr7:45339125-45370564	0.200797	1.19258	2.57028
Ly6c2	chr15:75108160-75111949	0.992414	5.74994	2.53453
1700064M15Rik	chr12:99626052-99627974	0.192732	1.10663	2.52151
Ifi44l	chr3:151758736-151762891	0.254067	1.45787	2.52059
Pacsin3	chr2:91256164-91264680	0.291448	1.63292	2.48614
Dusp2	chr2:127336158-127338377	13.5598	75.6395	2.4798
Mgarp	chr3:51388412-51396547	0.53163	2.96215	2.47815
Thbs1	chr2:118111921-118127133	1.68726	9.36163	2.47208
Nod1	chr6:54923941-54972612	0.374596	2.07556	2.4701
Slc15a3	chr19:10842543-10869779	6.35309	35.1709	2.46885
Ifi202b	chr1:173962568-173982844	0.24258	1.33677	2.46222
Rhob	chr12:8497758-8499985	15.3372	84.2346	2.45738
Isg20	chr7:78913423-78920396	0.432151	2.35799	2.44795
Klra2	chr6:131219234-131247362	0.316639	1.70559	2.42936
Irs2	chr8:10986963-11054541	1.00649	5.37478	2.41687
Dusp1	chr17:26505590-26508472	3.50885	18.607	2.40678
Ntn1	chr11:68209363-68386826	0.261014	1.36616	2.38792
Fermt2	chr14:45458791-45530065	0.506085	2.63268	2.37908
2010005H15Rik	chr16:36221561-36257427	0.346283	1.79518	2.37411
Dlk2	chr17:46297482-46328023	0.221903	1.14407	2.36618
Tfec	chr6:16833380-16898441	0.807059	4.08575	2.33986
A430078I02Rik	chr6:86438397-86480830	0.414559	2.0831	2.32908
Rnf223	chr4:156132169-156133419	0.222819	1.11721	2.32596
Siglec1	chr2:131069219-131086765	0.211968	1.06063	2.323
Hoxc13	chr15:102921130-102928814	0.240874	1.2038	2.32124
Aoah	chr13:20794112-21024254	4.24147	21.1183	2.31586
Gcm2	chr13:41101426-41109988	0.358674	1.77578	2.30771
Gm4432	chr17:32196271-32284133	0.280095	1.38022	2.30091
Dusp5	chr19:53529317-53541322	2.58956	12.7525	2.3
Sell	chr1:164062075-164080785	0.4985	2.41265	2.27496
Camkk1	chr11:73019007-73042065	0.272337	1.30206	2.25733
Ppp1r27	chr11:120549974-120551132	0.969087	4.62878	2.25594
Jun	chr4:95049035-95052222	27.331	130.44	2.25478
Ier2	chr8:84661330-84662852	16.6966	79.172	2.24544
Lppos	chr16:24392555-24992578	0.219257	1.03114	2.23354
H1fx	chr6:87980420-87981482	0.328267	1.54001	2.23
C3	chr17:57203966-57228136	7.95613	36.9496	2.21542
Trem1	chr17:48232738-48246924	1.95128	9.05945	2.215
Bcl2l11	chr2:128126037-128162547	10.2998	47.7191	2.21195
Gm5129	chr5:29735333-29786478	0.912345	4.21863	2.20912

Cpm	chr10:117629499-117687352	0.54233	2.505	2.20757
Cnbd2	chr2:156311845-156375638	0.232068	1.0663	2.19998
E130218I03Rik	chr4:134243305-134245873	0.444046	2.0357	2.19674
Tgm2	chr2:158116404-158146392	1.41617	6.46636	2.19095
Atad3aos	chr4:155761191-155763560	0.313641	1.42909	2.18791
Gpr84	chr15:103308234-103310438	17.1484	77.9934	2.18528
Ier3	chr17:35821712-35822911	39.0756	177.139	2.18054
Lrrc16a	chr13:24012483-24280790	0.248138	1.12048	2.17491
Ass1	chr2:31470269-31520670	1.23455	5.56968	2.1736
Ehd1	chr19:6276895-6300096	36.3948	163.93	2.17128
Slc39a4	chr15:76612382-76616852	1.80856	8.14569	2.1712
Egr3	chr14:70077444-70082613	0.270464	1.19725	2.14621
C1qb	chr4:136880144-136886177	9.16846	40.3518	2.13788
Ifitm5	chr7:140948961-140950239	0.627993	2.76018	2.13594
5730559C18Rik	chr1:136213521-136234280	0.277167	1.21178	2.1283
Ptger2	chr14:44988110-45003820	0.910113	3.97433	2.12659
Rasgrp2	chr19:6400582-6415216	5.22599	22.6508	2.11578
1700026L06Rik	chr2:28692079-28699651	0.538085	2.32539	2.11157
Ldlrad4	chr18:67933256-68255549	0.254817	1.09887	2.10848
Dmwd	chr7:19076199-19082775	4.3284	18.5927	2.10283
2410006H16Rik	chr11:62602876-62604806	36.3692	156.098	2.10166
Ppbp	chr5:90768517-90770060	1.01128	4.33844	2.10099
Bcl2a1a	chr9:88956919-88962416	0.827674	3.5493	2.1004
3425401B19Rik	chr14:32659118-32685272	0.325057	1.39363	2.10008
1700102H20Rik	chr17:3557823-3559863	0.817494	3.48652	2.09251
Fam43a	chr16:30599722-30602797	0.468883	1.99627	2.09001
Gm13889	chr2:93955809-93957100	0.611941	2.58111	2.07653
Ikbke	chr1:131254601-131279563	7.90781	33.2662	2.07271
AW011738	chr4:156203283-156206028	0.258246	1.08286	2.06803
Six4	chr12:73100258-73113245	0.568296	2.37493	2.06317
Chac1	chr2:119351241-119354327	1.96967	8.16659	2.05178
Pde4b	chr4:102254741-102607262	5.53299	22.8994	2.04918
Wfdc17	chr11:83704055-83706269	7.28327	30.09	2.04663
Slamf8	chr1:172581376-172590568	3.67714	15.1631	2.04391
Zfp36	chr7:28376783-28379228	12.0423	49.6272	2.04303
Nfkbia	chr12:55280813-55492647	50.0157	205.724	2.04026
Ifitm1	chr7:140967428-140969827	50.184	205.472	2.03364
A230072C01Rik	chrX:20951664-20987349	0.589743	2.40958	2.03062
Spry1	chr3:37639946-37644599	0.868859	3.54449	2.02839
Elane	chr10:79697304-80369637	0.433926	1.76489	2.02406
Cd244	chr1:171559192-171585316	0.5371	2.18062	2.02147
Abtb2	chr2:103566309-103718423	2.53005	10.2375	2.01662
Fjx1	chr2:102449365-102451792	0.668995	2.70299	2.01449
Twist2	chr1:91801476-91848027	3.33911	13.4334	2.00829
Ddit3	chr10:127290792-127311786	3.69599	14.8472	2.00616
