# Supplementary material for: RNA Sequencing of Murine Norovirus-Infected Cells Reveals Transcriptional Alteration of Genes Important to Viral Recognition and Antigen Presentation
Source: Front Immunol. 2017 Aug 11;8:959. doi: 10.3389/fimmu.2017.00959 (PMC5554501; doi:10.3389/fimmu.2017.00959)
Supplement: Supplementary file 2 [file Table_2.PDF]

TABLE S2 Significantly expressed genes (MNV 12 hpi). Genes were ranked by fold-change. Significantly differentially expressed genes are a subset of this list with the following stringencies applied: 2-fold or more change in transcript abundance, and FPKM value >1 in at least one sample.

Gene ID	Chromosome	FPKM mock	FPKM mnv	log2(fold change)	q-value	Significant
Hist1h2br	chr13:21806411-21810199	7.34243	0.0610839	-6.90932	0.00915681	yes
H2-Q10	chr17:35470088-35474563	1.44336	0.0612835	-4.55779	0.0183466	yes
Hist1h2ad	chr13:23574380-23574915	56.5219	3.05783	-4.20823	0.00064883	yes
Dancr,Snora26	chr5:74093082-74094336	313.027	17.9763	-4.12212	0.0410745	yes
Ppia	chr11:6415869-6419810	3.38145	0.267967	-3.65752	0.00602054	yes
Rps23	chr13:90923121-90924732	11.6689	0.932911	-3.64478	0.00064883	yes
Snord89	chr1:39548746-39548840	765.326	65.6184	-3.5439	0.0130772	yes
Snord22	chr19:8723486-8726326	1628.83	144.634	-3.49336	0.0100593	yes
Hist1h3h	chr13:21717627-21718115	535.887	49.0521	-3.44954	0.00064883	yes
Cacng8	chr7:3394116-3415605	24.2231	2.22662	-3.44345	0.00064883	yes
Hist1h4n	chr13:21831792-21832158	68.7431	6.63131	-3.37385	0.00064883	yes
Snora78	chr17:24719530-24719965	181.276	19.7713	-3.19671	0.00064883	yes
Rps15a-ps4	chr4:132219892-132220589	8.10697	0.88828	-3.19008	0.00064883	yes
Rpl9	chr5:65388363-65391431	1.97909	0.224928	-3.1373	0.00701693	yes
Hist2h2aa2	chr3:96239778-96240374	9.62441	1.13065	-3.08954	0.00064883	yes
Dnaja1	chr4:40720153-40757885	62.1181	7.97757	-2.96099	0.00064883	yes
Snora74a	chr18:35553409-35558316	668.574	86.2473	-2.95454	0.00118536	yes
Mir682	chr13:75645045-75645141	1155.39	154.54	-2.90233	0.00064883	yes
Rps10	chr17:27630428-27635242	6.13967	0.846001	-2.85943	0.00064883	yes
Rps14	chr18:60774595-60778546	156.5	23.3949	-2.7419	0.00064883	yes
Oaz1	chr10:80826655-80829290	36.4049	5.47122	-2.7342	0.00064883	yes
Rpl36	chr17:56613394-56614246	21.2139	3.26059	-2.70181	0.00064883	yes
Hist1h2bp	chr13:21787487-21789213	118.522	18.576	-2.67364	0.00064883	yes
Rps28	chr17:33823036-33824498	4.51011	0.728925	-2.62932	0.0114308	yes
Tufm	chr7:126487354-126490731	4.99376	0.827788	-2.59279	0.00064883	yes
Hist1h2bg	chr13:23571399-23571863	345.964	58.1812	-2.572	0.00064883	yes
Ube2s	chr7:4808013-4812340	5.136	0.882407	-2.54113	0.00064883	yes
Atp5l	chr9:44913247-44920742	3.63409	0.632738	-2.52191	0.00568302	yes
Hist1h4k	chr13:21750144-21750553	1396.61	248.314	-2.49169	0.00064883	yes
Hist1h2bk	chr13:22035820-22036320	407.987	72.8139	-2.48624	0.00064883	yes
Hist1h4c	chr13:23698083-23698458	1321.53	238.291	-2.47141	0.00064883	yes
Rpl22l1	chr3:28805510-28807415	41.0848	7.41246	-2.47058	0.00064883	yes
Rps15a-ps6	chr11:6105691-6200451	27.0218	4.87921	-2.4694	0.0148682	yes
Rpl17	chr18:75000476-75003381	1.84055	0.343384	-2.42224	0.00064883	yes
Hist1h2ab	chr13:23751087-23751592	468.198	88.0148	-2.4113	0.00064883	yes
Rps27a	chr11:29545841-29578352	16.5582	3.17837	-2.38119	0.00064883	yes
Rpl35	chr2:38998308-39005131	10.2045	2.03873	-2.32346	0.00064883	yes
Hist1h4m	chr13:21811745-21812150	63.7915	12.9488	-2.30055	0.00064883	yes
Olfr110	chr17:37492467-37499674	0.920828	0.194642	-2.24211	0.040848	yes
Mcrs1	chr15:99224975-99262041	6.39565	1.37281	-2.21996	0.00064883	yes
Hist1h4a	chr13:23760794-23761249	682.951	146.888	-2.21706	0.00064883	yes
Terc	chr3:96414436-96414833	235.998	50.9731	-2.21097	0.00064883	yes
Rpl18	chr7:45718070-45720835	55.6517	12.1018	-2.2012	0.00064883	yes
Rpl37a	chr1:72711259-72713813	70.1645	15.2839	-2.19872	0.00064883	yes
Snrpg	chr6:86371539-86378902	60.9615	13.3443	-2.19168	0.00064883	yes
Tspo	chr15:83563572-83574203	74.3276	16.305	-2.18858	0.00064883	yes
Ppp1cc	chr5:122158278-122175269	6.83122	1.53684	-2.15218	0.00064883	yes
Uqcrq	chr11:53428947-53430831	301.048	68.2261	-2.1416	0.00064883	yes
Ndufc1	chr3:51405478-51408955	24.2118	5.52424	-2.13186	0.00064883	yes
Rpl19	chr11:98026709-98030493	42.8662	10.1798	-2.07413	0.00064883	yes
Rpl30	chr15:34440505-34443276	23.9794	5.70757	-2.07085	0.00064883	yes
1810043H04Rik	chr11:120098933-120100424	44.413	10.6121	-2.06526	0.00064883	yes
Npm3	chr19:45747733-45749563	20.8457	4.98648	-2.06365	0.00064883	yes
1810044D09Rik	chr6:91440986-91441755	1.42568	0.346666	-2.04003	0.0119604	yes
Rps2	chr17:24720062-24721927	39.426	9.63897	-2.03219	0.00064883	yes
Hist1h4i	chr13:22040959-22041362	651.414	161.829	-2.00911	0.00064883	yes
Tst	chr15:78399555-78405859	1.02332	0.255495	-2.00189	0.00531709	yes
Bcl7c	chr7:127704977-127708766	9.37253	2.3872	-1.97312	0.00064883	yes
Ppp1r14b	chr19:6975047-6977324	106.709	27.4446	-1.95909	0.00064883	yes

Rprl3	chr8:3803124-3803361	2757.14	709.348	-1.95861	0.00064883	yes
Nme2	chr11:93949813-93956256	155.693	40.0946	-1.95723	0.00064883	yes
Trappc6a	chr7:19508728-19516145	37.3672	9.69549	-1.94639	0.00064883	yes
Tmem256	chr11:69838524-69839558	39.8759	10.3589	-1.94465	0.00064883	yes
Lsm7	chr10:80852824-80855209	2.1303	0.553751	-1.94375	0.013835	yes
Gm11127	chr17:36042960-36058645	3.62547	0.961625	-1.91462	0.00064883	yes
Rpl10a	chr17:28328470-28331033	3.89337	1.0359	-1.91013	0.00064883	yes
Dynlt1b	chr17:6430111-6436295	2.01427	0.542778	-1.89182	0.00118536	yes
Gm15421	chr5:22486488-22550331	70.7636	19.1084	-1.8888	0.00419912	yes
Tmem205	chr9:21921008-21935872	32.0505	8.65613	-1.88855	0.00064883	yes
Pgk1	chrX:106187099-106203699	19.1329	5.18903	-1.88252	0.00064883	yes
Ndufs5	chr4:123712709-123718186	13.7204	3.72238	-1.88202	0.00064883	yes
Icam2	chr11:106377655-106382641	3.45941	0.968789	-1.83627	0.00064883	yes
Rnaset2b	chr17:8128590-8147832	7.03746	1.97172	-1.8356	0.00064883	yes
Spata5l1	chr2:122630624-122632704	12.291	3.47038	-1.82444	0.00064883	yes
Hist1h2bm	chr13:21722043-21722526	1095.37	311.533	-1.81397	0.00064883	yes
Klhl30	chr1:91351072-91362404	0.570845	0.16323	-1.80619	0.00118536	yes
Selenbp1	chr3:94933082-94944758	1.38137	0.401275	-1.78344	0.00064883	yes
Id3	chr4:136143821-136145392	98.7827	28.9706	-1.76967	0.00064883	yes
Hist1h2ae	chr13:23570662-23571220	69.4798	20.5405	-1.75812	0.00064883	yes
Rpl37	chr15:5116612-5119140	11.2699	3.34896	-1.75069	0.00064883	yes
Syngr1	chr15:80091333-80119501	38.2776	11.4861	-1.73661	0.00064883	yes
Rpl27	chr11:101442244-101445596	1.39626	0.420362	-1.73186	0.0116752	yes
Fam13c	chr10:70440667-70599291	6.4081	1.94078	-1.72326	0.00064883	yes
Uqcr11	chr10:80402996-80406821	90.4142	27.7173	-1.70576	0.00064883	yes
Scand1	chr2:156311845-156375638	66.0104	20.2799	-1.70265	0.00064883	yes
Ndufa11	chr17:56717761-56724248	12.773	3.9372	-1.69785	0.00064883	yes
Rps4x	chrX:102184942-102188371	333.211	103.14	-1.69183	0.00064883	yes
Crip1	chr12:113152011-113153879	365.575	113.358	-1.68929	0.00064883	yes
Tspan4	chr7:141475235-141539857	75.8451	23.5553	-1.68701	0.00118536	yes
H2-Q2	chr17:35342332-35345722	5.76971	1.79483	-1.68465	0.00064883	yes
Rps21	chr2:180257378-180258444	157.3	49.32	-1.67328	0.00064883	yes
Hist1h2ah	chr13:22035121-22035643	186.651	58.6217	-1.67083	0.00064883	yes
Tmem160	chr7:16452778-16455490	40.1123	12.6384	-1.66623	0.00064883	yes
Batf3	chr1:191098413-191108943	1.90108	0.599101	-1.66595	0.00454713	yes
Naca	chr10:128035345-128048637	9.54835	3.01973	-1.66083	0.00064883	yes
Atp5e	chr2:174461074-174464101	334.618	106.475	-1.65201	0.00064883	yes
Rpl34	chr3:130726826-130730398	106.6	34.0429	-1.64678	0.00064883	yes
Hist1h2bh	chr13:23542922-23543444	175.19	56.3377	-1.63675	0.00064883	yes
Hist1h3c	chr13:23745041-23745521	961.649	310.599	-1.63046	0.00064883	yes
Zfp771	chr7:127244525-127254801	20.8779	6.76135	-1.62659	0.00064883	yes
Fam195a	chr17:25863697-25868738	20.2424	6.61592	-1.61336	0.00064883	yes
Cbx3	chr6:51470615-51483704	4.42531	1.44745	-1.61226	0.00064883	yes
Hist1h1c	chr13:23738806-23739531	1869.37	613.444	-1.60755	0.00064883	yes
H2afx	chr9:44334714-44336073	415.951	136.978	-1.60247	0.00064883	yes
Ydjc	chr16:17139063-17148857	46.6855	15.39	-1.60098	0.00064883	yes
Get4	chr5:139252323-139270050	25.9138	8.55432	-1.599	0.00064883	yes
Atp5g1	chr11:96072792-96075694	25.8921	8.57204	-1.5948	0.00064883	yes
Rabac1	chr7:24969749-24972728	47.85	15.8857	-1.59079	0.00064883	yes
Rmrp	chr4:43492784-43493059	34513.9	11475.4	-1.58864	0.00257544	yes
Rps8	chr4:117153835-117156132	194.378	64.8204	-1.58434	0.00064883	yes
Rpl13	chr8:123102349-123105242	120.663	40.2653	-1.58338	0.00064883	yes
Rpp25l	chr4:41712032-41713517	30.5675	10.2134	-1.58153	0.00064883	yes
Hist1h2ag	chr13:22042477-22042949	266.534	89.3692	-1.57647	0.00064883	yes
Rps19	chr7:24884713-24889802	149.05	50.2932	-1.56736	0.00064883	yes
Hist1h1b	chr13:21779831-21780625	1391.11	470.478	-1.56403	0.00064883	yes
Ubald2	chr11:116434093-116439077	5.85514	1.9932	-1.55462	0.00064883	yes
Anxa11	chr14:25842154-25886804	2.91757	0.999059	-1.54612	0.00064883	yes
Cox5b	chr1:36691486-36693388	80.1816	27.6527	-1.53585	0.00064883	yes
Atp5k	chr5:108433252-108434378	233.323	81.1195	-1.52421	0.00064883	yes
Rpl12	chr2:32961711-32964045	3.12991	1.09572	-1.51425	0.00168106	yes
Rpph1	chr14:50807446-50807771	17351.1	6109.46	-1.50591	0.00064883	yes
Mrps11	chr7:78783130-78792988	21.4875	7.58542	-1.50219	0.00064883	yes
Cox7c	chr13:86044797-86046795	17.9019	6.40778	-1.48222	0.00064883	yes
Abhd17a	chr10:80583648-80590341	53.3428	19.1817	-1.47556	0.00064883	yes

Peg12	chr7:62461870-62464510	4.24789	1.53052	-1.47273	0.00064883	yes
Uqcr10	chr11:4701967-4704344	264.363	95.4757	-1.46931	0.00064883	yes
Rnaseh2c	chr19:5601872-5602959	58.1285	21.0109	-1.46811	0.00064883	yes
Cox14	chr15:99725617-99728136	47.5103	17.2141	-1.46465	0.00064883	yes
Psmg3	chr5:139823593-139826843	41.7533	15.1334	-1.46415	0.00064883	yes
Itpa	chr2:130667840-130681614	15.8961	5.76986	-1.46207	0.00064883	yes
Ntmt1	chr2:30807976-30823014	19.3429	7.05722	-1.45463	0.00064883	yes
Slc2a8	chr2:32972988-32982056	2.9055	1.06657	-1.44581	0.00064883	yes
Shb	chr4:45423275-45530828	4.24915	1.5682	-1.43807	0.00064883	yes
Pgp	chr17:24470472-24471596	59.7095	22.0401	-1.43783	0.00064883	yes
2010107E04Rik	chr12:111961375-111966977	303.561	112.268	-1.43504	0.00064883	yes
Atox1	chr11:55446642-55461138	144.781	53.5757	-1.43422	0.00064883	yes
Bloc1s1	chr10:128919913-128923524	23.9801	8.87794	-1.43354	0.00064883	yes
2410127L17Rik	chr19:18670779-18704792	1.53266	0.571346	-1.4236	0.00168106	yes
Uqcc2	chr17:27122664-27133891	138.104	51.5341	-1.42216	0.00064883	yes
Thap3	chr4:151982637-151988986	11.739	4.38679	-1.42007	0.00064883	yes
Fbxl8	chr8:105264647-105269326	2.67217	0.999597	-1.41859	0.00064883	yes
Hist1h2bj	chr13:22043229-22043658	406.411	152.525	-1.4139	0.00064883	yes
Ndufa7	chr17:33824571-33838316	133.329	50.0411	-1.4138	0.00064883	yes
Rpl11	chr4:136049947-136053371	2.96199	1.11944	-1.40379	0.00257544	yes
Bola1	chr3:96196587-96197586	37.1958	14.0773	-1.40177	0.00064883	yes
Dbndd2	chr2:164486139-164493323	3.12746	1.18948	-1.39466	0.00064883	yes
Mbd3	chr10:80392538-80399531	62.6394	23.8706	-1.39184	0.00064883	yes
1110001J03Rik	chr6:38534860-38539449	39.3887	15.0873	-1.38445	0.00064883	yes
Tmed2	chr5:124540790-124550503	20.1138	7.71719	-1.38204	0.00064883	yes
Nudt14	chr12:112934732-112942118	19.8385	7.61902	-1.38063	0.00064883	yes
Hmgcl	chr4:135946452-135962617	25.1492	9.68088	-1.3773	0.00064883	yes
Paox	chr7:140125684-140134334	0.554482	0.213736	-1.37531	0.029439	yes
Sec61b	chr4:47474660-47483233	69.8443	27.1281	-1.36435	0.00064883	yes
Ndufa2	chr18:36735069-36744587	138.374	53.76	-1.36397	0.00118536	yes
Tuba1b	chr15:98931430-98934390	152.004	59.1237	-1.3623	0.00064883	yes
Mlycd	chr8:119394891-119411088	6.04849	2.3579	-1.35907	0.00064883	yes
Shisa8	chr15:82206951-82212815	0.841432	0.328133	-1.35857	0.0191506	yes
2700094K13Rik	chr2:84669220-84670708	151.567	59.5856	-1.34692	0.00064883	yes
Psmb4	chr3:94884323-94886958	242.188	95.6356	-1.34051	0.00064883	yes
Itga7	chr10:128933812-128960988	0.895729	0.354773	-1.33617	0.00064883	yes
Fam187b	chr7:30973803-30989725	0.708107	0.281016	-1.33332	0.0401707	yes
Atp5j2	chr5:145183705-145191592	182.874	72.7152	-1.33052	0.00064883	yes
Psme2	chr14:55587439-55591101	1.16669	0.466795	-1.32156	0.00886675	yes
Ndufb8	chr19:44550253-44555415	294.493	117.952	-1.32003	0.00064883	yes
Mrps24	chr11:5703982-5707699	62.0018	24.8871	-1.31691	0.00064883	yes
Lst1	chr17:35185094-35188440	71.8131	28.8636	-1.31499	0.00064883	yes
AF251705	chr11:114996768-115001880	16.2602	6.54566	-1.31274	0.00064883	yes
Hint2	chr4:43654226-43656445	21.9188	8.87878	-1.30374	0.00064883	yes
Ndufb11	chrX:20615325-20650905	69.7732	28.2667	-1.30357	0.00886675	yes
Metrn	chr17:25794570-25797045	29.1104	11.8149	-1.30092	0.00064883	yes
Nr2f6	chr8:71374118-71381952	18.2791	7.42099	-1.30052	0.00064883	yes
Hist1h2bc	chr13:23684198-23692480	359.769	146.13	-1.29982	0.00064883	yes
Rundc3a	chr11:102393402-102402939	0.945285	0.384669	-1.29713	0.00064883	yes
Psma5	chr3:108256925-108279952	19.4542	7.92185	-1.29618	0.00064883	yes
Ptma	chr1:86526735-86530698	789.673	322.163	-1.29346	0.00064883	yes
Abhd8	chr8:71456699-71463657	9.71065	3.97925	-1.28707	0.00064883	yes
Cebpe	chr14:54710362-54712174	2.10116	0.861146	-1.28686	0.00064883	yes
Ppp2r4	chr2:30416049-30447807	73.0811	30.0358	-1.28281	0.00064883	yes
Mrps26	chr2:130563756-130565394	109.713	45.1381	-1.28131	0.00064883	yes
Fcna	chr2:25624666-25627974	9.3119	3.84359	-1.27662	0.00064883	yes
Mnd1	chr3:84087933-84155786	7.6483	3.1602	-1.27512	0.00064883	yes
Psmb5	chr14:54614119-54617995	54.2796	22.4519	-1.27357	0.00064883	yes
Coprs	chr8:13884787-13890271	11.7239	4.87736	-1.26528	0.00064883	yes
Arl10	chr13:54575012-54581128	6.12854	2.55592	-1.2617	0.00064883	yes
Pafah1b3	chr7:25295048-25297955	17.4367	7.27908	-1.2603	0.00064883	yes
Amz1	chr5:140724126-140753312	8.21821	3.43094	-1.26022	0.00064883	yes
Snta1	chr2:154376313-154408084	4.30236	1.80018	-1.25699	0.00064883	yes
Sh2d5	chr4:138250410-138260968	0.577885	0.24274	-1.25137	0.0034365	yes
Galr3,Gcat	chr15:79030873-79043558	25.6396	10.7892	-1.24878	0.00064883	yes

Llph	chr10:120227059-120232070	14.8809	6.26533	-1.248	0.00064883	yes
Znhit2	chr19:6061206-6062468	19.6912	8.29257	-1.24766	0.00064883	yes
Sac3d1	chr19:6116003-6118586	18.0821	7.62485	-1.24578	0.00064883	yes
Gm6682	chr12:4782169-4783512	4.23094	1.78485	-1.24518	0.00064883	yes
C1qc	chr4:136889801-136892914	2.07251	0.874508	-1.24484	0.00064883	yes
Pgam1	chr19:41911870-41918665	22.6613	9.56516	-1.24437	0.00064883	yes
Anapc15	chr7:101881324-101899545	13.8007	5.83623	-1.24164	0.00064883	yes
Klc4	chr17:46630630-46645144	17.8292	7.5402	-1.24156	0.00064883	yes
Nhej1	chr1:74967345-75046639	7.77145	3.28938	-1.24037	0.00064883	yes
Lrrc17	chr5:21483846-21645605	1.39714	0.593095	-1.23615	0.00064883	yes
Hist3h2ba	chr11:58948910-58949372	13.0014	5.55767	-1.22612	0.00118536	yes
Gnb2	chr5:137528128-137533229	123.527	52.8185	-1.22571	0.00064883	yes
Armc6	chr8:70220192-70263105	10.8933	4.66088	-1.22476	0.0226785	yes
Ltc4s	chr11:50236471-50238471	7.43969	3.18442	-1.22421	0.00301495	yes
Mgst3	chr1:167372383-167393797	103.145	44.3373	-1.21809	0.00064883	yes
Myadml2	chr11:120646030-120648337	2.75087	1.18314	-1.21727	0.00064883	yes
Acsf3	chr8:122775504-122817881	2.64515	1.14195	-1.21185	0.00064883	yes
2310009B15Rik	chr1:138851978-138856854	28.2469	12.1951	-1.21179	0.00064883	yes
Amhr2	chr15:102445366-102454639	0.951076	0.410978	-1.2105	0.00602054	yes
Pop7	chr5:137501438-137502429	23.7267	10.262	-1.2092	0.00064883	yes
Psmb3	chr11:97703433-97713500	32.1778	13.9211	-1.20879	0.00064883	yes
Tnfaip8l2	chr3:95134087-95142360	22.1322	9.57721	-1.20847	0.00064883	yes
Fbxw4	chr19:45560614-45660193	10.7492	4.66175	-1.20529	0.00858867	yes
Gemin7	chr7:19564948-19573343	32.1217	13.9525	-1.20302	0.00064883	yes
Rgs3	chr4:62559846-62703019	0.685927	0.298819	-1.19878	0.00064883	yes
Ndufa4	chr6:11900372-11907446	293.679	128.158	-1.19632	0.00064883	yes
Clstn3	chr6:124430755-124464784	1.35483	0.592625	-1.19292	0.00064883	yes
Phgdh	chr3:98313170-98339969	20.3078	8.88496	-1.1926	0.00064883	yes
2410015M20Rik	chr17:56607451-56609771	85.4777	37.4314	-1.1913	0.00064883	yes
Selm	chr11:3514701-3517351	12.9045	5.6542	-1.19047	0.00064883	yes
Fam173a	chr17:25786579-25792394	31.9656	14.0893	-1.18192	0.00064883	yes
Tmem115	chr9:107533944-107538656	11.1538	4.92389	-1.17966	0.00064883	yes
Ttc5	chr14:50765408-50785520	11.9754	5.29578	-1.17716	0.00064883	yes
Ech1	chr7:28825337-28832239	46.7274	20.6919	-1.1752	0.00064883	yes
Map3k10	chr7:27656374-27674581	4.36863	1.93696	-1.17338	0.00064883	yes
Mpnd	chr17:56009200-56036637	33.9186	15.0437	-1.17291	0.00064883	yes
Pdcd5	chr7:35641984-35647482	16.2969	7.24291	-1.16996	0.00064883	yes
Oxld1	chr11:120456603-120458063	9.62094	4.27819	-1.16918	0.00064883	yes
Gng5	chr3:146499835-146505543	14.9395	6.64679	-1.16841	0.00118536	yes
Coa6	chr8:126422500-126425435	12.6689	5.64043	-1.16742	0.00064883	yes
Tpgs1	chr10:79669409-79676126	21.6194	9.63401	-1.16612	0.00064883	yes
Echdc3	chr2:6188464-6321611	1.50465	0.671297	-1.1644	0.00168106	yes
Nubpl	chr12:52097745-52310959	5.1625	2.30477	-1.16345	0.00118536	yes
Gchfr	chr2:119167787-119172389	6.39777	2.85687	-1.16313	0.00168106	yes
1810011H11Rik	chr14:32785962-32817968	13.2734	5.92969	-1.16251	0.00064883	yes
Ssbp4	chr8:70597489-70608314	62.0972	27.7412	-1.1625	0.00064883	yes
Polr2f	chr15:79141366-79151767	123.576	55.3243	-1.15941	0.00064883	yes
Tbc1d16	chr11:119143042-119228499	10.5038	4.70744	-1.1579	0.00064883	yes
Slc39a3	chr10:81028539-81033912	3.40059	1.52513	-1.15685	0.00064883	yes
Ten1	chr11:116171882-116215318	30.8622	13.8447	-1.15651	0.0130772	yes
Cela1	chr15:100674421-100687920	2.14245	0.961778	-1.15549	0.00858867	yes
Ap1s1	chr5:137034993-137046060	80.6126	36.2242	-1.15405	0.00064883	yes
Pgls	chr8:71592183-71596267	121.991	54.8287	-1.15377	0.00064883	yes
1700037C18Rik	chr16:3905797-3908689	2.67345	1.20466	-1.15008	0.00064883	yes
Coro1a	chr7:126699773-126704816	262.884	118.555	-1.14887	0.00064883	yes
Gm10069	chr6:128438756-128526720	1.58602	0.715438	-1.14852	0.00064883	yes
Mrpl27	chr11:94653790-94660087	26.531	11.9856	-1.14638	0.00064883	yes
Ddx5	chr11:106780355-106788494	762.35	344.594	-1.14556	0.00064883	yes
Bckdha	chr7:25629851-25658761	14.7924	6.7035	-1.14187	0.00064883	yes
Taldo1	chr7:141392159-141402976	242.246	110.004	-1.13892	0.00064883	yes
Pddc1	chr7:141408183-141414125	12.5505	5.69948	-1.13884	0.00064883	yes
Ocel1	chr8:71371297-71373689	11.3392	5.15137	-1.13829	0.00064883	yes
Snx20	chr8:88626827-88636128	14.9295	6.78459	-1.13783	0.00064883	yes
Rpusd3	chr6:113415318-113419340	2.81116	1.27753	-1.13781	0.00064883	yes
Mrps16	chr14:20391230-20393555	58.4686	26.6091	-1.13574	0.00064883	yes

Prdx4	chrX:155323919-155338454	125.425	57.1114	-1.13498	0.00064883	yes
Alkbh7	chr17:56997338-56999336	13.0377	5.93978	-1.13421	0.00064883	yes
Hddc3	chr7:80343136-80346097	30.0548	13.6933	-1.13413	0.00064883	yes
Ap2s1	chr7:16738443-16749290	133.507	60.946	-1.13131	0.00064883	yes
Slc25a11	chr11:70644026-70647039	54.3007	24.8502	-1.12771	0.00064883	yes
Trp53i11	chr2:93187583-93201757	0.583572	0.267194	-1.12702	0.00531709	yes
Apbb1	chr7:105558464-105581653	1.28295	0.58874	-1.12376	0.00064883	yes
Capg	chr6:72544390-72562983	542.507	248.959	-1.12373	0.00064883	yes
Ogfod2	chr5:124112337-124115476	6.02394	2.76918	-1.12125	0.00064883	yes
Adprhl2	chr4:126316350-126321703	21.658	9.95974	-1.12072	0.00064883	yes
Gps1	chr11:120784271-120789102	6.10276	2.81502	-1.11632	0.00064883	yes
Psmg4	chr13:34162963-34178172	72.9559	33.6527	-1.1163	0.00064883	yes
Acaa1a	chr9:119341293-119350295	55.3133	25.5267	-1.11562	0.00064883	yes
Cyc1	chr15:76343522-76345934	123.544	57.0472	-1.1148	0.00064883	yes
Il17rc	chr6:113471454-113483163	4.26451	1.97121	-1.1133	0.00064883	yes
Hist1h4h	chr13:23531043-23531478	863.5	399.307	-1.1127	0.00064883	yes
Cds1	chr5:101765129-101823852	0.798671	0.369374	-1.11252	0.00064883	yes
Bad	chr19:6938069-6951905	20.0803	9.2897	-1.11208	0.00064883	yes
Prss50	chr9:110857966-110864628	17.138	7.93583	-1.11075	0.00064883	yes
Hist1h4j	chr13:21735095-21735407	187.036	86.6691	-1.10973	0.00064883	yes
Hist1h3i	chr13:21782914-21783397	1063.7	493.07	-1.10923	0.00064883	yes
Hspbp1	chr7:4660520-4684963	31.7852	14.7577	-1.10689	0.00064883	yes
Ppfia4	chr1:134296782-134332928	5.36048	2.49051	-1.10592	0.00064883	yes
Acbd6	chr1:155558119-155687233	46.1553	21.4518	-1.1054	0.00064883	yes
Stard3nl	chr13:19357675-19395813	31.9855	14.8826	-1.10379	0.00064883	yes
Zfand2b	chr1:75168645-75171626	15.9934	7.46161	-1.09992	0.00064883	yes
Galk1	chr11:115974724-116012719	53.1313	24.7948	-1.09953	0.00064883	yes
Mpc2	chr1:165461207-165481214	55.2456	25.7903	-1.09903	0.00064883	yes
Scrn2	chr11:97029951-97033960	3.93133	1.83668	-1.09792	0.00064883	yes
0610011F06Rik	chr17:25875499-25877163	13.1135	6.12953	-1.09721	0.00064883	yes
Nenf	chr1:191306796-191318118	30.718	14.3612	-1.09691	0.00064883	yes
Guca1a	chr17:47394557-47400584	13.2517	6.19582	-1.09682	0.00064883	yes
Snrpd2	chr7:19149837-19152726	125.197	58.5659	-1.09606	0.00064883	yes
Chmp1a	chr8:123204260-123212788	24.0789	11.2644	-1.096	0.00064883	yes
Cd300lg	chr11:102041510-102055617	0.489741	0.229668	-1.09247	0.0237709	yes
Uap1l1	chr2:25361491-25365626	38.3344	17.9921	-1.09127	0.00064883	yes
Fancd2os	chr6:113596761-113600715	1.12026	0.526522	-1.08927	0.00382257	yes
Rdm1	chr11:101627948-101636081	9.81827	4.61854	-1.08803	0.00064883	yes
Tmsb4x	chrX:167207093-167209218	1454.08	684.578	-1.08682	0.00064883	yes
Fkbp8	chr8:70527742-70535328	83.9318	39.5309	-1.08624	0.00064883	yes
Dnajc22	chr15:99099483-99104707	2.2396	1.05506	-1.08592	0.00064883	yes
Aldh16a1	chr7:45141839-45160064	29.7662	14.0289	-1.08527	0.00064883	yes
Snrnp25	chr11:32205414-32208995	18.6423	8.78882	-1.08484	0.00064883	yes
Mydgf	chr17:56176540-56183920	123.153	58.256	-1.07998	0.00064883	yes
Cox7a1	chr7:30184170-30186030	9.96699	4.71866	-1.07878	0.00257544	yes
Exoc3l4	chr12:111417429-111431680	2.15722	1.02211	-1.07762	0.00064883	yes
Dok3	chr13:55523234-55528538	21.9093	10.4014	-1.07477	0.00064883	yes
Mrpl36	chr13:73331008-73332178	64.692	30.7139	-1.0747	0.00064883	yes
Rita1	chr5:120609059-120612589	3.55632	1.68878	-1.07441	0.00064883	yes
Timm23	chr14:32180165-32201891	14.7483	7.01309	-1.07243	0.00064883	yes
Hist1h2bb	chr13:23746733-23747223	1352.41	643.661	-1.07116	0.00064883	yes
Esrra	chr19:6909697-6921808	18.6478	8.87555	-1.0711	0.00064883	yes
Tmem134	chr19:4125959-4132307	13.6748	6.51885	-1.06883	0.00064883	yes
Rgl2	chr17:33929893-33937687	18.8171	8.97534	-1.06801	0.00064883	yes
Fam188b	chr6:55203382-55320222	0.619877	0.296406	-1.0644	0.0497441	yes
Mzt2	chr16:15848440-15863322	10.4313	4.98999	-1.06381	0.00064883	yes
Snx15	chr19:6119403-6128215	5.91911	2.83411	-1.06249	0.00064883	yes
Rpl8	chr15:76904070-76906318	1503.51	720.948	-1.06036	0.00064883	yes
Pla2g16	chr19:7557458-7588545	0.732897	0.35145	-1.06029	0.00886675	yes
Commd4	chr9:57155040-57158299	43.1217	20.6823	-1.06002	0.00064883	yes
Myeov2	chr1:92637144-92641985	249.172	119.584	-1.05912	0.00064883	yes
Ccdc142,Mrpl53	chr6:83101515-83109932	52.3457	25.1263	-1.05887	0.00064883	yes
Atp13a2	chr4:140986872-141007701	34.5302	16.5994	-1.05673	0.00064883	yes
Aacs	chr5:125475872-125517403	13.1159	6.31009	-1.05558	0.00064883	yes
Sec61g	chr11:16501637-16508484	2.23886	1.07867	-1.0535	0.0228852	yes

Ndufa3	chr7:3617372-3620161	156.169	75.3462	-1.05151	0.00064883	yes
Hmg20b	chr10:81346045-81350457	16.9434	8.17719	-1.05104	0.00064883	yes
Gltscr2	chr7:15937835-15946108	49.3669	23.8475	-1.04971	0.00064883	yes
M1ap	chr6:82946921-83030309	3.23285	1.56511	-1.04654	0.00064883	yes
Gabarapl2	chr8:111940702-111952915	12.0857	5.85195	-1.04631	0.00064883	yes
Med28	chr5:45520228-45529284	13.9268	6.75719	-1.04337	0.00064883	yes
Saysd1	chr14:20075635-20083172	5.2712	2.56137	-1.04122	0.00064883	yes
Atp6v1f	chr6:29467782-29470509	89.6688	43.5863	-1.04073	0.00064883	yes
Sdhaf4	chr1:23995938-24005640	31.1446	15.1393	-1.04068	0.00064883	yes
Echs1	chr7:140105722-140116423	20.6764	10.0792	-1.0366	0.00064883	yes
Nradd	chr9:110621134-110624393	4.22874	2.06146	-1.03656	0.00168106	yes
Rhox5	chrX:37754607-37808878	4.60315	2.24413	-1.03646	0.00064883	yes
D5Ertd605e	chr5:147418619-147423044	2.30413	1.12567	-1.03344	0.00602054	yes
Cir1	chr2:73283871-73312592	0.988108	0.484438	-1.02836	0.00829754	yes
Rps17	chr7:81342732-81345234	140.914	69.0908	-1.02825	0.00064883	yes
Dtymk	chr1:93792575-93801934	267.468	131.17	-1.02792	0.00064883	yes
BC022687	chr12:112808974-112816245	1.54714	0.759212	-1.02703	0.00454713	yes
Faap100	chr11:120369561-120378746	6.89447	3.38365	-1.02686	0.00064883	yes
Mvb12a	chr8:71542929-71548026	49.9454	24.5331	-1.02562	0.00064883	yes
Snrpb	chr2:130171635-130179364	350.327	172.326	-1.02356	0.00064883	yes
Abhd15	chr11:77515116-77520628	3.20691	1.57766	-1.0234	0.00064883	yes
Sash3	chrX:48146526-48161563	1.318	0.64932	-1.02135	0.00118536	yes
Pycr2	chr1:180904273-180908088	46.5527	22.9432	-1.0208	0.00064883	yes
Tk1	chr11:117815518-117839908	42.8488	21.1321	-1.01982	0.00064883	yes
Cops7b	chr1:86587099-86606500	13.1632	6.49636	-1.01881	0.00064883	yes
Rsph3a	chr17:7945613-7979556	2.71996	1.34264	-1.01851	0.00064883	yes
Ambp	chr4:63143278-63154142	1.30318	0.64404	-1.01681	0.0116752	yes
Bst2	chr8:71534261-71537437	25.577	12.6478	-1.01596	0.00064883	yes
Zbtb8os	chr4:129336025-129347029	10.27	5.09793	-1.01045	0.00064883	yes
Tmem9	chr1:136008218-136035030	9.99171	4.96141	-1.00998	0.00064883	yes
Wbscr16	chr5:134148057-134176767	15.6376	7.76732	-1.00953	0.00064883	yes
Adck1	chr12:88360513-88461726	11.3923	5.65982	-1.00923	0.00064883	yes
Mrps18c	chr5:100798758-100804467	48.7542	24.2392	-1.00818	0.00064883	yes
Ndufs6	chr13:73319875-73328482	36.895	18.3445	-1.00808	0.00064883	yes
Dhrs7b	chr11:60830630-60858423	29.9983	14.9454	-1.00518	0.00064883	yes
2210408F21Rik	chr6:31220350-31337394	10.7434	5.35328	-1.00495	0.00064883	yes
Gm561	chr2:144594064-144595365	30.7246	15.3123	-1.00471	0.00064883	yes
Gtpbp3	chr8:71488102-71493400	10.3433	5.15762	-1.00392	0.00064883	yes
Cib1	chr7:80227155-80232805	31.052	15.4874	-1.00359	0.00064883	yes
Mief2	chr11:60728397-60732951	3.7065	1.84908	-1.00325	0.00064883	yes
Psmb9	chr17:34182098-34187330	19.2013	9.59992	-1.00011	0.00064883	yes
Rab3il1	chr19:10018227-10035586	23.6952	11.8627	-0.998163	0.00064883	yes
Tmem208	chr8:105326363-105329057	59.1667	29.6225	-0.998091	0.00064883	yes
Mfsd12	chr10:81357569-81365820	21.0022	10.5254	-0.996664	0.00064883	yes
Ndufab1	chr7:122088043-122101848	13.2534	6.64416	-0.996206	0.00118536	yes
Oscp1	chr4:126058564-126089334	0.679741	0.340794	-0.996083	0.0213708	yes
Twf2	chr9:106203107-106215387	43.6989	21.9097	-0.996025	0.00064883	yes
Wdr8	chr4:154142371-154156818	12.9723	6.51042	-0.994608	0.00064883	yes
Trappc2l	chr8:122611625-122615591	47.4572	23.8328	-0.993676	0.00064883	yes
Tmem119	chr5:113793728-113800352	2.24532	1.12765	-0.993601	0.00064883	yes
Cdpf1	chr15:85806971-85811697	11.2143	5.63476	-0.992911	0.00064883	yes
Mrpl30	chr1:37890552-37898333	105.625	53.0902	-0.992435	0.00064883	yes
Chst10	chr1:38863872-38898160	10.6119	5.33425	-0.992333	0.00064883	yes
Gm5148	chr3:37714189-37724360	162.238	81.6034	-0.991412	0.00064883	yes
Capn10	chr1:92933542-92950590	5.09796	2.56741	-0.989607	0.00064883	yes
Sigirr	chr7:141091174-141100546	3.00631	1.51445	-0.989201	0.00064883	yes
Nfe2	chr15:103248211-103258403	0.605926	0.305445	-0.988228	0.0363016	yes
Polr1c	chr17:46243919-46248045	46.0998	23.2818	-0.985559	0.00064883	yes
Aig1	chr10:13652708-13868830	19.8885	10.0499	-0.984752	0.00064883	yes
Ndufs8	chr19:3908862-3912774	171.845	86.9227	-0.983304	0.00064883	yes
Rps18	chr17:33951998-33955641	36.155	18.2975	-0.98255	0.00064883	yes
Maf1	chr15:76351293-76354378	24.4358	12.3667	-0.982532	0.00064883	yes
9930012K11Rik	chr14:70154404-70159502	2.38996	1.21171	-0.979938	0.00064883	yes
Abcg1	chr17:31057693-31117981	5.6776	2.87891	-0.979759	0.00064883	yes
Cope	chr8:70302784-70312990	71.0151	36.1166	-0.975465	0.00064883	yes

Ivd	chr2:118861999-118881357	15.8032	8.04162	-0.974657	0.00064883	yes
Use1	chr8:71366847-71369732	65.4016	33.284	-0.974498	0.00064883	yes
Spsb3	chr17:24886673-24892147	11.4287	5.81846	-0.973956	0.00064883	yes
Cyb5rl	chr4:107070167-107084805	5.1449	2.62096	-0.973049	0.00064883	yes
Brk1	chr6:113604771-113616951	95.5823	48.7335	-0.971829	0.00064883	yes
Uba52	chr8:70508265-70510367	140.528	71.658	-0.971653	0.00064883	yes
Atp5f1	chr3:105942677-105969760	135.662	69.1872	-0.971442	0.00419912	yes
Best1	chr19:9985171-10001633	1.09665	0.559671	-0.970455	0.00829754	yes
Yipf1	chr4:107314362-107359823	18.0874	9.23091	-0.970438	0.00064883	yes
Cbx8	chr11:119038435-119040913	7.58929	3.8742	-0.970065	0.00064883	yes
Psmb10	chr8:105935727-105938392	28.5043	14.5563	-0.969535	0.00064883	yes
Tmem141	chr2:25620065-25622005	27.1116	13.8495	-0.969073	0.00064883	yes
Pkig	chr2:163658385-163726158	41.5311	21.2192	-0.968825	0.00064883	yes
Ms4a6b	chr19:11518558-11530403	3.77083	1.92869	-0.967263	0.00064883	yes
Rpl28	chr7:4792964-4794547	5.74236	2.93737	-0.967119	0.017885	yes
Nme6	chr9:109832793-109842961	16.2736	8.33387	-0.965475	0.00064883	yes
Rpl39	chrX:37082519-37085184	229.121	117.697	-0.961028	0.00064883	yes
Ccdc106	chr7:5056151-5060784	9.80915	5.04668	-0.958793	0.00064883	yes
B4galt7	chr13:55600110-55609954	10.9207	5.62018	-0.958373	0.00064883	yes
Hn1	chr11:115497352-115514370	138.052	71.1388	-0.956505	0.00064883	yes
Mycn	chr12:12936092-12941836	134.182	69.1599	-0.956182	0.00064883	yes
Ebpl	chr14:61339762-61360445	9.02128	4.65013	-0.956059	0.00064883	yes
Mfap1b	chr2:121461665-121474023	0.985194	0.507838	-0.95604	0.0143874	yes
Hpdl	chr4:116819906-116821508	1.34408	0.693208	-0.955265	0.0135968	yes
Cox5a	chr9:57521231-57532426	215.206	111.047	-0.954548	0.00064883	yes
Pebp1	chr5:117282650-117287564	47.9095	24.7469	-0.953066	0.00064883	yes
Aaed1	chr13:64291835-64312710	16.7628	8.67077	-0.95103	0.00064883	yes
Lypla2	chr4:135968224-135972594	23.3083	12.0651	-0.950005	0.00064883	yes
Amdhd2	chr17:24155832-24163733	12.3665	6.4258	-0.94449	0.00064883	yes
Cbr3	chr16:93683218-93690991	5.01011	2.60483	-0.943651	0.00064883	yes
Isoc2a	chr7:4877052-4895716	12.7206	6.61725	-0.942864	0.00064883	yes
Cerk	chr15:86139100-86186141	22.957	11.9595	-0.940774	0.00064883	yes
Ndufb6	chr4:40270662-40279368	136.243	71.1065	-0.938132	0.00064883	yes
Josd2	chr7:44467979-44471658	31.282	16.3395	-0.936972	0.00064883	yes
Adap1	chr5:139271875-139325464	47.7098	24.9543	-0.934998	0.00064883	yes
Rab40c	chr17:25882113-25919714	9.25662	4.84382	-0.934341	0.00064883	yes
Hist1h2bf	chr13:23573759-23574190	94.1901	49.2919	-0.934226	0.00064883	yes
2310040G24Rik	chr6:86483375-86488227	9.01297	4.72628	-0.931297	0.00064883	yes
Smpd2	chr10:41487171-41490340	4.02408	2.11018	-0.931291	0.00064883	yes
Tmem219	chr7:126886218-126898278	23.594	12.3784	-0.930591	0.00064883	yes
Pars2	chr4:106651068-106655282	5.03695	2.64323	-0.93025	0.00064883	yes
Trappc4	chr9:44403758-44407548	34.027	17.8692	-0.929206	0.00064883	yes
1700112E06Rik	chr14:22019711-23094571	6.62244	3.47782	-0.92918	0.00168106	yes
Nudt18	chr14:70577846-70582571	1.00922	0.530448	-0.927954	0.00419912	yes
Tspan17	chr13:54789404-54796775	3.03765	1.59742	-0.927211	0.00602054	yes
Ufc1	chr1:171288563-171294982	49.9224	26.258	-0.926929	0.00064883	yes
Sumo1	chr1:59639433-59670834	47.0326	24.7536	-0.926024	0.00064883	yes
C330006A16Rik	chr2:26136806-26140506	10.9445	5.76348	-0.925194	0.00064883	yes
Akr1b3	chr6:34303929-34317489	6.81426	3.59207	-0.923742	0.00168106	yes
Ftl1	chr7:45457943-45459886	1706.49	899.87	-0.923241	0.00064883	yes
Pex6	chr17:46711462-46725541	10.6751	5.62975	-0.923101	0.00064883	yes
Asl	chr5:130011502-130024331	14.126	7.46321	-0.920483	0.00064883	yes
Dctpp1	chr7:127256958-127260667	142.507	75.3422	-0.919503	0.00064883	yes
Avpi1	chr19:42123274-42128993	34.6521	18.3373	-0.918161	0.00064883	yes
Rnf187	chr11:58932287-58938906	99.8868	52.8921	-0.917243	0.00064883	yes
Dnajc12	chr10:63382442-63408840	4.97156	2.63533	-0.915713	0.00118536	yes
Glrx5	chr12:105032688-105040910	101.344	53.7426	-0.915124	0.00064883	yes
Tgm4	chr9:123034740-123067558	2.49766	1.32479	-0.914812	0.00064883	yes
Zkscan3	chr13:21387003-21402755	10.2184	5.42096	-0.914554	0.00064883	yes
Hmx2	chr7:131554061-131556582	7.41452	3.9356	-0.913772	0.00064883	yes
Fis1	chr5:136953274-136966234	74.6669	39.6344	-0.913716	0.00064883	yes
Atp5o	chr16:91925222-91931630	132.408	70.2928	-0.913542	0.00064883	yes
Map1lc3a	chr2:155276363-155278073	2.16844	1.1529	-0.911386	0.0146074	yes
Eif4ebp1	chr8:27260326-27275656	71.3152	37.9554	-0.909906	0.00064883	yes
Susd3	chr13:49230830-49248706	46.4694	24.8016	-0.905845	0.00064883	yes

Jund	chr8:70697738-70700616	17.9681	9.59774	-0.904671	0.00064883	yes
Arhgap22	chr14:33216822-33369936	3.83267	2.04925	-0.903254	0.00064883	yes
Tfeb	chr17:47737036-47792416	10.0384	5.36778	-0.903137	0.00064883	yes
Acvr2b	chr9:119402500-119433506	1.83014	0.979235	-0.902226	0.00602054	yes
Trim47	chr11:116105749-116110235	3.69898	1.97959	-0.901928	0.00257544	yes
Zfp775	chr6:48613179-48623227	1.09928	0.588315	-0.901893	0.00419912	yes
Abcb8	chr5:24394155-24409947	16.9221	9.0629	-0.900859	0.00064883	yes
Gpaa1	chr15:76331293-76334899	23.9536	12.835	-0.900156	0.00064883	yes
Slc35b2	chr17:45564151-45567669	28.3771	15.2244	-0.898345	0.00064883	yes
Lamtor4	chr5:138255481-138259395	33.3155	17.8964	-0.896523	0.00064883	yes
Impdh2	chr9:108560500-108565566	1.55727	0.83656	-0.896483	0.0200293	yes
Lgals3	chr14:47373859-47386167	327.007	175.802	-0.89537	0.00064883	yes
Cst3	chr2:148871731-148875468	2585.26	1390.34	-0.894871	0.00064883	yes
AB124611	chr9:21526176-21545331	31.8113	17.1225	-0.893642	0.00064883	yes
Nme1	chr11:93958924-93968521	167.439	90.1308	-0.893542	0.00064883	yes
Pcyt2	chr11:120610086-120617890	22.8261	12.2905	-0.893143	0.00064883	yes
Gm6251	chr10:20148919-20281590	93.5159	50.3621	-0.892873	0.00064883	yes
Rab4b	chr7:27168432-27178883	12.7411	6.8719	-0.890713	0.00064883	yes
Exosc5	chr7:25659152-25668032	83.89	45.2676	-0.890022	0.00064883	yes
Prpsap2	chr11:61729649-61762088	9.17717	4.95345	-0.889616	0.00064883	yes
Fam64a	chr11:72042501-72047370	49.2223	26.5737	-0.88931	0.00064883	yes
Aarsd1	chr11:101406839-101417433	55.771	30.1177	-0.888906	0.00064883	yes
Hk3	chr13:54949431-55021385	10.5404	5.69759	-0.887507	0.00064883	yes
Yars2	chr16:16302964-16309640	41.6907	22.5552	-0.886264	0.00064883	yes
Cnp	chr11:100574938-100581739	6.54847	3.5457	-0.885087	0.00064883	yes
Lpxn	chr19:12798608-12833808	81.8769	44.3461	-0.884651	0.00064883	yes
Apba3	chr10:81268171-81291581	10.172	5.51202	-0.883948	0.00257544	yes
Sod1	chr16:90220741-90226324	212.551	115.264	-0.882867	0.00064883	yes
Map2k2	chr10:81105946-81124697	23.0709	12.5136	-0.882576	0.00064883	yes
Fgd2	chr17:29360913-29379535	12.2011	6.62917	-0.880107	0.00064883	yes
Macrod1	chr19:7056767-7198062	4.40528	2.39379	-0.879937	0.00382257	yes
Engase	chr11:118476959-118489198	3.02983	1.64648	-0.879853	0.00064883	yes
Wdr34	chr2:30031557-30048879	13.5961	7.39076	-0.879393	0.00064883	yes
2210013O21Rik	chrX:153723553-153741296	17.9181	9.74813	-0.878223	0.00118536	yes
Aars2	chr17:45506840-45520843	5.96978	3.2509	-0.876838	0.00064883	yes
Ing4	chr6:125009237-125049853	13.7242	7.47411	-0.876744	0.0197691	yes
Fam73b	chr2:30364232-30385519	5.78628	3.15203	-0.876356	0.00064883	yes
Mgmt	chr7:136894610-137128188	10.669	5.8179	-0.874858	0.00118536	yes
Pim3	chr15:88862193-88865726	17.772	9.69133	-0.874842	0.00064883	yes
1810043G02Rik	chr10:77978649-77985438	4.53554	2.47424	-0.874288	0.00064883	yes
Rnh1	chr7:141160325-141172851	81.2924	44.3787	-0.873255	0.00064883	yes
Kptn	chr7:16119875-16127516	7.10928	3.88417	-0.872096	0.00064883	yes
Ccdc163	chr4:116708929-116715104	3.57874	1.95572	-0.871755	0.00257544	yes
Fdxr	chr11:115268024-115276969	11.5271	6.30363	-0.870771	0.00064883	yes
Acss1	chr2:150618110-150668932	0.822959	0.4502	-0.870256	0.00915681	yes
Cisd1	chr10:71330493-71344849	65.1493	35.6534	-0.869711	0.00064883	yes
Gnb2l1	chr11:48800359-48806241	1463.62	801.823	-0.86819	0.00064883	yes
Edf1	chr2:25557899-25562082	83.4023	45.7082	-0.867634	0.00064883	yes
Exosc4	chr15:76327396-76330670	13.9838	7.66893	-0.866657	0.00064883	yes
Gemin6	chr17:80224488-80228497	30.8416	16.9261	-0.865628	0.00064883	yes
Spns1	chr7:126370059-126377934	15.5594	8.54336	-0.864909	0.00064883	yes
Fam69b	chr2:26628456-26636497	3.8676	2.12441	-0.864381	0.00531709	yes
Hcst	chr7:30417711-30419854	13.7779	7.58213	-0.861681	0.0100593	yes
Trmt112	chr19:6909697-6921808	19.4302	10.6985	-0.860897	0.0412463	yes
Rxrb	chr17:34031811-34038403	24.5043	13.495	-0.860607	0.00064883	yes
Ilvbl	chr10:78574499-78584502	6.97557	3.8424	-0.860302	0.00064883	yes
Atg9b	chr5:24364818-24392143	6.42259	3.53963	-0.859556	0.00064883	yes
Zfp524	chr7:5015507-5018488	5.87477	3.23794	-0.859457	0.00168106	yes
Msrb1	chr17:24736641-24742778	34.882	19.2461	-0.857917	0.00064883	yes
Tmem203	chr2:25255438-25256352	8.44615	4.66078	-0.857722	0.0103347	yes
S1pr1	chr3:115710432-115715055	14.3899	7.95178	-0.855708	0.00064883	yes
Snx29	chr16:11322903-11755473	5.67477	3.139	-0.854255	0.00064883	yes
Arhgef39	chr4:43496143-43499660	24.7093	13.6705	-0.853989	0.00064883	yes
Rps20	chr4:3834472-3835600	568.837	314.925	-0.853005	0.00064883	yes
Tma7	chr9:109077987-109082381	29.6047	16.3935	-0.852704	0.00064883	yes

1500011K16Rik	chr2:127791376-127792488	33.8295	18.7403	-0.852139	0.00064883	yes
Ckb	chr12:111669354-111672338	95.1142	52.7098	-0.85159	0.00064883	yes
Ccdc51	chr9:109082495-109093363	8.78055	4.86804	-0.850971	0.00064883	yes
1110012L19Rik	chrX:70385912-70389416	7.74122	4.29668	-0.849338	0.00064883	yes
Kifc3	chr8:95081200-95142540	18.2274	10.1218	-0.84864	0.00064883	yes
Anapc13	chr9:102626295-102634244	75.544	41.9558	-0.848448	0.00064883	yes
Grcc10	chr6:124739183-124741079	146.698	81.4984	-0.848008	0.00064883	yes
Acaa2	chr18:74779211-74806207	32.1394	17.8617	-0.847478	0.00064883	yes
Vmac	chr17:56713931-56717699	1.71366	0.952746	-0.846912	0.00454713	yes
Zfp213	chr17:23550798-23565294	1.99829	1.11126	-0.846568	0.00701693	yes
Cenpa	chr5:30666885-30674837	99.4774	55.3379	-0.846103	0.00064883	yes
Wfdc17	chr11:83704055-83706269	7.00498	3.90254	-0.843968	0.035173	yes
AI413582	chr17:27563768-27565727	38.6119	21.5387	-0.842117	0.00064883	yes
Fam117a	chr11:95337017-95381872	2.47936	1.38323	-0.841921	0.00217763	yes
Vaultrc5	chr18:36801762-36802107	7.40931	4.1341	-0.841764	0.0288111	yes
Rhebl1	chr15:98877759-98881414	4.72544	2.63701	-0.841547	0.00454713	yes
Rps5	chr7:12922310-12926686	1567.36	875.442	-0.840249	0.00064883	yes
Tssc4	chr7:143069367-143071087	24.1344	13.489	-0.83931	0.00064883	yes
B9d1	chr11:61505171-61512927	4.47277	2.50406	-0.836899	0.0116752	yes
Mrpl28	chr17:26123502-26126613	71.164	39.868	-0.835916	0.00064883	yes
Bdh2	chr3:135281220-135304425	7.30468	4.09322	-0.835586	0.0034365	yes
Hint3	chr10:30608206-30618366	11.8745	6.65466	-0.835433	0.0034365	yes
Aph1c	chr9:66814993-66834706	0.752592	0.421826	-0.835219	0.018127	yes
Ppapdc3	chr2:32095650-32110820	2.24374	1.25762	-0.835207	0.00634725	yes
Tcp11l2	chr10:84576946-84614355	1.48743	0.833837	-0.83498	0.0164013	yes
Mvd	chr8:122433595-122443422	17.842	10.0142	-0.833239	0.00064883	yes
Ptgr1	chr4:58965589-58987078	4.85925	2.72794	-0.83292	0.00064883	yes
Hebp1	chr6:135137518-135168215	4.78227	2.68483	-0.832867	0.00168106	yes
Hyi	chr4:118359998-118409263	17.4348	9.79938	-0.831204	0.0473024	yes
Ang,Rnase4	chr14:51091076-51106151	7.84104	4.40727	-0.831158	0.00064883	yes
Uqcrfs1	chr13:30540311-30545316	121.168	68.1159	-0.83095	0.00064883	yes
Mknk2	chr10:80665317-80676293	26.5649	14.9433	-0.830019	0.00064883	yes
Crebl2	chr6:134830198-134857883	1.56479	0.881272	-0.828312	0.0135968	yes
Gale	chr4:135965164-135968178	9.71387	5.47086	-0.82828	0.00064883	yes
Arl11	chr14:61309752-61311936	6.4131	3.61347	-0.827637	0.00064883	yes
Poll	chr19:45552275-45560543	4.73476	2.67119	-0.825809	0.00168106	yes
Eci1	chr17:24426682-24439316	33.8102	19.0775	-0.825585	0.00064883	yes
Mgst2	chr3:51661192-51682675	33.362	18.8284	-0.825296	0.00064883	yes
Atg16l2	chr7:101289615-101302088	8.44139	4.76692	-0.824422	0.00064883	yes
Itgb5	chr16:33829664-33949338	22.7457	12.8468	-0.82418	0.00064883	yes
Dscr3	chr16:94497723-94526629	21.391	12.0953	-0.822562	0.00064883	yes
Pop5	chr5:115235850-115240970	62.3093	35.2337	-0.822491	0.00064883	yes
Cox7a2	chr9:79755240-79759853	306.391	173.314	-0.821991	0.00064883	yes
Slc16a7	chr10:125227484-125328535	5.91208	3.34433	-0.821951	0.00064883	yes
B3galt4	chr17:33949911-33951488	3.38508	1.91507	-0.821792	0.00168106	yes
Nelfe	chr17:34850390-34856372	29.769	16.8418	-0.821761	0.00064883	yes
Samm50	chr15:84192232-84214303	97.2839	55.0576	-0.821261	0.00064883	yes
Snrpc	chr17:27840086-27851967	18.0772	10.2308	-0.821259	0.00064883	yes
Ccdc28b	chr4:129619273-129623908	7.29362	4.12856	-0.820996	0.00118536	yes
Sbk1	chr7:126272618-126294999	2.3479	1.32905	-0.820971	0.00118536	yes
Sdhb	chr4:140961270-140979192	177.809	100.676	-0.82061	0.00064883	yes
Slco4a1	chr2:180460977-180474853	34.7047	19.6645	-0.819541	0.00064883	yes
Bloc1s2	chr19:44139246-44146446	10.6867	6.05821	-0.818861	0.00168106	yes
Coq6	chr12:84361967-84373796	18.3532	10.4127	-0.817689	0.00064883	yes
Tomm40l	chr1:171213969-171226379	11.1605	6.33877	-0.81613	0.00064883	yes
Mxd4	chr5:34176579-34187710	4.542	2.57979	-0.816077	0.00666366	yes
Dcaf15	chr8:84097071-84104762	8.45907	4.80853	-0.814903	0.00064883	yes
Tsen54	chr11:115814738-115823102	14.0765	8.00482	-0.814342	0.00064883	yes
Loh12cr1	chr6:134639511-134711184	6.50191	3.69856	-0.8139	0.00257544	yes
Qrsl1	chr10:43874189-43901736	12.2687	6.98418	-0.812818	0.00064883	yes
Zbtb42	chr12:112678839-112682747	1.6607	0.947292	-0.809911	0.0034365	yes
Vps33b	chr7:80269654-80291579	4.86674	2.77702	-0.80942	0.00064883	yes
Smpdl3b	chr4:132732965-132757171	44.8417	25.596	-0.808921	0.00064883	yes
Hist1h4f	chr13:23551285-23551643	624.687	356.612	-0.808776	0.00064883	yes
Plekhh3	chr11:101162679-101171302	1.31157	0.749441	-0.807413	0.0125053	yes

Psma7	chr2:180036366-180042464	354.13	202.445	-0.806751	0.00064883	yes
Adssl1	chr12:112620046-112641355	33.1968	18.9793	-0.806615	0.00064883	yes
Hmces	chr6:87913975-87936613	15.737	8.99794	-0.80649	0.00064883	yes
Lime1	chr2:181381234-181383628	3.81147	2.18104	-0.805329	0.00257544	yes
Tsfm	chr10:127022331-127030814	42.2585	24.1884	-0.804928	0.00064883	yes
Rab34	chr11:78188426-78192193	14.9957	8.5908	-0.803681	0.00064883	yes
Rilpl2	chr5:124463264-124478235	16.6686	9.54995	-0.803565	0.00064883	yes
Lrrc42	chr4:107233513-107253533	13.1796	7.55267	-0.803246	0.00064883	yes
Psmb8	chr17:34198194-34201454	103.404	59.3054	-0.802053	0.00064883	yes
Ring1	chr17:34020791-34024680	8.48815	4.87037	-0.801419	0.00064883	yes
Mocs3	chr2:168230621-168232303	12.3992	7.12301	-0.799686	0.00064883	yes
Tyrobp	chr7:30413787-30417579	878.565	504.973	-0.798942	0.00064883	yes
Cyb5d1	chr11:69393611-69395346	3.57682	2.05636	-0.798584	0.0257373	yes
Myoz1	chr14:20649101-20656540	4.25664	2.44814	-0.798031	0.00382257	yes
Hmbs	chr9:44336347-44344228	28.4875	16.4004	-0.7966	0.00064883	yes
Nfatc2ip	chr7:126382853-126396737	7.47845	4.30855	-0.795536	0.00064883	yes
Lemd2	chr17:27189599-27204438	12.8598	7.42171	-0.793048	0.00064883	yes
Sssca1	chr19:5730305-5731732	56.3966	32.5603	-0.792494	0.00064883	yes
Usf1	chr1:171411312-171418759	13.1279	7.57956	-0.792447	0.00064883	yes
Mpst	chr15:78406711-78414015	13.6945	7.91984	-0.79005	0.00118536	yes
Mvk	chr5:114444268-114460590	12.7375	7.37247	-0.788862	0.00064883	yes
Plekho1	chr3:95988835-95995839	51.7905	29.9842	-0.788487	0.00064883	yes
Haus4	chr14:54541784-54554361	22.5896	13.0843	-0.787824	0.00064883	yes
Fam195b	chr11:120542887-120549727	18.228	10.5621	-0.787268	0.00118536	yes
Wdr89	chr12:75630593-75669537	808.196	468.384	-0.787013	0.0159056	yes
Tecpr1	chr5:144195346-144223578	1.44889	0.840074	-0.786361	0.00419912	yes
Trappc3	chr4:126262404-126275883	34.9745	20.279	-0.786319	0.00064883	yes
Atxn7l3	chr11:102284819-102296629	12.4541	7.2216	-0.786231	0.0135968	yes
Ube2i	chr17:25260510-25275528	12.1647	7.06016	-0.78493	0.00064883	yes
H2-T22,H2-T9	chr17:36038408-36042702	4.05211	2.3519	-0.784848	0.00257544	yes
Tmed9	chr13:55593134-55597694	72.423	42.0425	-0.7846	0.00064883	yes
Pld4	chr12:112760654-112768986	53.0033	30.783	-0.783945	0.00064883	yes
Nmnat1	chr4:149468786-149485142	4.44687	2.58329	-0.783582	0.00797055	yes
Gtpbp6	chr5:110099968-110108197	7.0089	4.07165	-0.783573	0.00977864	yes
Usp5	chr6:124815018-124829447	42.4855	24.683	-0.783452	0.00064883	yes
Kctd7	chr5:130144887-130155808	1.39862	0.812825	-0.782989	0.00382257	yes
Klf16	chr10:80567120-80577296	7.97956	4.63866	-0.7826	0.00064883	yes
Ffar4	chr19:38097078-38114263	4.51554	2.62518	-0.782481	0.00977864	yes
Atg7	chr6:114643096-114860614	9.85565	5.73353	-0.781526	0.00064883	yes
D10Jhu81e	chr10:78162066-78169768	24.9208	14.498	-0.781497	0.00064883	yes
Mfng	chr15:78755882-78773445	26.2514	15.2725	-0.781459	0.00064883	yes
Fbxo6	chr4:148145715-148152135	11.1451	6.48534	-0.781152	0.00118536	yes
Nubp1	chr16:10411937-10447350	29.4431	17.1354	-0.78095	0.00064883	yes
Unc119	chr11:78343521-78349156	26.3217	15.3309	-0.779808	0.00118536	yes
Hist1h4b	chr13:23756936-23757386	555.613	323.778	-0.779072	0.00064883	yes
Gins2	chr8:120488865-120589075	34.207	19.9354	-0.778955	0.0211532	yes
Mcat	chr15:83546796-83555711	11.5962	6.76723	-0.777016	0.00064883	yes
Pomgnt1	chr4:116150517-116159844	12.8566	7.50406	-0.776771	0.00064883	yes
Fam103a1	chr7:81762952-81769490	10.5984	6.18776	-0.776353	0.00064883	yes
Mettl7a1	chr15:100304816-100314348	4.74529	2.77098	-0.776097	0.00257544	yes
Eci2	chr13:34977747-34994144	18.781	10.9674	-0.77605	0.00064883	yes
Sf3b5	chr10:13008449-13009183	166.183	97.1255	-0.77485	0.00064883	yes
Pdia5	chr16:35397311-35490873	8.61941	5.03782	-0.774789	0.00118536	yes
Mrpl33	chr5:31613950-31622644	185.409	108.476	-0.773336	0.00064883	yes
Dedd2	chr7:25202839-25219859	5.89723	3.45201	-0.772601	0.00301495	yes
2410006H16Rik	chr11:62602876-62604806	52.683	30.8624	-0.77149	0.00168106	yes
Aldh1l1	chr6:90550847-90599171	1.14511	0.671001	-0.771101	0.0125053	yes
Ndufb2	chr6:39592582-39599471	106.514	62.4204	-0.770952	0.00064883	yes
Klf4	chr4:55527136-55532475	3.76057	2.20381	-0.770949	0.00217763	yes
Ehmt2	chr17:34898491-34914050	35.4038	20.778	-0.768848	0.00064883	yes
Lamtor1	chr7:101899807-101911903	65.8227	38.6355	-0.768658	0.00064883	yes
Sirt5	chr13:43370715-43395203	5.05593	2.96767	-0.768646	0.00666366	yes
Tmem59	chr4:107178629-107200996	98.1949	57.6601	-0.768074	0.00064883	yes
Acap3	chr4:155891874-155907251	7.04945	4.13952	-0.768049	0.00064883	yes
Pstk	chr7:131371145-131387838	11.2329	6.59709	-0.767832	0.00217763	yes

Ppp1r37	chr7:19530966-19562398	9.57301	5.62289	-0.767661	0.00064883	yes
Cuedc2	chr19:46328183-46338660	51.981	30.5425	-0.767164	0.00064883	yes
Timm9	chr12:71111427-71136675	57.8533	34.0095	-0.766458	0.00064883	yes
Narfl	chr17:25773775-25785586	13.476	7.92595	-0.765735	0.00301495	yes
Hes6	chr1:91411482-91413222	11.7525	6.92016	-0.764093	0.00118536	yes
Qdpr	chr5:45434031-45450229	56.4938	33.2666	-0.764017	0.00064883	yes
Dcxr	chr11:120725372-120727281	9.13773	5.38141	-0.763851	0.00666366	yes
Rps11	chr7:45122387-45124389	310.431	182.948	-0.76284	0.00064883	yes
Ptpn18	chr1:34459745-34473779	67.9419	40.0471	-0.762602	0.00064883	yes
Snx17	chr5:31193303-31198900	45.3625	26.7463	-0.762159	0.00064883	yes
Ndrg4	chr8:95703036-95715119	19.9457	11.7611	-0.762061	0.00064883	yes
Rps27l	chr9:66946117-66949509	253.089	149.239	-0.762019	0.00064883	yes
Mus81	chr19:5482839-5488336	8.43321	4.97405	-0.761662	0.00064883	yes
Thap4	chr1:93705390-93754838	16.8189	9.92094	-0.761539	0.00064883	yes
Rpl7	chr1:16101295-16104433	304.48	179.705	-0.760715	0.00064883	yes
9130008F23Rik	chr17:40875481-40880558	2.28072	1.34655	-0.760222	0.0119604	yes
Cox4i1	chr8:120668224-120674209	660.537	390.384	-0.758748	0.00064883	yes
Zc3hc1	chr6:30366387-30391010	18.0742	10.6826	-0.758671	0.00064883	yes
Rab29	chr1:131867276-131872889	11.0805	6.55268	-0.757865	0.00257544	yes
Ddx39	chr8:83715176-83741311	168.598	99.7655	-0.756978	0.00064883	yes
Ppp1r12c	chr7:4481520-4501680	24.9185	14.7521	-0.756297	0.00064883	yes
Supt4a	chr11:87737564-87743617	19.3907	11.4909	-0.75488	0.00168106	yes
Mad2l1bp	chr17:46147384-46153551	10.4619	6.20504	-0.753638	0.00257544	yes
Pard6a	chr8:105701147-105703494	4.29836	2.55041	-0.753056	0.0111526	yes
Cenpm	chr15:82233775-82244747	21.6858	12.8711	-0.752607	0.00064883	yes
Gpr176	chr2:118277097-118373419	2.97242	1.76519	-0.751817	0.00568302	yes
Cox6a1	chr5:115345653-115348955	266.825	158.476	-0.75163	0.00064883	yes
Phlda3	chr1:135766084-135769134	2.47981	1.47347	-0.751012	0.0237709	yes
Tmc6	chr11:117765984-117780683	4.60454	2.73639	-0.75078	0.0279983	yes
Ctnnbip1	chr4:149518240-149566437	15.2146	9.04816	-0.74976	0.00064883	yes
Eif3m	chr2:104886323-105017027	21.4923	12.7961	-0.748121	0.00168106	yes
Kctd2	chr11:115420125-115431274	6.86463	4.08783	-0.747849	0.00301495	yes
Kif22	chr7:127027730-127042420	44.484	26.4926	-0.747698	0.00064883	yes
Aamdc	chr7:97550330-97579497	3.28	1.95347	-0.747654	0.0373535	yes
Cecr5	chr6:120509493-120531299	9.34875	5.56861	-0.747455	0.00118536	yes
Ccdc23	chr4:119195309-119201298	16.9699	10.1152	-0.746448	0.00454713	yes
9930111J21Rik1,9	chr11:48945351-48979398	63.9348	38.1397	-0.745307	0.00064883	yes
Ly86	chr13:37345344-37419036	164.694	98.2715	-0.744938	0.00064883	yes
Hgh1	chr15:76368897-76371411	13.9097	8.3002	-0.744877	0.00257544	yes
Ppp1r35	chr5:137778917-137780107	27.9588	16.6838	-0.744853	0.00168106	yes
Itgb7	chr15:102215994-102231935	14.3772	8.5821	-0.744381	0.00064883	yes
Rab31	chr17:65651725-65772752	164.228	98.0939	-0.743463	0.00064883	yes
Dgcr6	chr16:18052859-18071632	14.0941	8.41967	-0.743259	0.00382257	yes
Gipc1	chr8:83652677-83664789	51.5943	30.8252	-0.743101	0.00064883	yes
Dusp9	chrX:73639440-73643514	1.09923	0.656805	-0.74296	0.0159056	yes
Slc6a12	chr6:121346696-121365773	62.3715	37.2791	-0.742521	0.00064883	yes
Pik3r2	chr8:70768180-70776712	15.162	9.06366	-0.742298	0.00064883	yes
Ppp1r16a	chr15:76671679-76694915	5.16488	3.08779	-0.742162	0.00064883	yes
Csl	chr10:99757704-99759658	13.8176	8.26115	-0.742088	0.00118536	yes
Yipf3	chr17:46248079-46252537	9.77403	5.84397	-0.742004	0.00168106	yes
Bmf	chr2:118528756-118549678	3.16469	1.89356	-0.740963	0.00064883	yes
Cars2	chr8:11514016-11550771	17.2518	10.3227	-0.740922	0.00064883	yes
Mul1	chr4:138434671-138442265	4.77179	2.85571	-0.740682	0.00168106	yes
Cd33	chr7:43527455-43533171	2.61318	1.5639	-0.74066	0.00531709	yes
Tsn	chr1:118298517-118311132	71.6378	42.9068	-0.739513	0.00064883	yes
Gde1	chr7:118688557-118705738	30.3423	18.182	-0.73882	0.00064883	yes
Rarb	chr14:16430839-17082331	2.10032	1.25916	-0.738149	0.00947319	yes
Zfp637	chr6:117841241-117845956	9.89014	5.93408	-0.736966	0.00217763	yes
Hddc2	chr10:31313404-31328086	26.5952	15.9672	-0.736057	0.00118536	yes
Etfa	chr9:55454435-55512243	30.0393	18.035	-0.73605	0.00301495	yes
Emg1	chr6:124663103-124712178	143.477	86.1453	-0.735975	0.0034365	yes
Rbck1	chr2:152316333-152332639	51.516	30.9408	-0.735508	0.00064883	yes
Galt	chr4:41755227-41759224	4.13227	2.4831	-0.734793	0.00734768	yes
Sp110	chr1:85576898-85645036	4.66043	2.8006	-0.734727	0.0481279	yes
Atp6v0e	chr17:26676395-26699646	223.021	134.188	-0.732926	0.00064883	yes

Polr2h	chr16:20717825-20722265	39.214	23.6149	-0.73167	0.00064883	yes
Sec13	chr6:113728051-113740681	110.565	66.6136	-0.731013	0.00064883	yes
Pcbp1	chr6:86524496-86526165	77.7759	46.8905	-0.730028	0.00064883	yes
Pdxp	chr15:78913918-78919517	9.10412	5.48904	-0.729967	0.00257544	yes
Hdac1	chr4:129516103-129542646	2.00888	1.21202	-0.728979	0.016917	yes
Dusp14	chr11:84048044-84068357	2.88073	1.73806	-0.728955	0.0164013	yes
St6gal1	chr16:23224739-23360350	13.1418	7.93252	-0.728314	0.00064883	yes
Ankrd54	chr15:79053093-79062859	12.636	7.62814	-0.728132	0.00257544	yes
Slc9a3r1	chr11:115163340-115181178	11.0832	6.69378	-0.727482	0.00118536	yes
Zfp688	chr7:127418965-127422034	3.67986	2.22326	-0.726975	0.0248059	yes
Frat2	chr19:41845975-41848132	2.11498	1.27802	-0.726736	0.0228852	yes
Smim7	chr8:72565197-72571048	19.5037	11.7898	-0.726215	0.00064883	yes
Tbc1d2	chr4:46604389-46650199	1.68388	1.01819	-0.725787	0.00602054	yes
Hist1h2ai	chr13:21716411-21716859	584.841	353.867	-0.724839	0.00064883	yes
Alkbh6	chr7:30308752-30314303	13.3459	8.07517	-0.724833	0.00454713	yes
2410016O06Rik	chr12:83950607-83952953	39.4831	23.8905	-0.724795	0.00064883	yes
Cox7b	chrX:106015699-106022450	132.768	80.3361	-0.724791	0.00064883	yes
Taf1c	chr8:119575234-119605240	3.26946	1.97856	-0.724597	0.0034365	yes
Tbca	chr13:94788942-94842899	71.7577	43.4512	-0.723736	0.00064883	yes
Lzts2	chr19:45015175-45045772	9.95245	6.03072	-0.722721	0.00168106	yes
Ric8	chr7:140857396-140882309	14.3785	8.71503	-0.722335	0.00064883	yes
Rsrp1	chr4:134923624-134927370	77.6118	47.0422	-0.722321	0.00168106	yes
Gstp1	chr19:4035410-4037912	4.99696	3.03035	-0.721567	0.0312197	yes
Pmm1	chr15:81951105-81960930	18.6083	11.2862	-0.721381	0.00118536	yes
Tarbp2	chr15:102518191-102523676	5.20294	3.1561	-0.721181	0.00766564	yes
Tpst1	chr5:130073325-130135733	23.6629	14.3543	-0.721148	0.00064883	yes
Ssh3	chr19:4261668-4269172	6.26203	3.79892	-0.721042	0.00217763	yes
Ctsf	chr19:4855128-4860912	17.1738	10.4316	-0.719249	0.00118536	yes
9530082P21Rik	chr17:23749235-23754065	2.72321	1.65431	-0.719081	0.00666366	yes
Mat2b	chr11:40679313-40695203	42.3293	25.7196	-0.718792	0.00168106	yes
Psrc1	chr3:108383803-108388231	17.8424	10.8436	-0.718464	0.00118536	yes
Ndufc2	chr7:97400002-97407800	174.306	105.95	-0.718248	0.00064883	yes
Cnpy3	chr17:46735710-46752214	36.1137	21.9512	-0.718245	0.00064883	yes
Cldn11	chr3:31149919-31164326	16.6264	10.1078	-0.718007	0.00118536	yes
Dtnbp1	chr13:44922079-45002096	24.2776	14.7644	-0.717502	0.00064883	yes
Gpr180	chr14:118137126-118164232	11.4502	6.96472	-0.717234	0.00168106	yes
Def8	chr8:123442955-123463899	7.42803	4.52379	-0.715445	0.00064883	yes
0610009O20Rik	chr18:38238404-38262629	13.0013	7.92097	-0.714908	0.00064883	yes
Timm8a1	chrX:134537257-134541629	7.68493	4.68376	-0.714367	0.0119604	yes
Arf5	chr6:28423639-28426499	63.5048	38.7227	-0.713687	0.00064883	yes
Plekhj1	chr10:80796098-80798626	36.5723	22.3026	-0.71354	0.00064883	yes
Frat1	chr19:41829969-41832583	2.01419	1.22837	-0.713451	0.0174052	yes
Ankrd39	chr1:36538172-36547201	4.59541	2.80321	-0.713115	0.0111526	yes
Gnb1l	chr16:18498712-18566680	3.03625	1.85214	-0.713101	0.00568302	yes
Ece2	chr16:20611600-20645915	45.2395	27.5974	-0.713051	0.00217763	yes
Gtf2ird2	chr5:134184037-134218143	4.02029	2.45352	-0.712445	0.0034365	yes
Parl	chr16:20279820-20302362	40.8683	24.9577	-0.711495	0.00064883	yes
Prkra	chr2:76629936-76647994	13.4079	8.19213	-0.710772	0.00118536	yes
Stard7	chr2:127270228-127298934	78.4471	47.9379	-0.710553	0.00168106	yes
Timm13	chr10:80879815-80900969	85.5484	52.2892	-0.710227	0.00064883	yes
Pnpo	chr11:96937815-96944019	9.44988	5.776	-0.710226	0.00301495	yes
Fzr1	chr10:81366878-81378370	30.3612	18.5633	-0.709776	0.00064883	yes
Fbxl6	chr15:76535727-76538746	14.4492	8.84335	-0.708329	0.00217763	yes
Wnt6	chr1:74771891-74785319	8.23039	5.03853	-0.707958	0.00382257	yes
Nlrx1	chr9:44252712-44268599	2.65168	1.62467	-0.706757	0.0109203	yes
Cbr1	chr16:93607836-93610349	7.85854	4.81535	-0.70662	0.00419912	yes
Socs5	chr17:87107678-87137588	11.4553	7.01966	-0.70654	0.00217763	yes
Cmc2	chr8:116888684-116921436	24.468	14.9976	-0.706168	0.00168106	yes
Timm17b	chrX:7899397-7907652	10.4527	6.40897	-0.705718	0.00382257	yes
Tbc1d22a	chr15:86214458-86498503	10.2413	6.27947	-0.705686	0.00064883	yes
Tcf19	chr17:35512734-35516824	28.7792	17.6636	-0.704249	0.00064883	yes
Sesn1	chr10:41810573-41908436	23.1664	14.2262	-0.703482	0.00064883	yes
Slc25a26	chr6:94500313-94700145	5.68579	3.49265	-0.703038	0.0300881	yes
Pygo2	chr3:89430213-89435130	14.8116	9.09974	-0.702828	0.00064883	yes
Anapc11	chr11:120598420-120608198	11.0268	6.78473	-0.700647	0.00257544	yes

Slc39a11	chr11:113244854-113565815	19.4622	11.9843	-0.699528	0.00118536	yes
Nmral1	chr16:4711317-4719356	48.6058	29.9308	-0.699497	0.00064883	yes
Hnrnpa1	chr15:103240396-103246698	44.7713	27.5719	-0.699379	0.00064883	yes
Ubac1	chr2:25996957-26021760	12.755	7.857	-0.699009	0.00064883	yes
Slc27a1	chr8:71568926-71586708	33.9688	20.9368	-0.69817	0.00118536	yes
Rplp1	chr9:61913282-61914510	1351.12	832.942	-0.697867	0.00064883	yes
Tssc1	chr12:28751827-28867491	23.4437	14.453	-0.697835	0.00064883	yes
Telo2	chr17:25099568-25115967	6.87743	4.24118	-0.697404	0.00257544	yes
Cops5	chr1:10024599-10038159	94.9271	58.5435	-0.697312	0.00064883	yes
C030006K11Rik,L	chr15:76715275-76723845	7.55229	4.66138	-0.696157	0.00766564	yes
Grhpr	chr4:44981410-44990696	9.10398	5.62698	-0.694137	0.00797055	yes
Nfs1	chr2:156123636-156145794	34.525	21.345	-0.693747	0.00301495	yes
Rpl41	chr10:128548109-128549168	1611.21	996.218	-0.693607	0.00118536	yes
Eno3	chr11:70657175-70662513	37.4124	23.1417	-0.693025	0.00064883	yes
5031439G07Rik	chr15:84945719-84987971	21.6956	13.4204	-0.692977	0.00064883	yes
Cdk2ap1	chr5:124345438-124354628	36.9214	22.8497	-0.692281	0.00118536	yes
Klhl36	chr8:119862304-119876989	3.67573	2.27539	-0.691916	0.01225	yes
Ranbp3	chr17:56673224-56711769	48.1686	29.8214	-0.691744	0.00064883	yes
Pin1	chr9:20652129-20666584	16.0653	9.94611	-0.691741	0.00064883	yes
Pcbd2	chr13:55727367-55776830	33.7355	20.8885	-0.691559	0.00454713	yes
Ndufaf3	chr9:108565864-108567342	20.3492	12.6091	-0.690511	0.00257544	yes
4930427A07Rik	chr12:113156420-113165458	5.57552	3.4572	-0.689501	0.00382257	yes
Chpf2	chr5:24586749-24592486	15.3131	9.49541	-0.689468	0.00217763	yes
Mex3d	chr10:80380354-80387651	6.63379	4.11372	-0.68939	0.00568302	yes
Mrpl4	chr9:21002736-21008837	36.7445	22.7872	-0.689305	0.00301495	yes
Ntpcr	chr8:125734202-125748235	11.4697	7.11555	-0.688779	0.00666366	yes
Arl6ip4	chr5:124116107-124118195	46.3843	28.776	-0.688768	0.00064883	yes
Polg	chr7:79392337-79466273	19.3842	12.0266	-0.688657	0.0218243	yes
Spag4	chr2:156065212-156069499	2.14681	1.33252	-0.688041	0.035782	yes
Apitd1	chr4:149128348-149137600	20.92	12.9869	-0.687834	0.00568302	yes
Wbp1	chr6:83119043-83121461	14.1344	8.77615	-0.687551	0.00419912	yes
Ankrd10	chr8:11611580-11635757	30.6359	19.0299	-0.686958	0.00064883	yes
Fbxl19	chr7:127746774-127768928	3.72873	2.3166	-0.686676	0.00382257	yes
Nat9	chr11:115182831-115187316	5.69935	3.54211	-0.686188	0.0109203	yes
Rad23a	chr8:84832281-84840665	26.2655	16.3288	-0.68575	0.0281891	yes
Hpgds	chr6:65117292-65144730	22.8007	14.1771	-0.68551	0.00064883	yes
Pycrl	chr15:75916462-75921560	34.224	21.2878	-0.684981	0.00118536	yes
Sdf2	chr11:78245745-78255496	22.7248	14.1353	-0.684962	0.00257544	yes
Arhgap15	chr2:43748823-44395953	9.57948	5.95923	-0.684822	0.00257544	yes
Lrrc25	chr8:70616843-70620850	9.50825	5.91523	-0.684744	0.00531709	yes
Zfp212	chr6:47920567-47932637	3.75179	2.33457	-0.684423	0.00915681	yes
Ngdn	chr14:55015453-55024137	46.3457	28.8463	-0.684049	0.00118536	yes
Mif	chr10:75859352-75860250	511.622	318.492	-0.683822	0.00118536	yes
N6amt2	chr14:57549597-57571569	46.5323	28.9671	-0.683816	0.00168106	yes
Commd5	chr15:76899940-76901297	21.5339	13.415	-0.682766	0.00531709	yes
Zfyve28	chr5:34194893-34288324	0.901532	0.561877	-0.682123	0.0207355	yes
Cox8a	chr19:7215157-7217616	473.795	295.717	-0.680046	0.00257544	yes
Zscan25	chr5:145283342-145291469	3.59854	2.2461	-0.679989	0.0109203	yes
Tor2a	chr2:32757025-32775633	17.4816	10.9182	-0.6791	0.00419912	yes
Slc45a3	chr1:131962914-131982972	1.48837	0.929945	-0.678512	0.0343789	yes
Pygb	chr2:150786795-150831748	18.739	11.7091	-0.678417	0.00064883	yes
Dennd3	chr15:73512559-73572242	1.87188	1.17008	-0.677875	0.00886675	yes
Kat8	chr7:127912516-127930113	7.31825	4.58308	-0.675182	0.0100593	yes
AI837181	chr19:5425143-5427316	11.4959	7.20017	-0.675015	0.00568302	yes
Nipal3	chr4:135448900-135494504	3.68185	2.30824	-0.673635	0.00419912	yes
Hps1	chr19:42755195-42779976	11.4293	7.16593	-0.673505	0.00168106	yes
Fdx1	chr9:51943024-51963602	19.5432	12.2572	-0.673034	0.00977864	yes
Commd1	chr11:22899727-22982284	105.909	66.4256	-0.673017	0.00217763	yes
Zfp444	chr7:6172512-6193104	2.8951	1.81638	-0.672551	0.00634725	yes
Ext2	chr2:93695630-93822568	16.3091	10.2323	-0.672545	0.00064883	yes
Rps9	chr7:3704040-3706897	1192.81	748.435	-0.672411	0.00301495	yes
Churc1	chr12:76765572-76783178	26.8668	16.859	-0.67231	0.00701693	yes
G630025P09Rik	chr11:69803594-69806038	2.06565	1.29645	-0.672034	0.0373535	yes
Klhl25	chr7:75848337-75874130	7.39905	4.64404	-0.671959	0.0103347	yes
Timm10b	chr7:105640539-105641845	42.6043	26.7426	-0.671859	0.00257544	yes

Hacl1	chr14:31607225-31640965	2.75378	1.72911	-0.671385	0.018127	yes
Rftn1	chr17:49993306-50190497	18.4013	11.5595	-0.67073	0.00118536	yes
Commd2	chr3:57644348-57651684	7.05169	4.43035	-0.670549	0.00419912	yes
Ttc38	chr15:85832303-85858822	1.08499	0.682031	-0.669777	0.0277512	yes
Wbscr22	chr5:135052956-135065365	36.7102	23.0801	-0.669529	0.00217763	yes
Ndufv3	chr17:31520114-31531325	130.933	82.3243	-0.669443	0.00454713	yes
Gtf2h4	chr17:35667727-35673743	18.8413	11.8602	-0.667771	0.00419912	yes
Gm11974	chr11:6525590-6528760	31.4152	19.7757	-0.667731	0.0100593	yes
Malsu1	chr6:49073794-49084717	36.2726	22.8348	-0.667646	0.00257544	yes
Gbas	chr5:129725074-129758325	30.6573	19.3154	-0.66648	0.00168106	yes
Dcaf11	chr14:55560028-55570065	11.2869	7.11142	-0.666438	0.00257544	yes
Prdx1	chr4:116684964-116700000	184.491	116.26	-0.6662	0.00301495	yes
Timm10	chr2:84827020-84830213	65.5588	41.3229	-0.665847	0.00168106	yes
Fam174a	chr1:95313627-95335284	81.817	51.6011	-0.664998	0.00118536	yes
Eif3i	chr4:129591973-129600648	206.095	130.017	-0.66461	0.00118536	yes
Eno1b	chr18:47922840-48107980	53.5194	33.7805	-0.663871	0.00118536	yes
Slc37a1	chr17:31295482-31350698	3.5328	2.23045	-0.66348	0.00858867	yes
Senp3	chr11:69673109-69682084	33.4036	21.1058	-0.662362	0.00064883	yes
Mtmr14	chr6:113237842-113281392	12.2135	7.7219	-0.661451	0.00217763	yes
Zfyve21	chr12:111814169-111828388	7.16869	4.5329	-0.661273	0.0141124	yes
Fuk	chr8:110882455-110902488	3.8018	2.40448	-0.66096	0.00797055	yes
Mob2	chr7:142008552-142061034	18.1482	11.4826	-0.660372	0.00168106	yes
Hyal2	chr9:107569162-107572778	10.4463	6.61473	-0.659235	0.00454713	yes
Mrm1	chr11:84813060-84819515	6.68335	4.23295	-0.658908	0.0111526	yes
Lrrc20	chr10:61475832-61582228	7.0181	4.44524	-0.658819	0.00602054	yes
Agk	chr6:40325477-40396762	3.12997	1.98284	-0.658585	0.0205128	yes
H2afj	chr6:136808247-136810074	41.9294	26.5677	-0.658286	0.00301495	yes
Hsd3b7	chr7:127800608-127803802	8.15123	5.16753	-0.657544	0.010626	yes
Med22	chr2:26905266-26910642	22.2776	14.1244	-0.657405	0.0125053	yes
Suv420h2	chr7:4740126-4747514	7.45162	4.72467	-0.657339	0.00766564	yes
Enkd1	chr8:105703651-105708168	9.85035	6.24781	-0.656824	0.00886675	yes
Cdkn2c	chr4:109660875-109666756	37.278	23.6537	-0.656259	0.00257544	yes
Mrpl40	chr16:18872017-18876637	54.1907	34.3932	-0.655923	0.00168106	yes
Fam134c	chr11:101096321-101119843	14.2777	9.06216	-0.655834	0.00257544	yes
Nr0b2	chr4:133553389-133556536	7.26643	4.6121	-0.655824	0.0224583	yes
Smco4	chr9:15505494-15545259	15.7175	9.97655	-0.655754	0.0135968	yes
Slc26a6	chr9:108854042-108862143	2.80293	1.77957	-0.655407	0.029439	yes
Cyba	chr8:122424770-122432940	243.093	154.431	-0.654547	0.00301495	yes
Smad6	chr9:63953075-64022059	7.17806	4.56647	-0.652515	0.00766564	yes
Cuta	chr17:26937971-26939538	71.4407	45.4651	-0.651987	0.00382257	yes
Pts	chr9:50521616-50528641	44.7477	28.4891	-0.651403	0.00217763	yes
4930461G14Rik	chr9:58455172-58469623	4.22691	2.69112	-0.651395	0.0275447	yes
Tmem60	chr5:20882452-20886870	22.0616	14.0485	-0.651122	0.0125053	yes
Akr1a1	chr4:116636509-116651674	401.176	255.511	-0.65085	0.00454713	yes
Acaa1b	chr9:119148042-119157093	4.44669	2.83219	-0.650814	0.024169	yes
Lztr1	chr16:17508970-17526330	19.767	12.5937	-0.650391	0.00257544	yes
Prpf19	chr19:10895230-10909559	35.9239	22.8983	-0.649702	0.00118536	yes
Mrps7	chr11:115604150-115607624	51.8895	33.0831	-0.649348	0.00118536	yes
Bet1l	chr7:140853383-140856383	8.09831	5.16332	-0.649324	0.0135968	yes
Ifitm6	chr7:141015811-141016892	21.2415	13.5482	-0.648783	0.0188838	yes
Ddt	chr10:75771232-75773374	22.4013	14.288	-0.648773	0.0174052	yes
Klhl24	chr16:20097553-20127744	5.65039	3.60493	-0.648379	0.0034365	yes
S100a6	chr3:90612893-90614414	585.961	373.864	-0.648289	0.00118536	yes
Hist2h2ac	chr3:96220412-96220880	1745.79	1114.69	-0.647236	0.00257544	yes
Vat1	chr11:101458747-101466199	22.0721	14.096	-0.646941	0.00382257	yes
Snx8	chr5:140340302-140389247	23.4328	14.9742	-0.646047	0.00454713	yes
Aimp1	chr3:132660497-132683879	85.4806	54.6792	-0.644605	0.00257544	yes
Zkscan14	chr5:145194945-145201882	4.90526	3.13811	-0.644433	0.00734768	yes
Lrrc14	chr15:76710739-76715091	12.0397	7.70234	-0.644429	0.00666366	yes
Ttc32	chr12:9029996-9036394	39.9592	25.5929	-0.642785	0.00382257	yes
Tmem147	chr7:30727700-30729534	91.8701	58.8924	-0.641514	0.0034365	yes
Mthfr	chr4:148039076-148059562	2.77458	1.77888	-0.641299	0.00766564	yes
Ptp4a3	chr15:73723144-73758766	21.1263	13.5461	-0.641163	0.00118536	yes
Fuom	chr7:140097814-140102441	3.21187	2.06076	-0.640239	0.0166553	yes
Ube2e3	chr2:78869046-78920583	38.4297	24.6789	-0.638946	0.00257544	yes

Acox3	chr5:35581226-35613801	3.40208	2.18495	-0.638818	0.00915681	yes
Naa10	chrX:73916869-73921944	68.5231	44.0287	-0.638147	0.00118536	yes
Map3k11	chr19:5689130-5702864	12.3824	7.95654	-0.638078	0.00257544	yes
Ccdc32	chr2:119017778-119029393	10.6208	6.8282	-0.637321	0.00858867	yes
Lat2	chr5:134600102-134615025	79.0868	50.8526	-0.637115	0.00118536	yes
Rpusd4	chr9:35267880-35275957	29.9966	19.3009	-0.636127	0.00217763	yes
Coro1b	chr19:4148662-4154035	59.5869	38.3442	-0.635986	0.00064883	yes
Slc40a1	chr1:45908069-45925594	26.8378	17.2744	-0.63563	0.0049451	yes
Trem2	chr17:48346400-48352276	200.034	128.794	-0.635175	0.00217763	yes
Abhd11	chr5:135009151-135013157	32.0374	20.6345	-0.634699	0.00701693	yes
Hist1h1d	chr13:23555031-23555807	764.265	492.269	-0.634625	0.00419912	yes
Tprgl	chr4:154157484-154160684	39.8486	25.6938	-0.633109	0.0034365	yes
1110008P14Rik	chr2:32379100-32381915	21.0583	13.5861	-0.632259	0.0161452	yes
Emp3	chr7:45918022-45921426	300.773	194.085	-0.63199	0.00257544	yes
Nop9	chr14:55745692-55758424	21.0223	13.5665	-0.631876	0.0049451	yes
Card9	chr2:26352311-26359547	28.5783	18.4441	-0.631761	0.00301495	yes
Hps6	chr19:46003477-46006173	5.86203	3.78341	-0.631711	0.0100593	yes
Slc27a4	chr2:29802679-29817522	4.03534	2.60609	-0.630801	0.0100593	yes
Trpv2	chr11:62574485-62600305	30.9538	19.9911	-0.630757	0.00301495	yes
Tyw1	chr5:130255618-130341567	13.249	8.55737	-0.630645	0.00419912	yes
Foxo4	chrX:101254527-101260873	2.61188	1.68732	-0.630349	0.016917	yes
Ppie	chr4:123127124-123139941	35.0435	22.6405	-0.630242	0.00382257	yes
Fam83d	chr2:158768098-158786637	10.521	6.79833	-0.630016	0.00829754	yes
H2-M3	chr17:37270233-37274485	27.3049	17.6451	-0.629893	0.0034365	yes
Tusc3	chr8:39005866-39130817	38.2447	24.7163	-0.629795	0.00666366	yes
Hint1	chr11:54866437-54870496	611.67	395.604	-0.628695	0.00168106	yes
Ddx56	chr11:6257544-6267729	25.8482	16.7203	-0.628469	0.00257544	yes
Cdc20	chr4:118428092-118437343	69.2756	44.8246	-0.628056	0.0233549	yes
0610012G03Rik	chr16:31947050-31948521	20.2284	13.0974	-0.627104	0.00634725	yes
Ccs	chr19:4825365-4839322	13.1058	8.48711	-0.626854	0.010626	yes
Brat1	chr5:140705010-140719378	3.99288	2.58637	-0.6265	0.0146074	yes
Ppm1f	chr16:16896468-16927375	11.4728	7.43169	-0.626461	0.00168106	yes
Asb6	chr2:30823097-30828300	12.1696	7.88776	-0.625591	0.00257544	yes
Zfp324	chr7:12965863-12973822	5.60019	3.6315	-0.624913	0.00568302	yes
Pdcd2l	chr7:34184496-34196647	21.3526	13.8501	-0.624514	0.00797055	yes
Sdhd	chr9:50596339-50603849	214.118	138.889	-0.624471	0.0034365	yes
Slc45a4	chr15:73580290-73624744	4.27025	2.77017	-0.624348	0.0116752	yes
Gpx1	chr9:108339079-108340344	765.357	496.563	-0.624157	0.00382257	yes
Fam53a	chr5:33600352-33629635	15.5558	10.0985	-0.623323	0.00766564	yes
Hemk1	chr9:107327081-107338350	2.67964	1.74016	-0.62282	0.0332955	yes
Ripk3	chr14:55784994-55788857	9.84424	6.39753	-0.621766	0.0103347	yes
Hist1h3a	chr13:23761884-23762386	556.507	361.705	-0.621586	0.00257544	yes
Sarnp	chr10:128821770-128877638	9.22412	5.99543	-0.621548	0.0193303	yes
Rfc2	chr5:134582689-134598328	66.9002	43.4884	-0.621378	0.00217763	yes
Myo1f	chr17:33555706-33607764	45.4961	29.5863	-0.620814	0.00257544	yes
BC029722	chr2:155775343-155819203	19.5619	12.7236	-0.620533	0.00531709	yes
Erh	chr12:80634022-80643861	75.5016	49.1278	-0.619967	0.00419912	yes
Pwp2	chr10:78170909-78185149	19.4222	12.6414	-0.619548	0.00382257	yes
Sergef	chr7:46443158-46639807	13.1675	8.57575	-0.618648	0.0114308	yes
Hist1h2bn	chr13:21754122-21754553	443.835	289.105	-0.618428	0.0109203	yes
Ears2	chr7:122034161-122067086	2.55527	1.66452	-0.618372	0.0125053	yes
Ppp2r5b	chr19:6226400-6235840	3.01029	1.96149	-0.617951	0.0171867	yes
Cnpy2	chr10:128322458-128327187	47.3454	30.8518	-0.617873	0.00217763	yes
Rpa3	chr6:8255935-8259141	39.4581	25.7124	-0.617857	0.0133799	yes
Spr	chr6:85133679-85137764	43.2466	28.2024	-0.616769	0.00531709	yes
Smug1	chr15:103153289-103163284	3.15432	2.05716	-0.616675	0.0224583	yes
2310039H08Rik	chr17:46772634-46773407	23.2151	15.1422	-0.616487	0.0111526	yes
Dse	chr10:34151392-34207551	15.4026	10.049	-0.616118	0.00301495	yes
Dhodh	chr8:109593247-109608673	8.96211	5.84785	-0.615932	0.00829754	yes
Rbfa	chr18:80186238-80200619	43.752	28.5547	-0.615622	0.0034365	yes
Bbc3	chr7:16309582-16318334	3.11977	2.03615	-0.615597	0.0320864	yes
Slc25a1	chr16:17925210-17928219	28.0469	18.3059	-0.615529	0.00454713	yes
Nxt1	chr2:148672614-148676026	85.0013	55.4912	-0.615227	0.00454713	yes
Tcf3	chr10:80409164-80433653	19.3008	12.6032	-0.614866	0.00257544	yes
Pwwp2b	chr7:139248481-139267253	7.90632	5.16348	-0.614661	0.010626	yes

St6galnac4,St6gal	chr2:32587077-32620809	27.8079	18.1659	-0.614265	0.00797055	yes
Nudt16	chr9:105129337-105131805	5.06067	3.30634	-0.614091	0.0254961	yes
Agap2	chr10:127078906-127093170	0.786544	0.513886	-0.614079	0.037051	yes
Tsta3	chr15:75924682-75929730	23.6384	15.4522	-0.613324	0.00454713	yes
Ip6k1	chr9:108002647-108048782	10.4812	6.85196	-0.61322	0.00257544	yes
Actr1b	chr1:36699201-36709925	45.4459	29.7187	-0.61278	0.00168106	yes
Rassf1	chr9:107551554-107562267	18.553	12.1404	-0.611839	0.0100593	yes
Ncaph2	chr15:89355718-89377037	45.3701	29.6991	-0.611324	0.00382257	yes
2310047M10Rik	chr11:69059774-69061576	7.78374	5.09694	-0.610831	0.0127966	yes
Mien1	chr11:98412483-98438988	40.908	26.8045	-0.609907	0.00634725	yes
Atp5b	chr10:128083306-128090388	861.945	565.189	-0.608862	0.0193303	yes
Miip	chr4:147860777-147868719	11.4693	7.52503	-0.608008	0.00977864	yes
Pfdn5	chr15:102326115-102331489	69.7362	45.762	-0.607758	0.00454713	yes
Rfc5	chr5:117379144-117389023	41.7544	27.4094	-0.607259	0.00454713	yes
Bnip1	chr17:26781078-26792521	22.5389	14.7998	-0.606837	0.00858867	yes
Rtca	chr3:116488963-116508175	29.7055	19.5073	-0.606716	0.00531709	yes
Gtf3a	chr5:146948656-146963797	49.3365	32.4055	-0.606419	0.0261255	yes
Rgs14	chr13:55369731-55384687	9.19355	6.0392	-0.606264	0.0111526	yes
Dnaaf3	chr7:4522956-4532442	2.19444	1.44156	-0.606222	0.0397131	yes
Cdc25c	chr18:34732994-34751533	14.4907	9.51999	-0.606098	0.00531709	yes
Mogs	chr6:83115505-83118898	62.3874	40.9885	-0.606035	0.00634725	yes
Osgep	chr14:50915373-50924893	10.8704	7.14203	-0.605999	0.0116752	yes
Nudt22	chr19:6993018-6996037	10.1891	6.69577	-0.605707	0.0237709	yes
Bmyc	chr2:25706878-25707719	25.4663	16.7393	-0.605348	0.0127966	yes
Stk38l	chr6:146724929-146778814	8.15325	5.36005	-0.605129	0.00634725	yes
Coq10a	chr10:128363096-128370037	3.19993	2.10376	-0.605068	0.0275447	yes
Plgrkt	chr19:29348676-29361871	30.8334	20.2775	-0.60461	0.00915681	yes
Ercc8	chr13:108158737-108194981	9.49557	6.2453	-0.604484	0.0114308	yes
Adprh	chr16:38445398-38452689	27.4294	18.0437	-0.604228	0.00568302	yes
Rgs10	chr7:128373624-128418172	126.382	83.1401	-0.604177	0.0034365	yes
Pex16	chr2:92374675-92381220	8.65394	5.69455	-0.603773	0.0213708	yes
Dph2	chr4:117888642-117892003	7.06193	4.64844	-0.603317	0.0114308	yes
Rpl36al	chr12:69182733-69184067	274.587	180.785	-0.602987	0.00666366	yes
Camk1	chr6:113326975-113343922	48.7168	32.1196	-0.600966	0.00701693	yes
Dhrs1	chr14:55739019-55745684	11.9326	7.86866	-0.600715	0.0135968	yes
Anapc2	chr2:25272465-25285916	14.4726	9.54509	-0.600492	0.0116752	yes
Fcgrt	chr7:45092992-45103822	2.69139	1.77548	-0.600138	0.0492804	yes
Mdh1	chr11:21556691-21571934	174.928	115.407	-0.60003	0.00531709	yes
Pmf1	chr3:88394142-88410316	70.7197	46.66	-0.599926	0.00382257	yes
Sec14l2	chr11:4097039-4118729	1.85871	1.22681	-0.599387	0.0259255	yes
Tnni2	chr7:142442467-142444405	63.8403	42.1388	-0.599316	0.00666366	yes
Ino80b	chr6:83121827-83125029	12.3796	8.17149	-0.599298	0.017885	yes
Slc16a12	chr19:34668405-34747111	45.7675	30.2161	-0.599007	0.00257544	yes
Ncf2	chr1:152807889-152836990	28.2512	18.6534	-0.598876	0.00419912	yes
Fez2	chr17:78200247-78418152	11.224	7.41209	-0.598633	0.0161452	yes
Crot	chr5:8966047-8997146	10.47	6.91466	-0.598527	0.0119604	yes
Rplp0	chr5:115559466-115563729	377.61	249.413	-0.598357	0.00797055	yes
2010320M18Rik	chr8:70776861-70777606	7.50661	4.95974	-0.597897	0.0469332	yes
Cbr4	chr8:61487733-61503500	16.6496	11.0071	-0.597053	0.0195507	yes
2010111I01Rik	chr13:62964892-63431745	27.0782	17.902	-0.597007	0.0482593	yes
Tpst2	chr5:112276706-112315356	21.9653	14.5275	-0.596447	0.00886675	yes
Vdac3	chr8:22577074-22593813	27.9076	18.4659	-0.595793	0.00666366	yes
Hscb	chr5:110829069-110839777	22.3207	14.7715	-0.595564	0.0171867	yes
Naa38	chr11:69395790-69398234	112.713	74.6069	-0.595271	0.00886675	yes
Triap1	chr5:115341246-115343552	24.606	16.2977	-0.594341	0.0153915	yes
Hist1h3e	chr13:23561895-23562365	646.335	428.099	-0.594336	0.00454713	yes
Gsn	chr2:35256358-35307902	180.965	120.075	-0.591777	0.00454713	yes
Tor1b	chr2:30953000-30959015	17.7968	11.8187	-0.590547	0.00766564	yes
Tex261	chr6:83770413-83775812	22.56	14.9871	-0.590039	0.0141124	yes
Polr2g	chr19:8793128-8798557	53.8971	35.8157	-0.589617	0.00734768	yes
Lsm11	chr11:45928268-45944935	3.58713	2.38515	-0.588748	0.0130772	yes
Etfb	chr7:43444071-43457800	64.1897	42.6901	-0.588442	0.00382257	yes
Cstb	chr10:78425669-78427622	192.788	128.257	-0.587973	0.00257544	yes
Agpat2	chr2:26593056-26604417	27.2042	18.0983	-0.587972	0.00666366	yes
Coq9	chr8:94838416-94854895	29.0397	19.3291	-0.587255	0.00634725	yes

Oas1c	chr5:120800198-120812514	2.08412	1.38749	-0.586965	0.0415334	yes
Rsl24d1	chr9:73113468-73123333	41.0589	27.3532	-0.585986	0.00886675	yes
Tmem222	chr4:133266044-133277790	26.7947	17.8576	-0.585407	0.0119604	yes
Pgm2	chr4:99929450-99987294	28.2269	18.8242	-0.58448	0.00419912	yes
Rangap1	chr15:81704247-81729919	67.804	45.2201	-0.584406	0.00602054	yes
Anxa6	chr11:54978961-55033471	13.501	9.00618	-0.584084	0.00634725	yes
Brf1	chr12:112959861-113000621	17.1451	11.4409	-0.583601	0.0245865	yes
Als2cl	chr9:110880173-110900530	1.28518	0.857922	-0.583056	0.0364732	yes
Ppm1j	chr3:104781055-104786017	9.36415	6.2531	-0.582577	0.0193303	yes
Cdk9	chr2:32705781-32712784	16.4838	11.0108	-0.582139	0.0111526	yes
Nup35	chr2:80638811-80660071	7.54633	5.04121	-0.582005	0.017885	yes
Cebpa	chr7:35119292-35121931	9.04766	6.04471	-0.581871	0.0153915	yes
Daglb	chr5:143464492-143504442	14.0771	9.40785	-0.581415	0.00701693	yes
Eef2kmt	chr16:5233620-5255956	15.8949	10.6257	-0.58101	0.0474965	yes
Rab3d	chr9:21907510-21918121	3.20612	2.14336	-0.580957	0.0228852	yes
Zkscan6	chr11:65807174-65829239	5.17255	3.45802	-0.580928	0.018127	yes
Lrmp	chr6:145115635-145210970	68.6983	45.9373	-0.58061	0.00634725	yes
Dnase2a	chr8:84908623-84911461	41.3051	27.628	-0.58019	0.00382257	yes
Ppap2c	chr10:79526423-79533787	18.0473	12.0766	-0.57957	0.0141124	yes
Dnlz	chr2:26315532-26352110	6.97604	4.66866	-0.579399	0.0444182	yes
Ptges2	chr2:32395889-32402740	20.0527	13.4296	-0.578378	0.0111526	yes
Hdac5	chr11:102195746-102230172	15.1779	10.1652	-0.578333	0.00766564	yes
Klrg2	chr6:38626659-38637239	8.96365	6.00406	-0.578148	0.0135968	yes
Hist1h1e	chr13:23621776-23622558	730.455	489.34	-0.577957	0.00766564	yes
Pithd1	chr4:135975601-135987244	40.4498	27.1079	-0.577418	0.00568302	yes
Sh3tc1	chr5:35697179-35729276	9.50437	6.3709	-0.577093	0.00947319	yes
Zer1	chr2:30097282-30124611	1.85537	1.24371	-0.577051	0.0355411	yes
Clec4a2	chr6:123122689-123143999	9.95515	6.67418	-0.576853	0.0153915	yes
Edc3	chr9:57708568-57750162	7.20375	4.83282	-0.575883	0.0195507	yes
Bphl	chr13:34037640-34074074	9.00317	6.04007	-0.575867	0.0366609	yes
Sh3bp5l	chr11:58330706-58347728	8.7587	5.87645	-0.57577	0.0109203	yes
Snapc2	chr8:4253101-4256220	9.54201	6.40214	-0.575738	0.024169	yes
Hist1h3f	chr13:23544051-23544954	198.994	133.516	-0.575715	0.00915681	yes
Zfand1	chr3:10339955-10351301	6.10925	4.09958	-0.575517	0.0286094	yes
Aurkaip1	chr4:155831268-155833098	93.2352	62.5791	-0.575193	0.00634725	yes
Aurkb	chr11:69045642-69051662	62.9934	42.2856	-0.575034	0.00531709	yes
Ppox	chr1:171276991-171281186	4.09274	2.74734	-0.575031	0.0456964	yes
Cryl1	chr14:57275033-57398483	25.1473	16.8815	-0.574961	0.0159056	yes
Ccdc22	chrX:7558561-7605420	33.5375	22.5161	-0.574817	0.00666366	yes
Cmtm3	chr8:104340593-104347672	27.9521	18.7665	-0.5748	0.00602054	yes
Tuba1c	chr15:99029890-99038105	63.8574	42.8818	-0.57449	0.00858867	yes
Acad9	chr3:36065999-36092857	13.8885	9.33488	-0.573191	0.00734768	yes
Stx2	chr5:128984557-129008572	16.7122	11.2332	-0.573125	0.00797055	yes
Rdh11	chr12:79175550-79191819	22.8721	15.3781	-0.572712	0.0111526	yes
Cdc26	chr4:62394588-62408623	21.0876	14.1824	-0.572294	0.0148682	yes
Hnrnpa0	chr13:58125878-58128556	110.266	74.1613	-0.572245	0.00977864	yes
Toe1	chr4:116720954-116807559	20.297	13.6572	-0.571604	0.0193303	yes
Rabggta	chr14:55715876-55722176	8.81657	5.93382	-0.571256	0.0239707	yes
Ruvbl2	chr7:45421897-45434464	103.948	69.9612	-0.571239	0.00634725	yes
Zfp41	chr15:75616683-75625300	4.45866	3.00106	-0.571141	0.0166553	yes
Xbp1	chr11:5520640-5525993	147.266	99.1478	-0.570771	0.00915681	yes
Chpf	chr1:75474568-75479471	15.3536	10.3373	-0.570721	0.0151535	yes
Zfp593	chr4:134243305-134245873	12.0964	8.14435	-0.570701	0.0125053	yes
Ndfip1	chr18:38418974-38464406	69.2358	46.6252	-0.570409	0.00797055	yes
Nmnat3	chr9:98296582-98411428	4.90085	3.30087	-0.570188	0.0309976	yes
Nudt6	chr3:37404981-37419596	6.1273	4.12745	-0.570001	0.035173	yes
Lgals1	chr15:78926724-78930465	379.412	255.605	-0.569848	0.00858867	yes
Ptdss2	chr7:141131285-141156154	9.34612	6.29813	-0.569445	0.0176712	yes
Ing1	chr8:11555761-11563251	21.2877	14.3463	-0.569342	0.0103347	yes
Thap11	chr8:105855102-105856950	26.7728	18.0641	-0.567645	0.00734768	yes
Ndufa12	chr10:94199008-94220948	24.7853	16.7319	-0.56688	0.0248059	yes
Ppp4c	chr7:126785867-126792471	150.332	101.487	-0.566857	0.00915681	yes
Zfp740	chr15:102203644-102215610	18.304	12.3573	-0.566799	0.00634725	yes
Nrm	chr17:35861317-35865400	25.1162	16.96	-0.566485	0.0159056	yes
Grwd1	chr7:45825222-45830789	30.0929	20.3294	-0.565851	0.00829754	yes

Med30	chr15:52712444-52730431	69.6865	47.0836	-0.565656	0.00858867	yes
Idnk	chr13:58157648-58164693	6.77527	4.57821	-0.565495	0.0403634	yes
Ppcs	chr4:119418532-119422420	10.0741	6.8115	-0.564604	0.0384653	yes
Pdrg1	chr2:153008889-153015383	39.3708	26.6204	-0.564591	0.0114308	yes
Fam207a	chr10:77486654-77515813	17.716	11.9801	-0.564418	0.0116752	yes
Ttc7	chr17:87282885-87381770	24.9562	16.8764	-0.56439	0.00915681	yes
Ssu72	chr4:155704814-155733873	51.2844	34.685	-0.56421	0.00858867	yes
Zfp787	chr7:6131488-6155971	10.1033	6.83408	-0.564014	0.0197691	yes
Srebf1	chr11:60105012-60220604	17.843	12.072	-0.563691	0.0368647	yes
Haus8	chr8:71251123-71272590	32.3548	21.9024	-0.562892	0.010626	yes
Ubtd1	chr19:41981762-42034641	14.3562	9.71921	-0.56276	0.017885	yes
Fam105a	chr15:27655070-27681542	38.1538	25.8406	-0.562186	0.00634725	yes
Dpp7	chr2:25352289-25356332	18.8553	12.7704	-0.562165	0.0141124	yes
Pttg1	chr11:43420247-43426248	77.9558	52.8372	-0.561101	0.0100593	yes
Klhl41	chr2:69670119-69684239	4.54266	3.08067	-0.560295	0.0300881	yes
Gm14403	chr2:177498225-177512311	2.36059	1.60136	-0.559847	0.0337719	yes
Scarna13	chr12:105030616-105032279	1242.79	843.185	-0.559658	0.0049451	yes
Fem1a	chr17:56256792-56263608	17.0295	11.5611	-0.558752	0.00734768	yes
Rpl32	chr6:115774556-115808743	649.705	441.365	-0.557812	0.00977864	yes
Atp6v1g1	chr4:63544764-63550701	111.641	75.8439	-0.557764	0.00829754	yes
Arfgap2	chr2:91265114-91277371	10.7497	7.3037	-0.557598	0.0153915	yes
Atp5g3	chr2:73908446-73911326	448.557	304.826	-0.557303	0.0111526	yes
Espnl	chr1:91322074-91348303	1.62591	1.10534	-0.556752	0.0309976	yes
Spag5	chr11:78301590-78322454	29.8409	20.2933	-0.556287	0.00797055	yes
Hist2h2bb	chr3:96269699-96270192	4852.08	3300.83	-0.555775	0.0332955	yes
Rcc1	chr4:132331918-132345750	51.636	35.1299	-0.555679	0.00701693	yes
Npepl1	chr2:174110350-174122702	61.7896	42.0381	-0.555666	0.00858867	yes
Ndufs4	chr13:114287794-114388094	43.6679	29.7123	-0.555512	0.0141124	yes
Stk19	chr17:34823992-34836903	10.8961	7.4182	-0.554666	0.0453196	yes
Gss	chr2:155563180-155592810	11.9056	8.11452	-0.55306	0.0156727	yes
Bola2	chr7:126695999-126696693	332.427	226.622	-0.55275	0.00734768	yes
Nit2	chr16:57156664-57167332	30.14	20.5473	-0.55273	0.0148682	yes
Myl6	chr10:128490860-128493825	13.451	9.17276	-0.55228	0.0418725	yes
Mrpl55	chr11:59202485-59206135	22.7385	15.5069	-0.552225	0.024169	yes
Eri3	chr4:117550323-117674297	36.9534	25.2011	-0.552218	0.013835	yes
H2-Ke6	chr17:34026032-34028055	25.4646	17.368	-0.552059	0.0193303	yes
Kifc5b	chr17:26917090-26932579	7.04733	4.80673	-0.552023	0.0275447	yes
Slc25a33	chr4:149744035-149774267	9.18567	6.26786	-0.551412	0.0323053	yes
Fam109a	chr5:121849027-121854599	4.48916	3.06325	-0.551382	0.0451403	yes
Wrap53	chr11:69561753-69579324	7.75769	5.29442	-0.551156	0.0277512	yes
Snhg20	chr11:117076782-117078955	21.1259	14.421	-0.550844	0.0444182	yes
Uqcc1	chr2:155846885-155930310	14.5037	9.90213	-0.550612	0.0111526	yes
Dvl1	chr4:155847316-155863353	16.2081	11.0666	-0.5505	0.0130772	yes
Txn1	chr4:57943372-57956411	394.921	269.731	-0.550042	0.0133799	yes
Stoml2	chr4:43027689-43031384	47.7856	32.6393	-0.549965	0.00947319	yes
Uqcrc1	chr9:108936647-108949641	173.431	118.538	-0.549007	0.00734768	yes
Zbtb48	chr4:152019775-152027671	3.58334	2.45028	-0.548358	0.0413811	yes
Cks2	chr13:51645231-51650662	42.7705	29.2491	-0.548222	0.0261255	yes
Necap2	chr4:141066511-141078345	30.3855	20.7843	-0.547893	0.00797055	yes
3010026O09Rik	chr11:50174850-50200115	8.56933	5.86307	-0.547527	0.0298919	yes
Rnpepl1	chr1:92910824-92920585	9.74069	6.66717	-0.546949	0.0174052	yes
Phc2	chr4:128654701-128752881	16.4384	11.2524	-0.546833	0.0103347	yes
Prdx3	chr19:60864065-60874538	132.826	90.9228	-0.546825	0.0119604	yes
Man1c1	chr4:134561689-134704290	7.16873	4.90766	-0.54668	0.0143874	yes
Mrps14	chr1:160195259-160201186	54.7231	37.4647	-0.546618	0.0143874	yes
Mtg2	chr2:180070592-180085902	13.3902	9.16752	-0.546571	0.0261255	yes
Speg	chr1:75375296-75432306	27.0605	18.5308	-0.546266	0.0332955	yes
Polr1d	chr5:147077345-147111361	119.268	81.6963	-0.545861	0.00947319	yes
Zfp956	chr6:47943174-47965299	3.36725	2.30656	-0.545827	0.040848	yes
Tomm5	chr4:45105209-45108113	130.911	89.7221	-0.545053	0.0119604	yes
Sqle	chr15:59315091-59331193	47.4482	32.5339	-0.544408	0.00886675	yes
B4gat1	chr19:5038825-5041134	11.4217	7.83337	-0.54407	0.0215983	yes
Nudt19	chr7:35547184-35555928	58.1138	39.8615	-0.543883	0.0127966	yes
Gmpr	chr13:45507443-45546386	30.7446	21.0908	-0.543718	0.0114308	yes
Pef1	chr4:130107555-130128134	17.1919	11.7963	-0.543399	0.0213708	yes

Lzic	chr4:149485332-149496667	15.4018	10.5795	-0.541831	0.0188838	yes
Sys1	chr2:164460970-164465510	23.0428	15.8369	-0.541027	0.0209453	yes
Wrb	chr16:96145418-96157852	21.8577	15.0273	-0.540554	0.0174052	yes
Dgcr2	chr16:17840355-17891728	14.6157	10.0533	-0.539852	0.0148682	yes
Afp	chr5:90490713-90508907	5.22259	3.59309	-0.539539	0.0466216	yes
Cenpl	chr1:161070766-161086724	18.8248	12.9517	-0.539487	0.0207355	yes
9130401M01Rik	chr15:58022270-58034294	38.1212	26.2317	-0.539281	0.0143874	yes
Casp9	chr4:141793611-141826003	7.80937	5.37615	-0.538633	0.0213708	yes
Fbxo31	chr8:121549442-121578806	4.99762	3.44054	-0.538603	0.0228852	yes
Emc6	chr11:73175502-73177042	72.4308	49.8674	-0.538507	0.0135968	yes
Dctn2	chr10:127266261-127288771	42.4782	29.2528	-0.538147	0.0146074	yes
Dpm3	chr3:89266460-89267079	53.3175	36.729	-0.537687	0.0250369	yes
Wdpcp	chr11:21572280-21898686	5.08859	3.50598	-0.537449	0.0340016	yes
Akr7a5	chr4:139310743-139318786	10.4343	7.19076	-0.537114	0.027115	yes
Ube2m	chr7:13035119-13038275	72.2134	49.7755	-0.536832	0.0111526	yes
Fuca1	chr4:135920725-135940300	26.8083	18.4858	-0.536262	0.0153915	yes
Galk2	chr2:125859108-126152004	26.3862	18.2113	-0.534947	0.0100593	yes
Dffa	chr4:149104141-149120653	9.90356	6.83604	-0.534786	0.0455374	yes
Pald1	chr10:61319656-61383523	9.57654	6.61367	-0.534052	0.0141124	yes
Gpn2	chr4:133584372-133591735	16.2227	11.2059	-0.533762	0.0275447	yes
Zdhhc7	chr8:120081094-120101472	14.1972	9.81214	-0.53297	0.0193303	yes
Tango2	chr16:18300824-18343932	6.76849	4.68169	-0.531805	0.0388425	yes
Btbd2	chr10:80642616-80657071	4.95708	3.42929	-0.53158	0.036006	yes
Yif1a	chr19:5088537-5098521	28.8432	19.9538	-0.531566	0.018127	yes
Dgkg	chr16:22466568-22657231	3.8631	2.67279	-0.531413	0.0176712	yes
Gucd1	chr10:75506813-75517322	6.16336	4.26801	-0.530154	0.029439	yes
Mettl17	chr14:51884841-51891868	13.0421	9.03231	-0.530012	0.0332955	yes
Fbxo46	chr7:19119858-19138261	3.91877	2.71508	-0.529407	0.0372196	yes
Mtx1	chr3:89209080-89214335	46.7703	32.4045	-0.529398	0.0153915	yes
Plrg1	chr3:83055537-83072291	61.9457	42.9346	-0.528864	0.013835	yes
Mgat1	chr11:49244190-49263024	13.2449	9.18236	-0.528502	0.0257373	yes
Klhl22	chr16:17759620-17793382	11.2199	7.77877	-0.528448	0.0191506	yes
Pi4k2a	chr19:42090434-42122218	15.1737	10.5208	-0.528323	0.0200293	yes
Snx11	chr11:96767548-96777555	8.18069	5.67257	-0.52822	0.0363016	yes
Rps25	chr9:44407713-44418007	108.846	75.4761	-0.528201	0.0183466	yes
Kifc1	chr17:33875665-33890633	16.4191	11.3882	-0.527835	0.0125053	yes
Fbxo25	chr8:13907805-13940521	5.23184	3.63074	-0.527058	0.0464453	yes
Sin3b	chr8:72723269-72758203	49.2832	34.2045	-0.526911	0.0363016	yes
Ndufb7	chr8:83566757-83571623	193.42	134.264	-0.526659	0.0116752	yes
Asb13	chr13:3634031-3651779	3.36844	2.3383	-0.52662	0.0478434	yes
Hist2h3b	chr3:96268653-96269155	614.612	427.002	-0.525433	0.0103347	yes
Eml3	chr19:8929693-8941582	10.4799	7.28131	-0.525352	0.0205128	yes
Plin3	chr17:56278961-56290511	13.6551	9.48753	-0.525339	0.0183466	yes
Zfp692	chr11:58307068-58314613	7.27721	5.05857	-0.524656	0.0384653	yes
Ciapin1	chr8:94819817-94838340	10.0965	7.01884	-0.524552	0.0220255	yes
Slc2a3	chr6:122727808-122742745	10.3868	7.22104	-0.52447	0.0159056	yes
Sdf2l1	chr16:17130137-17132383	130.403	90.6616	-0.524409	0.0161452	yes
Chmp7	chr14:69716978-69732570	21.3222	14.8268	-0.524146	0.0151535	yes
Akip1	chr7:109703736-109723771	23.8462	16.5823	-0.524116	0.0193303	yes
Saal1	chr7:46686107-46710651	9.64564	6.71087	-0.523379	0.0166553	yes
Wdfy2	chr14:62837689-62956886	35.8792	24.9732	-0.522766	0.0286094	yes
Slc19a1	chr10:77032738-77050432	26.8739	18.7056	-0.522733	0.0153915	yes
Mrpl21	chr19:3283046-3292837	21.1808	14.7461	-0.522422	0.0316924	yes
Tsc22d3	chrX:140539528-140600522	8.6614	6.0304	-0.522345	0.0361626	yes
Cdkn3	chr14:46760540-46771525	28.5607	19.8873	-0.522185	0.0300881	yes
Xylt2	chr11:94663846-94677493	4.88534	3.4026	-0.521821	0.0309976	yes
Mrpl45	chr11:97315715-97329920	35.2397	24.5552	-0.521176	0.0148682	yes
Mau2	chr8:70016122-70042734	13.7895	9.61005	-0.520957	0.0161452	yes
Tmco1	chr1:167308669-167333978	35.0959	24.4601	-0.520874	0.0116752	yes
Unc50	chr1:37430171-37439124	57.7975	40.2936	-0.520458	0.0220255	yes
Aar2	chr2:156547575-156568972	9.83225	6.85613	-0.520128	0.0224583	yes
Coq4	chr2:29788262-29797743	6.92511	4.83016	-0.519765	0.0345906	yes
Akt2	chr7:27591559-27639453	7.88835	5.50235	-0.519677	0.0243535	yes
Apobec1	chr6:122577791-122602444	74.2664	51.8155	-0.519327	0.0130772	yes
Mpeg1	chr19:12460778-12465285	46.1375	32.1974	-0.518994	0.0114308	yes

Mrpl13	chr15:55534094-55557312	120.003	83.7544	-0.518836	0.0119604	yes
Arsa	chr15:89472475-89484850	3.04686	2.12685	-0.518606	0.0437076	yes
Unc45a	chr7:80325291-80340219	6.89811	4.8171	-0.518036	0.0343789	yes
Bin2	chr15:100641081-100669500	13.6597	9.53921	-0.517982	0.0127966	yes
D2Wsu81e	chr2:30171523-30178459	25.909	18.1142	-0.516335	0.0494491	yes
Nostrin	chr2:69135799-69189329	16.4032	11.4694	-0.516185	0.0205128	yes
Sdhc	chr1:171129156-171150603	153.68	107.469	-0.516007	0.0171867	yes
Med11	chr11:70451930-70453726	36.8376	25.7677	-0.515612	0.0226785	yes
Idh1	chr1:65158615-65186479	65.7542	46.0003	-0.515439	0.0146074	yes
Atf6b	chr17:34647145-34655074	25.8717	18.1039	-0.515077	0.017885	yes
Maea	chr5:33335571-33373294	46.9659	32.9005	-0.513506	0.0127966	yes
Yeats4	chr10:117215140-117224507	53.7038	37.6246	-0.513349	0.0195507	yes
Rrp9	chr9:106477308-106485415	43.9382	30.7846	-0.513268	0.0164013	yes
Rnf167	chr11:70647588-70651414	14.2033	9.95138	-0.513254	0.0364732	yes
Fastk	chr5:24441039-24445235	18.0489	12.6462	-0.5132	0.0327099	yes
Slc35e4	chr11:3907021-3914664	30.3121	21.2421	-0.512971	0.0156727	yes
Slc25a39	chr11:102402975-102407517	84.8233	59.444	-0.512929	0.0143874	yes
Fgd3	chr13:49263109-49309208	14.0418	9.84471	-0.512308	0.0174052	yes
Slc25a38	chr9:120110398-120124319	19.0517	13.3587	-0.512135	0.0343789	yes
Csnk1g2	chr10:80622779-80640771	52.5079	36.8228	-0.511936	0.0109203	yes
Mrps35	chr6:147042769-147070902	68.3152	47.9087	-0.511921	0.0156727	yes
Fam78a	chr2:32066884-32083705	4.5691	3.20583	-0.511212	0.0393598	yes
Lamtor2	chr3:88549818-88552927	99.4988	69.8455	-0.510511	0.0195507	yes
Leprot	chr4:101647782-101659358	14.8777	10.4473	-0.510017	0.0328955	yes
Jagn1	chr6:113442516-113448229	53.3314	37.4541	-0.509862	0.0261255	yes
Myom1	chr17:71019556-71126856	2.12652	1.49371	-0.509596	0.0478434	yes
Asf1b	chr8:83955693-83970195	64.2232	45.1607	-0.508025	0.0130772	yes
Fbxw8	chr5:118064980-118155458	18.739	13.1771	-0.508015	0.0195507	yes
Agmo	chr12:37241638-37581932	11.8612	8.34091	-0.507972	0.0375376	yes
Ube2e1	chr14:18271141-18331844	43.6558	30.713	-0.507327	0.0417032	yes
Scd2	chr19:44293675-44306862	151.208	106.403	-0.506995	0.0209453	yes
Coasy	chr11:101082564-101086619	15.9356	11.2153	-0.506782	0.0319128	yes
Tsr3	chr17:25240169-25256364	16.4159	11.5559	-0.506465	0.0440544	yes
Rnaseh2a	chr8:84956609-84966011	20.1435	14.1843	-0.506014	0.0248059	yes
Galnt11	chr5:25222892-25265918	19.386	13.6518	-0.505924	0.0211532	yes
Tbcc	chr17:46890620-46892463	13.4135	9.44651	-0.505832	0.0397131	yes
Mthfsd	chr8:121097556-121108379	11.5521	8.13577	-0.505808	0.0314714	yes
Arhgef10l	chr4:140514484-140665905	18.7598	13.2123	-0.505764	0.0174052	yes
Fam167b	chr4:129576814-129578580	64.4345	45.3913	-0.505418	0.0202883	yes
Tm2d2	chr8:25017210-25023260	53.2355	37.5324	-0.504253	0.0218243	yes
Aprt	chr8:122574636-122576907	329.401	232.253	-0.504146	0.0161452	yes
Smpd1	chr7:105554359-105558389	6.51949	4.59868	-0.503539	0.0413811	yes
Zranb3	chr1:127954178-128103047	6.58206	4.64453	-0.503005	0.0277512	yes
Slc25a5	chrX:36795596-36798808	581.395	410.417	-0.502426	0.0312197	yes
Zfp954	chr7:7114682-7121476	7.19567	5.08262	-0.501557	0.0422828	yes
Thap7	chr16:17527981-17531052	26.1768	18.4924	-0.501353	0.0277512	yes
Rpp21	chr17:36255672-36257846	41.5393	29.3646	-0.500396	0.0481279	yes
Aamp	chr1:74279839-74284738	82.9143	58.6173	-0.500294	0.0146074	yes
Chchd4	chr6:91464275-91473423	65.3391	46.194	-0.500241	0.024169	yes
Chrac1	chr15:73090411-73094075	66.496	47.0269	-0.499782	0.0231387	yes
Dok1	chr6:83030935-83033471	21.9679	15.5387	-0.499533	0.0266413	yes
Ndufa6	chr15:82350138-82354291	184.154	130.302	-0.499052	0.018127	yes
Ndufa9	chr6:126821862-126849144	96.6151	68.4077	-0.498089	0.0205128	yes
Ddx49	chr8:70282998-70302452	33.1259	23.4747	-0.496855	0.0298919	yes
Cd300a	chr11:114890040-114904651	21.0962	14.9502	-0.496815	0.017885	yes
Pus1	chr5:110773666-110780615	36.7284	26.0505	-0.495583	0.0239707	yes
Cd320	chr17:33843090-33849774	12.3506	8.76066	-0.495465	0.0417032	yes
Lsm8	chr6:18848634-18854052	91.2042	64.7005	-0.495324	0.0316924	yes
AI467606	chr7:127091435-127094049	10.5413	7.47963	-0.495016	0.0397131	yes
Ndst2	chr14:20723729-20734562	7.33889	5.20793	-0.494852	0.036006	yes
Hist4h4	chr6:136803992-136804431	2207.51	1566.81	-0.494586	0.0320864	yes
Syne3	chr12:104929932-105009809	23.4318	16.6312	-0.494574	0.0268919	yes
Rassf2	chr2:131992849-132029988	12.5599	8.9194	-0.493802	0.0200293	yes
Nsmce4a	chr7:130532525-130547381	119.663	84.9845	-0.493701	0.0164013	yes
Mrpl11	chr19:4962305-4966995	20.16	14.3191	-0.493552	0.0207355	yes

Anapc5	chr5:122787460-122821342	134.625	95.7317	-0.491873	0.0254961	yes
Suds3	chr5:117091677-117115993	53.8746	38.3213	-0.49146	0.0211532	yes
Nr1h2	chr7:44549615-44553965	14.2893	10.1658	-0.491209	0.0432835	yes
Bod1	chr11:31665149-31671862	21.3439	15.1913	-0.490575	0.040848	yes
Pfkfb4	chr9:108991901-109032225	5.64519	4.0183	-0.490435	0.040848	yes
Hmgn1	chr16:96121587-96127725	271.545	193.361	-0.489896	0.0209453	yes
Ncln	chr10:81486458-81496363	25.6051	18.2367	-0.489588	0.0228852	yes
Oxa1l	chr14:54360840-54417702	20.6692	14.7234	-0.48937	0.032539	yes
Abhd12	chr2:150832514-150904731	49.5989	35.3461	-0.488758	0.0205128	yes
Col4a5	chrX:141475418-141689235	9.89863	7.06047	-0.487464	0.0243535	yes
Ppp1r8	chr4:132826923-132843169	48.2554	34.4273	-0.487135	0.0239707	yes
Gga2	chr7:121986721-122021198	6.77328	4.83479	-0.486399	0.0361626	yes
1700020I14Rik	chr2:119594295-119600744	25.5094	18.2112	-0.486201	0.0490909	yes
Fh1	chr1:175601377-175625635	173.608	123.954	-0.486028	0.0193303	yes
Tbc1d9b	chr11:50131359-50172785	8.73723	6.24237	-0.485082	0.0228852	yes
Gabarap	chr11:69991369-69994949	101.33	72.4195	-0.484616	0.017885	yes
Hnrnpl	chr7:28810889-28822266	154.233	110.245	-0.48439	0.0252797	yes
Ctsd	chr7:142375915-142387870	305.705	218.559	-0.484112	0.0355411	yes
Traf2	chr2:25517981-25546940	10.1221	7.2371	-0.484025	0.0405196	yes
Dnajb14	chr3:137867674-137908931	23.7182	16.9598	-0.483882	0.0368647	yes
Marveld1	chr19:42147388-42151703	12.6449	9.0441	-0.483501	0.0418725	yes
Lias	chr5:65391496-65409207	20.7767	14.8612	-0.483421	0.0332955	yes
Acad11	chr9:104063702-104127646	8.0788	5.77949	-0.483199	0.0363016	yes
Gramd4	chr15:86057694-86137636	10.7258	7.67695	-0.48248	0.0292401	yes
Ninj1	chr13:49187546-49196251	38.8611	27.8478	-0.480763	0.0380686	yes
Cdc34	chr10:79682194-79688398	47.091	33.7458	-0.480744	0.0300881	yes
Fcrl1	chr3:87376386-87392133	39.3995	28.2353	-0.480676	0.0243535	yes
Zfp180	chr7:24081896-24107708	10.7999	7.7398	-0.480654	0.0237709	yes
Aes	chr10:81559443-81566371	65.6672	47.0704	-0.480354	0.0235697	yes
Abhd16a	chr17:35089290-35102987	23.7538	17.0354	-0.479617	0.0281891	yes
Rtfdc1	chr2:172440577-172469899	31.4691	22.5704	-0.479507	0.0245865	yes
Psma1	chr7:114264549-114276116	258.299	185.327	-0.478969	0.0292401	yes
Smdt1	chr15:82346045-82349062	170.125	122.111	-0.478402	0.0224583	yes
Acadvl	chr11:70010182-70015428	28.2582	20.2866	-0.478142	0.0259255	yes
Depdc1b	chr13:108316336-108389557	10.5086	7.54542	-0.477898	0.0469332	yes
Golm1	chr13:59634995-59675784	30.9365	22.2138	-0.477855	0.0231387	yes
Mccc2	chr13:99948531-100015639	13.433	9.65101	-0.477026	0.0403634	yes
Pepd	chr7:34912406-35044708	52.3411	37.6087	-0.476875	0.0259255	yes
Grk6	chr13:55445071-55460927	30.4606	21.8969	-0.476221	0.027115	yes
Cox19	chr5:139337821-139345166	28.1722	20.255	-0.475995	0.0470882	yes
Ifi30	chr8:70762772-70766663	264.569	190.28	-0.475521	0.0252797	yes
Shq1	chr6:100571810-100671157	8.75584	6.30756	-0.473162	0.0484508	yes
Cdk4	chr10:127063602-127067282	301.032	216.927	-0.472705	0.0361626	yes
Rnf25	chr1:74593747-74601397	15.8394	11.4167	-0.472368	0.0393598	yes
Man1b1	chr2:25332742-25352213	17.3484	12.5045	-0.472353	0.0277512	yes
Ccm2	chr11:6546886-6596761	19.676	14.1845	-0.472127	0.0388425	yes
Ncf4	chr15:78244810-78262580	79.2893	57.1807	-0.471599	0.0303127	yes
Zfp651	chr9:121760032-121771742	3.50214	2.52569	-0.471557	0.0456964	yes
Sirt7	chr11:120618371-120625002	23.4494	16.9182	-0.470973	0.0376868	yes
Gtf2h3	chr5:124579147-124597680	15.0909	10.8896	-0.470724	0.0407291	yes
Cog4	chr8:110847023-110882234	17.2256	12.4304	-0.470682	0.0376868	yes
Egln2	chr7:27158657-27166802	23.0317	16.6231	-0.47043	0.0327099	yes
Fam134a	chr1:75142785-75147909	50.3545	36.37	-0.469372	0.0263902	yes
Gpr108	chr17:57234914-57247689	17.2948	12.4933	-0.469186	0.0388425	yes
Ubxn6	chr17:56068252-56074989	22.0881	15.956	-0.469169	0.0364732	yes
Urm1	chr2:29827388-29844996	20.4785	14.7964	-0.46886	0.0397131	yes
Dmap1	chr4:117674685-117682225	16.2108	11.7187	-0.46815	0.0417032	yes
Snx25	chr8:46033260-46124146	20.4778	14.8034	-0.468131	0.0342148	yes
Ldlr	chr9:21723575-21749918	20.4003	14.75	-0.46788	0.0250369	yes
Atg101	chr15:101284300-101290934	26.4207	19.117	-0.466808	0.0466216	yes
Atg4b	chr1:93755032-93789529	18.6648	13.5089	-0.466407	0.0342148	yes
Mrps34	chr17:24895119-24896273	46.02	33.317	-0.466001	0.0372196	yes
Wdr45b	chr11:121327202-121354447	19.809	14.3498	-0.465126	0.0376868	yes
Papss1	chr3:131564767-131643671	25.1752	18.2634	-0.463051	0.0379037	yes
Lgmn	chr12:102394097-102439697	223.495	162.149	-0.462929	0.0486014	yes

Rps6ka4	chr19:6829083-6840627	20.1699	14.6335	-0.462929	0.040848	yes
Sh3glb2	chr2:30344776-30359316	23.1798	16.8295	-0.461875	0.0342148	yes
Itpk1	chr12:102568582-102704869	9.06835	6.58546	-0.461555	0.0420778	yes
Zfp277	chr12:40315045-40445790	18.2804	13.2765	-0.461422	0.0440544	yes
Nop10	chr2:112261925-112262898	233.752	169.797	-0.461163	0.0323053	yes
Adk	chr14:21052573-21448569	90.9362	66.0606	-0.461065	0.0252797	yes
Guk1	chr11:59183854-59191952	46.4588	33.7652	-0.460416	0.0366609	yes
Impdh1	chr6:29200436-29216364	19.673	14.2996	-0.460247	0.0428769	yes
Lancl1	chr1:67000516-67038872	11.3789	8.27277	-0.459919	0.0459136	yes
Pkn1	chr8:83666639-83699179	23.8932	17.3789	-0.459264	0.0487312	yes
Pgam5	chr5:110259134-110269899	35.3273	25.7379	-0.456892	0.0469332	yes
Ppp2r1a	chr17:20945453-20965905	59.7431	43.5456	-0.456245	0.0361626	yes
Msh2	chr17:87672556-87723713	59.8564	43.6424	-0.455773	0.0353656	yes
Cpsf4	chr5:145167212-145182041	24.5783	17.9315	-0.454887	0.0437076	yes
Rassf3	chr10:121410350-121476250	22.2651	16.2442	-0.454863	0.0380686	yes
Actr10	chr12:70937856-70964717	22.2267	16.2165	-0.454828	0.0415334	yes
Npc2	chr12:84754558-84773112	67.7347	49.4343	-0.454383	0.0363016	yes
Trabd	chr15:89076063-89087075	21.2532	15.5121	-0.454282	0.0386629	yes
Galnt2	chr8:124231393-124345723	14.2421	10.3976	-0.453912	0.035173	yes
Arpc1b	chr5:145114255-145128186	357.165	260.995	-0.452568	0.0415334	yes
Cant1	chr11:118406288-118419118	13.3705	9.77233	-0.45228	0.0460913	yes
Dap3	chr3:88920802-88950282	18.155	13.2698	-0.452222	0.0413811	yes
Rab1b	chr19:5099206-5106996	83.0634	60.714	-0.452184	0.0364732	yes
Wdr4	chr17:31494321-31512487	15.7275	11.5067	-0.450819	0.047996	yes
Atp6v1e1	chr6:120795243-120822685	78.1242	57.1841	-0.450157	0.0355411	yes
Kbtbd11	chr8:15011024-15033332	38.4035	28.1162	-0.449836	0.0412463	yes
Gpn1	chr5:31494760-31511627	39.4409	28.8923	-0.449004	0.0413811	yes
Dyrk3	chr1:131128440-131138234	28.6174	20.971	-0.448497	0.0435051	yes
Tmem144	chr3:79813152-79842662	16.8168	12.3316	-0.447545	0.0499321	yes
Mrpl2	chr17:46646247-46650132	86.0421	63.1073	-0.447236	0.0372196	yes
Scamp2	chr9:57560943-57588798	28.7221	21.0684	-0.447082	0.0497441	yes
Gtf2h5	chr17:6079827-6085485	61.0756	44.8156	-0.446596	0.0424874	yes
Xrcc6	chr15:82016368-82040084	44.9027	32.9655	-0.445846	0.0364732	yes
Nudt5	chr2:5845033-5868736	75.7295	55.6116	-0.445471	0.0355411	yes
Cfp	chrX:20925534-20931524	125.017	91.9419	-0.443334	0.0397131	yes
Mrps9	chr1:42851232-42905683	63.6753	46.8692	-0.442095	0.0375376	yes
Slc38a10	chr11:120103950-120151351	24.7156	18.1952	-0.441864	0.0432835	yes
Pcyox1	chr6:86386005-86397150	14.1516	10.4185	-0.441812	0.0440544	yes
Sat1	chrX:155213125-155216449	55.8212	41.118	-0.441042	0.0482593	yes
Psmd13	chr7:140882393-140898642	103.874	76.5557	-0.440253	0.0453196	yes
Uqcrh	chr4:116066964-116075070	431.172	317.965	-0.439392	0.0405196	yes
Otulin	chr15:27605920-27630693	43.8827	32.3687	-0.439052	0.0426916	yes
Copg2	chr6:30733505-30896794	25.2735	18.6446	-0.438866	0.0478434	yes
Ttf2	chr3:100938859-100969663	17.1923	12.6869	-0.438423	0.0418725	yes
Dram1	chr10:88322803-88357075	30.02	22.1593	-0.438014	0.0438907	yes
Pnpla7	chr2:24976032-25054072	11.579	8.54738	-0.437962	0.0490909	yes
Rnpep	chr1:135262698-135284084	71.4822	52.8441	-0.435842	0.0445611	yes
Ctbp1	chr5:33247722-33275004	87.4581	64.6707	-0.43548	0.0482593	yes
Nsmce1	chr7:125467639-125491542	48.6709	35.9918	-0.435392	0.0489214	yes
Apeh	chr9:108085413-108094480	18.8093	13.9103	-0.435294	0.047996	yes
Hist2h2ab	chr3:96219915-96220353	200.264	148.177	-0.434582	0.0497441	yes
Vps11	chr9:44348104-44361670	16.3262	12.0844	-0.434047	0.0496379	yes
Smyd2	chr1:189880491-189922288	46.4466	34.3878	-0.433675	0.0428769	yes
Scarb1	chr5:125277086-125341094	37.8239	28.0265	-0.432505	0.0420778	yes
Rpa2	chr4:132768359-132778746	61.1833	45.3916	-0.430713	0.0451403	yes
Samd1	chr8:83997671-84000386	46.8384	34.8098	-0.428196	0.0426916	yes
Chchd1	chr14:20702011-20704425	131.209	97.6234	-0.426572	0.0486014	yes
Ndufv1	chr19:4007498-4012755	80.6385	60.2005	-0.421694	0.0451403	yes
Lamc2	chr1:153122755-153186447	0.0311318	1.08237	5.11966	0.00064883	yes
Egr1	chr18:34861206-34864956	0.966744	32.1181	5.05411	0.00064883	yes
Cxcl2	chr5:90903898-90905938	0.881604	17.5825	4.31787	0.00064883	yes
Flrt3	chr2:140395429-142390050	0.123078	1.78155	3.85549	0.00064883	yes
Plk2	chr13:110395043-110400843	0.901843	12.5358	3.79703	0.00064883	yes
Egr2	chr10:67537868-67542188	1.17793	9.51729	3.01429	0.00064883	yes
Il1a	chr2:129297369-129309972	0.131639	1.02387	2.95937	0.00118536	yes

Ccrl2	chr9:111054833-111057518	0.216661	1.57862	2.86516	0.00064883	yes
Csf1	chr3:107741047-107760469	0.133589	0.972646	2.86412	0.00064883	yes
Areg	chr5:91139614-91148432	0.0721868	0.511694	2.82548	0.0373535	yes
Kdm6b	chr11:69398517-69413675	0.244775	1.71439	2.80817	0.00064883	yes
1810011O10Rik	chr8:24437615-24438946	0.136412	0.904614	2.72933	0.0049451	yes
Arc	chr15:74669080-74672570	1.15488	7.44407	2.68835	0.00064883	yes
Mir155,Mir155hg	chr16:84713022-84715244	0.966611	6.21823	2.6855	0.00064883	yes
Irg1	chr14:103047011-103056573	0.675928	4.29679	2.66832	0.00064883	yes
Phlda1	chr10:111506285-111508649	0.26387	1.58879	2.59003	0.00064883	yes
Map1b	chr13:99421463-99516602	0.168379	0.941302	2.48295	0.00064883	yes
Rtp4	chr16:23609918-23614222	0.324537	1.80335	2.47423	0.00064883	yes
Bcl2a1a	chr9:88956919-88962416	0.188447	1.04655	2.47341	0.0281891	yes
Ccl7	chr11:82045711-82047523	0.414603	2.26312	2.44851	0.00118536	yes
Oas2	chr5:120730332-120749848	0.639064	3.33762	2.38479	0.00064883	yes
Olr1	chr6:129485246-129507165	0.204135	1.00842	2.3045	0.00064883	yes
Il7r	chr15:9506158-9529876	0.210969	1.00647	2.25419	0.00064883	yes
Arid5a	chr1:36307732-36324029	0.323573	1.51517	2.22732	0.00064883	yes
Ifit1	chr19:34640888-34650009	0.234765	1.09606	2.22304	0.00064883	yes
Gem	chr4:11704446-11714993	0.242297	1.12258	2.21197	0.00064883	yes
Ppp1r15a	chr7:45473562-45526268	3.74796	16.4608	2.13485	0.00257544	yes
Tnfaip3	chr10:19000909-19015410	1.84529	8.02907	2.12138	0.00064883	yes
Myc	chr15:61985340-61990361	1.32505	5.73716	2.11429	0.00064883	yes
Dusp5	chr19:53529317-53541322	1.63352	6.8969	2.07796	0.00064883	yes
Maff	chr15:79346620-79359076	1.16313	4.88013	2.0689	0.00064883	yes
Cxcl10	chr5:92331840-92414627	1.13272	4.69578	2.05157	0.00064883	yes
Nfkbiz	chr16:55811376-55838641	2.96872	12.2837	2.04884	0.00064883	yes
Zfp36	chr7:28376783-28379228	12.2713	49.8615	2.02263	0.00064883	yes
Ifi44	chr3:151730922-151749959	0.485829	1.91622	1.97974	0.00064883	yes
Ccr1	chr9:123962125-123968692	0.445495	1.73	1.95729	0.00064883	yes
Dusp1	chr17:26505590-26508472	3.44967	13.2328	1.93959	0.00064883	yes
Egr3	chr14:70077444-70082613	0.248771	0.943007	1.92245	0.00064883	yes
Tnc	chr4:63959784-64047015	0.144531	0.537289	1.89432	0.00064883	yes
Ccl2	chr11:82035576-82037452	24.0661	89.0437	1.88751	0.00064883	yes
AI607873	chr1:173723427-173741809	0.949841	3.4423	1.85762	0.00064883	yes
Isg15	chr4:156199423-156200818	0.256824	0.923337	1.84608	0.0422828	yes
Lars2	chr9:123366939-123462664	473.274	1696.81	1.84208	0.0188838	yes
Hbegf	chr18:36504926-36515805	0.17377	0.606561	1.80347	0.00419912	yes
Rgs16	chr1:153740352-153745468	9.10458	31.1866	1.77626	0.00064883	yes
C030037D09Rik	chr11:88718642-88728572	0.310329	1.04717	1.75463	0.017885	yes
Gpr35	chr1:92973118-92986391	0.304015	1.01289	1.73627	0.00064883	yes
Jun	chr4:95049035-95052222	34.6437	114.077	1.71934	0.00064883	yes
Creb5	chr6:53573373-53695832	0.18422	0.599473	1.70227	0.00064883	yes
Gadd45a	chr6:67035095-67080652	0.701262	2.28099	1.70163	0.00064883	yes
Il1b	chr2:129364579-129375733	0.715388	2.29378	1.68093	0.00064883	yes
1700012D01Rik	chr10:127667122-127668851	2.26548	7.11427	1.6509	0.00064883	yes
3300002P13Rik	chr7:3421664-3423445	0.611797	1.89432	1.63056	0.0146074	yes
Ifi202b	chr1:173962568-173982844	0.980237	3.01632	1.62159	0.00064883	yes
Ly6g5b	chr17:35113945-35115400	0.269013	0.824832	1.61642	0.0486014	yes
Thbs1	chr2:118111921-118127133	0.212085	0.645203	1.60511	0.00064883	yes
Il1rn	chr2:24336859-24351491	2.76881	8.33106	1.58924	0.00064883	yes
Slfn5	chr11:82911252-82964850	0.394215	1.1718	1.57167	0.00064883	yes
Rgs1	chr1:144244668-144249104	20.9433	62.0154	1.56614	0.00064883	yes
Bcar1	chr8:111710474-111743849	0.350759	1.03858	1.56606	0.00064883	yes
H2-T24	chr17:35994503-36038174	1.36352	4.00162	1.55325	0.00257544	yes
Cacnb2	chr2:14604305-14987908	0.189971	0.55744	1.55304	0.00064883	yes
Cd80	chr16:38458932-38486932	1.38599	4.05899	1.5502	0.00064883	yes
Naalad2	chr9:18323020-18385928	0.188859	0.550818	1.54427	0.00602054	yes
Ptgs2	chr1:150100123-150108012	4.63193	13.3348	1.52551	0.00064883	yes
Cd40	chr2:165055635-165071654	1.17663	3.37372	1.51968	0.00064883	yes
Usp18	chr6:121245905-121270917	0.736358	2.10245	1.51359	0.00064883	yes
Ddx58	chr4:40203776-40239825	1.43198	4.08717	1.51309	0.00064883	yes
Lilr4b	chr10:51480611-51486329	7.1719	20.1103	1.48751	0.00064883	yes
Ghr	chr15:3317754-3583352	0.187713	0.5194	1.46832	0.00301495	yes
Bcl3	chr7:19808461-19822755	2.24463	6.12815	1.44897	0.00064883	yes
Gm4262	chr16:11008897-11015184	0.322793	0.879629	1.44628	0.00257544	yes

B230217O12Rik	chr19:57323196-57360899	0.942287	2.55122	1.43695	0.00064883	yes
Fas	chr19:34290658-34327770	2.60331	6.97174	1.42117	0.00064883	yes
Gbp2	chr3:142620662-142638008	0.193801	0.514108	1.4075	0.010626	yes
Mmp12	chr9:7347373-7360461	2.06022	5.46163	1.40653	0.00064883	yes
Scimp	chr11:70790931-70812561	0.372851	0.975308	1.38726	0.0202883	yes
Irf7	chr7:141263182-141266424	0.866924	2.25947	1.38201	0.00064883	yes
Tmem44	chr16:30511854-30550578	0.217513	0.565867	1.37936	0.00064883	yes
Bicd1	chr6:149408983-149563326	0.215858	0.560909	1.37768	0.00064883	yes
Spred3	chr7:29158828-29168647	0.231405	0.596207	1.3654	0.00064883	yes
2210417A02Rik	chr5:148741839-148743139	5.21485	13.4328	1.36506	0.00064883	yes
Clec4n	chr6:123229842-123247024	0.875581	2.24412	1.35784	0.00064883	yes
Wnt1	chr15:98789856-98793830	0.229371	0.581129	1.34117	0.0259255	yes
Gpr84	chr15:103308234-103310438	12.1636	30.7689	1.3389	0.00064883	yes
Gapt	chr13:110352614-110357172	0.421942	1.06402	1.33441	0.00064883	yes
1300002E11Rik	chr16:21794346-21809039	6.34206	15.9779	1.33306	0.00217763	yes
Bzrap1	chr11:87760540-87785928	0.416776	1.04635	1.32802	0.00064883	yes
Zbtb10	chr3:9250566-9285332	0.451277	1.13267	1.32764	0.00064883	yes
Sbspon	chr1:15853861-15892722	0.356915	0.878855	1.30005	0.0034365	yes
Lilrb4a	chr10:51490897-51496611	52.7317	129.329	1.2943	0.00064883	yes
Slfn10-ps	chr11:83028125-83040533	3.54216	8.68313	1.29358	0.00064883	yes
Tbx20	chr9:24720811-24774303	0.333044	0.815016	1.29112	0.00064883	yes
Rnu12	chr15:83149644-83149794	105.768	258.61	1.28987	0.00064883	yes
Gm10782	chr13:56362899-56368857	0.398095	0.971815	1.28757	0.0220255	yes
S100a11	chr3:93520495-93526288	109.251	265.797	1.28268	0.00064883	yes
Mmp9	chr2:164948218-164955849	14.5344	35.073	1.27088	0.00064883	yes
Pla2g4c	chr7:13325666-13360668	0.439014	1.05849	1.26967	0.00064883	yes
Itgax	chr7:128129567-128150657	0.43376	1.03789	1.25869	0.00064883	yes
Gp1bb,Sept5	chr16:18620318-18629938	0.435047	1.03817	1.2548	0.00064883	yes
Gm6377	chrX:109196755-109200445	1.72841	4.11068	1.24993	0.00064883	yes
C3	chr17:57203966-57228136	1.35938	3.2301	1.24863	0.00064883	yes
Trib1	chr15:59648653-59657099	2.69302	6.37759	1.24379	0.00064883	yes
Kctd1	chr18:14968684-15151446	0.225661	0.531958	1.23716	0.00064883	yes
Ccl4	chr11:83662583-83664683	10.9687	25.7319	1.23016	0.00064883	yes
Igfals	chr17:24878769-24882008	0.445427	1.03848	1.22121	0.00797055	yes
Gimap6	chr6:48701582-48708244	0.394901	0.919259	1.21898	0.00419912	yes
Nr1d1	chr11:98767931-98775377	0.901106	2.09431	1.2167	0.00064883	yes
Cald1	chr6:34709443-34775469	0.453243	1.05001	1.21205	0.00064883	yes
Atf3	chr1:191170296-191183333	10.2738	23.592	1.19933	0.00064883	yes
Rgs8	chr1:153653036-153697665	1.19055	2.73079	1.19769	0.00064883	yes
Marcks	chr10:37133242-37138926	1.40769	3.22626	1.19653	0.00064883	yes
Tal2	chr4:53779704-53786885	0.319988	0.732757	1.19532	0.0496379	yes
Fgr	chr4:132974094-133001882	4.412	10.0555	1.18848	0.00064883	yes
Creb3l2	chr6:37331020-37442148	1.34216	3.04483	1.18181	0.00064883	yes
C3ar1	chr6:122847139-122856157	7.84492	17.747	1.17774	0.00064883	yes
Six4	chr12:73100258-73113245	0.253239	0.570931	1.17282	0.00168106	yes
Marcksl1	chr4:129513580-129515981	32.9413	73.9511	1.16667	0.00064883	yes
Cpeb4	chr11:31870939-31935635	5.93951	13.3163	1.16478	0.00064883	yes
Cd69	chr6:129267324-129275369	0.339866	0.761825	1.16449	0.0211532	yes
Jazf1	chr6:52768067-53068624	0.603405	1.35228	1.16419	0.00118536	yes
Myo1d	chr11:80482126-80780025	2.05688	4.58065	1.15509	0.00064883	yes
Hectd2	chr19:36554638-36689479	0.403283	0.893628	1.14788	0.01225	yes
Cpm	chr10:117629499-117687352	0.320804	0.709356	1.14481	0.00064883	yes
AA388235	chr17:33981491-33985358	0.233967	0.516821	1.14336	0.00858867	yes
Saa3	chr7:46711997-46715676	14.7879	32.6439	1.14239	0.00064883	yes
Ier3	chr17:35821712-35822911	24.6002	53.8998	1.13161	0.00064883	yes
Stx11	chr10:12939982-12964259	1.02826	2.25069	1.13016	0.00064883	yes
Ehd2	chr7:15948986-15967535	1.54141	3.36787	1.12758	0.00064883	yes
Gnat3	chr5:17962569-18019668	0.455963	0.991142	1.12018	0.0207355	yes
Aqp9	chr9:71110658-71163289	0.268901	0.583804	1.11841	0.0164013	yes
Cyp4f37	chr17:32621318-32636184	0.534317	1.1576	1.11537	0.00419912	yes
Abca1	chr4:53030788-53159895	1.32379	2.85936	1.11102	0.00064883	yes
Hnrnpd	chr5:99955934-99978938	21.4029	46.1773	1.10938	0.00064883	yes
Spink5	chr18:43963240-44022487	10.2458	21.9972	1.10228	0.00064883	yes
Irf9	chr14:55603984-55610030	4.94676	10.5831	1.09721	0.00064883	yes
Slc15a3	chr19:10842543-10869779	3.32943	7.09076	1.09066	0.00064883	yes

Pde4b	chr4:102254741-102607262	2.96904	6.31904	1.08971	0.00064883	yes
Cacnb1	chr11:98001507-98022627	0.444758	0.944936	1.0872	0.00797055	yes
Cgnl1	chr9:71626506-71771602	0.44569	0.946292	1.08624	0.00064883	yes
6820431F20Rik	chr8:20268285-20297432	0.608425	1.28424	1.07777	0.00064883	yes
Srgn	chr10:62494427-62507755	94.527	198.729	1.072	0.00064883	yes
Slc7a2	chr8:40862366-40922070	0.900081	1.88819	1.06887	0.00064883	yes
Btg2	chr1:134074864-134079155	7.1741	15.0081	1.06487	0.00064883	yes
Gadd45b	chr10:80930090-80932204	5.09261	10.6506	1.06446	0.00064883	yes
Aoah	chr13:20794112-21024254	0.940163	1.96501	1.06356	0.00118536	yes
Ccl3	chr11:83647842-83649378	70.3127	146.656	1.06058	0.00064883	yes
Kcnab1	chr3:65109367-65378225	0.789041	1.64573	1.06055	0.00168106	yes
Lgals9	chr11:78962978-78984924	0.66556	1.38477	1.057	0.00666366	yes
Rnu11	chr4:132270078-132270186	183.891	381.889	1.0543	0.024169	yes
Prkcg	chr7:3303531-3331097	0.481439	0.999449	1.05378	0.00168106	yes
Ifih1	chr2:62595792-62646255	0.767053	1.59135	1.05285	0.00064883	yes
Cd44	chr2:102811141-102901665	58.01	120.281	1.05203	0.00064883	yes
Hivep3	chr4:119814677-120135411	0.544396	1.128	1.05104	0.00064883	yes
Serpine1	chr5:137061505-137072272	6.77238	13.9746	1.04508	0.00064883	yes
Slfn8	chr11:83002157-83020810	3.85369	7.94981	1.04468	0.00064883	yes
Lims2	chr18:31931506-31958619	1.09063	2.24526	1.04172	0.0049451	yes
Oas3	chr5:120753097-120777659	1.99669	4.10885	1.04112	0.00064883	yes
Mmp13	chr9:7272513-7283333	0.371455	0.762977	1.03845	0.00947319	yes
Olfr934	chr9:38982109-38983042	1.58804	3.2431	1.03013	0.0296658	yes
Tanc2	chr11:105589985-105929303	0.669692	1.36592	1.02831	0.00064883	yes
Ikbke	chr1:131254601-131279563	4.61117	9.39811	1.02724	0.00064883	yes
Nfkbie	chr17:45555699-45563168	3.35543	6.80376	1.01983	0.00064883	yes
Trem1	chr17:48232738-48246924	0.900721	1.82242	1.01671	0.00064883	yes
Pde3b	chr7:114415253-114537937	4.69424	9.4818	1.01427	0.00064883	yes
Procr	chr2:155751216-155755478	1.78513	3.60523	1.01406	0.00118536	yes
Atg2a	chr19:6241667-6262304	1.88302	3.80211	1.01375	0.00064883	yes
Rassf8	chr6:145808382-145817584	9.10454	18.3552	1.01153	0.00064883	yes
Nrn1	chr13:36725621-36734477	0.415493	0.836264	1.00913	0.0499321	yes
Kazald1	chr19:45076138-45079289	0.677548	1.35572	1.00067	0.0412463	yes
Zyx	chr6:42349827-42380558	4.05522	8.1037	0.9988	0.00064883	yes
Efr3b	chr12:3962553-4038915	2.15245	4.28551	0.993486	0.00064883	yes
Lin28b	chr10:45376618-45470201	0.939232	1.86845	0.99229	0.00064883	yes
Adgre4	chr17:55749983-55853662	0.79966	1.58969	0.991289	0.00064883	yes
Nr4a2	chr2:57107225-57124003	1.14582	2.27157	0.987314	0.00064883	yes
Gpr1	chr1:63182570-63314575	2.14501	4.2427	0.984	0.00118536	yes
Sec24a	chr11:51692262-51756834	4.923	9.72715	0.982478	0.00064883	yes
Cdk6	chr5:3344311-3522225	21.2205	41.8969	0.981384	0.00064883	yes
Eya4	chr10:23104167-23349903	0.296705	0.585103	0.979664	0.00886675	yes
Taf9b	chrX:106206873-106221158	0.658138	1.29508	0.976579	0.00382257	yes
Nr4a1	chr15:101266845-101274794	1.31623	2.588	0.975427	0.00118536	yes
Zfp36l1	chr12:80107759-80113013	2.9236	5.74474	0.974499	0.00064883	yes
Fus	chr7:127967478-127982031	11.1307	21.8673	0.974233	0.00064883	yes
Neat1	chr19:5824709-5845480	23.0368	45.1936	0.972176	0.00064883	yes
Spry1	chr3:37639946-37644599	0.359553	0.70424	0.969864	0.0235697	yes
Tlr9	chr9:106222597-106226876	1.17796	2.30115	0.966066	0.00168106	yes
Vamp2	chr11:69088527-69092381	1.0767	2.10285	0.965726	0.00064883	yes
Rpl21	chr5:146832889-146837032	17.0916	33.3181	0.963016	0.00064883	yes
Cecr2	chr6:120666420-120771191	0.515733	1.00523	0.962825	0.00064883	yes
Gadd45g	chr13:51846674-51848474	7.10826	13.8218	0.959372	0.00064883	yes
H2-Ab1	chr17:34263226-34269418	0.790618	1.53729	0.959338	0.0218243	yes
Ier5	chr1:155096366-155099636	17.7293	34.4401	0.957949	0.00064883	yes
Gbp7	chr3:142530335-142550151	0.395865	0.765303	0.951023	0.0034365	yes
Olfr99	chr17:37279500-37280418	3.1934	6.16146	0.94818	0.0049451	yes
A630089N07Rik	chr16:98062511-98082439	5.23609	10.0987	0.947611	0.00064883	yes
Irgm2	chr11:58214976-58222783	0.706076	1.35993	0.945641	0.00064883	yes
Xcr1	chr9:123852314-123862029	0.294495	0.563769	0.936858	0.013835	yes
Btbd19	chr4:117119217-117125725	9.48408	18.131	0.934877	0.00064883	yes
Ass1	chr2:31470269-31520670	0.633942	1.20849	0.930782	0.01225	yes
Clec4e	chr6:123281788-123289871	59.9536	113.977	0.926828	0.00064883	yes
Fzd5	chr1:64730557-64737750	1.0879	2.06564	0.925046	0.00064883	yes
Ppp1r10	chr17:35917195-35932283	12.3466	23.4177	0.923481	0.00064883	yes

Flt1	chr5:147562195-147725988	1.27445	2.41379	0.921424	0.00064883	yes
Dhx58	chr11:100694883-100704271	1.00931	1.90979	0.920047	0.00168106	yes
Kpna2	chr11:106988628-106999525	3.5121	6.63825	0.918468	0.00064883	yes
P2ry2	chr7:100996567-101012053	1.81012	3.41482	0.915722	0.00064883	yes
Hivep1	chr13:42052020-42185026	2.02376	3.8167	0.915289	0.00064883	yes
Anpep	chr7:79821802-79842352	1.993	3.74367	0.909511	0.00064883	yes
Bcl2a1b	chr9:89199272-89207838	27.7751	52.1304	0.908331	0.00064883	yes
Flrt2	chr12:95692225-95785213	9.09701	17.0594	0.907099	0.00064883	yes
Apoo	chrX:94367109-94417092	4.94841	9.27886	0.906983	0.00257544	yes
Hoxa9	chr6:52223096-52227370	1.75442	3.28883	0.90658	0.00118536	yes
Mcemp1	chr8:3665761-3668905	0.665773	1.24505	0.903106	0.0254961	yes
Tacc2	chr7:130577483-130764784	2.20644	4.11942	0.900716	0.00064883	yes
Chn1	chr2:73596525-73775346	0.539425	1.0066	0.899999	0.00301495	yes
Per1	chr11:69098955-69109957	1.3168	2.44924	0.895304	0.00064883	yes
Ppig	chr2:69723087-69754059	30.3097	56.1754	0.89016	0.00064883	yes
C5ar1	chr7:16246742-16259540	3.62829	6.72327	0.889874	0.00064883	yes
Chst15	chr7:132236254-132318291	0.72505	1.3405	0.886616	0.00168106	yes
Chst11	chr10:82985496-83195891	0.382801	0.705868	0.882805	0.00382257	yes
Plk3	chr4:117126812-117133952	3.39452	6.25619	0.882077	0.00064883	yes
Dusp16	chr6:134715467-134792628	3.5434	6.52834	0.881582	0.00064883	yes
Lamc1	chr1:153218921-153332786	11.9428	22.0033	0.881578	0.00064883	yes
Pnrc1	chr4:33245422-33248787	18.0561	33.2564	0.881143	0.00064883	yes
Ahr	chr12:35497978-35534989	0.279158	0.514151	0.88111	0.0171867	yes
Tlr8	chrX:167242731-167263788	0.409513	0.75395	0.880561	0.0197691	yes
Ccdc122	chr14:77036771-77112204	2.82996	5.21019	0.880554	0.00064883	yes
Bcl2l11	chr2:128126037-128162547	4.32967	7.96501	0.879418	0.00064883	yes
Nova1	chr12:46694516-46818775	0.89754	1.65068	0.879014	0.00118536	yes
Pmepa1	chr2:173224464-173276533	7.426	13.6523	0.878489	0.00064883	yes
Bhlhe40	chr6:108577035-108666925	11.4748	21.0881	0.877954	0.00064883	yes
Csf2ra	chr19:61224401-61228418	21.8678	40.1285	0.875818	0.00064883	yes
Tmem223	chr19:8770995-8772475	3.95344	7.25376	0.87562	0.010626	yes
Diap2	chrX:129749741-130465833	3.57241	6.5454	0.873584	0.00064883	yes
Gm15446	chr5:109933562-109941710	0.805985	1.47498	0.871869	0.0218243	yes
Foxo1	chr3:52268336-52350109	0.876669	1.60397	0.871542	0.00064883	yes
1500015A07Rik	chr18:61726389-61728253	3.13336	5.72928	0.870645	0.00118536	yes
Enah	chr1:181896385-182019980	2.09983	3.83472	0.868847	0.00064883	yes
Osm	chr11:4236784-4241026	1.22361	2.23414	0.868577	0.0049451	yes
Rhbdf2	chr11:116598165-116627019	5.02139	9.14454	0.864824	0.00064883	yes
Dnajc28	chr16:91614256-91618999	0.298941	0.543786	0.863179	0.0245865	yes
Ptger4	chr15:5233398-5244187	5.04351	9.17312	0.862985	0.00064883	yes
Fcgr2b	chr1:170960558-170976071	11.1484	20.1859	0.856517	0.00064883	yes
Hexim1	chr11:103116324-103119724	16.6881	30.2045	0.855948	0.00064883	yes
Gpr65	chr12:98268634-98276722	16.3781	29.585	0.853095	0.00064883	yes
Fam49a	chr12:12262138-12392475	6.62337	11.9521	0.851628	0.00064883	yes
Ifrd1	chr12:40203128-40223189	123.79	223.383	0.851623	0.00064883	yes
Ssbp1	chr6:40471414-40481823	64.4152	116.206	0.851215	0.00382257	yes
Hlx	chr1:184727144-184732493	1.08905	1.96365	0.850473	0.00829754	yes
Atxn2	chr5:121711608-121814950	5.90951	10.6504	0.849792	0.00064883	yes
Myh10	chr11:68691914-68816624	0.445536	0.802216	0.848448	0.00064883	yes
9430037G07Rik	chr9:88595324-88599243	0.538259	0.968838	0.847956	0.0259255	yes
Soat2	chr15:102150574-102163436	0.643208	1.15728	0.847379	0.0222603	yes
Myadm	chr7:3289037-3299349	20.8132	37.4394	0.847059	0.00064883	yes
Cbll1	chr12:31484828-31499616	4.36387	7.84616	0.846378	0.00064883	yes
Birc3	chr9:7848700-7873170	10.3865	18.6717	0.846151	0.00064883	yes
Nfatc1	chr18:80606204-80713071	30.2838	54.3122	0.842731	0.00064883	yes
Nfia	chr4:97567874-98118876	0.612985	1.09931	0.842674	0.00118536	yes
Igsf6	chr7:121034444-121076835	55.2087	98.995	0.842461	0.0146074	yes
Met	chr6:17463956-17573980	2.66557	4.77894	0.842248	0.00064883	yes
Hcar2	chr5:123863569-123865516	0.698044	1.25147	0.842231	0.0218243	yes
Slc7a11	chr3:50364935-50443613	3.94309	7.06394	0.841145	0.00064883	yes
Rai14	chr15:10568977-10714631	34.2471	61.3002	0.83991	0.00064883	yes
Ubap2	chr4:41194314-41275135	11.505	20.5661	0.838007	0.00064883	yes
Elovl4	chr9:83778691-83806305	1.59127	2.84358	0.83753	0.00217763	yes
Phactr4	chr4:132355924-132422446	1.97911	3.52601	0.833184	0.00118536	yes
Prrc2c	chr1:162671784-162740556	28.7461	51.1854	0.832368	0.00064883	yes

Hmga2	chr10:120361274-120476935	14.2506	25.343	0.830559	0.00064883	yes
Hipk2	chr6:38697839-38876190	8.72075	15.5	0.829745	0.00064883	yes
Helz2	chr2:181227614-181242027	1.73016	3.0724	0.828459	0.00064883	yes
Bcl2a1d	chr9:88723287-88731850	15.9397	28.3039	0.828378	0.00064883	yes
Zc3hav1l	chr6:38287393-38299259	1.18737	2.10747	0.827742	0.00118536	yes
Sep-03	chr15:82274934-82294442	0.391292	0.694268	0.827248	0.0103347	yes
Actg1	chr11:120345689-120348484	41.2285	73.151	0.827234	0.00064883	yes
Itga8	chr2:12106659-12312315	0.856287	1.51922	0.827168	0.00118536	yes
Dpys	chr15:39768484-39857470	1.00118	1.77292	0.824421	0.00568302	yes
Safb2	chr17:56562941-56584583	14.5392	25.7428	0.82422	0.00064883	yes
Gm9958	chr5:90366996-90368488	1.68278	2.97556	0.822315	0.0188838	yes
Bag3	chr7:128523582-128546979	7.29339	12.8951	0.822161	0.00064883	yes
Ece1	chr4:137862236-137965229	0.724534	1.27944	0.820383	0.00257544	yes
Zic5	chr14:122459159-122465658	0.780059	1.375	0.817773	0.0166553	yes
Atn1	chr6:124742543-124756487	0.899392	1.57964	0.812573	0.00301495	yes
BC052040	chr2:115581715-115778768	2.32251	4.07691	0.81179	0.00118536	yes
Patl1	chr19:11912398-11945096	6.75603	11.8456	0.810098	0.00064883	yes
Mns1	chr9:72438528-72491959	4.17721	7.32282	0.80986	0.0237709	yes
Tmem26	chr10:68723745-68782654	2.16251	3.79061	0.809723	0.00064883	yes
BC048403	chr10:121739936-121752859	1.75794	3.07816	0.808185	0.00118536	yes
Smndc1	chr19:53379213-53390573	22.6284	39.6216	0.808154	0.00064883	yes
Cd36	chr5:17781689-17888959	36.4315	63.7309	0.806805	0.00064883	yes
Abca5	chr11:110269368-110337716	0.386429	0.675605	0.805977	0.0100593	yes
Dnajb9	chr12:44205896-44210068	9.70801	16.9606	0.804941	0.00064883	yes
Acta2	chr19:34240335-34255373	0.779517	1.36183	0.804891	0.0161452	yes
Slamf8	chr1:172581376-172590568	2.09925	3.66305	0.803168	0.00064883	yes
Neto2	chr8:85636587-85691009	0.577786	1.008	0.802882	0.00797055	yes
Casp4	chr9:5308848-5336791	2.43938	4.25236	0.801751	0.00217763	yes
Jpx	chrX:103493557-103506425	0.436892	0.760681	0.800016	0.0470882	yes
Fmn1	chr2:113220186-113716767	8.79359	15.303	0.79929	0.00064883	yes
Fmnl2	chr2:52857867-53134202	1.63784	2.84964	0.798988	0.00064883	yes
Ehd1	chr19:6276895-6300096	29.3422	50.9949	0.797376	0.00064883	yes
Med15	chr16:17651207-17722947	10.1272	17.5764	0.795405	0.00064883	yes
Yes1	chr5:32611170-32687066	0.728971	1.26411	0.794186	0.0111526	yes
Cd9	chr6:125460265-125494755	725.459	1257.78	0.793908	0.00168106	yes
Dgkh	chr14:78569608-78725089	0.75922	1.31618	0.793762	0.00568302	yes
Sap130	chr18:31634382-31723061	5.48808	9.49443	0.790781	0.00118536	yes
Ptpn23	chr9:110385088-110408210	1.42421	2.46374	0.790686	0.00168106	yes
Fam57a	chr11:76202055-76208257	8.80161	15.225	0.790601	0.00454713	yes
Meis1	chr11:18880427-19018969	1.70391	2.94453	0.789193	0.00301495	yes
Brd4	chr17:32196271-32284133	3.93605	6.79494	0.787713	0.00419912	yes
Tmem171	chr13:98686237-98694831	4.38602	7.57126	0.787622	0.0034365	yes
Rnf111	chr9:70425428-70503725	12.3934	21.3797	0.786665	0.00064883	yes
Dnajb4	chr3:152183870-152210083	6.3447	10.9443	0.78655	0.00064883	yes
Parp8	chr13:116854823-117025516	2.88666	4.9782	0.786221	0.00064883	yes
Kctd11	chr11:69878263-69880985	1.14663	1.97733	0.786156	0.00568302	yes
Kctd12	chr14:102976580-102982637	6.40743	11.0438	0.785424	0.00064883	yes
Fos	chr12:85473900-85477270	17.1087	29.4221	0.782172	0.00064883	yes
Braf	chr6:39603236-39725463	5.01997	8.62313	0.780532	0.00064883	yes
Aff1	chr5:103754161-103855322	2.98608	5.12919	0.780478	0.00064883	yes
Nes	chr3:87971092-87980451	1.21486	2.08357	0.778268	0.00064883	yes
Nol4l	chr2:153407460-153529971	1.36061	2.33243	0.777584	0.00064883	yes
Gja1	chr10:56377299-56390419	18.289	31.3355	0.776821	0.00064883	yes
Ppp1r13b	chr12:111828457-111908055	2.51058	4.30018	0.776376	0.00064883	yes
Apbb1ip	chr2:22774326-22875653	8.86559	15.1817	0.776045	0.00064883	yes
Ctnnd2	chr15:30172592-31029343	0.350386	0.599579	0.775007	0.0176712	yes
Proser3	chr7:30539133-30552272	0.594607	1.01747	0.774983	0.0248059	yes
Meis2	chr2:115861263-116065058	1.78031	3.04532	0.774465	0.00168106	yes
Tnip1	chr11:54910786-54962940	17.3256	29.6033	0.772858	0.00064883	yes
L3mbtl3	chr10:26275451-26375185	1.03392	1.7665	0.772763	0.00419912	yes
Il2rg	chrX:101264384-101268255	16.5001	28.1598	0.77116	0.00064883	yes
Atxn1	chr13:45549755-45964991	2.13319	3.63263	0.768003	0.00064883	yes
R3hdm2	chr10:127390310-127499384	4.66438	7.93835	0.767155	0.00064883	yes
Casc4	chr2:121866969-121936207	1.37754	2.34374	0.766717	0.00382257	yes
Sulf2	chr2:166073898-166155683	3.60725	6.13472	0.766097	0.00064883	yes

Slc35d1	chr4:103171717-103214884	4.21288	7.16233	0.765621	0.00168106	yes
Plxna3	chrX:74329065-74344689	0.280976	0.477141	0.763971	0.0220255	yes
Wiz	chr17:32354049-32389439	1.76716	2.99213	0.759742	0.00064883	yes
Samd9l	chr6:3372257-3399571	9.37571	15.8486	0.757358	0.00064883	yes
Crtc2	chr3:90254280-90264125	2.53069	4.27512	0.756434	0.00118536	yes
Zfand3	chr17:30005086-30210020	15.5364	26.2168	0.754841	0.00064883	yes
9430020K01Rik	chr18:4634928-4682869	0.589301	0.994323	0.754709	0.00531709	yes
Rnf44	chr13:54679398-54693960	4.54645	7.66661	0.753851	0.00118536	yes
Arhgap6	chrX:168795098-169304440	0.33891	0.57139	0.753574	0.0261255	yes
Mapk1ip1l	chr14:47298313-47323091	32.9118	55.4604	0.752852	0.00217763	yes
Lcor	chr19:41549638-41559781	4.42047	7.42673	0.748526	0.00064883	yes
Adm	chr7:110627668-110629819	2.30475	3.86908	0.747383	0.00886675	yes
Cnot3	chr7:3645268-3677553	4.80145	8.058	0.746951	0.0224583	yes
Abtb2	chr2:103566309-103718423	0.834629	1.398	0.744156	0.00977864	yes
4930526I15Rik	chr9:124423250-124424856	8.41289	14.0891	0.743904	0.00382257	yes
Prkcb	chr7:122289124-122634401	1.69455	2.83549	0.742701	0.00064883	yes
Stx16	chr2:174077050-174099771	21.8108	36.49	0.742454	0.00064883	yes
Pilra	chr5:137787801-137836278	2.38411	3.98666	0.74173	0.0296658	yes
Itga5	chr15:103344285-103366748	7.2429	12.0904	0.739225	0.00064883	yes
Ube2k	chr5:65537260-65598989	26.5448	44.3081	0.739142	0.00064883	yes
Pabpc1	chr15:36595657-36608973	209.449	349.544	0.738876	0.00118536	yes
Eif5a2	chr3:28781310-28798846	1.73651	2.89459	0.737166	0.00454713	yes
Ccl9	chr11:83572916-83578636	205.861	342.298	0.733581	0.00064883	yes
Elk4	chr1:132007604-132025684	12.36	20.5492	0.733411	0.00064883	yes
Wac	chr18:7868831-7929028	32.0083	53.1421	0.73141	0.00118536	yes
Ttpa	chr4:20008427-20030785	0.796199	1.32167	0.731159	0.0340017	yes
4921531C22Rik	chr2:179976852-179979013	0.988137	1.63984	0.73077	0.018127	yes
Parp14	chr16:35832877-35871382	2.39843	3.97919	0.730387	0.00064883	yes
Tpt1	chr14:75845255-75848303	93.849	155.692	0.730286	0.00064883	yes
Megf9	chr4:70431926-70534928	23.0334	38.2111	0.730268	0.00064883	yes
Ptprk	chr10:28074819-28597397	0.999183	1.6566	0.729407	0.00419912	yes
Gm13139	chr4:145781542-145831978	0.949261	1.57375	0.729334	0.00947319	yes
E330033B04Rik	chr15:96268563-96275275	0.656869	1.08897	0.729281	0.00602054	yes
Traf1	chr2:34943257-34961772	3.08193	5.10823	0.72899	0.00301495	yes
5830417I10Rik	chr3:88777056-88832487	0.745866	1.23611	0.728817	0.0034365	yes
4833427F10Rik	chr17:35772449-35780687	1.01639	1.68294	0.727523	0.0478434	yes
Tab2	chr10:7905647-7956123	30.4979	50.491	0.727317	0.00064883	yes
Il1rap	chr16:26581704-26755502	7.74565	12.8228	0.727258	0.00064883	yes
Plekhn1	chr4:156221455-156228542	0.820035	1.35754	0.727233	0.0231387	yes
Proser1	chr3:53463816-53481755	0.87703	1.45091	0.726258	0.0111526	yes
Smcr8	chr11:60777524-60788287	14.8531	24.5655	0.725873	0.00064883	yes
Ifi204	chr1:173747293-173766919	1.09175	1.80447	0.72493	0.0116752	yes
Rabgap1l	chr1:160219173-160792938	0.686755	1.13491	0.724706	0.0183466	yes
Fkbp1a	chr2:151542482-151561691	89.5541	147.825	0.723061	0.00064883	yes
Ssbp2	chr13:91461096-91786148	0.914034	1.50828	0.722587	0.00886675	yes
Lrrc32	chr7:98494221-98501830	0.522906	0.862699	0.722307	0.0226785	yes
Atp6v0a1	chr11:101009451-101063717	5.18704	8.54294	0.719821	0.00168106	yes
Padi2	chr4:140906359-140957902	1.21649	2.00349	0.719787	0.00454713	yes
Trim13	chr14:61598225-61682373	5.30092	8.7264	0.719144	0.0347854	yes
Rnf225	chr7:12927415-12931072	0.799356	1.31466	0.717778	0.0183466	yes
Fn1	chr1:71585472-71653234	2.48044	4.07934	0.717741	0.00118536	yes
Nfkb2	chr19:46304736-46327156	9.30668	15.3048	0.717649	0.00217763	yes
Srcap,Tmem265	chr7:127510437-127565270	4.72052	7.76206	0.717493	0.00419912	yes
Atxn7l1	chr12:33302514-33394760	2.68891	4.42105	0.717365	0.0116752	yes
Hspa8	chr9:40801272-40805199	312.528	513.421	0.716156	0.00568302	yes
Ncl	chr1:86344718-86359455	285.71	469.089	0.715313	0.0205128	yes
Nab1	chr1:52455848-52500448	35.5843	58.4107	0.714994	0.00064883	yes
Scaf4	chr16:90229143-90284425	6.00082	9.84595	0.71437	0.00064883	yes
Rnd3	chr2:51130438-51149111	4.44013	7.27846	0.713033	0.00257544	yes
Zswim5	chr4:116877401-116989105	0.291793	0.477862	0.711648	0.0481279	yes
Hmgn2	chr4:133964738-133967991	21.0949	34.5381	0.711293	0.00064883	yes
Agpat9	chr5:100846228-100899102	0.671926	1.09968	0.710708	0.0355411	yes
2210018M11Rik	chr7:98590605-98656569	7.50444	12.2771	0.710157	0.00118536	yes
Tet2	chr3:133463676-133544390	6.60952	10.812	0.710015	0.00064883	yes
Zc3h12c	chr9:52111984-52168111	9.71249	15.8788	0.70919	0.00064883	yes

Btf3l4	chr4:108814294-108833584	3.59119	5.86459	0.707569	0.00454713	yes
Srrm1	chr4:135320483-135353214	18.1442	29.5956	0.705875	0.00064883	yes
Kctd21	chr7:97332322-97350216	0.775851	1.26385	0.703969	0.0207355	yes
Sh3kbp1	chrX:159627271-159975920	21.2663	34.6101	0.702629	0.00064883	yes
Stap1	chr5:86071827-86103993	40.1914	65.3667	0.701669	0.00064883	yes
Sowahc	chr10:59221921-59226433	7.11948	11.5783	0.701581	0.00064883	yes
Dmtf1	chr5:9100736-9161776	24.8295	40.3656	0.701067	0.00301495	yes
Ell2	chr13:75707483-75772358	35.5391	57.7544	0.700522	0.00064883	yes
Osgin1	chr8:119437161-119446256	1.52283	2.47253	0.699237	0.0141124	yes
Sik3	chr9:46012819-46224194	5.24578	8.50751	0.697581	0.00118536	yes
Slfn2	chr11:83065111-83070678	11.4721	18.5963	0.696885	0.00064883	yes
Vma21	chrX:71816079-71824706	24.8416	40.2331	0.695624	0.00064883	yes
Col25a1	chr3:130180844-130599883	0.53863	0.871974	0.69499	0.013835	yes
Med14	chrX:12675370-12761973	15.2181	24.6247	0.694315	0.00118536	yes
Rock1	chr18:10064400-10181792	42.3803	68.5422	0.693598	0.00168106	yes
4632427E13Rik	chr7:92740705-92741459	8.81891	14.2569	0.692985	0.0103347	yes
Cnot4	chr6:35022064-35133737	11.4907	18.5744	0.692852	0.00064883	yes
Phf8	chrX:151520671-151633857	7.98756	12.9051	0.692113	0.00064883	yes
Mex3a	chr3:88532394-88541394	0.587119	0.948437	0.691899	0.0135968	yes
Slc30a1	chr1:191894071-191913247	14.1207	22.8064	0.691631	0.00064883	yes
Mtus1	chr8:40990911-41133726	0.588141	0.949427	0.690895	0.0215983	yes
Gatad2b	chr3:90341653-90358120	5.7901	9.34481	0.690578	0.00301495	yes
Rcan1	chr16:92391950-92466169	33.8753	54.6354	0.689603	0.00064883	yes
Cdk12	chr11:98203304-98253540	14.8854	23.9955	0.688867	0.00064883	yes
Hacd2	chr16:35022420-35109175	2.91604	4.69782	0.68798	0.0034365	yes
Med12	chrX:101274090-101298934	3.30841	5.32857	0.68761	0.00118536	yes
Jag1	chr2:137081450-137116520	2.09874	3.38024	0.687598	0.00118536	yes
Tiam1	chr16:89787110-89974699	9.85083	15.8642	0.687459	0.0103347	yes
Plekho2	chr9:65552576-65580087	14.6314	23.5605	0.687298	0.00064883	yes
Dhrs9	chr2:69380461-69403086	40.4802	65.0714	0.684809	0.00064883	yes
2810474O19Rik	chr6:149309413-149335663	19.7669	31.7698	0.684572	0.00064883	yes
Arl5b	chr2:15055361-15079191	7.27469	11.6744	0.682397	0.00118536	yes
Pcgf2	chr11:97688822-97700497	0.81955	1.31475	0.681883	0.0286094	yes
B3galnt1	chr3:69573918-69598960	2.16314	3.46911	0.681443	0.0183466	yes
Ankrd12	chr17:65967500-66077046	3.62107	5.80462	0.680786	0.00064883	yes
Phf11d	chr14:59347406-59365490	6.08178	9.74685	0.680443	0.00301495	yes
Hyou1	chr9:44379489-44392369	50.8831	81.535	0.680233	0.00064883	yes
Cmip	chr8:117257018-117461505	9.85826	15.7833	0.678992	0.00168106	yes
Ier2	chr8:84661330-84662852	25.3976	40.6484	0.67851	0.00382257	yes
Peak1	chr9:56201128-56418050	9.50235	15.2005	0.677766	0.00064883	yes
Prdm2	chr4:143107390-143212709	4.05577	6.46913	0.673597	0.00064883	yes
Tmem17	chr11:22512282-22519231	1.76976	2.82244	0.673393	0.0418725	yes
Rbms1	chr2:60751952-60963204	41.4582	66.0665	0.672259	0.00064883	yes
Smim3	chr18:60474190-60501983	3.71884	5.92622	0.672258	0.00947319	yes
Mitf	chr6:97807057-98021358	29.9242	47.66	0.671467	0.00064883	yes
Homer1	chr13:93304494-93405129	12.5437	19.9726	0.671057	0.0034365	yes
Ptbp2	chr3:119718741-119783388	14.0339	22.3365	0.670486	0.00217763	yes
Tcf7l2	chr19:55741809-55933655	0.907725	1.44366	0.6694	0.0193303	yes
Thap2	chr10:115369965-115384435	11.2295	17.8491	0.668555	0.00168106	yes
B2m	chr2:122120107-122186189	1610.68	2559.74	0.668326	0.016917	yes
Ptpn22	chr3:103859794-103912252	49.498	78.6432	0.667953	0.00064883	yes
Hist1h2ak	chr13:21753376-21753912	1567.3	2486.26	0.665699	0.00419912	yes
Plau	chr14:20836661-20843388	79.1719	125.575	0.665487	0.00168106	yes
Id1	chr2:152736250-152737410	45.419	72.0265	0.66523	0.00064883	yes
Calr	chr8:84842087-84846931	818.578	1297.91	0.664998	0.0397131	yes
Pcdh7	chr5:57718020-58132240	61.6853	97.7771	0.664569	0.00217763	yes
Magohb	chr6:131284388-131293244	8.99463	14.2571	0.664546	0.01225	yes
Fam222b	chr11:78094672-78157339	2.4579	3.89523	0.66428	0.00531709	yes
Bcorl1	chrX:48341357-48406728	0.359903	0.570341	0.664216	0.0279983	yes
Spty2d1	chr7:46990395-47008414	6.61557	10.4767	0.663242	0.00118536	yes
Gripap1	chrX:7789992-7820567	18.5133	29.3089	0.66278	0.00064883	yes
Tmem158	chr9:123259056-123260789	4.00846	6.3449	0.662548	0.0174052	yes
Maml3	chr3:51687612-52105006	1.70346	2.69521	0.661931	0.00382257	yes
Atp2a2	chr5:122453512-122502225	95.5102	151.088	0.66166	0.00257544	yes
Rprd2	chr3:95759872-95818953	3.71882	5.8814	0.661314	0.00118536	yes

H2-K2	chr17:33974658-33978791	3.32014	5.2488	0.660745	0.0156727	yes
Nlrp1a	chr11:71091196-71144704	0.870269	1.37546	0.660376	0.0156727	yes
Cd276	chr9:58524299-58540940	3.70267	5.85141	0.66022	0.00734768	yes
Hist1h2af	chr13:23533910-23534378	103.465	163.446	0.659672	0.00419912	yes
Crebbp	chr16:4084047-4213404	4.87421	7.69913	0.659528	0.00168106	yes
Dab2	chr15:6299788-6440709	65.4675	103.279	0.657696	0.0034365	yes
Kmt2a	chr9:44803354-44881274	1.91456	3.01893	0.657026	0.00064883	yes
Flnc	chr6:29433152-29461888	0.766224	1.20818	0.656997	0.00858867	yes
Bend6	chr1:33852051-33907621	2.59445	4.08959	0.656526	0.013835	yes
Slc29a3	chr10:60712071-60752782	1.14517	1.80464	0.656142	0.0151535	yes
Ccar1	chr10:62743927-62792368	48.5594	76.4079	0.653972	0.00118536	yes
Xk	chrX:9272783-9313245	0.500844	0.787951	0.653745	0.0424874	yes
Wipf2	chr11:98863597-98905578	1.50063	2.36077	0.653688	0.00666366	yes
Dusp18	chr11:3895239-3901296	0.805666	1.26631	0.652375	0.0235697	yes
Basp1	chr15:25363276-25413764	2.20953	3.47232	0.652158	0.0153915	yes
Ifi203	chr1:173920400-173942672	3.08541	4.84823	0.651996	0.0125053	yes
Phc1	chr6:122317730-122340227	1.74814	2.74676	0.651908	0.0103347	yes
Diap1	chr18:37843600-37935423	9.11389	14.3121	0.651094	0.0034365	yes
Eif5b	chr1:37998009-38129662	20.788	32.577	0.6481	0.0135968	yes
Klf3	chr5:64803522-64830129	1.55361	2.43458	0.648052	0.0233549	yes
Zfp770	chr2:114193460-114201432	9.57701	15.0033	0.647633	0.00217763	yes
Has3	chr8:106870241-106882902	1.60609	2.5157	0.647407	0.00766564	yes
Il1r1	chr1:40225079-40316177	0.715299	1.12017	0.6471	0.0320864	yes
Gnaq	chr19:16132830-16387453	18.0708	28.2947	0.646873	0.00257544	yes
Fem1c	chr18:46504605-46525971	18.7474	29.3239	0.645388	0.00168106	yes
Pxdn	chr12:29938035-30017658	2.19695	3.43519	0.644889	0.00602054	yes
Pmaip1	chr18:66458603-66465558	12.524	19.5811	0.64477	0.00217763	yes
Srp54b	chr12:55230167-55263480	6.53281	10.2088	0.644035	0.00666366	yes
Gm13139,Gm132	chr4:146270384-146469440	0.629582	0.983745	0.643891	0.0286094	yes
Tnf	chr17:35199366-35202007	50.3192	78.5475	0.642455	0.00118536	yes
Atp2b1	chr10:98915151-99026143	51.5353	80.3804	0.641283	0.00382257	yes
Zbtb33	chrX:38189792-38252481	6.15226	9.59434	0.641068	0.00257544	yes
Nufip2	chr11:77686138-77717966	31.4791	49.0769	0.640648	0.0034365	yes
D330050G23Rik	chr2:116900151-116912791	2.29768	3.58067	0.640048	0.0343789	yes
Ttc39aos1	chr4:109402278-109406257	4.57291	7.12608	0.639995	0.0148682	yes
Gspt1	chr16:11203382-11254325	89.5877	139.58	0.639725	0.00829754	yes
Tmtc3	chr10:100443901-100487347	12.8204	19.9727	0.639586	0.00118536	yes
Ywhae	chr11:75732886-75765841	232.424	361.777	0.638339	0.00634725	yes
Tnfrsf22	chr7:143636724-143649638	2.42839	3.77904	0.638019	0.0215983	yes
Fam107b	chr2:3713457-3782134	23.2915	36.2325	0.63748	0.00168106	yes
Hoxc4	chr15:103034394-103036852	2.25228	3.50199	0.636787	0.0202883	yes
Cd59a	chr2:104095800-104115410	2.12988	3.31141	0.636672	0.0224583	yes
Rbm47	chr5:66016548-66151954	5.2419	8.1497	0.636656	0.00382257	yes
Rhob	chr12:8497758-8499985	5.73574	8.91422	0.636128	0.0116752	yes
Eif5	chr12:111538100-111546753	140.844	218.853	0.635869	0.00257544	yes
Pld3	chr7:27532017-27553112	10.8884	16.9136	0.635383	0.00257544	yes
Zfp414	chr17:33629091-33631714	15.8504	24.6155	0.635054	0.00947319	yes
Fndc3a	chr14:72537952-72710003	10.9048	16.9218	0.633916	0.00257544	yes
Ceacam19	chr7:19875741-19887965	0.719186	1.11578	0.633619	0.0347854	yes
Nmd3	chr3:69722054-69749046	68.2699	105.904	0.63343	0.00301495	yes
Dnajc3	chr14:118937931-118981702	53.3306	82.666	0.63233	0.00064883	yes
Kmt2d	chr15:98831668-98871205	1.70986	2.65032	0.632286	0.00168106	yes
Ankrd17	chr5:90227165-90366185	20.5364	31.8316	0.632276	0.00257544	yes
Zfp592	chr7:80993683-81045162	2.70691	4.1955	0.632196	0.00454713	yes
1810055G02Rik	chr19:3708332-3717881	14.3819	22.2572	0.630015	0.00568302	yes
Prelid2	chr18:41875695-41951194	34.4975	53.3631	0.629353	0.00797055	yes
Usp10	chr8:119910851-119957557	29.1988	45.165	0.629294	0.00257544	yes
Ptprj	chr2:90429755-90580647	15.6467	24.2004	0.629177	0.00382257	yes
Cd24a	chr10:43579168-43584265	5.01706	7.75898	0.629027	0.0146074	yes
Taf15	chr11:83473107-83506740	14.4177	22.2628	0.626791	0.0034365	yes
Tgm2	chr2:158116404-158146392	1.84953	2.85455	0.626101	0.01225	yes
Plaur	chr7:24462499-24475873	25.2183	38.9038	0.625443	0.00419912	yes
Pfdn2	chr1:171345698-171358170	41.336	63.7243	0.624445	0.00168106	yes
Ifitm2	chr7:140954838-140955961	339.002	522.485	0.624095	0.00602054	yes
Trp53	chr11:69580358-69591873	38.1327	58.7686	0.624016	0.00217763	yes

Ndel1	chr11:68821445-68853131	33.7817	52.0522	0.623717	0.00454713	yes
Ylpm1	chr12:84996320-85070515	5.10997	7.87161	0.623345	0.00168106	yes
Galnt3	chr2:66082765-66124793	2.0638	3.17758	0.622628	0.01225	yes
Nsun3	chr16:62734851-62786716	5.89233	9.07203	0.622587	0.0151535	yes
Rslcan18,Zfp708	chr13:67069397-67114028	2.10413	3.23952	0.622558	0.0327099	yes
Tsc22d2	chr3:58415688-58466787	2.65346	4.08115	0.621104	0.00301495	yes
Atp6v0c	chr17:24163864-24169429	5.95558	9.15099	0.619685	0.0222603	yes
Rel	chr11:23741728-23770970	7.67119	11.7856	0.619497	0.00766564	yes
Ktn1	chr14:47649310-47736564	17.8773	27.4636	0.619392	0.00419912	yes
Cblb	chr16:52031548-52208046	0.907129	1.39332	0.619146	0.0159056	yes
Tab3	chrX:85574021-85634469	4.27456	6.56438	0.618885	0.00531709	yes
Emb	chr13:117220572-117274415	62.2764	95.6327	0.618819	0.00382257	yes
Foxj3	chr4:119539660-119629119	12.0153	18.4414	0.618073	0.00257544	yes
Clec5a	chr6:40529090-40910666	136.61	209.563	0.61732	0.0049451	yes
Angptl2	chr2:33133418-33371494	82.6188	126.695	0.61682	0.0049451	yes
Gxylt1	chr15:93239741-93275084	8.15745	12.5092	0.616796	0.00301495	yes
B330016D10Rik	chr4:141546161-141548313	2.15472	3.30409	0.616753	0.0353656	yes
Nucb2	chr7:116504368-116540588	18.9051	28.9883	0.616698	0.0049451	yes
Prr3	chr17:35972538-35979825	6.44963	9.8777	0.614958	0.0197691	yes
Igf2bp3	chr6:49085217-49214954	6.87967	10.5344	0.614697	0.00454713	yes
Stxbp3-ps	chr19:9557605-9559248	16.5067	25.2654	0.614107	0.01225	yes
Kdm7a	chr6:39118472-39206773	10.4087	15.9274	0.613721	0.00217763	yes
Slc24a3	chr2:145242610-145641939	0.619561	0.947796	0.61333	0.0466216	yes
Ube2d3	chr3:135438758-135467178	294.292	450.101	0.613003	0.00634725	yes
Ldlrad3	chr2:101950200-102186460	1.19182	1.82129	0.611795	0.0268919	yes
Bcl6	chr16:23965051-23988612	12.3171	18.8202	0.611627	0.0034365	yes
AA414768	chrX:12936872-12938541	8.5713	13.0895	0.610823	0.00666366	yes
Wdr83os	chr8:85080962-85082339	9.46441	14.4528	0.610763	0.0288111	yes
Dusp7	chr9:106368631-106375723	12.8356	19.5986	0.610601	0.00634725	yes
9930021J03Rik	chr19:29714401-29806009	5.08627	7.76483	0.610346	0.00454713	yes
Hspb6	chr7:30553301-30555439	2.74587	4.19044	0.609837	0.0476903	yes
Plek	chr11:16971205-17008718	90.3735	137.916	0.609816	0.0049451	yes
Gsap	chr5:21186266-21291701	11.4496	17.4716	0.609712	0.00217763	yes
Zfp36l2	chr17:84183927-84187947	38.578	58.8627	0.609574	0.00118536	yes
Tmem170b	chr13:41606215-41641357	10.0009	15.2419	0.607918	0.00568302	yes
Acvr1	chr2:58446437-58566828	6.07463	9.25613	0.607613	0.00666366	yes
Gm608	chr16:44173396-44227466	3.07653	4.68733	0.607463	0.00634725	yes
Zfp9	chr6:118461949-118479273	0.494225	0.752095	0.605746	0.0445611	yes
Irf2	chr8:46739744-46847458	5.046	7.66986	0.604059	0.00977864	yes
Zswim4	chr8:84210941-84237042	3.33541	5.06844	0.603678	0.010626	yes
Mtmr11	chr3:96161969-96171718	0.987503	1.49973	0.602841	0.0486014	yes
Sep-09	chr11:117199660-117362325	39.5725	60.0889	0.602598	0.00531709	yes
Usp36	chr11:118259652-118290244	5.96517	9.05371	0.601944	0.0049451	yes
Csf2rb	chr15:78325989-78351001	5.76358	8.74767	0.601933	0.0130772	yes
Rasa1	chr13:85214698-85289486	54.7217	83.0536	0.60193	0.00257544	yes
Zfp462	chr4:54947944-55083563	0.927028	1.40663	0.601553	0.010626	yes
Zfp800	chr6:28239930-28261601	25.359	38.4784	0.60155	0.00382257	yes
Arhgap23	chr11:97450159-97502400	0.923451	1.40081	0.601155	0.0288111	yes
Arid1a	chr4:133679007-133753611	6.24045	9.46553	0.601032	0.0034365	yes
Fubp1	chr3:152210457-152236830	68.4134	103.759	0.600881	0.00701693	yes
Cited2	chr10:17723227-17725674	72.7862	110.335	0.600153	0.00734768	yes
Tspyl2	chrX:152336851-152342484	3.49552	5.29492	0.599101	0.0135968	yes
Ppp2r2a	chr14:67014055-67072471	9.64674	14.6105	0.598897	0.00602054	yes
Ints6	chr14:62663666-62830126	13.6952	20.7414	0.598845	0.00634725	yes
Scoc	chr8:83434491-83458396	13.4487	20.3671	0.598776	0.0103347	yes
Msl2	chr9:101074761-101104799	30.8575	46.7311	0.598763	0.00301495	yes
Farp1	chr14:121035573-121283726	0.778374	1.17863	0.598581	0.0353656	yes
Arhgap31	chr16:38598342-38713035	2.1061	3.18762	0.597903	0.00666366	yes
Prrg4	chr2:104830740-104849850	1.99876	3.02505	0.597852	0.0222603	yes
Zbtb39	chr10:127739537-127747339	1.39539	2.10988	0.596498	0.0143874	yes
Ppargc1b	chr18:61298135-61400431	1.76279	2.664	0.595734	0.0176712	yes
Qpct	chr17:79051905-79090243	3.19119	4.82261	0.595722	0.0245865	yes
2900097C17Rik	chr2:156388062-156392979	43.7827	66.1396	0.595153	0.00602054	yes
Gm5434	chr12:36090378-36091829	7.95256	12.0125	0.595045	0.0166553	yes
Lyrm7	chr11:54839288-54860591	2.01443	3.0424	0.594837	0.0395897	yes

Ccdc186	chr19:56790962-56813683	10.1741	15.3645	0.594697	0.00701693	yes
Dusp4	chr8:34807609-34819894	14.8108	22.3655	0.594625	0.0049451	yes
Manf	chr9:106887414-106891938	96.2648	145.355	0.594505	0.0049451	yes
Per2	chr1:91415981-91459328	1.0402	1.57063	0.59449	0.0193303	yes
Tmem69	chr4:116551527-116555943	13.4368	20.2864	0.594327	0.00602054	yes
Ucp2	chr7:100493357-100499629	193.697	292.235	0.59333	0.00602054	yes
Dst	chr1:33908224-34308662	8.61732	13.0005	0.593256	0.00734768	yes
Fosl2	chr5:32136471-32157839	11.1863	16.8756	0.593208	0.00257544	yes
Frs2	chr10:117070126-117148474	3.69526	5.57446	0.593157	0.00858867	yes
Fnbp4	chr2:90745369-90781020	24.0929	36.3352	0.592758	0.00602054	yes
Ccdc15	chr9:37275834-37348392	1.56005	2.35184	0.5922	0.0213708	yes
Rab11fip2	chr19:59902883-59943654	5.58723	8.42106	0.591868	0.00766564	yes
Rbms2	chr10:128129469-128180297	1.47803	2.2275	0.591755	0.0239707	yes
Mbtd1	chr11:93886218-93946984	7.4575	11.2368	0.591463	0.00531709	yes
Cdyl2	chr8:116568723-116732991	1.44899	2.18218	0.590718	0.0148682	yes
H3f3b	chr11:116021960-116024504	513.53	773.229	0.590447	0.0127966	yes
Ccr2	chr9:124102182-124109140	2.1433	3.22698	0.590352	0.0233549	yes
Kcnq1ot1	chr7:143107253-143427042	2.46981	3.71844	0.590296	0.00602054	yes
Trp53inp1	chr4:11156440-11174377	1.35322	2.03697	0.590026	0.0292401	yes
Etv6	chr6:134035699-134270147	8.18602	12.3212	0.589906	0.00568302	yes
P2rx7	chr5:122643910-122691432	16.341	24.5935	0.589782	0.0171867	yes
Brd2	chr17:34112018-34122607	55.4684	83.3997	0.588376	0.00915681	yes
Pde8a	chr7:81213803-81333622	12.3642	18.5864	0.58808	0.00666366	yes
Eif4e	chr3:138526190-138557599	117.528	176.647	0.587864	0.00701693	yes
Ifitm1	chr7:140967428-140969827	15.7483	23.665	0.58756	0.0205128	yes
Zfp608	chr18:54888044-54990180	1.69079	2.54036	0.587331	0.013835	yes
Tnfrsf23	chr7:143665806-143685875	2.9921	4.49421	0.58691	0.0211532	yes
Rnps1	chr17:24414674-24425897	16.1124	24.1992	0.586783	0.0111526	yes
Slc25a37	chr14:69241850-69285103	6.50525	9.76944	0.586672	0.00886675	yes
Wdr54	chr6:83152709-83156379	3.89546	5.84924	0.586454	0.0481279	yes
Plxna1	chr6:89316313-89362613	1.98196	2.97544	0.586176	0.00977864	yes
Snhg17	chr2:158353699-158361522	5.4202	8.13516	0.585826	0.0125053	yes
Zmym6	chr4:127077382-127124374	5.27808	7.92178	0.585812	0.00766564	yes
Bmpr2	chr1:59764278-59878081	7.9857	11.9795	0.585077	0.00454713	yes
Setd1a	chr7:127777388-127800119	3.54516	5.31723	0.584824	0.00666366	yes
Btla	chr16:45224336-45252895	2.90138	4.35164	0.584823	0.0209453	yes
Hif1a	chr12:73907866-73947530	95.6363	143.436	0.584774	0.00886675	yes
E2f8	chr7:48866428-48881041	12.8101	19.1986	0.58372	0.00634725	yes
Hist2h2be	chr3:96221120-96223738	7.79702	11.685	0.583663	0.0146074	yes
Sfpq	chr4:127021300-127037014	111.5	167.074	0.583441	0.00977864	yes
Cldn12	chr5:5505014-5514976	2.10711	3.15452	0.582152	0.029439	yes
Gfpt1	chr6:87042845-87092207	18.3529	27.4651	0.581593	0.00454713	yes
Micall2	chr5:139706692-139736333	1.4436	2.1601	0.581427	0.0266413	yes
Rbm33	chr5:28317188-28419242	5.83122	8.71844	0.580272	0.00568302	yes
Slc39a13	chr2:91061780-91070315	11.9694	17.8873	0.579583	0.010626	yes
Wasl	chr6:24613809-24664995	5.03413	7.51979	0.57895	0.0109203	yes
Mef2d	chr3:88142394-88169167	7.28191	10.8774	0.57894	0.0125053	yes
Prr12	chr7:45027706-45052881	0.762764	1.13934	0.578886	0.0275447	yes
Ccdc71l	chr12:32378788-32382943	9.15358	13.6724	0.578855	0.00766564	yes
0610030E20Rik	chr6:72347316-72353160	6.15231	9.18695	0.578459	0.0103347	yes
Layn	chr9:51056779-51077094	4.28069	6.39179	0.578378	0.0218243	yes
Plagl2	chr2:153227768-153241358	3.28706	4.90715	0.578086	0.0100593	yes
Pelp1	chr11:70392880-70410031	7.92	11.814	0.576922	0.00734768	yes
Fam98a	chr17:75537085-75551946	35.2475	52.5689	0.576687	0.00797055	yes
Sf1	chr19:6363689-6378038	25.7555	38.4036	0.576361	0.00602054	yes
Dnajb2	chr1:75236422-75245692	4.70762	7.01857	0.576179	0.0467776	yes
Slc4a7	chr14:14703024-14799943	28.8623	43.0303	0.576167	0.00634725	yes
Pnn	chr12:59066918-59074017	63.5705	94.7604	0.575927	0.0116752	yes
Adat1	chr8:111966907-111992302	5.45664	8.13327	0.575823	0.010626	yes
Ago2	chr15:73101624-73184947	9.31092	13.8772	0.575721	0.00419912	yes
Rhoc	chr3:104789033-104794459	45.2997	67.5076	0.575547	0.00454713	yes
Dlg3	chrX:100767721-100818410	2.18445	3.25413	0.575003	0.0288111	yes
Polk	chr13:96480688-96542485	9.1067	13.5646	0.574844	0.00602054	yes
Jmjd1c	chr10:67127257-67256326	26.172	38.9596	0.573955	0.00858867	yes
Snhg5	chr9:88521052-88522897	107.053	159.3	0.573416	0.00701693	yes

AI504432	chr3:107039503-107054322	1.05886	1.57478	0.572633	0.0397131	yes
Naa16	chr14:79334506-79390668	15.9549	23.7256	0.572449	0.00766564	yes
Pogz	chr3:94837566-94883567	3.88016	5.76975	0.572392	0.00858867	yes
Zfp318	chr17:46383765-46420918	4.19637	6.23563	0.571392	0.0382672	yes
Prrc2a	chr17:35149085-35164877	11.6445	17.3007	0.571175	0.00634725	yes
Tank	chr2:61578585-61654169	65.6503	97.5139	0.570805	0.0135968	yes
R3hdm1	chr1:128103305-128237735	9.94085	14.7639	0.570636	0.00257544	yes
Ago3	chr4:126340677-126429542	3.85275	5.72181	0.570581	0.00829754	yes
Papd4	chr13:93147399-93192283	13.4353	19.95	0.570357	0.0133799	yes
Gltscr1l	chr17:46798115-46831413	2.87567	4.26899	0.569999	0.0111526	yes
Nhsl1	chr10:18407674-18533891	0.742249	1.10116	0.569047	0.0320864	yes
Gsk3b	chr16:38085063-38246079	7.95083	11.7931	0.568764	0.00531709	yes
Tcerg1	chr18:42511486-42575785	34.971	51.8661	0.568631	0.0130772	yes
Adam8	chr7:139978931-139992562	38.1568	56.5897	0.568598	0.00701693	yes
Prpf38b	chr3:108902806-108911704	60.5434	89.7836	0.568482	0.013835	yes
Atf7	chr15:102525945-102625464	3.19529	4.73683	0.567973	0.0328955	yes
Hook1	chr4:95959710-96024274	5.5117	8.16908	0.567676	0.00915681	yes
Gpr179	chr11:97332108-97352073	1.79184	2.65536	0.567466	0.0135968	yes
Cdc73	chr1:143607498-143702684	17.4573	25.869	0.567397	0.00947319	yes
Dhrsx	chr4_GL456216_random:15880-3	35.6586	52.7985	0.566247	0.0111526	yes
Lcp2	chr11:34047200-34092280	24.75	36.6293	0.565569	0.00454713	yes
Ccdc88a	chr11:29374171-29510808	18.1673	26.8831	0.565354	0.00947319	yes
Rad54l2	chr9:106688079-106789213	4.32902	6.40542	0.565252	0.00666366	yes
Arhgef12	chr9:42963841-43105718	3.5929	5.31539	0.565027	0.00734768	yes
Samsn1	chr16:75858793-75909266	9.60431	14.1988	0.56401	0.0135968	yes
Trim30a	chr7:104409025-104465193	4.20413	6.21283	0.563444	0.0186262	yes
Slc35a3	chr3:116670797-116712280	50.7842	75.0477	0.563427	0.00454713	yes
Rab11fip1	chr8:27138772-27174646	3.25659	4.8111	0.563005	0.0133799	yes
C1galt1	chr6:7845223-7872042	26.78	39.5261	0.56165	0.00915681	yes
Nabp2	chr10:128401394-128409796	31.5137	46.5126	0.561642	0.0197691	yes
Med13l	chr5:118560718-118765437	3.50233	5.16884	0.561527	0.00915681	yes
Fip1l1	chr5:74535481-74702903	51.8121	76.4651	0.561513	0.018127	yes
Pigl	chr11:62458459-62513900	3.45861	5.10083	0.560538	0.0320864	yes
Mef2a	chr7:67231162-67372858	51.6769	76.2045	0.560357	0.0153915	yes
Trp53bp2	chr1:182409166-182462436	2.56483	3.78094	0.559881	0.0226785	yes
Atp10a	chr7:58658201-58829426	1.11928	1.64967	0.559608	0.0361626	yes
Tgfb1	chr7:25687001-25704996	19.574	28.8494	0.559601	0.0111526	yes
Bcl2l1	chr2:152754172-152831728	13.1399	19.3641	0.559424	0.0111526	yes
Zbtb21	chr16:97947434-97962621	5.04178	7.4294	0.559314	0.0103347	yes
Epc2	chr2:49451485-49551609	19.0792	28.1113	0.559152	0.0109203	yes
Smg7	chr1:152836994-152902646	27.4819	40.4606	0.558035	0.00797055	yes
Pfn1	chr11:70651846-70654650	1337.89	1969.73	0.558035	0.0347854	yes
N4bp1	chr8:86841138-86885258	9.20998	13.5579	0.557869	0.00858867	yes
Lacc1	chr14:77024200-77036617	6.92523	10.1938	0.557764	0.0159056	yes
Chd7	chr4:8690405-8868449	4.5514	6.69913	0.557663	0.00886675	yes
Reep3	chr10:67005074-67096988	16.7734	24.6812	0.55724	0.00797055	yes
Iqgap2	chr13:95627176-95891922	2.60013	3.82426	0.556599	0.013835	yes
Mdc1	chr17:35841497-35859670	6.42072	9.44182	0.556331	0.0100593	yes
Nktr	chr9:121719180-121759943	17.6066	25.8822	0.55584	0.00829754	yes
Zfhx4	chr3:5177823-5415855	3.01825	4.43498	0.555215	0.00829754	yes
Sec61a2	chr2:5870986-5895353	8.37161	12.2982	0.554872	0.0174052	yes
Zmiz1	chr14:25459184-25666747	3.8664	5.67832	0.554475	0.010626	yes
Ppp1r12b	chr1:134765942-134955940	4.01926	5.90225	0.554334	0.0250369	yes
Fam193a	chr5:34369932-34486458	4.74647	6.96658	0.553597	0.0109203	yes
Map4k4	chr1:39900912-40026310	52.8739	77.5793	0.553116	0.0211532	yes
Zcchc2	chr1:105990405-106034079	12.1613	17.8396	0.55278	0.0103347	yes
Camk2d	chr3:126596950-126846326	8.79783	12.9044	0.552645	0.0114308	yes
Clk2,Clk2-scamp3	chr3:89164794-89182770	35.6553	52.2906	0.552437	0.0215983	yes
Ccdc138	chr10:58497936-58576244	2.67048	3.91539	0.552059	0.0405196	yes
Ctif	chr18:75431220-75697696	0.732639	1.07385	0.551622	0.0388425	yes
Eno1	chr4:150237196-150248873	104.152	152.64	0.551433	0.00766564	yes
Mir17hg,Mir19b-:	chr14:115044304-115046728	39.6907	58.1516	0.551017	0.0125053	yes
Satb2	chr1:56793980-56971334	2.50044	3.66303	0.550856	0.0209453	yes
Chd9	chr8:90828834-91054508	6.51782	9.54522	0.550389	0.00734768	yes
Hspb11	chr4:107253933-107279888	44.1069	64.5813	0.550112	0.0316924	yes

Myo10	chr15:25622549-25813671	25.706	37.6383	0.550098	0.0100593	yes
Kdm2a	chr19:4316146-4397077	16.1034	23.5701	0.549593	0.00858867	yes
Ticam1	chr17:56269461-56276767	1.93232	2.82739	0.549137	0.0328955	yes
Smim10l1	chr6:133105238-133110899	41.3383	60.4822	0.549031	0.0114308	yes
4930503L19Rik	chr18:70453139-70501065	9.16695	13.404	0.548153	0.0207355	yes
Prkaa1	chr15:5143860-5181899	24.8448	36.2987	0.546971	0.00829754	yes
Pld1	chr3:27938679-28133362	2.5152	3.67416	0.546743	0.0215983	yes
Zfp959	chr17:55892092-55898930	7.35182	10.7017	0.54166	0.0233549	yes
Sec23ip	chr7:128744869-128784835	18.5378	26.9836	0.541613	0.00915681	yes
Paxip1	chr5:27740080-27791550	11.3061	16.4568	0.54158	0.0100593	yes
Chd1	chr17:15704966-15772612	35.7402	51.987	0.540605	0.0166553	yes
Cd86	chr16:36603868-36666077	2.50342	3.63837	0.539393	0.040848	yes
Drosha	chr15:12824814-12935291	13.4163	19.492	0.538895	0.01225	yes
Hnrnpdl	chr5:100033578-100039222	143.664	208.714	0.538827	0.0259255	yes
Malt1	chr18:64887755-65689436	24.5996	35.7362	0.538754	0.013835	yes
Ccnt1	chr15:98543210-98570864	25.2142	36.6224	0.538491	0.0235697	yes
Rap1b	chr10:117814596-117845974	220.934	320.885	0.53844	0.0159056	yes
Cybb	chrX:9435253-9469324	82.2562	119.436	0.53804	0.0119604	yes
1700017B05Rik	chr9:57252321-57262599	11.1492	16.1816	0.537413	0.00886675	yes
Stxbp3	chr3:108793179-108840502	18.8972	27.4194	0.537025	0.0116752	yes
Top1	chr2:160645896-160722763	74.4781	107.984	0.535924	0.0164013	yes
Zrsr2	chrX:163935442-163958666	12.3255	17.8679	0.535721	0.0174052	yes
Nlrp3	chr11:59542685-59566956	12.2589	17.7703	0.535639	0.00886675	yes
Zbtb1	chr12:76370265-76388747	26.3193	38.142	0.535258	0.0114308	yes
Hhex	chr19:37434840-37440731	16.2252	23.51	0.535042	0.0146074	yes
Sfr1	chr19:47731755-47735588	137.854	199.716	0.534814	0.0305514	yes
B4galnt1	chr10:127165155-127172340	66.6077	96.4909	0.534705	0.0195507	yes
Nt5dc3	chr10:86779004-86838389	3.89753	5.64347	0.534025	0.0209453	yes
Arf3	chr15:98737625-98763118	31.4376	45.5133	0.533795	0.00977864	yes
Fgd6	chr10:94036000-94145339	4.8318	6.99475	0.53371	0.0133799	yes
Phf12	chr11:77982815-78030535	7.28158	10.5406	0.53363	0.0161452	yes
Setd5	chr6:113077638-113153424	17.2401	24.9545	0.533535	0.0109203	yes
Uhrf1bp1l	chr10:89744990-89819869	14.5539	21.0553	0.532773	0.00734768	yes
Nabp1	chr1:51469487-51478399	5.50838	7.96564	0.532163	0.0211532	yes
Spcs3	chr8:54520432-54529998	213.358	308.504	0.532013	0.0159056	yes
Ube2v2	chr16:15550985-15594518	2.66957	3.85936	0.531756	0.0323053	yes
Nsd1	chr13:55209781-55318325	1.47881	2.13753	0.531513	0.0183466	yes
Bnip3	chr7:138890835-138909506	58.0194	83.8318	0.530962	0.00915681	yes
Ocrl	chrX:47912455-47965866	7.1264	10.2916	0.530222	0.0148682	yes
Notch2	chr3:98013537-98150367	9.29072	13.4092	0.529364	0.0135968	yes
Klf8	chrX:153238044-153396134	2.36124	3.40758	0.529204	0.0361626	yes
Uvssa	chr5:33378695-33419754	1.93043	2.78572	0.529124	0.0288111	yes
Sf3b4	chr3:96172505-96177564	7.75034	11.1838	0.529079	0.0284207	yes
Pigu	chr2:155278251-155357424	52.6821	76.0066	0.528812	0.0127966	yes
Erp44	chr4:48193330-48279589	60.9393	87.9174	0.528775	0.013835	yes
Snrpd3	chr10:75518041-75535440	81.1396	116.996	0.527976	0.0151535	yes
Chn2	chr6:54039931-54430221	1.86252	2.68406	0.527159	0.0442747	yes
Rrs1	chr1:9545407-9547455	90.0014	129.695	0.527103	0.0125053	yes
Ttc39b	chr4:83220300-83324189	8.86613	12.7735	0.526782	0.00886675	yes
Neo1	chr9:58874679-59036441	2.24865	3.23962	0.526767	0.0254961	yes
Ahcyl1	chr3:107663119-107696548	40.1307	57.7901	0.526116	0.0146074	yes
D930016D06Rik	chr5:104525734-104554211	2.89916	4.17426	0.525887	0.0445611	yes
Ube2d1	chr10:71254979-71285262	16.2737	23.4293	0.525767	0.0243535	yes
Ino80c	chr18:24104760-24121819	27.0677	38.9643	0.525582	0.016917	yes
Ifitm3	chr7:141009589-141010744	280.979	404.413	0.525368	0.0114308	yes
Pnpla2	chr7:141455187-141460743	5.02161	7.22718	0.525281	0.0373535	yes
Atrnl1	chr19:57611033-58133340	2.30939	3.32365	0.525256	0.027115	yes
Slc39a14	chr14:70303466-70351424	13.3882	19.258	0.5245	0.0116752	yes
Gas5	chr1:161035165-161038537	166.678	239.468	0.522768	0.0281891	yes
Rpl7a	chr2:26910806-26913311	215.433	309.456	0.522496	0.0363016	yes
Ubp1	chr9:113930933-114026751	21.4083	30.7515	0.522489	0.0193303	yes
Gpkow	chrX:7697133-7710259	10.7303	15.4096	0.522147	0.0143874	yes
Ubn2	chr6:38433924-38512763	4.88343	7.01208	0.52195	0.0284207	yes
Disp1	chr1:183086263-183221529	3.52902	5.06186	0.520403	0.0337719	yes
Srsf11	chr3:158010492-158036639	95.0157	136.258	0.520102	0.0243535	yes

Tnfsf9	chr17:57092022-57107757	31.5026	45.1748	0.520048	0.0430991	yes
Slc20a1	chr2:129198772-129211612	40.185	57.6067	0.51958	0.0176712	yes
Chst12	chr5:140505608-140525238	7.60157	10.8948	0.519266	0.0327099	yes
Ccdc115	chr1:34436669-34439672	106.943	153.25	0.519043	0.0222603	yes
Sema3c	chr5:17574815-17730267	2.07967	2.97762	0.517806	0.0391822	yes
Sash1	chr10:8722218-8886070	10.159	14.5325	0.516524	0.0146074	yes
Prkca	chr11:107933386-108343888	2.70947	3.87457	0.516027	0.0200293	yes
Mt1	chr8:94179088-94180327	1302.37	1861.6	0.515397	0.0345906	yes
Elmsan1	chr12:84149173-84218881	9.03452	12.9132	0.515322	0.016917	yes
Slc2a1	chr4:119108744-119137329	38.6836	55.2829	0.51511	0.013835	yes
Cacfd1	chr2:27009925-27021089	6.23203	8.90542	0.514982	0.0497441	yes
Luc7l2	chr6:38551443-38609470	49.7273	71.0514	0.514823	0.0209453	yes
Il13ra1	chrX:36112107-36171261	26.6857	38.1111	0.514148	0.016917	yes
Zswim6	chr13:107724616-107890064	2.72603	3.89314	0.514135	0.0286094	yes
Msn	chrX:96096044-96168553	121.028	172.796	0.513735	0.0290402	yes
Edem1	chr6:108828640-108859356	92.3415	131.827	0.513596	0.0193303	yes
Cass4	chr2:172393793-172433757	4.40169	6.28354	0.513519	0.024169	yes
Exosc1	chr19:41922979-41933314	50.8492	72.553	0.51281	0.0153915	yes
Ets2	chr16:95702406-95721049	20.7079	29.5456	0.512766	0.0161452	yes
Taf3	chr2:9914551-10048609	3.3795	4.82121	0.512586	0.0235697	yes
Utrn	chr10:12382187-12861735	1.74955	2.49418	0.511586	0.0215983	yes
BC005561	chr5:104508351-104522383	7.45733	10.6302	0.51144	0.017885	yes
Ccdc59	chr10:105841478-105847510	32.3376	46.0549	0.510139	0.0224583	yes
Sec24b	chr3:129983184-130060907	8.66136	12.3351	0.510107	0.0186262	yes
Smap2	chr4:120968316-121017247	12.2428	17.4188	0.508705	0.0213708	yes
Rela	chr19:5637489-5648130	10.7723	15.3214	0.508229	0.0222603	yes
Micall1	chr15:79108982-79141251	2.4755	3.52039	0.508017	0.0275447	yes
Mlkl	chr8:111311799-111337903	12.544	17.8382	0.507971	0.0259255	yes
Ncoa6	chr2:155390655-155440783	2.95115	4.19571	0.507638	0.0467776	yes
Lrp8	chr4:107802258-107876840	12.7671	18.1486	0.507433	0.0164013	yes
Zfp827	chr8:79028436-79193766	1.58571	2.25407	0.507403	0.0380686	yes
Baz2a	chr10:128092782-128129303	2.18122	3.09865	0.506501	0.0309976	yes
Aak1	chr6:86849516-87003227	1.83447	2.60591	0.506423	0.0202883	yes
Mmp8	chr9:7558428-7568486	28.0334	39.8117	0.506043	0.0183466	yes
Vimp	chr7:66079648-66089405	68.8461	97.7035	0.505036	0.017885	yes
5730508B09Rik	chr3:127869687-127896323	14.6717	20.8133	0.504466	0.0432835	yes
Appbp2	chr11:85191309-85235120	23.17	32.8665	0.504359	0.0174052	yes
A830080D01Rik	chrX:159532667-159593081	8.09838	11.4781	0.503183	0.0233549	yes
Pde7a	chr3:19223108-19311322	12.2705	17.389	0.502979	0.0146074	yes
Whsc1l1	chr8:25601600-25719667	13.5175	19.153	0.502747	0.0275447	yes
Apc	chr18:34207774-34322190	5.71315	8.0947	0.502691	0.0166553	yes
Pkib	chr10:57631980-57741112	2.33437	3.30698	0.502481	0.0449797	yes
Mcl1	chr3:95658720-95663178	150.607	213.315	0.502192	0.0254961	yes
Cep250	chr2:155956557-155998900	6.93379	9.81804	0.501791	0.0197691	yes
Sos1	chr17:80393751-80480453	6.13761	8.68522	0.500885	0.0183466	yes
Tug1	chr11:3639784-3648814	25.3724	35.9026	0.50083	0.0191506	yes
Zeb2	chr2:44983511-45114084	23.8375	33.7247	0.500572	0.0239707	yes
Iws1	chr18:32067733-32104331	23.4158	33.1236	0.500377	0.0243535	yes
Zbtb6	chr2:37425499-37430919	20.5559	29.0727	0.500115	0.0197691	yes
Bdp1	chr13:100017993-100104070	22.0778	31.224	0.500061	0.024169	yes
Plaa	chr4:94565138-94603247	47.7214	67.4772	0.499765	0.0243535	yes
Sypl	chr12:32953944-32979502	22.3578	31.6027	0.49927	0.0159056	yes
Pdcd6ip	chr9:113651743-113708259	36.3849	51.429	0.499242	0.0161452	yes
Alg12	chr15:88805242-88819318	5.69883	8.05486	0.499192	0.0415334	yes
Golim4	chr3:75876182-75956949	21.7267	30.7012	0.498825	0.0220255	yes
Trip11	chr12:101837371-101913171	11.9564	16.8762	0.497205	0.0195507	yes
Xpo6	chr7:126101718-126200408	18.0904	25.5329	0.497134	0.0211532	yes
Ccnh	chr13:85189476-85213723	59.8842	84.4581	0.496061	0.0209453	yes
Mark2	chr19:7275395-7341860	9.31866	13.1374	0.49548	0.0220255	yes
Tmem131	chr1:36792188-36939527	9.72004	13.7001	0.495152	0.0186262	yes
Myh9	chr15:77760584-77842175	53.6309	75.5815	0.494968	0.0328955	yes
Arrdc3	chr13:80883421-80896043	10.2427	14.4304	0.494513	0.0226785	yes
Cpsf7	chr19:10525243-10547735	14.8841	20.9655	0.494242	0.0237709	yes
Ube2l6	chr2:84798827-84810003	21.8724	30.8066	0.494127	0.0327099	yes
Clic4	chr4:135213969-135272760	177.248	249.616	0.493939	0.0349796	yes

Pdia3	chr2:121413901-121438686	346.562	487.771	0.493091	0.0451403	yes
Stim2	chr5:53998522-54121057	16.4224	23.1097	0.492828	0.0235697	yes
Syne1	chr10:5020195-5194707	4.01784	5.65224	0.492403	0.0228852	yes
Itgav	chr2:83724396-83806917	20.5849	28.9519	0.492068	0.0239707	yes
Pcf11	chr7:92643711-92669912	20.9597	29.4785	0.492049	0.0233549	yes
Nat10	chr2:103721258-103761250	34.1282	47.9564	0.49076	0.0195507	yes
Atrx	chrX:105797614-105929372	41.1207	57.7796	0.490697	0.0405196	yes
Rfx7	chr9:72532239-72622949	9.46879	13.3037	0.490576	0.0220255	yes
Arl8a	chr1:135146833-135156268	15.5991	21.907	0.489932	0.0290402	yes
Baz1a	chr12:54892988-54986336	48.6269	68.2811	0.489733	0.0375376	yes
Mndal	chr1:173857219-173880187	14.6182	20.5236	0.489518	0.0337719	yes
Smad4	chr18:73639012-73703741	24.9086	34.9686	0.489418	0.0197691	yes
Icam1	chr9:21015959-21028796	19.104	26.8191	0.489384	0.0243535	yes
Dgke	chr11:89037581-89060748	4.52	6.3431	0.488864	0.0373535	yes
Eif4g3	chr4:137993455-138207079	11.0489	15.504	0.488729	0.0250369	yes
Flnb	chr14:7817956-7951587	15.8888	22.2713	0.487174	0.0213708	yes
Ccdc55	chr11:77044291-77078437	21.5999	30.2595	0.486364	0.0366609	yes
Cep170	chr1:176733652-176807124	21.9357	30.7283	0.486292	0.0188838	yes
Sbno1	chr5:124368701-124425914	35.6104	49.8762	0.486054	0.0296658	yes
Rnpc3	chr3:113605066-113630149	18.0302	25.2412	0.485365	0.0368647	yes
Vezf1	chr11:88068278-88084729	21.4784	30.0679	0.485334	0.0207355	yes
Atf1	chr15:100227858-100261248	15.1629	21.2226	0.485061	0.0275447	yes
Fmnl1	chr11:103171137-103198900	22.9786	32.1594	0.484948	0.0224583	yes
Atad2b	chr12:4917352-5047410	11.6258	16.2702	0.484909	0.0191506	yes
Alg3	chr16:20605457-20610749	17.4297	24.3901	0.484755	0.0492804	yes
Hs3st3b1	chr11:63884692-63922284	3.16526	4.42919	0.48472	0.0470882	yes
Rbm27	chr18:42275352-42341540	19.3331	27.0465	0.484371	0.0239707	yes
Ints9	chr14:64950044-65039835	9.37244	13.1042	0.48353	0.0390214	yes
Upf3b	chrX:37091833-37110322	29.5633	41.3196	0.483017	0.0375376	yes
Naa15	chr3:51416015-51475985	18.7451	26.1936	0.482701	0.0248059	yes
Gpatch4	chr3:88043105-88055994	31.3063	43.7342	0.482309	0.0328955	yes
Tnrc6a	chr7:123123884-123195296	10.0665	14.0606	0.4821	0.0226785	yes
Ammecr1	chrX:142853473-142966728	11.0307	15.4063	0.481995	0.0331273	yes
Dph6	chr2:114516417-114654928	17.8807	24.9516	0.480729	0.0292401	yes
Dot1l	chr10:80755205-80794347	6.30651	8.79783	0.480305	0.0308046	yes
Sgk1	chr10:21882183-21999902	6.83233	9.52574	0.479454	0.0494491	yes
Sh3rf1	chr8:61224170-61396072	2.47178	3.44594	0.479346	0.0487312	yes
Abracl	chr10:18011259-18023252	109.445	152.477	0.478384	0.0312197	yes
Rbm26	chr14:105106727-105177327	29.1733	40.6182	0.477478	0.0391822	yes
Cd164	chr10:41519499-41531042	42.8198	59.6129	0.477346	0.027378	yes
Arpp19	chr9:75037613-75060313	40.6445	56.5775	0.477168	0.0220255	yes
Dpagt1	chr9:44326844-44333600	16.5102	22.981	0.477087	0.0407291	yes
Ptch1	chr13:63511532-63565520	4.36326	6.06839	0.475906	0.0447802	yes
Zic2	chr14:122475383-122480328	8.14571	11.325	0.475405	0.0462882	yes
Pigt	chr2:164497524-164508301	25.5309	35.4942	0.475338	0.0327099	yes
Ap3m1	chr14:21033741-21052442	35.0867	48.7299	0.473884	0.037051	yes
Atf7ip	chr6:136518850-136607379	5.8633	8.1427	0.473794	0.0332955	yes
Rnf19b	chr4:129058270-129084526	17.1669	23.8393	0.473717	0.027115	yes
Xiap	chrX:42067835-42109664	53.2862	73.9923	0.473615	0.032539	yes
Camsap1	chr2:25926837-25983282	5.20294	7.22249	0.473169	0.0298919	yes
Klf10	chr15:38291463-38300711	21.8682	30.3478	0.47276	0.0237709	yes
Fra10ac1	chr19:38188478-38224132	26.4444	36.6852	0.472235	0.0380686	yes
Safb	chr17:56584981-56606294	26.769	37.1354	0.47223	0.0300881	yes
Nup214	chr2:31974449-32053975	6.55771	9.0961	0.472055	0.0281891	yes
Abhd5	chr9:122351615-122381523	14.5436	20.1716	0.471942	0.0281891	yes
Lclat1	chr17:73107984-73243366	8.17762	11.3411	0.471804	0.0376868	yes
Psme3	chr11:101316250-101323530	86.3344	119.716	0.471603	0.0305514	yes
Larp1	chr11:58009063-58062032	27.3284	37.8946	0.471592	0.0261255	yes
Gm11545	chr11:94755135-94761182	6.89498	9.56026	0.471504	0.0415334	yes
Huwe1	chrX:151803281-151935417	26.8799	37.2651	0.471294	0.0413811	yes
Clec16a	chr16:10545338-10744878	3.08645	4.27746	0.470805	0.0372196	yes
Scai	chr2:39066214-39190730	3.27657	4.54019	0.470563	0.0438907	yes
Eps15l1	chr8:72340995-72421474	12.5819	17.4247	0.46978	0.0382672	yes
Ubl7	chr9:57910985-57929968	21.1191	29.2459	0.46969	0.0444182	yes
B4galt6	chr18:20684598-20746404	5.06634	7.01494	0.469486	0.0349796	yes

Rwdd4a	chr8:47533644-47552837	36.8016	50.9463	0.469212	0.0284207	yes
Sos2	chr12:69583760-69681852	10.4822	14.5078	0.468896	0.0349796	yes
Pdia4	chr6:47796140-47813512	122.763	169.886	0.468685	0.0319128	yes
Traf6	chr2:101678419-101701668	10.3223	14.2834	0.468579	0.0303127	yes
1810013L24Rik	chr16:8830099-8858924	16.1535	22.3475	0.468268	0.029439	yes
Gnpnat1	chr14:45351185-45388796	27.7973	38.4484	0.467978	0.0464453	yes
Hmgn5	chrX:109004536-109013380	10.4266	14.407	0.4665	0.0469332	yes
Lrp6	chr6:134446477-134566913	12.897	17.8099	0.465637	0.0257373	yes
Rbm34	chr8:126947172-126971079	9.3301	12.8786	0.465017	0.0345906	yes
Kcnk6	chr7:29221927-29232522	9.7231	13.4152	0.464375	0.0386629	yes
Sec63	chr10:42761495-42832514	17.0073	23.4402	0.462829	0.0286094	yes
Ankrd11	chr8:122883821-123042284	13.0897	18.0319	0.462127	0.0372196	yes
Ddx19a	chr8:110974990-110997823	19.6146	27.0079	0.461456	0.0391822	yes
Supt6	chr11:78206748-78245703	21.1996	29.1879	0.461331	0.0379037	yes
Nfxl1	chr5:72513303-72559645	9.30855	12.8093	0.460563	0.0428769	yes
Cd2ap	chr17:42792950-42876424	38.3529	52.768	0.460326	0.0445611	yes
Haus6	chr4:86581284-86612022	29.3619	40.3937	0.460183	0.0309976	yes
Dld	chr12:31331561-31351471	224.041	308.098	0.459625	0.0490909	yes
Rapgef2	chr3:79062528-79145875	7.43157	10.2196	0.459598	0.0343789	yes
N4bp2	chr5:65763520-65826784	4.4678	6.1376	0.45811	0.0453196	yes
Tgif1	chr17:70844204-70853532	30.8665	42.386	0.457545	0.0376868	yes
Hook3	chr8:26021420-26119224	13.5148	18.5558	0.457332	0.0292401	yes
Pdgfb	chr15:79995875-80014808	15.9028	21.8315	0.457126	0.0347854	yes
Larp1b	chr3:40950630-40977793	17.7904	24.4202	0.456974	0.0474965	yes
Degs1	chr1:182275769-182282759	86.4998	118.713	0.456712	0.0290402	yes
Prpf3	chr3:95830621-95855753	22.011	30.2025	0.456446	0.0380686	yes
Smim15	chr13:108044473-108049146	61.7328	84.7059	0.456426	0.0363016	yes
Por	chr5:135689144-135735326	36.0033	49.3735	0.455608	0.0386629	yes
Tmem245	chr4:56876012-56947429	7.81049	10.7103	0.455512	0.037051	yes
Ccdc82	chr9:13246978-13292353	12.4343	17.043	0.454856	0.0460913	yes
Zfc3h1	chr10:115384958-115432771	14.4077	19.7474	0.454826	0.0390214	yes
Erc1	chr6:119570795-119848150	5.66016	7.75663	0.454586	0.0462882	yes
Vcl	chr14:20929432-21033673	12.0654	16.5253	0.453802	0.0320864	yes
Ezr	chr17:6738130-6782780	29.243	40.0512	0.453756	0.0440544	yes
Tlk1	chr2:70712407-70825480	30.9323	42.3624	0.453668	0.0403634	yes
Itgb2	chr10:77530347-77565674	50.3621	68.9536	0.453288	0.0393598	yes
Anxa5	chr3:36448923-36475887	277.465	379.763	0.452793	0.0481279	yes
Zwint	chr10:72654845-72674964	72.2741	98.9143	0.4527	0.0413811	yes
Il1rl1	chr1:40429569-40465414	27.5945	37.7609	0.452514	0.0319128	yes
Klf6	chr13:5861488-5870393	29.2067	39.9659	0.45247	0.0343789	yes
Zfp871	chr17:32765496-32788287	13.7309	18.7886	0.452428	0.0373535	yes
Jdp2	chr12:85599104-85639878	50.1235	68.5302	0.451254	0.0390214	yes
Trps1	chr15:50654758-50890041	8.28211	11.3176	0.450495	0.0303127	yes
Spata13	chr14:60634728-60764556	8.86735	12.1173	0.450491	0.037051	yes
Bcor	chrX:12036737-12160355	6.24705	8.53286	0.449853	0.0482593	yes
Wnk1	chr6:119923968-120038655	18.7219	25.5699	0.44972	0.0464453	yes
Bptf	chr11:107033080-107131922	9.50828	12.9788	0.448902	0.0412463	yes
Plekha1	chr7:130865909-130913302	34.0134	46.3961	0.447899	0.0331273	yes
Ptpn2	chr18:67665500-67724621	60.0347	81.8389	0.446989	0.0487312	yes
Tfe3	chrX:7762660-7775202	13.4176	18.2897	0.446904	0.0417032	yes
Qser1	chr2:104754792-104816696	7.30548	9.95699	0.44673	0.0373535	yes
Kpna3	chr14:61365185-61439947	72.5349	98.8086	0.44596	0.0438907	yes
Arf4	chr14:26638196-26657258	43.1321	58.7522	0.44588	0.0391822	yes
Cirh1a	chr8:106893639-106923094	70.6381	96.2106	0.44575	0.0376868	yes
Alas1	chr9:106233454-106247954	21.2979	28.9868	0.444685	0.0349796	yes
Mink1	chr11:70562880-70614482	10.4454	14.2156	0.444614	0.0391822	yes
Frmd4a	chr2:4152862-4614043	4.31491	5.87134	0.444362	0.0460913	yes
Mapk6	chr9:75386781-75410016	47.3811	64.4668	0.444246	0.0382672	yes
Rrp12	chr19:41862850-41896153	17.532	23.8505	0.444029	0.0390214	yes
Nlk	chr11:78567167-78697425	6.21413	8.45253	0.443831	0.0464453	yes
Ankhd1	chr18:36560602-36658908	17.5216	23.8254	0.443368	0.0449797	yes
Sep-11	chr5:93093456-93174958	48.0065	65.2763	0.44333	0.0401707	yes
Ski	chr4:155154074-155222535	11.0556	15.0304	0.443102	0.0444182	yes
Slmap	chr14:26413174-26533740	24.6411	33.4981	0.44301	0.0384653	yes
Atf2	chr2:73816508-73892639	20.2159	27.4782	0.442795	0.0445611	yes

Ptpn12	chr5:20986644-21055797	29.7003	40.3481	0.442021	0.0353656	yes
Rtf1	chr2:119675067-119735407	13.9467	18.9439	0.441814	0.0444182	yes
Slc38a1	chr15:96571417-96642913	30.0026	40.7229	0.440751	0.0487312	yes
Shisa5	chr9:109038566-109057792	32.7224	44.4002	0.44029	0.0484508	yes
Zbtb41	chr1:139422382-139453007	21.6758	29.3982	0.439641	0.035173	yes
Kif1b	chr4:149176318-149307733	9.97675	13.5308	0.439601	0.0487312	yes
Map4k3	chr17:80580512-80728025	12.776	17.3239	0.439329	0.0456964	yes
Ctsc	chr7:88278092-88310875	32.7276	44.3686	0.439029	0.040848	yes
Fndc3b	chr3:27416161-27710439	11.535	15.6288	0.43819	0.0405196	yes
Nipbl	chr15:8289823-8444463	16.6308	22.5283	0.437885	0.0476903	yes
Sh3bp2	chr5:34525783-34563639	18.5607	25.059	0.433081	0.0470882	yes
Trmt6	chr2:132804214-132816054	63.2306	85.3436	0.432659	0.0467776	yes
Exoc6b	chr6:84618485-85069513	9.02749	12.1839	0.432584	0.0482593	yes
Upf2	chr2:5951468-6056703	16.1809	21.8372	0.432498	0.0489214	yes
Gtf2a1	chr12:91555261-91590487	22.2118	29.9549	0.431468	0.0366609	yes
Cdk14	chr5:4803384-5380251	29.8603	40.2475	0.430667	0.0494491	yes
Mier3	chr13:111686177-111718594	11.9351	16.0674	0.428926	0.047996	yes
Selt	chr3:58576657-58593546	44.3146	59.5822	0.427098	0.0467776	yes
Cpne8	chr15:90487480-90679388	32.1346	43.1249	0.424394	0.0393598	yes
Ash1l	chr3:88965811-89079375	9.25358	12.4149	0.423994	0.047996	yes
Lpgat1	chr1:191718023-191784257	26.1429	35.0517	0.423065	0.0456964	yes
Irf2bp2	chr8:126588295-126593436	26.0321	34.8716	0.42176	0.0497441	yes
Zfp281	chr1:136624900-136630391	16.3501	21.7818	0.413828	0.0497441	yes
