# Supplementary material for: RNA Sequencing of Murine Norovirus-Infected Cells Reveals Transcriptional Alteration of Genes Important to Viral Recognition and Antigen Presentation
Source: Front Immunol. 2017 Aug 11;8:959. doi: 10.3389/fimmu.2017.00959 (PMC5554501; doi:10.3389/fimmu.2017.00959)
Supplement: Supplementary file 3 [file Table_3.PDF]

TABLE S3 Significantly expressed genes (Lox 12 hpt). Genes are ranked by fold-change. A negative fold-change represents downregulation. Significantly differentially expressed genes are a subset of this list with the following stringencies applied: 2-fold or more change in transcript abundance, and FPKM value >1 in at least one sample.

Gene ID	Chromosome	FPKM mock	FPKM lox	log2(fold change)	q-value	Significant
Hist1h4n	chr13:21831792-21832158	69.9565	0.158372	-8.78699	0.0436584	yes
Cldn11	chr3:31149919-31164326	16.3709	0.242438	-6.07738	0.000195236	yes
C1qa	chr4:136895915-136898844	2.0505	0.0318017	-6.01073	0.0331378	yes
Hist1h2ad	chr13:23574380-23574915	55.4702	1.30977	-5.40433	0.000195236	yes
Aqp1	chr6:55336298-55348555	13.8702	0.360135	-5.2673	0.000195236	yes
Tgfbi	chr13:56609602-56639339	1.46487	0.0383525	-5.25531	0.000537301	yes
Cacng8	chr7:3394116-3415605	22.9829	0.649931	-5.14413	0.000195236	yes
Igf1	chr10:87859055-87937047	4.54422	0.166853	-4.76738	0.000195236	yes
Hist1h3h	chr13:21717627-21718115	526.961	19.9779	-4.72122	0.000195236	yes
Klhl30	chr1:91351072-91362404	0.55649	0.0228603	-4.60544	0.0208855	yes
Dancr,Snora26	chr5:74093082-74094336	306.183	13.5121	-4.50206	0.00847587	yes
Bdh2	chr3:135281220-135304425	7.14531	0.329471	-4.43877	0.000195236	yes
Hist2h2aa2	chr3:96239778-96240374	9.56764	0.463526	-4.36744	0.000370691	yes
Slc9b2	chr3:135307699-135342767	0.590041	0.0294476	-4.32459	0.0325793	yes
Hist1h2ab	chr13:23751087-23751592	459.888	24.13	-4.25239	0.000195236	yes
Rgs18	chr1:144752840-144775421	1.53384	0.0835246	-4.1988	0.000195236	yes
Atp5l	chr9:44913247-44920742	3.55668	0.203794	-4.12535	0.0209746	yes
Olfml3	chr3:103735393-103738001	4.46387	0.256685	-4.12022	0.000195236	yes
Gm5086	chr13:97559999-97583994	4.13723	0.244798	-4.079	0.000195236	yes
Ppia	chr11:6415869-6419810	3.1643	0.18822	-4.0714	0.00428914	yes
Hist1h2bp	chr13:21787487-21789213	115.008	7.0931	-4.01917	0.000195236	yes
Cyp4f37	chr17:32621318-32636184	0.525715	0.0328124	-4.00197	0.0132116	yes
Icam2	chr11:106377655-106382641	3.38233	0.212713	-3.99104	0.000195236	yes
Snord22	chr19:8723486-8726326	1593.04	100.446	-3.98728	0.0218594	yes
Col15a1	chr4:47208011-47313165	1.15314	0.0730134	-3.98126	0.000195236	yes
Adgre5	chr8:83715176-83741311	8.10162	0.534223	-3.9227	0.0036408	yes
Apbb1	chr7:105558464-105581653	1.25282	0.0832211	-3.91208	0.000195236	yes
Hist1h2bg	chr13:23571399-23571863	333.725	22.2409	-3.90737	0.000195236	yes
Gpr176	chr2:118277097-118373419	2.91271	0.194717	-3.90291	0.000195236	yes
Haao	chr17:83831353-83846790	1.42241	0.0952449	-3.90055	0.000855787	yes
Rps28	chr17:33823036-33824498	4.42463	0.309249	-3.83872	0.0210807	yes
Zfyve28	chr5:34194893-34288324	0.883147	0.0623336	-3.82457	0.000195236	yes
Spns3	chr11:72498155-72550246	1.31706	0.0957365	-3.78211	0.000855787	yes
Hist1h2bk	chr13:22035820-22036320	382.229	28.066	-3.76754	0.000195236	yes
Olfr112	chr17:37563238-37569451	0.618982	0.0456724	-3.7605	0.00311626	yes
S1pr1	chr3:115710432-115715055	14.1363	1.05028	-3.75057	0.000195236	yes
Syngr1	chr15:80091333-80119501	37.2142	2.87784	-3.69279	0.000195236	yes
Odf3l1	chr9:56848658-56851963	2.87091	0.222891	-3.6871	0.000195236	yes
Hp	chr8:109575127-109579172	2.49022	0.194233	-3.68041	0.000195236	yes
Zfp651	chr9:121760032-121771742	3.42576	0.274185	-3.6432	0.000195236	yes
Opcml	chr9:27791268-28925048	0.648343	0.0519025	-3.64288	0.000195236	yes
Eno3	chr11:70657175-70662513	36.5989	3.10068	-3.56114	0.000195236	yes
Olfr113	chr17:37574482-37575421	1.0435	0.0893026	-3.54658	0.0450719	yes
Rasgrp2	chr19:6400582-6415216	1.14802	0.100158	-3.5188	0.000195236	yes
Hist1h2bm	chr13:21722043-21722526	1068.64	94.3886	-3.50102	0.000195236	yes
Snora78	chr17:24719530-24719965	177.401	15.8647	-3.48312	0.000195236	yes
Scarna6	chr1:87756010-87792428	267.813	24.2426	-3.46561	0.0362595	yes
Olfr111	chr17:37529956-37530997	2.10328	0.191333	-3.45848	0.00174394	yes
Rnase6	chr14:51129067-51131121	0.638815	0.0587388	-3.44301	0.0164008	yes
Myadml2	chr11:120646030-120648337	2.68936	0.248139	-3.43804	0.000195236	yes
5430437J10Rik	chr15:5496317-5594983	0.880277	0.0822045	-3.42067	0.0222609	yes
Tmem119	chr5:113793728-113800352	2.18936	0.204491	-3.4204	0.000195236	yes
Hist1h4m	chr13:21811745-21812150	63.0486	5.91995	-3.41281	0.000195236	yes
Cyp2s1	chr7:25802475-25816530	0.935972	0.0878913	-3.41267	0.000195236	yes
Hist1h2ah	chr13:22035121-22035643	178.92	16.8924	-3.40486	0.000195236	yes
Ambp	chr4:63143278-63154142	1.27436	0.122128	-3.38331	0.000195236	yes
Rps15a-ps4	chr4:132219892-132220589	7.94114	0.76168	-3.38209	0.000195236	yes
Cbx3	chr6:51470615-51483704	4.34462	0.426784	-3.34765	0.000195236	yes
Fam189a2	chr19:23972749-24031019	0.466128	0.0458887	-3.34452	0.00428914	yes

Psrc1	chr3:108383803-108388231	17.3721	1.7125	-3.3426	0.000195236	yes
Lrrc32	chr7:98494221-98501830	0.513315	0.0510596	-3.32959	0.000195236	yes
Scel	chr14:103513340-103613346	2.75637	0.276293	-3.3185	0.000195236	yes
Tst	chr15:78399555-78405859	1.00167	0.100711	-3.31412	0.00311626	yes
Snord89	chr1:39548746-39548840	748.863	76.3808	-3.29342	0.000855787	yes
Cxcr4	chr1:128588198-128592299	5.98468	0.614815	-3.28305	0.000195236	yes
Hist1h4i	chr13:22040959-22041362	648.407	67.1425	-3.2716	0.000195236	yes
Gpr183	chr14:121876860-122021035	70.7361	7.35382	-3.26588	0.000195236	yes
Fam81a	chr9:70089309-70141557	1.33185	0.140374	-3.24609	0.000195236	yes
Itga8	chr2:12106659-12312315	0.841358	0.088924	-3.24207	0.000195236	yes
Pacrg	chr17:10403011-10840191	1.34036	0.141982	-3.23884	0.000370691	yes
Ube2s	chr7:4808013-4812340	4.93093	0.527927	-3.22345	0.000195236	yes
Cd300lg	chr11:102041510-102055617	0.481261	0.0518888	-3.21332	0.0361606	yes
Mcrs1	chr15:99224975-99262041	6.20857	0.672575	-3.2065	0.000195236	yes
Hist1h3c	chr13:23745041-23745521	937.594	101.587	-3.20624	0.000195236	yes
Neurl1b	chr17:26414964-26446342	6.7112	0.731957	-3.19674	0.000195236	yes
Ier5l	chr2:30472640-30474199	3.59036	0.394122	-3.18742	0.000195236	yes
Dlg3	chrX:100767721-100818410	2.14836	0.236172	-3.18532	0.000195236	yes
Serpinb12	chr1:106934448-106957080	8.99206	0.994887	-3.17605	0.000195236	yes
Hist1h4a	chr13:23760794-23761249	679.571	76.1517	-3.15768	0.000195236	yes
Oaz1	chr10:80826655-80829290	35.5248	4.05191	-3.13215	0.000195236	yes
Nrg2	chr18:36017657-36197160	0.536008	0.0613357	-3.12746	0.000195236	yes
Amz1	chr5:140724126-140753312	8.02701	0.919291	-3.12627	0.000195236	yes
Arhgef39	chr4:43496143-43499660	24.1046	2.76153	-3.12577	0.000195236	yes
5830454E08Rik	chr9:120577330-120578073	1.13758	0.130442	-3.12449	0.0171279	yes
Acss1	chr2:150618110-150668932	0.808992	0.0929829	-3.12109	0.000195236	yes
Dusp9	chrX:73639440-73643514	1.07133	0.1242	-3.10866	0.000195236	yes
F630028O10Rik,Mir223	chrX:96239925-96243642	7.39183	0.858174	-3.10659	0.000195236	yes
Cd24a	chr10:43579168-43584265	4.93337	0.573121	-3.10566	0.000195236	yes
Hist1h4k	chr13:21750144-21750553	1383.03	161.144	-3.10141	0.000195236	yes
Rdm1	chr11:101627948-101636081	9.57508	1.11906	-3.097	0.000195236	yes
Slamf8	chr1:172581376-172590568	2.06079	0.241128	-3.09533	0.000195236	yes
C1qb	chr4:136880144-136886177	2.65416	0.315664	-3.07179	0.000195236	yes
Hist1h2ae	chr13:23570662-23571220	68.0835	8.16468	-3.05984	0.000195236	yes
Hist1h4c	chr13:23698083-23698458	1308.11	157.604	-3.05312	0.000195236	yes
2310040G24Rik	chr6:86483375-86488227	8.79406	1.06232	-3.04931	0.000195236	yes
Hspb7	chr4:141420778-141425310	1.15984	0.140148	-3.04891	0.000195236	yes
Hist1h2ag	chr13:22042477-22042949	263.865	32.0204	-3.04274	0.000195236	yes
Icam4	chr9:21029372-21030531	0.905696	0.110368	-3.03671	0.0258596	yes
Hist1h1b	chr13:21779831-21780625	1369.33	167.906	-3.02775	0.000195236	yes
Rab3il1	chr19:10018227-10035586	23.1519	2.84217	-3.02607	0.000195236	yes
Cst7	chr2:150570414-150578944	82.6608	10.172	-3.0226	0.000195236	yes
Serpinf1	chr11:75410028-75422623	5.41439	0.670403	-3.0137	0.000195236	yes
Snora74a	chr18:35553409-35558316	652.765	81.4149	-3.0032	0.000370691	yes
Cd28	chr1:60746387-60773359	0.53996	0.0684866	-2.97896	0.000195236	yes
Gpr141	chr13:19749681-19824257	3.59049	0.468362	-2.93848	0.000195236	yes
Lifr	chr15:7129571-7197489	1.09848	0.14406	-2.93077	0.000195236	yes
Rgs8	chr1:153653036-153697665	1.16682	0.153296	-2.9282	0.000195236	yes
Abca9	chr11:110100821-110168153	7.75442	1.01923	-2.92753	0.000195236	yes
Hist1h2bh	chr13:23542922-23543444	169.973	22.4347	-2.9215	0.000195236	yes
Ppfia4	chr1:134296782-134332928	5.23201	0.695179	-2.91191	0.000195236	yes
D630023F18Rik	chr1:65105284-65123214	1.17192	0.156406	-2.9055	0.000195236	yes
Hist1h1c	chr13:23738806-23739531	1820.26	243.501	-2.90214	0.000195236	yes
Rpl22l1	chr3:28805510-28807415	40.2347	5.50224	-2.87035	0.000195236	yes
Rarb	chr14:16430839-17082331	2.06173	0.283786	-2.86098	0.000195236	yes
Terc	chr3:96414436-96414833	230.322	31.8334	-2.85504	0.000195236	yes
Shisa8	chr15:82206951-82212815	0.818511	0.113532	-2.8499	0.00454624	yes
Rps23	chr13:90923121-90924732	11.5966	1.61759	-2.84178	0.000195236	yes
Kbtbd11	chr8:15011024-15033332	37.6819	5.27141	-2.83761	0.000195236	yes
2700094K13Rik	chr2:84669220-84670708	147.888	20.8065	-2.8294	0.000195236	yes
Crip1	chr12:113152011-113153879	356.784	50.2232	-2.82863	0.000195236	yes
Smad6	chr9:63953075-64022059	7.05174	0.993038	-2.82806	0.000195236	yes
Endog	chr2:30171523-30178459	21.9201	3.11073	-2.81693	0.00710851	yes
Rps14	chr18:60774595-60778546	151.601	22.2195	-2.77038	0.000195236	yes
H2afx	chr9:44334714-44336073	409.014	60.4509	-2.75832	0.000195236	yes

Lyz1	chr10:117287794-117292868	80.7321	12.033	-2.74614	0.000195236	yes
0610009L18Rik	chr11:120348677-120351190	1.30818	0.196172	-2.73737	0.0247412	yes
Ndrg4	chr8:95703036-95715119	19.552	2.94152	-2.73268	0.000195236	yes
Rps15a-ps6	chr11:6105691-6200451	26.3945	4.02928	-2.71165	0.0109347	yes
Trp53i11	chr2:93187583-93201757	0.573957	0.087878	-2.70737	0.000195236	yes
Hist1h2bj	chr13:22043229-22043658	386.131	59.4957	-2.69823	0.000195236	yes
Ang,Rnase4	chr14:51091076-51106151	7.68043	1.18855	-2.69198	0.000195236	yes
Rpl9	chr5:65388363-65391431	1.90927	0.295512	-2.69173	0.00116073	yes
Hist1h3i	chr13:21782914-21783397	1046.78	162.404	-2.6883	0.000195236	yes
Calml4	chr9:62838786-62875917	5.24721	0.817221	-2.68275	0.00271906	yes
Hist1h2bb	chr13:23746733-23747223	1308.63	204.467	-2.67812	0.000195236	yes
Fam69b	chr2:26628456-26636497	3.77905	0.591818	-2.6748	0.000195236	yes
Tufm	chr7:126487354-126490731	4.89053	0.770301	-2.6665	0.000195236	yes
Matk	chr10:81252934-81262981	0.67926	0.106997	-2.66639	0.000370691	yes
Clec4a2	chr6:123122689-123143999	9.79305	1.55061	-2.65892	0.000195236	yes
Dbp	chr7:45705246-45718002	6.77438	1.07623	-2.6541	0.00639665	yes
Adssl1	chr12:112620046-112641355	32.4147	5.16856	-2.64882	0.000195236	yes
Cfh	chr1:140085854-140183411	24.4554	3.90143	-2.64808	0.000195236	yes
Tk1	chr11:117815518-117839908	41.898	6.75019	-2.63388	0.000195236	yes
Fam64a	chr11:72042501-72047370	47.9445	7.81951	-2.61622	0.000195236	yes
Cxcl14	chr13:56288642-56296551	106.552	17.3784	-2.61619	0.000195236	yes
Prkar1b	chr5:139017303-139130386	1.28942	0.21254	-2.60092	0.000195236	yes
Timp2	chr11:118301060-118355411	0.460721	0.076005	-2.59973	0.000195236	yes
Hist1h4j	chr13:21735095-21735407	189.358	31.2397	-2.59967	0.000195236	yes
Snrpg	chr6:86371539-86378902	59.4567	9.86268	-2.59179	0.000195236	yes
Adgre4	chr17:55749983-55853662	0.786099	0.131364	-2.58114	0.000195236	yes
Kifc3	chr8:95081200-95142540	17.8046	3.0297	-2.555	0.000195236	yes
Fabp7	chr10:57784922-57788450	2.60151	0.444285	-2.54979	0.000195236	yes
Cenpa	chr5:30666885-30674837	97.4646	16.672	-2.54745	0.000195236	yes
Btbd17	chr11:114790668-114795892	0.449148	0.0770681	-2.54299	0.00950212	yes
Fgd2	chr17:29360913-29379535	11.903	2.06502	-2.5271	0.000195236	yes
Trem2	chr17:48346400-48352276	194.973	34.083	-2.51615	0.000195236	yes
Rpl37a	chr1:72711259-72713813	68.3895	11.957	-2.51592	0.000195236	yes
Rpl36	chr17:56613394-56614246	21.4982	3.76774	-2.51244	0.000195236	yes
Rps2	chr17:24720062-24721927	38.52	6.7832	-2.50557	0.000195236	yes
Rpl30	chr15:34440505-34443276	24.0933	4.26076	-2.49945	0.000195236	yes
Hist3h2ba	chr11:58948910-58949372	12.7011	2.2635	-2.48833	0.000195236	yes
Fam195a	chr17:25863697-25868738	19.73	3.53367	-2.48115	0.000195236	yes
Mgst3	chr1:167372383-167393797	101.006	18.1016	-2.48026	0.000195236	yes
Fam26f	chr10:34126066-34127972	1.38162	0.247813	-2.47903	0.00881201	yes
Pebp4	chr14:69840406-70059918	0.6341	0.113778	-2.47848	0.00390381	yes
Frat2	chr19:41845975-41848132	2.07166	0.37211	-2.47699	0.000195236	yes
Oxld1	chr11:120456603-120458063	9.35814	1.68777	-2.47111	0.000195236	yes
Rpl17	chr18:75000476-75003381	1.8281	0.336897	-2.43997	0.000195236	yes
Igfbp4	chr11:99041259-99052643	108.64	20.2417	-2.42415	0.000195236	yes
Eps8l2	chr7:141339001-141365110	0.487956	0.0909526	-2.42356	0.000195236	yes
Rpl35	chr2:38998308-39005131	9.81097	1.83574	-2.41803	0.000195236	yes
Hist1h2bf	chr13:23573759-23574190	89.1859	16.7739	-2.4106	0.000195236	yes
Kcna3	chr3:107036161-107038129	0.867	0.163758	-2.40446	0.000195236	yes
1810043H04Rik	chr11:120098933-120100424	43.2381	8.17395	-2.4032	0.000195236	yes
Gm14322	chr2:177759287-177773275	0.633397	0.120476	-2.39437	0.000195236	yes
Fam83d	chr2:158768098-158786637	10.3374	1.96815	-2.39296	0.000195236	yes
Rpl18	chr7:45718070-45720835	54.5418	10.4006	-2.3907	0.000195236	yes
Ahnak	chr19:8989283-9076926	164.356	31.3829	-2.38878	0.000195236	yes
D5Ertd605e	chr5:147418619-147423044	2.26153	0.431847	-2.38871	0.000195236	yes
C130050O18Rik	chr5:139359738-139460534	23.159	4.44353	-2.3818	0.000195236	yes
Npm3	chr19:45747733-45749563	20.3145	3.92516	-2.37168	0.000195236	yes
Ppp1cc	chr5:122158278-122175269	6.69604	1.29673	-2.36844	0.000195236	yes
Sh2d3c	chr2:32721054-32755007	2.14082	0.415936	-2.36373	0.000195236	yes
St6gal1	chr16:23224739-23360350	12.8892	2.51434	-2.35791	0.000195236	yes
Hmx2	chr7:131554061-131556582	7.25075	1.41678	-2.35551	0.000195236	yes
Cd101	chr3:100993528-101029495	0.637552	0.124744	-2.35357	0.000195236	yes
Slc16a12	chr19:34668405-34747111	44.9483	8.81455	-2.35031	0.000195236	yes
Tspan4	chr7:141475235-141539857	74.0497	14.5704	-2.34546	0.000195236	yes
Grcc10	chr6:124739183-124741079	143.716	28.4023	-2.33914	0.000195236	yes

Rac3	chr11:120721467-120723969	0.556898	0.110094	-2.33868	0.0203128	yes
Dgkg	chr16:22466568-22657231	3.79022	0.753623	-2.33037	0.000195236	yes
Ssbp4	chr8:70597489-70608314	60.6058	12.0522	-2.33016	0.000195236	yes
5031425F14Rik	chr2:166447450-166458770	0.638085	0.127582	-2.32233	0.00202512	yes
Col25a1	chr3:130180844-130599883	0.529618	0.106565	-2.31322	0.000195236	yes
Hist2h2ab	chr3:96219915-96220353	197.118	40.069	-2.2985	0.000195236	yes
Id3	chr4:136143821-136145392	96.3302	19.6019	-2.29699	0.000195236	yes
Sepp1	chr15:3270766-3280508	3.23321	0.659127	-2.29434	0.000195236	yes
Usp2	chr9:44067020-44095627	5.16126	1.05263	-2.29373	0.000195236	yes
Marveld1	chr19:42147388-42151703	12.4035	2.54598	-2.28446	0.000195236	yes
Cyp4f18	chr8:71988481-72009626	0.492324	0.101337	-2.28044	0.00847587	yes
Prdm13	chr4:21677479-21685963	0.658093	0.136108	-2.27354	0.000195236	yes
Phgdh	chr3:98313170-98339969	19.7082	4.07917	-2.27245	0.000195236	yes
Rprl2	chr3:22251369-22251607	3.64116	0.755151	-2.26956	0.0376194	yes
St8sia4	chr1:95587681-95667594	161.28	33.4786	-2.26825	0.000195236	yes
Ctsh	chr9:90054266-90076095	8.77973	1.83211	-2.26067	0.000195236	yes
Aspm	chr1:139454772-139494088	56.1967	11.7363	-2.25951	0.000195236	yes
Fam13c	chr10:70440667-70599291	6.28782	1.31605	-2.25634	0.000195236	yes
Sun2	chr15:79724067-79742536	12.7413	2.66743	-2.25599	0.000195236	yes
Bola1	chr3:96196587-96197586	36.2761	7.61116	-2.25283	0.000195236	yes
Prss46	chr9:110844505-110856522	2.32995	0.490764	-2.2472	0.000195236	yes
Batf3	chr1:191098413-191108943	1.82453	0.385923	-2.24114	0.000698636	yes
Fcna	chr2:25624666-25627974	9.06246	1.91974	-2.23899	0.000195236	yes
Rnaseh2c	chr19:5601872-5602959	56.4545	12.0404	-2.22921	0.000195236	yes
Hist1h4b	chr13:23756936-23757386	551.801	117.712	-2.22889	0.000195236	yes
Sapcd2	chr2:25372034-25378213	14.0833	3.01092	-2.22571	0.000195236	yes
Klf4	chr4:55527136-55532475	3.69471	0.792235	-2.22146	0.000195236	yes
Metrn	chr17:25794570-25797045	28.3757	6.11896	-2.2133	0.000195236	yes
Tfeb	chr17:47737036-47792416	9.8068	2.11684	-2.21187	0.000195236	yes
Nusap1	chr2:119609531-119650160	68.6355	14.8541	-2.20809	0.000195236	yes
Gpx1	chr9:108339079-108340344	748.555	162.289	-2.20554	0.000195236	yes
Map2k6	chr11:110399121-110513637	2.86793	0.622282	-2.20437	0.000195236	yes
Chst3	chr10:60181527-60219260	3.09248	0.671309	-2.20372	0.000195236	yes
Frat1	chr19:41829969-41832583	1.97379	0.429948	-2.19873	0.000195236	yes
Smpdl3a	chr10:57794543-57811830	8.87614	1.96143	-2.17803	0.000195236	yes
Spink5	chr18:43963240-44022487	10.0686	2.22509	-2.17793	0.000195236	yes
Ppm1j	chr3:104781055-104786017	9.12958	2.02009	-2.17613	0.000195236	yes
Nr0b2	chr4:133553389-133556536	7.11056	1.57743	-2.17239	0.000195236	yes
Ppp1r14b	chr19:6975047-6977324	103.983	23.1131	-2.16957	0.000195236	yes
Hist2h3b	chr3:96268653-96269155	599.09	133.175	-2.16945	0.000195236	yes
Cdc20	chr4:118428092-118437343	67.9517	15.1132	-2.1687	0.000195236	yes
Hist1h4h	chr13:23531043-23531478	857.37	190.737	-2.16834	0.000195236	yes
Agmo	chr12:37241638-37581932	11.6484	2.59241	-2.16777	0.000195236	yes
Kazald1	chr19:45076138-45079289	0.662963	0.147624	-2.167	0.011588	yes
Hist1h2ai	chr13:21716411-21716859	576.786	128.501	-2.16625	0.000195236	yes
Cenpm	chr15:82233775-82244747	21.2746	4.74721	-2.16398	0.000195236	yes
Uqcc2	chr17:27122664-27133891	134.573	30.1837	-2.15654	0.000195236	yes
Atg9b	chr5:24364818-24392143	6.27793	1.41347	-2.15105	0.000195236	yes
Alox5	chr6:116410070-116461178	14.5445	3.27532	-2.15077	0.000195236	yes
Six5	chr7:19094543-19098345	0.567178	0.12837	-2.14349	0.000537301	yes
Klc4	chr17:46630630-46645144	17.3895	3.93714	-2.14299	0.000195236	yes
Rpl37	chr15:5116612-5119140	10.9771	2.48621	-2.14247	0.000195236	yes
Spata5l1	chr2:122630624-122632704	11.9503	2.70674	-2.14242	0.000195236	yes
Mnd1	chr3:84087933-84155786	7.46872	1.69738	-2.13755	0.000195236	yes
Galr3,Gcat	chr15:79030873-79043558	25.025	5.69741	-2.13499	0.000195236	yes
Ndufa11	chr17:56717761-56724248	12.4286	2.83766	-2.13089	0.000195236	yes
Lsr	chr7:30957769-30973469	0.814132	0.186253	-2.128	0.000195236	yes
Lhfpl2	chr13:94057795-94195409	22.1046	5.06982	-2.12434	0.000195236	yes
Angptl2	chr2:33133418-33371494	81.1058	18.6553	-2.12022	0.000195236	yes
D830046C22Rik	chr5:139359738-139460534	3.77944	0.869982	-2.11911	0.0154682	yes
Pwwp2b	chr7:139248481-139267253	7.73157	1.78064	-2.11837	0.000195236	yes
Ptma	chr1:86526735-86530698	773.263	178.209	-2.11739	0.000195236	yes
Rgs2	chr1:143999337-144004149	31.3627	7.26033	-2.11094	0.000195236	yes
Bcl7c	chr7:127704977-127708766	9.10354	2.10977	-2.10934	0.000195236	yes
Nme3	chr17:24896499-24936977	24.8487	5.75999	-2.10903	0.00615568	yes

Pkd1l2	chr8:116995678-117082449	0.835231	0.193646	-2.10875	0.000195236	yes
B930041F14Rik	chr4:155694341-155696483	2.67521	0.620328	-2.10855	0.000195236	yes
Slc40a1	chr1:45908069-45925594	26.3317	6.11867	-2.10551	0.000195236	yes
Pif1	chr9:65587204-65595962	9.89603	2.30218	-2.10385	0.000195236	yes
Oit3	chr10:59422959-59441779	0.582613	0.136037	-2.09853	0.000195236	yes
Myb	chr10:21124929-21160984	3.04489	0.711202	-2.09806	0.000195236	yes
Fblim1	chr4:141576061-141606052	4.6012	1.07641	-2.09578	0.000195236	yes
Tsc22d3	chrX:140539528-140600522	8.50492	1.99525	-2.09173	0.000195236	yes
S100a3	chr3:90600214-90602702	1.38558	0.32553	-2.08963	0.00915655	yes
Arhgap15	chr2:43748823-44395953	9.39838	2.21351	-2.08608	0.000195236	yes
Hist1h2bc	chr13:23684198-23692480	336.597	79.5387	-2.08129	0.000195236	yes
Ech1	chr7:28825337-28832239	45.6924	10.8623	-2.07263	0.000195236	yes
2410127L17Rik	chr19:18670779-18704792	1.50055	0.357015	-2.07144	0.000195236	yes
Gas6	chr8:13465373-13494535	1.6251	0.387268	-2.06912	0.000195236	yes
Ankle1	chr8:71406011-71409904	8.67831	2.06848	-2.06884	0.000195236	yes
Angptl4	chr17:33774899-33781575	7.29019	1.74258	-2.06474	0.000195236	yes
Ptpn18	chr1:34459745-34473779	66.2739	15.8518	-2.0638	0.000195236	yes
Sbk1	chr7:126272618-126294999	2.29603	0.550763	-2.05964	0.000195236	yes
Tbxas1	chr6:38918985-39084579	2.58121	0.619968	-2.05778	0.000195236	yes
Mafb	chr2:160363676-160367065	3.2592	0.788131	-2.04801	0.000195236	yes
Igflr1	chr7:30565426-30567962	1.13117	0.273705	-2.04712	0.00416625	yes
Nudt14	chr12:112934732-112942118	19.3667	4.68648	-2.047	0.000195236	yes
Hpgds	chr6:65117292-65144730	22.374	5.41975	-2.04552	0.000195236	yes
Mir682	chr13:75645045-75645141	1135.22	276.606	-2.03707	0.000195236	yes
Rin1	chr19:5050807-5057071	1.60933	0.392467	-2.03582	0.000195236	yes
Tesc	chr5:118027823-118061870	0.957122	0.233533	-2.03508	0.00130921	yes
Thap3	chr4:151982637-151988986	11.3603	2.77557	-2.03314	0.000195236	yes
Emp2	chr16:10281748-10313968	1.76064	0.430709	-2.03132	0.000195236	yes
Bckdha	chr7:25629851-25658761	14.4509	3.53648	-2.03077	0.000195236	yes
AI504432	chr3:107039503-107054322	1.03777	0.254003	-2.03057	0.000195236	yes
Cx3cr1	chr9:120048682-120068296	26.4985	6.49066	-2.02947	0.000195236	yes
Ccnb2	chr9:70407688-70421554	153.399	37.6452	-2.02675	0.000195236	yes
Sirpb1a	chr3:15371826-15426427	0.558169	0.137022	-2.02629	0.0204073	yes
Susd3	chr13:49230830-49248706	45.3491	11.1419	-2.02507	0.000195236	yes
Hotairm1	chr6:52158523-52162020	4.73404	1.1648	-2.02299	0.0119065	yes
Plxdc1	chr11:97923236-97986446	1.1668	0.287471	-2.02106	0.000195236	yes
Atp5g1	chr11:96072792-96075694	25.2929	6.23711	-2.01978	0.000195236	yes
Rpl12	chr2:32961711-32964045	3.1462	0.782661	-2.00715	0.000195236	yes
Vaultrc5	chr18:36801762-36802107	7.23662	1.80087	-2.00662	0.000195236	yes
Ocstamp	chr2:165395449-165400394	6.20507	1.54645	-2.00448	0.000195236	yes
Myo1f	chr17:33555706-33607764	44.768	11.2324	-1.9948	0.000195236	yes
Hist1h4f	chr13:23551285-23551643	604.719	151.942	-1.99275	0.000195236	yes
Mmp8	chr9:7558428-7568486	27.5194	6.91622	-1.99239	0.000195236	yes
Ccnb1	chr13:100778738-100786486	57.172	14.42	-1.98724	0.000195236	yes
L3hypdh	chr12:72073427-72085313	17.4973	4.42147	-1.98454	0.000195236	yes
Hist1h3a	chr13:23761884-23762386	545.727	137.928	-1.98426	0.000195236	yes
2010300C02Rik	chr1:37611675-37719811	0.913138	0.230989	-1.98301	0.000195236	yes
Ankrd34a	chr3:96596635-96599778	1.48625	0.376861	-1.97957	0.000195236	yes
Mtus1	chr8:40990911-41133726	0.577682	0.14683	-1.97613	0.000195236	yes
Clec7a	chr6:129461590-129472779	20.3465	5.17371	-1.97551	0.000195236	yes
Dner	chr1:84369838-84696221	8.54231	2.17429	-1.97408	0.000195236	yes
Zfp524	chr7:5015507-5018488	5.73689	1.46024	-1.97406	0.000195236	yes
Tmem160	chr7:16452778-16455490	39.1257	9.97106	-1.9723	0.000195236	yes
Rab26	chr17:24529053-24533747	0.931285	0.238067	-1.96786	0.000195236	yes
1110001J03Rik	chr6:38534860-38539449	38.4433	9.87445	-1.96096	0.000195236	yes
Klk8	chr7:43797576-43803822	2.0299	0.521664	-1.96022	0.000195236	yes
Frmd4b	chr6:97286866-97617657	31.1276	8.01455	-1.9575	0.000195236	yes
Cytip	chr2:58129138-58160122	2.82056	0.726599	-1.95675	0.000195236	yes
Cela1	chr15:100674421-100687920	2.0817	0.536896	-1.95505	0.000195236	yes
Rps21	chr2:180257378-180258444	153.368	39.5883	-1.95385	0.000195236	yes
Gm15421	chr5:22486488-22550331	69.2587	17.8998	-1.95205	0.00160026	yes
Nme2	chr11:93949813-93956256	150.799	38.9843	-1.95166	0.000195236	yes
Pdgfa	chr5:138976971-138994953	14.2466	3.68356	-1.95144	0.000195236	yes
Uqcr11	chr10:80402996-80406821	87.8638	22.7522	-1.94927	0.000195236	yes
Depdc1b	chr13:108316336-108389557	10.33	2.67718	-1.94806	0.000195236	yes

Ydjc	chr16:17139063-17148857	45.3174	11.7814	-1.94355	0.000195236	yes
Pgls	chr8:71592183-71596267	119.172	30.9887	-1.94323	0.000195236	yes
Iqgap3	chr3:88082050-88121048	11.047	2.8849	-1.93706	0.000195236	yes
Fancd2os	chr6:113596761-113600715	1.09447	0.285985	-1.93622	0.000195236	yes
Rilp	chr11:75510093-75513166	0.477132	0.124684	-1.93611	0.00492649	yes
Rpl34	chr3:130726826-130730398	103.556	27.1985	-1.92881	0.000195236	yes
Hist1h1d	chr13:23555031-23555807	749.078	196.985	-1.92703	0.000195236	yes
Cst3	chr2:148871731-148875468	2527.26	664.926	-1.92631	0.000195236	yes
Rabac1	chr7:24969749-24972728	46.4653	12.3029	-1.91715	0.000195236	yes
3830432H09Rik	chr1:119529479-119536165	0.738844	0.195664	-1.9169	0.000370691	yes
Pmf1	chr3:88394142-88410316	69.2486	18.4101	-1.91129	0.000195236	yes
Trib2	chr12:15791726-15816785	0.651003	0.17308	-1.91122	0.000195236	yes
Ube2c	chr2:164769928-164772902	184.722	49.1436	-1.91028	0.000195236	yes
Hsd3b7	chr7:127800608-127803802	7.95444	2.11941	-1.9081	0.000195236	yes
Adamts10	chr17:33524195-33553782	5.26403	1.40486	-1.90574	0.000195236	yes
Tmem256	chr11:69838524-69839558	38.9193	10.4297	-1.89979	0.000195236	yes
Nr2f6	chr8:71374118-71381952	17.8395	4.81262	-1.89018	0.000195236	yes
Pkn3	chr2:30078765-30093635	14.4421	3.8964	-1.89006	0.000195236	yes
C1ql1	chr11:102939263-102946461	0.525291	0.141814	-1.88912	0.0080155	yes
Macrod1	chr19:7056767-7198062	4.30723	1.16307	-1.88882	0.000195236	yes
Phyhd1	chr2:30266202-30282149	3.481	0.942596	-1.88479	0.000195236	yes
Tuba1b	chr15:98931430-98934390	148.679	40.3136	-1.88286	0.000195236	yes
Ank	chr15:27466676-27594907	295.85	80.2235	-1.88277	0.000195236	yes
4930461G14Rik	chr9:58455172-58469623	4.13788	1.12441	-1.87972	0.000195236	yes
Itgb5	chr16:33829664-33949338	22.3455	6.0909	-1.87526	0.000195236	yes
Slc2a5	chr4:150119343-150144168	0.520139	0.141891	-1.87411	0.000370691	yes
Hfe	chr13:23703840-23710811	7.06777	1.92937	-1.87313	0.000195236	yes
M1ap	chr6:82946921-83030309	3.1589	0.865817	-1.86729	0.000195236	yes
Scrn2	chr11:97029951-97033960	3.8371	1.05242	-1.86631	0.000195236	yes
Mblac1	chr5:138194313-138195621	3.44123	0.945336	-1.86402	0.000195236	yes
Retsat	chr6:72598627-72607488	51.4137	14.1486	-1.86149	0.000195236	yes
Ndufs8	chr19:3908862-3912774	167.662	46.1636	-1.86073	0.000195236	yes
Mgst2	chr3:51661192-51682675	32.7257	9.02811	-1.85793	0.000195236	yes
Hexb	chr13:97137936-97198357	157.268	43.4336	-1.85634	0.000195236	yes
Mrpl36	chr13:73331008-73332178	63.312	17.5183	-1.85362	0.000195236	yes
4833418N02Rik	chr17:87274885-87282814	0.528648	0.146444	-1.85196	0.0169236	yes
Ctsf	chr19:4855128-4860912	16.8255	4.66996	-1.84917	0.000195236	yes
Rps8	chr4:117153835-117156132	189.832	52.6894	-1.84914	0.000195236	yes
9030617O03Rik	chr12:100779122-100872610	22.7044	6.30261	-1.84895	0.000195236	yes
Ndufs5	chr4:123712709-123718186	13.3557	3.71267	-1.84693	0.000195236	yes
Hist1h3f	chr13:23544051-23544954	196.276	54.579	-1.84647	0.000195236	yes
9930012K11Rik	chr14:70154404-70159502	2.32926	0.650011	-1.84134	0.000195236	yes
Ntpcr	chr8:125734202-125748235	11.2327	3.14354	-1.83723	0.000195236	yes
Hist1h2ao	chr13:21810366-21810918	2.75165	0.771588	-1.8344	0.00553759	yes
Ptgs1	chr2:36230425-36252271	45.75	12.846	-1.83246	0.000195236	yes
Atp5e	chr2:174461074-174464101	327.087	92.0584	-1.82905	0.000195236	yes
Kcnn4	chr7:24370262-24385212	76.1971	21.4486	-1.82885	0.000195236	yes
Kif22	chr7:127027730-127042420	43.5173	12.2664	-1.82687	0.000195236	yes
Uqcrq	chr11:53428947-53430831	292.767	82.5409	-1.82657	0.000195236	yes
Rnaset2b	chr17:8128590-8147832	6.85553	1.93361	-1.82597	0.000195236	yes
Pax6	chr2:105536079-105904564	7.73298	2.18388	-1.82413	0.000195236	yes
Sec16b	chr1:157506795-157568424	0.838448	0.236954	-1.82311	0.000195236	yes
Notch4	chr17:34564294-34588543	0.896018	0.253466	-1.82173	0.000195236	yes
Svip	chr7:51997160-52006018	1.06914	0.302981	-1.81915	0.000195236	yes
Sigirr	chr7:141091174-141100546	2.92661	0.829411	-1.81907	0.000195236	yes
Acaa2	chr18:74779211-74806207	31.4487	8.91611	-1.81851	0.000195236	yes
Ankrd13d	chr19:4270179-4283137	0.967301	0.274534	-1.81698	0.000195236	yes
Ndrg2	chr14:51905270-51913488	128.247	36.5049	-1.81276	0.000195236	yes
Hist2h2ac	chr3:96220412-96220880	1715.36	489.25	-1.80987	0.000195236	yes
Zfp771	chr7:127244525-127254801	20.2927	5.79757	-1.80744	0.000195236	yes
Rprl3	chr8:3803124-3803361	2725.1	780.196	-1.8044	0.000195236	yes
Slc13a3	chr2:165405294-165473197	0.540246	0.154929	-1.80201	0.000195236	yes
Mbd3	chr10:80392538-80399531	61.1015	17.5351	-1.80097	0.000195236	yes
Rasgrp3	chr17:75435904-75529053	92.6859	26.6317	-1.7992	0.000195236	yes
Rrad	chr8:104628065-104631321	4.7213	1.37312	-1.78172	0.000195236	yes

Cd84	chr1:171839696-171890718	0.782423	0.227839	-1.77993	0.000195236	yes
Acot1	chr12:84009501-84017669	1.58745	0.463385	-1.77642	0.000195236	yes
Naca	chr10:128035345-128048637	9.23133	2.69505	-1.77623	0.000195236	yes
Hist2h2bb	chr3:96269699-96270192	4760.62	1391	-1.77503	0.000195236	yes
Rps19	chr7:24884713-24889802	145.386	42.4977	-1.77444	0.000195236	yes
2610528A11Rik	chr14:37102139-37110101	1.2767	0.373903	-1.77168	0.00602767	yes
Rassf3	chr10:121410350-121476250	21.8329	6.3948	-1.77153	0.000195236	yes
Myoz1	chr14:20649101-20656540	4.15126	1.22143	-1.76497	0.000195236	yes
Pop7	chr5:137501438-137502429	23.1592	6.82039	-1.76366	0.000195236	yes
AI413582	chr17:27563768-27565727	37.5821	11.08	-1.76209	0.000195236	yes
Cd109	chr9:78615545-78716260	6.46205	1.90567	-1.7617	0.000195236	yes
Avpi1	chr19:42123274-42128993	33.7993	9.98158	-1.75965	0.000195236	yes
Mtfp1	chr11:4091480-4095431	11.1737	3.30394	-1.75785	0.000195236	yes
Rps27a	chr11:29545841-29578352	16.0676	4.75151	-1.7577	0.000195236	yes
Rps4x	chrX:102184942-102188371	325.761	96.5063	-1.75512	0.000195236	yes
Tnfrsf12a	chr17:23660522-23677449	12.7487	3.77873	-1.75438	0.0100707	yes
Cbr3	chr16:93683218-93690991	4.87777	1.44865	-1.75152	0.000195236	yes
1810011H11Rik	chr14:32785962-32817968	12.9671	3.85208	-1.75114	0.000195236	yes
Trappc6a	chr7:19508728-19516145	36.2999	10.7845	-1.75101	0.000195236	yes
Lrfn4	chr19:4510471-4621752	9.52394	2.83595	-1.74773	0.000195236	yes
Phlda3	chr1:135766084-135769134	2.42355	0.722795	-1.74546	0.000195236	yes
Bbc3	chr7:16309582-16318334	3.04063	0.908733	-1.74244	0.000195236	yes
Ccna2	chr3:36552605-36571996	215.165	64.5202	-1.73762	0.000195236	yes
Cox7a1	chr7:30184170-30186030	9.73276	2.91871	-1.73752	0.000195236	yes
Atp5k	chr5:108433252-108434378	228.113	68.4235	-1.73718	0.000195236	yes
Kif18a	chr2:109280737-109341746	30.0301	9.0116	-1.73655	0.000195236	yes
Pram1	chr17:33638055-33645706	1.19987	0.361191	-1.73204	0.000195236	yes
Rmrp	chr4:43492784-43493059	33897.8	10205.8	-1.73181	0.000195236	yes
Rgl2	chr17:33929893-33937687	18.2676	5.50306	-1.73098	0.000195236	yes
Bloc1s1	chr10:128919913-128923524	23.3046	7.02247	-1.73056	0.000195236	yes
Amhr2	chr15:102445366-102454639	0.92863	0.279949	-1.72994	0.000195236	yes
Rpl15	chr14:18267822-18270986	33.0273	9.96193	-1.72916	0.000195236	yes
Crnde	chr8:92326030-92356120	3.47601	1.04945	-1.72779	0.000195236	yes
Cdkn3	chr14:46760540-46771525	28.024	8.46834	-1.72651	0.000195236	yes
Cebpa	chr7:35119292-35121931	8.84877	2.67409	-1.72643	0.000195236	yes
Hint2	chr4:43654226-43656445	21.3864	6.46552	-1.72585	0.000195236	yes
Hist1h2an	chr13:21786771-21787218	290.948	88.0552	-1.72428	0.000195236	yes
Angpt2	chr8:18595172-18803188	4.77216	1.44435	-1.72423	0.000370691	yes
Qpct	chr17:79051905-79090243	3.13582	0.949634	-1.7234	0.000195236	yes
Selm	chr11:3514701-3517351	12.5823	3.82079	-1.71945	0.000195236	yes
Gm10069	chr6:128438756-128526720	1.55223	0.471809	-1.71807	0.000195236	yes
Ckb	chr12:111669354-111672338	93.1458	28.3225	-1.71754	0.000195236	yes
Mcf2l	chr8:12915892-13020509	2.45004	0.746266	-1.71504	0.000195236	yes
Lsm7	chr10:80852824-80855209	2.06453	0.629676	-1.71313	0.00504708	yes
Knstrn	chr2:118814002-118836212	53.1361	16.2076	-1.71302	0.000195236	yes
Acad12	chr5:121598280-121618938	0.508658	0.155186	-1.7127	0.00285786	yes
Actr3b	chr5:25760025-25850341	1.08245	0.33043	-1.71188	0.000195236	yes
Khk	chr5:30921894-30931246	13.5526	4.14063	-1.71065	0.000195236	yes
Ppp2r4	chr2:30416049-30447807	71.3897	21.8277	-1.70955	0.000195236	yes
Ube2i	chr17:25260510-25275528	11.9246	3.6489	-1.7084	0.000195236	yes
Cdc25c	chr18:34732994-34751533	14.2007	4.34993	-1.7069	0.000195236	yes
Cox5b	chr1:36691486-36693388	78.4026	24.024	-1.70643	0.000195236	yes
Uap1l1	chr2:25361491-25365626	37.4359	11.4772	-1.70566	0.000195236	yes
Rps18	chr17:33951998-33955641	35.3161	10.8303	-1.70525	0.000195236	yes
Ttc7	chr17:87282885-87381770	24.5415	7.52905	-1.70469	0.000195236	yes
Trim65	chr11:116124707-116131128	3.07329	0.945679	-1.70036	0.000195236	yes
Sytl2	chr7:90302354-90410719	0.58345	0.179905	-1.69737	0.000195236	yes
Ndufb8	chr19:44550253-44555415	287.492	88.9897	-1.69181	0.000195236	yes
Sac3d1	chr19:6116003-6118586	17.667	5.46993	-1.69146	0.000195236	yes
Shcbp1l	chr1:153425208-153452574	2.55886	0.793173	-1.68979	0.000195236	yes
Serpinb1c	chr13:32881396-32898140	1.51375	0.469565	-1.68873	0.000195236	yes
Syne3	chr12:104929932-105009809	22.9544	7.12499	-1.68781	0.000195236	yes
Hist1h3e	chr13:23561895-23562365	628.437	195.549	-1.68424	0.000195236	yes
Plxnd1	chr6:115954810-115995005	17.8221	5.54969	-1.68319	0.000195236	yes
Rpl10a	chr17:28328470-28331033	3.591	1.12226	-1.67798	0.000195236	yes

Mrps26	chr2:130563756-130565394	106.776	33.4111	-1.67619	0.000195236	yes
Rab26os	chr17:24528250-24528744	33.7846	10.5727	-1.67602	0.000195236	yes
Ube2cbp	chr9:86307233-86464916	3.50518	1.09831	-1.6742	0.000195236	yes
Efna1	chr3:89271729-89280951	1.65843	0.519988	-1.67327	0.000195236	yes
Ak1	chr2:32621757-32635058	2.3318	0.731394	-1.67273	0.000195236	yes
Cyb5rl	chr4:107070167-107084805	5.01849	1.57887	-1.66836	0.000195236	yes
Spag4	chr2:156065212-156069499	2.08706	0.658542	-1.66412	0.000195236	yes
Gpt	chr15:76696763-76699675	3.7509	1.18711	-1.65978	0.000195236	yes
Gm14403	chr2:177498225-177512311	2.32047	0.734435	-1.65971	0.000195236	yes
Anapc15	chr7:101881324-101899545	13.4077	4.24589	-1.65892	0.000195236	yes
Nrm	chr17:35861317-35865400	24.7018	7.86129	-1.65178	0.000195236	yes
Noxred1	chr12:87221122-87238601	0.542687	0.172813	-1.6509	0.0270102	yes
Cenpf	chr1:189640613-189688086	36.6187	11.6719	-1.64954	0.000195236	yes
Myom1	chr17:71019556-71126856	2.08907	0.665924	-1.64944	0.000195236	yes
Rpp25l	chr4:41712032-41713517	29.7813	9.51221	-1.64655	0.000195236	yes
Cdkn2c	chr4:109660875-109666756	36.5912	11.7077	-1.64404	0.000195236	yes
Pgp	chr17:24470472-24471596	58.052	18.5801	-1.64359	0.000195236	yes
Lgals3	chr14:47373859-47386167	319.764	102.345	-1.64357	0.000195236	yes
Tmem9	chr1:136008218-136035030	9.74802	3.12617	-1.64071	0.000195236	yes
Pkig	chr2:163658385-163726158	40.6497	13.0664	-1.63738	0.000195236	yes
Tmem143	chr7:45897068-45917413	9.18399	2.95293	-1.63697	0.000195236	yes
Fhad1	chr4:141890622-142011651	0.657079	0.211452	-1.63574	0.000195236	yes
Card11	chr5:140872998-141000596	7.5585	2.43362	-1.63499	0.000195236	yes
Fgf13	chrX:59062145-59585572	1.43791	0.463251	-1.6341	0.000195236	yes
Nav2	chr7:48959072-49610088	1.95572	0.63009	-1.63407	0.000195236	yes
Pira6	chr7:4274142-4282928	1.12426	0.362654	-1.63231	0.000195236	yes
Gpnmb	chr6:49036517-49058182	83.6375	27.0017	-1.6311	0.000195236	yes
Rasal1	chr5:120648811-120679610	2.51944	0.815352	-1.62761	0.000195236	yes
Uqcr10	chr11:4701967-4704344	258.386	83.6284	-1.62746	0.000195236	yes
Racgap1	chr15:99620495-99651656	65.3534	21.1611	-1.62685	0.000195236	yes
Hcst	chr7:30417711-30419854	13.4562	4.3598	-1.62594	0.000195236	yes
Hist1h2ac	chr13:23683472-23683959	114.738	37.2877	-1.62157	0.000195236	yes
Miip	chr4:147860777-147868719	11.1612	3.63167	-1.61979	0.000195236	yes
Pgk1	chrX:106187099-106203699	18.6883	6.08271	-1.61935	0.000195236	yes
4930426L09Rik	chr2:18998318-18999804	1.24271	0.404812	-1.61816	0.000195236	yes
Hist1h3b	chr13:23752379-23752840	775.149	252.987	-1.61541	0.000195236	yes
Pald1	chr10:61319656-61383523	9.3891	3.06491	-1.61514	0.000195236	yes
Smpd2	chr10:41487171-41490340	3.92245	1.28207	-1.61328	0.000195236	yes
Oscp1	chr4:126058564-126089334	0.662887	0.216667	-1.61328	0.000370691	yes
Sesn1	chr10:41810573-41908436	22.758	7.45074	-1.61092	0.000195236	yes
Vash1	chr12:86678699-86695681	1.27827	0.4185	-1.61089	0.000195236	yes
Stard10	chr7:101321318-101346312	1.56116	0.512043	-1.60828	0.000195236	yes
Havcr2	chr11:46193848-46589232	11.585	3.80421	-1.60659	0.000195236	yes
Pitpnm3	chr11:72047527-72135889	1.03601	0.340456	-1.60549	0.000195236	yes
Cdca3	chr6:124830175-124833701	51.882	17.0595	-1.60466	0.000195236	yes
Exosc5	chr7:25659152-25668032	81.9642	26.9716	-1.60355	0.000195236	yes
Isoc2b	chr7:4844959-4866179	0.53197	0.175567	-1.59933	0.0283815	yes
Arhgdig	chr17:26199182-26201350	2.37801	0.785021	-1.59895	0.000195236	yes
Rpl13	chr8:123102349-123105242	117.23	38.7051	-1.59874	0.000195236	yes
Hist1h2bn	chr13:21754122-21754553	440.075	145.304	-1.59867	0.000195236	yes
Lbh	chr17:72918304-72941946	0.490853	0.162266	-1.59693	0.00285786	yes
AF251705	chr11:114996768-115001880	15.8995	5.25996	-1.59586	0.000195236	yes
Parpbp	chr10:88091071-88146941	20.7802	6.87502	-1.59578	0.000195236	yes
Klhl35	chr7:99466003-99474020	0.615576	0.203689	-1.59557	0.0232747	yes
Abhd15	chr11:77515116-77520628	3.14401	1.04042	-1.59543	0.000195236	yes
Msh5	chr17:35028604-35046745	1.48257	0.490788	-1.59493	0.000195236	yes
2010107E04Rik	chr12:111961375-111966977	297.318	98.5583	-1.59296	0.000195236	yes
Tspan32	chr7:143005045-143019485	1.82039	0.604556	-1.5903	0.000195236	yes
Aldh16a1	chr7:45141839-45160064	29.0123	9.64194	-1.58927	0.000195236	yes
Cep89	chr7:35397092-35438684	10.1262	3.36619	-1.58891	0.000195236	yes
H2afz	chr3:137864486-137866922	83.7176	27.8784	-1.58638	0.000195236	yes
Ten1	chr11:116171882-116215318	30.1507	10.0405	-1.58636	0.00216236	yes
Bmyc	chr2:25706878-25707719	25.015	8.33095	-1.58624	0.000195236	yes
Dctpp1	chr7:127256958-127260667	138.976	46.3096	-1.58545	0.000195236	yes
Abi3	chr11:95824499-95845731	4.08672	1.36262	-1.58456	0.00130921	yes

Ubald2	chr11:116434093-116439077	5.71182	1.90493	-1.58421	0.000195236	yes
Galk1	chr11:115974724-116012719	51.818	17.2822	-1.58417	0.000195236	yes
Zfp473	chr7:44731481-44748617	1.03389	0.345543	-1.58115	0.000195236	yes
Mgmt	chr7:136894610-137128188	10.4392	3.49214	-1.57983	0.000195236	yes
Gsg1	chr6:135237329-135254336	0.561033	0.187873	-1.57833	0.00627268	yes
Tomm40l	chr1:171213969-171226379	10.9147	3.66	-1.57636	0.000195236	yes
Adgrl1	chr8:83900097-83941954	3.57795	1.19989	-1.57622	0.000195236	yes
Lgmn	chr12:102394097-102439697	219.279	73.6526	-1.57396	0.000195236	yes
Lpin3	chr2:160880669-160906000	2.07608	0.697659	-1.57327	0.000195236	yes
Ndufc1	chr3:51405478-51408955	23.6394	7.95555	-1.57116	0.000195236	yes
Twist2	chr1:91801476-91848027	1.39912	0.471083	-1.57046	0.00100977	yes
Eldr	chr11:16934708-16951282	4.21385	1.4202	-1.56904	0.000195236	yes
Tbc1d16	chr11:119143042-119228499	10.2954	3.47057	-1.56876	0.000195236	yes
Gm6682	chr12:4782169-4783512	4.14619	1.39979	-1.56657	0.000195236	yes
Impdh2	chr9:108560500-108565566	1.51825	0.512828	-1.56587	0.000195236	yes
Fam174a	chr1:95313627-95335284	80.6142	27.2464	-1.56497	0.000195236	yes
Asb13	chr13:3634031-3651779	3.31095	1.11981	-1.564	0.000195236	yes
Prdx4	chrX:155323919-155338454	122.383	41.4664	-1.56139	0.000195236	yes
Tspo	chr15:83563572-83574203	72.3967	24.5471	-1.56037	0.000195236	yes
Acot2	chr12:83987860-83993875	2.65478	0.900879	-1.55919	0.000195236	yes
Kifc5b	chr17:26917090-26932579	6.87544	2.33461	-1.55827	0.000195236	yes
Tmem107	chr11:69070808-69073293	7.70875	2.61857	-1.55772	0.000195236	yes
Sec61b	chr4:47474660-47483233	68.0902	23.1305	-1.55765	0.000195236	yes
Tead3	chr17:28331672-28350805	0.508367	0.172897	-1.55596	0.000698636	yes
Cbx8	chr11:119038435-119040913	7.40655	2.52137	-1.55459	0.000195236	yes
Ntmt1	chr2:30807976-30823014	18.8594	6.43244	-1.55185	0.000195236	yes
Fdxr	chr11:115268024-115276969	11.2611	3.84095	-1.55181	0.000195236	yes
Zcwpw1	chr5:137787801-137836278	3.46441	1.18195	-1.55145	0.00744706	yes
Spag5	chr11:78301590-78322454	29.179	9.95742	-1.55109	0.000195236	yes
Ears2	chr7:122034161-122067086	2.50511	0.855614	-1.54984	0.000195236	yes
Kank2	chr9:21766772-21798546	2.8508	0.974612	-1.54846	0.000195236	yes
Zfp2	chr11:50898711-50916176	0.523877	0.179402	-1.54604	0.000698636	yes
Slc44a2	chr9:21320718-21355028	31.8874	10.9213	-1.54584	0.000195236	yes
Bub1b	chr2:118598210-118641592	101.977	34.9465	-1.54502	0.000195236	yes
Nutf2	chr8:105860633-105880401	4.59305	1.57528	-1.54384	0.000195236	yes
Aarsd1	chr11:101406839-101417433	54.5454	18.726	-1.54242	0.000195236	yes
Pvrl3	chr16:46394857-46496967	22.5316	7.75025	-1.53963	0.000195236	yes
6430531B16Rik	chr7:139972302-139978755	2.546	0.876192	-1.53892	0.000195236	yes
Fut7	chr2:25423241-25426373	3.61854	1.24688	-1.53709	0.000195236	yes
Rnf167	chr11:70647588-70651414	13.8883	4.79975	-1.53283	0.000195236	yes
Plk1	chr7:122159436-122169875	28.5727	9.89597	-1.52973	0.000195236	yes
Pafah1b3	chr7:25295048-25297955	16.9363	5.87034	-1.5286	0.000195236	yes
Llph	chr10:120227059-120232070	14.5832	5.06501	-1.52567	0.000195236	yes
Smpdl3b	chr4:132732965-132757171	43.8625	15.2429	-1.52485	0.000195236	yes
Enkd1	chr8:105703651-105708168	9.60294	3.33914	-1.524	0.000195236	yes
Fkbp7	chr2:76663033-76673098	2.27241	0.790252	-1.52384	0.000195236	yes
Mrps11	chr7:78783130-78792988	20.968	7.3005	-1.52212	0.000195236	yes
Atp5j2	chr5:145183705-145191592	178.113	62.0342	-1.52166	0.000195236	yes
Cenpn	chr8:116921739-116941503	26.5291	9.24148	-1.52138	0.000195236	yes
Lsp1	chr7:142460811-142516009	34.3142	11.9589	-1.52072	0.000195236	yes
Agap1	chr1:89454810-89895282	11.4207	3.99189	-1.51651	0.000195236	yes
Cd1d1	chr3:86995835-86999340	0.643513	0.224952	-1.51635	0.00416625	yes
1700025G04Rik	chr1:151884523-152090320	17.8137	6.22882	-1.51595	0.000195236	yes
Hebp1	chr6:135137518-135168215	4.6831	1.63767	-1.51581	0.000195236	yes
S100a4	chr3:90603769-90606045	3750.1	1312.24	-1.5149	0.000195236	yes
Tmsb15b1,Tmsb15b2,Tm	chrX:136954987-136976874	2.09227	0.732564	-1.51404	0.00733312	yes
Cks2	chr13:51645231-51650662	41.9568	14.6911	-1.51396	0.000195236	yes
Cxxc5	chr18:35829817-35861688	3.7868	1.3272	-1.51259	0.000195236	yes
Tmem205	chr9:21921008-21935872	31.243	10.9545	-1.51201	0.000195236	yes
Aurkb	chr11:69045642-69051662	61.8278	21.7109	-1.50984	0.000195236	yes
1110034G24Rik	chr2:132690282-132751055	2.97954	1.04835	-1.50697	0.000195236	yes
Trf	chr9:103208875-103230286	5.68564	2.00117	-1.50648	0.000195236	yes
Itga4	chr2:79255425-79428988	130.63	45.9788	-1.50645	0.000195236	yes
Fahd2a	chr2:127436214-127444565	1.32963	0.468309	-1.50549	0.000698636	yes
Arhgap24	chr5:102481390-102897937	6.52114	2.29858	-1.50438	0.000195236	yes

Cxcr3	chrX:101731534-101734147	7.84591	2.76603	-1.50412	0.000195236	yes
Calhm2	chr19:47132231-47138294	13.8562	4.88549	-1.50395	0.000195236	yes
Efcab11	chr12:99717530-99883442	6.60049	2.32763	-1.50371	0.000195236	yes
2310039H08Rik	chr17:46772634-46773407	22.6586	7.99146	-1.50353	0.000195236	yes
Ndufb11	chrX:20615325-20650905	67.6085	23.8924	-1.50065	0.00100977	yes
Selenbp1	chr3:94933082-94944758	1.34881	0.476753	-1.50038	0.000537301	yes
Ccdc106	chr7:5056151-5060784	9.56529	3.3814	-1.50019	0.000195236	yes
Gm12522	chr3:108364910-108383741	0.81824	0.289755	-1.49769	0.000370691	yes
Emp3	chr7:45918022-45921426	294.92	104.47	-1.49724	0.000195236	yes
Nhej1	chr1:74967345-75046639	7.5809	2.68759	-1.49606	0.000195236	yes
Ndufa2	chr18:36735069-36744587	134.492	47.7221	-1.49479	0.000195236	yes
Exoc3l4	chr12:111417429-111431680	2.10034	0.745371	-1.49459	0.000195236	yes
Peg12	chr7:62461870-62464510	4.1576	1.47574	-1.49431	0.000195236	yes
Pbk	chr14:65805910-65817822	107.129	38.0294	-1.49417	0.000195236	yes
Tigd5	chr15:75909734-75914535	0.838254	0.297664	-1.4937	0.000195236	yes
Cmbl	chr15:31568911-31590119	1.48386	0.526988	-1.49351	0.00160026	yes
Faap100	chr11:120369561-120378746	6.761	2.40327	-1.49223	0.000195236	yes
Dapk1	chr13:60601946-60763191	0.469851	0.167043	-1.49198	0.000195236	yes
Kifc1	chr17:33875665-33890633	16.0343	5.70179	-1.49168	0.000195236	yes
Hnrnpa3	chr2:75659258-75669407	4.94624	1.75902	-1.49156	0.000195236	yes
Taldo1	chr7:141392159-141402976	236.807	84.2198	-1.49148	0.000195236	yes
Mycn	chr12:12936092-12941836	132.14	47.1741	-1.486	0.000195236	yes
Zfp385a	chr15:103313894-103340086	1.25629	0.44916	-1.48387	0.000195236	yes
Cyc1	chr15:76343522-76345934	120.646	43.1484	-1.4834	0.000195236	yes
Nuf2	chr1:169497933-169531464	95.0526	34.027	-1.48205	0.000195236	yes
Coq6	chr12:84361967-84373796	17.9491	6.42626	-1.48186	0.000195236	yes
Ckap2	chr8:22168151-22185819	45.2231	16.2074	-1.48041	0.000195236	yes
Hist1h1e	chr13:23621776-23622558	717.34	257.672	-1.47712	0.000195236	yes
Hspa1l	chr17:34972702-34979228	0.839761	0.30202	-1.47533	0.000195236	yes
Ing4	chr6:125009237-125049853	13.4205	4.83092	-1.47407	0.000195236	yes
Ankrd10	chr8:11611580-11635757	30.02	10.8189	-1.47237	0.000195236	yes
B230312C02Rik	chr2:180370857-180401802	2.99104	1.07807	-1.47219	0.000195236	yes
Wdr34	chr2:30031557-30048879	13.2423	4.77498	-1.47159	0.000195236	yes
Hn1	chr11:115497352-115514370	134.727	48.6044	-1.47088	0.000195236	yes
Zfp273	chr13:67796593-67830985	0.684257	0.247175	-1.469	0.000537301	yes
Lsm11	chr11:45928268-45944935	3.51824	1.27211	-1.46763	0.000195236	yes
Slc9a9	chr9:94669891-95230452	1.5451	0.559134	-1.46644	0.000195236	yes
Gemin6	chr17:80224488-80228497	30.1629	10.943	-1.46276	0.000195236	yes
Rad51b	chr12:79297281-79814690	5.44531	1.97623	-1.46226	0.000195236	yes
Galnt12	chr4:47091952-47123042	1.34924	0.490453	-1.45996	0.000195236	yes
Vmac	chr17:56713931-56717699	1.67717	0.61059	-1.45776	0.000195236	yes
Lzts2	chr19:45015175-45045772	9.71966	3.54531	-1.455	0.000195236	yes
Lgals1	chr15:78926724-78930465	371.696	135.666	-1.45406	0.000195236	yes
Gins1	chr2:150909593-150931280	44.8491	16.377	-1.4534	0.000195236	yes
Rundc3a	chr11:102393402-102402939	0.921613	0.336609	-1.45309	0.000195236	yes
Hist4h4	chr6:136803992-136804431	2183.66	798.901	-1.45066	0.000195236	yes
Mri1	chr8:84250575-84257324	15.0239	5.49864	-1.45012	0.000195236	yes
Ndufb2	chr6:39592582-39599471	104.004	38.0945	-1.44898	0.000195236	yes
Snhg7	chr2:26637175-26640244	11.5787	4.24458	-1.44778	0.000195236	yes
D330023K18Rik	chr2:31151048-31152291	1.70689	0.626482	-1.44603	0.0217808	yes
4930432K21Rik	chr8:84148037-84172597	6.37355	2.343	-1.44374	0.000195236	yes
Nup37	chr10:88146991-88178395	80.9986	29.7785	-1.44363	0.000195236	yes
Cd36	chr5:17781689-17888959	35.7809	13.1548	-1.44361	0.000195236	yes
Cks1b	chr3:89415471-89418291	183.682	67.7111	-1.43975	0.000195236	yes
Aig1	chr10:13652708-13868830	19.4984	7.19103	-1.43909	0.000195236	yes
Ffar4	chr19:38097078-38114263	4.42389	1.63179	-1.43886	0.000195236	yes
Engase	chr11:118476959-118489198	2.9567	1.09069	-1.43875	0.000195236	yes
Plcb4	chr2:135741829-136013068	36.0001	13.284	-1.43831	0.000195236	yes
Tpgs1	chr10:79669409-79676126	20.9879	7.75126	-1.43706	0.000195236	yes
A930001A20Rik	chr3:14971200-15002727	0.813144	0.300331	-1.43695	0.0136535	yes
Ltc4s	chr11:50236471-50238471	7.28957	2.69312	-1.43656	0.000537301	yes
Hgh1	chr15:76368897-76371411	13.6056	5.02912	-1.43583	0.000195236	yes
Kif2c	chr4:117159632-117182624	35.7331	13.2141	-1.43518	0.000195236	yes
Zfp773	chr7:7130677-7136755	1.34531	0.497689	-1.43462	0.000195236	yes
2810025M15Rik	chr1:157135182-157420236	91.4867	33.8547	-1.43421	0.000195236	yes

Cadm1	chr9:47530351-47853385	107.29	39.7396	-1.43286	0.000195236	yes
Ptov1	chr7:44863067-44869788	12.7743	4.73289	-1.43245	0.000195236	yes
Gtf3a	chr5:146948656-146963797	48.4915	17.983	-1.4311	0.000195236	yes
Mir99ahg	chr16:77329327-77558428	5.23357	1.94251	-1.42987	0.000195236	yes
Cbr2	chr11:120729484-120732021	1.67191	0.620665	-1.42961	0.00285786	yes
Sulf2	chr2:166073898-166155683	3.55342	1.32131	-1.42724	0.000195236	yes
Kdsr	chr1:106720409-106759742	7.27025	2.70359	-1.42713	0.000195236	yes
Hspbp1	chr7:4660520-4684963	30.9766	11.5227	-1.4267	0.000195236	yes
Fcrl1	chr3:87376386-87392133	38.6101	14.372	-1.42572	0.000195236	yes
Kif11	chr19:37376402-37421859	91.662	34.1581	-1.4241	0.000195236	yes
E2f2	chr4:136172273-136196056	8.22428	3.06553	-1.42375	0.000195236	yes
Erf	chr7:25242559-25250758	13.7813	5.1374	-1.42361	0.000195236	yes
Rps17	chr7:81342732-81345234	138.302	51.5757	-1.42306	0.000195236	yes
Rgs3	chr4:62559846-62703019	0.672482	0.251053	-1.42151	0.000195236	yes
Rab34	chr11:78188426-78192193	14.6642	5.47669	-1.42092	0.000195236	yes
Lpar5	chr6:125067919-125082472	5.77306	2.15662	-1.42057	0.000195236	yes
Dhrs7b	chr11:60830630-60858423	29.2924	10.9545	-1.41901	0.000195236	yes
Klf2	chr8:72319061-72321654	4.79495	1.79524	-1.41734	0.000195236	yes
Nenf	chr1:191306796-191318118	29.9436	11.2229	-1.41581	0.000195236	yes
Hmmr	chr11:40701387-40733437	56.6419	21.233	-1.41556	0.000195236	yes
4930427A07Rik	chr12:113156420-113165458	5.45419	2.04726	-1.41367	0.000195236	yes
BC049715	chr6:136828842-136840557	1.69245	0.636575	-1.41071	0.0321137	yes
Snhg20	chr11:117076782-117078955	20.456	7.6953	-1.41048	0.000195236	yes
Ctxn1	chr8:4257645-4259274	0.469768	0.176857	-1.40936	0.0368888	yes
Cox14	chr15:99725617-99728136	46.338	17.4476	-1.40916	0.000195236	yes
Tmc6	chr11:117765984-117780683	4.49726	1.69339	-1.40913	0.000195236	yes
Plekho1	chr3:95988835-95995839	50.5224	19.0423	-1.40771	0.000195236	yes
Magef1	chr16:21331899-21333356	4.41783	1.66529	-1.40757	0.000195236	yes
Fzr1	chr10:81366878-81378370	29.7067	11.1994	-1.40737	0.000195236	yes
Pttg1	chr11:43420247-43426248	76.296	28.8155	-1.40476	0.000195236	yes
Timm8a1	chrX:134537257-134541629	7.52647	2.84298	-1.40457	0.000195236	yes
Hist1h2ao	chr13:21833023-21833575	1.76585	0.66705	-1.4045	0.0346427	yes
Pgam1	chr19:41911870-41918665	22.247	8.40589	-1.40414	0.000195236	yes
Zfp213	chr17:23550798-23565294	1.9425	0.734707	-1.40267	0.000195236	yes
Zfp41	chr15:75616683-75625300	4.36854	1.65315	-1.40194	0.000195236	yes
Kank3	chr17:33810522-33822914	1.1664	0.441516	-1.40153	0.000195236	yes
Agk	chr6:40325477-40396762	3.0619	1.15903	-1.40151	0.000195236	yes
Rgs10	chr7:128373624-128418172	123.949	46.9497	-1.40056	0.000195236	yes
Armc6	chr8:70220192-70263105	10.6211	4.0271	-1.39912	0.00377278	yes
Fam188b	chr6:55203382-55320222	0.604536	0.229685	-1.39617	0.00230418	yes
A930006K02Rik	chr16:91465103-91470123	0.904479	0.344392	-1.39303	0.0141985	yes
Atox1	chr11:55446642-55461138	141.271	53.8543	-1.39134	0.000195236	yes
Prss50	chr9:110857966-110864628	16.7657	6.39743	-1.38995	0.000195236	yes
Psmg3	chr5:139823593-139826843	40.7252	15.5567	-1.38839	0.000195236	yes
Cradd	chr10:95174745-95324097	2.25332	0.861092	-1.38781	0.000195236	yes
1700037C18Rik	chr16:3905797-3908689	2.60396	0.995688	-1.38694	0.000195236	yes
Mrps24	chr11:5703982-5707699	60.654	23.2099	-1.38586	0.000195236	yes
Spata33	chr8:123212857-123222045	1.14525	0.438313	-1.38563	0.0229541	yes
Ankdd1b	chr13:96416133-96471160	0.966109	0.370131	-1.38415	0.000195236	yes
Magix	chrX:7673165-7681251	0.672024	0.257581	-1.38349	0.000195236	yes
Pdxp	chr15:78913918-78919517	8.88392	3.40666	-1.38284	0.000195236	yes
Trim47	chr11:116105749-116110235	3.61165	1.38516	-1.38261	0.000195236	yes
Fam185a	chr5:21424902-21482124	15.5617	5.98003	-1.37978	0.000195236	yes
Rpph1	chr14:50807446-50807771	17118.1	6579.85	-1.3794	0.000195236	yes
Ttc5	chr14:50765408-50785520	11.6741	4.48752	-1.37932	0.000195236	yes
0610011F06Rik	chr17:25875499-25877163	12.7831	4.9144	-1.37916	0.000195236	yes
Acsf3	chr8:122775504-122817881	2.58413	0.994166	-1.37812	0.000195236	yes
Aars2	chr17:45506840-45520843	5.84145	2.24786	-1.37777	0.000195236	yes
2210408F21Rik	chr6:31220350-31337394	10.5	4.04383	-1.3766	0.000195236	yes
Anxa11	chr14:25842154-25886804	2.85692	1.10046	-1.37636	0.000195236	yes
Scand1	chr2:156311845-156375638	63.9897	24.6559	-1.37591	0.000195236	yes
Hmbs	chr9:44336347-44344228	27.8546	10.751	-1.37345	0.000195236	yes
Serpinb8	chr1:107590005-107608978	37.6541	14.5418	-1.3726	0.000195236	yes
Fbxl8	chr8:105264647-105269326	2.60799	1.00733	-1.3724	0.000195236	yes
Dbndd2	chr2:164486139-164493323	3.06399	1.18413	-1.37159	0.000195236	yes

2410016O06Rik	chr12:83950607-83952953	38.7252	14.9661	-1.37157	0.000195236	yes
Fv1	chr4:147868978-147870358	3.03303	1.17277	-1.37084	0.000195236	yes
Wdr8	chr4:154142371-154156818	12.647	4.89262	-1.37012	0.000195236	yes
Fhod1	chr8:105329159-105347970	2.36034	0.913547	-1.36944	0.000195236	yes
Kctd2	chr11:115420125-115431274	6.71574	2.60099	-1.36849	0.000195236	yes
Pnpla7	chr2:24976032-25054072	11.3467	4.39526	-1.36825	0.000195236	yes
Gsn	chr2:35256358-35307902	177.12	68.6294	-1.36783	0.000195236	yes
Cd33	chr7:43527455-43533171	2.56592	0.99442	-1.36755	0.000195236	yes
Prtg	chr9:72807273-72917307	5.4386	2.11147	-1.36499	0.000195236	yes
Shpk	chr11:73199481-73224506	0.782753	0.303926	-1.36484	0.000195236	yes
Kif18b	chr11:102905518-102925124	12.5348	4.86772	-1.36462	0.000195236	yes
Hist1h4d	chr13:23581601-23581969	1084.22	421.47	-1.36316	0.000195236	yes
Bcl6	chr16:23965051-23988612	12.1304	4.7219	-1.36119	0.000195236	yes
Ccnf	chr17:24223231-24251409	29.4085	11.4532	-1.36048	0.000195236	yes
Slc2a8	chr2:32972988-32982056	2.85113	1.11151	-1.35901	0.000195236	yes
Nmral1	chr16:4711317-4719356	47.4557	18.504	-1.35875	0.000195236	yes
Akr1b3	chr6:34303929-34317489	6.63737	2.59164	-1.35675	0.000195236	yes
Tcea2	chr2:181680309-181688051	1.93843	0.756987	-1.35655	0.000195236	yes
Camk1	chr6:113326975-113343922	47.7023	18.6309	-1.35636	0.000195236	yes
Zkscan4	chr13:21478848-21485505	0.62107	0.242967	-1.354	0.000370691	yes
Cxx1b	chrX:53669176-53670408	1.37051	0.536841	-1.35215	0.00298992	yes
Haus4	chr14:54541784-54554361	22.162	8.68196	-1.35199	0.000195236	yes
2410015M20Rik	chr17:56607451-56609771	83.4204	32.6949	-1.35134	0.000195236	yes
Itgb7	chr15:102215994-102231935	14.0923	5.52396	-1.35113	0.000195236	yes
Mpc2	chr1:165461207-165481214	54.1485	21.2312	-1.35074	0.000195236	yes
Qrsl1	chr10:43874189-43901736	11.9915	4.70689	-1.34916	0.000195236	yes
Fam110a	chr2:151969395-151980219	5.42519	2.13072	-1.34833	0.000195236	yes
Snx17	chr5:31193303-31198900	44.4541	17.4743	-1.34708	0.000195236	yes
Snx21	chr2:164786020-164804881	3.78893	1.4913	-1.34522	0.0100707	yes
Rnd3	chr2:51130438-51149111	4.36283	1.71894	-1.34375	0.000195236	yes
Gltscr2	chr7:15937835-15946108	48.1621	18.9776	-1.3436	0.000195236	yes
Egfl7	chr2:26581055-26592682	8.83917	3.48791	-1.34155	0.000195236	yes
Nt5dc2	chr14:31134852-31168641	32.5391	12.8403	-1.3415	0.000195236	yes
Sdhb	chr4:140961270-140979192	173.781	68.6362	-1.34023	0.000195236	yes
Fam129c	chr8:71597645-71608149	1.74205	0.689157	-1.33788	0.000195236	yes
2900026A02Rik	chr5:113086322-113163313	2.56467	1.01475	-1.33765	0.000195236	yes
Tmem141	chr2:25620065-25622005	26.5146	10.4922	-1.33747	0.000195236	yes
Coa6	chr8:126422500-126425435	12.341	4.90157	-1.33215	0.000195236	yes
Amica1	chr9:45079182-45135606	5.97695	2.37425	-1.33194	0.000195236	yes
A430046D13Rik	chr10:128499246-128504242	1.64695	0.654502	-1.33133	0.000195236	yes
Paox	chr7:140125684-140134334	0.538721	0.214163	-1.33083	0.0154682	yes
Tnni2	chr7:142442467-142444405	62.1047	24.6963	-1.33041	0.000195236	yes
Capg	chr6:72544390-72562983	528.955	210.546	-1.32901	0.000195236	yes
Dtymk	chr1:93792575-93801934	261.541	104.114	-1.32887	0.000195236	yes
Erp29	chr5:121444752-121452474	379.49	151.093	-1.32862	0.000195236	yes
Slc25a45	chr19:5878465-5885768	17.6183	7.01486	-1.32859	0.000195236	yes
Mdh1	chr11:21556691-21571934	171.629	68.3988	-1.32725	0.000195236	yes
Chchd10	chr10:75935572-75940672	20.6893	8.24771	-1.32682	0.000195236	yes
Tspan13	chr12:36014554-36042478	3.11003	1.23992	-1.32668	0.000195236	yes
Tlr13	chrX:106143274-106160493	14.7411	5.88587	-1.32451	0.000195236	yes
Fam131a	chr16:20695056-20716636	0.92327	0.368657	-1.32447	0.00271906	yes
Hmgb2	chr8:57511842-57515999	37.7066	15.0837	-1.32183	0.000195236	yes
2410006H16Rik	chr11:62602876-62604806	51.1146	20.4517	-1.32151	0.000195236	yes
Pars2	chr4:106651068-106655282	4.92349	1.97003	-1.32147	0.000195236	yes
Atp5o	chr16:91925222-91931630	129.523	51.8282	-1.3214	0.000195236	yes
Hmx3	chr7:131542962-131544931	0.861715	0.344986	-1.32067	0.00766958	yes
Neil3	chr8:53586866-53639065	16.0524	6.42693	-1.32059	0.000195236	yes
Adap1	chr5:139271875-139325464	46.6304	18.68	-1.31978	0.000195236	yes
Zfp775	chr6:48613179-48623227	1.07741	0.431618	-1.31974	0.000195236	yes
Pdcd2l	chr7:34184496-34196647	20.9326	8.386	-1.3197	0.000195236	yes
Snrpb	chr2:130171635-130179364	342.554	137.268	-1.31934	0.000195236	yes
Tmed2	chr5:124540790-124550503	19.7461	7.9167	-1.3186	0.000195236	yes
Hyi	chr4:118359998-118409263	17.0187	6.82564	-1.31808	0.00188078	yes
Arl4c	chr1:88698225-88702191	3.83673	1.53888	-1.318	0.000195236	yes
Id2	chr12:25093798-25096092	71.5203	28.6997	-1.31732	0.000195236	yes

Hist1h2be	chr13:23583669-23621124	467.421	187.704	-1.31626	0.000195236	yes
Pcif1	chr2:164879367-164891437	10.2953	4.13557	-1.31583	0.000195236	yes
Acaa1a	chr9:119341293-119350295	54.0117	21.75	-1.31225	0.000195236	yes
Emp1	chr6:135362930-135383173	91.6699	36.9535	-1.31074	0.000195236	yes
Abhd8	chr8:71456699-71463657	9.46833	3.81782	-1.31036	0.000195236	yes
Fancf	chr7:51860576-51862267	16.0101	6.4578	-1.30986	0.000195236	yes
Ndufab1	chr7:122088043-122101848	12.9877	5.25324	-1.30586	0.000195236	yes
Klhl17	chr4:156229043-156234857	6.82282	2.76296	-1.30415	0.000195236	yes
Abcb8	chr5:24394155-24409947	16.551	6.71066	-1.3024	0.000195236	yes
Espl1	chr15:102296292-102324356	11.052	4.48186	-1.30214	0.000195236	yes
Rpl19	chr11:98026709-98030493	41.8517	16.9738	-1.30198	0.000195236	yes
Rxrb	chr17:34031811-34038403	23.9318	9.7147	-1.30069	0.000195236	yes
Zranb3	chr1:127954178-128103047	6.44714	2.61777	-1.30032	0.000195236	yes
St6galnac4,St6galnac6	chr2:32587077-32620809	27.1907	11.0451	-1.2997	0.000195236	yes
Cdca2	chr14:67676353-67715841	35.4208	14.3948	-1.29905	0.000195236	yes
Snx29	chr16:11322903-11755473	5.56127	2.26137	-1.29822	0.000195236	yes
Cep72	chr13:74036494-74062285	3.08502	1.25508	-1.2975	0.000195236	yes
Ndc80	chr17:71496099-71526857	56.9307	23.178	-1.29645	0.000195236	yes
Ifi30	chr8:70762772-70766663	259.666	105.733	-1.29624	0.000195236	yes
Tsfm	chr10:127022331-127030814	41.3874	16.8576	-1.29579	0.000195236	yes
Gramd4	chr15:86057694-86137636	10.4849	4.27096	-1.29568	0.000195236	yes
Slc39a3	chr10:81028539-81033912	3.32002	1.35245	-1.29561	0.000195236	yes
D17H6S56E-5	chr17:34996723-35000746	103.827	42.3815	-1.29267	0.000195236	yes
Aif1	chr17:35170991-35176001	3.60651	1.47282	-1.29202	0.000195236	yes
Kdm8	chr7:125444619-125462268	3.41448	1.3947	-1.29171	0.000195236	yes
Hscb	chr5:110829069-110839777	21.8933	8.95121	-1.29033	0.000195236	yes
Hip1	chr5:135406518-135545122	10.0117	4.09448	-1.28993	0.000195236	yes
Epha2	chr4:141301220-141329384	1.0536	0.431268	-1.28867	0.000195236	yes
Ada	chr2:163726570-163750239	10.7893	4.41954	-1.28763	0.000195236	yes
Kif15	chr9:122951080-123018733	55.4227	22.7038	-1.28754	0.000195236	yes
Cdk2ap1	chr5:124345438-124354628	36.1639	14.8279	-1.28623	0.000195236	yes
Wbscr16	chr5:134148057-134176767	15.2595	6.26283	-1.28482	0.000195236	yes
Cc2d2a	chr5:43662378-43740970	1.37208	0.563575	-1.28369	0.000195236	yes
Rnf187	chr11:58932287-58938906	97.8292	40.207	-1.28282	0.000195236	yes
Rsrp1	chr4:134923624-134927370	75.5054	31.0324	-1.2828	0.000195236	yes
Ndufa7	chr17:33824571-33838316	129.904	53.4313	-1.28169	0.000195236	yes
Dlg4	chr11:70018604-70045531	1.25914	0.517985	-1.28146	0.000195236	yes
B130034C11Rik	chr16:87496072-87504038	1.13014	0.465042	-1.28106	0.00116073	yes
Ccdc51	chr9:109082495-109093363	8.57973	3.5308	-1.28094	0.000195236	yes
Nudt6	chr3:37404981-37419596	5.98351	2.46346	-1.2803	0.000195236	yes
Fam105a	chr15:27655070-27681542	37.4932	15.4376	-1.28018	0.000195236	yes
Abhd17a	chr10:80583648-80590341	52.0722	21.4411	-1.28014	0.000195236	yes
Fbxo31	chr8:121549442-121578806	4.9099	2.02358	-1.27878	0.000195236	yes
Sod1	chr16:90220741-90226324	207.657	85.5932	-1.27863	0.000195236	yes
Kif14	chr1:136467847-136530819	13.1908	5.43757	-1.2785	0.000195236	yes
Cdkn2d	chr9:21288463-21291209	32.843	13.5452	-1.27781	0.000195236	yes
Zbtb8os	chr4:129336025-129347029	10.0266	4.13552	-1.27769	0.000195236	yes
Ccdc85c	chr12:108206344-108275417	11.8858	4.90571	-1.2767	0.000195236	yes
Per3	chr4:151003654-151044665	0.893929	0.369024	-1.27645	0.000195236	yes
Ap1m2	chr9:21295456-21312333	0.443037	0.182913	-1.27627	0.0276926	yes
Mki67	chr7:135689787-135716379	161.366	66.6705	-1.27522	0.000195236	yes
Ehmt2	chr17:34898491-34914050	34.6672	14.33	-1.27453	0.000195236	yes
4933413G19Rik,Foxm1	chr6:128362993-128385144	19.3583	8.00404	-1.27415	0.000195236	yes
Sars2	chr7:28741967-28753879	9.764	4.03859	-1.27362	0.000195236	yes
Rfc5	chr5:117379144-117389023	40.8972	16.9398	-1.27158	0.000195236	yes
Samd1	chr8:83997671-84000386	45.9359	19.0398	-1.2706	0.000195236	yes
Ankrd55	chr13:112288450-112384002	1.72555	0.715739	-1.26955	0.000195236	yes
Tfdp2	chr9:96196274-96323646	6.88539	2.85863	-1.26822	0.000195236	yes
H2afv	chr11:6427225-6444443	24.4929	10.1752	-1.26731	0.000195236	yes
Rita1	chr5:120609059-120612589	3.45341	1.43489	-1.26708	0.000195236	yes
Hpdl	chr4:116819906-116821508	1.31043	0.544679	-1.26657	0.000537301	yes
Cenpw	chr10:30196008-30200540	17.387	7.23148	-1.26564	0.000195236	yes
Rcbtb2	chr14:73142509-73184054	11.3432	4.71986	-1.26501	0.000195236	yes
Plau	chr14:20836661-20843388	77.6066	32.2992	-1.26468	0.000195236	yes
Rap1gap	chr4:137664725-137729861	0.669533	0.278881	-1.26351	0.000195236	yes

Kif20a	chr18:34598614-34651736	92.4561	38.5815	-1.26086	0.000195236	yes
Phb	chr11:95666956-95680773	28.536	11.9131	-1.26024	0.000195236	yes
Ddx5	chr11:106780355-106788494	756.076	315.7	-1.25998	0.000195236	yes
Adprhl2	chr4:126316350-126321703	21.1525	8.83268	-1.2599	0.000195236	yes
Hmg20b	chr10:81346045-81350457	16.4895	6.88958	-1.25906	0.000195236	yes
Ly86	chr13:37345344-37419036	161.421	67.4992	-1.25788	0.000195236	yes
Fam173a	chr17:25786579-25792394	31.093	13.0117	-1.25678	0.000195236	yes
Plekhg5	chr4:152086862-152115404	1.9295	0.808619	-1.25469	0.000195236	yes
Gm14305	chr2:176708352-176721813	2.90461	1.21739	-1.25456	0.000195236	yes
B230217C12Rik	chr11:97840779-97843043	5.00872	2.09949	-1.2544	0.000195236	yes
Zfp931	chr2:178066877-178078425	5.85934	2.46075	-1.25164	0.000195236	yes
Wdpcp	chr11:21572280-21898686	4.99182	2.0967	-1.25144	0.000195236	yes
Psmg4	chr13:34162963-34178172	71.1943	29.9046	-1.25139	0.000195236	yes
Cdpf1	chr15:85806971-85811697	10.9286	4.59158	-1.25104	0.000195236	yes
Tbc1d8	chr1:39367850-39478747	3.36602	1.4144	-1.25086	0.00244513	yes
Prss12	chr3:123446912-123506602	1.82615	0.767367	-1.25082	0.000195236	yes
Nek2	chr1:191821472-191833049	51.6965	21.7315	-1.25028	0.000195236	yes
Nfe2l3	chr6:51432669-51458768	1.35184	0.568521	-1.24964	0.000195236	yes
Yars2	chr16:16302964-16309640	40.7818	17.1539	-1.24939	0.000195236	yes
Fanci	chr7:79392337-79466273	13.6965	5.76156	-1.24927	0.000195236	yes
Dnase2a	chr8:84908623-84911461	40.3978	16.9988	-1.24884	0.000195236	yes
Tyrobp	chr7:30413787-30417579	857.078	360.673	-1.24873	0.000195236	yes
Zbtb12	chr17:34894558-34896844	8.94624	3.76666	-1.24799	0.000195236	yes
Snrpd2	chr7:19149837-19152726	122.089	51.4071	-1.2479	0.000195236	yes
Tmem51	chr4:142030992-142088101	10.7775	4.5393	-1.24749	0.000195236	yes
Rpl26	chr11:68901565-68904534	55.821	23.5201	-1.24691	0.000195236	yes
Dnph1	chr17:46496788-46499618	59.4213	25.0499	-1.24617	0.000195236	yes
Efemp2	chr19:5474689-5481854	3.21961	1.35757	-1.24586	0.000195236	yes
Zfp219	chr14:51984832-52020733	6.75013	2.84675	-1.2456	0.00244513	yes
Best1	chr19:9985171-10001633	1.06373	0.449289	-1.24342	0.000855787	yes
Dpp7	chr2:25352289-25356332	18.3873	7.76779	-1.24314	0.000195236	yes
Ephx1	chr1:180989555-181017495	5.15053	2.1788	-1.24119	0.000195236	yes
Scarna13	chr12:105030616-105032279	1215.28	514.448	-1.2402	0.000195236	yes
Ppcdc	chr9:57412659-57440114	11.3115	4.79003	-1.23969	0.000195236	yes
Man2a2	chr7:80349096-80371375	12.0092	5.08687	-1.23928	0.000195236	yes
2610507I01Rik	chr11:59197792-59202431	1.17344	0.497369	-1.23835	0.000195236	yes
Gm17745	chr10:93336231-93348879	3.56711	1.51353	-1.23684	0.000195236	yes
Sdhaf4	chr1:23995938-24005640	30.429	12.9127	-1.23665	0.000195236	yes
Fam19a3	chr3:104767404-104777547	1.22237	0.518811	-1.2364	0.000195236	yes
Slc16a7	chr10:125227484-125328535	5.81052	2.46749	-1.23562	0.000195236	yes
Ddx39	chr8:83715176-83741311	164.932	70.0753	-1.23489	0.000195236	yes
Lamtor4	chr5:138255481-138259395	32.4469	13.7944	-1.23399	0.000195236	yes
Zfp692	chr11:58307068-58314613	7.07582	3.00861	-1.2338	0.000195236	yes
Dcaf15	chr8:84097071-84104762	8.24225	3.50914	-1.23192	0.000195236	yes
Polr1c	chr17:46243919-46248045	44.9914	19.1616	-1.23143	0.000195236	yes
Steap3	chr1:120226415-120271082	11.3981	4.85569	-1.23105	0.000195236	yes
Tfap4	chr16:4544660-4559720	14.799	6.31201	-1.22933	0.000195236	yes
Stx2	chr5:128984557-129008572	16.3849	6.99119	-1.22875	0.000195236	yes
Cars2	chr8:11514016-11550771	16.8589	7.19412	-1.22862	0.000195236	yes
Bphl	chr13:34037640-34074074	8.8005	3.7559	-1.22843	0.000195236	yes
Zfp1	chr8:111643442-111671011	4.89553	2.08952	-1.22829	0.000195236	yes
Naa10	chrX:73916869-73921944	67.1453	28.6675	-1.22787	0.000195236	yes
Slc24a3	chr2:145242610-145641939	0.609598	0.260301	-1.22768	0.000370691	yes
Polr2f	chr15:79141366-79151767	120.756	51.5701	-1.22749	0.000195236	yes
Slc25a26	chr6:94500313-94700145	5.55927	2.37535	-1.22675	0.00188078	yes
Gmpr	chr13:45507443-45546386	30.1186	12.8805	-1.22547	0.000195236	yes
Ndufa4	chr6:11900372-11907446	286.838	122.69	-1.22521	0.000195236	yes
Colec12	chr18:9707647-9877995	186.534	79.8237	-1.22455	0.000195236	yes
N4bp3	chr11:51643088-51657681	3.54898	1.521	-1.22239	0.000195236	yes
Pik3r2	chr8:70768180-70776712	14.861	6.37059	-1.22203	0.000195236	yes
Hyls1	chr9:35559465-35570069	17.7116	7.59354	-1.22185	0.00202512	yes
Hist1h1a	chr13:23763667-23764412	855.641	367.202	-1.22043	0.000195236	yes
Rpusd3	chr6:113415318-113419340	2.73978	1.17689	-1.21909	0.000195236	yes
Tmsb4x	chrX:167207093-167209218	1420.58	610.325	-1.21883	0.000195236	yes
Cerk	chr15:86139100-86186141	22.5128	9.68507	-1.21691	0.000195236	yes

Ppapdc3	chr2:32095650-32110820	2.19369	0.943889	-1.21667	0.000195236	yes
Hmgb1	chr5:149047226-149053037	20.3978	8.78372	-1.21551	0.000195236	yes
Hsd17b10	chrX:152001895-152004442	87.075	37.5102	-1.21498	0.000195236	yes
Cenpp	chr13:49464058-49652731	24.4885	10.5492	-1.21497	0.000195236	yes
1810043G02Rik	chr10:77978649-77985438	4.43892	1.9127	-1.2146	0.000195236	yes
Cuedc2	chr19:46328183-46338660	50.7041	21.8534	-1.21425	0.000195236	yes
Cd300a	chr11:114890040-114904651	20.6883	8.91822	-1.21399	0.000195236	yes
Itpa	chr2:130667840-130681614	15.5178	6.69756	-1.21222	0.000195236	yes
Saysd1	chr14:20075635-20083172	5.17919	2.23663	-1.2114	0.000195236	yes
Mnd1-ps	chr14:9550093-11162035	4.72818	2.04458	-1.20948	0.000195236	yes
Lair1	chr7:4007072-4063204	9.05874	3.91881	-1.2089	0.000195236	yes
Mettl20	chr6:149141512-149151170	2.39659	1.03698	-1.2086	0.000195236	yes
Sssca1	chr19:5730305-5731732	55.0074	23.823	-1.20727	0.000195236	yes
Slc6a8	chrX:73673132-73682500	5.23624	2.26864	-1.20671	0.000195236	yes
Alpl	chr4:137741730-137796384	2.91262	1.26254	-1.20598	0.000195236	yes
Cdkn2b	chr4:89306288-89311032	1.13785	0.493521	-1.20512	0.00403597	yes
Etohi1	chr2:178023283-178035859	24.1474	10.5096	-1.20016	0.000195236	yes
Rpl27	chr11:101442244-101445596	1.36027	0.59249	-1.19903	0.0140893	yes
Sirt5	chr13:43370715-43395203	4.94491	2.1551	-1.19819	0.000195236	yes
Hist1h2af	chr13:23533910-23534378	102.53	44.7213	-1.197	0.000195236	yes
Gnb2	chr5:137528128-137533229	120.86	52.7422	-1.19631	0.000195236	yes
Dmpk	chr7:19083848-19093820	23.5552	10.2941	-1.19422	0.000195236	yes
2700099C18Rik	chr17:94750099-94834799	8.35844	3.65322	-1.19406	0.00578321	yes
Lpcat1	chr13:73467382-73514538	54.7753	23.9427	-1.19394	0.000195236	yes
Cd276	chr9:58524299-58540940	3.6434	1.59297	-1.19356	0.000195236	yes
Troap	chr15:99074972-99083409	7.42415	3.24637	-1.1934	0.000195236	yes
Echdc3	chr2:6188464-6321611	1.47192	0.643789	-1.19304	0.000370691	yes
Kptn	chr7:16119875-16127516	6.93156	3.03362	-1.19214	0.000195236	yes
Chd3	chr11:69343272-69369426	11.1717	4.89565	-1.19028	0.000195236	yes
Alkbh7	chr17:56997338-56999336	12.7467	5.59141	-1.18884	0.000195236	yes
Man1c1	chr4:134561689-134704290	7.05545	3.09719	-1.18778	0.000195236	yes
Alox5ap	chr5:149265003-149288153	159.954	70.2344	-1.18741	0.000195236	yes
Prc1	chr7:80294450-80316259	88.149	38.7151	-1.18705	0.000195236	yes
Cuta	chr17:26937971-26939538	69.8944	30.7406	-1.18503	0.000195236	yes
Kif23	chr9:61917277-61946799	56.0213	24.6412	-1.18491	0.000195236	yes
S100a6	chr3:90612893-90614414	572.498	252.046	-1.18358	0.000195236	yes
Pros1	chr16:62854333-62929340	7.2552	3.19449	-1.18343	0.000195236	yes
Gtse1	chr15:85859706-85876573	13.0553	5.74872	-1.18332	0.000195236	yes
Kif4	chrX:100626064-100727271	29.0746	12.8087	-1.18263	0.000195236	yes
Tcam1	chr11:106276671-106288143	0.877523	0.386652	-1.1824	0.000370691	yes
Pddc1	chr7:141408183-141414125	12.3174	5.4303	-1.18159	0.000195236	yes
Map3k10	chr7:27656374-27674581	4.25577	1.87796	-1.18025	0.000195236	yes
Aes	chr10:81559443-81566371	64.2994	28.3776	-1.18005	0.000195236	yes
Gps1	chr11:120784271-120789102	5.94743	2.62607	-1.17936	0.000195236	yes
1700112E06Rik	chr14:22019711-23094571	6.49131	2.86923	-1.17785	0.000195236	yes
Clstn3	chr6:124430755-124464784	1.32438	0.58546	-1.17768	0.000195236	yes
Rcc2	chr4:140701472-140723220	182.159	80.5625	-1.17702	0.000195236	yes
Rps10	chr17:27630428-27635242	6.0194	2.6628	-1.17668	0.000370691	yes
Naaa	chr5:92257659-92278181	17.5899	7.78189	-1.17655	0.000195236	yes
Spc24	chr9:21755441-21760286	47.0699	20.8399	-1.17546	0.000195236	yes
Dusp28	chr1:92906988-92908620	5.69555	2.52188	-1.17533	0.000195236	yes
Tspan33	chr6:29694213-29718558	0.623381	0.276287	-1.17395	0.0186727	yes
Tmem238	chr7:4784784-4789560	14.4154	6.38994	-1.17374	0.000195236	yes
H2-Q2	chr17:35342332-35345722	5.60278	2.48908	-1.17053	0.000195236	yes
Osgep	chr14:50915373-50924893	10.6401	4.72713	-1.17048	0.000195236	yes
Mpnd	chr17:56009200-56036637	33.0165	14.6788	-1.16945	0.000195236	yes
Mocs3	chr2:168230621-168232303	12.084	5.37411	-1.169	0.000195236	yes
Asrgl1	chr19:9111718-9135566	17.795	7.91807	-1.16825	0.000195236	yes
Pck2	chr14:55540265-55550017	9.02436	4.01758	-1.1675	0.000195236	yes
Arl10	chr13:54575012-54581128	5.97598	2.66168	-1.16684	0.000195236	yes
Lrrc75a	chr11:62604883-62648523	29.9548	13.3519	-1.16574	0.000195236	yes
Tagap1	chr17:6954964-6961156	5.19103	2.31424	-1.16548	0.000195236	yes
Gtf2ird2	chr5:134184037-134218143	3.93416	1.75469	-1.16484	0.000195236	yes
B3galt6	chr4:155989465-155992678	7.58643	3.39381	-1.16052	0.000195236	yes
Arl4d	chr11:101665540-101667832	0.662979	0.296668	-1.16012	0.0209746	yes

Casc5	chr2:119047118-119104121	37.6085	16.8348	-1.15961	0.000195236	yes
Ect2	chr3:27097221-27153878	39.7471	17.7942	-1.15944	0.000195236	yes
Ttc32	chr12:9029996-9036394	39.352	17.6274	-1.15862	0.000195236	yes
Glrx5	chr12:105032688-105040910	99.3464	44.5256	-1.15783	0.000195236	yes
Cnpy3	chr17:46735710-46752214	35.2914	15.8186	-1.1577	0.000195236	yes
Agpat2	chr2:26593056-26604417	26.6115	11.9353	-1.15681	0.000195236	yes
Csf1r	chr18:61105571-61131139	273.137	122.605	-1.1556	0.000195236	yes
Hddc2	chr10:31313404-31328086	25.9483	11.6492	-1.15542	0.000195236	yes
Use1	chr8:71366847-71369732	63.7427	28.6202	-1.15522	0.000195236	yes
Tnfsf12,Tnfsf13,Tnfsfm1	chr11:69682576-69696098	6.00001	2.69466	-1.15486	0.000195236	yes
Gstm1	chr3:108012249-108017973	3.07621	1.38192	-1.15449	0.000195236	yes
Lsm5	chr6:56701062-56704699	5.25088	2.35922	-1.15425	0.00428914	yes
Snhg8	chr3:123507551-123508336	45.0543	20.2474	-1.15393	0.000195236	yes
Cdk1	chr10:69336634-69352912	133.808	60.1339	-1.15391	0.000195236	yes
Ncaph2	chr15:89355718-89377037	44.4137	19.9712	-1.15308	0.000195236	yes
Atoh8	chr6:72206176-72235577	0.580702	0.261147	-1.15294	0.003245	yes
Fancd2	chr6:113531681-113596285	22.317	10.0434	-1.15189	0.000195236	yes
Tmem191c	chr16:17276299-17278661	1.07731	0.484878	-1.15174	0.0162041	yes
Nipsnap1	chr11:4874002-4894200	14.8434	6.68474	-1.15088	0.000195236	yes
Jag2	chr12:112908589-112929495	5.11582	2.30428	-1.15065	0.000195236	yes
Zfp248	chr6:118427318-118455506	1.19683	0.539165	-1.15042	0.000195236	yes
Slc43a2	chr11:75531693-75577572	18.1719	8.18684	-1.15033	0.000195236	yes
Ndufs6	chr13:73319875-73328482	36.1077	16.2689	-1.15019	0.000195236	yes
Cryz	chr3:154576519-154623182	9.99954	4.50725	-1.14962	0.000537301	yes
Qdpr	chr5:45434031-45450229	55.2239	24.92	-1.14799	0.000195236	yes
Lipa	chr19:34492315-34527474	300.823	135.814	-1.14728	0.000195236	yes
Gpr19	chr6:134869091-134897925	2.2173	1.0013	-1.14693	0.000195236	yes
Guca1a	chr17:47394557-47400584	12.925	5.83821	-1.14656	0.000195236	yes
Tbc1d2	chr4:46604389-46650199	1.6559	0.748084	-1.14635	0.000195236	yes
Zbtb3	chr19:8802495-8804854	1.60949	0.727661	-1.14526	0.000195236	yes
Psma5	chr3:108256925-108279952	19.0244	8.60649	-1.14435	0.000195236	yes
Samm50	chr15:84192232-84214303	95.0627	43.0234	-1.14376	0.000195236	yes
Rpl8	chr15:76904070-76906318	1471.99	666.452	-1.14319	0.000195236	yes
Gemin7	chr7:19564948-19573343	31.3915	14.2474	-1.13967	0.000195236	yes
Usf2	chr7:30945247-30956803	20.0509	9.11043	-1.13808	0.000195236	yes
Aurka	chr2:172356189-172370570	93.1117	42.3662	-1.13605	0.000195236	yes
Mettl17	chr14:51884841-51891868	12.7683	5.81025	-1.13589	0.000195236	yes
Fam117a	chr11:95337017-95381872	2.42841	1.10595	-1.13472	0.000195236	yes
Gng5	chr3:146499835-146505543	14.5869	6.64659	-1.13399	0.000370691	yes
Sfxn5	chr6:85213050-85333422	4.34914	1.9827	-1.13326	0.000195236	yes
Pycr1	chr11:120635711-120643670	0.668176	0.304701	-1.13284	0.000855787	yes
Ptges2	chr2:32395889-32402740	19.5849	8.93149	-1.13277	0.000195236	yes
Top2a	chr11:98992946-99024189	280.701	128.02	-1.13267	0.000195236	yes
Rhbdd3	chr11:5099272-5106100	4.96023	2.26229	-1.13262	0.000195236	yes
Pmm1	chr15:81951105-81960930	18.1254	8.27232	-1.13165	0.000195236	yes
1600010M07Rik	chr7:109998376-110006646	8.46207	3.86217	-1.1316	0.000370691	yes
Gm6402	chr17:30215523-30576287	10.7429	4.90484	-1.13111	0.0381645	yes
Atg16l2	chr7:101289615-101302088	8.20954	3.74826	-1.13108	0.000195236	yes
Gnb1l	chr16:18498712-18566680	2.96957	1.35691	-1.12993	0.000195236	yes
Gins2	chr8:120488865-120589075	33.3336	15.2595	-1.12727	0.000537301	yes
Ppp1r16a	chr15:76671679-76694915	5.03686	2.30599	-1.12714	0.000195236	yes
Nthl1	chr17:24632681-24638838	8.38035	3.83889	-1.12632	0.000195236	yes
Ccdc166	chr15:75979871-75982285	1.88885	0.865472	-1.12595	0.000195236	yes
Nsmce4a	chr7:130532525-130547381	117.324	53.8164	-1.12437	0.000195236	yes
Rpa3	chr6:8255935-8259141	38.6321	17.7506	-1.12193	0.000195236	yes
Mypop	chr7:18991244-19001766	1.37013	0.629647	-1.1217	0.000195236	yes
Saal1	chr7:46686107-46710651	9.45627	4.34763	-1.12104	0.000195236	yes
Asf1b	chr8:83955693-83970195	62.916	28.9274	-1.12099	0.000195236	yes
Eef2k	chr7:120842830-120907219	1.70623	0.784766	-1.12047	0.000195236	yes
Mrps16	chr14:20391230-20393555	57.2532	26.335	-1.12038	0.000195236	yes
Poll	chr19:45552275-45560543	4.61674	2.12361	-1.12036	0.000195236	yes
Kmo	chr1:175632192-175660853	1.05831	0.486872	-1.12014	0.00188078	yes
Birc5	chr11:117849236-117855743	222.185	102.258	-1.11954	0.000195236	yes
Nit2	chr16:57156664-57167332	29.5728	13.6216	-1.11838	0.000195236	yes
Tmod4	chr3:95124513-95129208	0.481517	0.221928	-1.11749	0.0343814	yes

9130008F23Rik	chr17:40875481-40880558	2.23362	1.02958	-1.11733	0.000195236	yes
Josd2	chr7:44467979-44471658	30.5364	14.0802	-1.11686	0.000195236	yes
Mzt2	chr16:15848440-15863322	10.1932	4.70846	-1.11428	0.000195236	yes
Anln	chr9:22055459-22389206	67.2735	31.0924	-1.11348	0.000195236	yes
Zfp467	chr6:48427691-48445825	1.47806	0.683395	-1.11291	0.00160026	yes
A830082N09Rik	chr10:33987215-33995055	0.701884	0.324589	-1.11262	0.000195236	yes
Tacc3	chr5:33658146-33672197	85.2302	39.4368	-1.11182	0.000195236	yes
Sort1	chr3:108284063-108361519	6.71136	3.10716	-1.11101	0.000195236	yes
2010111I01Rik	chr13:62964892-63431745	26.4521	12.2492	-1.11069	0.000195236	yes
Rbck1	chr2:152316333-152332639	50.3534	23.3283	-1.11001	0.000195236	yes
Med22	chr2:26905266-26910642	21.7965	10.1051	-1.10902	0.000195236	yes
Mutyh	chr4:116807733-116819431	4.51809	2.09506	-1.10872	0.000195236	yes
Fkbp8	chr8:70527742-70535328	81.6571	37.8678	-1.10861	0.000195236	yes
Nudt1	chr5:140331921-140338135	19.6107	9.09948	-1.10778	0.000195236	yes
Galt	chr4:41755227-41759224	4.04155	1.87687	-1.10658	0.000195236	yes
Vwa7	chr17:35016578-35028016	0.846938	0.393351	-1.10644	0.000195236	yes
Znhit2	chr19:6061206-6062468	19.2386	8.93814	-1.10595	0.000195236	yes
Rftn1	chr17:49993306-50190497	18.074	8.39861	-1.10569	0.000195236	yes
Kbtbd3	chr9:4309742-4331732	1.78235	0.828554	-1.10512	0.00100977	yes
Cenpl	chr1:161070766-161086724	18.4534	8.58527	-1.10395	0.000195236	yes
Coq7	chr7:118509658-118533356	52.7321	24.538	-1.10366	0.000195236	yes
Tnfaip8l1	chr17:56162490-56173955	4.67915	2.1785	-1.10291	0.000195236	yes
Suv39h1	chrX:8061170-8074760	27.5327	12.824	-1.1023	0.000195236	yes
Ino80b	chr6:83121827-83125029	12.0849	5.62913	-1.10222	0.000195236	yes
Tcf3	chr10:80409164-80433653	18.8844	8.80164	-1.10135	0.000195236	yes
Gas2	chr7:51879144-51994458	5.88379	2.74276	-1.10112	0.000195236	yes
Synpo	chr18:60593989-60624305	1.48054	0.690492	-1.10042	0.000195236	yes
Gmnn	chr13:24751844-24761937	120.01	55.9808	-1.10015	0.000195236	yes
Tsku	chr7:98350667-98361328	2.21246	1.03229	-1.0998	0.000370691	yes
Spc25	chr2:69193894-69206213	69.0212	32.2097	-1.09954	0.000195236	yes
Ascc1	chr10:59987908-60099990	17.6971	8.26292	-1.09879	0.00271906	yes
Mydgf	chr17:56176540-56183920	120.059	56.0631	-1.09863	0.000195236	yes
Slc29a1	chr17:45585199-45599603	77.264	36.0926	-1.09809	0.000195236	yes
Uba52	chr8:70508265-70510367	137.052	64.0354	-1.09779	0.000195236	yes
Cacna1s	chr1:136052900-136119822	0.467196	0.218358	-1.09733	0.000195236	yes
Tmtc4	chr14:122918974-122983261	9.13745	4.271	-1.09722	0.000195236	yes
Park7	chr4:150897132-150909921	258.015	120.623	-1.09695	0.000195236	yes
Xrcc6bp1	chr10:126868427-126901371	20.001	9.35323	-1.09654	0.000195236	yes
1700113A16Rik	chr3:88171559-88177785	3.12238	1.46026	-1.09642	0.000370691	yes
Cmc2	chr8:116888684-116921436	23.9851	11.2234	-1.09563	0.000195236	yes
2310009B15Rik	chr1:138851978-138856854	27.6513	12.9414	-1.09536	0.000195236	yes
Eci1	chr17:24426682-24439316	33.0361	15.4643	-1.0951	0.000195236	yes
Siva1	chr12:112644827-112649152	118.97	55.7069	-1.09467	0.000195236	yes
Phf19	chr2:34893754-34913976	10.6499	4.98838	-1.0942	0.000195236	yes
Nlrp10	chr7:108921852-108930158	8.44536	3.95584	-1.09417	0.000195236	yes
Eri3	chr4:117550323-117674297	36.2434	16.9796	-1.09392	0.000195236	yes
Get4	chr5:139252323-139270050	25.2708	11.8405	-1.09375	0.000195236	yes
Fxn	chr19:24261452-24280586	15.9543	7.47791	-1.09324	0.000195236	yes
Slc6a12	chr6:121346696-121365773	61.049	28.6243	-1.09273	0.000195236	yes
C87436	chr6:86438397-86480830	8.30686	3.8951	-1.09264	0.000195236	yes
Ndufa3	chr7:3617372-3620161	152.968	71.7312	-1.09256	0.000195236	yes
Elp6	chr9:110305191-110322102	9.45463	4.43476	-1.09217	0.000195236	yes
Dyrk3	chr1:131128440-131138234	28.0358	13.1539	-1.09179	0.000195236	yes
Tmem147	chr7:30727700-30729534	89.8313	42.1481	-1.09175	0.000195236	yes
Crebzf	chr7:90442780-90448043	26.0045	12.2153	-1.09007	0.000195236	yes
Ahrr	chr13:74211117-74292309	0.478956	0.225009	-1.08991	0.00145427	yes
2810417H13Rik	chr9:65828925-65908794	96.4597	45.3255	-1.0896	0.000195236	yes
Zfp958	chr8:4613169-4630231	25.1684	11.827	-1.08953	0.000195236	yes
Ccsap	chr8:123840843-123860209	4.77681	2.24541	-1.08907	0.000195236	yes
Rpusd4	chr9:35267880-35275957	29.3416	13.8015	-1.08812	0.000195236	yes
Platr8	chr2:109902395-109913319	0.577408	0.271724	-1.08745	0.0401573	yes
Dut	chr2:125247247-125259049	98.942	46.5944	-1.08643	0.000195236	yes
AA474331	chr10:39892759-39899238	4.17138	1.96444	-1.08641	0.00416625	yes
Htra2	chr6:83034223-83057682	53.7837	25.3288	-1.08639	0.0345557	yes
Apobec1	chr6:122577791-122602444	72.7636	34.2792	-1.08588	0.000195236	yes

Hadh	chr3:131233419-131272101	37.554	17.6936	-1.08573	0.000195236	yes
0610009O20Rik	chr18:38238404-38262629	12.7545	6.01137	-1.08525	0.000195236	yes
Il12rb2	chr6:67292017-67376137	0.676533	0.318882	-1.08514	0.00230418	yes
Arf2	chr11:103966724-103985350	36.0003	16.9833	-1.08389	0.000195236	yes
Cops7b	chr1:86587099-86606500	12.8968	6.08774	-1.08303	0.000195236	yes
Limd2	chr11:106156255-106160142	30.0459	14.1956	-1.08172	0.000195236	yes
Zscan25	chr5:145283342-145291469	3.52409	1.66523	-1.08153	0.000195236	yes
Eif4ebp1	chr8:27260326-27275656	69.673	33.054	-1.07578	0.000195236	yes
Cox7c	chr13:86044797-86046795	17.5108	8.30895	-1.07551	0.000195236	yes
G2e3	chr12:51348229-51376986	24.1405	11.4825	-1.07202	0.000195236	yes
Cox8a	chr19:7215157-7217616	464.831	221.525	-1.06924	0.000195236	yes
Wrb	chr16:96145418-96157852	21.4814	10.241	-1.06873	0.000195236	yes
Zfp12	chr5:143235162-143248834	6.39708	3.05036	-1.06843	0.000195236	yes
Gm6251	chr10:20148919-20281590	91.3967	43.5849	-1.06831	0.000195236	yes
Aunip	chr4:134510998-134523922	15.0259	7.16637	-1.06814	0.000195236	yes
Wdr4	chr17:31494321-31512487	15.3805	7.33609	-1.06802	0.000195236	yes
Maf1	chr15:76351293-76354378	23.9538	11.4263	-1.0679	0.000195236	yes
Pstk	chr7:131371145-131387838	11.0068	5.25347	-1.06705	0.000195236	yes
Pard6a	chr8:105701147-105703494	4.20348	2.00655	-1.06687	0.000195236	yes
Ttk	chr9:83834688-83872390	38.4458	18.3679	-1.06564	0.000195236	yes
Tnfsf13b	chr8:10006632-10035999	5.76002	2.7521	-1.06554	0.000195236	yes
Itpr3	chr17:27057303-27122223	18.766	8.9683	-1.06521	0.000195236	yes
Dnlz	chr2:26315532-26352110	6.83048	3.267	-1.06402	0.000195236	yes
Cox5a	chr9:57521231-57532426	211.491	101.186	-1.06358	0.000195236	yes
Srd5a1	chr13:69573448-69611463	3.18034	1.52455	-1.0608	0.000195236	yes
Ptms	chr6:124913674-124917946	74.9742	35.9585	-1.06006	0.000195236	yes
1810044D09Rik	chr6:91440986-91441755	1.38968	0.667063	-1.05886	0.0413997	yes
Arhgef6	chrX:57231484-57338729	96.7888	46.4748	-1.05839	0.000195236	yes
Lage3	chrX:74352161-74353618	47.2169	22.6788	-1.05796	0.000195236	yes
Rhebl1	chr15:98877759-98881414	4.60932	2.2146	-1.05751	0.000195236	yes
Gbas	chr5:129725074-129758325	30.0563	14.4441	-1.05718	0.000195236	yes
Fis1	chr5:136953274-136966234	72.8688	35.0216	-1.05706	0.000195236	yes
Btbd18	chr2:84659078-84668781	0.534702	0.256996	-1.05699	0.00271906	yes
Fdps	chr3:89093587-89101967	17.4124	8.37245	-1.05639	0.000195236	yes
Cdk4	chr10:127063602-127067282	294.543	141.658	-1.05607	0.000195236	yes
Bambi	chr18:3507956-3516404	0.698172	0.335876	-1.05565	0.000195236	yes
Vegfb	chr19:6982471-6987651	10.7672	5.18316	-1.05474	0.000195236	yes
Rpl3	chr15:80077780-80083406	15.7871	7.60502	-1.05372	0.000195236	yes
C030006K11Rik,Lrrc24	chr15:76715275-76723845	7.43665	3.58273	-1.05359	0.000195236	yes
Fkbp11	chr15:98724367-98728198	30.0052	14.4559	-1.05355	0.000195236	yes
Thap11	chr8:105855102-105856950	26.1871	12.6242	-1.05267	0.000195236	yes
Cdt1	chr8:122568014-122573130	45.4636	21.9202	-1.05245	0.000195236	yes
Rbm3	chrX:8138974-8147963	11.9925	5.78317	-1.0522	0.000698636	yes
Isoc2a	chr7:4877052-4895716	12.3507	5.95809	-1.05167	0.000195236	yes
Megf9	chr4:70431926-70534928	22.6416	10.9228	-1.05163	0.000195236	yes
Mis18bp1	chr12:65132734-65172580	58.2554	28.1132	-1.05115	0.000195236	yes
Shcbp1	chr8:4735979-4779534	75.9197	36.644	-1.0509	0.000195236	yes
Sorl1	chr9:41968488-42124289	2.99001	1.44352	-1.05056	0.000195236	yes
Rpl27a	chr7:109519194-109522369	42.2675	20.4165	-1.04981	0.000195236	yes
Ttf2	chr3:100938859-100969663	16.8714	8.15206	-1.04934	0.000195236	yes
Tgfbr1	chr4:47353304-47414924	64.1617	31.0091	-1.04902	0.000195236	yes
Mdm1	chr10:118141786-118168999	8.21394	3.96989	-1.04897	0.000195236	yes
Mgme1	chr2:144270903-144281227	14.4179	6.97658	-1.04727	0.000195236	yes
B9d1	chr11:61505171-61512927	4.37447	2.1173	-1.04688	0.000370691	yes
Reep4	chr14:70545250-70548935	24.5319	11.877	-1.04649	0.000195236	yes
Uqcrfs1	chr13:30540311-30545316	118.908	57.5716	-1.04642	0.000195236	yes
Ptp4a3	chr15:73723144-73758766	20.722	10.0381	-1.04568	0.000195236	yes
Dars2	chr1:161040612-161070632	45.5258	22.0632	-1.04504	0.000195236	yes
Cdca7	chr2:72476218-72486890	47.3139	22.9358	-1.04466	0.000195236	yes
H2-Ke6	chr17:34026032-34028055	24.8616	12.055	-1.04428	0.000195236	yes
Slc26a6	chr9:108854042-108862143	2.73705	1.32725	-1.04419	0.000195236	yes
Pebp1	chr5:117282650-117287564	46.7679	22.6842	-1.04383	0.000195236	yes
Atp5f1	chr3:105942677-105969760	132.521	64.2991	-1.04335	0.00116073	yes
Anapc13	chr9:102626295-102634244	74.0246	35.9372	-1.04253	0.000195236	yes
0610012G03Rik	chr16:31947050-31948521	19.8498	9.64477	-1.0413	0.000195236	yes

Commd4	chr9:57155040-57158299	41.903	20.3661	-1.04088	0.000195236	yes
Snrnp25	chr11:32205414-32208995	18.1727	8.83462	-1.04053	0.000195236	yes
1190002F15Rik	chr6:134929091-134951718	12.9404	6.29696	-1.03915	0.000195236	yes
Zfp759	chr13:67128227-67141787	4.30459	2.09534	-1.03869	0.000195236	yes
Cope	chr8:70302784-70312990	69.436	33.7995	-1.03868	0.000195236	yes
Fam107b	chr2:3713457-3782134	22.9126	11.1572	-1.03817	0.000195236	yes
Hist3h2a	chr11:58954684-58955192	604.017	294.759	-1.03505	0.000195236	yes
Dqx1	chr6:83057843-83067219	0.452321	0.220744	-1.03497	0.0111512	yes
Agap2	chr10:127078906-127093170	0.772073	0.376875	-1.03465	0.000195236	yes
Gm5464	chr14:66868849-66871005	1.31204	0.640525	-1.03448	0.00311626	yes
Rsph3b	chr17:6904715-6948356	0.552871	0.270212	-1.03285	0.0145094	yes
Gclc	chr9:77754534-77794489	93.5146	45.7047	-1.03285	0.000195236	yes
Gstz1	chr12:87106865-87164723	22.5039	11.0031	-1.03227	0.000195236	yes
Mast1	chr8:84911852-84937353	0.57776	0.282749	-1.03095	0.00416625	yes
Slc26a11	chr11:119355556-119381076	0.757706	0.370842	-1.03083	0.00893132	yes
Gm5148	chr3:37714189-37724360	158.947	77.8187	-1.03035	0.000195236	yes
Impa2	chr18:67289222-67318841	27.82	13.6235	-1.03002	0.000195236	yes
Nsun5	chr5:135369952-135394546	11.0032	5.38929	-1.02976	0.00428914	yes
6330416G13Rik	chr4:63560359-63586353	8.87059	4.34484	-1.02973	0.000195236	yes
Npepl1	chr2:174110350-174122702	60.1798	29.4818	-1.02946	0.000195236	yes
Gins3	chr8:95633558-95645059	22.7372	11.1433	-1.02888	0.000195236	yes
Them6	chr15:74721233-74724373	14.445	7.07964	-1.02883	0.000195236	yes
Slc25a39	chr11:102402975-102407517	83.1659	40.7645	-1.02868	0.000195236	yes
Gsg2	chr11:73090594-73147449	12.9015	6.32494	-1.02842	0.000195236	yes
Ctnnbip1	chr4:149518240-149566437	14.8989	7.30575	-1.0281	0.000195236	yes
Ethe1	chr7:24583837-24608926	13.962	6.84975	-1.02738	0.000195236	yes
Tgif2	chr2:156840006-156862945	3.16513	1.55401	-1.02627	0.000195236	yes
Rps5	chr7:12922310-12926686	1528.43	750.447	-1.02622	0.000195236	yes
Ajuba	chr14:54567468-54577661	0.780689	0.383679	-1.02485	0.00202512	yes
Hdac5	chr11:102195746-102230172	14.8801	7.31443	-1.02457	0.000195236	yes
Dfna5	chr6:50207402-50261769	2.29524	1.12945	-1.02302	0.000195236	yes
Zfp787	chr7:6131488-6155971	9.86285	4.85391	-1.02286	0.000195236	yes
Cdca8	chr4:124918464-124936917	58.899	28.9943	-1.02247	0.000195236	yes
Zfpm1	chr8:122282140-122337247	5.44678	2.68235	-1.0219	0.000195236	yes
Sgol2a	chr1:57985339-58026277	47.95	23.6266	-1.02112	0.000195236	yes
Pold1	chr7:44532743-44548815	33.4334	16.4834	-1.02028	0.000195236	yes
Sgk1	chr10:21882183-21999902	6.71626	3.31308	-1.01949	0.000195236	yes
Irak1bp1	chr9:82829805-82847688	7.14623	3.52853	-1.01812	0.000195236	yes
Nkain1	chr4:130530130-130574036	3.67051	1.81414	-1.01669	0.000195236	yes
Eif3m	chr2:104886323-105017027	21.0326	10.4061	-1.01519	0.000195236	yes
Sf3b5	chr10:13008449-13009183	161.624	80.0125	-1.01434	0.000195236	yes
Prps2	chrX:167346332-167382699	43.1391	21.3719	-1.01328	0.000195236	yes
Depdc1a	chr3:159495432-159529955	10.1237	5.01571	-1.01321	0.000195236	yes
Capn5	chr7:98121558-98184799	6.14746	3.04575	-1.01319	0.000195236	yes
Commd1	chr11:22899727-22982284	103.78	51.4256	-1.01297	0.000195236	yes
Sap18	chr14:57798188-57804980	9.20603	4.56555	-1.01179	0.000195236	yes
Serinc3	chr2:163623272-163645800	215.172	106.739	-1.0114	0.000195236	yes
Idh2	chr7:80094846-80115350	170.537	84.6205	-1.011	0.000195236	yes
Ruvbl2	chr7:45421897-45434464	101.71	50.5004	-1.0101	0.000195236	yes
Lime1	chr2:181381234-181383628	3.70801	1.84153	-1.00974	0.000195236	yes
Il12rb1	chr8:70808448-70821423	0.488627	0.242673	-1.00972	0.0116945	yes
H2afj	chr6:136808247-136810074	41.0865	20.4063	-1.00965	0.000195236	yes
Rnf26	chr9:44110780-44113051	9.3722	4.65528	-1.00952	0.000195236	yes
Pih1d1	chr7:45141839-45160064	45.0469	22.3802	-1.00921	0.00174394	yes
Smtn	chr11:3517521-3539292	0.515462	0.256264	-1.00824	0.000698636	yes
Rnf166	chr8:122466146-122476064	15.5527	7.73291	-1.00808	0.000195236	yes
Ccs	chr19:4825365-4839322	12.8088	6.37837	-1.00588	0.000195236	yes
Mfap1b	chr2:121461665-121474023	0.95934	0.477755	-1.00577	0.003245	yes
Ppp1r37	chr7:19530966-19562398	9.35862	4.66196	-1.00536	0.000195236	yes
Rpl3l	chr17:24727828-24736149	4.1651	2.07646	-1.00422	0.000195236	yes
Glod4	chr11:76220394-76243699	30.4327	15.1729	-1.00412	0.000195236	yes
Cep55	chr19:38055024-38074425	69.3825	34.6242	-1.00279	0.000195236	yes
Ulk4	chr9:120964453-121277172	1.22657	0.612187	-1.00259	0.000195236	yes
Thap4	chr1:93705390-93754838	16.4821	8.23097	-1.00177	0.000195236	yes
Grk6	chr13:55445071-55460927	29.8019	14.8883	-1.00122	0.000195236	yes

Fam73b	chr2:30364232-30385519	5.65431	2.8254	-1.0009	0.000195236	yes
Snrpc	chr17:27840086-27851967	17.6411	8.81616	-1.00072	0.000195236	yes
Nubpl	chr12:52097745-52310959	5.05044	2.52482	-1.00023	0.000537301	yes
Mrpl27	chr11:94653790-94660087	25.8621	12.9311	-0.999996	0.000195236	yes
4930413G21Rik	chr7:122969057-122970459	4.17603	2.08907	-0.999271	0.000195236	yes
Gm11974	chr11:6525590-6528760	30.4286	15.2299	-0.998526	0.000195236	yes
Cntrob	chr11:69299495-69323873	4.50904	2.25717	-0.998307	0.000195236	yes
Melk	chr4:44300916-44364675	37.5408	18.7937	-0.998215	0.000195236	yes
Dffb	chr4:153964448-153975081	4.95082	2.47864	-0.99812	0.000195236	yes
Cdca7l	chr12:117843860-118199043	61.0069	30.5509	-0.997759	0.000195236	yes
Pxdn	chr12:29938035-30017658	2.16519	1.08433	-0.997686	0.000195236	yes
Tyms	chr5:30058199-30073625	19.8794	9.96307	-0.996613	0.000195236	yes
Psmb4	chr3:94884323-94886958	235.847	118.202	-0.996593	0.000195236	yes
Bsn	chr9:108096021-108190383	0.539204	0.270261	-0.996478	0.000195236	yes
Lancl1	chr1:67000516-67038872	11.1734	5.60115	-0.996268	0.000195236	yes
Mvb12a	chr8:71542929-71548026	48.6235	24.3803	-0.995936	0.000195236	yes
Rdh10	chr1:16105881-16132550	16.2754	8.17615	-0.9932	0.000195236	yes
Ndufc2	chr7:97400002-97407800	170.763	85.85	-0.992108	0.000195236	yes
Hmgcl	chr4:135946452-135962617	24.6024	12.3797	-0.990825	0.000195236	yes
Lsm2	chr17:34981853-34985893	68.6826	34.5673	-0.990538	0.000195236	yes
Zfp637	chr6:117841241-117845956	9.68016	4.87542	-0.989505	0.000195236	yes
Tmem186	chr16:8633730-8637701	11.9446	6.01789	-0.989029	0.000195236	yes
Hnrnpa0	chr13:58125878-58128556	108.678	54.7729	-0.988531	0.000195236	yes
Rps11	chr7:45122387-45124389	304.218	153.327	-0.988493	0.000195236	yes
Anapc11	chr11:120598420-120608198	10.799	5.44364	-0.988249	0.000195236	yes
Adck1	chr12:88360513-88461726	11.1333	5.61218	-0.988244	0.000195236	yes
Set	chr2:30061995-30072577	251.591	126.832	-0.988166	0.000195236	yes
Gm5512	chr19:12905230-12906985	16.1679	8.1517	-0.987957	0.000195236	yes
Gstp1	chr19:4035410-4037912	4.8808	2.46213	-0.987208	0.00271906	yes
Rab40c	chr17:25882113-25919714	9.05652	4.56956	-0.986901	0.000195236	yes
Spr	chr6:85133679-85137764	42.1466	21.266	-0.986867	0.000195236	yes
Fuk	chr8:110882455-110902488	3.7168	1.8767	-0.985861	0.000195236	yes
Fgd4	chr16:16416914-16599978	37.2667	18.8226	-0.98542	0.000195236	yes
Rxra	chr2:27677200-27763319	2.31299	1.16932	-0.984084	0.000195236	yes
E4f1	chr17:24443777-24455392	5.69277	2.8804	-0.982861	0.000195236	yes
Smad3	chr9:63646766-63757994	3.39117	1.71771	-0.981294	0.000195236	yes
Prickle3	chrX:7657378-7671278	7.20248	3.64903	-0.98098	0.000195236	yes
Slc25a15	chr8:22375549-22398621	9.75485	4.94328	-0.980651	0.000195236	yes
Gm13375	chr2:20968873-20970348	0.794589	0.403066	-0.979194	0.0323114	yes
Gm14326	chr2:177935992-177957288	7.95548	4.03552	-0.979193	0.000195236	yes
Atp6v0d2	chr4:19876837-19922566	9.40317	4.7705	-0.979006	0.000195236	yes
Nde1	chr16:14163274-14192923	64.0727	32.5066	-0.97898	0.000195236	yes
Tmem144	chr3:79813152-79842662	16.5218	8.39542	-0.976699	0.000195236	yes
Rad9b	chr5:122325507-122354195	1.95222	0.992402	-0.976121	0.00216236	yes
Gadd45gip1	chr8:84832281-84840665	36.2315	18.4202	-0.975957	0.000537301	yes
Grwd1	chr7:45825222-45830789	29.5152	15.0298	-0.97363	0.000195236	yes
Hexim2	chr11:103133338-103139908	2.98306	1.51906	-0.973616	0.000195236	yes
Sppl2b	chr10:80855274-80868708	8.23428	4.19484	-0.973028	0.000195236	yes
Lyl1	chr8:84701456-84800338	26.7511	13.6324	-0.972561	0.000195236	yes
B4galt4	chr16:38742258-38769054	2.22699	1.13559	-0.971648	0.000195236	yes
Rangap1	chr15:81704247-81729919	66.4121	33.9052	-0.969939	0.000195236	yes
Zfp709	chr8:71882067-71892565	2.78002	1.41956	-0.969648	0.000195236	yes
Lrrc14	chr15:76710739-76715091	11.7808	6.01781	-0.969132	0.000195236	yes
Taf1c	chr8:119575234-119605240	3.19585	1.63264	-0.96899	0.000195236	yes
Dok3	chr13:55523234-55528538	21.2983	10.8815	-0.968871	0.000195236	yes
Slc25a23	chr17:57043710-57059863	3.47591	1.77613	-0.968655	0.000195236	yes
Mybl2	chr2:163054634-163084687	21.0823	10.7871	-0.966726	0.000195236	yes
Nlrc3	chr16:3946932-3976632	1.99321	1.01993	-0.966616	0.000370691	yes
Ndufb6	chr4:40270662-40279368	133.458	68.2917	-0.966609	0.000195236	yes
Fuom	chr7:140097814-140102441	3.13968	1.60663	-0.966576	0.000195236	yes
Mcat	chr15:83546796-83555711	11.3322	5.8088	-0.964114	0.000195236	yes
Rps7	chr12:28630846-28635953	7.17856	3.68117	-0.963532	0.000195236	yes
Mccc2	chr13:99948531-100015639	13.1682	6.75489	-0.963057	0.000195236	yes
Zfp606	chr7:12478304-12496235	4.63983	2.38165	-0.962112	0.000195236	yes
Trim45	chr3:100922492-100936925	1.8039	0.926055	-0.961946	0.000195236	yes

Nsmce1	chr7:125467639-125491542	47.7081	24.4986	-0.961537	0.000195236	yes
Lamtor1	chr7:101899807-101911903	64.3591	33.054	-0.961319	0.000195236	yes
Gchfr	chr2:119167787-119172389	6.24861	3.20943	-0.961218	0.00258324	yes
Tigd3	chr19:5891137-5894107	0.496116	0.254885	-0.960833	0.0476469	yes
Sdsl	chr5:120458201-120472763	2.25544	1.1588	-0.960784	0.00847587	yes
Hps6	chr19:46003477-46006173	5.73103	2.94484	-0.960607	0.000195236	yes
Psenen	chr7:30561865-30563184	2.82212	1.45058	-0.960148	0.023171	yes
Prpf19	chr19:10895230-10909559	35.1494	18.075	-0.959503	0.000195236	yes
Wbp1	chr6:83119043-83121461	13.8153	7.10459	-0.959444	0.000195236	yes
Cir1	chr2:73283871-73312592	0.967967	0.497807	-0.959373	0.00390381	yes
Zfp433	chr10:81704824-81721976	0.668892	0.344035	-0.959216	0.0292744	yes
Acaa1b	chr9:119148042-119157093	4.35213	2.239	-0.958865	0.000370691	yes
Rfc2	chr5:134582689-134598328	65.4589	33.6766	-0.958842	0.000195236	yes
Lmna	chr3:88481147-88503352	382.218	196.782	-0.957798	0.000195236	yes
Cox7b	chrX:106015699-106022450	129.889	66.888	-0.957458	0.000195236	yes
Recql4	chr15:76701541-76710559	8.49578	4.37502	-0.957457	0.000195236	yes
Fbxl6	chr15:76535727-76538746	14.1093	7.26835	-0.956952	0.000195236	yes
Maz	chr7:127022136-127026479	50.4989	26.0335	-0.955884	0.000195236	yes
Gm14325	chr2:177828990-177840318	4.23821	2.18556	-0.955453	0.000195236	yes
Hist3h2bb-ps	chr11:58954047-58954512	17.233	8.88992	-0.954932	0.000370691	yes
Tab1	chr15:80133153-80161702	9.37343	4.83616	-0.954715	0.000195236	yes
Ap1s1	chr5:137034993-137046060	78.7172	40.6257	-0.954286	0.000195236	yes
Fam98c	chr7:29134932-29156210	13.636	7.03943	-0.953893	0.0036408	yes
Cryl1	chr14:57275033-57398483	24.6241	12.7119	-0.95389	0.000195236	yes
Nubp1	chr16:10411937-10447350	28.7943	14.8678	-0.953596	0.000195236	yes
Slc44a1	chr4:53440412-53622478	80.4349	41.5413	-0.953273	0.000195236	yes
Cep44	chr8:56531521-56550566	16.4827	8.5134	-0.953142	0.000195236	yes
Mylip	chr13:45389741-45411940	16.4925	8.5198	-0.952917	0.000195236	yes
Hddc3	chr7:80343136-80346097	29.2865	15.1379	-0.952073	0.000195236	yes
Klhdc9	chr1:171358448-171360798	2.98275	1.54216	-0.951692	0.000855787	yes
Orai2	chr5:136147460-136170656	9.23597	4.77754	-0.950995	0.000195236	yes
Endov	chr11:119491346-119511465	1.72334	0.891534	-0.950846	0.000195236	yes
Hist1h2bl	chr13:21715712-21716143	160.424	82.9964	-0.950768	0.000195236	yes
Mfsd10	chr5:34633646-34637114	17.4837	9.04685	-0.950522	0.000195236	yes
Paqr7	chr4:134496760-134510237	8.59516	4.4487	-0.950142	0.000195236	yes
Tsen54	chr11:115814738-115823102	13.7349	7.10979	-0.949971	0.000195236	yes
Cox7a2	chr9:79755240-79759853	299.768	155.197	-0.94975	0.000195236	yes
Zbtb2	chr10:4367073-4388108	8.86333	4.58997	-0.949364	0.000195236	yes
Abca2	chr2:25428673-25448539	5.40284	2.79893	-0.948841	0.000195236	yes
Gnb2l1	chr11:48800359-48806241	1430.72	741.995	-0.947262	0.000370691	yes
Zfp94	chr7:24301703-24316666	1.64381	0.852567	-0.947156	0.000698636	yes
Map3k9	chr12:81714949-81781170	2.53898	1.31688	-0.947122	0.000537301	yes
Mfsd12	chr10:81357569-81365820	20.5319	10.6502	-0.946985	0.000195236	yes
Rnmtl1	chr11:76243735-76250622	12.4861	6.47736	-0.946843	0.000195236	yes
Sgol1	chr17:53674786-53689315	26.934	13.9762	-0.946452	0.000195236	yes
C330006A16Rik	chr2:26136806-26140506	10.7132	5.56024	-0.946175	0.000195236	yes
Fbxo44	chr4:148152798-148160094	2.52978	1.31393	-0.945128	0.00100977	yes
Ift140	chr17:25016085-25099497	6.1071	3.17646	-0.943068	0.000195236	yes
Bckdk	chr7:127904072-127909664	48.4848	25.221	-0.942905	0.000195236	yes
Ankrd9	chr12:110975352-110979021	2.10755	1.09635	-0.942856	0.00130921	yes
Lat2	chr5:134600102-134615025	77.4007	40.2684	-0.942699	0.000195236	yes
Mrps35	chr6:147042769-147070902	67.0637	34.9102	-0.941882	0.000195236	yes
Naa38	chr11:69395790-69398234	109.874	57.2756	-0.939854	0.000195236	yes
3010026O09Rik	chr11:50174850-50200115	8.38574	4.37165	-0.939761	0.000195236	yes
Tmem106c	chr15:97964228-97970286	22.1813	11.5831	-0.937324	0.000195236	yes
Bmf	chr2:118528756-118549678	3.09403	1.61682	-0.93633	0.000195236	yes
Rpl28	chr7:4792964-4794547	5.89019	3.07799	-0.936325	0.0148245	yes
Car5b	chrX:163976821-164028010	17.2251	9.00598	-0.935561	0.000195236	yes
Fignl1	chr11:11800287-11808962	45.8574	23.9839	-0.93509	0.000195236	yes
Atp6v1f	chr6:29467782-29470509	87.5034	45.7667	-0.935042	0.000195236	yes
Cyp27a1	chr1:74713573-74737890	13.4906	7.06009	-0.934196	0.000195236	yes
Nfe2	chr15:103248211-103258403	0.58763	0.307646	-0.933636	0.0309827	yes
Stard3nl	chr13:19357675-19395813	31.3504	16.4196	-0.933067	0.000195236	yes
1810032O08Rik,Snord1a	chr11:116671659-116675798	9.85034	5.15992	-0.932825	0.00116073	yes
AB124611	chr9:21526176-21545331	31.1006	16.2957	-0.932448	0.000195236	yes

Zfp472	chr17:32965830-32979211	13.0522	6.83941	-0.932349	0.000195236	yes
Mtch1	chr17:29332075-29347904	80.7158	42.3164	-0.931635	0.000195236	yes
Cyb5a	chr18:84851413-84879863	342.72	179.681	-0.931594	0.000195236	yes
Lztr1	chr16:17508970-17526330	19.3613	10.1529	-0.931292	0.000195236	yes
Syce2	chr8:84872110-84893921	153.449	80.469	-0.931257	0.000195236	yes
Lyz2	chr10:117277540-117282272	2256.13	1184.99	-0.928974	0.000195236	yes
Repin1	chr6:48593882-48599082	5.3524	2.81161	-0.928789	0.000195236	yes
Zfp629	chr7:127607034-127614433	3.73235	1.96134	-0.928244	0.000195236	yes
Npl	chr1:153503015-153549714	16.769	8.8239	-0.926305	0.000195236	yes
Rab4b	chr7:27168432-27178883	12.4808	6.56766	-0.92626	0.000195236	yes
Sgsh	chr11:119314786-119355510	5.39132	2.83788	-0.925827	0.000195236	yes
Vash2	chr1:190947645-190979296	0.550058	0.289609	-0.925478	0.00766958	yes
Brf1	chr12:112959861-113000621	16.8189	8.85598	-0.925361	0.000195236	yes
Enc1	chr13:97241104-97253040	7.04701	3.71094	-0.925226	0.000195236	yes
Timm13	chr10:80879815-80900969	83.5589	44.0622	-0.923251	0.000195236	yes
Stmn1	chr4:134468319-134473843	102.788	54.2135	-0.922948	0.000195236	yes
Dctd	chr8:48099091-48141667	22.4696	11.8512	-0.922939	0.000195236	yes
Lamtor2	chr3:88549818-88552927	97.2234	51.3416	-0.921174	0.000195236	yes
Ranbp3	chr17:56673224-56711769	47.071	24.9036	-0.918483	0.000195236	yes
Tbc1d14	chr5:36490603-36586226	19.2436	10.1852	-0.917901	0.000195236	yes
Wbp5	chrX:136245079-136247139	32.8387	17.3916	-0.91701	0.000195236	yes
Pla2g16	chr19:7557458-7588545	0.719278	0.381098	-0.916387	0.00733312	yes
Fam103a1	chr7:81762952-81769490	10.4234	5.52289	-0.916333	0.000195236	yes
Chmp6	chr11:119602904-119928917	26.0562	13.8226	-0.914599	0.0104999	yes
Pygo2	chr3:89430213-89435130	14.5062	7.69719	-0.914264	0.000195236	yes
Zfp459	chr13:67405712-67421418	0.690814	0.366757	-0.913473	0.0125567	yes
Btbd2	chr10:80642616-80657071	4.87739	2.58954	-0.91341	0.000195236	yes
Gnat2	chr3:108093065-108101430	0.654545	0.34784	-0.912068	0.0243709	yes
Pycrl	chr15:75916462-75921560	33.4044	17.7583	-0.91154	0.000195236	yes
Snx8	chr5:140340302-140389247	22.9139	12.1854	-0.911068	0.000195236	yes
Slc2a9	chr5:38349272-38502152	5.80939	3.08954	-0.910995	0.000195236	yes
Mlycd	chr8:119394891-119411088	5.90006	3.14019	-0.909877	0.000195236	yes
Flcn	chr11:59791407-59810039	10.2562	5.45966	-0.909619	0.000195236	yes
Sptssa	chr12:54645373-54656572	120.54	64.1706	-0.909524	0.000195236	yes
2010012O05Rik	chr19:46689905-46703382	4.30195	2.29196	-0.908408	0.000370691	yes
Stk38l	chr6:146724929-146778814	8.01492	4.2757	-0.906528	0.000195236	yes
Med28	chr5:45520228-45529284	13.656	7.28533	-0.906468	0.000195236	yes
Tef	chr15:81802672-81826863	7.26778	3.88038	-0.905316	0.000195236	yes
Fam167b	chr4:129576814-129578580	62.9306	33.6001	-0.905295	0.000195236	yes
Rnaseh2b	chr14:62332104-62372992	50.914	27.186	-0.905201	0.000195236	yes
Trpm1	chr7:64153834-64269759	1.32642	0.708683	-0.904329	0.000195236	yes
Clmp	chr9:40685963-40784046	1.25694	0.671651	-0.904131	0.000370691	yes
Fdxacb1	chr9:50768237-50772670	6.23509	3.33243	-0.903837	0.000195236	yes
Gm11127	chr17:36042960-36058645	3.52956	1.8913	-0.900111	0.000855787	yes
Ankrd54	chr15:79053093-79062859	12.3354	6.61277	-0.89948	0.000195236	yes
Ankrd13b	chr11:77470486-77489678	5.41388	2.9043	-0.898471	0.000195236	yes
Atp6v1g1	chr4:63544764-63550701	109.249	58.6698	-0.896934	0.000195236	yes
Slc19a1	chr10:77032738-77050432	26.3465	14.1581	-0.895985	0.000195236	yes
AI837181	chr19:5425143-5427316	11.2568	6.05349	-0.894961	0.000195236	yes
Banf1	chr19:5364632-5371511	194.996	104.865	-0.894912	0.00145427	yes
Auh	chr13:52835109-52929677	18.8064	10.1215	-0.8938	0.000195236	yes
Psmb5	chr14:54614119-54617995	52.8665	28.4608	-0.893374	0.000195236	yes
Mrm1	chr11:84813060-84819515	6.53329	3.51925	-0.892541	0.000195236	yes
Ncapd2	chr6:125168006-125191586	42.4576	22.8786	-0.892024	0.000195236	yes
Hist1h3d	chr13:23575762-23576266	215.757	116.272	-0.891904	0.000195236	yes
Zfp239	chr6:117863076-117872766	4.70231	2.53419	-0.891846	0.000195236	yes
E330020D12Rik	chr1:153404185-153414233	2.59168	1.39763	-0.890899	0.000195236	yes
Acvr2b	chr9:119402500-119433506	1.79137	0.966356	-0.89044	0.00467182	yes
9430015G10Rik	chr4:156109997-156127263	12.3867	6.68579	-0.889623	0.000195236	yes
Rbfa	chr18:80186238-80200619	42.7738	23.0924	-0.889308	0.000195236	yes
Msh2	chr17:87672556-87723713	58.7592	31.7375	-0.888627	0.000195236	yes
St3gal2	chr8:110919864-110972497	4.08686	2.20745	-0.888614	0.000195236	yes
Brca1	chr11:101488763-101551955	21.5091	11.6195	-0.888399	0.000195236	yes
Shb	chr4:45423275-45530828	4.16937	2.25393	-0.887389	0.000195236	yes
Prkch	chr12:73585040-73778184	49.0205	26.5097	-0.886865	0.000195236	yes

Tsn	chr1:118298517-118311132	70.2766	38.0081	-0.886739	0.000195236	yes
B4galt7	chr13:55600110-55609954	10.64	5.75667	-0.88619	0.000195236	yes
Hp1bp3	chr4:138216611-138244682	42.6122	23.0625	-0.88572	0.000195236	yes
Fut4	chr9:14748458-14752122	0.882799	0.478083	-0.884823	0.00675438	yes
Clec11a	chr7:44303765-44306959	1.4127	0.765077	-0.884783	0.00675438	yes
Pank4	chr4:154964122-154980938	13.9332	7.54922	-0.884127	0.000195236	yes
Cluap1	chr16:3909008-3941147	10.7234	5.81121	-0.883853	0.000195236	yes
Rnf144b	chr13:47122719-47247991	2.16169	1.17178	-0.883464	0.000370691	yes
Man2b1	chr8:85083268-85098739	19.0746	10.3429	-0.883006	0.000195236	yes
Acbd6	chr1:155558119-155687233	45.1794	24.4995	-0.882916	0.000195236	yes
Apitd1	chr4:149128348-149137600	20.4392	11.0874	-0.882421	0.000195236	yes
Dlgap5	chr14:47387778-47418407	50.4597	27.3856	-0.881715	0.000195236	yes
Fam216a	chr5:122364583-122371963	17.7474	9.63435	-0.881345	0.000195236	yes
Irf2bp1	chr7:19004064-19006763	12.9069	7.00706	-0.881259	0.000195236	yes
Mto1	chr9:78448209-78474152	23.1501	12.5701	-0.881022	0.000195236	yes
Abhd11	chr5:135009151-135013157	31.2569	16.974	-0.880849	0.000195236	yes
Hint1	chr11:54866437-54870496	598.423	324.991	-0.880766	0.000195236	yes
Uevld	chr7:46923215-46958518	8.27921	4.50061	-0.879372	0.000195236	yes
Ercc8	chr13:108158737-108194981	9.31264	5.06252	-0.879334	0.000195236	yes
Trmt61a	chr12:111678104-111683902	20.362	11.076	-0.87844	0.000195236	yes
Cav2	chr6:17281184-17289130	9.38757	5.10847	-0.877861	0.000195236	yes
Dync2li1	chr17:84626498-84655564	0.802416	0.436817	-0.877323	0.0245514	yes
Mastl	chr2:23116543-23156024	12.195	6.64024	-0.876988	0.000195236	yes
Amdhd2	chr17:24155832-24163733	12.0708	6.57384	-0.876717	0.000370691	yes
Tfdp1	chr8:13338750-13378448	71.8126	39.1306	-0.87594	0.000195236	yes
Sdhc	chr1:171129156-171150603	150.454	81.9972	-0.875677	0.000195236	yes
Ndufv3	chr17:31520114-31531325	128.074	69.804	-0.875597	0.000195236	yes
Accs	chr2:93833466-93849943	1.43427	0.782266	-0.874581	0.000537301	yes
Slc35e4	chr11:3907021-3914664	29.6422	16.1686	-0.874463	0.000195236	yes
Pf4	chr5:90772434-90773383	37.8386	20.6418	-0.874291	0.000195236	yes
Pex11b	chr3:96635356-96645381	19.3881	10.5796	-0.873885	0.000195236	yes
Gatsl3	chr11:4218250-4222409	1.46436	0.799076	-0.873862	0.011374	yes
Rpl39	chrX:37082519-37085184	230.541	125.878	-0.872998	0.000370691	yes
Ap2s1	chr7:16738443-16749290	130.364	71.1959	-0.872678	0.000195236	yes
Ctc1	chr11:69015910-69036473	7.70383	4.20781	-0.872506	0.000195236	yes
Tamm41	chr6:115004380-115037874	24.1498	13.1931	-0.872233	0.000195236	yes
Polr2h	chr16:20717825-20722265	38.2772	20.9127	-0.872104	0.000195236	yes
Zfp764	chr7:127403667-127406822	6.52033	3.56248	-0.872063	0.000195236	yes
Anapc5	chr5:122787460-122821342	132.163	72.2377	-0.871499	0.000195236	yes
Sirpa	chr2:129592838-129632228	127.996	69.9635	-0.871424	0.000195236	yes
Timm9	chr12:71111427-71136675	56.4223	30.8536	-0.870826	0.000195236	yes
Lrmp	chr6:145115635-145210970	67.1283	36.7106	-0.870725	0.000195236	yes
Mrpl28	chr17:26123502-26126613	69.5731	38.0661	-0.870022	0.000195236	yes
Dsn1	chr2:156995061-157007075	16.9876	9.2948	-0.86999	0.000195236	yes
2210013O21Rik	chrX:153723553-153741296	17.5755	9.61975	-0.86949	0.000370691	yes
Spsb3	chr17:24886673-24892147	11.1334	6.09432	-0.869356	0.000195236	yes
Rps27l	chr9:66946117-66949509	248.032	135.784	-0.869216	0.000195236	yes
Rab11fip5	chr6:85334961-85374634	4.50351	2.46713	-0.868212	0.00160026	yes
Def8	chr8:123442955-123463899	7.27693	3.98671	-0.86813	0.000195236	yes
Lincppara,Mirlet7b,Mirle	chr15:85703772-85707524	4.55758	2.4974	-0.86784	0.003245	yes
Vrk1	chr12:106010262-106077410	39.1121	21.4376	-0.86747	0.000195236	yes
Rnaseh2a	chr8:84956609-84966011	19.7724	10.8519	-0.865538	0.000195236	yes
Fh1	chr1:175601377-175625635	170.168	93.4344	-0.864935	0.000195236	yes
Rps20	chr4:3834472-3835600	553.243	303.788	-0.864848	0.000195236	yes
Coprs	chr8:13884787-13890271	11.4604	6.29444	-0.864505	0.000195236	yes
Lmnb2	chr10:80901362-80918245	23.3883	12.8609	-0.86279	0.000195236	yes
Apex1	chr14:50924948-50930849	231.582	127.416	-0.861978	0.000195236	yes
Rpl7	chr1:16101295-16104433	297.869	163.943	-0.861479	0.000195236	yes
Det1	chr7:78827473-78847211	11.5632	6.36514	-0.861278	0.000195236	yes
Malsu1	chr6:49073794-49084717	35.4722	19.5412	-0.860168	0.000195236	yes
Emg1	chr6:124663103-124712178	140.149	77.231	-0.859713	0.000195236	yes
Vars2	chr17:35655634-35667592	6.70381	3.69429	-0.859683	0.000195236	yes
Galnt11	chr5:25222892-25265918	18.9774	10.4608	-0.859283	0.000195236	yes
Phldb1	chr9:44686307-44735198	7.43856	4.10094	-0.859067	0.000195236	yes
E030030I06Rik	chr10:22113049-22149270	3.09694	1.70741	-0.859036	0.0132116	yes

Psmb3	chr11:97703433-97713500	31.2093	17.2068	-0.858996	0.000195236	yes
Cenpb	chr2:131127411-131604824	87.9833	48.5186	-0.858691	0.0124526	yes
Atp13a2	chr4:140986872-141007701	33.8373	18.6711	-0.857807	0.000195236	yes
Ppp1r8	chr4:132826923-132843169	47.2173	26.0585	-0.857565	0.000195236	yes
Cln6	chr9:62838786-62875917	8.50086	4.69448	-0.856645	0.00271906	yes
Mrpl4	chr9:21002736-21008837	36.0589	19.9205	-0.856099	0.000195236	yes
Slc25a11	chr11:70644026-70647039	53.0331	29.3014	-0.855926	0.000195236	yes
Ndufa6	chr15:82350138-82354291	179.653	99.3016	-0.855323	0.000195236	yes
Tma7	chr9:109077987-109082381	29.0248	16.0478	-0.854913	0.000370691	yes
Chmp1a	chr8:123204260-123212788	23.5361	13.0174	-0.854436	0.000195236	yes
Tdrd5	chr1:156255295-156303606	4.92323	2.72333	-0.854232	0.000195236	yes
2810013P06Rik	chr8:123042574-123044602	2.87698	1.59151	-0.85416	0.00298992	yes
Hes6	chr1:91411482-91413222	11.4789	6.35143	-0.853829	0.000195236	yes
Cux1	chr5:136248134-136567490	50.4303	27.9073	-0.853646	0.000195236	yes
Cdk20	chr13:64432552-64439721	2.9623	1.64014	-0.852898	0.00130921	yes
Rsad1	chr11:94539797-94549207	1.68877	0.935054	-0.852854	0.000698636	yes
Gypc	chr18:32528319-32560034	2.30314	1.27593	-0.852056	0.00130921	yes
Trappc4	chr9:44403758-44407548	33.3107	18.4685	-0.850921	0.000195236	yes
Fbxw4	chr19:45560614-45660193	10.4988	5.82276	-0.850443	0.0321137	yes
Dynlt1b	chr17:6430111-6436295	1.96393	1.09097	-0.848127	0.0334032	yes
Ttc39a	chr4:109407099-109444745	2.75307	1.53006	-0.847453	0.000855787	yes
Gm9079	chr10:122078111-122079879	1.06717	0.59324	-0.847105	0.0372338	yes
Pycr2	chr1:180904273-180908088	45.3631	25.2175	-0.847095	0.000195236	yes
Dhx34	chr7:16197220-16222032	5.43244	3.02089	-0.846624	0.000195236	yes
Fxyd5	chr7:31032722-31042331	801.911	446.02	-0.846335	0.00298992	yes
Slc2a4rg-ps	chr2:181384249-181387596	0.846002	0.470767	-0.845649	0.00836289	yes
Hist1h3g	chr13:23535417-23535909	527.576	293.66	-0.845232	0.000195236	yes
Gpr155	chr2:73341505-73386480	2.85628	1.59037	-0.844775	0.000370691	yes
Ptprv	chr1:135108497-135132575	0.606209	0.337896	-0.843237	0.00188078	yes
Slmo1	chr18:67464848-67480581	1.1867	0.661511	-0.843113	0.00188078	yes
Rhd	chr4:134864535-134896172	5.18675	2.89329	-0.842123	0.000370691	yes
Cox6a1	chr5:115345653-115348955	260.646	145.401	-0.842053	0.000195236	yes
G630025P09Rik	chr11:69803594-69806038	2.02233	1.12853	-0.841581	0.00244513	yes
Thyn1	chr9:26999676-27030094	75.015	41.8642	-0.841463	0.00541779	yes
Aacs	chr5:125475872-125517403	12.8512	7.17323	-0.841208	0.000195236	yes
Ndufaf6	chr4:11051045-11076205	18.2684	10.2005	-0.840705	0.000195236	yes
Kdelc2	chr9:53384022-53401867	24.5984	13.7374	-0.840451	0.000195236	yes
Snx24	chr18:53245661-53390825	12.709	7.09937	-0.840089	0.000195236	yes
Tmem109	chr19:10870660-10881743	35.9853	20.1133	-0.839262	0.000195236	yes
Chrac1	chr15:73090411-73094075	65.0456	36.3568	-0.839224	0.000195236	yes
Mif	chr10:75859352-75860250	499.227	279.083	-0.839001	0.000195236	yes
5830415F09Rik	chr4:46376974-46389423	4.65913	2.60562	-0.838434	0.000537301	yes
Nfkbil1	chr17:35220174-35235815	20.3796	11.4034	-0.837661	0.000195236	yes
Cox4i1	chr8:120668224-120674209	645.336	361.195	-0.837273	0.000195236	yes
Ngdn	chr14:55015453-55024137	45.1213	25.271	-0.836328	0.000195236	yes
Bahcc1	chr11:120232946-120292297	0.848696	0.475397	-0.836116	0.000195236	yes
Apoe	chr7:19696243-19699188	2.9475	1.6518	-0.835456	0.00311626	yes
Hps1	chr19:42755195-42779976	11.1808	6.26631	-0.835341	0.000195236	yes
Armc9	chr1:86154779-86278284	4.72298	2.6471	-0.835283	0.000370691	yes
Lrwd1	chr5:136123065-136136074	27.2671	15.2833	-0.835202	0.000195236	yes
C330027C09Rik	chr16:48994187-49019705	86.7247	48.616	-0.835013	0.000195236	yes
Ivd	chr2:118861999-118881357	15.4328	8.65178	-0.834936	0.000195236	yes
Smarcal1	chr1:72583250-72636790	7.62523	4.27499	-0.83486	0.000195236	yes
Gtf2h4	chr17:35667727-35673743	18.3713	10.2997	-0.834854	0.000195236	yes
Syt7	chr19:10389089-10453181	0.520911	0.292098	-0.834584	0.00174394	yes
Arsb	chr13:93771678-93943016	27.4148	15.3829	-0.833622	0.000195236	yes
Usp5	chr6:124815018-124829447	41.6192	23.3621	-0.833081	0.000195236	yes
Rad23a	chr8:84832281-84840665	25.7315	14.4604	-0.831427	0.00160026	yes
Nme6	chr9:109832793-109842961	15.9154	8.95178	-0.830175	0.000195236	yes
Mis18a	chr16:90719311-90727371	38.7393	21.7973	-0.829649	0.000195236	yes
H2-M3	chr17:37270233-37274485	26.6664	15.0069	-0.8294	0.000195236	yes
Mrps6,Slc5a3	chr16:92058321-92112227	100.902	56.788	-0.829301	0.00602767	yes
Klhl23	chr2:69822369-69836651	3.18756	1.79428	-0.829045	0.000370691	yes
Tmem115	chr9:107533944-107538656	10.9377	6.15701	-0.829011	0.000195236	yes
Rbks	chr5:31624438-31697610	7.93313	4.4665	-0.828745	0.00100977	yes

Cep131	chr11:120064429-120086827	1.83603	1.0339	-0.828487	0.000195236	yes
Zfp647	chr15:76910369-76925448	0.591982	0.333427	-0.828181	0.0178389	yes
C1ra	chr6:124512620-124523440	1.84092	1.03712	-0.827846	0.000855787	yes
Fahd1	chr17:24848895-24850302	15.9559	8.9899	-0.827716	0.000195236	yes
Smug1	chr15:103153289-103163284	3.09186	1.74276	-0.827102	0.00100977	yes
Ncapg	chr5:45669924-45857540	86.6964	48.8736	-0.826918	0.000195236	yes
Pmepa1	chr2:173224464-173276533	7.29407	4.11251	-0.826707	0.000195236	yes
Haus5	chr7:30653707-30664994	3.2805	1.85041	-0.826073	0.00130921	yes
Nme1	chr11:93958924-93968521	163.768	92.4113	-0.825515	0.000195236	yes
Cenpe	chr3:135212562-135273540	38.0516	21.4792	-0.825013	0.000195236	yes
Brca2	chr5:150522620-150570146	7.60468	4.29305	-0.824885	0.000195236	yes
Mbp	chr18:82475122-82585637	23.3073	13.1588	-0.824753	0.000195236	yes
Ing1	chr8:11555761-11563251	20.8915	11.7988	-0.824274	0.000195236	yes
Sh3tc1	chr5:35697179-35729276	9.30207	5.25517	-0.823815	0.000195236	yes
Lemd2	chr17:27189599-27204438	12.5348	7.08236	-0.823639	0.000195236	yes
Pogk	chr1:166379166-166409828	29.113	16.4526	-0.823343	0.000195236	yes
Zfp932	chr5:109996526-110010411	7.87961	4.45543	-0.822561	0.000195236	yes
Adarb1	chr10:77290726-77418273	2.68817	1.52021	-0.822346	0.000195236	yes
Sh2d5	chr4:138250410-138260968	0.564099	0.319065	-0.822097	0.0173206	yes
Mcm7	chr5:138164588-138171862	38.8573	21.9787	-0.822081	0.000195236	yes
Exosc8	chr3:54728678-54735364	61.2711	34.6584	-0.822001	0.000195236	yes
Tmem138	chr19:10570477-10590041	4.86527	2.75238	-0.821838	0.0386168	yes
Zfyve21	chr12:111814169-111828388	7.03692	3.98256	-0.821248	0.000537301	yes
Mrpl40	chr16:18872017-18876637	52.9832	29.9879	-0.821154	0.000195236	yes
Phf7	chr14:31237695-31251218	5.81229	3.29233	-0.819997	0.000195236	yes
Zkscan14	chr5:145194945-145201882	4.80494	2.72299	-0.819327	0.000370691	yes
Ptafr	chr4:132564066-132580866	3.99893	2.2666	-0.819083	0.00116073	yes
Erh	chr12:80634022-80643861	74.0169	41.9634	-0.818722	0.000195236	yes
Als2cl	chr9:110880173-110900530	1.25794	0.713844	-0.817381	0.000195236	yes
Dmap1	chr4:117674685-117682225	15.8688	9.00598	-0.817243	0.000195236	yes
Dnaaf3	chr7:4522956-4532442	2.14611	1.21919	-0.815801	0.00160026	yes
Dpm3	chr3:89266460-89267079	52.1621	29.634	-0.815748	0.000195236	yes
Narfl	chr17:25773775-25785586	13.1451	7.4687	-0.815596	0.000537301	yes
Hdac9	chr12:34047581-34917095	17.8334	10.1331	-0.815502	0.000195236	yes
Hist1h2ak	chr13:21753376-21753912	1544.24	877.987	-0.814627	0.000195236	yes
Lrrc45	chr11:120713952-120721127	9.69487	5.51253	-0.814508	0.000195236	yes
Fabp4	chr3:10204342-10208576	1.98306	1.1278	-0.814221	0.040068	yes
Tmem97	chr11:78541816-78550735	110.044	62.5882	-0.814124	0.000195236	yes
Tomm20	chr8:126930663-126945921	14.2131	8.08557	-0.813801	0.000195236	yes
Klf16	chr10:80567120-80577296	7.81333	4.44547	-0.813603	0.000195236	yes
Sarnp	chr10:128821770-128877638	9.01447	5.13268	-0.81253	0.000370691	yes
Arhgef4	chr1:34801721-34812754	1.51059	0.860362	-0.812098	0.00202512	yes
Rassf2	chr2:131992849-132029988	12.3135	7.01467	-0.811793	0.000195236	yes
Ubtf	chr11:102304562-102319096	60.1405	34.2688	-0.811441	0.000195236	yes
Sox12	chr2:152393611-152398046	0.589474	0.335952	-0.811176	0.00813362	yes
Gpaa1	chr15:76331293-76334899	23.4971	13.3966	-0.810614	0.000195236	yes
Myeov2	chr1:92637144-92641985	243.188	138.691	-0.810202	0.000195236	yes
Eid2b	chr7:28277705-28280129	3.10123	1.76887	-0.810013	0.00130921	yes
3300005D01Rik	chr17:5798656-5803242	17.2342	9.83066	-0.809916	0.000370691	yes
Zfp953	chr13:67339308-67360572	5.06431	2.88892	-0.809836	0.000195236	yes
Stard7	chr2:127270228-127298934	76.8837	43.8586	-0.809818	0.000370691	yes
Tgm4	chr9:123034740-123067558	2.44702	1.39592	-0.809805	0.00130921	yes
Rpl5	chr5:107900527-107987077	23.9041	13.643	-0.8091	0.0300914	yes
Trp53rkb	chr2:166793766-166799492	1.69016	0.964796	-0.808867	0.000537301	yes
Tyms-ps	chr10:87966670-87968017	7.16403	4.0899	-0.808704	0.000537301	yes
Ppp1r26	chr2:28447940-28455508	2.62489	1.49858	-0.808661	0.000195236	yes
Plcd3	chr11:103070295-103101658	3.28592	1.87627	-0.808428	0.000195236	yes
Nrf1	chr6:30047987-30153458	19.2089	10.9686	-0.808391	0.000195236	yes
Csrp2	chr10:110920175-110939514	1.85825	1.06137	-0.808016	0.0384504	yes
Wdr89	chr12:75630593-75669537	793.149	453.066	-0.807872	0.00441531	yes
Coq4	chr2:29788262-29797743	6.78823	3.8782	-0.807648	0.000195236	yes
Gnptab	chr10:88379411-88447329	45.9567	26.2841	-0.806081	0.000195236	yes
Zbtb42	chr12:112678839-112682747	1.62725	0.931034	-0.805529	0.00202512	yes
Map4k1	chr7:28982853-29003278	9.30913	5.32726	-0.805254	0.000195236	yes
Rpl32	chr6:115774556-115808743	635.023	363.446	-0.805066	0.000195236	yes

Tns4	chr11:99065677-99089306	0.739726	0.423572	-0.804383	0.00130921	yes
Nmnat3	chr9:98296582-98411428	4.80621	2.75221	-0.804308	0.00116073	yes
B4gat1	chr19:5038825-5041134	11.221	6.42716	-0.803951	0.000195236	yes
Zdhhc14	chr17:5492599-5753891	7.37449	4.22528	-0.803495	0.000195236	yes
Actl6a	chr3:32708545-32726971	67.6701	38.7736	-0.803444	0.000195236	yes
Fam78a	chr2:32066884-32083705	4.47629	2.56503	-0.80333	0.000195236	yes
Asap3	chr4:136206364-136246573	0.468416	0.268481	-0.80297	0.00416625	yes
Dbi	chr1:120113279-120121096	354.997	203.598	-0.802087	0.000195236	yes
Dph2	chr4:117888642-117892003	6.91102	3.96371	-0.802046	0.000195236	yes
Gtpbp3	chr8:71488102-71493400	10.1053	5.79728	-0.801666	0.000195236	yes
Cpsf1	chr15:76576358-76607591	26.5974	15.2621	-0.801333	0.000195236	yes
Ndufaf3	chr9:108565864-108567342	19.8789	11.4071	-0.80131	0.000370691	yes
Mmp19	chr10:128790909-128804370	1.83902	1.05567	-0.800785	0.0217808	yes
Zfp706	chr15:36997026-37007402	28.6111	16.425	-0.800684	0.000195236	yes
Slc27a1	chr8:71568926-71586708	33.172	19.0501	-0.800165	0.000195236	yes
Bub1	chr2:127800199-127831859	74.1684	42.6241	-0.799136	0.000195236	yes
Zbtb22	chr17:33916175-33919325	11.273	6.48234	-0.798281	0.000195236	yes
Iscu	chr5:113772811-113778282	42.8514	24.6439	-0.798114	0.000195236	yes
Rab31	chr17:65651725-65772752	161.081	92.691	-0.797287	0.000195236	yes
Spryd4	chr10:128194634-128211794	10.0501	5.78402	-0.797072	0.00100977	yes
Med30	chr15:52712444-52730431	68.3722	39.3562	-0.796819	0.000195236	yes
C1d	chr11:17257617-17269176	29.0042	16.6998	-0.796426	0.000195236	yes
Nrde2	chr12:100125449-100159653	10.1953	5.87223	-0.795924	0.000195236	yes
Tnk2	chr16:32644642-32683493	5.39397	3.10856	-0.795101	0.000195236	yes
Sdhd	chr9:50596339-50603849	210	121.127	-0.793866	0.000195236	yes
Irf2bp2	chr8:126588295-126593436	25.6302	14.7948	-0.792751	0.000195236	yes
Mvb12b	chr2:33729955-33887946	6.82736	3.94111	-0.792724	0.000195236	yes
Nup43	chr10:7667503-7678886	50.1533	28.954	-0.792581	0.000195236	yes
Fbxo6	chr4:148145715-148152135	10.8906	6.28987	-0.791979	0.000195236	yes
Rplp0	chr5:115559466-115563729	369.002	213.118	-0.791975	0.000195236	yes
Dus3l	chr17:56764750-56770093	25.5762	14.7731	-0.79183	0.000195236	yes
E130012A19Rik	chr11:97627386-97629716	1.95378	1.12856	-0.791784	0.00675438	yes
Mars	chr10:127290792-127311786	61.932	35.7775	-0.791631	0.000195236	yes
Arhgap22	chr14:33216822-33369936	3.73957	2.16046	-0.791535	0.000698636	yes
Ccdc22	chrX:7558561-7605420	32.7446	18.919	-0.791424	0.000370691	yes
Timm10	chr2:84827020-84830213	64.1089	37.0477	-0.791141	0.000195236	yes
Atp5c1	chr2:10056030-10080510	375.424	217.005	-0.790794	0.000195236	yes
Poc1a	chr9:106281060-106349891	13.209	7.63757	-0.790333	0.000195236	yes
Mad2l1	chr6:66535467-66540991	89.8134	51.9348	-0.790229	0.000195236	yes
Tmem81	chr1:132506229-132508639	9.80262	5.67028	-0.789747	0.000537301	yes
Rbl2	chr8:91070056-91135494	7.97862	4.61531	-0.789713	0.00216236	yes
Lpxn	chr19:12798608-12833808	80.1201	46.3563	-0.789399	0.000195236	yes
Tifa	chr3:127789912-127798389	12.7171	7.36067	-0.788859	0.000537301	yes
BC030867	chr11:102248881-102265183	3.67292	2.12629	-0.788588	0.000370691	yes
Gpr151	chr18:42578019-42579652	0.985855	0.57073	-0.788569	0.0486308	yes
C030034I22Rik	chr17:69416446-69419192	1.99293	1.15376	-0.788539	0.00377278	yes
Nup35	chr2:80638811-80660071	7.43376	4.30737	-0.787284	0.000370691	yes
Pop5	chr5:115235850-115240970	60.9025	35.2893	-0.787271	0.000195236	yes
2810428I15Rik	chr8:70504295-70506739	108.321	62.7685	-0.787194	0.000195236	yes
Atp6v0e	chr17:26676395-26699646	218.273	126.503	-0.786963	0.000195236	yes
Klhl6	chr16:19946491-19983049	54.9297	31.8369	-0.786889	0.000195236	yes
Ppp1r35	chr5:137778917-137780107	27.0735	15.6952	-0.786555	0.000537301	yes
Mcm8	chr2:132816140-132844188	18.9722	11.0003	-0.78634	0.000195236	yes
Gna11	chr10:81528731-81545046	3.52553	2.0443	-0.786236	0.000195236	yes
Rpl35a	chr16:33056452-33060188	20.9429	12.1448	-0.786124	0.00100977	yes
Crocc	chr4:141016636-141060545	1.92303	1.11526	-0.786	0.000370691	yes
Parp8	chr13:116854823-117025516	2.83438	1.645	-0.784944	0.000855787	yes
Eif1ad	chr19:5364632-5371511	55.9753	32.5098	-0.783914	0.00529192	yes
Cd300lb	chr11:114922780-114934386	62.8624	36.5208	-0.783478	0.000195236	yes
Kansl2	chr15:98517657-98534269	5.25971	3.05676	-0.782981	0.000537301	yes
Fem1a	chr17:56256792-56263608	16.6735	9.6929	-0.782559	0.000195236	yes
Kxd1	chr8:70513395-70523180	24.9029	14.479	-0.782352	0.000195236	yes
Pts	chr9:50521616-50528641	43.7691	25.4527	-0.782092	0.000195236	yes
Trub1	chr19:57452905-57491005	9.89059	5.75202	-0.781989	0.000195236	yes
Cul7	chr17:46650337-46664364	0.948231	0.551558	-0.781726	0.00311626	yes

Clybl	chr14:122181693-122451146	12.4262	7.23222	-0.780874	0.000195236	yes
Dnajb14	chr3:137867674-137908931	23.316	13.5729	-0.78059	0.000195236	yes
Ckap5	chr2:91546321-91620665	59.2014	34.4667	-0.780427	0.000195236	yes
Lsm4	chr8:70673230-70678752	232.679	135.468	-0.780392	0.000195236	yes
Ankrd39	chr1:36538172-36547201	4.49841	2.61912	-0.780333	0.00188078	yes
Poldip2	chr11:78512295-78522736	32.8169	19.1155	-0.779701	0.000195236	yes
Tmem176a	chr6:48841482-48845364	17.2908	10.0751	-0.779214	0.000370691	yes
Spn	chr7:127132231-127137823	16.5363	9.63661	-0.77904	0.000537301	yes
Depdc7	chr2:104721786-104742801	19.8022	11.5412	-0.778869	0.000195236	yes
Klhl22	chr16:17759620-17793382	10.9734	6.3956	-0.778856	0.000195236	yes
Mlh1	chr9:111228227-111271608	21.9104	12.774	-0.778406	0.000195236	yes
Nfatc1	chr18:80606204-80713071	29.9235	17.4478	-0.778233	0.000195236	yes
Serf1	chr13:100108018-100114233	51.3403	29.9376	-0.778131	0.000195236	yes
Pdk1	chr2:71389259-71903858	44.6411	26.0457	-0.777328	0.0166035	yes
Mex3d	chr10:80380354-80387651	6.51294	3.80188	-0.776597	0.000370691	yes
U2af1l4	chr7:30563339-30565364	31.0623	18.1399	-0.775997	0.000195236	yes
Xrcc1	chr7:24547149-24573438	17.1646	10.0252	-0.7758	0.000195236	yes
Slc35b2	chr17:45564151-45567669	27.7042	16.1818	-0.775727	0.000370691	yes
2610015P09Rik	chr16:43889901-43964314	4.86847	2.84416	-0.775464	0.000195236	yes
Eml3	chr19:8929693-8941582	10.2567	5.99227	-0.775396	0.000195236	yes
Zfp951	chr5:104813167-104860068	3.68996	2.15664	-0.774822	0.00116073	yes
Ube2t	chr1:134962564-134974179	47.4946	27.7636	-0.774567	0.000195236	yes
Yipf1	chr4:107314362-107359823	17.6849	10.3387	-0.774472	0.000195236	yes
Ephb2	chr4:136653609-136836011	1.10246	0.644602	-0.774239	0.00188078	yes
Klhl25	chr7:75848337-75874130	7.23073	4.22815	-0.774115	0.000370691	yes
Bank1	chr3:136053363-136326046	7.35105	4.3013	-0.773176	0.000195236	yes
Rcc1	chr4:132331918-132345750	50.4475	29.5202	-0.773081	0.000195236	yes
Acot13	chr13:24817954-24831489	50.3456	29.4788	-0.772186	0.000195236	yes
Mbnl1	chr3:60472829-60629748	276.438	161.908	-0.771785	0.00100977	yes
Polh	chr17:46171992-46202625	36.4835	21.3712	-0.771577	0.000195236	yes
Arrb1	chr7:99535485-99606771	20.0801	11.7635	-0.771452	0.000195236	yes
Lrrc27	chr7:139103637-139242973	1.64031	0.961016	-0.77134	0.00467182	yes
Nxt1	chr2:148672614-148676026	83.1763	48.7349	-0.771217	0.000195236	yes
Atxn7l3	chr11:102284819-102296629	12.1749	7.13462	-0.770996	0.00541779	yes
Brk1	chr6:113604771-113616951	93.5778	54.846	-0.770781	0.000195236	yes
Uqcrc1	chr9:108936647-108949641	169.914	99.5914	-0.770715	0.000195236	yes
Tmem59	chr4:107178629-107200996	96.3112	56.4812	-0.769934	0.000195236	yes
Loh12cr1	chr6:134639511-134711184	6.38569	3.74533	-0.769752	0.00230418	yes
Mat2b	chr11:40679313-40695203	41.5001	24.3551	-0.768891	0.000195236	yes
Ifitm6	chr7:141015811-141016892	20.829	12.2277	-0.768443	0.00351242	yes
Sumo3	chr10:77606096-77618331	86.6917	50.9031	-0.768141	0.000195236	yes
Nup210	chr6:91013066-91116826	17.2435	10.1278	-0.767735	0.000195236	yes
Tmeff1	chr4:48585192-48663131	1.51764	0.891701	-0.767198	0.0124526	yes
Fcgrt	chr7:45092992-45103822	2.62654	1.54374	-0.766734	0.00504708	yes
Gid4	chr11:60417144-60445277	12.4447	7.31552	-0.766495	0.000195236	yes
Dnajc22	chr15:99099483-99104707	2.18011	1.28161	-0.76644	0.0122489	yes
Ttc30b	chr2:75935849-75938462	0.936585	0.550631	-0.766324	0.0159005	yes
Serpinb6b	chr13:32965512-32979037	14.9701	8.80265	-0.766078	0.000370691	yes
Fut10	chr8:31187330-31261924	4.92144	2.89445	-0.76579	0.000370691	yes
Hmgn1	chr16:96121587-96127725	266.742	156.895	-0.765646	0.000195236	yes
Zfp689	chr7:127442135-127449158	1.00357	0.590315	-0.765592	0.00517183	yes
Nrros	chr16:32142824-32165476	43.4672	25.5704	-0.765453	0.000195236	yes
Pak1	chr7:97842938-97912381	73.3039	43.1241	-0.765394	0.000195236	yes
Ssc4d	chr5:135960222-135974476	0.707544	0.416317	-0.765139	0.0242828	yes
Pin1	chr9:20652129-20666584	15.7281	9.2561	-0.764869	0.000195236	yes
Gart	chr16:91621396-91646972	85.1716	50.1253	-0.764835	0.000195236	yes
Snta1	chr2:154376313-154408084	4.20441	2.47463	-0.764691	0.00216236	yes
Timm21	chr18:84935024-84981392	25.9492	15.2844	-0.763628	0.000537301	yes
Acox3	chr5:35581226-35613801	3.33829	1.96647	-0.763499	0.000537301	yes
3110045C21Rik	chr1:169969408-170088944	1.73321	1.02171	-0.762462	0.0378993	yes
Zfp324	chr7:12965863-12973822	5.48945	3.23684	-0.762074	0.000370691	yes
Phka2	chrX:160502165-160598878	22.0023	12.9783	-0.761557	0.000195236	yes
Zfp444	chr7:6172512-6193104	2.83389	1.67171	-0.761462	0.00116073	yes
Arpc1b	chr5:145114255-145128186	349.622	206.253	-0.761382	0.000195236	yes
Pwp2	chr10:78170909-78185149	19.0197	11.2283	-0.760353	0.000195236	yes

Ttc30a1	chr2:75979105-75981967	0.767002	0.45287	-0.760134	0.0292744	yes
Sh3glb2	chr2:30344776-30359316	22.6345	13.3675	-0.7598	0.000195236	yes
Fkbp2	chr19:6977738-6980461	97.8771	57.8089	-0.759681	0.000195236	yes
Zfp873	chr10:82048126-82061586	2.09617	1.23817	-0.759547	0.00744706	yes
Gm561	chr2:144594064-144595365	29.9374	17.6896	-0.75905	0.00174394	yes
Tpcn1	chr5:120534156-120588613	10.9271	6.45808	-0.758735	0.000195236	yes
Esco2	chr14:65819026-65833969	45.0535	26.6278	-0.758708	0.000195236	yes
Pirb	chr7:3712504-3720382	1.9584	1.15767	-0.758456	0.00116073	yes
Irx2	chr13:72628977-72634194	16.7993	9.93125	-0.758354	0.000195236	yes
Cdca4	chr12:112820234-112829389	49.8512	29.473	-0.758236	0.000195236	yes
Car2	chr3:14886425-14900770	602.884	356.532	-0.757849	0.000195236	yes
A230050P20Rik	chr9:20868641-20879710	17.6601	10.4444	-0.757763	0.000370691	yes
Chchd1	chr14:20702011-20704425	128.298	75.958	-0.756225	0.000195236	yes
Ubtd1	chr19:41981762-42034641	13.9966	8.28726	-0.756107	0.000537301	yes
Ska1	chr18:74195298-74207818	19.684	11.6553	-0.756039	0.000370691	yes
Gm5617	chr9:48495342-48495975	7.41865	4.39296	-0.755963	0.00915655	yes
2010320M18Rik	chr8:70776861-70777606	7.30192	4.32409	-0.75588	0.00338276	yes
Pcyt2	chr11:120610086-120617890	22.2409	13.1719	-0.755753	0.000195236	yes
Atp5b	chr10:128083306-128090388	843.894	499.803	-0.755702	0.000195236	yes
Ttyh3	chr5:140620576-140649031	3.6725	2.17562	-0.755336	0.000537301	yes
Slc2a3	chr6:122727808-122742745	10.1901	6.0376	-0.755127	0.000195236	yes
Gsto1	chr19:47854988-47864788	89.2266	52.8752	-0.754884	0.000195236	yes
Mvk	chr5:114444268-114460590	12.449	7.37803	-0.754728	0.000195236	yes
B630019K06Rik	chrX:8892352-8894964	6.24829	3.70327	-0.75466	0.000698636	yes
Thoc6	chr17:23660522-23677449	29.6616	17.5817	-0.754517	0.00244513	yes
Rbm12b1	chr4:12140116-12146746	2.88926	1.71322	-0.753992	0.003245	yes
Ube2e3	chr2:78869046-78920583	37.6949	22.3576	-0.753599	0.000195236	yes
Sumo1	chr1:59639433-59670834	46.151	27.3833	-0.753066	0.000195236	yes
Lsm8	chr6:18848634-18854052	89.6619	53.2009	-0.753043	0.000370691	yes
Tagap	chr17:7925999-7934897	3.60011	2.13747	-0.752137	0.00100977	yes
Capn10	chr1:92933542-92950590	4.96742	2.95016	-0.751702	0.00216236	yes
Mrpl2	chr17:46646247-46650132	84.2475	50.0535	-0.751163	0.000195236	yes
Zfp85	chr13:67729261-67756838	3.09472	1.83959	-0.750425	0.00553759	yes
Dhcr24	chr4:106561037-106589113	13.825	8.21941	-0.75017	0.000195236	yes
Fam132b	chr1:91366429-91374217	0.79428	0.47224	-0.750126	0.0182627	yes
Chtf18	chr17:25717171-25727415	10.5986	6.30623	-0.749022	0.000698636	yes
Sdf2l1	chr16:17130137-17132383	127.602	75.9832	-0.747894	0.000195236	yes
Imp3	chr9:56937499-56938398	139.752	83.2374	-0.74757	0.000195236	yes
Rpp21	chr17:36255672-36257846	40.5958	24.1898	-0.746928	0.000370691	yes
Ndufb5	chr3:32737062-32751559	106.473	63.4857	-0.745986	0.000195236	yes
Pithd1	chr4:135975601-135987244	39.6559	23.6493	-0.745734	0.000195236	yes
Gm5113	chr7:30169921-30180209	1.48578	0.88614	-0.745615	0.0102954	yes
Rarg	chr15:102234937-102257522	2.63305	1.5709	-0.745143	0.00130921	yes
Papss1	chr3:131564767-131643671	24.6998	14.7379	-0.744967	0.000195236	yes
Spdl1	chr11:34809184-34833641	39.0059	23.2746	-0.744935	0.000195236	yes
Slc37a4	chr9:44396926-44402968	10.5832	6.31622	-0.744646	0.000698636	yes
Polg	chr7:79392337-79466273	18.9584	11.3161	-0.744464	0.00428914	yes
Oxnad1	chr14:32085727-32103203	20.6275	12.3128	-0.744409	0.000195236	yes
2010204K13Rik	chrX:7411816-7422988	21.3943	12.7711	-0.744345	0.00639665	yes
Gm20300	chr10:30603194-30606634	1.92529	1.14984	-0.74365	0.00504708	yes
Coq10a	chr10:128363096-128370037	3.14097	1.87616	-0.743423	0.00351242	yes
Ncaph	chr2:127103809-127133954	45.9995	27.4791	-0.743284	0.000195236	yes
Lrrc20	chr10:61475832-61582228	6.8747	4.10694	-0.743233	0.000370691	yes
Map3k6	chr4:133240817-133252928	0.788224	0.47091	-0.743155	0.00744706	yes
Grasp	chr15:101221190-101232756	0.766933	0.458217	-0.74307	0.0312726	yes
Josd1	chr15:79674249-79687872	19.0111	11.3651	-0.742227	0.000195236	yes
Spns1	chr7:126370059-126377934	15.2357	9.10988	-0.741952	0.000370691	yes
Gatb	chr3:85574128-85654470	22.3491	13.3652	-0.741738	0.000195236	yes
Tsen34	chr7:3693609-3701035	15.6499	9.35905	-0.741722	0.000698636	yes
Cep57l1	chr10:41718839-41809868	14.946	8.93816	-0.741706	0.000195236	yes
Sdc1	chr12:8771395-8793687	10.5562	6.31549	-0.741129	0.000195236	yes
Ndufa12	chr10:94199008-94220948	24.3109	14.5447	-0.741107	0.000537301	yes
Oat	chr7:132557474-132576398	92.7577	55.4986	-0.741014	0.000195236	yes
Timm23	chr14:32180165-32201891	14.4435	8.64213	-0.740962	0.000537301	yes
Thumpd2	chr17:81026326-81065085	7.9822	4.7765	-0.740832	0.000537301	yes

Mlf1	chr3:67374096-67400000	4.94116	2.95897	-0.739754	0.0036408	yes
Tcf19	chr17:35512734-35516824	28.1253	16.8447	-0.739573	0.000195236	yes
Csf3r	chr4:126024658-126044975	0.60246	0.360888	-0.739316	0.0135492	yes
Pim3	chr15:88862193-88865726	17.3519	10.3954	-0.739149	0.000698636	yes
AI839979	chr5:31569591-31571397	5.83121	3.49365	-0.739061	0.00271906	yes
Abhd16a	chr17:35089290-35102987	23.2098	13.9062	-0.739003	0.000195236	yes
Neurl3	chr1:36264601-36273425	47.557	28.4947	-0.738963	0.000195236	yes
Cecr5	chr6:120509493-120531299	9.1332	5.47289	-0.738817	0.000855787	yes
Rpl31	chr1:39367850-39478747	21.1657	12.6841	-0.738713	0.0293726	yes
Mrpl33	chr5:31613950-31622644	181.086	108.595	-0.737716	0.000195236	yes
Mdp1	chr14:55657878-55660508	14.0135	8.4042	-0.737633	0.000195236	yes
Pcbd2	chr13:55727367-55776830	33.0542	19.8263	-0.737423	0.000370691	yes
Mob2	chr7:142008552-142061034	17.74	10.6432	-0.737082	0.000698636	yes
Dok1	chr6:83030935-83033471	21.4373	12.8627	-0.736928	0.000195236	yes
Mettl7a1	chr15:100304816-100314348	4.64543	2.7875	-0.736839	0.00188078	yes
B3galt4	chr17:33949911-33951488	3.30949	1.98631	-0.736519	0.00428914	yes
Etfb	chr7:43444071-43457800	62.6042	37.6273	-0.734481	0.000195236	yes
B9d2	chr7:25681157-25686558	10.8801	6.54099	-0.734108	0.00116073	yes
Tmem222	chr4:133266044-133277790	26.2089	15.7568	-0.734084	0.000195236	yes
Tsc22d1	chr14:76415820-76507766	9.22882	5.54864	-0.734011	0.00188078	yes
Mogs	chr6:83115505-83118898	61.0362	36.6999	-0.733891	0.000195236	yes
Fgfbp3	chr19:36917549-36919599	2.45726	1.47842	-0.732996	0.0100707	yes
Tkt	chr14:30549130-30574726	243.319	146.406	-0.73287	0.000370691	yes
Ska3	chr14:57806560-57826163	20.6715	12.4485	-0.731675	0.000195236	yes
Kif20b	chr19:34922357-34975731	45.4079	27.3531	-0.731241	0.000195236	yes
G6pd2	chr5:61808842-61810477	1.04638	0.630555	-0.730717	0.0386168	yes
Rragb	chrX:153139957-153171943	10.5142	6.33637	-0.730607	0.000195236	yes
Prpsap2	chr11:61729649-61762088	8.98661	5.41987	-0.729518	0.00100977	yes
BC029214	chr2:25459487-25461094	2.75918	1.66475	-0.728936	0.0228548	yes
Ebpl	chr14:61339762-61360445	8.84001	5.33833	-0.727661	0.00145427	yes
Cycs	chr6:50562562-50566474	7.09881	4.28747	-0.727449	0.000698636	yes
Fam53a	chr5:33600352-33629635	15.1897	9.17496	-0.727324	0.000195236	yes
Cep128	chr12:90998491-91384409	11.0746	6.69099	-0.726963	0.000195236	yes
1500015A07Rik	chr18:61726389-61728253	3.07731	1.85923	-0.72696	0.00675438	yes
BC029722	chr2:155775343-155819203	19.101	11.5451	-0.726372	0.000370691	yes
Lrp1	chr10:127538157-127621148	19.7361	11.9314	-0.726075	0.000195236	yes
Slc6a13	chr6:121300295-121337718	8.09056	4.89177	-0.725883	0.000195236	yes
Ccdc101	chr7:126649308-126672779	26.8232	16.2221	-0.725521	0.000195236	yes
Ndufb7	chr8:83566757-83571623	188.896	114.256	-0.725325	0.000195236	yes
Vkorc1	chr7:127893062-127895617	45.0569	27.259	-0.725015	0.000698636	yes
Mrps18c	chr5:100798758-100804467	47.8137	28.9422	-0.724251	0.00100977	yes
Tlcd1	chr11:78178148-78180819	4.23777	2.56528	-0.724192	0.0269153	yes
Fbl	chr7:28169747-28179269	5.29011	3.20787	-0.721679	0.00504708	yes
Nsl1	chr1:191063020-191084558	19.1437	11.612	-0.721255	0.000195236	yes
Rcor3	chr1:192098545-192151025	7.52774	4.56926	-0.720258	0.00116073	yes
Fam102b	chr3:108970996-109027607	15.2546	9.25967	-0.720214	0.000195236	yes
Nop10	chr2:112261925-112262898	229.791	139.493	-0.720125	0.000195236	yes
Acp6	chr3:97158776-97176576	17.7224	10.7604	-0.719845	0.000370691	yes
Csnk1g2	chr10:80622779-80640771	51.3819	31.2012	-0.71966	0.000195236	yes
Pcgf1	chr6:83078389-83080855	5.35526	3.25196	-0.719645	0.00755508	yes
Dennd2c	chr3:103127555-103169733	6.01031	3.65237	-0.718606	0.000370691	yes
Zfand1	chr3:10339955-10351301	5.99862	3.64652	-0.718111	0.00174394	yes
Parl	chr16:20279820-20302362	40.0688	24.3621	-0.717842	0.000537301	yes
Fbxo5	chr10:5799157-5805465	91.6228	55.7146	-0.717651	0.000195236	yes
Trappc2l	chr8:122611625-122615591	46.3259	28.1776	-0.717271	0.000698636	yes
4632404H12Rik	chr3:89767510-89773400	2.54722	1.54972	-0.71691	0.0036408	yes
Pak4	chr7:28558818-28598184	4.07138	2.47721	-0.716803	0.000370691	yes
Fgd3	chr13:49263109-49309208	13.7612	8.37472	-0.71649	0.000370691	yes
Mknk2	chr10:80665317-80676293	25.9667	15.8036	-0.716406	0.000195236	yes
Pex10	chr4:155067029-155072406	14.6635	8.92491	-0.71632	0.000370691	yes
Ptpro	chr6:137252298-137464633	11.7141	7.13398	-0.715464	0.000537301	yes
Tctex1d2	chr16:32419701-32428892	29.1618	17.7604	-0.715413	0.00116073	yes
H2-DMa	chr17:34122831-34139101	3.33923	2.03392	-0.715254	0.00615568	yes
Osbpl3	chr6:50293326-50456170	10.3382	6.29906	-0.714776	0.000195236	yes
Mras	chr9:99385419-99436712	2.06707	1.25969	-0.714523	0.00188078	yes

Abhd12	chr2:150832514-150904731	48.6158	29.6277	-0.714478	0.000195236	yes
Cenph	chr13:100759685-100775899	20.6724	12.5984	-0.714468	0.000698636	yes
Mul1	chr4:138434671-138442265	4.67029	2.84666	-0.714241	0.00100977	yes
Aagab	chr9:63602654-63641889	29.9494	18.2611	-0.713756	0.000370691	yes
Zfp316	chr5:143249694-143270022	1.51193	0.922038	-0.713493	0.00188078	yes
Smim12	chr4:127243783-127247809	34.2831	20.9152	-0.712947	0.000855787	yes
Dvl1	chr4:155847316-155863353	15.7448	9.60886	-0.712435	0.000370691	yes
Hspb6	chr7:30553301-30555439	2.70089	1.64867	-0.712133	0.0148245	yes
Rcan1	chr16:92391950-92466169	33.1717	20.2578	-0.711475	0.000195236	yes
Gabarapl2	chr8:111940702-111952915	11.8662	7.24861	-0.711076	0.00377278	yes
Mrps28	chr3:8802145-8923857	131.319	80.2291	-0.710879	0.000195236	yes
Cit	chr5:115845655-116006341	3.76925	2.30398	-0.710147	0.000537301	yes
Zfp710	chr7:80024813-80092751	33.3282	20.3754	-0.709914	0.000195236	yes
Mrpl30	chr1:37890552-37898333	103.058	63.006	-0.709897	0.000370691	yes
Cisd1	chr10:71330493-71344849	63.8851	39.0589	-0.709829	0.000195236	yes
Osbpl8	chr10:111164801-111297247	89.2685	54.5923	-0.709453	0.000698636	yes
Calm3	chr7:16915378-16924032	191.975	117.408	-0.709388	0.000370691	yes
Dbf4	chr5:8396968-8422716	58.3665	35.6988	-0.709265	0.000195236	yes
Lmnb1	chr18:56707812-56753424	102.283	62.5668	-0.709101	0.000195236	yes
Nacc2	chr2:26055535-26122811	0.509145	0.311493	-0.708875	0.0154682	yes
Ankrd24	chr10:81628539-81647612	1.62737	0.99574	-0.7087	0.00216236	yes
Alyref	chr11:120594515-120598365	340.185	208.181	-0.708482	0.000195236	yes
Coro1a	chr7:126699773-126704816	256.45	156.969	-0.708196	0.00100977	yes
Xrcc6	chr15:82016368-82040084	43.9398	26.9174	-0.706989	0.000195236	yes
Coq9	chr8:94838416-94854895	28.3361	17.3626	-0.706655	0.000195236	yes
Mtx1	chr3:89209080-89214335	45.7564	28.0444	-0.706261	0.000195236	yes
Fads2	chr19:10064163-10101503	107.187	65.7104	-0.705938	0.000370691	yes
Nhp2	chr11:51619772-51635896	275.234	168.731	-0.705935	0.000195236	yes
Herc3	chr6:58833699-58920396	7.5996	4.65945	-0.705765	0.000370691	yes
Zkscan3	chr13:21387003-21402755	10.0121	6.14321	-0.704685	0.000698636	yes
Dcps	chr9:35046578-35175987	48.3337	29.6689	-0.704079	0.00950212	yes
Arl6ip1	chr7:118118889-118129625	204.65	125.638	-0.70388	0.000537301	yes
Mrps14	chr1:160195259-160201186	53.8536	33.0646	-0.703754	0.000537301	yes
Pagr1a	chr7:127015050-127017352	33.8255	20.7703	-0.703594	0.000195236	yes
Arhgap39	chr15:76723984-76818170	5.1621	3.1699	-0.703518	0.000537301	yes
D5Ertd579e	chr5:36600485-36696021	13.6708	8.39567	-0.703384	0.000195236	yes
Snx15	chr19:6119403-6128215	5.77592	3.54735	-0.703308	0.0036408	yes
Atp5sl	chr7:25619413-25625550	14.8228	9.10367	-0.703294	0.000855787	yes
Zc3hc1	chr6:30366387-30391010	17.6824	10.8601	-0.703272	0.000537301	yes
Tmem70	chr1:16665190-16678275	55.8899	34.3372	-0.702816	0.000370691	yes
Mrpl37	chr4:107055873-107066866	36.5026	22.4365	-0.702148	0.000537301	yes
Ifngr1	chr10:19591957-19610225	31.8718	19.5907	-0.702109	0.000195236	yes
Fxyd2	chr9:45399708-45410278	370.471	227.729	-0.702043	0.000855787	yes
Zfp383	chr7:29908516-29916813	2.19112	1.34703	-0.701881	0.0163026	yes
BC017158	chr7:128265696-128298131	7.38874	4.54453	-0.701197	0.00174394	yes
Traip	chr9:107950962-107972268	11.6703	7.1803	-0.700726	0.000537301	yes
Fam20c	chr5:138755080-138810063	13.8965	8.55256	-0.700296	0.00100977	yes
Ccdc12	chr9:110656502-110711593	24.5895	15.1341	-0.700247	0.000537301	yes
Col18a1	chr10:77052178-77166530	1.2622	0.776917	-0.700111	0.00230418	yes
Mir6236	chr9:110281286-110281409	2651.41	1633.79	-0.698536	0.00744706	yes
Tpd52l2	chr2:181497141-181517962	22.273	13.727	-0.698284	0.000370691	yes
Ceacam19	chr7:19875741-19887965	0.705534	0.434856	-0.698177	0.0179488	yes
2310067B10Rik	chr11:115765432-115799033	4.05227	2.49829	-0.697789	0.000537301	yes
Lfng	chr5:140607340-140615545	29.0387	17.9039	-0.697704	0.000195236	yes
Ndufa10	chr1:92439718-92473758	150.491	92.8204	-0.697162	0.000370691	yes
BC037704	chr19:43675177-43677170	3.37221	2.08213	-0.695635	0.00675438	yes
Mfng	chr15:78755882-78773445	25.6223	15.8247	-0.695226	0.000855787	yes
Acad8	chr9:26974138-26999549	11.9183	7.36564	-0.694296	0.000537301	yes
Hacl1	chr14:31607225-31640965	2.70327	1.67067	-0.69428	0.00766958	yes
Fads1	chr19:10182887-10196872	69.7424	43.1077	-0.69409	0.000195236	yes
Fancl	chr11:26387083-26593920	27.1582	16.7866	-0.694074	0.0133195	yes
A730008H23Rik,Hjurp	chr1:88227019-88277579	92.1609	57.0338	-0.692338	0.00428914	yes
Ufc1	chr1:171288563-171294982	48.7573	30.1744	-0.692293	0.000698636	yes
Selplg	chr5:113817797-113830501	7.75268	4.79814	-0.692221	0.00160026	yes
Syne1	chr10:5020195-5194707	3.93926	2.43948	-0.691353	0.000698636	yes

Zfp740	chr15:102203644-102215610	17.9283	11.105	-0.691028	0.000195236	yes
Sae1	chr7:16320235-16387896	110.307	68.3347	-0.690831	0.000195236	yes
Ttc12	chr9:49436960-49486225	7.62947	4.7282	-0.690291	0.000698636	yes
Scrn3	chr2:73312651-73337807	11.2521	6.97366	-0.690203	0.00130921	yes
Arhgef10l	chr4:140514484-140665905	18.3857	11.3958	-0.690085	0.000370691	yes
Klrg2	chr6:38626659-38637239	8.79221	5.45081	-0.689755	0.00116073	yes
Ptgr2	chr12:84285295-84315832	23.9491	14.853	-0.689222	0.000195236	yes
Jam2	chr16:84774122-84826375	0.823398	0.510727	-0.689038	0.0122489	yes
Zfand2b	chr1:75168645-75171626	15.5546	9.64874	-0.688933	0.00174394	yes
Slc25a5	chrX:36795596-36798808	569.668	353.49	-0.688452	0.000195236	yes
Gm17296	chr8:126426651-126475065	5.31996	3.30206	-0.688049	0.00145427	yes
Man1b1	chr2:25332742-25352213	16.9913	10.5464	-0.688048	0.000195236	yes
Rps6ka5	chr12:100549777-100725028	11.484	7.12933	-0.687783	0.000370691	yes
Pla2g4a	chr1:149829618-149961290	213.46	132.528	-0.687669	0.000370691	yes
Acap3	chr4:155891874-155907251	6.9007	4.28512	-0.687408	0.000698636	yes
Lysmd2	chr9:75625731-75637773	2.05581	1.27672	-0.687263	0.0403132	yes
Tmem194b	chr1:52630704-52651919	6.45603	4.01395	-0.685624	0.00188078	yes
5730409E04Rik	chr4:126609853-126614371	5.26607	3.27512	-0.68518	0.00130921	yes
Cenpi	chrX:134308083-134363104	28.7305	17.8699	-0.685055	0.000537301	yes
B330016D10Rik	chr4:141546161-141548313	2.1196	1.31855	-0.684837	0.0153646	yes
Dgat1	chr15:76502014-76511818	5.90405	3.67301	-0.684743	0.00847587	yes
Prmt3	chr7:49778357-49858265	52.3205	32.5705	-0.683812	0.000195236	yes
Sfxn3	chr19:45047575-45056383	3.11674	1.94049	-0.683614	0.00311626	yes
Mlec	chr5:115142980-115158176	88.7382	55.2524	-0.683518	0.000195236	yes
Esrra	chr19:6909697-6921808	18.1798	11.322	-0.6832	0.00652015	yes
Nradd	chr9:110621134-110624393	4.12964	2.57322	-0.682442	0.014404	yes
Lonrf3	chrX:36328408-36366856	12.4884	7.78383	-0.682032	0.000195236	yes
Sema6b	chr17:56123084-56140343	14.3133	8.92142	-0.682012	0.000370691	yes
Map3k14	chr11:103219763-103267401	4.43122	2.76216	-0.681906	0.000370691	yes
Rce1	chr19:4622550-4625617	23.7156	14.7869	-0.681515	0.000698636	yes
Parp1	chr1:180568974-180601254	129.25	80.6077	-0.681178	0.00116073	yes
Cog2	chr8:124520766-124552007	8.64523	5.39361	-0.680653	0.000698636	yes
Vps33b	chr7:80269654-80291579	4.76614	2.97429	-0.680277	0.00145427	yes
Aaed1	chr13:64291835-64312710	16.3631	10.2129	-0.680055	0.00258324	yes
Tmpo	chr10:91147570-91171619	238.838	149.081	-0.679936	0.00258324	yes
Zfp763	chr17:33016863-33033381	4.26681	2.66414	-0.679489	0.00338276	yes
Etfa	chr9:55454435-55512243	29.4144	18.3716	-0.679041	0.00230418	yes
Cbx6,Npcd,Nptxr	chr15:79786350-79834333	12.0302	7.51571	-0.678684	0.000698636	yes
Il17rc	chr6:113471454-113483163	4.16582	2.60287	-0.678499	0.00996804	yes
Atp5g3	chr2:73908446-73911326	440.181	275.054	-0.678386	0.000537301	yes
Slc37a2	chr9:37229148-37255738	11.4109	7.13132	-0.678173	0.000855787	yes
Incenp	chr19:9872296-9899533	67.2493	42.0284	-0.678156	0.000537301	yes
Rdh13	chr7:4425664-4445657	10.2771	6.42401	-0.677883	0.000698636	yes
Hdgf	chr3:87906320-87916132	254.709	159.22	-0.677826	0.000698636	yes
Ppan	chr9:20888174-20892179	73.2321	45.7799	-0.677763	0.000195236	yes
Dedd2	chr7:25202839-25219859	5.75566	3.59818	-0.677713	0.00517183	yes
Exosc6	chr8:111056338-111057664	94.4304	59.053	-0.677241	0.000537301	yes
Rasa3	chr8:13567217-13677587	37.2289	23.3024	-0.675943	0.000537301	yes
Snrpe	chr1:133603870-133610280	162.767	101.883	-0.675897	0.000537301	yes
Sall2	chr14:52311176-52328670	0.773375	0.484178	-0.67563	0.0110431	yes
Zfp180	chr7:24081896-24107708	10.574	6.62113	-0.675368	0.000370691	yes
Vamp1	chr6:125215580-125222306	2.21364	1.38615	-0.675331	0.00744706	yes
Numb	chr12:83794033-83921934	25.9843	16.2721	-0.675238	0.000195236	yes
Lincpint	chr6:31093562-31218474	5.17427	3.24158	-0.674658	0.0273047	yes
Cd81	chr7:143021783-143067930	111.972	70.1497	-0.674636	0.000195236	yes
Daglb	chr5:143464492-143504442	13.7925	8.64432	-0.674063	0.000370691	yes
Hyal2	chr9:107569162-107572778	10.2003	6.39452	-0.673709	0.00216236	yes
Pus1	chr5:110773666-110780615	35.982	22.5596	-0.673537	0.000195236	yes
Calr3	chr8:72424182-72443778	1.83763	1.15251	-0.673071	0.0299428	yes
H6pd	chr4:149979473-150009023	8.8016	5.52331	-0.672233	0.000537301	yes
Mrps9	chr1:42851232-42905683	62.5026	39.2288	-0.672004	0.000195236	yes
Csad	chr15:102176997-102189043	2.19758	1.3793	-0.671982	0.0138763	yes
Nxpe3	chr16:55839952-55895279	2.3232	1.45865	-0.671475	0.00338276	yes
Clpp	chr17:56990263-56996371	57.2606	35.9549	-0.671355	0.000195236	yes
Bcl2l12	chr7:44986899-44997579	4.56119	2.86478	-0.670984	0.0252408	yes

Fam58b	chr11:78750505-78751729	27.2538	17.1182	-0.67093	0.00202512	yes
Gm2a	chr11:55097984-55113028	16.8994	10.6175	-0.670534	0.000370691	yes
Dhodh	chr8:109593247-109608673	8.79108	5.52399	-0.670331	0.00160026	yes
Sh3bp5l	chr11:58330706-58347728	8.57535	5.38999	-0.669912	0.000698636	yes
Aimp1	chr3:132660497-132683879	83.5282	52.5057	-0.669791	0.000537301	yes
Leng9	chr7:4148182-4149872	1.51554	0.952753	-0.669662	0.0248334	yes
1500011K16Rik	chr2:127791376-127792488	33.1792	20.8635	-0.669299	0.00145427	yes
Itfg2	chr6:128409443-128424910	5.59971	3.52191	-0.668995	0.00244513	yes
Mrpl34	chr8:71464925-71465753	117.187	73.7099	-0.668888	0.000698636	yes
Rpa2	chr4:132768359-132778746	59.9049	37.7146	-0.66755	0.000195236	yes
Jade1	chr3:41555733-41616864	25.0512	15.7725	-0.667466	0.000195236	yes
Nme4	chr17:26091744-26095470	17.1849	10.8237	-0.666947	0.00160026	yes
Prkag2	chr5:24862734-25100642	12.0787	7.60816	-0.666845	0.00202512	yes
Rpl36al	chr12:69182733-69184067	268.728	169.269	-0.666831	0.000370691	yes
Oxa1l	chr14:54360840-54417702	20.286	12.7781	-0.666808	0.00202512	yes
Atxn7l2	chr3:108202227-108210527	2.29737	1.44729	-0.666635	0.00836289	yes
Churc1	chr12:76765572-76783178	26.3232	16.5841	-0.666532	0.00338276	yes
Hist2h4	chr3:96262933-96263317	976.014	614.961	-0.666406	0.000370691	yes
Zfp358	chr8:3493137-3497208	2.07611	1.30811	-0.666394	0.0173206	yes
Zfp61	chr7:24291045-24299549	4.1272	2.60154	-0.665797	0.00244513	yes
Slc25a1	chr16:17925210-17928219	27.4973	17.3357	-0.665546	0.00100977	yes
Cebpe	chr14:54710362-54712174	2.03845	1.28534	-0.665327	0.020214	yes
Harbi1	chr2:91710949-91721566	3.61765	2.28223	-0.664609	0.00870444	yes
Galm	chr17:80127470-80185032	4.38209	2.76552	-0.664066	0.00416625	yes
Rgs14	chr13:55369731-55384687	8.98398	5.6715	-0.663624	0.00160026	yes
Rtca	chr3:116488963-116508175	29.1182	18.3823	-0.663601	0.000855787	yes
Tmem237	chr1:59100592-59120096	27.8227	17.5786	-0.66244	0.000195236	yes
Rrm1	chr7:102441694-102469771	146.031	92.2674	-0.662378	0.000698636	yes
Telo2	chr17:25099568-25115967	6.72319	4.24878	-0.662097	0.00216236	yes
AI467606	chr7:127091435-127094049	10.3103	6.51755	-0.661683	0.00145427	yes
Cdk5r1	chr11:80477045-80481179	1.1119	0.703103	-0.661214	0.0164008	yes
Klhl36	chr8:119862304-119876989	3.59431	2.27286	-0.661208	0.00870444	yes
Nudt15	chr14:73519863-73548242	1.41892	0.89757	-0.660696	0.0204073	yes
Sec14l2	chr11:4097039-4118729	1.81345	1.14719	-0.660627	0.00755508	yes
2610301B20Rik	chr4:10874497-10899423	7.99452	5.05817	-0.660398	0.00351242	yes
Bub3	chr7:131560390-131571898	179.661	113.697	-0.66008	0.000855787	yes
Tagln2	chr1:172500245-172507375	169.377	107.198	-0.659965	0.000370691	yes
Nelfe	chr17:34850390-34856372	29.0982	18.4182	-0.659794	0.000855787	yes
Kctd7	chr5:130144887-130155808	1.37187	0.868722	-0.659179	0.0158044	yes
Ssbp2	chr13:91461096-91786148	0.898743	0.569174	-0.659039	0.0122489	yes
Pot1b	chr17:55652024-55712628	3.15906	2.00099	-0.658784	0.00285786	yes
Mrpl42	chr10:95480805-95501927	134.369	85.1159	-0.658696	0.000370691	yes
Mepce	chr5:137781905-137786701	13.3017	8.42938	-0.658117	0.000698636	yes
Ppie	chr4:123127124-123139941	34.2667	21.718	-0.657916	0.00116073	yes
Gm8615	chr5:149119564-149182036	1.39205	0.882348	-0.657789	0.0421425	yes
Pdrg1	chr2:153008889-153015383	38.4634	24.4079	-0.656139	0.00100977	yes
Snapc5	chr9:64179296-64182688	26.9573	17.1093	-0.655894	0.00174394	yes
Hmces	chr6:87913975-87936613	15.4237	9.78998	-0.655775	0.00298992	yes
Vps72	chr3:95111041-95123051	22.51	14.2884	-0.655726	0.000698636	yes
Tmem60	chr5:20882452-20886870	21.6358	13.7349	-0.655573	0.00492649	yes
Tm7sf2,Vps51	chr19:6062820-6077187	16.8496	10.7001	-0.655093	0.00174394	yes
Zfp101	chr17:33380178-33394637	8.82217	5.603	-0.654935	0.00271906	yes
Tmem219	chr7:126886218-126898278	23.0047	14.6104	-0.654934	0.003245	yes
Ptgr1	chr4:58965589-58987078	4.76155	3.02434	-0.654809	0.00454624	yes
Eef2kmt	chr16:5233620-5255956	15.5653	9.88785	-0.654606	0.0114837	yes
Eci2	chr13:34977747-34994144	18.3959	11.6906	-0.654036	0.00188078	yes
Wars2	chr3:99141089-99220203	4.73436	3.00995	-0.653432	0.00160026	yes
Zfp707	chr15:75969184-75975865	2.72557	1.73335	-0.652991	0.016003	yes
Tssc4	chr7:143069367-143071087	23.476	14.932	-0.652783	0.000855787	yes
Arap3	chr18:37972622-37998969	12.2194	7.77414	-0.652418	0.000698636	yes
Zfp825	chr13:74480056-74493950	6.4833	4.12494	-0.652353	0.00687627	yes
Pfkfb1	chrX:150589920-150643878	1.44722	0.920898	-0.652168	0.0254541	yes
Pcyox1l	chr18:61696836-61707635	14.5253	9.24441	-0.651912	0.00116073	yes
Arl6ip4	chr5:124116107-124118195	45.2858	28.8468	-0.650646	0.000698636	yes
Kcnab2	chr4:152390739-152477549	45.3399	28.8839	-0.650516	0.000195236	yes

Ftsj2	chr5:140327673-140331898	7.595	4.83891	-0.650368	0.00553759	yes
Sipa1	chr19:5651184-5663707	23.6279	15.0555	-0.650204	0.000370691	yes
Tbc1d22a	chr15:86214458-86498503	10.0217	6.38652	-0.650027	0.00188078	yes
Mad1l1	chr5:140008688-140321552	26.5082	16.8961	-0.649747	0.000855787	yes
Zfp354a	chr11:51059256-51072799	0.599361	0.382164	-0.649233	0.0385337	yes
Swsap1	chr9:21955283-21958270	4.96014	3.1632	-0.648998	0.00778355	yes
Smco4	chr9:15505494-15545259	15.4001	9.82518	-0.648382	0.00590647	yes
Xbp1	chr11:5520640-5525993	144.363	92.1387	-0.647823	0.00116073	yes
Ikbip	chr10:91083038-91102613	11.8701	7.57635	-0.647759	0.00578321	yes
Opa3	chr7:19228388-19246817	14.8528	9.48129	-0.647575	0.00116073	yes
Ppox	chr1:171276991-171281186	3.99491	2.55107	-0.647061	0.0100707	yes
Gnl3	chr14:30999825-31019131	121.248	77.4269	-0.647058	0.0036408	yes
Rad21	chr15:51962603-51991760	84.1711	53.7527	-0.646989	0.00116073	yes
Tbc1d2b	chr9:90202048-90270769	9.03726	5.77301	-0.646562	0.000537301	yes
Nat9	chr11:115182831-115187316	5.59591	3.57502	-0.646422	0.00859434	yes
Mplkip	chr13:17695412-17699105	11.1858	7.14745	-0.646161	0.00258324	yes
Cdca5	chr19:6085096-6091773	31.8613	20.3593	-0.646116	0.000698636	yes
Rsl24d1	chr9:73113468-73123333	40.2557	25.7259	-0.645974	0.000537301	yes
Timm17b	chrX:7899397-7907652	10.2252	6.53723	-0.645384	0.00271906	yes
Mief2	chr11:60728397-60732951	3.62567	2.3181	-0.645302	0.0101798	yes
Eif4b	chr15:102073772-102097173	190.86	122.123	-0.644182	0.00174394	yes
Sash3	chrX:48146526-48161563	1.29093	0.826167	-0.6439	0.0257635	yes
Cyb561d2	chr9:107539010-107541865	10.2151	6.54215	-0.642864	0.00492649	yes
Ctbp1	chr5:33247722-33275004	85.7587	54.9235	-0.642859	0.000855787	yes
Ahcy	chr2:155059311-155074497	3.37672	2.16332	-0.642377	0.00652015	yes
Thap7	chr16:17527981-17531052	25.6137	16.4096	-0.64237	0.000855787	yes
Ndufa13	chr8:69894181-69903518	74.5899	47.8007	-0.641948	0.000855787	yes
Camkk2	chr5:122731169-122779410	12.6802	8.12609	-0.641942	0.000855787	yes
Ring1	chr17:34020791-34024680	8.27303	5.30281	-0.641657	0.00216236	yes
Vars	chr17:35000906-35016329	47.9875	30.7591	-0.641642	0.000698636	yes
Ndufa9	chr6:126821862-126849144	94.8065	60.782	-0.641341	0.000537301	yes
Dpy19l1	chr9:24411778-24503140	45.3523	29.0779	-0.641255	0.00188078	yes
Slc9a6	chrX:56609834-56664230	20.6704	13.2635	-0.640097	0.000698636	yes
Nes	chr3:87971092-87980451	1.19223	0.765046	-0.640045	0.00377278	yes
Stoml2	chr4:43027689-43031384	46.6685	29.9476	-0.640006	0.000698636	yes
Rassf1	chr9:107551554-107562267	18.0723	11.6077	-0.638691	0.00216236	yes
Pcnp	chr16:56015507-56029717	37.1328	23.8585	-0.638191	0.000698636	yes
Nnt	chr13:119334316-119409257	17.382	11.1683	-0.638187	0.00116073	yes
Rpl41	chr10:128548109-128549168	1575.27	1012.21	-0.638094	0.00100977	yes
Fcf1	chr12:84970929-84983303	28.1081	18.0644	-0.637835	0.003245	yes
Pdcd5	chr7:35641984-35647482	15.8611	10.2001	-0.636916	0.00847587	yes
Zfp704	chr3:9427009-9610085	0.789658	0.508514	-0.634939	0.00723058	yes
Ccdc61	chr7:18890883-18910404	8.73556	5.62631	-0.634711	0.00454624	yes
Mrpl13	chr15:55534094-55557312	117.34	75.5802	-0.634617	0.000698636	yes
Bad	chr19:6938069-6951905	19.6342	12.648	-0.634457	0.0166035	yes
Rab29	chr1:131867276-131872889	10.844	6.98647	-0.634261	0.00441531	yes
Cog5	chr12:31654868-31950535	19.3642	12.4759	-0.634247	0.0386168	yes
Hexa	chr9:59539666-59565105	126.293	81.3806	-0.634021	0.000698636	yes
Pecr	chr1:72259172-72284314	11.1056	7.15665	-0.633929	0.00675438	yes
Mthfd1	chr12:76255231-76319820	85.2715	54.9582	-0.63373	0.000537301	yes
Acad10	chr5:121621028-121660510	1.05652	0.68128	-0.632994	0.0185753	yes
Dnajc12	chr10:63382442-63408840	4.87243	3.14369	-0.632185	0.00926854	yes
Glipr1	chr10:111972694-111997264	144.871	93.5071	-0.631622	0.0248334	yes
Mitf	chr6:97807057-98021358	29.3877	18.9688	-0.631586	0.000370691	yes
P3h3	chr6:124841094-124857687	3.5993	2.32373	-0.631275	0.00566351	yes
Lix1l	chr3:96601132-96629819	11.5195	7.43768	-0.631153	0.0187817	yes
Npm3-ps1	chr6:85076140-85077126	55.3516	35.7645	-0.630097	0.00130921	yes
Tbca	chr13:94788942-94842899	69.9153	45.1802	-0.629919	0.00258324	yes
Sec14l1	chr11:117115171-117159268	10.3961	6.7221	-0.629058	0.00504708	yes
Tmco4	chr4:138972282-139059171	5.9847	3.8711	-0.628537	0.0036408	yes
Ccdc9	chr7:16274041-16286795	10.6017	6.85782	-0.628478	0.00145427	yes
Echs1	chr7:140105722-140116423	20.1754	13.0528	-0.62824	0.00377278	yes
Mrpl38	chr11:116131816-116138868	41.3093	26.7287	-0.62808	0.000698636	yes
Rplp1	chr9:61913282-61914510	1322.59	855.858	-0.62792	0.00298992	yes
Rfx2	chr17:56775896-56831008	0.568509	0.367957	-0.627647	0.0456796	yes

Zfp74	chr7:29932790-29951893	2.77372	1.79559	-0.627365	0.003245	yes
Crlf2	chr5:109554708-109558993	2.67812	1.73392	-0.627185	0.0469626	yes
Ccdc69	chr11:55049737-55078131	1.43698	0.930952	-0.626256	0.0321137	yes
Rad51ap1	chr6:126923418-126939555	29.7691	19.291	-0.62589	0.00116073	yes
Zfp456	chr13:67363583-67375763	1.49413	0.968259	-0.625838	0.0162041	yes
Larp7	chr3:127536713-127553349	41.5439	26.9258	-0.625647	0.000698636	yes
Hsdl1	chr8:119561977-119575200	14.5081	9.40802	-0.624893	0.00174394	yes
Ssh3	chr19:4261668-4269172	6.10981	3.96332	-0.624419	0.00285786	yes
Zfp97	chr17:17121382-17146878	1.39981	0.908046	-0.624394	0.0214996	yes
Gmds	chr13:31819585-32338544	37.6323	24.4124	-0.624358	0.00145427	yes
Prkcb	chr7:122289124-122634401	1.66693	1.08158	-0.624053	0.00351242	yes
Sec13	chr6:113728051-113740681	108.119	70.1949	-0.623189	0.00100977	yes
Supt3	chr17:44495986-45119284	16.0169	10.4018	-0.62276	0.00663679	yes
Mef2c	chr13:83504033-83667079	54.8406	35.6151	-0.622755	0.000698636	yes
Zfp212	chr6:47920567-47932637	3.6679	2.38262	-0.622409	0.00825328	yes
Axl	chr7:25756499-25788733	17.2608	11.2125	-0.622391	0.000370691	yes
Aldh2	chr5:121566026-121593824	40.3013	26.187	-0.621976	0.0315437	yes
Tomm5	chr4:45105209-45108113	127.9	83.1115	-0.621901	0.00130921	yes
Zbtb45	chr7:13005665-13009800	5.36248	3.48502	-0.621733	0.00904622	yes
Txn1	chr4:57943372-57956411	387.742	252.046	-0.621414	0.00188078	yes
Hnrnpa1	chr15:103240396-103246698	43.8962	28.5409	-0.621065	0.00258324	yes
Adat3,Scamp4	chr10:80602879-80615783	19.2757	12.5357	-0.62074	0.003245	yes
Dtnbp1	chr13:44922079-45002096	23.7118	15.421	-0.620711	0.00188078	yes
Diap3	chr14:86656322-87141114	17.3249	11.2687	-0.620534	0.00145427	yes
Nup85	chr11:115564443-115583924	87.076	56.6393	-0.620472	0.00100977	yes
4930558J18Rik	chr1:57359221-57377544	1.78444	1.16088	-0.620246	0.0325793	yes
Osbpl1a	chr18:12755311-12941841	7.32704	4.76684	-0.620198	0.00311626	yes
Inf2	chr12:112588783-112615557	4.00937	2.60856	-0.620122	0.00311626	yes
Vdac3	chr8:22577074-22593813	27.3259	17.7803	-0.61999	0.00216236	yes
Lmf1	chr17:25579173-25662826	9.58503	6.24034	-0.619159	0.00492649	yes
Prmt7	chr8:106211053-106251694	40.9829	26.6876	-0.618853	0.00100977	yes
Pus7	chr5:23740164-23783711	22.9869	14.9707	-0.61867	0.000855787	yes
Exosc7	chr9:123113230-123136129	53.7972	35.0408	-0.618495	0.000370691	yes
Ndufv1	chr19:4007498-4012755	79.0051	51.4794	-0.617951	0.000855787	yes
Fkbp14	chr6:54577604-54593128	2.07995	1.35531	-0.617933	0.0194933	yes
Gpsm2	chr3:108653912-108722299	27.2381	17.7512	-0.617714	0.0428589	yes
Cnpy2	chr10:128322458-128327187	46.3943	30.2443	-0.617285	0.00230418	yes
Rufy1	chr11:50389302-50431111	27.3696	17.845	-0.61705	0.00188078	yes
Ddx56	chr11:6257544-6267729	25.3291	16.5213	-0.616465	0.00188078	yes
Med11	chr11:70451930-70453726	35.9893	23.4778	-0.61627	0.00244513	yes
Ankrd16	chr2:11777752-11790323	2.95336	1.92697	-0.616022	0.0172208	yes
Pole	chr5:110286318-110337453	17.8364	11.642	-0.615485	0.00145427	yes
Mtss1	chr15:58941233-59082026	10.8518	7.0855	-0.61499	0.00160026	yes
Mcm6	chr1:128331590-128359656	288.01	188.126	-0.614422	0.00441531	yes
Fkbp4	chr6:128430106-128438631	275.973	180.317	-0.61399	0.00258324	yes
Cstf1	chr2:172371002-172381086	49.7887	32.5448	-0.613391	0.00145427	yes
Srsf3	chr17:29032659-29043372	128.032	83.7424	-0.612472	0.00174394	yes
Pknox1	chr17:31564772-31607693	13.0829	8.55731	-0.612456	0.00230418	yes
Atad3a	chr4:155740639-155761098	37.233	24.3548	-0.612374	0.00160026	yes
Prdx6	chr1:161240111-161251210	152.055	99.4923	-0.611937	0.00188078	yes
Tmsb10	chr6:72957346-72958748	8.85992	5.7975	-0.611863	0.00825328	yes
Guk1	chr11:59183854-59191952	45.4329	29.7455	-0.611064	0.00145427	yes
Gpd1l	chr9:114899338-114933987	25.1208	16.4515	-0.610665	0.000698636	yes
N6amt1	chr16:87354184-87368649	11.1333	7.2942	-0.610058	0.00271906	yes
Chkb,Cpt1b	chr15:89416404-89429927	9.80567	6.42708	-0.609452	0.00602767	yes
Urm1	chr2:29827388-29844996	20.0126	13.1179	-0.60937	0.00145427	yes
Abcb4	chr5:8893720-8959226	27.7227	18.1781	-0.608867	0.000537301	yes
Cd302	chr2:60251992-60284484	16.8798	11.0742	-0.6081	0.00467182	yes
Thrap3	chr4:126164082-126202710	25.5279	16.7558	-0.60741	0.00188078	yes
A430005L14Rik	chr4:153957236-153961924	70.0573	46.0132	-0.606486	0.00116073	yes
Endod1	chr9:14353989-14381242	21.1402	13.8856	-0.606404	0.00160026	yes
Cbs	chr17:31612622-31637205	1.66795	1.09559	-0.606368	0.0229541	yes
Serf2	chr2:121449197-121456764	10.102	6.6428	-0.604772	0.00230418	yes
Sema5a	chr15:32244812-32696341	5.906	3.88394	-0.604659	0.00285786	yes
Pip4k2a	chr2:18842255-18998121	56.3051	37.0323	-0.604481	0.00202512	yes

Vim	chr2:13574310-13582826	537.867	353.822	-0.604225	0.00479652	yes
Snapc2	chr8:4253101-4256220	9.35768	6.15619	-0.604114	0.0103944	yes
Kbtbd6	chr14:79451834-79454816	1.55329	1.02189	-0.604078	0.0302743	yes
Fbxl19	chr7:127746774-127768928	3.6397	2.39524	-0.603648	0.00627268	yes
1110012L19Rik	chrX:70385912-70389416	7.56726	4.98182	-0.603098	0.0114837	yes
Fam198b	chr3:79885929-79946278	1.75077	1.15266	-0.603028	0.0127805	yes
Fance	chr17:28313529-28326574	12.5258	8.24829	-0.602737	0.00541779	yes
Zfp938	chr10:82224855-82241275	1.72807	1.13812	-0.602503	0.0480092	yes
Alg6	chr4:99715629-99763460	20.6888	13.6285	-0.602221	0.00311626	yes
Chek2	chr5:110840016-110874133	21.209	13.978	-0.601523	0.00338276	yes
Pip5k1c	chr10:81292962-81319974	26.4285	17.4276	-0.600717	0.00258324	yes
Suv420h2	chr7:4740126-4747514	7.27241	4.79677	-0.600369	0.00639665	yes
Zfat	chr15:68083737-68258856	3.66898	2.42182	-0.599285	0.00590647	yes
Ninl	chr2:150934518-151009398	2.35788	1.55659	-0.5991	0.00789883	yes
Srsf9	chr5:115327176-115333080	122.833	81.1013	-0.598902	0.00160026	yes
Acad9	chr3:36065999-36092857	13.6218	8.99466	-0.598774	0.00271906	yes
Fancg	chr4:43002336-43010301	3.21305	2.12165	-0.598755	0.0129867	yes
Senp3	chr11:69673109-69682084	32.6538	21.5626	-0.598722	0.00298992	yes
Plrg1	chr3:83055537-83072291	60.6089	40.0296	-0.598462	0.00116073	yes
Adk	chr14:21052573-21448569	89.3162	58.9971	-0.598278	0.00145427	yes
Mad2l1bp	chr17:46147384-46153551	10.2239	6.75719	-0.597448	0.0107144	yes
Supv3l1	chr10:62429377-62451353	24.9891	16.5196	-0.597115	0.00116073	yes
Mthfsd	chr8:121097556-121108379	11.2885	7.46292	-0.597047	0.00479652	yes
Exosc4	chr15:76327396-76330670	13.7145	9.07345	-0.595976	0.00467182	yes
Ctss	chr3:95526785-95556405	234.362	155.19	-0.594707	0.00174394	yes
Zfp128	chr7:12881177-12893422	1.20055	0.795027	-0.594617	0.0219511	yes
2410002F23Rik	chr7:44246721-44252319	20.7936	13.7712	-0.594488	0.00258324	yes
Dcaf4	chr12:83520465-83541992	10.9911	7.27982	-0.59436	0.0036408	yes
Ndufb9	chr15:58933809-58939489	219.097	145.165	-0.593875	0.00130921	yes
Ccdc142,Mrpl53	chr6:83101515-83109932	50.9638	33.7709	-0.593693	0.0259557	yes
Adh5	chr3:138437199-138455499	122.695	81.3076	-0.593608	0.00116073	yes
Cdk5	chr5:24418241-24423530	13.4228	8.89509	-0.593601	0.00553759	yes
Mar-02	chr1:184813067-184845847	30.2149	20.0377	-0.592546	0.00311626	yes
Prkra	chr2:76629936-76647994	13.1405	8.71577	-0.592319	0.00467182	yes
Espnl	chr1:91322074-91348303	1.59506	1.05826	-0.591914	0.0108186	yes
Tpk1	chr6:43345006-43666175	4.98462	3.30747	-0.591755	0.011374	yes
Thop1	chr10:81070082-81082360	36.3952	24.1564	-0.591344	0.00116073	yes
Polr3k	chr2:181864359-181870826	22.0561	14.6397	-0.591293	0.003245	yes
Bcat2	chr7:45567794-45589710	17.9464	11.9123	-0.591244	0.00428914	yes
Col4a5	chrX:141475418-141689235	9.66522	6.41709	-0.590883	0.003245	yes
Tdp2	chr13:24831658-24842153	45.9565	30.5204	-0.590495	0.00188078	yes
Vat1	chr11:101458747-101466199	21.6084	14.3519	-0.590344	0.00258324	yes
Apopt1	chr12:111713268-111755055	39.5993	26.3039	-0.590195	0.00188078	yes
Trmt2b	chrX:134222954-134276984	17.9424	11.9187	-0.590145	0.00529192	yes
Ndufs2	chr1:171234859-171247112	155.802	103.523	-0.589761	0.00216236	yes
Plekhj1	chr10:80796098-80798626	35.7755	23.7736	-0.589611	0.00428914	yes
Nphp1	chr2:127740731-127788891	5.82557	3.87171	-0.58943	0.00699437	yes
Txnl4a	chr18:80206797-80225851	116.489	77.4242	-0.589335	0.0118007	yes
Pygb	chr2:150786795-150831748	18.3432	12.1924	-0.589271	0.00202512	yes
Egln2	chr7:27158657-27166802	22.5351	14.9789	-0.589243	0.00216236	yes
Ctcf	chr8:105636537-105682922	41.6803	27.7159	-0.58865	0.00145427	yes
Hirip3	chr7:126861971-126865122	29.179	19.4047	-0.588521	0.003245	yes
Epb4.1l4aos	chr18:33794891-33795989	27.6378	18.3946	-0.587357	0.0173206	yes
Zwilch	chr9:64117146-64172931	30.8628	20.5439	-0.587159	0.00145427	yes
Slc39a11	chr11:113244854-113565815	19.0237	12.6635	-0.587119	0.00416625	yes
Cyba	chr8:122424770-122432940	237.794	158.319	-0.58688	0.00145427	yes
Psph	chr5:129765557-129787253	35.1892	23.4289	-0.586842	0.00244513	yes
Ndufb3	chr1:58586598-58595948	282.059	187.805	-0.586761	0.00271906	yes
Mars2	chr1:55237176-55240058	16.5181	10.9985	-0.586746	0.00390381	yes
Edf1	chr2:25557899-25562082	81.4776	54.2649	-0.586384	0.00216236	yes
Tpx2	chr2:152847963-152895321	88.9936	59.2805	-0.586144	0.0036408	yes
Pex26	chr6:121183666-121198837	2.54099	1.69442	-0.584595	0.0220515	yes
Epb4.1l4a	chr18:33796326-34007206	0.7521	0.501562	-0.584495	0.0396355	yes
Tbc1d31	chr15:57912198-57970068	21.5507	14.3744	-0.584231	0.00285786	yes
Dnmt1	chr9:20907205-20959888	78.0293	52.0484	-0.584163	0.00467182	yes

Chchd4	chr6:91464275-91473423	64.0791	42.7476	-0.58401	0.00285786	yes
Scrib	chr15:76047185-76069730	13.0358	8.69843	-0.583653	0.00216236	yes
Emc6	chr11:73175502-73177042	71.1234	47.4662	-0.583425	0.003245	yes
Ccr10	chr11:101172997-101175443	3.29721	2.20086	-0.583179	0.0357258	yes
Mmab	chr5:114431033-114444027	5.02073	3.35154	-0.583072	0.00778355	yes
Laptm5	chr4:130913333-130936148	139.044	92.8257	-0.582949	0.00311626	yes
Ruvbl1	chr6:88465422-88497566	13.042	8.70864	-0.58265	0.00467182	yes
Akr7a5	chr4:139310743-139318786	10.2073	6.81748	-0.582296	0.00710851	yes
Lpcat4	chr2:112239840-112247111	14.9421	9.98519	-0.581523	0.00590647	yes
Srsf7	chr17:80200079-80207305	151.234	101.11	-0.580861	0.003245	yes
Ankrd28	chr14:31700014-31830415	39.896	26.6742	-0.580799	0.00285786	yes
Adrb2	chr18:62177712-62179981	12.6847	8.48154	-0.580694	0.00652015	yes
Aurkaip1	chr4:155831268-155833098	90.9916	60.8426	-0.580653	0.00174394	yes
Creg1	chr1:165763779-165775304	151.698	101.441	-0.580561	0.00351242	yes
Rab1b	chr19:5099206-5106996	81.2554	54.3404	-0.580438	0.00244513	yes
Toe1	chr4:116720954-116807559	19.8488	13.2807	-0.579729	0.00755508	yes
Mrps18a	chr17:46111003-46128908	83.613	55.9546	-0.57947	0.00258324	yes
Prdx2	chr8:84969647-84974313	234.877	157.182	-0.579468	0.00244513	yes
Ergic1	chr17:26561511-26656933	39.4732	26.4257	-0.578935	0.00216236	yes
Mapk3	chr7:126759625-126765816	21.1073	14.1309	-0.578889	0.00244513	yes
Nsmce2	chr15:59374197-59601684	26.2024	17.5436	-0.578757	0.00390381	yes
Alkbh6	chr7:30308752-30314303	13.0258	8.72212	-0.578625	0.014404	yes
Card9	chr2:26352311-26359547	27.8611	18.658	-0.578456	0.00298992	yes
Eif3f	chr7:108934414-108941942	90.6699	60.7311	-0.578187	0.003245	yes
Sft2d3	chr18:31909093-31911903	4.84977	3.24844	-0.578171	0.0146178	yes
Fry	chr5:150259929-150497753	0.967431	0.648025	-0.57811	0.00938869	yes
Dap3	chr3:88920802-88950282	17.7883	11.9163	-0.577991	0.00298992	yes
Siah1b	chrX:164070702-164076493	14.191	9.51406	-0.576838	0.00755508	yes
Zfp296	chr7:19577286-19580656	5.20225	3.48937	-0.57617	0.0175233	yes
Parvg	chr15:84324719-84342978	52.7404	35.3938	-0.575411	0.00130921	yes
Ndst2	chr14:20723729-20734562	7.20204	4.83384	-0.575237	0.00639665	yes
Rps9	chr7:3704040-3706897	1166.23	783.18	-0.57444	0.00454624	yes
H1f0	chr15:79028211-79030500	93.4313	62.7541	-0.574195	0.00230418	yes
Il18rap	chr1:40515361-40551705	10.5193	7.06928	-0.573407	0.00216236	yes
Runx3	chr4:135120644-135177990	1.1699	0.786469	-0.57292	0.0159005	yes
Dguok	chr6:83480213-83506969	13.5827	9.13427	-0.572414	0.011588	yes
Trip6	chr5:137309898-137314241	29.1648	19.6134	-0.572389	0.00271906	yes
Zfp623	chr15:75940951-75949400	7.40276	4.97838	-0.572388	0.00627268	yes
2310047M10Rik	chr11:69059774-69061576	7.62127	5.12775	-0.571706	0.0108186	yes
Mvd	chr8:122433595-122443422	17.5067	11.7789	-0.571698	0.00441531	yes
Ldlrap1	chr4:134745411-134768004	16.5699	11.1489	-0.571667	0.0036408	yes
Hdac10	chr15:89123302-89128700	12.0002	8.07521	-0.571493	0.00504708	yes
Msrb1	chr17:24736641-24742778	34.0002	22.8832	-0.571252	0.00602767	yes
Man2c1	chr9:57129981-57142210	11.3882	7.66535	-0.571111	0.00553759	yes
Ttc39aos1	chr4:109402278-109406257	4.4978	3.02908	-0.570341	0.0218594	yes
Mrpl54	chr10:81264721-81266926	152.069	102.44	-0.569944	0.00298992	yes
2610203C22Rik	chr1:9548045-9631092	27.4757	18.5134	-0.569582	0.0311881	yes
Ilvbl	chr10:78574499-78584502	6.82613	4.59987	-0.569474	0.0125567	yes
Ugt1a1,Ugt1a10,Ugt1a2,	chr1:88055410-88220002	43.7504	29.4996	-0.568601	0.003245	yes
Psmb9	chr17:34182098-34187330	18.764	12.6534	-0.568448	0.0114837	yes
Tmem177	chr1:119907898-119913168	4.70023	3.17026	-0.568133	0.00733312	yes
Pspc1	chr14:56722448-56778316	41.8817	28.2516	-0.567987	0.00188078	yes
Topbp1	chr9:103305326-103350427	92.2377	62.2323	-0.567694	0.0036408	yes
Slco4a1	chr2:180460977-180474853	34.0583	22.9811	-0.567559	0.00258324	yes
Zfp955a	chr17:33239506-33255145	8.77709	5.92336	-0.567327	0.00602767	yes
Timm22	chr11:76406924-76416313	15.0044	10.1353	-0.565995	0.00529192	yes
Socs5	chr17:87107678-87137588	11.2407	7.59406	-0.565784	0.00675438	yes
Tecr	chr8:83571697-83594491	62.9464	42.5303	-0.565633	0.00285786	yes
Bap1	chr14:31251488-31259929	21.7481	14.6976	-0.565302	0.00377278	yes
Rwdd1	chr10:33996554-34019616	23.1391	15.6379	-0.565282	0.00553759	yes
Pfdn6	chr17:33938908-33940343	61.5508	41.6	-0.565195	0.0116945	yes
Twf2	chr9:106203107-106215387	42.6394	28.8199	-0.565121	0.00529192	yes
Dnmt3b	chr2:153649448-153687730	2.74199	1.85364	-0.564867	0.0124526	yes
Taf8	chr17:47488049-47502287	11.8739	8.02702	-0.564852	0.00529192	yes
Dpy30	chr17:74299473-74323944	178.086	120.43	-0.564383	0.00188078	yes

Ndufa5	chr6:24518665-24527687	149.242	100.93	-0.564296	0.00403597	yes
Bhlhe40	chr6:108577035-108666925	11.3014	7.64333	-0.564227	0.00578321	yes
Zfp189	chr4:49521175-49531558	3.98261	2.69427	-0.563822	0.0102954	yes
0610010K14Rik	chr11:70235203-70237914	30.6825	20.7592	-0.563666	0.0103944	yes
Rgs1	chr1:144244668-144249104	20.4749	13.8613	-0.562793	0.00733312	yes
Zfp566	chr7:30077336-30090510	3.67398	2.48727	-0.562779	0.0193933	yes
Jrk	chr15:74702411-74710374	3.47295	2.35176	-0.56242	0.00926854	yes
Bod1	chr11:31665149-31671862	20.9045	14.1582	-0.562169	0.00789883	yes
B230219D22Rik	chr13:55693123-55703500	33.7567	22.8633	-0.562136	0.00285786	yes
Mrps7	chr11:115604150-115607624	50.7594	34.3873	-0.561799	0.00428914	yes
Hemk1	chr9:107327081-107338350	2.61928	1.77564	-0.560828	0.0303714	yes
Neil1	chr9:57143255-57147034	3.2474	2.20149	-0.560805	0.0442967	yes
Ctdp1	chr18:80407958-80469667	22.57	15.3011	-0.560769	0.00244513	yes
Bbs2	chr8:94067953-94098811	2.8163	1.90959	-0.560534	0.0200117	yes
Zfp317	chr9:19622090-19649731	12.3632	8.38356	-0.560413	0.00492649	yes
Ctnnbl1	chr2:157737400-157891903	43.0383	29.1859	-0.560351	0.00298992	yes
Psmb10	chr8:105935727-105938392	27.8385	18.8826	-0.560022	0.00416625	yes
Cenpv	chr11:62524943-62539261	69.2809	46.9977	-0.559867	0.0036408	yes
Uqcrh	chr4:116066964-116075070	422.934	286.947	-0.559646	0.00403597	yes
Noa1	chr5:77294168-77310086	23.9069	16.2205	-0.559609	0.00351242	yes
Atg7	chr6:114643096-114860614	9.64946	6.54748	-0.559508	0.00517183	yes
Foxo4	chrX:101254527-101260873	2.55713	1.73539	-0.559262	0.0201232	yes
Rpa1	chr11:75300258-75348383	90.0787	61.1368	-0.559144	0.00403597	yes
1110059E24Rik	chr19:21597312-21652791	28.8366	19.5736	-0.558992	0.00590647	yes
Zfp688	chr7:127418965-127422034	3.59807	2.44246	-0.558886	0.0479166	yes
Snx25	chr8:46033260-46124146	20.063	13.6236	-0.558424	0.00517183	yes
Psap	chr10:60277627-60302600	220.662	149.866	-0.558166	0.00733312	yes
Tars2	chr3:95739973-95754977	37.9025	25.7586	-0.557238	0.00441531	yes
D2hgdh	chr1:93825239-93852174	1.96294	1.33405	-0.557206	0.0343814	yes
Dtx4	chr19:12466335-12501996	11.9703	8.13704	-0.55688	0.00271906	yes
Tep1	chr14:50824060-50870554	7.31076	4.97041	-0.556655	0.00479652	yes
Bcl2	chr1:106538177-106714290	4.85741	3.30261	-0.556582	0.0299428	yes
Tsen2	chr6:115544703-115578336	8.34186	5.672	-0.55651	0.00973453	yes
Tmem218	chr9:37208222-37223228	10.87	7.39349	-0.556027	0.0281835	yes
Cpsf4	chr5:145167212-145182041	24.0471	16.3562	-0.55602	0.00416625	yes
Dscc1	chr15:55076100-55090478	15.3051	10.4135	-0.555565	0.0114837	yes
Trappc3	chr4:126262404-126275883	34.2259	23.2897	-0.555393	0.00710851	yes
Kdm2b	chr5:122870674-122989099	11.2935	7.68683	-0.555036	0.00847587	yes
Kat8	chr7:127912516-127930113	7.13135	4.85545	-0.554572	0.0158044	yes
Haus8	chr8:71251123-71272590	31.5872	21.5072	-0.554516	0.00390381	yes
Tprgl	chr4:154157484-154160684	39.0574	26.596	-0.554385	0.00578321	yes
Dnpep	chr1:75308564-75317637	26.9349	18.3416	-0.554355	0.00627268	yes
4930579G24Rik	chr3:79629078-79632819	15.0304	10.2404	-0.553613	0.00836289	yes
Polr2j	chr5:136116690-136122947	134.288	91.4927	-0.553597	0.00285786	yes
Dnal4	chr15:79761448-79774467	8.169	5.56606	-0.553501	0.0129867	yes
Tyw1	chr5:130255618-130341567	13.0166	8.86974	-0.553386	0.00590647	yes
2410004B18Rik	chr3:145938031-145944275	23.4356	15.9734	-0.553032	0.00766958	yes
Zfp874b	chr13:67471512-67484253	2.04434	1.39346	-0.55297	0.0255556	yes
Zmat5	chr11:4704677-4737666	15.6566	10.6729	-0.552811	0.0173206	yes
Lrrc42	chr4:107233513-107253533	12.9282	8.81688	-0.552176	0.0104999	yes
Ttc27	chr17:74717749-74863570	42.7044	29.1309	-0.551837	0.0036408	yes
G3bp1	chr11:55469751-55500887	118.543	80.8796	-0.551562	0.00311626	yes
Arsa	chr15:89472475-89484850	2.99223	2.04419	-0.549692	0.0191808	yes
Lman2	chr13:55343832-55362783	68.8134	47.0324	-0.549034	0.00403597	yes
Jagn1	chr6:113442516-113448229	52.3419	35.7757	-0.548987	0.00766958	yes
Mmd	chr11:90249475-90278573	32.9674	22.5339	-0.548942	0.00517183	yes
Eif3i	chr4:129591973-129600648	201.204	137.532	-0.548896	0.00403597	yes
Sgsm2	chr11:74849263-74897080	2.00522	1.37092	-0.548621	0.0166035	yes
Tssc1	chr12:28751827-28867491	22.8789	15.6494	-0.547909	0.00541779	yes
Rmnd1	chr10:4403168-4432352	8.67268	5.93341	-0.547615	0.0133195	yes
Mtrf1	chr14:79397771-79423650	9.01895	6.17445	-0.546648	0.00984695	yes
Gm20554	chr13:72623465-72628564	1.28885	0.882382	-0.546607	0.0290891	yes
Nucks1	chr1:131910457-131936321	260.34	178.253	-0.546466	0.016003	yes
Fiz1	chr7:5007055-5014728	9.70438	6.64969	-0.54535	0.00755508	yes
Gpc1	chr1:92831685-92860196	1.38205	0.947328	-0.544873	0.0425167	yes

Pcgf6	chr19:47033618-47050845	21.6314	14.8282	-0.544778	0.00836289	yes
Ogfod2	chr5:124112337-124115476	5.88957	4.03805	-0.544503	0.0320425	yes
Hnrnpl	chr7:28810889-28822266	151.246	103.724	-0.544145	0.00529192	yes
Zxdb	chrX:94724568-94730191	1.26201	0.865543	-0.544047	0.0433818	yes
Arhgef10	chr8:14911662-15001085	4.55716	3.12696	-0.543374	0.00836289	yes
Mbip	chr12:56328306-56345894	13.2927	9.12118	-0.543337	0.0125567	yes
Iqgap2	chr13:95627176-95891922	2.55748	1.75527	-0.543028	0.00859434	yes
Rpl29	chr9:106429538-106431567	46.0048	31.5784	-0.542845	0.00602767	yes
Elovl6	chr3:129532385-129638493	14.9234	10.2438	-0.542828	0.00441531	yes
Gsdmd	chr15:75862338-75867404	39.1331	26.8673	-0.542541	0.00663679	yes
Rtcb	chr10:85938636-85957793	99.9199	68.6143	-0.542263	0.00351242	yes
Nxf7	chrX:135579786-135593855	11.7188	8.04835	-0.542065	0.0107144	yes
Galnt2	chr8:124231393-124345723	13.9426	9.5763	-0.541954	0.00416625	yes
Prex1	chr2:166566344-166722152	19.524	13.417	-0.541191	0.00285786	yes
Gstcd	chr3:132982550-133091740	16.2184	11.1454	-0.54118	0.00926854	yes
2610001J05Rik	chr6:13869073-13871483	42.2472	29.0342	-0.541104	0.00652015	yes
Sfi1	chr11:3124020-3193463	5.05292	3.47275	-0.54104	0.0166035	yes
Tbl3	chr17:24700652-24707653	38.8752	26.7187	-0.541002	0.00504708	yes
Clk4	chr11:51263113-51281770	27.4863	18.8917	-0.54096	0.00836289	yes
Cdan1	chr2:120716153-120731517	3.23846	2.22584	-0.54096	0.0124526	yes
Npm1	chr11:33152497-33163206	464.133	319.096	-0.540549	0.0103944	yes
Cers4	chr8:4493404-4526079	1.73845	1.19523	-0.540509	0.0273047	yes
Dnajc9	chr14:20384637-20388910	68.5621	47.1534	-0.540049	0.00351242	yes
Tnfaip8l2	chr3:95134087-95142360	21.5294	14.8099	-0.53975	0.0116945	yes
Mgat4b	chr11:50225334-50235103	25.5581	17.5905	-0.53899	0.00710851	yes
Ptcd2	chr13:99319648-99344678	50.4909	34.7512	-0.538963	0.00454624	yes
Trap1	chr16:4037144-4077810	85.9923	59.1867	-0.538934	0.003245	yes
Mfge8	chr7:79133767-79149060	35.9345	24.7352	-0.538801	0.00517183	yes
Pcbp1	chr6:86524496-86526165	76.3111	52.5333	-0.53866	0.00590647	yes
Wdsub1	chr2:59852364-59882606	4.63392	3.19021	-0.538583	0.0227508	yes
Sf3a3	chr4:124714860-124732422	76.9804	53.0077	-0.538289	0.00377278	yes
Cisd3	chr11:97685951-97688625	9.80728	6.75341	-0.538236	0.033674	yes
Zfp956	chr6:47943174-47965299	3.28967	2.26565	-0.538014	0.0221562	yes
Stk19	chr17:34823992-34836903	10.6089	7.3083	-0.53767	0.0301829	yes
Zfp748	chr13:67538640-67553152	8.47196	5.83787	-0.537254	0.00926854	yes
Mrpl19	chr6:81923668-81965949	16.536	11.3973	-0.53692	0.0165092	yes
Bax	chr7:45461694-45466898	165.997	114.433	-0.536652	0.00377278	yes
Ddx49	chr8:70282998-70302452	32.287	22.2598	-0.53651	0.0080155	yes
Ccdc117	chr11:5528887-5542217	17.3971	11.9987	-0.535972	0.00441531	yes
Slc39a10	chr1:46807543-46853509	43.4251	29.9574	-0.535616	0.00428914	yes
Hyal1,Nat6	chr9:107576951-107587359	4.63938	3.20061	-0.535584	0.0416643	yes
Add3	chr19:53140442-53247326	31.9935	22.0734	-0.535471	0.00553759	yes
Cysltr1	chrX:106576508-106710557	16.9185	11.6735	-0.535362	0.00789883	yes
Rtfdc1	chr2:172440577-172469899	30.8051	21.257	-0.535227	0.00479652	yes
Thoc3	chr13:54458836-54468840	36.9035	25.4698	-0.534972	0.00479652	yes
Bbs12	chr3:37312553-37321451	7.24016	4.99743	-0.534834	0.0182627	yes
Wdr61	chr9:54717152-54734549	88.4376	61.0461	-0.534761	0.00663679	yes
B3galnt2	chr13:13954673-14039638	19.3021	13.3273	-0.534379	0.0452271	yes
Taf6	chr5:138172020-138187186	25.7433	17.7759	-0.534273	0.0103944	yes
Mpst	chr15:78406711-78414015	13.4292	9.27308	-0.53425	0.0140893	yes
D030056L22Rik	chr19:18713235-18718428	33.3798	23.0521	-0.534081	0.0100707	yes
Nrp1	chr8:128359072-128505475	6.743	4.65736	-0.533878	0.00938869	yes
Fbxo21	chr5:117976769-118010191	10.7938	7.45618	-0.5337	0.00699437	yes
Ifnar2	chr16:91372782-91405587	65.7948	45.4498	-0.533698	0.014404	yes
Zfp940	chr7:29843935-29853648	1.9145	1.32308	-0.533073	0.0374232	yes
Mrps18b	chr17:35910384-35916369	23.2453	16.0675	-0.532788	0.0100707	yes
Bnip1	chr17:26781078-26792521	22.0814	15.2637	-0.532723	0.00881201	yes
Sh3bgrl	chrX:109095406-109162467	191.486	132.367	-0.532697	0.00639665	yes
Fzd7	chr1:59482146-59486955	6.4477	4.45755	-0.532534	0.0102954	yes
Sord	chr2:122234838-122265337	11.1634	7.71822	-0.532441	0.0100707	yes
Cdk19	chr10:40349307-40483818	11.7784	8.1444	-0.532264	0.00744706	yes
Med26	chr8:72494558-72548310	4.74593	3.28223	-0.532013	0.0148245	yes
9130019O22Rik	chr7:127382259-127387166	2.3277	1.60984	-0.531986	0.0191808	yes
Nfyc	chr4:120757434-120831579	6.9148	4.7828	-0.531832	0.014404	yes
Ywhaq	chr12:21390328-21417436	23.6873	16.3845	-0.531779	0.00789883	yes

Gna13	chr11:109354777-109401369	29.5549	20.4466	-0.53154	0.00390381	yes
Mus81	chr19:5482839-5488336	8.21686	5.68576	-0.531234	0.0141985	yes
Nop9	chr14:55745692-55758424	20.6055	14.262	-0.530858	0.00881201	yes
Tubb4b	chr2:25218744-25224702	259.596	179.71	-0.5306	0.00627268	yes
2510009E07Rik	chr16:21649044-21694665	56.996	39.4765	-0.529869	0.00566351	yes
Idh3g	chrX:73778962-73786897	179.479	124.325	-0.529694	0.00553759	yes
Deaf1	chr7:141297175-141338783	7.98207	5.53124	-0.52916	0.0415762	yes
Orai3	chr7:127769814-127775150	21.0517	14.5889	-0.529065	0.0104999	yes
Slx1b	chr7:126688926-126695783	2.04116	1.41487	-0.528726	0.0300914	yes
Mettl1	chr10:127041931-127045461	33.9716	23.5525	-0.528447	0.0116945	yes
Arfgap2	chr2:91265114-91277371	10.5098	7.28885	-0.527974	0.00984695	yes
Zfp595	chr13:67312997-67332560	4.93126	3.42032	-0.527825	0.0145094	yes
Tlr7	chrX:167304925-167330571	50.7945	35.2321	-0.527782	0.00733312	yes
Ctdsp1	chr1:74391608-74397285	11.4253	7.92613	-0.527536	0.00870444	yes
Gm14420	chr2:177464741-177479194	1.87953	1.30399	-0.527446	0.0262535	yes
Wee1	chr7:110122058-110143299	17.7385	12.3071	-0.527397	0.00699437	yes
Rnf157	chr11:116336344-116413032	5.73649	3.98298	-0.52632	0.0118007	yes
Uqcc1	chr2:155846885-155930310	14.1922	9.85423	-0.526279	0.0080155	yes
Armt1	chr10:4432604-4455140	23.9994	16.6665	-0.526043	0.00915655	yes
Rps3	chr7:99477896-99483709	727.844	505.516	-0.525874	0.0216008	yes
Nt5c	chr11:115475676-115491814	59.9276	41.6372	-0.525348	0.0139859	yes
Tbcc	chr17:46890620-46892463	13.1354	9.12768	-0.52514	0.014404	yes
Arhgap9	chr10:127321963-127341579	51.0771	35.4968	-0.524986	0.00663679	yes
Bin2	chr15:100641081-100669500	13.4696	9.36125	-0.524935	0.00428914	yes
Clec4a3	chr6:122952514-122969878	7.15416	4.9723	-0.52487	0.0332263	yes
Med6	chr12:81573563-81594958	30.4274	21.1493	-0.524758	0.0108186	yes
Ift27	chr15:78159462-78174108	6.60466	4.5909	-0.524708	0.0379901	yes
Fut11	chr14:20694967-20700197	6.36891	4.42737	-0.524596	0.0194933	yes
Taf5	chr19:47067747-47090625	11.7552	8.17554	-0.523915	0.00950212	yes
Cpt1a	chr19:3323300-3385733	9.96657	6.93404	-0.5234	0.00789883	yes
Ercc6l	chrX:102142819-102157091	23.1108	16.0886	-0.522533	0.00566351	yes
Suclg2	chr6:95473008-95718846	18.5575	12.92	-0.52239	0.00755508	yes
Slc25a10	chr11:120491836-120501161	6.4624	4.49945	-0.522321	0.0118007	yes
Gga2	chr7:121986721-122021198	6.64034	4.62441	-0.521988	0.0106073	yes
Tmem208	chr8:105326363-105329057	57.8219	40.2733	-0.521791	0.0126632	yes
Zfp626	chr7:27807195-27822916	4.99178	3.47736	-0.521562	0.0149322	yes
Zer1	chr2:30097282-30124611	1.82349	1.27047	-0.52134	0.0338498	yes
Aasdh	chr5:76875934-76905514	5.22546	3.6414	-0.521065	0.0169236	yes
Psat1	chr19:15905122-15925059	244.849	170.683	-0.520577	0.00938869	yes
Ccdc18	chr5:108132913-108232949	4.61575	3.21765	-0.52056	0.0171279	yes
Mthfsl	chr9:88688604-88719798	15.0687	10.5049	-0.520498	0.0244588	yes
0610009B22Rik	chr11:51685384-51688634	23.8692	16.6422	-0.520298	0.0255556	yes
Rad18	chr6:112619850-112696670	31.119	21.7015	-0.520001	0.00639665	yes
Ryr1	chr7:29003339-29125151	0.605088	0.422092	-0.519587	0.0224699	yes
Oxct1	chr15:4020110-4155344	109.018	76.0619	-0.51932	0.0080155	yes
Scp2	chr4:108043829-108118547	33.2766	23.2325	-0.518363	0.00590647	yes
Pex6	chr17:46711462-46725541	10.4341	7.28642	-0.518028	0.0161122	yes
Myg1	chr15:102331708-102338138	35.5746	24.847	-0.51778	0.0108186	yes
Vps16	chr2:130417683-130444269	35.2351	24.6105	-0.51774	0.0198169	yes
Wdfy2	chr14:62837689-62956886	35.3763	24.7112	-0.517616	0.0189835	yes
Mks1	chr11:87853224-87863679	2.56429	1.79135	-0.517513	0.0338498	yes
Ifrd2	chr9:107587717-107593038	65.5097	45.7847	-0.516842	0.00663679	yes
Fen1	chr19:10199131-10203943	96.7098	67.5908	-0.516836	0.00615568	yes
Lhpp	chr7:132610642-132706419	14.9794	10.4703	-0.516682	0.0123537	yes
Ctnnal1	chr4:56810934-56865211	4.55253	3.18238	-0.516563	0.0186727	yes
Atp6ap2	chrX:12587758-12617051	161.609	112.976	-0.516489	0.00733312	yes
Map2k2	chr10:81105946-81124697	22.6592	15.8405	-0.516474	0.00950212	yes
Pianp	chr6:124996719-125003097	2.80677	1.96249	-0.516229	0.0364231	yes
Klhl21	chr4:152008890-152017677	5.71303	3.99549	-0.515882	0.0188827	yes
Ndufaf4	chr4:24898082-24905001	24.9627	17.4628	-0.515487	0.00723058	yes
Phpt1	chr2:25573430-25574871	23.3404	16.3285	-0.51544	0.0212968	yes
Nudt5	chr2:5845033-5868736	74.2402	51.9388	-0.515389	0.00836289	yes
Tusc3	chr8:39005866-39130817	37.4491	26.202	-0.515253	0.0103944	yes
Mrpl11	chr19:4962305-4966995	19.7544	13.8247	-0.51493	0.0108186	yes
Pms2	chr5:143910000-143931756	17.121	11.9819	-0.514905	0.00723058	yes

Guf1	chr5:69556941-69573630	32.4349	22.6994	-0.514892	0.00733312	yes
Dtnb	chr12:3572390-3781398	6.44019	4.50753	-0.514767	0.0204073	yes
Ppm1f	chr16:16896468-16927375	11.239	7.87091	-0.513908	0.00881201	yes
Hrsp12	chr15:34484021-34495246	19.2807	13.5028	-0.513899	0.0199123	yes
Yif1a	chr19:5088537-5098521	28.2413	19.787	-0.513254	0.0126632	yes
Lap3	chr5:45493373-45512691	60.8124	42.6241	-0.512695	0.00699437	yes
Fam136a	chr6:86365682-86370058	84.9785	59.5642	-0.512652	0.00615568	yes
Epn1	chr7:5080234-5098178	26.8766	18.8492	-0.511847	0.00723058	yes
Zbtb14	chr17:69383977-69390544	10.0813	7.07035	-0.511832	0.00962395	yes
Klf9	chr19:23141225-23166911	9.8959	6.94087	-0.511715	0.0121412	yes
Tor2a	chr2:32757025-32775633	17.0814	11.983	-0.51144	0.0213983	yes
Nfs1	chr2:156123636-156145794	33.7222	23.6582	-0.51136	0.0244588	yes
Mettl15	chr2:109092299-109278290	4.37629	3.07084	-0.511075	0.0363584	yes
Utp18	chr11:93859242-93885766	66.8095	46.8886	-0.510814	0.00733312	yes
Rftn2	chr1:55170159-55226782	2.14063	1.50256	-0.510613	0.0324963	yes
Trp53rka	chr2:165490111-165493314	8.19661	5.75341	-0.510611	0.0232747	yes
Wdr74	chr19:8735838-8740624	47.6093	33.4187	-0.51059	0.00755508	yes
Mrpl47	chr3:32727496-32736755	27.8438	19.5479	-0.510345	0.0123537	yes
Per2	chr1:91415981-91459328	1.02075	0.716739	-0.510104	0.0340124	yes
Tmco1	chr1:167308669-167333978	34.5054	24.2315	-0.509937	0.00733312	yes
Atpif1	chr4:132530554-132535414	466.906	327.966	-0.509586	0.0159005	yes
Mrpl3	chr9:105053267-105077476	72.6572	51.0495	-0.509211	0.00602767	yes
Bzw2	chr12:36091834-36156825	160.004	112.444	-0.508896	0.00578321	yes
Lst1	chr17:35185094-35188440	70.2242	49.3545	-0.508786	0.0267306	yes
Plekhm1	chr11:103365091-103412664	12.5402	8.81812	-0.508021	0.00904622	yes
Exo1	chr1:175880777-175911396	14.7633	10.3834	-0.507729	0.00755508	yes
Pef1	chr4:130107555-130128134	16.8166	11.8311	-0.507306	0.0170277	yes
Smyd2	chr1:189880491-189922288	45.4764	32.0038	-0.506875	0.00710851	yes
Dscr3	chr16:94497723-94526629	20.9167	14.7259	-0.506299	0.0114837	yes
Gm4737	chr16:46152990-46155077	39.7983	28.0215	-0.506175	0.00881201	yes
Ndufv2	chr17:66078794-66101559	173.523	122.222	-0.505624	0.00766958	yes
Psmg2	chr18:67641598-67654162	68.1402	48.0082	-0.505224	0.0107144	yes
Cd2bp2	chr7:127191659-127196077	17.4541	12.2987	-0.505059	0.00778355	yes
Dgcr14	chr16:17900708-17911348	6.9326	4.88521	-0.504975	0.0159005	yes
Smg5	chr3:88336259-88362337	28.682	20.2135	-0.50483	0.00870444	yes
Ipo11	chr13:106794438-106936915	51.6514	36.4162	-0.504226	0.00825328	yes
Aar2	chr2:156547575-156568972	9.63354	6.79746	-0.503071	0.0156913	yes
Smarcd2	chr11:106263170-106272972	40.3303	28.4586	-0.502999	0.00825328	yes
Plin2	chr4:86656564-86670059	21.2683	15.0219	-0.501641	0.0130978	yes
Actr8	chr14:29978336-29993221	19.0344	13.4492	-0.50109	0.0100707	yes
Klf10	chr15:38291463-38300711	21.512	15.2004	-0.501029	0.011269	yes
Ppp1r12c	chr7:4481520-4501680	24.2429	17.139	-0.500279	0.00870444	yes
Bora	chr14:99046376-99074107	25.2689	17.8724	-0.499635	0.0127805	yes
Akip1	chr7:109703736-109723771	23.3585	16.5216	-0.499591	0.0161122	yes
0610037L13Rik	chr4:107889898-107897802	16.377	11.5854	-0.49936	0.0206813	yes
Akt2	chr7:27591559-27639453	7.72911	5.46776	-0.499352	0.0162041	yes
Mark3	chr12:111574509-111656227	33.8498	23.9513	-0.499043	0.00984695	yes
Rae1	chr2:173000116-173015739	42.5495	30.1208	-0.498383	0.00973453	yes
Wdr5b	chr16:36041189-36042974	4.43906	3.14422	-0.497554	0.0442183	yes
Arhgap19	chr19:41766587-41802084	6.1387	4.3482	-0.497514	0.0162041	yes
Lta4h	chr10:93453395-93484896	31.3225	22.1914	-0.497201	0.0106073	yes
Cc2d1b	chr4:108619955-108634122	22.1048	15.6619	-0.497105	0.00847587	yes
Trappc5	chr8:3676476-3680921	28.716	20.3483	-0.496943	0.011374	yes
Camta2,Spag7	chr11:70663768-70688105	44.0599	31.224	-0.496812	0.0165092	yes
Rps15a	chr7:118104375-118116147	13.9775	9.91205	-0.495856	0.015043	yes
Sil1	chr18:35266395-35498925	19.577	13.8839	-0.495745	0.0159005	yes
Comt	chr16:18348181-18479073	34.5208	24.4852	-0.495556	0.0402316	yes
Pqlc1	chr18:80255244-80292724	21.4822	15.2381	-0.495457	0.0183683	yes
Mib2	chr4:155654469-155669254	3.70504	2.62845	-0.495273	0.0269153	yes
Srm	chr4:148591512-148594619	160.416	113.815	-0.49513	0.0103944	yes
Zfp277	chr12:40315045-40445790	17.9488	12.7384	-0.494707	0.0156913	yes
Csrp1	chr1:135729196-135752229	51.3228	36.4319	-0.494397	0.0101798	yes
Zbtb8a	chr4:129353631-129378028	5.30942	3.76953	-0.494168	0.0338498	yes
Gla	chrX:134588168-134601005	7.86161	5.5823	-0.493965	0.0196053	yes
Lsm3	chr6:91516034-91522620	202.448	143.754	-0.493949	0.0133195	yes

Trim28	chr7:13024151-13031032	135.163	95.9924	-0.493712	0.0125567	yes
Copg2	chr6:30733505-30896794	24.7889	17.6078	-0.49348	0.0107144	yes
Raly	chr2:154791109-154867261	175.343	124.549	-0.493469	0.011588	yes
Urgcp	chr11:5713416-5784710	5.26557	3.74089	-0.493206	0.0349096	yes
Foxc1	chr13:31806645-31810635	2.48951	1.76926	-0.492712	0.0444694	yes
Mrpl15	chr1:4773199-4785726	58.5629	41.6271	-0.492465	0.016003	yes
Igbp1	chrX:100494290-100516125	47.9674	34.0986	-0.492341	0.00893132	yes
Micu2	chr14:57916279-57999262	36.5184	25.9625	-0.492192	0.00915655	yes
Higd1a	chr9:121848559-121858000	8.73505	6.21031	-0.492151	0.0345557	yes
Tdp1	chr12:99884514-99955216	16.0272	11.3976	-0.491796	0.0152609	yes
Smc4	chr3:69004971-69034623	108.875	77.4328	-0.491661	0.0162041	yes
Sephs2	chr7:127271878-127274059	184.444	131.183	-0.491602	0.0120252	yes
Tarbp2	chr15:102518191-102523676	5.07281	3.60862	-0.491337	0.0433027	yes
BC003965	chr17:25184560-25187662	13.6587	9.71674	-0.491281	0.0163026	yes
Plk4	chr3:40799950-40816883	31.8108	22.6367	-0.490854	0.011269	yes
Idh1	chr1:65158615-65186479	64.5242	45.9387	-0.490131	0.00904622	yes
Mrpl21	chr19:3283046-3292837	20.7681	14.7933	-0.489422	0.0217808	yes
Suds3	chr5:117091677-117115993	52.7549	37.6035	-0.488439	0.00950212	yes
Cntln	chr4:84884308-85131921	14.0059	9.98334	-0.488435	0.0139859	yes
Fundc2	chrX:75382398-75397158	23.6766	16.8796	-0.488182	0.0168229	yes
Zfp58	chr13:67490166-67500522	4.11226	2.93178	-0.488155	0.0471404	yes
Polr1d	chr5:147077345-147111361	116.635	83.1909	-0.487502	0.00825328	yes
Lipt2	chr7:100159276-100160931	11.8905	8.48711	-0.48646	0.0370511	yes
Yeats4	chr10:117215140-117224507	52.7198	37.6322	-0.486376	0.0104999	yes
Bckdhb	chr9:83925136-84124240	34.5783	24.6951	-0.485641	0.0166035	yes
Zfp862-ps	chr6:48504338-48534832	2.59679	1.85459	-0.485629	0.0328464	yes
1600002H07Rik	chr17:24215055-24220769	5.70668	4.07595	-0.485514	0.0274099	yes
Nudt19	chr7:35547184-35555928	56.9466	40.6879	-0.485011	0.0125567	yes
Sep-06	chrX:36910833-36989695	13.8562	9.90205	-0.484727	0.0146178	yes
Pomgnt1	chr4:116150517-116159844	12.563	8.97868	-0.48461	0.0194933	yes
Dolk	chr2:30284228-30286354	8.53762	6.10289	-0.484341	0.033586	yes
Zbtb48	chr4:152019775-152027671	3.47853	2.48695	-0.484103	0.0442967	yes
1810014B01Rik	chr10:86685526-86689954	9.3618	6.69626	-0.48343	0.031965	yes
Znrf3	chr11:5276328-5444847	1.95817	1.40093	-0.483128	0.0304744	yes
Sdha	chr13:74322254-74350240	144.743	103.595	-0.482542	0.0121412	yes
Pex7	chr10:19860089-19907674	24.4972	17.5342	-0.48245	0.0206813	yes
Chtop	chr3:90498538-90509498	49.1896	35.2107	-0.48234	0.0163026	yes
Nadk2	chr15:9071259-9110496	24.9812	17.9011	-0.480792	0.0171279	yes
Commd5	chr15:76899940-76901297	21.0189	15.0685	-0.480151	0.0292744	yes
1700017B05Rik	chr9:57252321-57262599	10.9733	7.86732	-0.480051	0.0165092	yes
B3glct	chr5:149678256-149762599	5.52943	3.96513	-0.479762	0.0209746	yes
Actr1b	chr1:36699201-36709925	44.4869	31.9015	-0.479757	0.011374	yes
Psma7	chr2:180036366-180042464	345.879	248.071	-0.479513	0.0181607	yes
Chmp3	chr6:71543853-71581574	44.1447	31.6746	-0.478912	0.0120252	yes
1110004E09Rik	chr16:90925810-90934849	38.1802	27.3975	-0.47878	0.0181607	yes
Tfam	chr10:71225476-71238044	27.9864	20.0844	-0.47865	0.0123537	yes
B230354K17Rik	chr17:45433830-45442544	6.75588	4.84912	-0.478422	0.0193933	yes
Nanos1	chr19:60755986-60759914	7.10179	5.09889	-0.478001	0.0272102	yes
Tmem234	chr4:129600706-129607879	49.9977	35.9006	-0.477854	0.0218594	yes
Fam109a	chr5:121849027-121854599	4.37257	3.14003	-0.477701	0.0496925	yes
Tomm22	chr15:79670867-79672862	88.2237	63.3651	-0.477478	0.012881	yes
Umps	chr16:33955011-33967003	56.7249	40.7466	-0.477302	0.0101798	yes
Rp9	chr9:22448311-22468356	48.2984	34.6974	-0.477145	0.0149322	yes
Bag2	chr1:33745483-33757750	44.733	32.1436	-0.476807	0.011588	yes
Rnf220	chr4:117271463-117496915	25.2276	18.1298	-0.47664	0.0119065	yes
Ndufs4	chr13:114287794-114388094	42.8421	30.7913	-0.476507	0.0199123	yes
Phkb	chr8:85841001-86060642	12.8719	9.25125	-0.476504	0.0136535	yes
Srsf2	chr11:116849896-116853094	350.04	251.684	-0.475909	0.0170277	yes
Clk1	chr1:58411987-58424088	94.4554	67.9163	-0.475875	0.0205017	yes
Dap	chr15:31224384-31274338	35.5258	25.5459	-0.475778	0.0169236	yes
Eif2d	chr1:131153206-131173471	39.4782	28.3999	-0.475167	0.0137694	yes
Scarb1	chr5:125277086-125341094	37.1671	26.7465	-0.474677	0.0143075	yes
Wdr12	chr1:60076867-60153953	27.5141	19.8089	-0.474025	0.033762	yes
Galk2	chr2:125859108-126152004	25.8799	18.6343	-0.47387	0.0146178	yes
Recql	chr6:142345696-142387087	28.2717	20.357	-0.473837	0.0378013	yes

Smc2	chr4:52439220-52488365	122.487	88.2098	-0.473613	0.0263336	yes
Klhl42	chr6:147091074-147112778	7.4021	5.33128	-0.473451	0.0214996	yes
Pola1	chrX:93304765-93632155	56.4948	40.6922	-0.473366	0.0179488	yes
Anapc2	chr2:25272465-25285916	14.1679	10.2097	-0.472688	0.0218594	yes
Alyref2	chr1:171503477-171504750	8.39556	6.05017	-0.472651	0.0394561	yes
Shmt1	chr11:60788896-60811265	26.9071	19.3958	-0.47224	0.0159005	yes
Gpn1	chr5:31494760-31511627	38.6153	27.8379	-0.47212	0.0164008	yes
Tsta3	chr15:75924682-75929730	23.0888	16.6491	-0.471749	0.0264395	yes
Mkx	chr18:6934965-7004779	3.32707	2.39985	-0.471309	0.0418557	yes
Tprn	chr2:25262597-25269886	6.64911	4.79655	-0.471163	0.0321137	yes
Sclt1	chr3:41626704-41742514	14.3279	10.3361	-0.471136	0.0209746	yes
Coil	chr11:88973934-88991613	11.118	8.02056	-0.471125	0.0219511	yes
L3mbtl2	chr15:81663888-81697287	18.9347	13.6597	-0.471101	0.0271155	yes
Maea	chr5:33335571-33373294	45.9387	33.1558	-0.47045	0.0172208	yes
Clec12a	chr6:129350243-129365303	117.962	85.1524	-0.470201	0.0166035	yes
Tbc1d4	chr14:101442359-101609191	4.44135	3.20614	-0.470164	0.0265349	yes
Eri2	chr7:119760922-119794058	9.73135	7.02556	-0.470026	0.0216975	yes
Mcm2	chr6:88883473-88898780	81.9278	59.1493	-0.469991	0.0141985	yes
Iffo1	chr6:125145223-125161782	4.03775	2.91584	-0.469639	0.042684	yes
Ubac1	chr2:25996957-26021760	12.4803	9.01469	-0.469306	0.0208855	yes
Map3k11	chr19:5689130-5702864	12.1089	8.74725	-0.469168	0.0204073	yes
Pes1	chr11:3963974-3980004	29.6066	21.3877	-0.469133	0.0129867	yes
Puf60	chr15:76070181-76080946	85.0161	61.4179	-0.469077	0.0130978	yes
C2cd5	chr6:143010919-143100152	10.5969	7.65637	-0.468911	0.0205916	yes
Atg101	chr15:101284300-101290934	25.8129	18.6511	-0.468831	0.0259557	yes
Siae	chr9:37613846-37648318	8.80898	6.36503	-0.468808	0.0241012	yes
Atl3	chr19:7494039-7538609	38.0228	27.4797	-0.468501	0.016003	yes
Nup133	chr8:123897122-123949265	17.4354	12.604	-0.468134	0.012881	yes
Plxnb2	chr15:89155545-89180788	49.058	35.4839	-0.467326	0.0175233	yes
Zfp930	chr8:69209045-69230539	17.3235	12.536	-0.466647	0.0191808	yes
Cep78	chr19:15955772-15984989	25.5383	18.4807	-0.466646	0.0138763	yes
Gas2l3	chr10:89408822-89443967	20.5013	14.8359	-0.466628	0.0148245	yes
Dhx35	chr2:158794806-158858220	10.7723	7.79738	-0.466262	0.0234724	yes
Unc50	chr1:37430171-37439124	56.7098	41.0612	-0.465823	0.0241012	yes
Lypla2	chr4:135968224-135972594	22.7446	16.4713	-0.465572	0.0342942	yes
Rilpl1	chr5:124493079-124531391	10.7682	7.80017	-0.465206	0.0243709	yes
Focad	chr4:88094629-88411011	7.33169	5.31148	-0.465033	0.0199123	yes
Dus4l	chr12:31640054-31654826	8.79691	6.37545	-0.464468	0.0300914	yes
Zfp367	chr13:64133056-64153199	34.1011	24.7324	-0.463418	0.0156913	yes
Zfp65	chr13:67705305-67729173	5.32354	3.8614	-0.46326	0.0334032	yes
Cdc34	chr10:79682194-79688398	45.8761	33.2803	-0.463074	0.0181607	yes
Gcsh	chr8:116981827-116993449	98.7721	71.6621	-0.462893	0.0149322	yes
Prdx3	chr19:60864065-60874538	130.161	94.476	-0.462279	0.0207877	yes
Lig1	chr7:13258966-13311427	40.5857	29.4623	-0.4621	0.0176188	yes
Itpk1	chr12:102568582-102704869	8.88766	6.45341	-0.461742	0.0244588	yes
Agap3	chr5:24452176-24502047	19.9287	14.4709	-0.461686	0.0186727	yes
Vbp1	chrX:75514296-75534946	66.0472	47.9972	-0.460547	0.0203128	yes
Sergef	chr7:46443158-46639807	12.8682	9.35182	-0.460491	0.0409198	yes
Syngr2	chr11:117809666-117814286	82.0463	59.6279	-0.46045	0.0164008	yes
Akr1a1	chr4:116636509-116651674	392.619	285.377	-0.460261	0.0263336	yes
Csnk2a1	chr2:152226839-152281851	30.0124	21.8151	-0.460233	0.0151569	yes
Asna1	chr8:85017930-85025278	85.4547	62.1153	-0.460212	0.0143075	yes
Tefm	chr11:80136677-80142153	13.611	9.89376	-0.460182	0.042684	yes
Fam96a	chr9:66126610-66138968	195.933	142.439	-0.460015	0.0147323	yes
Acsl5	chr19:55253368-55296628	138.98	101.061	-0.459642	0.0247412	yes
Coasy	chr11:101082564-101086619	15.5988	11.3433	-0.459591	0.032407	yes
Ninj1	chr13:49187546-49196251	37.8351	27.5144	-0.459541	0.0245514	yes
Rbak	chr5:143172185-143180775	2.90268	2.11154	-0.459086	0.042047	yes
Slc15a4	chr5:127595665-127617392	25.4655	18.5268	-0.458928	0.0200117	yes
Zbtb24	chr10:41450357-41465582	6.09199	4.43376	-0.458383	0.0465131	yes
9130401M01Rik	chr15:58022270-58034294	37.2763	27.1344	-0.458134	0.0226558	yes
Gpr180	chr14:118137126-118164232	11.2475	8.18807	-0.458009	0.0321137	yes
Pdik1l	chr4:134275004-134287846	23.3198	16.978	-0.457886	0.0170277	yes
Git1	chr11:77493411-77507774	15.2727	11.1221	-0.457524	0.0219511	yes
Wwox	chr8:114439651-115352712	6.59535	4.8058	-0.456673	0.0365931	yes

Ncf1	chr5:134220052-134229625	127.155	92.6698	-0.45642	0.0180585	yes
Tpra1	chr6:88902250-88912240	8.72815	6.36121	-0.456375	0.0449001	yes
9430038I01Rik	chr7:137375568-137410756	16.6141	12.1091	-0.456309	0.0370511	yes
Mettl25	chr10:105763184-105841380	9.2071	6.71376	-0.455626	0.0392619	yes
Slc16a10	chr10:40033534-40142254	29.9487	21.8402	-0.455512	0.0182627	yes
Pold2	chr11:5861865-5878256	55.5276	40.4979	-0.455357	0.0176188	yes
Ankrd46	chr15:36477667-36496791	13.0588	9.52439	-0.455323	0.0341064	yes
Dusp3	chr11:101971143-101984791	23.4294	17.0915	-0.45504	0.0177326	yes
Sap30l	chr11:57801636-57810615	13.4412	9.80588	-0.454946	0.0390137	yes
Sec11c	chr18:65800577-65817657	219.887	160.418	-0.454927	0.0170277	yes
Yif1b	chr7:29238322-29248467	46.8389	34.1733	-0.45484	0.0241896	yes
Gemin4	chr11:76210570-76217572	17.1486	12.5116	-0.454825	0.0213983	yes
Pfdn5	chr15:102326115-102331489	68.3093	49.858	-0.454259	0.0222609	yes
Tmem64	chr4:15265819-15286753	20.2039	14.7503	-0.453896	0.0225652	yes
Ddx41	chr13:55530409-55536658	70.7093	51.6312	-0.453655	0.0184699	yes
Mtrf1l	chr10:5811886-5823943	6.00314	4.38397	-0.453479	0.0404022	yes
Nipal3	chr4:135448900-135494504	3.6121	2.63845	-0.453147	0.0399787	yes
Tcf20	chr15:82808625-82912134	46.7462	34.1544	-0.452775	0.0280844	yes
Bend3	chr10:43479139-43515417	4.41609	3.22696	-0.452592	0.0324963	yes
Glrx3	chr7:137437647-137468594	22.7225	16.6043	-0.452562	0.0300114	yes
Lias	chr5:65391496-65409207	20.393	14.9095	-0.451839	0.0227508	yes
Zmynd8	chr2:165784151-165884838	23.7536	17.3698	-0.451563	0.0443912	yes
Cbr4	chr8:61487733-61503500	16.3062	11.9285	-0.451006	0.0482697	yes
Dram1	chr10:88322803-88357075	29.4878	21.5713	-0.451005	0.0197173	yes
Nubp2	chr17:24882610-24886350	65.0682	47.6089	-0.45072	0.0188827	yes
Ppp4c	chr7:126785867-126792471	147.062	107.612	-0.450585	0.0203128	yes
Haus1	chr18:77757566-77767780	59.0433	43.2148	-0.450248	0.0225652	yes
Fastk	chr5:24441039-24445235	17.5854	12.8743	-0.449882	0.0327512	yes
Tbc1d32	chr10:56014293-56228689	4.14803	3.038	-0.449306	0.0332263	yes
Rps25	chr9:44407713-44418007	107.134	78.4713	-0.449179	0.0280844	yes
Mid1ip1	chrX:10717364-10719702	21.7764	15.9505	-0.449167	0.0259557	yes
Calm2	chr17:87433400-87446935	411.082	301.355	-0.447961	0.0239982	yes
Cdk5rap2	chr4:70223023-70410367	13.4938	9.89233	-0.447911	0.0192851	yes
Zbtb4	chr11:69765911-69784026	3.46988	2.54381	-0.447892	0.0325793	yes
Snrpd1	chr18:10617795-10628230	162.453	119.143	-0.447328	0.0211866	yes
Fsbp,Rad54b	chr4:11558919-11615808	17.366	12.7478	-0.446014	0.0261526	yes
N6amt2	chr14:57549597-57571569	45.5514	33.4527	-0.445371	0.0355539	yes
Apmap	chr2:150583080-150608523	29.1356	21.4017	-0.445057	0.0237919	yes
Cfap20	chr8:95420249-95434869	39.406	28.9487	-0.444915	0.0218594	yes
Arl2	chr19:6134388-6141137	28.2424	20.7479	-0.444897	0.0365931	yes
Stt3b	chr9:115242581-115310421	81.0722	59.574	-0.444525	0.0206813	yes
Exosc2	chr2:31670736-31681307	44.6725	32.8308	-0.444336	0.0181607	yes
Cenpt	chr8:105844677-105852008	8.66611	6.36973	-0.444153	0.0472165	yes
Col4a3bp	chr13:96542734-96640167	24.6992	18.1646	-0.443334	0.0174348	yes
Dnase1l1	chrX:74273216-74282333	15.3316	11.2759	-0.443271	0.0346427	yes
Ttc4	chr4:106661807-106678944	18.4267	13.5603	-0.442408	0.0224699	yes
C1qbp	chr11:70970199-70983026	190.526	140.235	-0.442142	0.0263336	yes
Pcnt	chr10:76351253-76442912	8.59614	6.32794	-0.441953	0.0216008	yes
Angel2	chr1:190925107-190946491	22.2105	16.3552	-0.441488	0.0283815	yes
Cib1	chr7:80227155-80232805	30.2452	22.2777	-0.441107	0.0469626	yes
Nudt4	chr10:95547006-95564167	48.2492	35.551	-0.440615	0.0190803	yes
Polr2i	chr7:30232073-30236765	43.7482	32.2374	-0.440487	0.0323114	yes
Me2	chr18:73770039-73815392	104.817	77.2399	-0.440451	0.0257635	yes
Rabepk	chr2:34778665-34799912	9.40392	6.93655	-0.439044	0.0346427	yes
Klhdc4	chr8:121796307-121829569	26.199	19.333	-0.438445	0.0246488	yes
Rcbtb1	chr14:59201227-59237265	14.3452	10.5885	-0.438071	0.0280844	yes
Akap8l	chr17:32321423-32350577	9.30166	6.86581	-0.438059	0.0415762	yes
Fam134c	chr11:101096321-101119843	13.9812	10.3202	-0.438017	0.0357258	yes
Numa1	chr7:101969842-102014959	27.1047	20.0127	-0.437628	0.0234724	yes
Cdc26	chr4:62394588-62408623	20.6283	15.243	-0.436477	0.0474025	yes
Mgat1	chr11:49244190-49263024	12.9735	9.58766	-0.436321	0.0480846	yes
Tspan14	chr14:40906443-40966807	21.6712	16.0179	-0.436091	0.0275042	yes
Ext2	chr2:93695630-93822568	16.0401	11.8561	-0.436052	0.0297474	yes
Necap2	chr4:141066511-141078345	29.7339	21.9825	-0.435754	0.0269153	yes
Ddx51	chr5:110653450-110660496	9.2473	6.83718	-0.43563	0.0372338	yes

Nudt3	chr17:27579381-27623452	67.648	50.0565	-0.434491	0.0226558	yes
Pdf	chr8:107046289-107048614	29.5148	21.8468	-0.434016	0.0344621	yes
Cdc16	chr8:13757689-13781882	47.0315	34.8194	-0.433736	0.0218594	yes
Nol12	chr15:78934932-78941910	16.9599	12.5608	-0.433199	0.0291875	yes
Zbed4	chr15:88751710-88784516	5.96637	4.41985	-0.432854	0.0356533	yes
Aprt	chr8:122574636-122576907	321.901	238.517	-0.432529	0.0265349	yes
Commd2	chr3:57644348-57651684	6.92962	5.13467	-0.432505	0.0499323	yes
Noc2l	chr4:156236009-156255338	95.0223	70.412	-0.432445	0.0260568	yes
Mrps30	chr13:118380109-118387252	61.7549	45.7824	-0.43176	0.0242828	yes
Tcf12	chr9:71844251-72111819	45.0598	33.409	-0.431603	0.0263336	yes
Pdlim2	chr14:70164217-70177672	20.9118	15.5071	-0.431389	0.0364231	yes
Akap1	chr11:88830791-88881395	17.9965	13.3483	-0.431065	0.0269153	yes
Hmgb3	chrX:71555916-71560673	16.8097	12.4701	-0.43082	0.0432236	yes
Nhp2l1	chr15:82041344-82047598	93.9396	69.7273	-0.430009	0.0300914	yes
Cep68	chr11:20227036-20249424	12.6927	9.42345	-0.429673	0.0272102	yes
Nup205	chr6:35177615-35247598	85.1452	63.2258	-0.429412	0.0340124	yes
Tln2	chr9:67217084-67559703	2.63421	1.95615	-0.429352	0.0342942	yes
Eif4h	chr5:134619875-134639328	202.377	150.303	-0.429174	0.0347296	yes
Ccdc32	chr2:119017778-119029393	10.4151	7.73603	-0.429006	0.0438319	yes
Otub1	chr19:7198205-7206284	56.3433	41.8602	-0.428663	0.0261526	yes
Ncapg2	chr12:116405401-116463531	53.7034	39.9075	-0.428352	0.0275985	yes
Zfp84	chr7:29768551-29781419	9.40306	6.99323	-0.42717	0.0280844	yes
Ptpra	chr2:130450277-130563749	81.5037	60.6209	-0.427049	0.0276926	yes
Usp28	chr9:48985384-49042517	6.24749	4.6476	-0.426792	0.0468864	yes
Gpatch3	chr4:133574744-133584242	8.61284	6.40833	-0.426542	0.048798	yes
1700066M21Rik	chr1:57377619-57385422	11.6019	8.64189	-0.424947	0.0428589	yes
Ssr4	chrX:73787027-73790828	222.687	165.875	-0.424921	0.0294593	yes
Rnh1	chr7:141160325-141172851	79.5016	59.2228	-0.424832	0.0355539	yes
Gipc1	chr8:83652677-83664789	50.3261	37.4895	-0.424818	0.0298509	yes
Zfp512	chr5:31452435-31481753	12.9708	9.67218	-0.423355	0.0350894	yes
Rtel1	chr2:181319723-181356616	20.6697	15.4214	-0.422586	0.0320425	yes
Parvb	chr15:84232042-84315609	5.35126	3.99582	-0.421387	0.0477311	yes
Zfp60	chr7:27731408-27751689	8.29469	6.19419	-0.421271	0.044986	yes
Akr1b10	chr6:34384246-34396949	16.3473	12.2111	-0.420853	0.0462412	yes
Smarce1	chr11:99209047-99231017	18.5883	13.8871	-0.420644	0.0370511	yes
Foxred2	chr15:77940521-77956722	4.65123	3.47571	-0.420304	0.0461477	yes
Rpl14-ps1	chr7:45303154-45333780	1151.9	860.978	-0.419965	0.0497577	yes
Mrps22	chr9:98588729-98601679	77.5788	58.0192	-0.419133	0.0312726	yes
Tmem43	chr6:91473750-91488458	18.3391	13.7161	-0.419052	0.0326747	yes
Psmd5	chr2:34852088-34870962	70.5345	52.7957	-0.417909	0.0270102	yes
Ggta1	chr2:35400178-35461449	21.3099	15.9544	-0.417574	0.0348164	yes
Coa3	chr11:101277969-101278948	112.823	84.5272	-0.416569	0.0358116	yes
Manba	chr3:135485610-135571403	21.5048	16.1147	-0.416282	0.0320425	yes
Irf8	chr8:120736357-120756692	15.3037	11.4719	-0.415778	0.0379901	yes
Nap1l1	chr10:111473191-111498150	70.2673	52.6925	-0.415255	0.0433818	yes
Nudt9	chr5:104046863-104065378	45.2035	33.9128	-0.414603	0.0326747	yes
Hira	chr16:18876749-18970308	15.1345	11.3602	-0.413854	0.0424213	yes
Zfp598	chr17:24669751-24682016	12.8692	9.66405	-0.413219	0.0397251	yes
Bloc1s5	chr13:38602705-38635109	21.4277	16.0981	-0.412591	0.0454066	yes
Ubxn6	chr17:56068252-56074989	21.6267	16.257	-0.411753	0.0402316	yes
Lrrk1	chr7:66258746-66388341	4.75795	3.5773	-0.411471	0.0427793	yes
Fam126b	chr1:58522805-58586333	18.4249	13.8529	-0.411463	0.0331378	yes
Ssna1	chr2:25271038-25272418	69.6159	52.3512	-0.411195	0.0369734	yes
Cops8	chr1:90603424-90613341	50.4427	37.955	-0.410354	0.0395532	yes
Cep70	chr9:99243467-99300403	15.9453	11.9985	-0.410269	0.0487144	yes
Hars2	chr18:36783208-36792560	12.099	9.10503	-0.41015	0.0497577	yes
Ube2m	chr7:13035119-13038275	70.6453	53.2215	-0.408583	0.0367903	yes
Wdr91	chr6:34880425-34910831	21.0366	15.8523	-0.408207	0.0391938	yes
Cpped1	chr16:11803720-11930594	15.3628	11.5779	-0.408075	0.041488	yes
Fam207a	chr10:77486654-77515813	17.3257	13.0581	-0.40797	0.0475883	yes
Reep5	chr18:34344885-34373415	33.721	25.4162	-0.407903	0.035282	yes
Cad	chr5:31054779-31078479	21.9433	16.5411	-0.407726	0.0437531	yes
Rasa2	chr9:96539299-96631503	22.6812	17.1143	-0.406292	0.0330554	yes
Aaas	chr15:102338246-102350759	33.5219	25.3025	-0.405825	0.0397999	yes
Traf7	chr17:24488782-24527938	30.3051	22.8751	-0.405784	0.0392619	yes

Bcap31	chrX:73686182-73716175	136.89	103.357	-0.405384	0.033586	yes
Slc4a2	chr5:24425231-24440947	12.349	9.32405	-0.40537	0.0424213	yes
Snrpb2	chr2:143063068-143072052	114.22	86.2608	-0.405035	0.0357258	yes
Tmed9	chr13:55593134-55597694	70.7297	53.4608	-0.403833	0.0426043	yes
Mrpl18	chr17:12911354-12916091	179.32	135.541	-0.403812	0.0365931	yes
Tuba1c	chr15:99029890-99038105	62.4789	47.3011	-0.401496	0.0396355	yes
Vps13c	chr9:67840395-67995634	20.035	15.172	-0.401113	0.0418557	yes
Mrpl44	chr1:79776017-79781445	36.9693	27.9984	-0.400984	0.0452271	yes
Spg7	chr8:123065507-123097751	28.3036	21.4365	-0.400918	0.0392619	yes
Cd99l2	chrX:71420059-71492849	13.156	9.97014	-0.40004	0.0456024	yes
Ppil1	chr17:29250834-29263971	83.4747	63.2896	-0.399371	0.0377174	yes
Mrps17	chr5:129715527-129718691	48.8286	37.0245	-0.399248	0.0491492	yes
Cetn3	chr13:81783291-81797157	172.676	130.949	-0.399065	0.0358116	yes
Uba2	chr7:34140696-34168529	136.629	103.645	-0.398609	0.0416643	yes
Pdxk	chr10:78436746-78464948	11.6892	8.88077	-0.396416	0.0432236	yes
Slc30a5	chr13:100802647-100833427	80.1998	60.9711	-0.395473	0.0374232	yes
Zfp236	chr18:82593596-82692734	5.10045	3.88108	-0.394166	0.0476469	yes
Arpc4,Ttll3	chr6:113378112-113414571	135.441	103.079	-0.393915	0.0476469	yes
Farsa	chr8:84856985-84869257	51.5883	39.2806	-0.393226	0.0391038	yes
Mtmr4	chr11:87592216-87616296	12.2326	9.31461	-0.393162	0.0452271	yes
Lancl2	chr6:57702454-57739449	18.557	14.1377	-0.392417	0.0442967	yes
Mrpl45	chr11:97315715-97329920	34.5341	26.3307	-0.391273	0.0477311	yes
Pkp2	chr16:16213344-16272712	19.3802	14.7835	-0.390593	0.0456796	yes
Ccz1	chr5:143987908-144014853	62.4278	47.6327	-0.390238	0.0445395	yes
Axin1	chr17:26138685-26195811	15.3175	11.6874	-0.39023	0.0465981	yes
Hspa14	chr2:3488853-3512814	80.9436	61.7785	-0.389814	0.0449001	yes
Wdr5	chr2:27515146-27536538	42.7335	32.6832	-0.38682	0.0471404	yes
Vwa5a	chr9:38718267-38743337	57.7024	44.1645	-0.385745	0.0496925	yes
Ehmt1	chr2:24790768-24919609	13.0098	9.96346	-0.384879	0.0486308	yes
Gtf3c4	chr2:28822299-28840360	18.9772	14.5356	-0.384674	0.0445395	yes
Thumpd1	chr7:119715093-119720798	48.0037	36.7868	-0.383958	0.0423414	yes
Snrnp40	chr4:130360131-130390030	80.9126	62.0226	-0.38357	0.044986	yes
Cotl1	chr8:119809213-119840579	97.7403	75.0053	-0.381961	0.0485558	yes
Elp2	chr18:24603960-24638830	75.2516	57.8728	-0.378836	0.0491492	yes
Il1a	chr2:129297369-129309972	0.129177	314.274	11.2485	0.000195236	yes
Csf3	chr11:98701312-98703629	0.0689723	74.6788	10.0805	0.0080155	yes
Ccl5	chr11:83525778-83530518	0.251773	219.322	9.76671	0.00377278	yes
Irg1	chr14:103047011-103056573	0.663499	576.451	9.76289	0.000195236	yes
Il1b	chr2:129364579-129375733	0.697491	484.11	9.43894	0.000195236	yes
Gbp2b	chr3:142594846-142619176	0.0160159	10.1888	9.31326	0.0358116	yes
Rsad2	chr12:26442742-26456452	0.0526843	30.7622	9.18957	0.000195236	yes
Oasl2	chr5:114896933-114912245	0.0548852	27.5886	8.97344	0.000195236	yes
Cmpk2	chr12:26469214-26479837	0.0674089	20.6917	8.2619	0.000195236	yes
Ifit3	chr19:34583528-34588982	0.0969827	29.3054	8.23923	0.000195236	yes
Ccl22	chr8:94745683-94751388	0.127643	36.7111	8.16795	0.000195236	yes
Ifit1	chr19:34640888-34650009	0.229742	65.9083	8.1643	0.000195236	yes
Isg15	chr4:156199423-156200818	0.251238	70.1397	8.12503	0.000195236	yes
Pyhin1	chr1:173630858-173647928	0.0183135	4.80582	8.03573	0.00504708	yes
Lix1	chr17:17402685-17459388	0.0511995	12.8973	7.97673	0.000195236	yes
Usp18	chr6:121245905-121270917	0.722188	179.888	7.96051	0.000195236	yes
Ifi44	chr3:151730922-151749959	0.478193	114.975	7.90951	0.000195236	yes
Lcn2	chr2:32384636-32387739	0.336464	78.2176	7.8609	0.000195236	yes
Ifi202b	chr1:173962568-173982844	0.962352	220.377	7.83919	0.000195236	yes
Rtp4	chr16:23609918-23614222	0.319184	70.3318	7.78364	0.000195236	yes
Ifit1bl2	chr19:34617050-34640743	0.0177709	3.79486	7.73838	0.0161122	yes
Ifi44l	chr3:151758736-151762891	0.193773	37.9226	7.61255	0.000195236	yes
Cxcl2	chr5:90903898-90905938	0.868313	161.654	7.54048	0.000195236	yes
Slfn5	chr11:82911252-82964850	0.387066	60.4522	7.28707	0.000195236	yes
Isg20	chr7:78913423-78920396	0.232974	34.7257	7.21969	0.000195236	yes
Mnda	chr1:173896340-173913046	0.124727	17.6202	7.14232	0.000195236	yes
Ddx60	chr8:61928086-62037701	0.0493568	6.60003	7.06308	0.000195236	yes
Ccl4	chr11:83662583-83664683	10.7006	1409.38	7.04123	0.000195236	yes
Irf7	chr7:141263182-141266424	0.84994	109.214	7.00558	0.000195236	yes
Ccl7	chr11:82045711-82047523	0.40335	44.279	6.77845	0.000195236	yes
Gbp7	chr3:142530335-142550151	0.388898	42.573	6.7744	0.000195236	yes

Ccl3	chr11:83647842-83649378	68.9739	7390.16	6.74341	0.000195236	yes
Gbp9	chr5:105078393-105110292	0.0247171	2.55992	6.69445	0.000855787	yes
Upp1	chr11:9118007-9136170	0.230095	23.0605	6.64705	0.000195236	yes
Lgals9	chr11:78962978-78984924	0.655229	62.8974	6.58486	0.000195236	yes
Ddx58	chr4:40203776-40239825	1.40741	125.269	6.47585	0.000195236	yes
Gbp3	chr3:142560051-142573212	0.387372	31.2275	6.33295	0.000195236	yes
Bcl2a1a	chr9:88956919-88962416	0.182962	12.5566	6.10076	0.000855787	yes
Ptgs2	chr1:150100123-150108012	4.55088	311.717	6.09795	0.000195236	yes
Ifi204	chr1:173747293-173766919	1.07285	70.2262	6.03248	0.000195236	yes
Il1f9	chr2:24186475-24193567	0.0468504	3.00374	6.00256	0.00915655	yes
Gvin1	chr7:105895118-105953970	0.0135922	0.871067	6.00194	0.0354745	yes
Cd300e	chr11:115051916-115062038	0.330283	19.9901	5.91944	0.000195236	yes
Cd40	chr2:165055635-165071654	1.15331	66.83	5.85665	0.000195236	yes
Cish	chr9:107296688-107301961	0.0328108	1.84241	5.81128	0.00996804	yes
H2-T24	chr17:35994503-36038174	1.3403	73.3355	5.77388	0.000195236	yes
Ccl2	chr11:82035576-82037452	23.6165	1279.68	5.75984	0.000195236	yes
Ifih1	chr2:62595792-62646255	0.753845	40.5329	5.74868	0.000195236	yes
Gm14023	chr2:129297369-129309972	0.0905296	4.36925	5.59285	0.0321137	yes
Oas2	chr5:120730332-120749848	0.622837	29.5221	5.5668	0.000195236	yes
Dhx58	chr11:100694883-100704271	0.988244	44.7931	5.50227	0.000195236	yes
Hcar2	chr5:123863569-123865516	0.683664	26.6794	5.28629	0.000195236	yes
Cxcl3	chr5:90786100-90788093	0.0743226	2.8562	5.26415	0.0303714	yes
Gbp2	chr3:142620662-142638008	0.18854	7.02554	5.21967	0.000195236	yes
Trim34b	chr7:104329470-104336617	0.0928408	3.35668	5.17613	0.000195236	yes
Slc15a3	chr19:10842543-10869779	3.26416	116.733	5.16036	0.000195236	yes
Lipg	chr18:74939321-74961263	0.0255388	0.862902	5.07844	0.00100977	yes
Vnn3	chr10:23851461-23869843	0.137857	4.54214	5.04213	0.000195236	yes
Irgm2	chr11:58214976-58222783	0.694099	22.35	5.00899	0.000195236	yes
Oas3	chr5:120753097-120777659	1.96393	62.4996	4.99203	0.000195236	yes
Trim30a	chr7:104409025-104465193	4.13195	130.345	4.97937	0.000195236	yes
Xaf1	chr11:72301628-72313733	0.438402	13.0063	4.89081	0.000195236	yes
Socs3	chr11:117966086-117969366	5.89253	174.375	4.88716	0.000195236	yes
Scimp	chr11:70790931-70812561	0.364093	10.696	4.87662	0.000195236	yes
Rhobtb1	chr10:69208551-69291784	0.0586875	1.69699	4.85378	0.000195236	yes
Il27	chr7:126589294-126594910	0.249729	7.12098	4.83364	0.000195236	yes
Procr	chr2:155751216-155755478	1.75354	49.9733	4.83281	0.000195236	yes
AI607873	chr1:173723427-173741809	0.934987	26.1739	4.80704	0.000195236	yes
Rnf125	chr18:20944624-20983848	0.137924	3.83692	4.79801	0.000195236	yes
Phlda1	chr10:111506285-111508649	0.257633	6.80629	4.72348	0.000195236	yes
Parp14	chr16:35832877-35871382	2.35569	61.7231	4.71159	0.000195236	yes
Cd274	chr19:29367437-29388094	0.203334	4.89899	4.59056	0.000195236	yes
Dusp5	chr19:53529317-53541322	1.59687	37.9919	4.57237	0.000195236	yes
Cxcl10	chr5:92331840-92414627	1.11522	26.0136	4.54387	0.000195236	yes
Il1rn	chr2:24336859-24351491	2.72498	62.906	4.52888	0.000195236	yes
Ifi203	chr1:173920400-173942672	3.03177	65.8341	4.4406	0.000195236	yes
Igtp	chr11:58199555-58207592	0.94629	19.5214	4.36663	0.000195236	yes
Gm12250	chr11:58183842-58190198	0.064746	1.2501	4.27111	0.000195236	yes
Nfkbiz	chr16:55811376-55838641	2.917	55.5149	4.25032	0.000195236	yes
Gstt1	chr10:75783812-75798584	0.712026	13.5374	4.24888	0.000195236	yes
Mmp13	chr9:7272513-7283333	0.364731	6.62074	4.18209	0.000195236	yes
Irgm1	chr11:48865248-48871346	5.42531	95.4868	4.13752	0.000195236	yes
Trim34a	chr7:104244456-104262236	0.259797	4.50234	4.11522	0.000195236	yes
Phf11d	chr14:59347406-59365490	5.9817	103.299	4.11012	0.000195236	yes
Csf1	chr3:107741047-107760469	0.131223	2.26018	4.10634	0.000195236	yes
Fam214b	chr4:43032413-43046220	0.577265	9.93343	4.10499	0.000195236	yes
Slfn8	chr11:83002157-83020810	3.78814	63.1323	4.05882	0.000195236	yes
Ifit2	chr19:34550693-34576534	3.47032	57.6053	4.05306	0.000195236	yes
Arid5a	chr1:36307732-36324029	0.317048	5.12609	4.01509	0.000195236	yes
Ccrl2	chr9:111054833-111057518	0.213092	3.37947	3.98725	0.000195236	yes
Slfn4	chr11:83175185-83190216	11.6495	184.739	3.98714	0.000195236	yes
Sdc3	chr4:130792536-130826318	0.479128	7.58564	3.98479	0.000195236	yes
Csprs	chr1_GL456221_random:11157	0.139218	2.16635	3.95985	0.000195236	yes
Tpbg	chr9:85842379-85847055	0.0587781	0.897269	3.93219	0.000195236	yes
Lilr4b	chr10:51480611-51486329	7.01594	105.713	3.91337	0.000195236	yes
Siglec1	chr2:131069219-131086765	0.103266	1.54968	3.90753	0.000195236	yes

Phf11a	chr14:59276912-59297522	0.0518239	0.775695	3.9038	0.0172208	yes
Stat2	chr10:128270575-128292849	2.58839	38.2528	3.88543	0.000195236	yes
Srgn	chr10:62494427-62507755	93.0178	1371.43	3.88203	0.000195236	yes
Oasl1	chr5:114923239-114937911	2.81133	41.3649	3.87908	0.000195236	yes
AW011738	chr4:156203283-156206028	0.0689904	0.985461	3.83633	0.000195236	yes
Sema3e	chr5:14025275-14256689	0.17149	2.43283	3.82643	0.000195236	yes
Mir155,Mir155hg	chr16:84713022-84715244	0.951139	13.4594	3.82282	0.000195236	yes
Stx11	chr10:12939982-12964259	1.01069	14.0949	3.80177	0.000195236	yes
Slc7a11	chr3:50364935-50443613	3.87469	53.9042	3.79824	0.000195236	yes
Oas1b	chr5:120812634-120824160	0.511846	7.08559	3.79111	0.000195236	yes
Sp100	chr1:85650049-85709448	3.66973	48.7798	3.73254	0.000195236	yes
Igf2bp2	chr16:22059008-22163299	2.12524	27.8199	3.71042	0.000195236	yes
Gimap9	chr6:48676134-48678704	0.0411211	0.523224	3.66948	0.0324963	yes
Egr1	chr18:34861206-34864956	0.949712	12.0348	3.66358	0.000195236	yes
Scin	chr12:40059770-40134228	0.0425101	0.537318	3.6599	0.00116073	yes
Slfn2	chr11:83065111-83070678	11.2148	141.379	3.65609	0.000195236	yes
Bcl2a1d	chr9:88723287-88731850	15.6467	193.715	3.63001	0.000195236	yes
Csf2	chr11:54247269-54249899	0.481885	5.86722	3.60592	0.000195236	yes
Tarm1	chr7:3489075-3502552	0.109533	1.3232	3.5946	0.00160026	yes
Herc6	chr6:57580991-57665136	2.56536	30.7213	3.58201	0.000195236	yes
Helz2	chr2:181227614-181242027	1.70258	20.2515	3.57224	0.000195236	yes
F3	chr3:121723536-121735052	0.12393	1.46866	3.5669	0.000195236	yes
Cxcl11	chr5:92331840-92414627	0.121622	1.41953	3.54493	0.0136535	yes
Gm5483	chr16:36184211-36188110	0.45845	5.01499	3.45141	0.00298992	yes
Edn1	chr13:42301269-42307989	0.0676133	0.725275	3.42315	0.000370691	yes
Agpat9	chr5:100846228-100899102	0.658492	7.02984	3.41625	0.000195236	yes
Lilrb4a	chr10:51490897-51496611	51.6533	545.975	3.4019	0.000195236	yes
Socs2	chr10:95411489-95416857	0.0895473	0.934405	3.38333	0.000195236	yes
Plaur	chr7:24462499-24475873	24.6947	256.92	3.37905	0.000195236	yes
Clec2d	chr6:129180614-129186535	0.670339	6.85019	3.35318	0.000195236	yes
Samd9l	chr6:3372257-3399571	9.21388	93.4246	3.34192	0.000195236	yes
Dgkh	chr14:78569608-78725089	0.747222	7.34138	3.29644	0.000195236	yes
Myc	chr15:61985340-61990361	1.30651	12.7676	3.2887	0.000195236	yes
Tnfrsf1b	chr4:145212367-145246870	4.55141	44.4567	3.28802	0.000195236	yes
Ms4a4d	chr19:11536848-11558466	0.085819	0.831897	3.27703	0.00174394	yes
Basp1	chr15:25363276-25413764	2.17016	20.9287	3.2696	0.000195236	yes
Pla1a	chr16:38396116-38433145	0.315084	3.0152	3.25845	0.000195236	yes
Ccl9	chr11:83572916-83578636	202.44	1934.73	3.25657	0.000195236	yes
Pou2f2	chr7:25091114-25132460	0.896328	8.55438	3.25457	0.000195236	yes
Plek	chr11:16971205-17008718	88.7532	840.895	3.24406	0.000195236	yes
Stat1	chr1:52119437-52161865	20.7867	195.787	3.23555	0.000195236	yes
Fcgr4	chr1:171018925-171029761	1.54727	14.305	3.20872	0.000195236	yes
Steap1	chr5:5736321-5749317	0.0552657	0.509029	3.20329	0.0371494	yes
Creb5	chr6:53573373-53695832	0.181374	1.67033	3.20309	0.000195236	yes
Ptges	chr2:30889470-30903297	0.127283	1.15331	3.17967	0.000195236	yes
Traf1	chr2:34943257-34961772	3.01463	27.1768	3.17232	0.000195236	yes
Pim1	chr17:29491044-29495459	3.12526	27.9659	3.16162	0.000195236	yes
C3ar1	chr6:122847139-122856157	7.70799	68.8503	3.15904	0.000195236	yes
Epsti1	chr14:77904238-78002656	6.06206	53.1633	3.13255	0.000195236	yes
Ms4a4c	chr19:11407660-11427246	0.0955955	0.837172	3.13051	0.000537301	yes
Nod2	chr8:88647346-88688474	0.466807	4.06571	3.12261	0.000195236	yes
Marcks	chr10:37133242-37138926	1.38255	11.9688	3.11388	0.000195236	yes
Pim2	chrX:7878305-7883432	1.46819	12.6782	3.11024	0.000195236	yes
Oas1g	chr5:120876141-120887613	1.73128	14.6613	3.0821	0.000195236	yes
Mlkl	chr8:111311799-111337903	12.3272	104.377	3.08189	0.000195236	yes
Ikbke	chr1:131254601-131279563	4.50834	37.7773	3.06685	0.000195236	yes
Plekhn1	chr4:156221455-156228542	0.80328	6.62896	3.04481	0.000195236	yes
Irak3	chr10:120141653-120201537	5.41094	44.5857	3.04263	0.000195236	yes
Cpd	chr11:76777207-76847008	26.9048	220.498	3.03483	0.000195236	yes
Lgals3bp	chr11:118392751-118401931	6.77543	54.5084	3.00809	0.000195236	yes
Zc3h12a	chr4:125118413-125127881	1.60074	12.7862	2.99778	0.000195236	yes
Slc31a2	chr4:62286428-62298412	3.29823	26.148	2.98693	0.000195236	yes
Stap1	chr5:86071827-86103993	39.4316	311.388	2.98129	0.000195236	yes
Gadd45a	chr6:67035095-67080652	0.690824	5.44852	2.97947	0.000195236	yes
Perm1	chr4:156215926-156221307	0.114598	0.90055	2.97422	0.000195236	yes

Optn	chr2:5020641-5063938	0.0681122	0.535195	2.97408	0.000698636	yes
Abhd3	chr18:10644410-10706696	0.309552	2.42935	2.97232	0.000195236	yes
Oas1a	chr5:120896256-120907525	6.35008	49.6444	2.96678	0.000195236	yes
Phf11b	chr14:59320963-59341330	2.0424	15.9439	2.96467	0.000195236	yes
BC021614	chr19:4057486-4059294	1.12655	8.79268	2.96439	0.000195236	yes
Klra2	chr6:131219234-131247362	0.635685	4.92332	2.95325	0.000195236	yes
Serpinb9b	chr13:33027413-33040558	0.142071	1.09984	2.95262	0.000195236	yes
S100a8	chr3:90669070-90670034	0.811002	6.22091	2.93935	0.000195236	yes
Bcl2a1b	chr9:89199272-89207838	27.3027	207.52	2.92613	0.000195236	yes
Slfn10-ps	chr11:83028125-83040533	3.4769	26.2548	2.91671	0.000195236	yes
Stk40	chr4:126103956-126141029	3.12351	22.8939	2.87372	0.000195236	yes
1300002E11Rik	chr16:21794346-21809039	6.24276	45.4607	2.86436	0.000195236	yes
Map1b	chr13:99421463-99516602	0.165687	1.20535	2.86291	0.000195236	yes
Fam177a	chr12:55124527-55142082	0.0762503	0.553671	2.86021	0.000195236	yes
Casp4	chr9:5308848-5336791	2.38214	17.1828	2.85064	0.000195236	yes
Gm8369	chr19:11492037-11512577	0.0625051	0.446818	2.83764	0.026831	yes
Cdc42ep2	chr19:5917555-5924816	0.33962	2.4269	2.83712	0.000195236	yes
Cpm	chr10:117629499-117687352	0.315459	2.20734	2.80678	0.000195236	yes
Car13	chr3:14641726-14663002	5.27599	36.6403	2.79592	0.000195236	yes
Cd80	chr16:38458932-38486932	1.35668	9.38284	2.78994	0.000195236	yes
Gadd45b	chr10:80930090-80932204	4.9757	34.1798	2.78017	0.000195236	yes
Bcl2l11	chr2:128126037-128162547	4.25078	29.054	2.77294	0.000195236	yes
Zfp36	chr7:28376783-28379228	12.0552	82.3864	2.77274	0.000195236	yes
Fam19a2	chr10:123264075-123741204	0.400664	2.73699	2.77212	0.000195236	yes
Thbs1	chr2:118111921-118127133	0.209711	1.42818	2.7677	0.000195236	yes
Itgax	chr7:128129567-128150657	0.426722	2.89361	2.7615	0.000195236	yes
Hsh2d	chr8:72189667-72200958	0.263664	1.78615	2.76008	0.000195236	yes
Trafd1	chr5:121371724-121385615	5.70089	38.4284	2.75291	0.000195236	yes
Ier3	chr17:35821712-35822911	24.1148	161.544	2.74393	0.000195236	yes
Cd82	chr2:93419101-93469874	9.76085	65.1713	2.73916	0.000195236	yes
Aox2	chr1:58278325-58379264	0.0716734	0.473792	2.72474	0.000195236	yes
Dgka	chr10:128706257-128744056	0.487993	3.22151	2.72281	0.000195236	yes
Hbegf	chr18:36504926-36515805	0.170848	1.12358	2.71732	0.000195236	yes
Ifit1bl1	chr19:34592887-34601968	0.124961	0.820301	2.71468	0.000195236	yes
Ehd1	chr19:6276895-6300096	28.8042	187.686	2.70397	0.000195236	yes
Irf9	chr14:55603984-55610030	4.85418	31.469	2.69663	0.000195236	yes
Rffl	chr11:82781108-82871210	2.41609	15.6475	2.69519	0.000537301	yes
Tlr3	chr8:45395664-45410539	1.09877	7.07643	2.68713	0.000195236	yes
Parp9	chr16:35926514-35972621	9.96039	64.0132	2.6841	0.000195236	yes
Olfr549	chr7:102554285-102555236	0.122514	0.785908	2.68142	0.0476469	yes
Errfi1	chr4:150855090-150868880	4.72265	30.0828	2.67127	0.000195236	yes
Batf2	chr19:6164457-6172475	0.429138	2.72852	2.6686	0.000195236	yes
Cfb	chr17:34856373-34862514	0.15889	0.997831	2.65077	0.000195236	yes
Tnf	chr17:35199366-35202007	49.1864	308.651	2.64965	0.000195236	yes
Uba7	chr9:107975566-107984056	2.89535	18.0788	2.64249	0.000195236	yes
Gimap6	chr6:48701582-48708244	0.387581	2.4047	2.63328	0.000195236	yes
Parp12	chr6:39086411-39118349	20.6404	127.486	2.62679	0.000195236	yes
Tmem171	chr13:98686237-98694831	4.30664	26.5919	2.62635	0.000195236	yes
Tor3a	chr1:156653616-156674339	25.5505	157.145	2.62067	0.000195236	yes
Cflar	chr1:58711490-58759209	10.2938	62.782	2.60858	0.000195236	yes
Tap1	chr17:34187555-34197225	4.64918	27.6621	2.57286	0.000195236	yes
Ebi3	chr17:55952622-55967951	5.63726	33.5253	2.57218	0.000195236	yes
Slamf9	chr1:172475359-172478409	0.447008	2.59437	2.53701	0.000195236	yes
BC049352	chr9:45172725-45249985	0.114549	0.662833	2.53268	0.00116073	yes
Bcl3	chr7:19808461-19822755	2.21765	12.7746	2.52618	0.000195236	yes
Tmem202	chr9:59518684-59525501	0.267362	1.51791	2.50522	0.000195236	yes
Pde4b	chr4:102254741-102607262	2.91406	16.4401	2.49612	0.000195236	yes
Smim3	chr18:60474190-60501983	3.64094	20.4693	2.49107	0.000195236	yes
Galnt3	chr2:66082765-66124793	2.02783	11.3948	2.49037	0.000195236	yes
Ppbp	chr5:90768517-90770060	0.435588	2.41069	2.46841	0.000195236	yes
Trim12c	chr7:104338753-104353358	4.0618	22.2743	2.45519	0.000195236	yes
Itga5	chr15:103344285-103366748	7.10871	38.8965	2.45198	0.000195236	yes
Tnip1	chr11:54910786-54962940	17.019	93.04	2.4507	0.000195236	yes
Slc6a4	chr11:76998596-77032343	0.105672	0.577033	2.44906	0.000370691	yes
Nckap1	chr2:80500511-80581182	0.614773	3.35136	2.44662	0.000195236	yes

Fosl1	chr19:5447697-5457563	2.57886	14.0295	2.44366	0.0359964	yes
Slc11a1	chr1:74375202-74386051	27.7992	151.005	2.44148	0.000195236	yes
Cd83	chr13:43785111-43803133	6.04195	32.5012	2.42741	0.000195236	yes
Marcksl1	chr4:129513580-129515981	32.2652	172.876	2.42169	0.000195236	yes
Gm16712	chr17:55952622-55967951	1.38474	7.41099	2.42005	0.00467182	yes
Osm	chr11:4236784-4241026	1.19901	6.41294	2.41914	0.000195236	yes
Pml	chr9:58217179-58249786	2.64726	14.1051	2.41365	0.000195236	yes
Mndal	chr1:173857219-173880187	14.372	76.2184	2.40688	0.000195236	yes
Trem1	chr17:48232738-48246924	0.883763	4.66403	2.39985	0.000195236	yes
Ube2l6	chr2:84798827-84810003	21.4767	113.307	2.39939	0.000195236	yes
Trim21	chr7:102557919-102565482	3.84639	20.2068	2.39326	0.000195236	yes
Batf	chr12:85686719-85709087	0.132282	0.690672	2.38438	0.00778355	yes
Serpina3f,Serpina3g	chr12:104214543-104241934	0.135556	0.707504	2.38384	0.000195236	yes
Ms4a6d	chr19:11586605-11604804	21.2178	110.313	2.37826	0.000195236	yes
Maff	chr15:79346620-79359076	1.14252	5.89025	2.36611	0.000195236	yes
Dtx3l	chr16:35926514-35972621	6.95068	35.3969	2.3484	0.000195236	yes
Ticam1	chr17:56269461-56276767	1.8946	9.60006	2.34115	0.000195236	yes
Clec4a1	chr6:122921847-122934619	0.422787	2.12139	2.32701	0.000195236	yes
Sdc4	chr2:164424246-164443188	36.6339	183.383	2.32361	0.000195236	yes
Prrg4	chr2:104830740-104849850	1.96404	9.81127	2.32062	0.000195236	yes
Ptprj	chr2:90429755-90580647	15.4192	76.8164	2.31669	0.000195236	yes
Tlcd2	chr11:75468049-75470899	0.364326	1.80159	2.30597	0.000195236	yes
2010005H15Rik	chr16:36221561-36257427	0.221733	1.09415	2.30291	0.0350894	yes
St3gal6	chr16:58470540-58523312	10.2333	50.2279	2.29521	0.000195236	yes
Adora2b	chr11:62248983-62266452	4.1463	20.2695	2.28941	0.000195236	yes
Fam46a	chr9:85320438-85327150	2.77846	13.4651	2.27687	0.000195236	yes
Ell2	chr13:75707483-75772358	34.9454	169.178	2.27536	0.000195236	yes
Gm5431	chr11:48887421-48902152	0.254863	1.23061	2.27158	0.000195236	yes
Rel	chr11:23741728-23770970	7.54076	36.269	2.26595	0.000195236	yes
Enpp4	chr17:44096309-44105808	3.49558	16.6965	2.25594	0.000195236	yes
H2-T23	chr17:35994503-36038174	3.23924	15.3932	2.24857	0.000195236	yes
Aqp9	chr9:71110658-71163289	0.263426	1.2487	2.24495	0.000195236	yes
Ifi35	chr11:101448411-101458701	13.8426	65.5284	2.243	0.000195236	yes
Plet1os	chr9:50488798-50505639	0.218332	1.03153	2.24019	0.00915655	yes
Tet2	chr3:133463676-133544390	6.50296	30.5447	2.23175	0.000195236	yes
Fndc3a	chr14:72537952-72710003	10.7216	50.2461	2.22849	0.000195236	yes
Parp10	chr15:76232994-76243440	3.92057	18.3402	2.22588	0.000195236	yes
Glrx	chr13:75839885-75850151	29.4366	137.445	2.22317	0.000195236	yes
Flrt2	chr12:95692225-95785213	8.94153	41.7067	2.22169	0.000195236	yes
Il20rb	chr9:100457718-100486473	0.104948	0.489186	2.22071	0.000370691	yes
Micall2	chr5:139706692-139736333	1.42109	6.60593	2.21676	0.000195236	yes
Nfkbia	chr12:55280813-55492647	65.0842	301.875	2.21357	0.000195236	yes
Atp6v0a1	chr11:101009451-101063717	5.10511	23.2754	2.18879	0.000195236	yes
C3	chr17:57203966-57228136	1.33814	6.09458	2.1873	0.000195236	yes
Icosl	chr10:78069367-78079525	1.12943	5.14272	2.18694	0.000195236	yes
Abtb2	chr2:103566309-103718423	0.81855	3.71524	2.18231	0.000195236	yes
Plk2	chr13:110395043-110400843	0.882576	3.97498	2.17116	0.000195236	yes
4930415F15Rik	chr11:11489265-11515190	0.130446	0.581656	2.15672	0.0364231	yes
St3gal1	chr15:67102874-67176882	1.71817	7.62524	2.14991	0.000195236	yes
Rhbdf2	chr11:116598165-116627019	4.9447	21.6735	2.13198	0.000195236	yes
Zc3h12c	chr9:52111984-52168111	9.54664	41.6939	2.12677	0.000195236	yes
Osgin2	chr4:15997120-16013877	13.8361	60.3289	2.12441	0.000195236	yes
Prokr1	chr6:87578591-87590701	0.187671	0.812685	2.11449	0.000195236	yes
Mr1	chr1:155127877-155146780	0.318897	1.37532	2.1086	0.000195236	yes
Sp140	chr1:85576898-85645036	3.30457	14.1467	2.09793	0.000195236	yes
Lgals8	chr13:12439401-12461738	18.9655	80.9043	2.09284	0.000195236	yes
Dusp16	chr6:134715467-134792628	3.48338	14.7643	2.08356	0.000195236	yes
Trib1	chr15:59648653-59657099	2.64915	11.1961	2.07939	0.000195236	yes
Tubb2b	chr13:34127007-34130354	0.50362	2.11367	2.06934	0.000195236	yes
Tnfrsf23	chr7:143665806-143685875	2.93754	12.3172	2.06799	0.000195236	yes
Arhgef3	chr14:27143992-27403911	1.4294	5.91453	2.04886	0.000195236	yes
Eif2ak2	chr17:78850504-78882572	43.2651	178.097	2.04139	0.000195236	yes
Ptpn5	chr7:47077799-47133684	0.149003	0.612563	2.03952	0.000195236	yes
Cpeb4	chr11:31870939-31935635	5.83796	23.9404	2.03591	0.000195236	yes
Itgal	chr7:127296259-127335137	1.37798	5.63318	2.03139	0.000195236	yes

Ifitm3	chr7:141009589-141010744	276.27	1127.35	2.02878	0.000195236	yes
Nlrc5	chr8:94472762-94527272	1.96915	8.02193	2.02638	0.000195236	yes
Rassf4	chr6:116633007-116673836	20.2781	81.6779	2.01002	0.000195236	yes
Il23a	chr10:128296139-128298084	0.183931	0.733869	1.99635	0.00188078	yes
S100a11	chr3:93520495-93526288	107.635	429.35	1.996	0.000195236	yes
Arid3b	chr9:57790504-57834234	0.273436	1.0856	1.98922	0.000195236	yes
Nlrp3	chr11:59542685-59566956	12.0233	47.6907	1.98788	0.000195236	yes
Lck	chr4:129548343-129573641	0.752466	2.97245	1.98195	0.000195236	yes
Cd44	chr2:102811141-102901665	56.962	224.569	1.97909	0.000195236	yes
Atrip,Trex1	chr9:109057931-109074124	24.3604	95.6607	1.97339	0.000195236	yes
Pvr	chr7:19903577-19921143	3.91428	15.3446	1.97091	0.000195236	yes
Kctd12	chr14:102976580-102982637	6.29521	24.6668	1.97024	0.000195236	yes
Gch1	chr14:47153894-47189402	5.94483	23.2203	1.96568	0.000195236	yes
Dusp1	chr17:26505590-26508472	3.39071	13.1887	1.95965	0.000195236	yes
Diap2	chrX:129749741-130465833	3.51146	13.6476	1.95851	0.000195236	yes
Clec4n	chr6:123229842-123247024	0.859013	3.33163	1.95548	0.000195236	yes
5730508B09Rik	chr3:127869687-127896323	14.4435	55.8723	1.95172	0.000195236	yes
Zyx	chr6:42349827-42380558	3.98787	15.3736	1.94676	0.000195236	yes
Tlr6	chr5:64953094-64960034	3.34294	12.8027	1.93725	0.000195236	yes
Dnajb9	chr12:44205896-44210068	9.53915	36.5101	1.93636	0.000195236	yes
Trp53inp2	chr2:155381855-155389847	0.576332	2.20068	1.93298	0.000195236	yes
Prkx	chrX:77762029-77795960	8.848	33.7108	1.92979	0.000195236	yes
Cdk6	chr5:3344311-3522225	20.8887	79.4305	1.92697	0.000195236	yes
Mxd1	chr6:86647044-86669159	1.10458	4.17262	1.91745	0.000195236	yes
Ppm1k	chr6:57506501-57535426	2.52245	9.49952	1.91303	0.000195236	yes
Spred3	chr7:29158828-29168647	0.227293	0.853744	1.90925	0.000195236	yes
I830077J02Rik	chr3:105925890-105932664	0.671681	2.5017	1.89706	0.000195236	yes
Rnf19b	chr4:129058270-129084526	16.8468	62.705	1.89611	0.000195236	yes
Wfdc17	chr11:83704055-83706269	6.86889	25.56	1.89574	0.000195236	yes
Il4ra	chr7:125552281-125579474	1.2954	4.81236	1.89335	0.000195236	yes
Tapbp	chr17:33919477-33929290	20.0139	74.2854	1.89208	0.000195236	yes
Mfap3l	chr8:60632824-60676731	0.364791	1.35169	1.88962	0.000195236	yes
Bcl2l1	chr2:152754172-152831728	12.8668	47.6316	1.88826	0.000195236	yes
Syk	chr13:52583436-52648792	11.1965	41.4064	1.88681	0.000195236	yes
Stat3	chr11:100886809-100939511	8.29692	30.6289	1.88425	0.000195236	yes
Plagl2	chr2:153227768-153241358	3.22868	11.8926	1.88105	0.000195236	yes
Insl3,Jak3	chr8:71676382-71690577	1.47398	5.3998	1.87319	0.000370691	yes
Rab11fip1	chr8:27138772-27174646	3.20708	11.7486	1.87315	0.000195236	yes
Polm	chr11:5827859-5838016	0.842131	3.0527	1.85797	0.000195236	yes
Bcorl1	chrX:48341357-48406728	0.355517	1.28828	1.85746	0.000195236	yes
Tmem106a	chr11:101582241-101591785	9.51602	34.31	1.8502	0.000195236	yes
4933432I03Rik	chr14:102987411-103033803	0.548484	1.9736	1.84731	0.000195236	yes
Fyb	chr15:6579846-6665608	18.4006	65.7824	1.83795	0.000195236	yes
Serpini1	chr3:75557532-75642523	0.249434	0.889151	1.83377	0.000195236	yes
Gas7	chr11:67532997-67688992	14.8842	52.8141	1.82714	0.000195236	yes
1700030J22Rik	chr8:116969598-116978943	0.133312	0.469471	1.81623	0.000698636	yes
Etnk1	chr6:143167229-143208547	25.387	89.0413	1.81038	0.000195236	yes
Tubb2a-ps2	chr12:11882195-11882899	0.321779	1.12793	1.80954	0.00893132	yes
Mustn1	chr14:30879256-30881610	0.344975	1.20212	1.80102	0.000537301	yes
Ccr1	chr9:123962125-123968692	0.437878	1.52437	1.79961	0.000195236	yes
Denr	chr5:123907274-123928832	71.534	248.915	1.79896	0.000195236	yes
Rras	chr7:45018006-45021644	2.40456	8.30447	1.78812	0.000195236	yes
Colgalt2	chr1:152399866-152510695	0.180806	0.622699	1.78409	0.000195236	yes
Sass6	chr3:116562972-116630986	12.9262	44.4757	1.78272	0.000195236	yes
Crem	chr18:3266353-3366863	4.11542	14.0757	1.7741	0.000195236	yes
Flt3l	chr7:45131188-45136432	1.33604	4.56511	1.77269	0.000195236	yes
Wfs1	chr5:36966103-36988982	0.329619	1.12561	1.77183	0.000195236	yes
Asb2	chr12:103321141-103356001	0.132444	0.45082	1.76717	0.00160026	yes
Nfkb2	chr19:46304736-46327156	9.15186	31.1414	1.7667	0.000195236	yes
LOC100041034	chr1_GL456211_random:14534	1.18763	4.04023	1.76636	0.000195236	yes
Itsn1	chr16:91729370-91920579	7.20544	24.4881	1.76493	0.000195236	yes
Cd14	chr18:36725063-36726815	134.527	455.865	1.76071	0.000195236	yes
Slc11a2	chr15:100387899-100423055	11.9405	40.3813	1.75782	0.000195236	yes
Rhoc	chr3:104789033-104794459	44.2741	149.684	1.75739	0.000195236	yes
Sntb2	chr8:106935749-107014192	3.47371	11.7189	1.75429	0.000195236	yes

Hgsnat	chr8:25944458-25976744	14.9836	50.398	1.74998	0.000195236	yes
Itgb2	chr10:77530347-77565674	49.5129	166.116	1.74632	0.000195236	yes
Pld3	chr7:27532017-27553112	10.6851	35.7849	1.74375	0.000195236	yes
Ddt	chr10:75771232-75773374	21.8746	73.1272	1.74115	0.000195236	yes
Ly96	chr1:16688455-16709605	12.6817	42.3159	1.73845	0.000195236	yes
Nabp1	chr1:51469487-51478399	5.41662	18.0338	1.73524	0.000195236	yes
Jdp2	chr12:85599104-85639878	49.2376	163.721	1.73341	0.000195236	yes
Diap1	chr18:37843600-37935423	8.98459	29.8691	1.73313	0.000195236	yes
Tnfrsf22	chr7:143636724-143649638	2.38358	7.92186	1.73271	0.000195236	yes
Ms4a6c	chr19:11469367-11482196	8.2423	27.3882	1.73244	0.000195236	yes
Apbb1ip	chr2:22774326-22875653	8.71716	28.8438	1.72633	0.000195236	yes
Lrrc8d	chr5:105699968-105815215	12.5012	41.344	1.72561	0.000195236	yes
Olfr543	chr7:102476772-102477902	0.192581	0.632617	1.71587	0.0355539	yes
Lima1	chr15:99778467-99875456	5.23341	17.171	1.71415	0.000195236	yes
Tnnc1	chr14:31208311-31211711	0.347079	1.13417	1.7083	0.0103944	yes
Ktn1	chr14:47649310-47736564	17.5716	57.3846	1.70742	0.000195236	yes
Dusp2	chr2:127336158-127338377	9.38628	30.6507	1.7073	0.000195236	yes
Odc1	chr12:17544872-17551502	72.6094	236.966	1.70645	0.000195236	yes
Cd69	chr6:129267324-129275369	0.332448	1.08424	1.70549	0.000195236	yes
Fcgr2b	chr1:170960558-170976071	10.9416	35.6796	1.70527	0.000195236	yes
Kdm5b	chr1:134560177-134632878	1.4804	4.80911	1.69978	0.000195236	yes
P2rx4	chr5:122707556-122729042	4.62653	15.012	1.69811	0.000195236	yes
Ntng2	chr2:29194725-29252993	1.33866	4.34343	1.69804	0.000195236	yes
Tnfrsf10b	chr14:69767471-69784411	0.304756	0.988709	1.69789	0.000195236	yes
Akap3	chr6:126853097-126874308	0.15018	0.487051	1.69738	0.000195236	yes
Dst	chr1:33908224-34308662	8.48292	27.4613	1.69477	0.000195236	yes
Pilra	chr5:137787801-137836278	2.3317	7.49853	1.68522	0.000195236	yes
Rap1b	chr10:117814596-117845974	217.472	697.082	1.6805	0.000195236	yes
Tnfaip3	chr10:19000909-19015410	1.81067	5.80108	1.6798	0.000195236	yes
Mitd1	chr1:37878889-37890411	29.1759	93.4695	1.67972	0.000195236	yes
Znfx1	chr2:167035795-167065862	11.2766	36.0839	1.67803	0.000195236	yes
Gyk	chrX:85701936-85776819	18.0656	57.8054	1.67796	0.000195236	yes
Gca	chr2:62664326-62694109	0.14546	0.464457	1.67492	0.003245	yes
Mov10	chr3:104794833-104818563	1.89716	6.05533	1.67436	0.000195236	yes
Adar	chr3:89715021-89764632	6.34757	20.2153	1.67117	0.000195236	yes
Bst2	chr8:71534261-71537437	24.8082	78.8736	1.66872	0.000195236	yes
Qsox1	chr1:155778154-155812899	7.21489	22.9298	1.66817	0.000195236	yes
Plk3	chr4:117126812-117133952	3.3342	10.5447	1.6611	0.000195236	yes
Ms4a6b	chr19:11518558-11530403	3.7004	11.6919	1.65976	0.000195236	yes
Lrp12	chr15:39870602-39943757	4.65309	14.676	1.6572	0.000195236	yes
Ehd2	chr7:15948986-15967535	1.521	4.77613	1.65082	0.000195236	yes
Furin	chr7:80389193-80405436	6.47972	20.3009	1.64754	0.000195236	yes
Arhgef12	chr9:42963841-43105718	3.53399	11.0225	1.64109	0.000195236	yes
N4bp1	chr8:86841138-86885258	9.04522	28.211	1.64103	0.000195236	yes
Zufsp	chr10:33926935-33951212	5.76957	17.9698	1.63904	0.000195236	yes
Spty2d1	chr7:46990395-47008414	6.50736	20.2632	1.63871	0.000195236	yes
Arap1	chr7:101348068-101412586	8.92541	27.788	1.63847	0.000195236	yes
Tmem2	chr19:21778339-21858360	3.44331	10.718	1.63816	0.000195236	yes
Xcr1	chr9:123852314-123862029	0.289394	0.899293	1.63575	0.000195236	yes
Rapgef2	chr3:79062528-79145875	7.31567	22.6801	1.63237	0.000195236	yes
Tubb2a	chr13:34074279-34078008	6.47207	20.0617	1.63215	0.000195236	yes
Anxa3	chr5:96793384-96845968	7.02889	21.7628	1.63049	0.000195236	yes
Apobr	chr7:126585007-126589092	14.593	44.9896	1.62431	0.000195236	yes
Frk	chr10:34483399-34611230	1.84318	5.67817	1.62323	0.000195236	yes
Sqrdl	chr2:122765358-122809551	2.8414	8.74635	1.62208	0.000195236	yes
Cd164	chr10:41519499-41531042	42.0625	129.433	1.6216	0.000195236	yes
Fermt2	chr14:45458791-45530065	0.351795	1.08162	1.62038	0.000195236	yes
Serpine1	chr5:137061505-137072272	6.62806	20.3523	1.61853	0.000195236	yes
Hk2	chr6:82725026-82774454	16.2663	49.9142	1.61756	0.000195236	yes
Rbms1	chr2:60751952-60963204	40.7756	124.904	1.61504	0.000195236	yes
Ptgs2os2	chr1:150159042-150164948	4.23079	12.9518	1.61416	0.000195236	yes
Prkce	chr17:86167784-86657919	0.767062	2.34803	1.61403	0.000195236	yes
Hivep1	chr13:42052020-42185026	1.99672	6.11004	1.61355	0.000195236	yes
Lrp11	chr10:7589799-7625477	1.42144	4.33248	1.60784	0.000195236	yes
Hcls1	chr16:36934982-36963214	108.434	330.023	1.60575	0.000195236	yes

Treml2	chr17:48300037-48312534	0.52022	1.58135	1.60396	0.000195236	yes
Pstpip2	chr18:77794549-77882879	9.51474	28.8885	1.60226	0.000195236	yes
Cldn12	chr5:5505014-5514976	2.07114	6.27707	1.59966	0.000195236	yes
Lyn	chr4:3678120-3791612	66.6393	201.578	1.59689	0.000195236	yes
Ripk2	chr4:16123374-16163498	14.9002	45.0543	1.59633	0.000195236	yes
Bcar1	chr8:111710474-111743849	0.345226	1.04306	1.59521	0.000195236	yes
AU022793	chr15:39962648-39967515	0.673511	2.03465	1.59501	0.000195236	yes
Map3k8	chr18:4331326-4352953	3.57918	10.7995	1.59326	0.000195236	yes
Gstt3	chr10:75774121-75781414	6.41682	19.3319	1.59105	0.000195236	yes
Kdm6b	chr11:69398517-69413675	0.240562	0.724356	1.59029	0.000195236	yes
Napepld	chr5:21662902-21701345	4.44855	13.3008	1.5801	0.000195236	yes
Zswim6	chr13:107724616-107890064	2.68508	8.00098	1.57521	0.000195236	yes
Degs1	chr1:182275769-182282759	85.0464	252.506	1.57	0.000195236	yes
Peak1	chr9:56201128-56418050	9.35813	27.7746	1.56948	0.000195236	yes
Dnajb4	chr3:152183870-152210083	6.24381	18.5286	1.56926	0.000195236	yes
Itgam	chr7:128062639-128118491	13.4433	39.7972	1.56578	0.000195236	yes
Myo1d	chr11:80482126-80780025	2.02846	5.99747	1.56397	0.000195236	yes
Lacc1	chr14:77024200-77036617	6.80119	20.0863	1.56235	0.000195236	yes
Gsap	chr5:21186266-21291701	11.253	33.2133	1.56146	0.000195236	yes
Il18	chr9:50565367-50581837	11.0304	32.4725	1.55774	0.000195236	yes
Nfkb1	chr3:135584654-135691547	32.855	96.4486	1.55365	0.000195236	yes
Aff1	chr5:103754161-103855322	2.94144	8.62318	1.5517	0.000195236	yes
Cdkn1a	chr17:29090985-29100722	11.8422	34.703	1.55112	0.000195236	yes
Myo1e	chr9:70207349-70400067	19.2295	56.3118	1.55011	0.000195236	yes
Parp11	chr6:127453722-127494239	3.95182	11.5526	1.54762	0.000195236	yes
B2m	chr2:122120107-122186189	1590.63	4642.39	1.54527	0.000195236	yes
Daam1	chr12:71831067-71992376	5.55607	16.1618	1.54045	0.000195236	yes
Casp8	chr1:58795373-58847503	26.8688	77.9714	1.53701	0.000195236	yes
Fus	chr7:127967478-127982031	10.9042	31.5788	1.53407	0.000195236	yes
Nfkbie	chr17:45555699-45563168	3.2958	9.53375	1.53241	0.000195236	yes
Baz2a	chr10:128092782-128129303	2.14474	6.19044	1.52924	0.000195236	yes
Il1rap	chr16:26581704-26755502	7.61596	21.9565	1.52755	0.000195236	yes
Entpd1	chr19:40612365-40741602	0.226753	0.653422	1.52689	0.000195236	yes
Litaf	chr16:10959272-10993121	30.2076	86.8955	1.52437	0.000195236	yes
Saa3	chr7:46711997-46715676	14.5168	41.7375	1.52362	0.000195236	yes
Prr13	chr15:102459169-102462806	13.7343	39.4219	1.52121	0.000195236	yes
Gab3	chrX:74988544-75084905	0.278439	0.796828	1.51691	0.000195236	yes
Mip	chr10:128225837-128231811	0.195839	0.559715	1.51503	0.00403597	yes
Mid2	chrX:140678027-140767715	0.280658	0.800871	1.51276	0.000195236	yes
Pigl	chr11:62458459-62513900	3.3952	9.68402	1.51211	0.000195236	yes
Tor1aip1	chr1:156004598-156068859	15.58	44.3566	1.50945	0.00116073	yes
Sgtb	chr13:104109789-104141441	1.42942	4.05938	1.50583	0.000195236	yes
Zswim4	chr8:84210941-84237042	3.27158	9.28732	1.50528	0.000195236	yes
Ap1s3	chr1:79606875-79671972	7.12326	20.1886	1.50293	0.000195236	yes
Cd47	chr16:49855653-49911683	18.5648	52.5107	1.50004	0.000195236	yes
Tdrd7	chr4:45965334-46034765	8.25123	23.3256	1.49924	0.000195236	yes
Rassf8	chr6:145808382-145817584	8.94219	25.2729	1.49889	0.000195236	yes
Nr1d1	chr11:98767931-98775377	0.888575	2.50732	1.49658	0.000195236	yes
Pear1	chr3:87749096-87768953	0.183007	0.516313	1.49635	0.000195236	yes
Slc2a1	chr4:119108744-119137329	38.0261	107.131	1.49431	0.000195236	yes
Sesn3	chr9:14276300-14326134	0.240288	0.675826	1.49189	0.003245	yes
Ly6g5b	chr17:35113945-35115400	0.262488	0.737514	1.49042	0.033674	yes
Plekho2	chr9:65552576-65580087	14.3595	40.3292	1.48982	0.000195236	yes
Ms4a4b	chr19:11443557-11463548	0.171553	0.480983	1.48733	0.0227508	yes
Il2rg	chrX:101264384-101268255	16.2047	45.3984	1.48623	0.000195236	yes
Dcbld2	chr16:58408534-58469745	6.21171	17.3989	1.48593	0.000195236	yes
Per1	chr11:69098955-69109957	1.29385	3.61422	1.48201	0.000195236	yes
Tm9sf4	chr2:153161300-153210463	12.8322	35.7895	1.47977	0.000195236	yes
Sod2	chr17:13007838-13018119	42.6074	118.817	1.47956	0.000195236	yes
Lamc1	chr1:153218921-153332786	11.7686	32.8095	1.47917	0.000195236	yes
Slc4a7	chr14:14703024-14799943	28.3866	79.085	1.47819	0.000195236	yes
Spin4	chrX:95022506-95026682	0.466259	1.29878	1.47796	0.000195236	yes
Nfat5	chr8:107256163-107558595	13.5162	37.6359	1.47742	0.000195236	yes
Antxr2	chr5:97884687-98030962	28.1031	78.1361	1.47526	0.000195236	yes
Sez6l2	chr7:126950534-126970606	0.191213	0.53037	1.47182	0.000195236	yes

Hacd2	chr16:35022420-35109175	2.86307	7.93921	1.47143	0.000195236	yes
Kantr	chrX:152294823-152327493	0.525044	1.45579	1.47129	0.000195236	yes
Atp7a	chrX:106027223-106128160	7.86766	21.7806	1.46904	0.000195236	yes
Pcyt1a	chr16:32430920-32475065	4.9093	13.5897	1.46892	0.000195236	yes
Iqcg	chr16:32914099-33056186	3.89328	10.7742	1.46853	0.00492649	yes
Mir22,Mir22hg	chr11:75461538-75466690	1.7479	4.83014	1.46644	0.000195236	yes
Gdi1	chrX:74305011-74311867	8.24555	22.7512	1.46425	0.000195236	yes
Lrrc25	chr8:70616843-70620850	9.30192	25.6489	1.4633	0.000195236	yes
Por	chr5:135689144-135735326	35.5148	97.8183	1.46169	0.000195236	yes
Tlr9	chr9:106222597-106226876	1.16272	3.19782	1.45959	0.000195236	yes
Slfn9	chr11:82980302-82991830	11.9984	32.9865	1.45903	0.000195236	yes
C920009B18Rik	chr10:22158608-22374139	4.8473	13.292	1.4553	0.0390137	yes
Braf	chr6:39603236-39725463	4.93085	13.5079	1.4539	0.000195236	yes
Hapln4	chr8:70083528-70090862	0.201698	0.552532	1.45386	0.000855787	yes
Fas	chr19:34290658-34327770	2.53821	6.95277	1.45378	0.000195236	yes
Mocos	chr18:24653690-24701556	1.36365	3.73162	1.45233	0.000195236	yes
Mtf1	chr4:124802548-124850730	3.21896	8.7917	1.44955	0.000195236	yes
Myh10	chr11:68691914-68816624	0.437816	1.19572	1.44949	0.000195236	yes
Il10ra	chr9:45253838-45269146	5.56264	15.1755	1.4479	0.000195236	yes
Agrn	chr4:156165289-156197488	0.844949	2.30127	1.4455	0.000195236	yes
Tab2	chr10:7905647-7956123	30.0043	81.6075	1.44353	0.000195236	yes
Rbpms	chr8:33782643-33929863	2.82981	7.69453	1.44313	0.000195236	yes
Ctif	chr18:75431220-75697696	0.719882	1.951	1.43838	0.000195236	yes
3110057O12Rik	chr3:40894276-40936307	1.13333	3.06993	1.43764	0.000195236	yes
Sik3	chr9:46012819-46224194	5.16752	13.9805	1.43588	0.000195236	yes
Samsn1	chr16:75858793-75909266	9.42612	25.4359	1.43213	0.000195236	yes
Glcci1	chr6:8509599-8597549	0.825443	2.22221	1.42875	0.000195236	yes
Homer1	chr13:93304494-93405129	12.3321	33.1749	1.42767	0.000195236	yes
Ccdc186	chr19:56790962-56813683	9.99746	26.8509	1.42534	0.000195236	yes
Maf	chr8:115703252-115706894	0.185916	0.499283	1.42521	0.000855787	yes
Gripap1	chrX:7789992-7820567	18.1284	48.6227	1.42337	0.000195236	yes
Hap1	chr11:100347326-100356141	2.67343	7.15849	1.42096	0.000195236	yes
Tank	chr2:61578585-61654169	64.628	173.046	1.42092	0.000195236	yes
Etv4	chr11:101769741-101785310	0.255485	0.683708	1.42014	0.00100977	yes
Casc4	chr2:121866969-121936207	1.35773	3.62984	1.41871	0.000195236	yes
Birc3	chr9:7848700-7873170	10.1854	27.1646	1.41523	0.000195236	yes
Parp3	chr9:106470352-106476651	2.99526	7.98179	1.41403	0.000195236	yes
Uso1	chr5:92137937-92202795	20.2088	53.8452	1.41383	0.000195236	yes
Zfand3	chr17:30005086-30210020	15.2726	40.6461	1.41217	0.000195236	yes
Msn	chrX:96096044-96168553	119.111	316.787	1.41121	0.000195236	yes
Fzd5	chr1:64730557-64737750	1.06806	2.84002	1.41091	0.000195236	yes
Adam8	chr7:139978931-139992562	37.4673	99.5996	1.41051	0.000195236	yes
B3gnt3	chr8:71691719-71701800	2.03243	5.37999	1.4044	0.000195236	yes
Klf8	chrX:153238044-153396134	2.3217	6.13173	1.40112	0.000195236	yes
Tha1	chr11:117867948-117873526	1.36969	3.6139	1.39971	0.000195236	yes
Btg2	chr1:134074864-134079155	7.03203	18.552	1.39956	0.000195236	yes
Ccdc122	chr14:77036771-77112204	2.78515	7.34012	1.39805	0.000195236	yes
Ahr	chr12:35497978-35534989	0.274565	0.72252	1.39589	0.000195236	yes
Sult6b1	chr17:78883937-78906992	0.460692	1.20989	1.393	0.000195236	yes
Phf11c	chr14:59380832-59393512	1.40908	3.70032	1.39289	0.000195236	yes
5033406O09Rik	chr12:111941990-111944482	0.974215	2.5578	1.39259	0.000195236	yes
Sap130	chr18:31634382-31723061	5.4195	14.2255	1.39225	0.000195236	yes
Actg1	chr11:120345689-120348484	40.6119	106.563	1.39173	0.000195236	yes
Carhsp1	chr16:8658586-8672153	6.44954	16.9157	1.3911	0.000195236	yes
Alg2	chr4:47469832-47474367	6.61723	17.3426	1.39002	0.000195236	yes
Lppr2	chr9:21937009-21948907	0.178532	0.467362	1.38836	0.00710851	yes
2810474O19Rik	chr6:149309413-149335663	19.4315	50.8655	1.38829	0.000195236	yes
Dnajb2	chr1:75236422-75245692	4.61913	12.0907	1.38821	0.000195236	yes
Dbt	chr3:116513078-116549981	11.7795	30.7423	1.38394	0.000195236	yes
Il13ra1	chrX:36112107-36171261	26.2232	68.3628	1.38237	0.000195236	yes
Lyrm5	chr6:145211133-145216542	18.6668	48.6203	1.38109	0.000195236	yes
Slc35g2	chr9:100552187-100571085	0.257989	0.671854	1.38084	0.011588	yes
Nmi	chr2:51948498-51973208	15.6996	40.8351	1.37908	0.000195236	yes
Rnf114	chr2:167481135-167516166	19.4477	50.5171	1.37717	0.000195236	yes
Sec24a	chr11:51692262-51756834	4.84632	12.5801	1.37619	0.000195236	yes

Siglece	chr7:43651069-43660161	1.55217	4.01622	1.37155	0.000195236	yes
Abca1	chr4:53030788-53159895	1.30125	3.36686	1.37151	0.000195236	yes
Aplp1	chr7:30434979-30445582	0.277454	0.715585	1.36687	0.00145427	yes
Sesn2	chr4:132492806-132510456	1.64526	4.24307	1.36679	0.000195236	yes
Specc1	chr11:61956762-62223013	12.8743	33.0993	1.36231	0.000195236	yes
Ppp1r13b	chr12:111828457-111908055	2.47132	6.33361	1.35775	0.000195236	yes
Eif5b	chr1:37998009-38129662	20.4436	52.3541	1.35666	0.000195236	yes
Amn1	chr6:149157576-149188712	3.63899	9.31348	1.35578	0.000195236	yes
Casp1	chr9:5298516-5307281	31.7547	81.0368	1.35161	0.000195236	yes
Dnmt3l	chr10:78030021-78063622	2.30975	5.87725	1.34741	0.000195236	yes
Jak2	chr19:29251802-29313080	18.7235	47.6038	1.34623	0.000195236	yes
Gramd1b	chr9:40297906-40455764	6.67807	16.9721	1.34566	0.000195236	yes
C5ar1	chr7:16246742-16259540	3.56284	9.0516	1.34514	0.000195236	yes
Id1	chr2:152736250-152737410	44.3568	112.459	1.34217	0.000195236	yes
Rnd1	chr15:98669204-98677461	1.41158	3.57842	1.34201	0.000195236	yes
Csrnp1	chr9:119971165-119984658	1.97996	5.01916	1.34197	0.000195236	yes
Naalad2	chr9:18323020-18385928	0.185849	0.470884	1.34124	0.00454624	yes
Acadsb	chr7:131410600-131446211	17.4566	44.1767	1.33951	0.000195236	yes
Dclre1c	chr2:3424130-3474986	4.69204	11.8705	1.33909	0.00578321	yes
Lyrm1	chr7:119895860-119916756	6.4816	16.3531	1.33514	0.000195236	yes
Creb3l4	chr3:90237497-90243512	0.258563	0.652326	1.33508	0.00578321	yes
Zmat1	chrX:134971372-135009209	0.543943	1.37145	1.33417	0.000195236	yes
Lims2	chr18:31931506-31958619	1.06966	2.69513	1.33321	0.000195236	yes
Mbd5	chr2:48949507-49317069	1.63508	4.11653	1.33207	0.000195236	yes
Gm6623	chr17:36178776-36181364	0.911209	2.29127	1.33029	0.000195236	yes
Eif4g3	chr4:137993455-138207079	10.8767	27.3316	1.32932	0.000195236	yes
4833419F23Rik	chr18:4353546-4368945	1.00113	2.51136	1.32684	0.000370691	yes
Arl5b	chr2:15055361-15079191	7.15097	17.9311	1.32625	0.000195236	yes
Lcat	chr8:105939550-105943402	0.242102	0.606967	1.326	0.0163026	yes
Lnpep	chr17:17527722-17624489	34.5034	86.5009	1.32598	0.000195236	yes
Psmd11	chr11:80428614-80472133	31.6445	79.2321	1.32413	0.000195236	yes
Evi2a,Evi2b,Gm21975	chr11:79339891-79581609	72.9306	182.568	1.32384	0.000195236	yes
Slpi	chr2:164354069-164356507	4.70437	11.7731	1.32342	0.000195236	yes
Rad54l2	chr9:106688079-106789213	4.25671	10.6411	1.32183	0.000195236	yes
Card6	chr15:5097438-5108533	0.362186	0.905334	1.32172	0.000195236	yes
Prmt2	chr10:76207225-76237865	4.65089	11.604	1.31905	0.000195236	yes
Daxx	chr17:33909444-33915590	7.27998	18.0984	1.31386	0.000195236	yes
Sidt2	chr9:45937856-45955249	4.59372	11.4105	1.31263	0.000195236	yes
Yes1	chr5:32611170-32687066	0.718313	1.7827	1.31138	0.000195236	yes
Ccnd2	chr6:127125708-127212419	11.3774	28.219	1.31049	0.000195236	yes
Fam161a	chr11:23013386-23023741	1.00143	2.47588	1.30588	0.000195236	yes
Glipr2	chr4:43957701-43979118	1.26313	3.12252	1.30571	0.000195236	yes
Tgfb1	chr7:25687001-25704996	19.3526	47.7634	1.30338	0.000195236	yes
Mob3c	chr4:115828091-115836183	2.01166	4.94735	1.29827	0.000195236	yes
Dusp10	chr1:184034460-184075636	1.14244	2.80934	1.29811	0.000195236	yes
Pla2g5	chr4:138799246-138863469	5.91594	14.5398	1.29732	0.000195236	yes
Fgd6	chr10:94036000-94145339	4.7508	11.6582	1.2951	0.000195236	yes
Cep83	chr10:94688789-94790336	57.245	140.327	1.29357	0.000195236	yes
Tuba1a	chr15:98949846-98953501	24.7636	60.568	1.29033	0.000195236	yes
Pnpla2	chr7:141455187-141460743	4.94776	12.0894	1.2889	0.000195236	yes
Aak1	chr6:86849516-87003227	1.80316	4.40468	1.28851	0.000195236	yes
Spry2	chr14:105891946-105896819	0.583036	1.42339	1.28767	0.000698636	yes
Atp6v1d	chr12:78842981-78861638	66.0201	161.019	1.28626	0.000195236	yes
Ywhag	chr5:135908378-135934641	108.318	263.989	1.28521	0.000195236	yes
Dock10	chr1:80501067-80758553	41.5844	101.327	1.2849	0.000195236	yes
Myo6	chr9:80165033-80311729	1.0357	2.52346	1.28479	0.000195236	yes
Tmem184b	chr15:79360683-79403303	5.52957	13.4686	1.28436	0.000195236	yes
Cd52	chr4:134093537-134095073	153.286	372.978	1.28286	0.000195236	yes
Fam219a	chr4:41517436-41569527	0.485131	1.18015	1.28252	0.000698636	yes
Dcun1d3	chr7:119853162-119895745	3.08366	7.4965	1.28157	0.000195236	yes
Il17ra	chr6:120463196-120483727	11.4339	27.7956	1.28154	0.000195236	yes
Coq10b	chr1:55052769-55072702	16.6867	40.5564	1.28123	0.000195236	yes
Trim25	chr11:88999402-89020293	12.7913	31.0816	1.28089	0.000195236	yes
Ppp1r15a	chr7:45473562-45526268	3.67231	8.91097	1.27889	0.0445395	yes
Ptpn23	chr9:110385088-110408210	1.40278	3.4036	1.27877	0.000195236	yes

Sgcb	chr5:73632748-73647731	3.74896	9.0798	1.27617	0.000195236	yes
Mbnl2	chr14:120275668-120431698	34.8418	84.2852	1.27446	0.000195236	yes
Rac2	chr15:78559168-78572783	58.9056	142.284	1.2723	0.000195236	yes
Foxp1	chr6:98925341-99435345	13.3568	32.2003	1.2695	0.000195236	yes
Egr3	chr14:70077444-70082613	0.244673	0.588937	1.26726	0.000195236	yes
Crtc2	chr3:90254280-90264125	2.48177	5.97361	1.26723	0.000195236	yes
Trim13	chr14:61598225-61682373	5.20601	12.4917	1.26272	0.000195236	yes
Bag3	chr7:128523582-128546979	7.19336	17.2602	1.26271	0.000195236	yes
Tnfsf9	chr17:57092022-57107757	30.9466	74.2479	1.26257	0.000195236	yes
Uba6	chr5:86110729-86172743	38.1262	91.4571	1.26231	0.000195236	yes
Srp54b	chr12:55230167-55263480	6.42441	15.4065	1.26191	0.000195236	yes
Ahi1	chr10:20952546-21080429	4.87588	11.6579	1.25757	0.000195236	yes
Whsc1l1	chr8:25601600-25719667	13.291	31.7566	1.2566	0.000195236	yes
Mogat1	chr1:78511059-78538173	1.00236	2.39487	1.25655	0.000855787	yes
Usb1	chr8:95332283-95347513	9.80936	23.4219	1.25562	0.000195236	yes
Frmd6	chr12:70825513-70902234	0.751162	1.79144	1.25393	0.000195236	yes
Acsl4	chrX:142317992-142390535	53.8126	128.287	1.25336	0.000195236	yes
Rgl1	chr1:152517529-152625111	24.5336	58.4836	1.25327	0.000195236	yes
Anxa7	chr14:20455260-20480133	47.3735	112.905	1.25296	0.000195236	yes
Lcp2	chr11:34047200-34092280	24.3742	57.9887	1.25042	0.000195236	yes
Meis2	chr2:115861263-116065058	1.75182	4.1673	1.25026	0.000195236	yes
Abca5	chr11:110269368-110337716	0.380096	0.903909	1.24981	0.000195236	yes
Acot11	chr4:106733914-106799831	0.837942	1.99248	1.24965	0.000195236	yes
A230072C01Rik	chrX:20951664-20987349	1.43649	3.4136	1.24875	0.000370691	yes
Igsf6	chr7:121034444-121076835	54.2749	128.748	1.24619	0.000195236	yes
Susd6	chr12:80790531-80880833	11.2181	26.6106	1.24617	0.000195236	yes
Mapre2	chr18:23752332-23893861	8.86405	21.0176	1.24556	0.000195236	yes
Setdb2	chr14:59402010-59440877	10.223	24.2263	1.24476	0.000195236	yes
Arl8a	chr1:135146833-135156268	15.335	36.3406	1.24475	0.000195236	yes
Rgl3	chr9:21971526-21989453	0.507417	1.20161	1.24372	0.000195236	yes
Egr2	chr10:67537868-67542188	1.1591	2.74123	1.24181	0.000195236	yes
Map9	chr3:82358071-82395268	0.230569	0.545166	1.24149	0.000195236	yes
Tor1aip2	chr1:156004598-156068859	81.3144	192.257	1.24145	0.000195236	yes
Vimp	chr7:66079648-66089405	67.5744	159.645	1.24032	0.000195236	yes
Prkaa2	chr4:105029649-105109898	0.21215	0.501056	1.23989	0.000195236	yes
Map2	chr1:66175328-66442583	0.194881	0.459951	1.23888	0.000195236	yes
Vamp2	chr11:69088527-69092381	1.05603	2.49165	1.23845	0.000195236	yes
Shisa5	chr9:109038566-109057792	32.1164	75.7733	1.23838	0.000195236	yes
Pi4k2b	chr5:52741573-52769344	14.9599	35.2832	1.23788	0.000195236	yes
Kctd13	chr7:126928878-126945609	10.4314	24.5947	1.23742	0.000195236	yes
Lrp4	chr2:91457530-91513901	0.594424	1.40033	1.2362	0.000195236	yes
Adgre1	chr17:57358685-57483529	37.9242	89.2793	1.23521	0.000195236	yes
Pik3ap1	chr19:41274217-41385070	29.0936	68.477	1.23492	0.000195236	yes
Prrc2c	chr1:162671784-162740556	28.3349	66.6885	1.23486	0.000195236	yes
Fubp1	chr3:152210457-152236830	67.3181	158.438	1.23485	0.000195236	yes
Ksr1	chr11:79014800-79146354	0.304384	0.716173	1.23442	0.000195236	yes
Il15	chr8:82331623-82402586	1.05948	2.49264	1.23431	0.000855787	yes
Cnrip1	chr11:17051933-17079372	1.04425	2.45362	1.23244	0.000195236	yes
Icam1	chr9:21015959-21028796	18.7251	43.9953	1.23237	0.000195236	yes
Hivep3	chr4:119814677-120135411	0.537233	1.25919	1.22888	0.000195236	yes
Ero1l	chr14:45283086-45318572	23.9719	56.1475	1.22788	0.000195236	yes
Nt5dc3	chr10:86779004-86838389	3.83001	8.95967	1.2261	0.000195236	yes
Agpat4	chr17:12119283-12219640	6.06428	14.1735	1.22478	0.000195236	yes
Zswim5	chr4:116877401-116989105	0.287707	0.671988	1.22383	0.000195236	yes
Brd4	chr17:32196271-32284133	3.88027	9.05629	1.22276	0.000195236	yes
Impact	chr18:12972251-12992948	15.0327	35.0824	1.22265	0.000195236	yes
Lysmd3	chr13:81657805-81671890	13.9092	32.456	1.22244	0.000195236	yes
Ppic	chr18:53406340-53418007	18.6946	43.5981	1.22164	0.000195236	yes
Supt6	chr11:78206748-78245703	20.8661	48.6403	1.22099	0.000195236	yes
Dnajb5	chr4:42953093-42958732	0.483577	1.12681	1.22043	0.000195236	yes
Gpatch11	chr17:78835515-78848308	11.5527	26.9083	1.21982	0.000195236	yes
Cpeb3	chr19:37021290-37207471	0.333849	0.777508	1.21966	0.000195236	yes
Taf15	chr11:83473107-83506740	14.1643	32.9817	1.2194	0.000195236	yes
Chst11	chr10:82985496-83195891	0.376107	0.875574	1.21909	0.000195236	yes
Appbp2os	chr11:85235165-85238304	0.545131	1.26777	1.21762	0.0294593	yes

Trip12	chr1:84721188-84839304	52.2776	121.531	1.21706	0.000195236	yes
Camk2d	chr3:126596950-126846326	8.65066	20.1068	1.2168	0.000195236	yes
Brwd3	chrX:108742207-108834355	4.39394	10.1712	1.2109	0.000195236	yes
Ndel1	chr11:68821445-68853131	33.1611	76.7588	1.21084	0.000195236	yes
Stxbp3-ps	chr19:9557605-9559248	16.2099	37.4759	1.20909	0.000195236	yes
Csf2rb	chr15:78325989-78351001	5.6658	13.0817	1.2072	0.000195236	yes
Pde1b	chr15:103503033-103530056	2.93157	6.7541	1.20409	0.000195236	yes
Smap2	chr4:120968316-121017247	12.0494	27.7574	1.20392	0.000195236	yes
Trim26	chr17:36837145-36859398	11.7968	27.1628	1.20323	0.000195236	yes
L3mbtl3	chr10:26275451-26375185	1.01326	2.33287	1.2031	0.000195236	yes
Cbwd1	chr19:24919915-24961616	22.9873	52.9045	1.20255	0.000195236	yes
Ocrl	chrX:47912455-47965866	7.00736	16.1191	1.20182	0.000195236	yes
Gpkow	chrX:7697133-7710259	10.543	24.2471	1.20154	0.000195236	yes
Arhgef7	chr8:11728104-11835217	25.8627	59.4166	1.19999	0.000195236	yes
Cited2	chr10:17723227-17725674	71.5937	164.378	1.19912	0.000195236	yes
Gab2	chr7:97081750-97308951	3.51586	8.07146	1.19895	0.000195236	yes
Fam222b	chr11:78094672-78157339	2.41835	5.55029	1.19854	0.000195236	yes
H2-K2	chr17:33974658-33978791	3.24449	7.43776	1.19688	0.000195236	yes
Tfcp2l1	chr1:118627944-118685168	0.32334	0.740961	1.19635	0.000195236	yes
Gm10825	chr10:22402812-22407470	0.259809	0.594114	1.19329	0.000698636	yes
Dgat2	chr7:99153662-99182713	2.27936	5.20994	1.19264	0.000195236	yes
Als2,Mpp4	chr1:59120934-59237231	4.951	11.3156	1.19252	0.000195236	yes
Fchsd1	chr18:37955558-37969731	0.769599	1.75707	1.191	0.000195236	yes
4930440I19Rik	chr2:78051176-78194430	1.96713	4.4885	1.19014	0.000195236	yes
Prelid2	chr18:41875695-41951194	34.0077	77.4817	1.18799	0.000195236	yes
Pik3r3	chr4:116221913-116303056	1.02187	2.32801	1.18789	0.000195236	yes
Fam57a	chr11:76202055-76208257	8.66732	19.7442	1.18777	0.000195236	yes
Stk3	chr15:34875498-35155806	26.9386	61.3552	1.18751	0.000195236	yes
Rraga	chr4:86575672-86577283	32.6556	74.3561	1.18712	0.000195236	yes
Slc7a8	chr14:54722214-54781886	0.987408	2.24758	1.18666	0.000195236	yes
Ppapdc1b	chr8:25720047-25724887	10.2311	23.2655	1.18523	0.000195236	yes
Spryd7	chr14:61534304-61556886	43.2058	98.0437	1.1822	0.000195236	yes
Stap2	chr17:55997075-56005606	0.343738	0.779366	1.18099	0.00847587	yes
Gpr84	chr15:103308234-103310438	11.9743	27.1151	1.17916	0.000195236	yes
Gramd3	chr18:56432131-56503792	0.44043	0.996141	1.17744	0.000537301	yes
Ccdc109b	chr3:129914959-129970206	28.6246	64.704	1.1766	0.000195236	yes
Brd2	chr17:34112018-34122607	54.4468	122.985	1.17556	0.000195236	yes
Atxn1	chr13:45549755-45964991	2.09614	4.73282	1.17496	0.000195236	yes
Cln5	chr14:103070215-103077630	36.8424	83.113	1.17371	0.000195236	yes
Pgm1	chr5:64092949-64128158	21.6126	48.7556	1.1737	0.000195236	yes
Taok3	chr5:117120128-117275098	23.0656	51.9427	1.17118	0.000195236	yes
Csf2ra	chr19:61224401-61228418	21.5422	48.4627	1.16971	0.000195236	yes
Rrm2b	chr15:37923952-37961055	5.05164	11.3456	1.16731	0.000195236	yes
Creb3l2	chr6:37331020-37442148	1.32259	2.96987	1.16703	0.000195236	yes
Sec23ip	chr7:128744869-128784835	18.2608	40.9918	1.16659	0.000195236	yes
Traf3ip2	chr10:39612933-39732007	5.622	12.6104	1.16545	0.000195236	yes
2210018M11Rik	chr7:98590605-98656569	7.38717	16.5678	1.16529	0.000195236	yes
Kcnc3	chr7:44590885-44604751	0.200099	0.448427	1.16416	0.000537301	yes
Hmga2	chr10:120361274-120476935	14.0104	31.3371	1.16137	0.000195236	yes
2210417A02Rik	chr5:148741839-148743139	5.11349	11.425	1.15982	0.000537301	yes
Fkbp1a	chr2:151542482-151561691	88.1082	196.835	1.15964	0.000195236	yes
Traf5	chr1:191997202-192092599	2.46504	5.50148	1.15821	0.000195236	yes
Taf3	chr2:9914551-10048609	3.33014	7.42673	1.15714	0.000195236	yes
Dnajc1	chr2:18055236-18392830	9.48511	21.1475	1.15675	0.000195236	yes
Rnf31	chr14:55591789-55603671	4.82484	10.7425	1.15478	0.000195236	yes
Ppig	chr2:69723087-69754059	29.8337	66.404	1.15433	0.000195236	yes
Cdc42bpa	chr1:179961088-180196020	0.469447	1.04324	1.15204	0.000698636	yes
Stxbp3	chr3:108793179-108840502	18.5633	41.2385	1.15154	0.000195236	yes
Irak2	chr6:113638466-113695027	7.67925	17.0593	1.15152	0.000195236	yes
Ascc3	chr10:50592668-50851202	28.6448	63.4998	1.14848	0.000195236	yes
Fam210b	chr2:172345576-172355749	1.16479	2.58014	1.14738	0.000195236	yes
Cd74	chr18:60803848-60812652	91.958	203.613	1.14678	0.000195236	yes
Atf3	chr1:191170296-191183333	10.0621	22.2676	1.14601	0.000195236	yes
Stk10	chr11:32533265-32624595	14.6275	32.3593	1.1455	0.000195236	yes
Bmpr2	chr1:59764278-59878081	7.84958	17.3617	1.14522	0.000195236	yes

Cald1	chr6:34709443-34775469	0.445517	0.985175	1.1449	0.000195236	yes
Ppp1r13l	chr7:19361215-19378533	0.293156	0.648214	1.1448	0.00285786	yes
Prkd2	chr7:16842901-16870461	1.68973	3.73542	1.14448	0.000195236	yes
Pabpc1	chr15:36595657-36608973	206.592	456.683	1.14441	0.000195236	yes
Rmdn2	chr17:79614899-79682152	2.99824	6.62208	1.14317	0.000195236	yes
Smim15	chr13:108044473-108049146	60.7866	134.212	1.14269	0.000195236	yes
Vps37c	chr19:10688814-10714419	4.65907	10.2868	1.14268	0.000195236	yes
Ypel5	chr17:72836703-72851195	6.57248	14.5041	1.14195	0.000195236	yes
Bicd1	chr6:149408983-149563326	0.212309	0.46796	1.14022	0.000195236	yes
Eaf1	chr14:31495078-31509858	19.63	43.1846	1.13745	0.000195236	yes
Nfkbib	chr7:28756173-28766644	15.9502	35.0807	1.1371	0.000195236	yes
Srpr	chr9:35211202-35267805	19.159	42.1021	1.13587	0.000195236	yes
Asah2	chr19:31984650-32103140	1.2893	2.83173	1.13509	0.000195236	yes
Tmem120b	chr5:123076274-123117445	2.82835	6.21145	1.13497	0.000195236	yes
Ubap2	chr4:41194314-41275135	11.3488	24.9177	1.13463	0.000195236	yes
Whamm	chr7:81571291-81596836	4.47784	9.82965	1.13434	0.000195236	yes
Zak	chr2:72285636-72442610	23.9698	52.5971	1.13377	0.000195236	yes
Pcdh7	chr5:57718020-58132240	60.6748	133.138	1.13375	0.000195236	yes
Chd9	chr8:90828834-91054508	6.41283	14.0623	1.1328	0.000195236	yes
Mapkbp1	chr2:119972698-120027403	1.25793	2.75651	1.13179	0.000195236	yes
Asph	chr4:9269316-9669344	63.7078	139.544	1.13117	0.000195236	yes
Samd8	chr14:21750530-21798725	7.58647	16.6126	1.13077	0.000195236	yes
Zwint	chr10:72654845-72674964	70.8939	155.107	1.12953	0.000195236	yes
D330045A20Rik	chrX:139480366-139554580	11.6967	25.5456	1.12697	0.000195236	yes
Tap2	chr17:34204478-34216321	8.20367	17.9114	1.12654	0.000195236	yes
Satb2	chr1:56793980-56971334	2.4611	5.36536	1.12437	0.000195236	yes
Themis2	chr4:132782356-132796364	9.62581	20.9429	1.12148	0.000195236	yes
4932438A13Rik	chr3:36863105-37053033	5.5535	12.0809	1.12126	0.000195236	yes
Eml2	chr7:19181169-19206482	4.14934	9.02465	1.12099	0.000195236	yes
Ccdc64	chr5:115649285-115731559	0.243373	0.529206	1.12066	0.0129867	yes
Appbp2	chr11:85191309-85235120	22.8009	49.5502	1.1198	0.000195236	yes
Gsk3b	chr16:38085063-38246079	7.79819	16.9321	1.11855	0.000195236	yes
Elf4	chrX:48411048-48463132	3.91682	8.49225	1.11646	0.000195236	yes
Shox2	chr3:66971722-66981771	1.44775	3.13881	1.1164	0.000195236	yes
Pla2g6	chr15:79286227-79328371	1.53087	3.31863	1.11624	0.000195236	yes
Sh3pxd2b	chr11:32347810-32428183	7.09983	15.3903	1.11616	0.000195236	yes
Rock1	chr18:10064400-10181792	41.7126	90.3416	1.11491	0.000195236	yes
Ypel3	chr7:126776974-126780514	1.43352	3.10452	1.11481	0.000370691	yes
Sqstm1	chr11:50200151-50210820	76.9203	166.197	1.11146	0.000195236	yes
Zswim8	chr14:20707551-20723619	2.57036	5.55303	1.1113	0.000195236	yes
Hectd2	chr19:36554638-36689479	0.396963	0.857385	1.11094	0.00351242	yes
F11r	chr1:171437560-171464593	1.08626	2.34563	1.1106	0.000195236	yes
Atp2b1	chr10:98915151-99026143	50.7607	109.558	1.1099	0.000195236	yes
Cdkl2	chr5:92006073-92043042	2.12527	4.58653	1.10976	0.000195236	yes
Tmem63b	chr17:45660176-45686218	3.68153	7.94387	1.10954	0.000195236	yes
Tnfaip2	chr12:111442660-111455018	28.0087	60.3721	1.10801	0.000195236	yes
Ncoa7	chr10:30645581-30803107	9.18581	19.796	1.10773	0.000195236	yes
Nova1	chr12:46694516-46818775	0.881763	1.89937	1.10706	0.000195236	yes
Usp32	chr11:84984487-85139955	5.27688	11.3665	1.10703	0.000195236	yes
Inpp4a	chr1:37299837-37410740	1.86992	4.0269	1.1067	0.000195236	yes
Ngly1	chr14:16238658-16311926	11.9566	25.7377	1.10608	0.000195236	yes
Tmem131	chr1:36792188-36939527	9.5979	20.6337	1.10421	0.000195236	yes
Myadm	chr7:3289037-3299349	20.3837	43.8113	1.10389	0.000195236	yes
Gm6329	chr8:45160493-45165145	11.3769	24.4486	1.10364	0.000195236	yes
Notch1	chr2:26457901-26503822	0.31978	0.687142	1.10353	0.000195236	yes
Mar-11	chr15:26309071-26409573	0.332995	0.71498	1.1024	0.0261526	yes
Mafk	chr5:139791535-139802652	13.1322	28.1962	1.10239	0.000195236	yes
Scaf4	chr16:90229143-90284425	5.91077	12.688	1.10205	0.000195236	yes
Stambp	chr6:83543205-83588126	2.8935	6.21068	1.10194	0.000195236	yes
Arnt	chr3:95434389-95497239	7.54758	16.197	1.10164	0.000195236	yes
Trip4	chr9:65828925-65908794	4.71493	10.1173	1.10151	0.0355539	yes
Peli1	chr11:21091323-21150327	10.8848	23.3371	1.10031	0.000195236	yes
Med15	chr16:17651207-17722947	9.99096	21.4171	1.10007	0.000195236	yes
Pdcd6ip	chr9:113651743-113708259	35.8034	76.6573	1.09833	0.000195236	yes
Zbtb7a	chr10:81136270-81151657	6.06556	12.9832	1.09794	0.000195236	yes

Acy1	chr9:106432980-106438236	13.072	27.9512	1.09643	0.000195236	yes
Glrp1	chr1:88499870-88510066	1.83741	3.9257	1.09528	0.000195236	yes
Erich1	chr8:14027564-14090327	12.1629	25.9859	1.09524	0.000195236	yes
H2-T22,H2-T9	chr17:36038408-36042702	3.9679	8.47486	1.09481	0.000195236	yes
Igf2r	chr17:12682405-12868143	10.0383	21.4333	1.09433	0.000195236	yes
Rilpl2	chr5:124463264-124478235	16.2585	34.7098	1.09415	0.000195236	yes
Sec24b	chr3:129983184-130060907	8.54664	18.2314	1.093	0.000195236	yes
Tceb3	chr4:136003369-136021649	31.4778	67.1281	1.09258	0.000195236	yes
Ern1	chr11:106397619-106487796	2.07691	4.4284	1.09235	0.000195236	yes
Zfp9	chr6:118461949-118479273	0.486012	1.03526	1.09092	0.000195236	yes
Wipf2	chr11:98863597-98905578	1.47307	3.13697	1.09055	0.000195236	yes
Utrn	chr10:12382187-12861735	1.72231	3.66421	1.08916	0.000195236	yes
Gm1821	chr14:46084026-46084957	70.2316	149.403	1.08902	0.000195236	yes
Micall1	chr15:79108982-79141251	2.42766	5.1604	1.08791	0.000195236	yes
Nampt	chr12:32820334-32853369	73.5573	156.348	1.08782	0.000195236	yes
Maml2	chr9:13619988-13709533	0.33295	0.7071	1.08661	0.000370691	yes
Ccnj	chr19:40831278-40848570	5.58609	11.8571	1.08584	0.000195236	yes
Elk4	chr1:132007604-132025684	12.1429	25.769	1.08552	0.000195236	yes
Ccdc82	chr9:13246978-13292353	12.2198	25.9289	1.08534	0.000195236	yes
Slc39a4	chr15:76612382-76616852	0.365224	0.773905	1.08337	0.00627268	yes
Sh3yl1	chr12:30911668-30960160	0.355687	0.753304	1.08262	0.00675438	yes
Them4	chr3:94310131-94332532	4.97817	10.5192	1.07934	0.000195236	yes
Tmem62	chr2:120977061-121007842	2.52678	5.33685	1.07869	0.000195236	yes
A630033H20Rik	chrX:107148926-107173661	9.73272	20.503	1.07492	0.000195236	yes
Atxn7	chr14:14012490-14107301	4.18134	8.80366	1.07414	0.000195236	yes
Tmem154	chr3:84666191-84704575	2.58827	5.4495	1.07414	0.000195236	yes
1600012H06Rik	chr17:14943183-14961260	15.0589	31.6977	1.07376	0.0164008	yes
Dennd5b	chr6:148988068-149101680	0.542059	1.14067	1.07336	0.000195236	yes
Stx12	chr4:132854063-132884458	39.7087	83.5555	1.07328	0.000195236	yes
Nucb2	chr7:116504368-116540588	18.5886	39.0619	1.07134	0.000195236	yes
Gm4262	chr16:11008897-11015184	0.31749	0.66703	1.07104	0.0100707	yes
C130026I21Rik	chr1:85246343-85270566	5.67579	11.9193	1.0704	0.000195236	yes
Ankrd17	chr5:90227165-90366185	20.2059	42.4151	1.06981	0.000195236	yes
Pik3r5	chr11:68432124-68497846	11.7746	24.6834	1.06787	0.000195236	yes
Ier5	chr1:155096366-155099636	17.4272	36.5304	1.06776	0.000195236	yes
Kctd21	chr7:97332322-97350216	0.760569	1.59357	1.06711	0.000195236	yes
Trp53bp2	chr1:182409166-182462436	2.52759	5.2944	1.0667	0.000195236	yes
Pnp	chr14:50944302-50953412	20.7539	43.437	1.06554	0.000195236	yes
Cblb	chr16:52031548-52208046	0.890892	1.8641	1.06515	0.000195236	yes
Slc25a22	chr7:141429748-141437874	5.22033	10.917	1.06437	0.000195236	yes
Rnf44	chr13:54679398-54693960	4.46798	9.34338	1.06432	0.000195236	yes
Snap23	chr2:120567670-120600722	15.9835	33.4109	1.06374	0.000195236	yes
Zfp609	chr9:65691582-65827564	1.75827	3.67165	1.06227	0.000195236	yes
Pigb	chr9:72985503-73039699	4.93147	10.2821	1.06004	0.0107144	yes
Blnk	chr19:40928926-40994535	17.0636	35.5559	1.05917	0.000195236	yes
Tnfsf14	chr17:57189473-57194181	0.288595	0.600852	1.05796	0.0101798	yes
Atp2a2	chr5:122453512-122502225	94.0508	195.784	1.05775	0.000195236	yes
Dmxl2	chr9:54365157-54501626	6.71941	13.98	1.05695	0.000195236	yes
Slc35d1	chr4:103171717-103214884	4.14707	8.62211	1.05595	0.000195236	yes
Gmppb	chr9:108049289-108079375	16.0561	33.3651	1.05522	0.000537301	yes
Pcgf3	chr5:108461331-108503099	13.5369	28.1279	1.05511	0.000195236	yes
5430427O19Rik	chrX:85870272-85891499	11.4467	23.7836	1.05503	0.000195236	yes
Vcpip1	chr1:9718621-9771256	21.0926	43.7929	1.05396	0.000195236	yes
Cd59a	chr2:104095800-104115410	2.09172	4.34268	1.05389	0.000195236	yes
Prdm2	chr4:143107390-143212709	3.9877	8.26168	1.05088	0.000195236	yes
Mvp	chr7:126986859-127014594	17.9538	37.1789	1.0502	0.000195236	yes
AI429214	chr8:36985572-37024238	0.23978	0.4958	1.04805	0.0223699	yes
BC028528	chr3:95871521-95891930	19.4315	40.1561	1.04722	0.000195236	yes
Med13	chr11:86201359-86357525	13.225	27.2909	1.04515	0.000370691	yes
Dock4	chr12:40446052-40846488	4.68342	9.66065	1.04456	0.000195236	yes
Gnpnat1	chr14:45351185-45388796	27.342	56.3309	1.0428	0.000195236	yes
Mapk1ip1l	chr14:47298313-47323091	32.3724	66.6216	1.04122	0.000195236	yes
Tiparp	chr3:65527484-65555518	12.5567	25.8397	1.04113	0.000195236	yes
Clec16a	chr16:10545338-10744878	3.03756	6.24771	1.04041	0.000195236	yes
Rusc1	chr3:89083978-89093363	4.41155	9.06707	1.03935	0.000195236	yes

Synj1	chr16:90936096-91044379	4.29007	8.81585	1.0391	0.0302743	yes
Zdhhc21	chr4:82798737-82859661	17.5468	36.026	1.03783	0.000195236	yes
Csnk1g1	chr9:65909009-66045014	5.84202	11.9871	1.03694	0.000195236	yes
App	chr16:84954435-85173707	38.177	78.2016	1.0345	0.000195236	yes
Vcl	chr14:20929432-21033673	11.8675	24.2685	1.03207	0.000195236	yes
Mlxip	chr5:123394797-123457931	3.0446	6.22413	1.03162	0.000195236	yes
Papd7	chr13:69497958-69533864	12.888	26.3346	1.03093	0.000195236	yes
Desi1	chr15:81992522-82016140	9.94111	20.312	1.03085	0.000195236	yes
Cdkl5	chrX:160768012-160994681	0.307976	0.628197	1.0284	0.00160026	yes
Grina	chr15:76246806-76249904	8.44638	17.2271	1.02828	0.000195236	yes
AU040320	chr4:126753554-126967923	5.70406	11.6335	1.02822	0.00529192	yes
Samd4b	chr7:28399521-28436191	2.87321	5.85931	1.02807	0.000195236	yes
Stx16	chr2:174077050-174099771	21.3997	43.6382	1.028	0.000195236	yes
Pdlim5	chr3:142239584-142395696	20.3429	41.4378	1.02642	0.000195236	yes
Natd1	chr11:60902245-60913792	0.507903	1.03421	1.02591	0.000195236	yes
Smndc1	chr19:53379213-53390573	22.2669	45.3209	1.02528	0.000195236	yes
Tmtc3	chr10:100443901-100487347	12.6176	25.6719	1.02475	0.000195236	yes
3110043O21Rik	chr4:35191281-35225880	16.855	34.2903	1.02462	0.000195236	yes
Dennd1b	chr1:138963708-139176042	16.3371	33.2249	1.02412	0.000195236	yes
Efcab5	chr11:77089914-77188968	0.30913	0.628403	1.02347	0.000195236	yes
Six4	chr12:73100258-73113245	0.248732	0.505621	1.02346	0.00258324	yes
Lrif1	chr3:106684986-106736576	12.3259	25.043	1.02271	0.000195236	yes
Nckap1l	chr15:103453824-103498800	53.737	109.178	1.02269	0.000195236	yes
Gm608	chr16:44173396-44227466	3.02749	6.1488	1.02218	0.000195236	yes
Mgea5	chr19:45750258-45783291	39.4563	80.0836	1.02125	0.000195236	yes
Arf4	chr14:26638196-26657258	42.3566	85.9274	1.02053	0.000195236	yes
Ube2k	chr5:65537260-65598989	26.0817	52.8738	1.01952	0.000195236	yes
Wsb2	chr5:117357304-117378589	13.3264	26.9982	1.01858	0.000195236	yes
Ids	chrX:70343069-70365085	4.17539	8.45739	1.0183	0.000195236	yes
Atp8b4	chr2:126320972-126491553	25.1336	50.9002	1.01806	0.000195236	yes
Rdh12	chr12:79208913-79222661	0.441538	0.894198	1.01805	0.0101798	yes
Ccr2	chr9:124102182-124109140	2.10683	4.26538	1.0176	0.000195236	yes
Irf2	chr8:46739744-46847458	4.96727	10.0434	1.01572	0.000195236	yes
Gfod1	chr13:43195518-43304172	4.95594	10.0165	1.01515	0.000195236	yes
Tcn2	chr11:3917077-3932078	0.270869	0.546598	1.01289	0.0182627	yes
Atn1	chr6:124742543-124756487	0.881133	1.77713	1.01212	0.000195236	yes
Scyl2	chr10:89640106-89686285	17.2672	34.8202	1.01189	0.000195236	yes
Herpud1	chr8:94386499-94395358	30.9303	62.3713	1.01186	0.000195236	yes
Herpud2	chr9:25108129-25151781	9.33708	18.8266	1.01173	0.000195236	yes
Tnfrsf26	chr7:143607684-143627845	7.04137	14.1969	1.01165	0.000195236	yes
Rab39	chr9:53684109-53706232	0.641896	1.2925	1.00975	0.000855787	yes
Tm2d1	chr4:98355369-98383265	5.04685	10.1613	1.00963	0.000195236	yes
Rnf11	chr4:109452856-109476505	45.7452	92.0731	1.00916	0.000195236	yes
Ttc39b	chr4:83220300-83324189	8.71485	17.5332	1.00854	0.000195236	yes
Tasp1	chr2:139833478-140066805	15.8259	31.8271	1.00797	0.000195236	yes
Zcchc2	chr1:105990405-106034079	11.9731	24.0599	1.00683	0.000195236	yes
Ctsz	chr2:174415803-174438992	309.713	622.324	1.00674	0.000195236	yes
Atf7	chr15:102525945-102625464	3.15119	6.32808	1.00587	0.000195236	yes
Wdr44	chrX:23693050-23806001	4.7366	9.50749	1.00521	0.000195236	yes
Hsph1	chr5:149616844-149636315	73.0126	146.472	1.00441	0.000195236	yes
Catsper2	chr2:121394354-121413792	0.408389	0.818997	1.00391	0.00926854	yes
Cd300lf	chr11:115091430-115162240	3.0228	6.05886	1.00316	0.000195236	yes
Ccdc71l	chr12:32378788-32382943	9.01452	18.062	1.00264	0.000195236	yes
Wipi1	chr11:109573520-109611389	0.24582	0.492427	1.00231	0.0217808	yes
Ano7	chr1:93373895-93404304	0.260628	0.521476	1.00061	0.00766958	yes
Gm16675	chr8:46730969-46739515	0.62505	1.24977	0.999618	0.0106073	yes
Rnf214	chr9:45863690-45936058	5.87702	11.7506	0.999579	0.00789883	yes
Nlk	chr11:78567167-78697425	6.10856	12.2109	0.999265	0.000195236	yes
Pxn	chr5:115506675-115555987	25.5218	51.008	0.998992	0.000195236	yes
Sec24d	chr3:123267495-123365636	12.3742	24.7291	0.998879	0.000195236	yes
Hsp90ab1	chr17:45567777-45573261	945.333	1889.17	0.998857	0.00428914	yes
Nfil3	chr13:52967208-52981039	3.89859	7.78388	0.997536	0.000195236	yes
Trappc6b	chr12:59043091-59061472	37.9659	75.7873	0.997251	0.000195236	yes
1810013L24Rik	chr16:8830099-8858924	15.8958	31.7259	0.997019	0.000195236	yes
Rab23	chr1:33719895-33742564	5.09553	10.1657	0.996403	0.000195236	yes

Adgrl2	chr3:148815585-148954635	1.16072	2.31437	0.9956	0.000195236	yes
Cdk12	chr11:98203304-98253540	14.6387	29.1858	0.995485	0.000195236	yes
Rnf111	chr9:70425428-70503725	12.2056	24.3217	0.994703	0.000195236	yes
Milr1	chr11:106751225-106779537	33.1447	66.0433	0.994634	0.000195236	yes
Gfpt1	chr6:87042845-87092207	18.0222	35.9085	0.994548	0.000195236	yes
Kremen1	chr11:5191552-5261610	1.49982	2.98803	0.994403	0.000195236	yes
Spsb1	chr4:149896283-149955006	0.285991	0.569448	0.993595	0.00825328	yes
Ets2	chr16:95702406-95721049	20.3824	40.5502	0.992382	0.000195236	yes
Fcgr1	chr3:96282908-96293969	14.6895	29.2042	0.991395	0.000195236	yes
Stx6	chr1:155158702-155203517	21.1314	42.0058	0.991204	0.000195236	yes
Tpt1	chr14:75845255-75848303	92.144	182.869	0.988852	0.000195236	yes
Ywhae	chr11:75732886-75765841	228.649	453.754	0.988777	0.000195236	yes
Ranbp10	chr8:105768307-105827350	4.00409	7.94314	0.988233	0.000195236	yes
Fmnl1	chr11:103171137-103198900	22.7124	45.0439	0.987855	0.000195236	yes
Ddhd1	chr14:45593170-45658143	5.85336	11.6083	0.987818	0.000195236	yes
Efcab14	chr4:115738072-115777327	8.99993	17.8408	0.987192	0.000195236	yes
Gxylt1	chr15:93239741-93275084	8.0242	15.9012	0.98671	0.000195236	yes
Tmem192	chr8:64947184-64969037	27.2706	54.0119	0.985932	0.000195236	yes
Lin28b	chr10:45376618-45470201	0.923448	1.82797	0.985141	0.000195236	yes
Gm9776	chr13:94356748-94358923	0.469763	0.929453	0.98445	0.00881201	yes
Tnrc6a	chr7:123123884-123195296	9.89048	19.567	0.98431	0.000195236	yes
Clec4e	chr6:123281788-123289871	58.8961	116.138	0.979596	0.000195236	yes
Aim2	chr1:173420603-173466036	25.3843	50.0533	0.97953	0.000195236	yes
Ssh1	chr5:113942218-113993757	1.06585	2.10069	0.978854	0.000195236	yes
Eif5	chr12:111538100-111546753	138.454	272.787	0.978365	0.000195236	yes
Ralgds	chr2:28513166-28553082	3.3928	6.68421	0.978282	0.000195236	yes
Rc3h2	chr2:37370070-37422903	11.8183	23.2553	0.976541	0.000195236	yes
Mbtd1	chr11:93886218-93946984	7.32822	14.4151	0.976042	0.000195236	yes
Ttll1	chr15:83483768-83510907	2.2036	4.33282	0.975446	0.000195236	yes
Hnrnpd	chr5:99955934-99978938	21.0082	41.3005	0.975208	0.000195236	yes
Rab6a	chr7:100549116-100641268	43.4988	85.4926	0.974824	0.000195236	yes
Pla2g4c	chr7:13325666-13360668	0.431391	0.847354	0.973968	0.00130921	yes
Mknk1	chr4:115839197-115879256	8.61976	16.9237	0.973324	0.000195236	yes
B4galt6	chr18:20684598-20746404	4.97777	9.7705	0.972934	0.000195236	yes
Pip5k1a	chr3:95058529-95106930	8.52305	16.7271	0.972746	0.000195236	yes
Cmip	chr8:117257018-117461505	9.71457	19.0623	0.972498	0.000195236	yes
Drosha	chr15:12824814-12935291	13.2149	25.9242	0.972138	0.000195236	yes
Klf3	chr5:64803522-64830129	1.52917	2.99954	0.971992	0.000195236	yes
Neu1	chr17:34931252-34937297	8.94148	17.539	0.971979	0.000195236	yes
Vprbp	chr9:106821975-106880992	7.20043	14.1227	0.97186	0.000195236	yes
Trim56	chr5:137111285-137116207	7.11276	13.9343	0.97016	0.000195236	yes
Sp110	chr1:85576898-85645036	4.55822	8.92953	0.970114	0.00271906	yes
Ogfr	chr2:180589406-180595837	20.1828	39.5195	0.969436	0.000195236	yes
Ints9	chr14:64950044-65039835	9.22546	18.0526	0.968512	0.000195236	yes
Chmp4b	chr2:154657025-154694783	87.5039	171.081	0.967257	0.000195236	yes
Sccpdh	chr1:179668230-179687184	8.74007	17.0867	0.96716	0.000195236	yes
Atg13	chr2:91674611-91710592	4.14264	8.09873	0.967146	0.000195236	yes
Otud7b	chr3:96080595-96161129	5.663	11.0657	0.96645	0.000195236	yes
Kctd11	chr11:69878263-69880985	1.12576	2.19966	0.966382	0.000195236	yes
Sertad3	chr7:27473839-27477364	2.16115	4.22169	0.966023	0.000195236	yes
Trim14	chr4:46505071-46536141	12.3772	24.1739	0.965765	0.000195236	yes
Cenpj	chr14:56526760-56571846	3.34383	6.52962	0.965497	0.000195236	yes
Tbrg1	chr9:37649181-37657312	111.363	217.382	0.964967	0.000195236	yes
Rhbdf1	chr11:32209584-32222293	0.905563	1.76656	0.964052	0.000698636	yes
Kif1b	chr4:149176318-149307733	9.81058	19.1178	0.962504	0.000195236	yes
Spred2	chr11:19924441-20022597	4.05095	7.88924	0.961628	0.000195236	yes
Fam161b	chr12:84345316-84361821	0.514783	1.002	0.960851	0.000855787	yes
Mlx	chr11:101087289-101095435	33.4543	65.1129	0.960754	0.000195236	yes
Dlgap4	chr2:156613696-156764363	3.96031	7.70761	0.96067	0.000195236	yes
Rala	chr13:17880574-17944217	40.751	79.3057	0.960591	0.000195236	yes
Rmi2	chr16:10835058-10843235	0.967761	1.88168	0.959297	0.000195236	yes
Pxk	chr14:8098212-8165111	9.95499	19.3379	0.957939	0.000195236	yes
Sertad2	chr11:20543252-20653023	5.09458	9.89567	0.957834	0.000195236	yes
Tm4sf20	chr1:82756648-82768456	0.301474	0.585406	0.957401	0.0446338	yes
Eif4g2	chr7:111067984-111083030	155.979	302.738	0.956719	0.000195236	yes

Tmed5	chr5:108121646-108132591	48.636	94.3703	0.956308	0.000195236	yes
Klhl18	chr9:110425925-110476694	2.30801	4.47566	0.955449	0.000195236	yes
Ubxn2a	chr12:4879031-4907520	20.4747	39.6881	0.954865	0.000195236	yes
Phactr4	chr4:132355924-132422446	1.9507	3.78048	0.95458	0.000195236	yes
Chrna2	chr14:66140959-66152948	0.394109	0.763252	0.953566	0.00244513	yes
Safb2	chr17:56562941-56584583	14.2798	27.6487	0.953234	0.000195236	yes
Dusp6	chr10:99263230-99267489	24.1044	46.6378	0.952201	0.000195236	yes
Kctd6	chr14:8214080-8223569	2.29022	4.42964	0.951706	0.000195236	yes
Xpo6	chr7:126101718-126200408	17.8519	34.519	0.951312	0.000195236	yes
Txnrd3	chr6:89643987-89675529	1.40913	2.7247	0.951294	0.000195236	yes
Pfkfb3	chr2:11471430-11553929	3.79963	7.34361	0.95063	0.000195236	yes
Atg4c	chr4:99193933-99259787	6.04426	11.6794	0.950322	0.000195236	yes
Ift20	chr11:78536435-78541473	35.5303	68.6217	0.949615	0.000195236	yes
4921531C22Rik	chr2:179976852-179979013	0.969422	1.87108	0.948672	0.000855787	yes
Fam53c	chr18:34758905-34773760	3.53503	6.82232	0.948539	0.000195236	yes
Tmem63a	chr1:180942517-180975104	3.92652	7.57174	0.947373	0.000195236	yes
Arrdc4	chr7:68736993-68749238	4.49297	8.66306	0.947207	0.00100977	yes
Crebbp	chr16:4084047-4213404	4.80945	9.26663	0.946172	0.000195236	yes
Micu3	chr8:40308050-40386304	4.28811	8.25167	0.944345	0.000195236	yes
Mb21d1	chr9:78430517-78443237	15.3833	29.5926	0.943875	0.000195236	yes
Zscan29	chr2:121158272-121171125	5.97821	11.4961	0.943354	0.000195236	yes
Nsun3	chr16:62734851-62786716	5.79537	11.1418	0.943007	0.000195236	yes
Patl1	chr19:11912398-11945096	6.64369	12.7712	0.942833	0.000195236	yes
Usp53	chr3:122933600-122984447	5.77772	11.0992	0.941877	0.000195236	yes
Arhgap26	chr18:38993144-39376285	2.51492	4.83102	0.941816	0.000195236	yes
Aida	chr1:183297059-183324501	17.5125	33.6175	0.94083	0.000195236	yes
Nfxl1	chr5:72513303-72559645	9.15121	17.5644	0.940623	0.000195236	yes
Slc37a3	chr6:39334770-39377707	3.71526	7.1289	0.940215	0.000195236	yes
Tspyl2	chrX:152336851-152342484	3.42647	6.57342	0.939923	0.000195236	yes
Ubr2	chr17:46928290-47010532	11.6586	22.3301	0.937592	0.000195236	yes
Sec23b	chr2:144556228-144590753	43.6081	83.5166	0.937467	0.000195236	yes
0610030E20Rik	chr6:72347316-72353160	6.04972	11.5826	0.937022	0.000195236	yes
Nupr1	chr7:126623245-126625470	13.3202	25.4986	0.936806	0.000195236	yes
Lonrf1	chr8:36216063-36249516	2.40599	4.60536	0.936684	0.000195236	yes
Psmd3	chr11:98682553-98695978	65.2933	124.923	0.936031	0.000195236	yes
Lig4	chr8:9970019-9976323	2.69436	5.14782	0.934021	0.000195236	yes
Fbrs	chr7:127485220-127491513	1.7078	3.26172	0.933497	0.000195236	yes
Eif2ak1	chr5:143871861-143909839	23.7015	45.2532	0.933041	0.0036408	yes
H2-T10	chr17:36115870-36121444	0.247613	0.472559	0.932404	0.020214	yes
Mark2	chr19:7275395-7341860	9.16855	17.4964	0.93229	0.000195236	yes
Hs3st3b1	chr11:63884692-63922284	3.10941	5.9324	0.931972	0.000195236	yes
Tapbpl	chr6:125224211-125239934	1.81928	3.47067	0.931845	0.000195236	yes
Dmtf1	chr5:9100736-9161776	24.404	46.5392	0.931331	0.000195236	yes
Lrp10	chr14:54464146-54470291	9.90788	18.8906	0.93102	0.000195236	yes
Fem1c	chr18:46504605-46525971	18.4596	35.183	0.930509	0.000195236	yes
Dck	chr5:88765012-88783277	38.6722	73.6895	0.930163	0.000195236	yes
Smg7	chr1:152836994-152902646	27.0087	51.4552	0.929892	0.000195236	yes
Renbp	chrX:73922120-73930850	13.2052	25.145	0.929165	0.000195236	yes
Frs2	chr10:117070126-117148474	3.6305	6.91185	0.928901	0.000195236	yes
H3f3b	chr11:116021960-116024504	506.173	963.592	0.928793	0.000195236	yes
Rap2c	chrX:51003913-51018018	34.7199	66.082	0.928495	0.000195236	yes
1810022K09Rik	chr3:14578670-14611256	123.031	234.005	0.927518	0.0325793	yes
Rtf1	chr2:119675067-119735407	13.7272	26.0963	0.926812	0.000195236	yes
Epb4.1l5	chr1:119545032-119649000	2.2839	4.34123	0.926603	0.000195236	yes
Rab8b	chr9:66843663-66919705	38.2267	72.5757	0.924906	0.000195236	yes
Pmaip1	chr18:66458603-66465558	12.3176	23.3809	0.924607	0.000195236	yes
Ctnnd1	chr2:84600780-84650740	20.8614	39.5946	0.924465	0.000195236	yes
Tmem127	chr2:127247974-127260764	11.3313	21.4952	0.923705	0.000195236	yes
9330111N05Rik	chr13:80964073-81079857	1.38618	2.62903	0.92341	0.00145427	yes
Serpinb9	chr13:33004540-33017955	3.53925	6.70217	0.921185	0.000195236	yes
Rnf121	chr7:102019871-102065132	12.9975	24.5463	0.91727	0.000195236	yes
Dync2h1	chr9:6928502-7177046	4.2963	8.11362	0.91725	0.000195236	yes
P2ry2	chr7:100996567-101012053	1.78042	3.36166	0.916959	0.000195236	yes
Nfkbid	chr7:30423303-30428746	10.3957	19.6243	0.916656	0.000195236	yes
Fndc3b	chr3:27416161-27710439	11.3389	21.3891	0.915591	0.000195236	yes

Ncl	chr1:86344718-86359455	280.623	529.154	0.915057	0.00230418	yes
Wdyhv1	chr15:58141435-58158654	3.85696	7.27062	0.914617	0.000195236	yes
Hsdl2	chr4:59581562-59618694	6.59433	12.4295	0.914472	0.000195236	yes
Ifi27	chr12:103433872-103443680	63.4262	119.522	0.914128	0.00130921	yes
Surf4	chr2:26913377-26933511	81.9834	154.442	0.913664	0.000195236	yes
Trib3	chr2:152337424-152344060	1.26269	2.37841	0.9135	0.000698636	yes
Foxo1	chr3:52268336-52350109	0.862225	1.6226	0.912168	0.000195236	yes
Taf9b	chrX:106206873-106221158	0.646449	1.21652	0.912154	0.00230418	yes
Sh3rf1	chr8:61224170-61396072	2.43273	4.57529	0.911289	0.000195236	yes
Tmem220	chr11:67025153-67035312	0.479392	0.901497	0.911117	0.014404	yes
Stim2	chr5:53998522-54121057	16.1526	30.3471	0.90979	0.000195236	yes
Zbtb10	chr3:9250566-9285332	0.444109	0.834149	0.90939	0.00130921	yes
Secisbp2l	chr2:125736985-125782870	14.5083	27.2318	0.908411	0.000195236	yes
Tcf7l2	chr19:55741809-55933655	0.891361	1.67262	0.90803	0.000698636	yes
Rbm33	chr5:28317188-28419242	5.73247	10.7565	0.907985	0.000195236	yes
Anxa4	chr6:86736839-86793584	47.2064	88.5656	0.907763	0.000195236	yes
Cnot3	chr7:3645268-3677553	4.73118	8.87597	0.907706	0.00202512	yes
Cybb	chrX:9435253-9469324	80.7879	151.561	0.907688	0.000195236	yes
Anpep	chr7:79821802-79842352	1.96886	3.69341	0.907592	0.000195236	yes
Zbed6	chr1:133619870-133661399	13.1795	24.7221	0.907499	0.00733312	yes
Nckap5l	chr15:99422033-99457748	0.543331	1.01912	0.907417	0.000195236	yes
Clip1	chr5:123577793-123684361	19.3928	36.3711	0.907272	0.000195236	yes
Ppp2r2a	chr14:67014055-67072471	9.48733	17.7916	0.907122	0.000195236	yes
Ankrd12	chr17:65967500-66077046	3.56222	6.67331	0.905624	0.000195236	yes
Zc3hav1	chr6:38310496-38354603	36.6308	68.5665	0.904448	0.000195236	yes
Mapk6	chr9:75386781-75410016	46.6736	87.3126	0.903583	0.000195236	yes
Atad2b	chr12:4917352-5047410	11.4309	21.3753	0.903006	0.000195236	yes
P4hb	chr11:120560303-120572936	439.829	822.353	0.902815	0.000195236	yes
Arl5c	chr11:97989579-97996173	0.607405	1.13484	0.901757	0.011269	yes
Lmbrd2	chr15:9111981-9197450	11.152	20.8313	0.901453	0.0205916	yes
1810037I17Rik	chr3:122924396-122926194	49.9686	93.2873	0.900658	0.000195236	yes
Gopc	chr10:52337023-52382124	18.3431	34.242	0.900534	0.000195236	yes
Myo19	chr11:84876312-84916356	5.72304	10.6823	0.900367	0.00984695	yes
Acss2	chr2:155518042-155562743	2.53608	4.73153	0.899706	0.000195236	yes
Atp1b3	chr9:96332674-96364299	234.195	436.846	0.899417	0.000195236	yes
Cdkl3	chr11:52004220-52084667	1.31541	2.45356	0.899366	0.000698636	yes
Cnnm4	chr1:36471596-36508776	7.00496	13.0636	0.899106	0.000195236	yes
Tbc1d15	chr10:115197870-115251493	11.3989	21.2487	0.898474	0.000195236	yes
E330033B04Rik	chr15:96268563-96275275	0.645325	1.20282	0.898324	0.000537301	yes
Slc39a1	chr3:90248191-90253612	33.5734	62.5687	0.898121	0.000195236	yes
Ptbp3	chr4:59471867-59549364	89.3412	166.478	0.897938	0.000195236	yes
Sppl2a	chr2:126890394-126933235	48.5091	90.3759	0.897684	0.000195236	yes
Slc30a7	chr3:115938972-116007406	11.1437	20.7466	0.896653	0.000195236	yes
Mccc1	chr3:35959305-36053547	3.75038	6.98039	0.896272	0.000195236	yes
Tstd3	chr4:21727700-21767211	14.2083	26.4408	0.896039	0.0482697	yes
Cln3	chr7:126571399-126584280	12.4043	23.0776	0.895646	0.000195236	yes
Plekhb2	chr1:34849958-34879585	45.2198	84.123	0.895547	0.000195236	yes
Insr	chr8:3150921-3279617	2.29137	4.26238	0.895448	0.000195236	yes
Snap29	chr16:17280350-17430826	19.0893	35.478	0.894164	0.00216236	yes
Alas1	chr9:106233454-106247954	20.9389	38.9125	0.894049	0.000195236	yes
Tlr1	chr5:64924679-64933558	5.94444	11.0428	0.893494	0.000195236	yes
Dnajc28	chr16:91614256-91618999	0.293109	0.544407	0.89325	0.00755508	yes
Pnpt1	chr11:29130750-29161828	61.035	113.338	0.892918	0.000195236	yes
Cacfd1	chr2:27009925-27021089	6.11596	11.356	0.8928	0.000195236	yes
Rab21	chr10:115289861-115315591	37.028	68.7505	0.892754	0.000195236	yes
Rgp1	chr4:43562633-43587487	2.49718	4.63247	0.891482	0.0221562	yes
Brdt	chr5:107331193-107387058	0.362034	0.671528	0.891324	0.00311626	yes
Ulk1	chr5:110784488-110810081	0.656096	1.21696	0.8913	0.000537301	yes
Erc1	chr6:119570795-119848150	5.56405	10.319	0.891098	0.000195236	yes
Nrd1	chr4:109000804-109202272	49.3587	91.5245	0.890854	0.000195236	yes
Neto2	chr8:85636587-85691009	0.568483	1.05377	0.890375	0.00130921	yes
Hoxb3	chr11:96323125-96347930	0.503548	0.933245	0.890128	0.00116073	yes
D330050G23Rik	chr2:116900151-116912791	2.25755	4.18316	0.889836	0.00145427	yes
Itpripl1	chr2:127138768-127143457	18.5951	34.4488	0.88953	0.000195236	yes
Fsd1l	chr4:53631470-53707009	1.04817	1.94173	0.88947	0.00100977	yes

Scoc	chr8:83434491-83458396	13.2287	24.4857	0.888269	0.000195236	yes
Naip6,Naip7	chr13:100281120-100317688	2.00657	3.714	0.888243	0.000195236	yes
Ap3m1	chr14:21033741-21052442	34.5063	63.8661	0.888192	0.000195236	yes
Plaa	chr4:94565138-94603247	46.9313	86.8229	0.887526	0.000195236	yes
Fbxw17	chr13:50417876-50433769	4.0073	7.4118	0.887192	0.000195236	yes
Ccdc112	chr18:46282150-46311928	1.22381	2.26352	0.887183	0.000370691	yes
Ccdc97	chr7:25711116-25719053	3.67012	6.78803	0.887164	0.000195236	yes
Eme2	chr17:24892151-24895087	8.86567	16.3923	0.886713	0.000195236	yes
Atxn2	chr5:121711608-121814950	5.83929	10.7952	0.886525	0.000195236	yes
Hif1a	chr12:73907866-73947530	94.2322	174.208	0.886518	0.000195236	yes
Slc38a6	chr12:73286847-73354045	3.8234	7.0674	0.886321	0.000195236	yes
Rsbn1	chr3:103914119-104007491	19.9799	36.9151	0.885662	0.000195236	yes
Eea1	chr10:95940662-96045518	25.3593	46.8247	0.884755	0.000195236	yes
Rab18	chr18:6765166-6791606	31.3794	57.9034	0.883831	0.000195236	yes
AW549877	chr15:3982034-3995752	9.74831	17.986	0.88365	0.000195236	yes
Abcc1	chr16:14361557-14474878	34.535	63.6645	0.88243	0.000195236	yes
Ralgapa2	chr2:146241298-146512004	2.96669	5.46895	0.88241	0.000195236	yes
Hck	chr2:153108467-153151441	41.9141	77.2529	0.882153	0.000195236	yes
Alg11	chr8:22060720-22071627	9.21034	16.9614	0.880933	0.000195236	yes
Wac	chr18:7868831-7929028	31.5192	58.0113	0.880104	0.000195236	yes
Zfp658	chr7:43562369-43575461	1.68437	3.09843	0.87933	0.000195236	yes
Zmiz2	chr11:6395124-6406162	3.92368	7.21741	0.879272	0.000195236	yes
Slc35e2	chr4:155601415-155624755	5.39063	9.91371	0.878972	0.000195236	yes
Ifi47,Olfr56	chr11:49037659-49135387	3.27669	6.01968	0.877446	0.003245	yes
Dhrs3	chr4:144892826-144927645	12.2453	22.474	0.876034	0.000195236	yes
Alg12	chr15:88805242-88819318	5.59609	10.2685	0.875741	0.000195236	yes
Orai1	chr5:123015073-123030452	9.84187	18.0501	0.874999	0.000195236	yes
Synrg	chr11:83964430-84044576	4.65429	8.53435	0.874718	0.000195236	yes
Hnrnpul2	chr19:8819400-8834142	19.7929	36.2834	0.874327	0.000195236	yes
Fam114a1	chr5:64970074-65041901	1.43311	2.62711	0.874326	0.000195236	yes
Galnt6	chr15:100689914-100729376	4.68904	8.59525	0.874246	0.000195236	yes
Map4	chr9:109931773-110083954	10.1968	18.6871	0.873927	0.000195236	yes
Dbn1	chr13:55473427-55488076	0.350068	0.640765	0.87216	0.0110431	yes
Zfp597	chr16:3861543-3872374	4.42491	8.09859	0.872023	0.000195236	yes
Sp4	chr12:118231685-118301440	3.10208	5.67422	0.871186	0.000195236	yes
Sec61a2	chr2:5870986-5895353	8.23214	15.0542	0.870823	0.000195236	yes
Tmem87b	chr2:128818302-128854261	9.06374	16.5744	0.870781	0.000195236	yes
Mtm1	chrX:71210766-71315295	3.66285	6.6934	0.869773	0.000195236	yes
Taf12	chr4:132274374-132293330	41.591	75.9646	0.869055	0.000195236	yes
Stac2	chr11:98036623-98053462	0.300527	0.548811	0.868816	0.00984695	yes
Sos2	chr12:69583760-69681852	10.3052	18.8029	0.867588	0.000195236	yes
Rwdd4a	chr8:47533644-47552837	36.2252	66.0672	0.866942	0.000195236	yes
5330426P16Rik	chr16:50726750-50732773	4.13887	7.53818	0.864981	0.000195236	yes
Helz	chr11:107547959-107686943	4.21399	7.6734	0.864677	0.000195236	yes
Nfe2l1	chr11:96817413-96829968	22.4696	40.9095	0.864465	0.000195236	yes
Uvssa	chr5:33378695-33419754	1.89709	3.45381	0.864403	0.000195236	yes
Bnip3	chr7:138890835-138909506	57.0517	103.828	0.86386	0.000195236	yes
3110002H16Rik	chr18:12168729-12236386	21.4413	38.9988	0.86304	0.00467182	yes
Abracl	chr10:18011259-18023252	108.088	196.592	0.863004	0.000195236	yes
Bzrap1	chr11:87760540-87785928	0.407139	0.740057	0.862116	0.000370691	yes
Tmem68	chr4:3549040-3574768	30.6546	55.6955	0.861457	0.000195236	yes
Fyco1	chr9:123789499-123851899	1.32428	2.40512	0.860901	0.000195236	yes
Fndc7	chr3:108853677-108890008	0.261767	0.475216	0.860301	0.00675438	yes
Rusc2	chr4:43381981-43429134	3.76448	6.8326	0.859982	0.000195236	yes
Ermp1	chr19:29609882-29648420	22.8696	41.5007	0.859704	0.000195236	yes
Irf1	chr11:53770013-53778374	8.70599	15.7899	0.858917	0.000195236	yes
Olfr934	chr9:38982109-38983042	1.5602	2.82855	0.858333	0.0442183	yes
Upf2	chr2:5951468-6056703	15.8995	28.7946	0.856819	0.000195236	yes
Lclat1	chr17:73107984-73243366	8.03917	14.5571	0.856607	0.000195236	yes
Myo7a	chr7:98051053-98119493	12.4014	22.4314	0.855012	0.000195236	yes
Klhdc1	chr12:69241831-69283961	2.12759	3.84553	0.853961	0.000195236	yes
Zcchc10	chr11:53324688-53333301	10.4001	18.7929	0.853598	0.000195236	yes
Myh9	chr15:77760584-77842175	52.875	95.5424	0.853556	0.000195236	yes
Gm5434	chr12:36090378-36091829	7.84468	14.1741	0.853466	0.000370691	yes
Malt1	chr18:64887755-65689436	24.1781	43.6347	0.851774	0.000195236	yes

Taf13	chr3:108571698-108582068	22.6939	40.941	0.85124	0.000195236	yes
Cpox	chr16:58670207-58680389	11.2885	20.3619	0.851021	0.000195236	yes
Riok3	chr18:12128849-12157367	27.8857	50.298	0.850977	0.000195236	yes
A230046K03Rik	chr10:83543940-83596473	42.2393	76.1501	0.850259	0.000195236	yes
Vma21	chrX:71816079-71824706	24.4494	44.0384	0.848963	0.000195236	yes
Gys1	chr7:45434838-45456617	10.8788	19.5774	0.847672	0.000195236	yes
Acvrl1	chr15:101128521-101145336	2.41281	4.33999	0.84698	0.000195236	yes
Xkr8	chr4:132724903-132732546	1.06236	1.91005	0.846337	0.000537301	yes
Lca5	chr9:83391354-83454574	3.0202	5.42743	0.845626	0.000195236	yes
Pdcd1	chr1:94038304-94052553	5.75086	10.3341	0.845565	0.000195236	yes
Usp47	chr7:112023505-112111386	32.4348	58.2378	0.844413	0.000195236	yes
Pde4dip	chr3:97689828-97888707	16.8628	30.2724	0.844157	0.000195236	yes
Dnm2	chr9:21424907-21510186	24.858	44.6137	0.843776	0.000698636	yes
Mreg	chr1:72159232-72212307	0.515722	0.92544	0.843545	0.0155848	yes
Pcgf2	chr11:97688822-97700497	0.804145	1.44245	0.842988	0.00130921	yes
Pstpip1	chr9:56089975-56128890	77.1484	138.336	0.842472	0.000195236	yes
Arhgap31	chr16:38598342-38713035	2.07265	3.71505	0.841909	0.000195236	yes
Rbm27	chr18:42275352-42341540	19.0005	34.0546	0.841814	0.000195236	yes
Hdlbp	chr1:93405939-93478917	96.9006	173.637	0.841498	0.000195236	yes
Lman1	chr18:65980738-66002635	91.163	163.318	0.841168	0.000537301	yes
Psme4	chr11:30771774-30880361	29.7392	53.2702	0.840963	0.000195236	yes
4930455G09Rik	chr4:142017897-142028995	3.83753	6.87227	0.84061	0.00870444	yes
Pdgfb	chr15:79995875-80014808	15.6379	28.0039	0.840585	0.000195236	yes
Rnf20	chr4:49632059-49656886	31.5867	56.542	0.840005	0.000195236	yes
Atf7ip	chr6:136518850-136607379	5.79515	10.3664	0.838994	0.000195236	yes
Map4k5	chr12:69746847-69987002	17.3243	30.9831	0.838683	0.00188078	yes
A830080D01Rik	chrX:159532667-159593081	7.95913	14.2334	0.838597	0.000195236	yes
Ghdc	chr11:100766331-100770957	1.59121	2.8453	0.838452	0.00130921	yes
Slc31a1	chr4:62360700-62391769	16.8477	30.1134	0.837859	0.000195236	yes
Kdm7a	chr6:39118472-39206773	10.2216	18.2663	0.837563	0.000195236	yes
Ifrd1	chr12:40203128-40223189	121.687	217.418	0.837291	0.000195236	yes
Slc25a47	chr12:108851128-108856815	0.604468	1.07953	0.836667	0.0228548	yes
Snx13	chr12:35047188-35147477	12.2681	21.9046	0.83632	0.000195236	yes
Rabgef1	chr5:130171818-130214337	7.04436	12.5698	0.835418	0.000195236	yes
Ficd	chr5:113735781-113740607	1.25516	2.2372	0.833824	0.00116073	yes
Tbc1d8b	chrX:139684995-139754218	4.86124	8.6602	0.833077	0.000195236	yes
Pbxip1	chr3:89436703-89450952	5.70368	10.1598	0.832901	0.000195236	yes
Cldnd1	chr16:58727909-58734247	10.3334	18.39	0.831599	0.000195236	yes
Dopey2	chr16:93711906-93810588	1.10209	1.96078	0.83119	0.0256621	yes
Xrn1	chr9:95954759-96053029	10.286	18.2852	0.83	0.000195236	yes
Serp1	chr3:58521970-58557501	108.255	192.428	0.829879	0.00100977	yes
Mob1b	chr5:88720870-88758455	10.3558	18.4046	0.829624	0.000195236	yes
Trip11	chr12:101837371-101913171	11.7573	20.888	0.829123	0.000195236	yes
Uhrf1bp1l	chr10:89744990-89819869	14.3191	25.4212	0.828089	0.000195236	yes
Ube3a	chr7:59228749-59306727	65.1551	115.665	0.828008	0.000195236	yes
Slc9a1	chr4:133369771-133423698	1.14586	2.03392	0.827831	0.0187817	yes
Prkca	chr11:107933386-108343888	2.66323	4.72481	0.827082	0.000195236	yes
Pnpla8	chr12:44269153-44313435	12.6275	22.3945	0.826579	0.000195236	yes
Adm	chr7:110627668-110629819	2.26124	4.00999	0.826486	0.00130921	yes
Lrrfip2	chr9:111118110-111225668	21.4953	38.097	0.825658	0.000195236	yes
Ccdc127	chr13:74350316-74365783	22.8992	40.5578	0.824682	0.000195236	yes
Clk3	chr9:57750710-57765860	20.6761	36.6112	0.824321	0.000195236	yes
Nt5dc1	chr10:34303611-34418528	17.1272	30.3052	0.823279	0.000195236	yes
Blcap	chr2:157556361-157566361	10.351	18.3057	0.822518	0.000370691	yes
Atf2	chr2:73816508-73892639	19.8755	35.1368	0.821994	0.000195236	yes
Kat2b	chr17:53566970-53672721	9.51284	16.8158	0.821869	0.000195236	yes
Atp6v1a	chr16:44085403-44139019	83.2045	147.036	0.821431	0.000195236	yes
St18	chr1:6487230-6860940	4.22331	7.45782	0.82038	0.000195236	yes
Magohb	chr6:131284388-131293244	8.86191	15.6485	0.820332	0.000370691	yes
Ginm1	chr10:7767946-7780917	20.3769	35.9543	0.819229	0.000195236	yes
Cyp4f13	chr17:32924687-32947361	2.44176	4.30801	0.819102	0.000537301	yes
Map2k4	chr11:65688243-65788297	15.2351	26.8759	0.818916	0.000195236	yes
Ywhaz	chr15:36770261-36796929	62.9489	111.04	0.818828	0.000195236	yes
Larp1b	chr3:40950630-40977793	17.5256	30.9126	0.81873	0.000195236	yes
Erp44	chr4:48193330-48279589	59.8753	105.61	0.818714	0.000195236	yes

Suco	chr1:161816111-161876661	12.5284	22.0975	0.81868	0.000195236	yes
Ece1	chr4:137862236-137965229	0.716492	1.26296	0.817782	0.000698636	yes
Ube4a	chr9:44923126-44965600	7.72202	13.6111	0.81773	0.000195236	yes
Spag9	chr11:93996090-94126082	20.9198	36.8591	0.817151	0.000195236	yes
Hmga1	chr17:27556573-27563672	20.4124	35.9631	0.817072	0.000195236	yes
Sec63	chr10:42761495-42832514	16.7206	29.4522	0.816749	0.000195236	yes
Nipbl	chr15:8289823-8444463	16.3563	28.7987	0.81616	0.000195236	yes
Lcor	chr19:41549638-41559781	4.34623	7.65159	0.815993	0.000195236	yes
Abi1	chr2:22895521-23040241	69.1513	121.707	0.815582	0.000195236	yes
Zfr	chr15:12117850-12185449	39.7323	69.8965	0.81491	0.000195236	yes
Gm29766	chr5:110361751-110448503	1.69458	2.97991	0.81434	0.0432236	yes
Rnf141	chr7:110816534-110844381	15.2537	26.817	0.813992	0.000195236	yes
Aph1a	chr3:95893920-95904639	6.26348	11.0078	0.81349	0.000195236	yes
A630089N07Rik	chr16:98062511-98082439	5.14414	9.04035	0.81345	0.000370691	yes
Atp10d	chr5:72203328-72298771	4.89344	8.59381	0.81245	0.000195236	yes
Tox4	chr14:52261758-52305124	17.0429	29.9123	0.81157	0.00145427	yes
Pfdn2	chr1:171345698-171358170	40.4249	70.9483	0.811525	0.000195236	yes
Dr1	chr5:108268896-108280521	47.5509	83.38	0.810227	0.000195236	yes
Rnf14	chr18:38296634-38317849	13.5864	23.8107	0.809444	0.000195236	yes
P4ha1	chr10:59323295-59373304	37.7482	66.1401	0.809116	0.000195236	yes
Rnasek	chr11:70238122-70239852	80.8301	141.567	0.808518	0.000195236	yes
Oaz2	chr9:65676547-65690300	7.23134	12.6626	0.808245	0.000195236	yes
Med12	chrX:101274090-101298934	3.2617	5.70837	0.807455	0.000195236	yes
Exoc6b	chr6:84618485-85069513	8.88043	15.5417	0.807439	0.000195236	yes
Fam98a	chr17:75537085-75551946	34.6591	60.6552	0.807399	0.000195236	yes
Zfp592	chr7:80993683-81045162	2.66504	4.6636	0.807287	0.000195236	yes
Ccnt1	chr15:98543210-98570864	24.8013	43.3835	0.806732	0.000195236	yes
Nr4a1	chr15:101266845-101274794	1.29133	2.25782	0.80607	0.00230418	yes
Hic2	chr16:17233586-17263430	0.817696	1.42945	0.805824	0.000698636	yes
Fam50a	chrX:74313032-74320149	27.1903	47.528	0.805684	0.000195236	yes
Tfe3	chrX:7762660-7775202	13.1659	23.0117	0.805563	0.000195236	yes
Hoxb4	chr11:96318266-96321638	1.66618	2.91054	0.804746	0.00100977	yes
1810055G02Rik	chr19:3708332-3717881	14.1659	24.7206	0.803288	0.000195236	yes
Ell	chr8:70539674-70592858	3.72775	6.50337	0.802881	0.000195236	yes
Psen1	chr12:83688562-83735199	33.0628	57.6613	0.802397	0.000195236	yes
Hspa5	chr2:34772089-34776529	598.93	1044.48	0.80232	0.00202512	yes
Sema4c	chr1:36548638-36558381	1.41866	2.47351	0.802028	0.00116073	yes
Pde8a	chr7:81213803-81333622	12.1743	21.2261	0.802001	0.000195236	yes
Ccdc55	chr11:77044291-77078437	21.2664	37.0432	0.800632	0.000195236	yes
Cdk18	chr1:132113546-132139685	14.0758	24.5165	0.800534	0.000195236	yes
Csnk1g3	chr18:53862112-53955684	13.7838	24.0057	0.800407	0.000195236	yes
Slc20a1	chr2:129198772-129211612	39.4778	68.7485	0.800286	0.000195236	yes
Rnft1	chr11:86415843-86544807	12.8153	22.3098	0.799814	0.0472165	yes
Celf1	chr2:90940396-91019497	18.4796	32.1515	0.798953	0.000195236	yes
Dot1l	chr10:80755205-80794347	6.20245	10.7893	0.798697	0.000195236	yes
Raet1a,Raet1b,Raet1c,Ra	chr10:22158608-22374139	94.4925	164.312	0.798169	0.000195236	yes
Chac1	chr2:119351241-119354327	2.67743	4.65541	0.798059	0.00188078	yes
Mns1	chr9:72438528-72491959	4.10219	7.13143	0.797796	0.0169236	yes
Ammecr1	chrX:142853473-142966728	10.8319	18.8259	0.797427	0.000195236	yes
Vav3	chr3:109340682-109685694	8.80456	15.2921	0.796464	0.000195236	yes
Plscr3	chr11:69846371-69858730	3.59028	6.23116	0.795405	0.000537301	yes
Atat1	chr17:35897597-35910068	2.19645	3.81171	0.795263	0.00160026	yes
Zfyve16	chr13:92487748-92530810	9.19533	15.9525	0.794814	0.000195236	yes
Lrch3	chr16:32914099-33056186	11.0264	19.1268	0.794636	0.000195236	yes
Srxn1	chr2:152105523-152111376	25.4175	44.0862	0.794507	0.000195236	yes
Mob3a	chr10:80685252-80701820	8.99846	15.6044	0.794204	0.000195236	yes
Arid4a	chr12:71015966-71099351	11.0733	19.2	0.794015	0.000195236	yes
Baz1a	chr12:54892988-54986336	47.8764	82.9826	0.793495	0.000195236	yes
Rybp	chr6:100228564-100287358	5.01101	8.68526	0.793467	0.000195236	yes
Ppp3cc	chr14:70217864-70289497	13.8029	23.9235	0.793459	0.000195236	yes
Pafah2	chr4:134396319-134427412	3.48817	6.04019	0.792125	0.000370691	yes
Kdm6a	chrX:18162666-18279358	8.86036	15.3396	0.791824	0.000195236	yes
Mob3b	chr4:34949073-35157484	0.944579	1.63516	0.791686	0.000855787	yes
Dennd4b	chr3:90266513-90280665	3.07435	5.32146	0.791541	0.000195236	yes
Scamp5	chr9:57441326-57468060	2.70016	4.67356	0.791479	0.000537301	yes

Ago3	chr4:126340677-126429542	3.78875	6.55391	0.790635	0.000195236	yes
Usp25	chr16:77014068-77116780	46.225	79.9376	0.790201	0.000195236	yes
Rnasel	chr1:153749425-153764221	2.18223	3.77366	0.790165	0.000370691	yes
Mgat5	chr1:127204985-127482972	6.90804	11.9286	0.78808	0.000370691	yes
Gbf1	chr19:46152557-46286510	9.67036	16.6908	0.787413	0.000195236	yes
Slc25a37	chr14:69241850-69285103	6.39369	11.0269	0.786312	0.000195236	yes
Pgpep1	chr8:70646435-70659738	1.35063	2.32918	0.786191	0.000537301	yes
Yod1	chr1:130689042-130729253	10.3589	17.8626	0.78607	0.0116945	yes
Wasf2	chr4:133130632-133198330	10.5872	18.2551	0.785987	0.000195236	yes
Bbx	chr16:50191843-50432389	16.2907	28.0831	0.785649	0.000195236	yes
Reep3	chr10:67005074-67096988	16.4702	28.3675	0.784383	0.000195236	yes
Yipf5	chr18:40204864-40219399	26.0334	44.8239	0.783903	0.000195236	yes
Ptpn12	chr5:20986644-21055797	29.1775	50.2285	0.783648	0.000195236	yes
Snx14	chr9:88376746-88438951	26.98	46.4402	0.783486	0.000195236	yes
Ppp2r5e	chr12:75450880-75596200	9.05835	15.5899	0.783291	0.000195236	yes
Arhgap29	chr3:121953325-122016153	0.557388	0.959289	0.783282	0.00351242	yes
Slc46a3	chr5:147878440-147894802	1.01653	1.74916	0.783013	0.00541779	yes
Dock6	chr9:21800179-21852635	0.475805	0.818613	0.782811	0.00188078	yes
Mon1b,Syce1l	chr8:113635585-113655533	7.38907	12.6972	0.781043	0.000195236	yes
Slc35e1	chr8:72477994-72492614	12.5293	21.5292	0.780987	0.000195236	yes
Ago2	chr15:73101624-73184947	9.16724	15.7501	0.7808	0.000195236	yes
Npc1	chr18:12168729-12236386	9.24555	15.8815	0.780517	0.0080155	yes
Lrrc8a	chr2:30237768-30263790	5.77332	9.91526	0.780249	0.000195236	yes
Trio	chr15:27730648-28025848	7.69565	13.216	0.780174	0.000195236	yes
Lrrc57	chr2:120604237-120621559	9.73742	16.7217	0.78011	0.0190803	yes
Gm4285	chr14:75842917-75844964	0.750961	1.28943	0.779917	0.0294593	yes
Esf1	chr2:140119880-140170558	34.8328	59.7972	0.779635	0.000195236	yes
Tmem170b	chr13:41606215-41641357	9.82014	16.8535	0.779233	0.000195236	yes
Slmap	chr14:26413174-26533740	24.2804	41.6599	0.778867	0.000195236	yes
Acbd3	chr1:180726042-180754204	25.8152	44.2887	0.77872	0.000195236	yes
Cd300ld	chr11:114982445-114989886	13.3965	22.9707	0.777942	0.000195236	yes
Myd88	chr9:119335987-119340040	30.0862	51.5855	0.777862	0.000195236	yes
Gk5	chr9:96119428-96182953	3.3698	5.77576	0.777347	0.000370691	yes
Ubqln2	chrX:153498231-153501558	4.57458	7.84011	0.777236	0.000370691	yes
Dnajc3	chr14:118937931-118981702	52.3782	89.7617	0.777133	0.000195236	yes
Parp4	chr14:56575618-56659798	1.72899	2.96298	0.777121	0.000195236	yes
Wdr26	chr1:181173225-181211978	32.6227	55.8844	0.776567	0.000195236	yes
Chrnb1	chr11:69784035-69795937	0.47637	0.815806	0.776143	0.0347296	yes
Atg2a	chr19:6241667-6262304	1.85243	3.1718	0.775882	0.000195236	yes
Ap2b1	chr11:83302696-83405033	28.6658	49.0796	0.775793	0.000195236	yes
Jade2	chr11:51813455-51857481	5.9989	10.2691	0.775534	0.000195236	yes
Elac1	chr18:73735037-73754479	4.36796	7.47403	0.774925	0.000195236	yes
Eif4ebp2	chr10:61432496-61452669	8.02133	13.7224	0.77462	0.000195236	yes
Gpd2	chr2:57237677-57370719	13.4528	23.0079	0.774219	0.000195236	yes
Mthfd2l	chr5:90931195-91021370	2.47758	4.23727	0.7742	0.00100977	yes
Hip1r	chr5:123973627-124003215	1.07154	1.83224	0.773924	0.00116073	yes
Klhl20	chr1:161088377-161131479	9.55003	16.3246	0.773471	0.000195236	yes
Fam217b	chr2:178414533-178422161	2.84709	4.86661	0.773432	0.000855787	yes
Slc41a2	chr10:83231138-83337817	6.62678	11.3156	0.771936	0.000195236	yes
Lrrfip1	chr1:90998726-91128944	56.1928	95.8483	0.770368	0.000370691	yes
Tnip3	chr6:65590397-65634040	37.3143	63.545	0.768047	0.000195236	yes
Nomo1	chr7:46033695-46084212	29.7253	50.6084	0.767687	0.000195236	yes
Tspan31	chr10:127067289-127070261	38.8467	66.1334	0.767585	0.000195236	yes
Plekha8	chr6:54595110-54645822	7.98029	13.578	0.76676	0.000195236	yes
Wsb1	chr11:79239381-79254695	111.519	189.732	0.766673	0.000195236	yes
Ifitm2	chr7:140954838-140955961	333.433	567.155	0.766348	0.000195236	yes
Zbtb41	chr1:139422382-139453007	21.3281	36.2322	0.764514	0.000195236	yes
Sec61a1	chr6:88503606-88518800	97.1106	164.897	0.763862	0.000195236	yes
Slc9a3r1	chr11:115163340-115181178	10.8557	18.4288	0.763518	0.000195236	yes
Zbtb33	chrX:38189792-38252481	6.05125	10.27	0.763128	0.000195236	yes
Ppp1r10	chr17:35917195-35932283	12.1451	20.6073	0.762781	0.000195236	yes
Lpin1	chr12:16535668-16589770	1.35391	2.29691	0.762557	0.000195236	yes
Kif3a	chr11:53567368-53604246	5.69625	9.66307	0.762469	0.000195236	yes
Entpd7	chr19:43689688-43753000	3.84297	6.51671	0.761921	0.0239982	yes
Prrc2a	chr17:35149085-35164877	11.4677	19.4402	0.761465	0.000195236	yes

Hyou1	chr9:44379489-44392369	49.941	84.6436	0.761178	0.000195236	yes
Tmx4	chr2:134594501-134644121	12.5857	21.3299	0.761097	0.000195236	yes
Pafah1b2	chr9:45965310-45984871	61.9842	105.021	0.7607	0.000195236	yes
Rspry1	chr8:94601940-94660276	17.3788	29.4238	0.759654	0.000195236	yes
Rab5b	chr10:128677182-128696268	10.7595	18.2156	0.759572	0.000195236	yes
Rrn3	chr16:13780698-13814841	55.7564	94.393	0.759544	0.000195236	yes
Cltb	chr13:54592938-54611272	59.1926	100.203	0.759434	0.000195236	yes
BC048403	chr10:121739936-121752859	1.7319	2.93144	0.759251	0.00145427	yes
Rab11a	chr9:64715299-64737756	60.9963	103.236	0.759152	0.000195236	yes
Rhof	chr5:123118179-123132629	12.051	20.3956	0.759098	0.000370691	yes
Ubr3	chr2:69897245-70024010	13.6821	23.1538	0.758953	0.000195236	yes
Cdc73	chr1:143607498-143702684	17.1424	29.0092	0.758941	0.000195236	yes
Sh2b3	chr5:121815480-121836859	15.6645	26.5043	0.758726	0.000195236	yes
Fam63a	chr3:95282934-95307176	5.12056	8.66272	0.75852	0.000698636	yes
Znrf1	chr8:111536639-111626030	2.78404	4.70595	0.757309	0.000537301	yes
H2-K1	chr17:33996011-34000333	91.674	154.95	0.757219	0.000195236	yes
Sfxn4	chr19:60837276-60861430	0.482611	0.815502	0.756829	0.0270102	yes
Bcl9	chr3:97203661-97227364	0.869487	1.469	0.756594	0.000855787	yes
Cd5	chr19:10718142-10738974	0.407228	0.687777	0.756103	0.0278914	yes
Wnk1	chr6:119923968-120038655	18.4589	31.1534	0.755073	0.000195236	yes
Klhl28	chr12:64942439-64965536	8.16882	13.7769	0.754056	0.000195236	yes
Acsl1	chr8:46471036-46536051	17.8379	30.0808	0.753895	0.000195236	yes
Hax1	chr3:89995445-89998716	16.1441	27.2232	0.753831	0.000195236	yes
Frrs1	chr3:116859566-116903750	35.1649	59.2749	0.753286	0.000195236	yes
Wbp2	chr11:116078572-116086964	9.6125	16.1959	0.752649	0.000370691	yes
Dgke	chr11:89037581-89060748	4.44159	7.48344	0.752627	0.000195236	yes
Tbx20	chr9:24720811-24774303	0.333408	0.561725	0.752574	0.0126632	yes
Insl6	chr19:29321353-29325318	3.97875	6.70326	0.752548	0.00517183	yes
Paf1	chr7:28392995-28399383	45.8594	77.253	0.752372	0.000195236	yes
Plekhb1	chr7:100643895-100662394	0.530582	0.893459	0.751826	0.0409198	yes
Ldlr	chr9:21723575-21749918	20.1112	33.8593	0.751556	0.000195236	yes
Prdx5	chr19:6906818-6909645	78.4385	132.031	0.751237	0.000195236	yes
Ap3b1	chr13:94358959-94566316	37.2519	62.6903	0.750928	0.000195236	yes
Gtf2a1	chr12:91555261-91590487	21.8381	36.7443	0.750672	0.000195236	yes
Prkaa1	chr15:5143860-5181899	24.4478	41.1349	0.75066	0.000195236	yes
Tgoln1	chr6:72608420-72617000	22.4448	37.759	0.750443	0.000195236	yes
Arl15	chr13:113794507-114157461	8.24499	13.8691	0.750287	0.000195236	yes
Sep-11	chr5:93093456-93174958	47.248	79.4709	0.750173	0.000195236	yes
Pja2	chr17:64281005-64331883	20.1446	33.8814	0.750101	0.000195236	yes
Phf21a	chr2:92184181-92364666	2.4487	4.11621	0.7493	0.000537301	yes
Hspa2	chr12:76404175-76406936	0.472275	0.793514	0.74863	0.0186727	yes
Tab3	chrX:85574021-85634469	4.20386	7.06308	0.748582	0.000195236	yes
Hspb11	chr4:107253933-107279888	43.3324	72.7909	0.748312	0.000698636	yes
Mef2d	chr3:88142394-88169167	7.17822	12.0576	0.748241	0.000195236	yes
Ccdc25	chr14:65837301-65866604	20.4502	34.3501	0.748197	0.000195236	yes
Ctnna1	chr18:35118911-35254775	61.4677	103.247	0.748195	0.000195236	yes
Bcat1	chr6:144993834-145076157	25.2188	42.3526	0.747953	0.000195236	yes
Psmd8	chr7:29174186-29180673	169.861	285.208	0.74766	0.000195236	yes
Mtpn	chr6:35508823-35539888	125.698	211.029	0.747476	0.000195236	yes
Prss16	chr13:22002175-22009741	1.3679	2.29463	0.746305	0.00778355	yes
Gatad2b	chr3:90341653-90358120	5.70884	9.57563	0.746171	0.000370691	yes
Jak1	chr4:101069037-101265282	36.2729	60.816	0.745559	0.000195236	yes
Lrrc59	chr11:94629823-94653754	149.95	251.279	0.744812	0.000195236	yes
Bdh1	chr16:31422296-31458901	4.17368	6.9932	0.744634	0.000370691	yes
C2cd2l	chr9:44309236-44320282	2.00591	3.36027	0.744322	0.000195236	yes
Ppp1r11	chr17:36948354-36951792	44.8303	75.0385	0.743157	0.000195236	yes
Atp10a	chr7:58658201-58829426	1.09893	1.83926	0.743034	0.00130921	yes
Mfn1	chr3:32529481-32579225	7.01319	11.7378	0.743023	0.000537301	yes
Foxn3	chr12:99195093-99450074	6.08833	10.1898	0.743005	0.000195236	yes
Psmc1	chr12:100112330-100123364	106.066	177.473	0.742638	0.000370691	yes
Nol4l	chr2:153407460-153529971	1.33812	2.23763	0.741766	0.00100977	yes
Mier1	chr4:103114389-103165754	18.8102	31.446	0.741356	0.000195236	yes
Agpat3	chr10:78271562-78351700	22.3841	37.3979	0.740482	0.000195236	yes
Cnot4	chr6:35022064-35133737	11.2964	18.8668	0.739989	0.000195236	yes
Kpna3	chr14:61365185-61439947	71.3311	119.098	0.739541	0.000195236	yes

Nr4a2	chr2:57107225-57124003	1.12935	1.88491	0.739013	0.00338276	yes
Txnrd1	chr10:82833950-82897724	186.56	311.328	0.738796	0.000537301	yes
Ythdc1	chr5:86804489-86836657	39.2577	65.5119	0.738782	0.000195236	yes
Snx16	chr3:10417816-10440130	8.1751	13.6414	0.738685	0.000537301	yes
Fam8a1	chr13:46669521-46675773	9.93478	16.5618	0.737298	0.000537301	yes
H2-D1,H2-L	chr17:35263093-35267497	163.361	272.211	0.736659	0.000195236	yes
Slc2a6	chr2:27021364-27027998	14.5983	24.3035	0.735362	0.000370691	yes
Aff4	chr11:53350766-53421830	12.4008	20.6428	0.735202	0.000195236	yes
Zfp871	chr17:32765496-32788287	13.4887	22.449	0.734892	0.000195236	yes
Prpf3	chr3:95830621-95855753	21.6228	35.9601	0.733847	0.000195236	yes
Hgs	chr11:120467634-120483984	10.9186	18.1572	0.733751	0.000370691	yes
Fam188a	chr2:12347263-12419460	34.6995	57.7027	0.733724	0.000195236	yes
Dad1	chr14:54235484-54253929	179.451	298.41	0.733708	0.000195236	yes
Eno1	chr4:150237196-150248873	102.221	169.927	0.733223	0.000195236	yes
Cdc14a	chr3:116272552-116424032	3.82745	6.35791	0.73217	0.00100977	yes
Slc29a3	chr10:60712071-60752782	1.12702	1.87165	0.731793	0.00230418	yes
Pi4ka	chr16:17280350-17430826	11.9104	19.7724	0.731262	0.00859434	yes
Proser1	chr3:53463816-53481755	0.865152	1.4361	0.731126	0.0036408	yes
Mpp5	chr12:78748946-78840713	17.0073	28.2273	0.730935	0.000195236	yes
Ap3m2	chr8:22787353-22805654	2.00832	3.33309	0.730869	0.00130921	yes
Sash1	chr10:8722218-8886070	9.99885	16.5937	0.730802	0.000195236	yes
Nufip2	chr11:77686138-77717966	30.9388	51.3009	0.729567	0.000195236	yes
Atp6v0d1	chr8:105524469-105566040	35.9956	59.6792	0.729407	0.000195236	yes
Copa	chr1:172066012-172122332	82.8545	137.331	0.72901	0.000537301	yes
Maml1	chr11:50255634-50292336	2.62239	4.34621	0.728875	0.000370691	yes
Prpf40b	chr15:99295408-99317007	2.09512	3.47031	0.728028	0.00174394	yes
Coro7	chr16:4626883-4679720	2.92376	4.84054	0.727342	0.00216236	yes
Lmo4	chr3:144188529-144205255	50.2477	83.1821	0.727216	0.000195236	yes
Stat5a	chr11:100859350-100885169	5.13315	8.49744	0.727185	0.000370691	yes
Prkcd	chr14:30595353-30626208	33.3378	55.1714	0.726763	0.000195236	yes
Kmt2e	chr5:23434428-23616571	4.90275	8.11253	0.726562	0.0299428	yes
Btg1	chr10:96617000-96622811	19.3018	31.9249	0.725945	0.000195236	yes
Neat1	chr19:5824709-5845480	22.5622	37.3098	0.725648	0.00699437	yes
Asb1	chr1:91540564-91559590	3.11443	5.14923	0.725391	0.000698636	yes
Slc3a2	chr19:8706881-8723369	71.3181	117.903	0.725256	0.000195236	yes
Slc35d2	chr13:64096309-64129330	1.42232	2.35109	0.725091	0.00699437	yes
4833427F10Rik	chr17:35772449-35780687	0.994909	1.64421	0.724757	0.0300114	yes
Ripk1	chr13:34002873-34035170	27.2251	44.9814	0.724392	0.000195236	yes
Golga3	chr5:110176700-110223155	14.5031	23.9614	0.724345	0.000195236	yes
Ccni	chr5:93181932-93206495	29.1541	48.1654	0.724296	0.000195236	yes
Prkab1	chr5:116013589-116024428	16.0263	26.4688	0.72385	0.000195236	yes
Sf1	chr19:6363689-6378038	25.3185	41.8061	0.723519	0.000195236	yes
Fbxo33	chr12:59200654-59219483	26.7678	44.1673	0.722478	0.000195236	yes
Papolg	chr11:23862645-23895270	10.3716	17.1119	0.722366	0.000195236	yes
Necap1	chr6:122874556-122888941	22.3495	36.8698	0.722197	0.000370691	yes
Kat6a	chr8:22859538-22943262	7.31813	12.0687	0.721726	0.000195236	yes
Spata6	chr4:111720009-111829140	3.45362	5.69478	0.721529	0.00188078	yes
Magi3	chr3:104013264-104220406	1.11953	1.84578	0.721341	0.000698636	yes
Safb	chr17:56584981-56606294	26.3661	43.4546	0.720823	0.000195236	yes
Ehd4	chr2:120089486-120154575	77.5694	127.825	0.720611	0.000537301	yes
Zdhhc9	chrX:48171970-48208702	2.20929	3.63942	0.720128	0.00130921	yes
Swt1	chr1:151367698-151428435	14.6105	24.0678	0.720098	0.000195236	yes
Cd9	chr6:125460265-125494755	714.023	1175.8	0.719603	0.000855787	yes
Tmem98	chr11:80810414-80822033	4.83689	7.96347	0.719318	0.00216236	yes
Slc38a1	chr15:96571417-96642913	29.5003	48.4985	0.717213	0.000195236	yes
BC037034	chr5:138259657-138272754	7.09047	11.6548	0.716977	0.00311626	yes
Mar-05	chr19:37207544-37224457	35.7993	58.8432	0.716946	0.00230418	yes
H2-Q1	chr17:35320557-35325099	1.81222	2.97831	0.716733	0.00652015	yes
Tmem39a	chr16:38558697-38592162	14.1674	23.2805	0.716544	0.000537301	yes
Srcap,Tmem265	chr7:127510437-127565270	4.59514	7.54979	0.716325	0.00174394	yes
Ubn1	chr16:5050067-5086285	11.51	18.9035	0.715771	0.000195236	yes
Cecr2	chr6:120666420-120771191	0.506792	0.83221	0.715552	0.00202512	yes
Cdk11b	chr4:155624868-155649932	17.7294	29.1107	0.715411	0.000195236	yes
Wdr47	chr3:108591277-108645719	3.93502	6.45963	0.715081	0.000195236	yes
Bbs4	chr9:59321965-59353508	3.48436	5.71902	0.714876	0.000855787	yes

Wiz	chr17:32354049-32389439	1.74006	2.85601	0.714863	0.00116073	yes
8030462N17Rik	chr18:77633280-77714010	13.9251	22.8551	0.714829	0.000195236	yes
Has3	chr8:106870241-106882902	1.5771	2.58829	0.71473	0.00116073	yes
Caprin2	chr6:148842491-148896237	1.29364	2.12141	0.713584	0.0036408	yes
Cep170	chr1:176733652-176807124	21.584	35.3922	0.713473	0.000195236	yes
Efhd2	chr4:141858141-141874920	117.352	192.421	0.713426	0.000855787	yes
Ankhd1	chr18:36560602-36658908	17.2334	28.2562	0.71336	0.000370691	yes
Pnrc1	chr4:33245422-33248787	17.7885	29.1615	0.713122	0.000195236	yes
Stam2	chr2:52692205-52742149	18.6306	30.5399	0.713021	0.000195236	yes
Cd2ap	chr17:42792950-42876424	37.7734	61.9043	0.712668	0.000195236	yes
Rab3ip	chr10:116905783-116950380	8.90702	14.5954	0.712499	0.000370691	yes
Slc12a7	chr13:73763696-73816742	5.28751	8.66263	0.712217	0.000195236	yes
Mmp9	chr2:164948218-164955849	14.3679	23.539	0.71221	0.000195236	yes
Ndst1	chr18:60685975-60713389	1.69152	2.77117	0.712173	0.000855787	yes
Tlr2	chr3:83836271-83841608	58.7732	96.2801	0.712078	0.000195236	yes
Ints8	chr4:11191350-11254259	31.2495	51.1855	0.711902	0.0375213	yes
Arih1	chr9:59388553-59486374	29.491	48.2978	0.711681	0.000195236	yes
Fam98b	chr2:117249738-117271540	9.91707	16.2373	0.711329	0.000195236	yes
1110002L01Rik	chr12:3365131-3426747	8.39444	13.7436	0.711259	0.0200117	yes
Il7	chr3:7572076-7613427	0.606528	0.992935	0.711126	0.0451577	yes
Kdm2a	chr19:4316146-4397077	15.8306	25.9143	0.711031	0.000195236	yes
Fcho2	chr13:98723405-98815449	10.9781	17.9702	0.710975	0.000370691	yes
Jmjd1c	chr10:67127257-67256326	25.7419	42.1143	0.71019	0.000537301	yes
Lrba	chr3:86224689-86782694	9.10835	14.8989	0.709944	0.000370691	yes
Clcn5	chrX:7158411-7319358	4.28151	7.00183	0.709614	0.000195236	yes
Golga2	chr2:32288252-32307921	9.24578	15.1171	0.709314	0.000195236	yes
Smcr8	chr11:60777524-60788287	14.5838	23.8299	0.708409	0.000195236	yes
Orc1	chr4:108579453-108614831	10.6546	17.4084	0.708302	0.000195236	yes
Fam45a	chr19:60811569-60836229	13.7324	22.4249	0.707512	0.000195236	yes
Rsu1	chr2:13076966-13271415	34.1053	55.6916	0.707466	0.000195236	yes
Chd8	chr14:52198150-52237572	11.884	19.4	0.707031	0.000195236	yes
Akirin1	chr4:123735194-123750299	34.6817	56.5462	0.705257	0.000370691	yes
Mtmr9	chr14:63523609-63543953	13.3771	21.8104	0.705252	0.000195236	yes
Ap1s2	chrX:163909016-163933666	51.0801	83.1524	0.702996	0.000195236	yes
Nab1	chr1:52455848-52500448	35.0044	56.9577	0.702357	0.000195236	yes
Naip2	chr13:100144062-100202122	7.61582	12.3922	0.702356	0.000195236	yes
Dync1h1	chr12:110601394-110666944	21.7474	35.3506	0.700893	0.000195236	yes
Wdr7	chr18:63708694-63989759	4.6332	7.52692	0.700051	0.000855787	yes
Tle3	chr9:61372365-61418497	3.37358	5.47918	0.699679	0.000370691	yes
Slc8b1	chr5:120511191-120534024	4.67262	7.58613	0.699131	0.00100977	yes
Trrap	chr5:144768791-144859773	5.24717	8.51767	0.698919	0.000195236	yes
Rb1cc1	chr1:6214661-6276104	14.5506	23.6182	0.698823	0.000195236	yes
Gatsl2	chr5:134099747-134141758	1.23701	2.00763	0.698643	0.00298992	yes
Dhx9	chr1:153455757-153487660	65.0322	105.533	0.698464	0.000537301	yes
D17Wsu92e	chr17:27751231-27820542	17.0104	27.6002	0.698265	0.000195236	yes
Erlec1	chr11:30929783-30954131	4.23724	6.8745	0.69813	0.000195236	yes
Dnajc21	chr15:10446762-10470516	31.3738	50.9006	0.698123	0.000195236	yes
6430548M08Rik	chr8:120114151-120165307	8.99521	14.5919	0.697939	0.020214	yes
Exoc5	chr14:49012143-49087723	41.8204	67.8386	0.697899	0.000698636	yes
Lpl	chr8:68880554-68906932	226.187	366.751	0.697283	0.00202512	yes
Psmd2	chr16:20651651-20663414	98.2028	159.196	0.696967	0.000195236	yes
Dusp4	chr8:34807609-34819894	14.5577	23.5969	0.69681	0.000195236	yes
Sel1l	chr12:91806042-91849157	24.544	39.7803	0.696686	0.000195236	yes
Myef2	chr2:125068126-125123660	46.2597	74.9388	0.695957	0.000195236	yes
Atp11b	chr3:35754137-35856276	20.1247	32.6008	0.695942	0.000370691	yes
Hectd1	chr12:51743721-51829536	39.4049	63.786	0.694864	0.000370691	yes
Zcchc6	chr13:59769965-59823147	12.4299	20.1197	0.694798	0.000195236	yes
Kdm5c	chrX:152233226-152279099	6.95092	11.2498	0.694629	0.000195236	yes
Pak2	chr16:32016289-32079342	38.8251	62.8223	0.694287	0.000195236	yes
Brpf3	chr17:28801125-28838789	0.864372	1.39841	0.694061	0.00298992	yes
Tirap	chr9:35184390-35200291	5.37209	8.68645	0.693284	0.000370691	yes
Pcgf5	chr19:36379066-36456204	16.8667	27.2617	0.692692	0.00100977	yes
H60c	chr10:3256207-3267771	1.25875	2.03402	0.692346	0.0103944	yes
Rab1	chr11:20201601-20226856	76.7049	123.932	0.692159	0.000370691	yes
Ncdn	chr4:126743749-126753429	10.3767	16.7627	0.691907	0.000195236	yes

Naa15	chr3:51416015-51475985	18.4104	29.7373	0.691749	0.000195236	yes
Naa25	chr5:121397981-121440113	36.8126	59.4453	0.69136	0.000195236	yes
Sema4a	chr3:88435961-88461182	5.04908	8.15059	0.690885	0.00100977	yes
M6pr	chr6:122309009-122317677	256.375	413.647	0.690144	0.000855787	yes
Ncoa6	chr2:155390655-155440783	2.9048	4.68572	0.68983	0.00230418	yes
4833439L19Rik	chr13:54503804-54565382	22.06	35.5771	0.689516	0.00578321	yes
Rhbdd1	chr1:82316578-82445367	9.69579	15.6302	0.688906	0.000537301	yes
Golgb1	chr16:36880518-36933085	7.88415	12.7097	0.688901	0.000195236	yes
Tmem184c	chr8:77595977-77610653	39.776	64.0962	0.688341	0.000855787	yes
Ddit4	chr10:59949674-59951770	3.15136	5.07662	0.687892	0.00938869	yes
Crtc3	chr7:80586631-80688877	1.63663	2.63637	0.687821	0.00116073	yes
Slc7a1	chr5:148327409-148399904	15.7513	25.3723	0.687785	0.000195236	yes
Tcof1	chr18:60813755-60848964	18.913	30.4548	0.687292	0.000370691	yes
Lman2l	chr1:36423185-36445239	6.38745	10.2852	0.687252	0.00160026	yes
Dcaf5	chr12:80335846-80436601	5.5217	8.88985	0.687048	0.000537301	yes
Plbd2	chr5:120476546-120503623	7.17403	11.5497	0.687003	0.000195236	yes
Apc	chr18:34207774-34322190	5.62332	9.04578	0.685822	0.000195236	yes
Arfip1	chr3:84496092-84582625	30.9263	49.7392	0.68555	0.000195236	yes
Ddx24	chr12:103407975-103425867	50.751	81.62	0.685486	0.000370691	yes
Ankfy1	chr11:72690001-72772146	30.2192	48.5827	0.684979	0.000370691	yes
Setd1a	chr7:127777388-127800119	3.48595	5.602	0.684389	0.00100977	yes
Fktn	chr4:53714181-53763271	7.60421	12.2198	0.684351	0.000370691	yes
Slc25a43	chrX:36743631-36777307	4.67282	7.50527	0.683609	0.00428914	yes
Jazf1	chr6:52768067-53068624	0.592879	0.951672	0.682727	0.0339376	yes
Zfp703	chr8:26977335-26981462	0.567617	0.910987	0.682514	0.0291875	yes
Slc16a6	chr11:109440077-109573329	22.2486	35.6842	0.681571	0.000370691	yes
Clock	chr5:76183879-76469519	14.9649	23.9976	0.681312	0.0116945	yes
Slc30a6	chr17:74395607-74424229	17.5702	28.1679	0.68092	0.000195236	yes
Ifitm1	chr7:140967428-140969827	15.4373	24.7475	0.68086	0.00188078	yes
Rc3h1	chr1:160906410-160974976	13.1679	21.105	0.680552	0.000195236	yes
Foxo3	chr10:42185785-42276742	8.78888	14.078	0.679696	0.000195236	yes
Cers6	chr2:68861556-69111290	22.7744	36.4693	0.679273	0.000537301	yes
Tmf1	chr6:97151949-97179124	10.9318	17.4959	0.678483	0.000195236	yes
Eya4	chr10:23104167-23349903	0.291503	0.46641	0.678091	0.0493334	yes
Cep250	chr2:155956557-155998900	6.80244	10.8809	0.677673	0.000195236	yes
Cdk2ap2	chr19:4097350-4099017	47.5546	76.0571	0.677498	0.000370691	yes
Cd37	chr7:45233631-45239115	27.3952	43.8119	0.6774	0.000855787	yes
Wdfy1	chr1:79702261-79761769	6.57906	10.5215	0.677382	0.000855787	yes
Strn3	chr12:51608540-51738939	49.5709	79.2676	0.677238	0.00145427	yes
Tbcel	chr9:42412316-42472226	5.08309	8.12746	0.6771	0.00116073	yes
Clk2,Clk2-scamp3,Scamp	chr3:89164794-89182770	34.9877	55.9292	0.676755	0.000855787	yes
Iffo2	chr4:139530547-139620382	4.52024	7.22253	0.676103	0.000370691	yes
Kcnk6	chr7:29221927-29232522	9.57544	15.2979	0.675924	0.000370691	yes
Nop14	chr5:34638535-34660148	40.8539	65.245	0.675393	0.000698636	yes
Selt	chr3:58576657-58593546	43.5855	69.5694	0.674606	0.000537301	yes
Tbk1	chr10:121546455-121586794	35.6797	56.9484	0.674554	0.000195236	yes
Trem3	chr17:48247728-48258847	3.64951	5.82475	0.674494	0.00881201	yes
Sav1	chr12:69746847-69987002	24.1959	38.6127	0.67431	0.0176188	yes
Flnb	chr14:7817956-7951587	15.6737	25.0125	0.674307	0.000370691	yes
Aldoart1	chr4:72850582-72852634	11.3973	18.187	0.674222	0.000855787	yes
Tmco3	chr8:13255962-13322924	4.71822	7.52842	0.674103	0.0488815	yes
St7	chr6:17693993-17943023	12.4275	19.8281	0.674007	0.000537301	yes
Camsap1	chr2:25926837-25983282	5.11959	8.16824	0.673997	0.000195236	yes
Capn2	chr1:182467258-182517483	35.8514	57.1475	0.672661	0.00100977	yes
Mfhas1	chr8:35582502-35679449	1.90888	3.04274	0.672644	0.00188078	yes
Gpr85	chr6:13835073-13839848	0.616605	0.982521	0.672141	0.0248334	yes
Smg1	chr7:118131311-118243637	17.9691	28.6167	0.671341	0.000698636	yes
Sec16a	chr2:26409430-26445216	8.37669	13.3398	0.671279	0.000855787	yes
Cdc40	chr10:40831621-40883143	17.4249	27.7461	0.671135	0.000195236	yes
Manea	chr4:26324505-26346652	12.5569	19.9934	0.67105	0.000370691	yes
Arf3	chr15:98737625-98763118	30.8343	49.0888	0.670859	0.000195236	yes
Cdyl2	chr8:116568723-116732991	1.42356	2.26629	0.670824	0.00160026	yes
Cep350	chr1:155844963-155973255	13.1962	21.0073	0.670765	0.000370691	yes
Srrm1	chr4:135320483-135353214	17.9141	28.5008	0.669905	0.000537301	yes
Zc3h7a	chr16:11136593-11176393	26.631	42.361	0.669632	0.000370691	yes

Rrbp1	chr2:143947394-144011263	17.0412	27.0871	0.668582	0.000698636	yes
Usp9x	chrX:13071497-13173327	41.4943	65.9419	0.668281	0.000195236	yes
Rb1	chr14:73195501-73325791	7.97266	12.6692	0.668191	0.00639665	yes
Zbtb6	chr2:37425499-37430919	20.2265	32.1399	0.668113	0.000195236	yes
Sugp1	chr8:70042812-70071953	14.3427	22.7894	0.668042	0.000855787	yes
Cep95	chr11:106789251-106818861	5.28817	8.40093	0.667781	0.00130921	yes
Pdpk1	chr17:24073679-24150922	22.1115	35.0979	0.666587	0.00298992	yes
Ppp4r2	chr6:100833637-100868717	16.7999	26.6626	0.666363	0.000195236	yes
Polr3c	chr3:96711894-96727439	22.4427	35.6009	0.665668	0.000195236	yes
4930526I15Rik	chr9:124423250-124424856	8.29063	13.1502	0.665526	0.00870444	yes
Ccdc169,Spg20	chr3:55112107-55172936	9.29317	14.7339	0.6649	0.000698636	yes
Kdm5a	chr6:120324321-120444574	16.0504	25.4314	0.664	0.00174394	yes
Slc30a1	chr1:191894071-191913247	13.877	21.9836	0.663727	0.000855787	yes
Acbd4	chr11:103101687-103112199	6.64518	10.525	0.663438	0.00145427	yes
Arpc5	chr1:152766541-152775580	356.803	565.112	0.663412	0.00160026	yes
9430020K01Rik	chr18:4634928-4682869	0.579042	0.916883	0.663069	0.00602767	yes
Mfap1a	chr2:121492680-121506656	2.20727	3.495	0.663026	0.00174394	yes
Wdr11	chr7:129591862-129635738	16.7523	26.5251	0.663001	0.000195236	yes
Etv6	chr6:134035699-134270147	8.06134	12.7629	0.662869	0.000537301	yes
Tln1	chr4:43531512-43562583	57.8565	91.5898	0.662707	0.00160026	yes
Polk	chr13:96480688-96542485	8.94858	14.1652	0.662616	0.000537301	yes
Sfmbt1	chr14:30714848-30822721	5.67765	8.98701	0.662549	0.000537301	yes
Ccrn4l	chr3:51224446-51251654	3.5383	5.6005	0.662499	0.00351242	yes
Ap2m1	chr16:20535479-20544909	16.7326	26.4636	0.661344	0.00116073	yes
Ubr4	chr4:139380658-139489532	7.20643	11.3965	0.661232	0.000370691	yes
Map4k4	chr1:39900912-40026310	51.9766	82.168	0.660715	0.00174394	yes
Ddx18	chr1:121553834-121567980	21.1018	33.3518	0.660398	0.000195236	yes
Slc36a4	chr9:15709768-15738789	17.6854	27.9426	0.659902	0.00100977	yes
Psme3	chr11:101316250-101323530	84.784	133.927	0.659585	0.000195236	yes
Bdp1	chr13:100017993-100104070	21.6925	34.2533	0.659043	0.00160026	yes
Praf2	chrX:7728570-7731063	15.2057	24.0091	0.658976	0.00130921	yes
Nus1	chr10:52417546-52440192	30.1793	47.6518	0.658974	0.000698636	yes
Rbm26	chr14:105106727-105177327	28.6833	45.2824	0.658737	0.00130921	yes
Tubb6	chr18:67390730-67402749	128.176	202.343	0.658677	0.000537301	yes
Azin1	chr15:38487429-38519266	81.5323	128.658	0.658096	0.000195236	yes
Foxj3	chr4:119539660-119629119	11.8185	18.6486	0.65802	0.000370691	yes
Txndc11	chr16:11066297-11134532	10.5946	16.7116	0.657519	0.000855787	yes
Ccdc92	chr5:124834431-124862221	2.23818	3.52953	0.65715	0.00836289	yes
Smim14	chr5:65448754-65492835	10.3799	16.3671	0.65701	0.00188078	yes
Cdk14	chr5:4803384-5380251	29.3226	46.2346	0.656959	0.000698636	yes
Myof	chr19:37899035-38043577	30.3419	47.8415	0.65695	0.000195236	yes
Gpr35	chr1:92973118-92986391	0.297566	0.469175	0.656915	0.0404912	yes
Stxbp1	chr2:32787606-32847237	13.2215	20.8422	0.656618	0.000537301	yes
Twf1	chr15:94577947-94589824	23.2909	36.7095	0.656386	0.000370691	yes
Prdm9	chr17:15543078-15563323	1.16062	1.82841	0.655698	0.00553759	yes
Blvrb	chr7:27447977-27465981	74.1741	116.835	0.655484	0.000537301	yes
Ripk3	chr14:55784994-55788857	9.61119	15.138	0.655387	0.00230418	yes
Xylt1	chr7:117380978-117667630	1.59905	2.51838	0.65528	0.00479652	yes
Met	chr6:17463956-17573980	2.62038	4.12663	0.655189	0.00116073	yes
Sec62	chr3:30792875-30821263	36.3033	57.1669	0.65508	0.00100977	yes
2410089E03Rik	chr15:8169105-8271158	2.00291	3.15296	0.654613	0.000698636	yes
Egln3	chr12:54178980-54203874	1.25047	1.96818	0.654389	0.011374	yes
Ccdc88a	chr11:29374171-29510808	17.8739	28.125	0.653997	0.000537301	yes
Rpl7a	chr2:26910806-26913311	211.805	333.273	0.653967	0.00338276	yes
Gng10	chr4:59035155-59041899	120.148	189.043	0.653902	0.000698636	yes
Pdzd8	chr19:59296083-59345780	31.768	49.9639	0.653315	0.000537301	yes
Pde3b	chr7:114415253-114537937	4.61393	7.25552	0.653082	0.000855787	yes
Qpctl	chr7:19140216-19149196	2.81606	4.42639	0.652454	0.00403597	yes
Helq	chr5:100762147-100798600	4.7017	7.38969	0.65233	0.00188078	yes
Hsf2	chr10:57486384-57513143	15.5052	24.364	0.652002	0.000370691	yes
Elovl4	chr9:83778691-83806305	1.56775	2.46337	0.65194	0.00973453	yes
Fbxo28	chr1:182313101-182341606	9.51344	14.9474	0.651853	0.00100977	yes
Bptf	chr11:107033080-107131922	9.37972	14.7365	0.651773	0.00174394	yes
Zfp292	chr4:34803109-34882948	14.9523	23.4896	0.651653	0.000195236	yes
Cdc42bpb	chr12:111292971-111377718	9.32808	14.6493	0.651185	0.000370691	yes

Aftph	chr11:20685084-20741556	35.2131	55.2912	0.650938	0.000370691	yes
Rasal3	chr17:32390660-32404808	1.7407	2.73323	0.650935	0.00529192	yes
Rhog	chr7:102239122-102250118	31.9766	50.2085	0.650915	0.00100977	yes
Mdm2	chr10:117688874-117710758	19.2349	30.2014	0.65089	0.000195236	yes
Dlg1	chr16:31663442-31873356	19.9657	31.3486	0.650874	0.000537301	yes
Acadl	chr1:66830838-66863309	42.358	66.5022	0.65077	0.000537301	yes
Itpripl2	chr7:118485111-118491975	13.1024	20.5644	0.650314	0.000195236	yes
Arl13b	chr16:62793688-62847040	5.26607	8.26266	0.649879	0.00130921	yes
Noc3l	chr19:38788127-38819237	29.8348	46.8048	0.649659	0.000370691	yes
Nploc4	chr11:120379797-120437700	25.4041	39.8491	0.649482	0.000195236	yes
Nat2	chr8:67494874-67502578	7.48955	11.7473	0.649379	0.00428914	yes
Isca2	chr12:84773269-84775089	25.6345	40.1934	0.648871	0.000855787	yes
Nceh1	chr3:27183003-27244911	11.4668	17.9787	0.648821	0.000537301	yes
Tvp23b	chr11:62879489-62895184	20.7029	32.4548	0.648598	0.00145427	yes
Gtf3c6	chr10:40249202-40257665	30.7751	48.224	0.64799	0.00174394	yes
Meis3	chr7:16175394-16186508	1.62889	2.5516	0.64751	0.0103944	yes
Rit1	chr3:88716853-88731048	8.60362	13.4766	0.647444	0.00116073	yes
Hsd17b7	chr1:169949536-169969205	13.0032	20.3663	0.647322	0.000537301	yes
Gnl3l	chrX:150983132-151017322	22.6216	35.4256	0.647092	0.000370691	yes
Slc41a1	chr1:131828011-131848864	4.48292	7.02014	0.647063	0.00116073	yes
Wapal	chr14:34673927-34747983	74.557	116.735	0.646824	0.000855787	yes
Cpne8	chr15:90487480-90679388	31.6036	49.4773	0.646679	0.000195236	yes
Ube4b	chr4:149328415-149426631	15.1278	23.6763	0.646245	0.000195236	yes
Grn	chr11:102430321-102436809	45.6741	71.4695	0.645951	0.00100977	yes
Plxna1	chr6:89316313-89362613	1.95342	3.0566	0.645926	0.00174394	yes
Gm13139	chr4:145781542-145831978	0.933176	1.4598	0.645555	0.0132116	yes
C1galt1c1	chrX:38630782-38635143	21.4356	33.5325	0.64555	0.00258324	yes
Serinc1	chr10:57515774-57532529	98.2148	153.624	0.645393	0.000195236	yes
Rnf183	chr4:62427541-62434726	1.69572	2.65201	0.645187	0.0172208	yes
Tmem189	chr2:167643224-167661544	23.2756	36.3968	0.644996	0.00130921	yes
Map7d1	chr4:126232167-126256319	16.5525	25.865	0.643949	0.00100977	yes
Dip2b	chr15:100038663-100219473	6.59653	10.3072	0.643868	0.00130921	yes
Notch2	chr3:98013537-98150367	9.16292	14.3128	0.64343	0.000698636	yes
Rcor1	chr12:111039797-111113386	11.9672	18.6927	0.64339	0.000698636	yes
Dhx29	chr13:112927792-112969187	21.8609	34.146	0.643363	0.000855787	yes
Neu3	chr7:99811438-99828417	0.593587	0.927065	0.643211	0.0323114	yes
Wdfy3	chr5:101832952-102069921	7.80738	12.1874	0.642482	0.000370691	yes
BC031181	chr18:75005899-75009933	62.9874	98.2156	0.64089	0.000537301	yes
Gorab	chr1:163384908-163403641	8.42603	13.1381	0.640828	0.000537301	yes
Kif3b	chr2:153291415-153333389	6.61172	10.3089	0.640799	0.000698636	yes
Snx33	chr9:56917199-56928371	1.51978	2.36919	0.640535	0.00390381	yes
Dhrsx	chr4_GL456216_random:15880	35.0136	54.5748	0.640319	0.00130921	yes
Aldoart2	chr12:55565204-55566896	3.13484	4.88353	0.639535	0.00847587	yes
Irgq	chr7:24530647-24538600	1.42076	2.21312	0.639414	0.0036408	yes
Iqgap1	chr7:80711582-80803331	90.7854	141.406	0.639313	0.00351242	yes
Cdk17	chr10:93160875-93241342	23.7163	36.9358	0.639142	0.000537301	yes
Sertad1	chr7:27486952-27490314	9.59015	14.9348	0.639053	0.00390381	yes
Kcnab1	chr3:65109367-65378225	0.776174	1.2087	0.639	0.0369734	yes
Mtus2	chr5:147957319-148316065	1.73122	2.69572	0.638888	0.00285786	yes
6820431F20Rik	chr8:20268285-20297432	0.598906	0.932487	0.638753	0.0199123	yes
Cirh1a	chr8:106893639-106923094	69.3968	108.04	0.638631	0.00116073	yes
Map2k3	chr11:60932056-60952803	28.803	44.8401	0.638569	0.000537301	yes
Heatr5b	chr17:78752905-78835381	7.8096	12.1567	0.638433	0.000537301	yes
Max	chr12:76937268-76962248	59.2976	92.2844	0.638114	0.000195236	yes
Psmd1	chr1:86064618-86139295	154.283	240.097	0.638039	0.00160026	yes
Rpe	chr1:66700892-66817595	69.5067	108.14	0.637676	0.00311626	yes
Sypl	chr12:32953944-32979502	21.9326	34.1223	0.637634	0.000370691	yes
Ccdc59	chr10:105841478-105847510	31.6884	49.2955	0.637499	0.00145427	yes
Mcl1	chr3:95658720-95663178	147.962	230.165	0.63744	0.000855787	yes
Apaf1	chr10:90989310-91082743	13.5112	21.0099	0.636916	0.000537301	yes
D16Ertd472e	chr16:78540335-78576688	6.30041	9.79416	0.636475	0.000698636	yes
Rnf38	chr4:44126211-44168283	9.76331	15.1771	0.636454	0.00130921	yes
Hoxc4	chr15:103034394-103036852	2.21406	3.44175	0.636448	0.0103944	yes
Trak1	chr9:121366957-121474918	3.57593	5.5586	0.636405	0.00116073	yes
Akap13	chr7:75455533-75754609	11.3128	17.5779	0.635809	0.000698636	yes

Wdr37	chr13:8802965-8871736	8.12444	12.621	0.635489	0.00202512	yes
Rlim	chrX:103957166-103981284	73.0543	113.472	0.635289	0.00230418	yes
Akap7	chr10:25169089-25299163	5.60183	8.70072	0.635237	0.00145427	yes
Rab2a	chr4:8535643-8607702	116.975	181.661	0.635055	0.00174394	yes
Dnajc18	chr18:35671104-35703144	4.49814	6.98152	0.634215	0.00202512	yes
Taok1	chr11:77529161-77607815	26.5631	41.2263	0.634144	0.00100977	yes
Tmem159	chr7:120102425-120120986	20.9117	32.4498	0.633901	0.00145427	yes
Nisch	chr14:31170927-31206826	14.8686	23.0692	0.633692	0.000855787	yes
Rab33b	chr3:51483965-51496212	4.4288	6.86723	0.632812	0.00271906	yes
Ribc1	chrX:152004583-152016295	1.70342	2.64086	0.63257	0.0149322	yes
Mboat7	chr7:3677788-3693525	10.5254	16.3172	0.632524	0.00298992	yes
Cers2	chr3:95315251-95357202	85.3846	132.367	0.6325	0.0152609	yes
Golim4	chr3:75876182-75956949	21.3792	33.1274	0.631817	0.00116073	yes
Tob2	chr15:81848269-81858326	7.02505	10.8812	0.631252	0.00174394	yes
Zfp719	chr7:43579585-43593710	4.63633	7.18097	0.631196	0.00188078	yes
Ttc17	chr2:94300765-94406689	10.5247	16.2995	0.631057	0.00130921	yes
Fcer1g	chr1:171229571-171234349	364.956	565.144	0.630898	0.00100977	yes
Gns	chr10:121365089-121397245	29.0713	45.0145	0.630794	0.000855787	yes
Dcakd	chr11:102994055-103017147	9.76689	15.1185	0.630344	0.00403597	yes
Morc2a	chr11:3649493-3690372	11.7105	18.1251	0.630186	0.00145427	yes
Tlk2	chr11:105178806-105283959	16.2318	25.1179	0.629887	0.00174394	yes
Psmd7	chr8:107580379-107588482	92.0419	142.402	0.62961	0.00130921	yes
Rprd2	chr3:95759872-95818953	3.65594	5.6558	0.629487	0.00160026	yes
Vps26a	chr10:62454842-62486805	81.8277	126.539	0.628916	0.00130921	yes
Samhd1	chr2:157097528-157135222	70.0521	108.316	0.628746	0.00100977	yes
Megf11	chr9:64385625-64709205	0.340791	0.526883	0.628597	0.0406913	yes
Kras	chr6:145216698-145250231	15.1174	23.3715	0.628538	0.00130921	yes
Mfsd9	chr1:40772039-40790657	0.962376	1.48771	0.628419	0.0217808	yes
Ttbk2	chr2:120732816-120850584	3.79618	5.86759	0.628219	0.000370691	yes
Cab39l	chr14:59440980-59548903	27.1621	41.975	0.627935	0.000855787	yes
Ltb	chr17:35194506-35196305	12.3529	19.0891	0.627898	0.0036408	yes
Tulp3	chr6:128321160-128355851	2.89725	4.47597	0.627515	0.00479652	yes
Mfsd2a	chr4:122946850-122961188	2.26282	3.49581	0.627503	0.0122489	yes
Nudt7	chr8:114133573-114152312	4.76392	7.35788	0.627141	0.0133195	yes
Arhgdib	chr6:136923660-136941899	350.429	540.866	0.62615	0.00578321	yes
Ankrd50	chr3:38449260-38484816	2.07819	3.207	0.625899	0.00566351	yes
Slc12a4	chr8:105943589-105966115	13.8774	21.4148	0.625876	0.00188078	yes
Man1a	chr10:53906032-54075796	6.77902	10.4591	0.625609	0.00174394	yes
Fam193a	chr5:34369932-34486458	4.6841	7.22633	0.625491	0.00188078	yes
Nol10	chr12:17348492-17430095	14.5391	22.4252	0.625181	0.000537301	yes
Gigyf2	chr1:87326997-87450810	23.6359	36.4513	0.62499	0.00145427	yes
Stau1	chr2:166947548-166996299	7.21589	11.1273	0.624849	0.00100977	yes
Ccser2	chr14:36874935-36968764	7.17216	11.0593	0.624783	0.00160026	yes
Gspt1	chr16:11203382-11254325	88.1729	135.946	0.624631	0.00517183	yes
Esco1	chr18:10566511-10610352	16.3917	25.2712	0.624533	0.000370691	yes
Faf2	chr13:54621783-54664063	16.7336	25.798	0.624512	0.000855787	yes
Ttc14	chr3:33800182-33844310	15.39	23.7197	0.624098	0.00145427	yes
Hmbox1	chr14:64822217-64949847	3.21911	4.96094	0.62395	0.00479652	yes
Tmbim4	chr10:120208825-120224897	78.8197	121.442	0.623641	0.00160026	yes
Vegfa	chr17:46016992-46032377	7.43576	11.456	0.623555	0.00216236	yes
Fth1	chr19:9980599-9985111	2907.87	4478.61	0.62309	0.0296495	yes
Scamp1	chr13:94201432-94285281	13.3406	20.5375	0.622432	0.00244513	yes
Ptbp2	chr3:119718741-119783388	13.8278	21.2772	0.621739	0.00188078	yes
Rnf103	chr6:71493876-71510880	7.80638	12.0099	0.621496	0.00230418	yes
Ubl7	chr9:57910985-57929968	20.7587	31.9264	0.621032	0.00216236	yes
Rrp12	chr19:41862850-41896153	17.2063	26.4625	0.621008	0.00130921	yes
Hspa8	chr9:40801272-40805199	307.135	472.324	0.620904	0.0116945	yes
Sfpq	chr4:127021300-127037014	109.776	168.701	0.619907	0.00244513	yes
Ccdc47	chr11:106199355-106216367	63.633	97.7838	0.619822	0.00130921	yes
Fbxw7	chr3:84815576-84979198	10.79	16.5788	0.619648	0.00116073	yes
Tbc1d22b	chr17:29549801-29606808	4.38776	6.73933	0.61912	0.00174394	yes
Tpm4	chr8:72135291-72153129	154.842	237.789	0.618882	0.00216236	yes
Chd1	chr17:15704966-15772612	35.1684	53.9856	0.618297	0.00258324	yes
Mcur1	chr13:43538405-43560191	7.9262	12.1657	0.618114	0.00216236	yes
Psmf1	chr2:151716061-151741310	9.09381	13.9561	0.617943	0.00202512	yes

Psma4	chr9:54950256-54958030	209.027	320.777	0.61788	0.00145427	yes
Cryzl1	chr16:91688897-91728802	24.7562	37.9899	0.617827	0.00100977	yes
Gm9833	chr3:10088276-10092562	38.846	59.6108	0.617806	0.00174394	yes
A230028O05Rik	chr16:25059638-25069058	0.860908	1.3207	0.617375	0.0314474	yes
Pcbp4	chr9:106453837-106465940	1.18966	1.82474	0.617144	0.0249403	yes
Rbm5	chr9:107740494-107771002	36.1304	55.411	0.616961	0.00202512	yes
Slc9a4	chr1:40580226-40630731	12.8704	19.7332	0.616572	0.000537301	yes
Blzf1	chr1:164289799-164307484	12.0683	18.4993	0.616245	0.000855787	yes
Inpp1	chr1:52789419-52817688	5.72256	8.7713	0.61613	0.00627268	yes
Cherp	chr8:72443879-72475233	5.14447	7.88425	0.615949	0.0185753	yes
Rnu12	chr15:83149644-83149794	103.904	159.219	0.615758	0.028985	yes
Tmem104	chr11:115187486-115247025	0.563947	0.864071	0.615591	0.0217808	yes
Zcchc11	chr4:108459425-108559415	11.119	17.0344	0.615423	0.00116073	yes
Bmp2k	chr5:96997688-97111596	6.81303	10.4307	0.614467	0.000698636	yes
Ints6	chr14:62663666-62830126	13.4685	20.6178	0.614305	0.00130921	yes
Sbno1	chr5:124368701-124425914	35.0169	53.5978	0.614125	0.00145427	yes
Dhrs11	chr11:84820727-84829003	2.87981	4.4078	0.614085	0.00778355	yes
Capn7	chr14:31336723-31371983	32.4643	49.6876	0.614031	0.00100977	yes
Hexim1	chr11:103116324-103119724	16.4095	25.113	0.613901	0.00160026	yes
Dmwd	chr7:19076199-19082775	2.59208	3.96628	0.613679	0.0101798	yes
Ep300	chr15:81586213-81652077	5.381	8.23172	0.61332	0.00100977	yes
Zcrb1	chr15:93386112-93398290	51.8236	79.2644	0.613064	0.00145427	yes
Gbe1	chr16:70313948-70569720	11.4274	17.4772	0.612981	0.00160026	yes
Acta2	chr19:34240335-34255373	0.76573	1.17103	0.612864	0.0398967	yes
Sec23a	chr12:58958383-59012017	20.746	31.723	0.612698	0.00116073	yes
Elmod2	chr8:83312631-83332486	11.4405	17.4879	0.612208	0.00202512	yes
Ogdh	chr11:6291596-6359094	22.7922	34.8222	0.611468	0.00100977	yes
Atg4a	chr3:103575281-103646068	7.17376	10.9601	0.611455	0.00441531	yes
Dsel	chr1:111858701-111864918	4.08124	6.235	0.611382	0.00216236	yes
Trak2	chr1:58900449-58973482	21.9082	33.4621	0.611055	0.00116073	yes
Eps15l1	chr8:72340995-72421474	12.3767	18.9037	0.611042	0.00202512	yes
Skap2	chr6:51859164-52012549	150.34	229.589	0.610829	0.00145427	yes
Hspbap1	chr16:35770385-35828462	1.64259	2.50811	0.610626	0.00893132	yes
Spcs3	chr8:54520432-54529998	209.967	320.457	0.609971	0.00271906	yes
Otud5	chrX:7841830-7876626	19.8937	30.3571	0.609722	0.00116073	yes
Azi2	chr9:118040521-118150196	23.0516	35.1359	0.608078	0.00627268	yes
Rars	chr11:35808380-35834528	193.235	294.508	0.607953	0.00271906	yes
Pea15a	chr1:172196728-172206781	11.7776	17.9482	0.607793	0.00188078	yes
Ufl1	chr4:25248585-25281821	15.8328	24.1233	0.607506	0.00130921	yes
Mina	chr16:59471774-59555752	34.0036	51.8068	0.607453	0.0378013	yes
Pogz	chr3:94837566-94883567	3.81876	5.81731	0.607246	0.00298992	yes
Rbm4b	chr19:4756524-4765941	5.49596	8.37224	0.607243	0.00566351	yes
Fryl	chr5:73020190-73256618	5.27377	8.03332	0.60716	0.00188078	yes
Fut8	chr12:77238103-77475996	6.27612	9.55907	0.606998	0.00311626	yes
Zfp513	chr5:31198980-31202303	6.28266	9.56359	0.606178	0.00441531	yes
Pitpnc1	chr11:107207891-107470720	2.42769	3.69483	0.605924	0.00377278	yes
Slc25a40	chr5:8422837-8454839	7.12898	10.8496	0.605879	0.00504708	yes
Usp10	chr8:119910851-119957557	28.7821	43.7954	0.605606	0.00188078	yes
Snupn	chr9:56950923-56983199	11.8062	17.9592	0.605179	0.00311626	yes
Fmnl2	chr2:52857867-53134202	1.60946	2.44818	0.605132	0.00675438	yes
Ptpn21	chr12:98676740-98737405	1.22745	1.86684	0.604937	0.0109347	yes
Smim10l1	chr6:133105238-133110899	40.622	61.7788	0.60485	0.00100977	yes
Ubap2l	chr3:89998759-90068347	37.2983	56.7237	0.604845	0.0348164	yes
Shoc2	chr19:53892230-54033278	25.7773	39.1979	0.604672	0.0110431	yes
Gdpgp1	chr7:80232892-80241420	3.26388	4.96311	0.604656	0.00492649	yes
Tmem57	chr4:134802759-134853345	14.8515	22.5819	0.604558	0.00100977	yes
E130308A19Rik	chr4:59626210-59754303	0.962805	1.46395	0.604549	0.0207877	yes
Kif5b	chr18:6201004-6241524	84.1	127.868	0.604476	0.00416625	yes
Pigs	chr11:78328421-78342776	18.2658	27.7705	0.60441	0.00100977	yes
Smurf1	chr5:144876494-144965830	2.61744	3.97926	0.604345	0.00467182	yes
Ube2d1	chr10:71254979-71285262	15.9949	24.3072	0.603771	0.00311626	yes
Morc3	chr16:93832120-93876073	63.4146	96.3636	0.603673	0.00160026	yes
Fam20b	chr1:156678570-156718910	34.4896	52.3889	0.603098	0.000855787	yes
Tmem248	chr5:130219743-130243765	23.5986	35.8382	0.602795	0.000855787	yes
Rbm18	chr2:36116078-36190283	10.6781	16.2125	0.602457	0.045765	yes

Arid4b	chr13:14063783-14199603	10.0215	15.2144	0.602339	0.00100977	yes
Psmd10	chrX:140948424-140956711	37.6552	57.1569	0.602078	0.00258324	yes
Zrsr2	chrX:163935442-163958666	12.1276	18.3987	0.601312	0.00311626	yes
Pigt	chr2:164497524-164508301	25.1225	38.102	0.600886	0.00145427	yes
Rbms2	chr10:128129469-128180297	1.45387	2.20451	0.600558	0.00859434	yes
Adam17	chr12:21316391-21373632	58.3668	88.4798	0.600201	0.00271906	yes
Mier3	chr13:111686177-111718594	11.7443	17.798	0.599752	0.00100977	yes
Atp6v0c	chr17:24163864-24169429	5.87384	8.90091	0.599649	0.0148245	yes
Cep290	chr10:100488288-100589259	8.46018	12.8182	0.599432	0.011374	yes
Chn1	chr2:73596525-73775346	0.530856	0.803903	0.598702	0.0392619	yes
Bloc1s6	chr2:122738504-122749487	20.9908	31.7774	0.598242	0.00145427	yes
Ugcg	chr4:59189549-59222833	14.7018	22.252	0.59794	0.00100977	yes
Unc13b	chr4:43058983-43264887	0.565781	0.85633	0.597921	0.0153646	yes
Snrpd3	chr10:75518041-75535440	79.7547	120.669	0.597416	0.00244513	yes
Rpgrip1l	chr8:91217029-91313222	2.78132	4.20773	0.597271	0.00479652	yes
Cpne2	chr8:94533027-94570529	5.18765	7.84679	0.597022	0.00529192	yes
Ric1	chr19:29522281-29605921	9.97577	15.0763	0.59578	0.00145427	yes
Smad4	chr18:73639012-73703741	24.5359	37.0781	0.595674	0.00145427	yes
Sdccag3	chr2:26382799-26389316	24.4646	36.9663	0.595511	0.00130921	yes
Msl2	chr9:101074761-101104799	30.3478	45.8509	0.595359	0.00130921	yes
Gdap2	chr3:100162462-100206989	14.9945	22.6525	0.595233	0.00285786	yes
Gnb1	chr4:155491360-155559269	88.698	133.959	0.594814	0.00174394	yes
Mcc	chr18:44425059-44812182	0.3774	0.569857	0.594507	0.027792	yes
Dvl3	chr16:20517063-20532187	1.67518	2.52905	0.594275	0.0163026	yes
Galc	chr12:98202299-98259459	3.1173	4.70324	0.593357	0.00541779	yes
Itsn2	chr12:4593007-4738383	47.5838	71.7811	0.593135	0.0123537	yes
Slc30a4	chr2:122681232-122702663	7.62193	11.4975	0.593089	0.00160026	yes
Thra	chr11:98741872-98765111	2.54465	3.83852	0.593086	0.00973453	yes
Timm17a	chr1:135301534-135313737	142.788	215.388	0.593061	0.00216236	yes
Itgav	chr2:83724396-83806917	20.2417	30.5326	0.593018	0.00145427	yes
Coa5	chr1:37417084-37430103	65.9952	99.5403	0.59292	0.00174394	yes
Emilin2	chr17:71252175-71310965	70.6669	106.586	0.59291	0.00467182	yes
Polb	chr8:22628118-22653437	28.0762	42.3305	0.592349	0.00298992	yes
Cyld	chr8:88697027-88751946	11.4074	17.1984	0.592306	0.00100977	yes
Slc25a24	chr3:109123148-109168409	23.0963	34.8051	0.591638	0.00174394	yes
Zfp827	chr8:79028436-79193766	1.55987	2.34992	0.591182	0.00566351	yes
9930021J03Rik	chr19:29714401-29806009	5.00689	7.53894	0.590447	0.00311626	yes
Znrf2	chr6:54816915-54890224	64.7673	97.5122	0.590318	0.00130921	yes
Prepl	chr17:85028346-85090274	13.4965	20.3094	0.589561	0.00145427	yes
Zfp935	chr13:62453015-62466812	3.5155	5.28956	0.589416	0.00778355	yes
Ube2d3	chr3:135438758-135467178	289.593	435.564	0.58886	0.00602767	yes
Tet3	chr6:83362373-83441678	5.78772	8.70399	0.588682	0.00174394	yes
Nfatc3	chr8:106059602-106130537	15.846	23.8241	0.588301	0.00202512	yes
Lsm14b	chr2:180024986-180035461	15.0885	22.6629	0.586879	0.00298992	yes
Nmd3	chr3:69722054-69749046	67.077	100.738	0.586712	0.00298992	yes
Slx4	chr16:3979105-4001678	4.53461	6.80735	0.586114	0.00202512	yes
Cnnm2	chr19:46761608-46878580	2.45034	3.67789	0.585895	0.0104999	yes
Prkar2a	chr9:108692142-108749511	46.2627	69.4242	0.585589	0.00311626	yes
Ik	chr18:36744655-36757639	137.41	206.204	0.585585	0.00687627	yes
Lats1	chr10:7681208-7716461	14.3859	21.5873	0.585529	0.00116073	yes
Wrnip1	chr13:32802029-32822610	15.364	23.0548	0.585511	0.00216236	yes
Dynlt3	chrX:9654269-9662983	55.4497	83.2018	0.585434	0.00271906	yes
Ubxn11	chr4:134102582-134126780	1.21423	1.82122	0.584867	0.0497577	yes
Msantd4	chr9:4383536-4386869	29.6707	44.5026	0.584851	0.00160026	yes
Nphp4	chr4:152478141-152563184	0.396273	0.594196	0.584444	0.0433027	yes
Sars	chr3:108424863-108445259	86.8313	130.151	0.583897	0.00553759	yes
Abhd6	chr14:8002901-8056555	2.74715	4.11602	0.583315	0.0151569	yes
Arc	chr15:74669080-74672570	1.12859	1.69083	0.583205	0.0183683	yes
Gng2	chr14:19872558-19977249	10.6195	15.9087	0.583102	0.00230418	yes
9230114K14Rik	chr5:52190680-52197984	0.830179	1.24343	0.582828	0.0344621	yes
Ammecr1l	chr18:31759823-31784083	9.0048	13.4764	0.581671	0.00271906	yes
Cd200r1	chr16:44765735-44794977	1.1439	1.71159	0.581373	0.0448304	yes
Stt3a	chr9:36732412-36767578	183.033	273.744	0.580725	0.00390381	yes
Casc3	chr11:98795768-98833807	9.66366	14.453	0.580723	0.0381645	yes
Ube2r2	chr4:41136020-41193370	18.3997	27.512	0.580378	0.00216236	yes

Pld4	chr12:112760654-112768986	51.9218	77.6315	0.580302	0.00160026	yes
Nsfl1c	chr2:151494181-151511310	30.6401	45.7995	0.579914	0.00298992	yes
Ikzf5	chr7:131388648-131410478	9.68157	14.4636	0.579116	0.00271906	yes
Abcf1	chr17:35956818-35969750	56.7155	84.6674	0.578064	0.00244513	yes
Huwe1	chrX:151803281-151935417	26.4789	39.5205	0.577757	0.00517183	yes
Bcl2l2	chr14:54883424-54888234	6.46335	9.64579	0.577616	0.00553759	yes
Prpf38b	chr3:108902806-108911704	59.6297	88.987	0.577564	0.00627268	yes
Ascc2	chr11:4637792-4683386	8.95004	13.3562	0.577542	0.00416625	yes
4930503L19Rik	chr18:70453139-70501065	9.00038	13.4283	0.577223	0.00755508	yes
Usp36	chr11:118259652-118290244	5.87707	8.76739	0.577049	0.00216236	yes
Clcn3	chr8:60910388-60983311	36.6563	54.6355	0.575778	0.00145427	yes
Me1	chr9:86581362-86701253	3.07698	4.58538	0.575526	0.0118007	yes
Mfap3	chr11:57518664-57533817	11.0701	16.492	0.575097	0.00244513	yes
R3hdm2	chr10:127390310-127499384	4.59683	6.84631	0.574687	0.00504708	yes
Snapc1	chr12:73964529-73984820	16.4341	24.4706	0.574356	0.00311626	yes
Lpgat1	chr1:191718023-191784257	25.6866	38.2442	0.574223	0.00258324	yes
Top1	chr2:160645896-160722763	73.3978	109.257	0.573913	0.00454624	yes
Sec31a	chr5:100361648-100416234	26.2496	39.0693	0.57374	0.00298992	yes
Aplp2	chr9:31149556-31211815	36.384	54.1498	0.573653	0.00258324	yes
Cth	chr3:157894247-157925063	2.01886	3.00346	0.573084	0.0348164	yes
Npepps	chr11:97205855-97280576	12.8166	19.0649	0.572904	0.00403597	yes
Ppp2r1b	chr9:50856923-51009073	29.3108	43.5962	0.572767	0.00699437	yes
Rpl23	chr11:97777525-97782439	152.589	226.891	0.572348	0.00870444	yes
Cnot6l	chr5:96070332-96161990	16.577	24.6424	0.571963	0.00258324	yes
Sowahc	chr10:59221921-59226433	7.00039	10.4059	0.571895	0.00403597	yes
Uba1	chrX:20658301-20683179	96.4857	143.385	0.571501	0.00454624	yes
Edem3	chr1:151755373-151822328	30.3014	45.0255	0.571357	0.00258324	yes
Tsc22d2	chr3:58415688-58466787	2.60858	3.87584	0.571246	0.00627268	yes
1600014C10Rik	chr7:38183216-38197565	7.50775	11.1488	0.570433	0.00566351	yes
Ero1lb	chr13:12565882-12609528	11.6994	17.3643	0.569689	0.00428914	yes
Zdhhc24	chr19:4878667-4885397	5.08493	7.54677	0.56963	0.0114837	yes
Gatad1	chr5:3639960-3647936	47.8817	71.0404	0.569164	0.00145427	yes
Dph6	chr2:114516417-114654928	17.6226	26.1439	0.569046	0.003245	yes
Ino80d	chr1:63047800-63114267	5.03109	7.46335	0.568953	0.00216236	yes
Ppp1r12a	chr10:108162399-108277575	29.5385	43.7953	0.568181	0.00467182	yes
Tspan3	chr9:56135883-56161070	76.486	113.335	0.567321	0.00244513	yes
Zeb2	chr2:44983511-45114084	23.4826	34.7914	0.567142	0.00467182	yes
Rara	chr11:98937695-98974942	3.08689	4.57304	0.566999	0.00744706	yes
Tcp11l1	chr2:104679979-104712162	2.85245	4.22488	0.566707	0.00973453	yes
Ppp1r9b	chr11:94991211-95006898	30.7916	45.6043	0.566632	0.00390381	yes
Mapk1ip1	chr7:138835817-138846267	2.90077	4.29575	0.566474	0.0206813	yes
Luc7l2	chr6:38551443-38609470	49.0096	72.5761	0.56643	0.00479652	yes
Eepd1	chr9:25481596-25604110	6.08853	9.01607	0.566405	0.00590647	yes
Tmem181a	chr17:6270469-6317474	2.77223	4.10357	0.565831	0.0178389	yes
Rab10	chr12:3235790-3309969	109.775	162.458	0.56552	0.00338276	yes
Enox2	chrX:49009706-49288242	16.7825	24.8358	0.565466	0.00390381	yes
Anxa5	chr3:36448923-36475887	272.687	403.53	0.565428	0.00479652	yes
Uhmk1	chr1:170199255-170215393	24.0419	35.576	0.565354	0.00351242	yes
Elk1	chrX:20933394-20950608	3.74454	5.53728	0.564388	0.00699437	yes
Pld1	chr3:27938679-28133362	2.47839	3.66481	0.564335	0.00859434	yes
Sptlc2	chr12:87307888-87388230	29.7058	43.9254	0.564312	0.00338276	yes
Pip4k2c	chr10:127190377-127211622	9.67837	14.3062	0.563799	0.00926854	yes
Ufd1l	chr16:18812293-18835261	23.5651	34.8307	0.563707	0.00351242	yes
Vcp	chr4:42979963-43000507	39.4804	58.3531	0.563672	0.00258324	yes
A630001G21Rik	chr1:85717082-85736606	17.5507	25.9404	0.56367	0.003245	yes
Prmt5	chr14:54507181-54517470	52.9582	78.2647	0.563506	0.00390381	yes
Papd5	chr8:88199212-88259722	23.9965	35.4634	0.563506	0.00298992	yes
Cast	chr13:74693293-74808760	10.9003	16.1086	0.563464	0.00230418	yes
Atg9a	chr1:75180860-75192010	7.17046	10.5963	0.563419	0.00541779	yes
Hnrnpa2b1	chr6:51460434-51469894	223.455	330.201	0.563356	0.00755508	yes
E330009J07Rik	chr6:40407497-40436133	3.64502	5.38586	0.563249	0.0148245	yes
Tpp2	chr1:43934006-44002971	96.24	142.195	0.563165	0.00541779	yes
Zmat2	chr18:36793922-36799660	70.1637	103.657	0.563013	0.00403597	yes
Rela	chr19:5637489-5648130	10.6094	15.6729	0.562933	0.00467182	yes
Fancm	chr12:65075605-65132058	5.43696	8.03031	0.562655	0.003245	yes

Tanc2	chr11:105589985-105929303	0.659143	0.973512	0.562607	0.012881	yes
Clcn6	chr4:148006483-148038767	1.7616	2.60176	0.5626	0.0205916	yes
Sos1	chr17:80393751-80480453	6.03655	8.91501	0.562512	0.0036408	yes
Fam120b	chr17:15396201-15433581	10.8805	16.0678	0.562423	0.00298992	yes
Mgat4a	chr1:37439339-37541016	26.7253	39.4587	0.562135	0.00311626	yes
Bpnt1	chr1:185332158-185357769	38.6949	57.1305	0.562118	0.00216236	yes
Med13l	chr5:118560718-118765437	3.45107	5.09484	0.561992	0.00454624	yes
Ssbp1	chr6:40471414-40481823	63.4633	93.6394	0.561192	0.0258596	yes
Nub1	chr5:24685814-24710378	24.0479	35.4473	0.559763	0.00311626	yes
Snd1	chr6:28480347-28888832	62.3959	91.9562	0.559496	0.00416625	yes
Ercc4	chr16:13109735-13152009	4.22362	6.2243	0.559432	0.00504708	yes
Dnal1	chr12:84114327-84143510	0.632716	0.932299	0.559234	0.0312726	yes
Piga	chrX:164419786-164433915	6.93392	10.2147	0.5589	0.00710851	yes
Ywhah	chr5:33018815-33027966	203.713	300.056	0.55869	0.00504708	yes
Phf1	chr17:26933126-26937908	1.00335	1.47753	0.558368	0.0431522	yes
Morf4l2	chrX:136732947-136803361	363.852	535.698	0.55807	0.0106073	yes
Leprotl1	chr8:34135571-34146739	17.5684	25.8597	0.557725	0.00504708	yes
Dynll2	chr11:87979524-87998298	17.2582	25.4022	0.557672	0.00428914	yes
Chm	chrX:113040591-113185515	16.9867	25.0002	0.557535	0.00311626	yes
Kcmf1	chr6:72841113-72899979	38.2019	56.2179	0.557385	0.00311626	yes
Maged2	chrX:150806420-150814339	7.15754	10.5329	0.557362	0.00723058	yes
Casp6	chr3:129901414-129914112	8.24554	12.1333	0.557287	0.00881201	yes
Fam135a	chr1:24010757-24100341	9.28641	13.6635	0.557139	0.00351242	yes
Ehbp1	chr11:22005825-22286795	2.62477	3.86163	0.557021	0.00744706	yes
Klf7	chr1:64035670-64121389	4.47627	6.58551	0.556997	0.0190803	yes
Preb	chr5:30950065-30960327	10.9378	16.0912	0.556941	0.00529192	yes
Dusp7	chr9:106368631-106375723	12.6266	18.5738	0.556799	0.00590647	yes
Chordc1	chr9:18292266-18314000	108.097	158.981	0.556528	0.00517183	yes
Ankib1	chr5:3689998-3803124	17.9177	26.3519	0.556521	0.00441531	yes
Soat1	chr1:156428107-156474328	127.062	186.821	0.55612	0.00744706	yes
Ccdc173	chr2:69758056-69789486	1.28744	1.89223	0.555581	0.0407726	yes
Zfp11	chr5:129654594-129670088	1.09536	1.60987	0.555541	0.0242828	yes
Slc39a9	chr12:80644214-80683342	13.0312	19.152	0.555521	0.00663679	yes
R3hdm1	chr1:128103305-128237735	9.78341	14.3779	0.555446	0.00338276	yes
Rnf149	chr1:39551295-39577347	99.084	145.609	0.555377	0.00377278	yes
Ube2h	chr6:30211289-30304539	4.6391	6.81559	0.554995	0.0111512	yes
Sepw1	chr7:15917207-15922371	45.3671	66.6331	0.554593	0.00615568	yes
Ccdc86	chr19:10937159-10949266	12.7396	18.7044	0.554056	0.00351242	yes
Ankrd52	chr10:128377123-128394006	5.24207	7.69627	0.554023	0.00870444	yes
Sh3glb1	chr3:144683677-144720335	39.2578	57.6361	0.553993	0.00675438	yes
Smarca4	chr9:21616168-21704230	26.8807	39.41	0.551992	0.00639665	yes
Rbm47	chr5:66016548-66151954	5.16733	7.57568	0.551958	0.00687627	yes
Ddx6	chr9:44604891-44640731	28.1456	41.2601	0.551839	0.00351242	yes
Trappc11	chr8:47490127-47533470	25.7841	37.7978	0.551824	0.00351242	yes
Mettl6	chr14:31478797-31494977	20.3567	29.8359	0.551545	0.00517183	yes
Tcirg1	chr19:3896049-3907133	44.6819	65.4703	0.551151	0.00311626	yes
Dlst	chr12:85110832-85134091	74.7574	109.536	0.551118	0.00311626	yes
Rbm34	chr8:126947172-126971079	9.16883	13.4337	0.551043	0.00590647	yes
Mcfd2	chr17:87254442-87265947	37.3664	54.7424	0.550918	0.00390381	yes
E2f7	chr10:110745464-110787384	2.78451	4.07888	0.55075	0.00813362	yes
Kin	chr2:10080611-10092701	18.4647	27.0467	0.550685	0.00904622	yes
Amotl1	chr9:14541966-14615000	3.04308	4.45707	0.550564	0.00578321	yes
Erbb2ip	chr13:103818785-103920586	39.6506	58.0619	0.55025	0.003245	yes
Ddx19a	chr8:110974990-110997823	19.3079	28.2695	0.550057	0.00578321	yes
Ccnc	chr4:21727700-21767211	67.7453	99.1848	0.549999	0.0267306	yes
Zc3hav1l	chr6:38287393-38299259	1.16491	1.70499	0.549538	0.0244588	yes
Fbxw11	chr11:32642554-32746814	25.4234	37.2099	0.549527	0.00338276	yes
Bms1	chr6:118383380-118419417	42.1865	61.7428	0.54949	0.00687627	yes
Tmod3	chr9:75497783-75559657	58.4374	85.5211	0.549387	0.00377278	yes
Hhex	chr19:37434840-37440731	15.9778	23.3807	0.549254	0.00733312	yes
Agl	chr3:116739998-116808166	8.26471	12.0884	0.548584	0.00441531	yes
Evi5	chr5:107744794-107875107	12.9904	18.9937	0.548069	0.00428914	yes
Ddx47	chr6:135011611-135023776	71.5098	104.526	0.547651	0.00377278	yes
Lamp2	chrX:38401356-38456460	160.22	234.185	0.547595	0.00836289	yes
Lyrm7	chr11:54839288-54860591	1.97855	2.89179	0.547514	0.035282	yes

Srp14	chr2:118475842-118479696	177.996	260.114	0.5473	0.00351242	yes
Eya3	chr4:132639045-132724765	14.5916	21.3228	0.547266	0.00390381	yes
Timmdc1	chr16:38497842-38522663	17.9368	26.2072	0.547042	0.00479652	yes
Nol9	chr4:152039320-152061494	41.1359	60.0524	0.545826	0.0110431	yes
Trim36	chr18:46165299-46212607	1.0436	1.52331	0.545636	0.0327512	yes
Sirt2	chr7:28766751-28788665	11.3211	16.5206	0.545248	0.0104999	yes
Ost4	chr5:30888851-30907788	100.674	146.878	0.544925	0.0108186	yes
Mon2	chr10:122992060-123076505	11.0488	16.1166	0.544658	0.00454624	yes
Zfp800	chr6:28239930-28261601	24.9575	36.3888	0.544018	0.00492649	yes
Rab7	chr6:87999105-88045270	62.11	90.5544	0.54396	0.00566351	yes
Akap17b	chrX:36608182-36645414	1.05517	1.53752	0.543138	0.0206813	yes
Apbb2	chr5:66298724-66618817	7.98338	11.6298	0.54276	0.00615568	yes
Ssr3	chr3:65379656-65392553	114.197	166.339	0.5426	0.00553759	yes
Pyurf	chr6:57684738-57692078	6.32277	9.20921	0.54252	0.00813362	yes
Htt	chr5:34761739-34912534	9.8863	14.3938	0.541942	0.00441531	yes
Avl9	chr6:56714904-56761911	9.97523	14.5193	0.541552	0.00441531	yes
Piwil2	chr14:70372479-70429094	1.07319	1.56196	0.541451	0.0288864	yes
G730013B05Rik	chr16:50526244-50559459	30.3902	44.2259	0.541282	0.00504708	yes
Manf	chr9:106887414-106891938	94.4449	137.435	0.541203	0.00553759	yes
Tigd2	chr6:59208869-59212033	9.72238	14.1436	0.540765	0.00710851	yes
Prkar1a	chr11:109650948-109669648	86.213	125.383	0.540364	0.00529192	yes
Fnbp1	chr2:31026205-31142008	15.803	22.9733	0.539758	0.0104999	yes
Hiatl1	chr13:65065029-65112982	50.3727	73.1995	0.539192	0.00377278	yes
Ttc3	chr16:94358762-94469221	16.4354	23.8783	0.53889	0.00553759	yes
Aars	chr8:111033841-111055569	68.6564	99.7453	0.538855	0.00755508	yes
Bahd1	chr2:118901614-118924524	3.01596	4.38149	0.538805	0.0118007	yes
Dnajc25	chr4:59003192-59023398	10.7724	15.6477	0.53861	0.00733312	yes
Tmem41b	chr7:109972186-109986230	7.80755	11.3271	0.536835	0.00789883	yes
Ahcyl1	chr3:107663119-107696548	39.5086	57.3138	0.536718	0.00529192	yes
Rasgef1b	chr5:99217419-99252927	13.7325	19.9201	0.536634	0.00517183	yes
Ambra1	chr2:91730137-91918849	6.88414	9.98423	0.536375	0.00566351	yes
Cyp4v3	chr8:45305801-45333196	3.77603	5.47633	0.536338	0.012881	yes
Rock2	chr12:16894977-16988274	38.516	55.8434	0.535929	0.00504708	yes
Ampd3	chr7:110768205-110812408	2.53296	3.67044	0.535128	0.015043	yes
Mtdh	chr15:34082718-34142385	171.186	248.047	0.535043	0.0129867	yes
Snx4	chr16:33251455-33299562	39.114	56.6722	0.534956	0.00541779	yes
Diablo	chr5:123510459-123524164	31.0699	45.0165	0.534937	0.00529192	yes
Sh3bp1	chr15:78899785-78912052	6.17053	8.93981	0.53485	0.00950212	yes
Btaf1	chr19:36926078-37014057	37.7721	54.7163	0.53465	0.00479652	yes
Atxn7l1	chr12:33302514-33394760	2.64495	3.83144	0.534646	0.0327512	yes
Tmem65	chr15:58782268-58823427	10.2966	14.9133	0.534434	0.00984695	yes
Otud3	chr4:138895378-138913947	8.24516	11.9391	0.534077	0.00973453	yes
Slc6a6	chr6:91684066-91759063	47.5342	68.8152	0.533762	0.00881201	yes
St14	chr9:31088589-31131799	3.05332	4.42026	0.533751	0.0118007	yes
Arhgap25	chr6:87458544-87533259	3.94541	5.71115	0.533608	0.0104999	yes
Gpcpd1	chr2:132529081-132578248	9.29995	13.4572	0.533085	0.00663679	yes
Sec22a	chr16:35311130-35363918	11.314	16.3704	0.532978	0.00627268	yes
Kctd10	chr5:114314940-114380505	13.6143	19.6934	0.532589	0.00529192	yes
Calcoco1	chr15:102706776-102722178	1.32403	1.91524	0.532581	0.0296495	yes
Larp1	chr11:58009063-58062032	26.9187	38.935	0.532458	0.00699437	yes
Dcaf6	chr1:165329500-165460463	6.70751	9.70095	0.532349	0.00813362	yes
Clcn4-2	chr7:7282308-7300851	25.4293	36.7754	0.532248	0.00541779	yes
Gm1943	chr8:109339799-109340908	6.63557	9.59077	0.531426	0.0314474	yes
Pdcl3	chr1:38987813-38997236	50.8192	73.4501	0.531391	0.00441531	yes
D430020J02Rik	chr12:116401946-116405161	4.52911	6.54462	0.531082	0.0148245	yes
Swap70	chr7:110221702-110283506	48.914	70.6627	0.530701	0.00733312	yes
Krcc1	chr6:71272018-71285319	45.0019	65.0107	0.530693	0.00578321	yes
Ubqln1	chr13:58176155-58215653	74.9175	108.222	0.530615	0.00652015	yes
Tnks1bp1	chr2:85050459-85073048	0.500628	0.723135	0.530526	0.0407726	yes
Nktr	chr9:121719180-121759943	17.2653	24.9362	0.530364	0.00766958	yes
Gosr1	chr11:76726601-76763555	16.6827	24.0884	0.529983	0.00529192	yes
Lmln	chr16:33062520-33125659	1.69767	2.45113	0.529891	0.0275985	yes
Tapt1	chr5:44175161-44226606	19.1131	27.5923	0.529705	0.00627268	yes
Clec4d	chr6:123262106-123275268	69.0362	99.6527	0.529555	0.00663679	yes
Pura	chr18:36281161-36288244	24.6106	35.5217	0.529422	0.00870444	yes

Uprt	chrX:104482781-104506262	5.07824	7.3291	0.529305	0.0279961	yes
Ppme1	chr7:100326736-100371896	19.0115	27.4287	0.528814	0.00733312	yes
Rbfox2	chr15:77078989-77307053	4.45858	6.43208	0.528702	0.00755508	yes
Nrbp1	chr5:31240917-31251562	20.4196	29.4525	0.528434	0.00492649	yes
Angel1	chr12:86700501-86726460	0.896272	1.29257	0.528233	0.0496183	yes
Xdh	chr17:73883894-73950196	1.71839	2.47779	0.527995	0.0189835	yes
Oas1c	chr5:120800198-120812514	2.036	2.9356	0.52792	0.0300114	yes
Nlrc4	chr17:74426294-74459108	1.50895	2.17524	0.527627	0.0247412	yes
Zfp948	chr17:21567045-21588682	9.33138	13.4509	0.527546	0.00836289	yes
Prdm4	chr10:85891967-85916986	9.6677	13.9341	0.527378	0.00602767	yes
Srrm2	chr17:23803186-23829109	21.883	31.5228	0.526586	0.0178389	yes
Snx3	chr10:42502053-42535369	109.265	157.396	0.526566	0.00428914	yes
Mfsd4	chr1:132026620-132068062	2.60162	3.74738	0.526471	0.0300914	yes
Caml	chr13:55623004-55632416	30.3079	43.6532	0.526397	0.00813362	yes
Zmpste24	chr4:121059237-121098243	49.979	71.9839	0.526352	0.00652015	yes
Dicer1	chr12:104687741-104751952	11.9559	17.2191	0.526288	0.00441531	yes
Trim24	chr6:37870810-37968445	4.26824	6.14535	0.525855	0.0107144	yes
Pkn2	chr3:142790901-142882004	30.6434	44.1149	0.52569	0.00504708	yes
Zdhhc5	chr2:84671310-84715164	22.0711	31.7735	0.525664	0.0331378	yes
Camsap2	chr1:136268122-136346104	22.8773	32.9126	0.524724	0.00778355	yes
Upf3b	chrX:37091833-37110322	29.0847	41.832	0.524343	0.0124526	yes
Fnta	chr8:25998721-26015601	61.6744	88.6856	0.524027	0.00479652	yes
Sec24c	chr14:20674320-20694850	17.2114	24.7484	0.52397	0.00663679	yes
Grap	chr11:61653320-61672777	11.906	17.1193	0.523927	0.00962395	yes
Prune	chr3:95253673-95282076	4.86479	6.99374	0.523685	0.0137694	yes
Rps6ka3	chrX:159255781-159368244	11.1953	16.0924	0.523492	0.00699437	yes
Arfgef1	chr1:10137506-10232670	32.4245	46.6033	0.523345	0.00687627	yes
Cwc25	chr11:97745469-97766613	6.75346	9.70658	0.523337	0.00973453	yes
Tanc1	chr2:59612041-59846213	7.87999	11.3236	0.523064	0.00710851	yes
Zbtb37	chr1:161017755-161034259	3.14616	4.52053	0.5229	0.0162041	yes
Gorasp2	chr2:70661508-70691725	56.7291	81.5081	0.522855	0.00813362	yes
Gpi1	chr7:34201326-34230336	91.7341	131.77	0.522488	0.0124526	yes
Arfgef3	chr10:18588010-18743758	5.28931	7.59713	0.522376	0.00789883	yes
Phtf2	chr5:20758663-20882124	17.4913	25.1113	0.521698	0.00602767	yes
Fam13b	chr18:34409422-34506823	12.9922	18.6505	0.521566	0.00687627	yes
Gm13157	chr4:147753973-147809788	8.72775	12.5286	0.521541	0.00926854	yes
2900097C17Rik	chr2:156388062-156392979	43.065	61.8068	0.521248	0.00723058	yes
Fam160b2	chr14:70583294-70599835	1.97149	2.82912	0.521069	0.0205916	yes
Kif13a	chr13:46749087-46929718	2.27964	3.27054	0.520724	0.0143075	yes
Tyk2	chr9:21104067-21131275	7.52083	10.7855	0.520124	0.00744706	yes
Fra10ac1	chr19:38188478-38224132	26.006	37.2877	0.519857	0.00962395	yes
Naa60	chr16:3884618-3904781	20.439	29.3034	0.519741	0.00663679	yes
Zfyve1	chr12:83546940-83597147	2.13493	3.06082	0.519727	0.0205017	yes
Ccne1	chr7:38097983-38107490	24.0644	34.4956	0.519515	0.00813362	yes
Aldh1l2	chr10:83487446-83534140	3.01426	4.32026	0.519315	0.0140893	yes
Fgr	chr4:132974094-133001882	4.32805	6.2031	0.519272	0.0130978	yes
Zbtb18	chr1:177444660-177450764	14.032	20.11	0.519194	0.00652015	yes
Hook1	chr4:95959710-96024274	5.41571	7.76087	0.519067	0.0110431	yes
Etv1	chr12:38780257-38868215	3.97524	5.69415	0.518439	0.0123537	yes
Ankzf1	chr1:75192159-75210778	12.5708	18.0055	0.518365	0.0452271	yes
N4bp2	chr5:65763520-65826784	4.39252	6.29097	0.518234	0.00915655	yes
Zbtb11	chr16:55973267-56008912	15.2185	21.7916	0.517946	0.00755508	yes
Taf1	chrX:101532734-101601789	19.9362	28.5438	0.517785	0.00710851	yes
Rgs16	chr1:153740352-153745468	8.91539	12.7644	0.517753	0.0110431	yes
Zfp959	chr17:55892092-55898930	7.22501	10.3437	0.517685	0.0175233	yes
Pgrmc1	chrX:36598224-36606079	59.3841	85.0175	0.517684	0.00627268	yes
Ccnyl1	chr1:64691344-64725642	3.7746	5.40134	0.516994	0.0220515	yes
Arhgap1	chr2:91650117-91672320	17.4685	24.9926	0.51674	0.00915655	yes
Txn2	chr15:77915050-77928994	93.0716	133.157	0.516711	0.00663679	yes
Hnrnpc	chr14:52073379-52104028	235.85	337.385	0.516528	0.0192851	yes
Ap3s1	chr18:46741875-46790826	10.9316	15.6349	0.516269	0.0183683	yes
Gtdc1	chr2:44564411-44861622	6.26282	8.95715	0.516227	0.0126632	yes
Tmem263	chr10:85102626-85117747	13.8961	19.8719	0.516055	0.0107144	yes
Rbpj	chr5:53555778-53657445	17.2153	24.6095	0.51553	0.00578321	yes
Ciz1	chr2:32363009-32378313	8.312	11.8789	0.515137	0.0152609	yes

Stard3	chr11:98358367-98381112	28.7615	41.0881	0.514584	0.00778355	yes
AI314180	chr4:58800030-58912725	54.8997	78.4285	0.514581	0.0080155	yes
Ggct	chr6:54985094-54992867	9.10682	13.0086	0.514447	0.0216008	yes
Tmem245	chr4:56876012-56947429	7.6753	10.9637	0.514435	0.00744706	yes
Plekha5	chr6:140424098-140594906	1.76324	2.51867	0.514428	0.0198169	yes
Setd5	chr6:113077638-113153424	16.9531	24.2148	0.514343	0.00687627	yes
Ndfip2	chr14:105258672-105309298	25.1368	35.9017	0.514251	0.00926854	yes
Fip1l1	chr5:74535481-74702903	51.012	72.8541	0.514173	0.0171279	yes
Cmtm6	chr9:114731202-114749343	20.3269	29.0218	0.513744	0.00893132	yes
Ccnk	chr12:108179737-108203359	3.70891	5.29454	0.513509	0.0163026	yes
Frmd4a	chr2:4152862-4614043	4.24865	6.06175	0.512728	0.0102954	yes
Man1a2	chr3:100562204-100685473	31.5207	44.9613	0.512383	0.00723058	yes
Prkab2	chr3:97658211-97673067	1.96885	2.80833	0.51236	0.0297474	yes
Atg3	chr16:45158828-45188538	78.7803	112.294	0.511379	0.0104999	yes
Zc3h11a	chr1:133619870-133661399	24.9298	35.5199	0.510755	0.0369734	yes
Myo9b	chr8:71272713-71360712	17.2774	24.6144	0.510617	0.00766958	yes
Map3k2	chr18:32163088-32236751	9.92585	14.1404	0.510565	0.00710851	yes
Ints12	chr3:133091952-133110985	15.6966	22.3494	0.509786	0.00733312	yes
Naip5	chr13:100211739-100247336	1.82398	2.59697	0.50974	0.0243709	yes
Ier2	chr8:84661330-84662852	24.7026	35.1669	0.509558	0.0130978	yes
Anapc10	chr8:79711819-79777321	9.8191	13.9781	0.509509	0.012881	yes
Slc17a9	chr2:180725338-180742278	2.23418	3.18021	0.509379	0.042684	yes
Rnpc3	chr3:113605066-113630149	17.7373	25.2457	0.509255	0.0126632	yes
Prpf40a	chr2:53138475-53191187	123.213	175.313	0.508779	0.0139859	yes
Bnip3l	chr14:66985239-67008877	22.4524	31.9384	0.508421	0.00733312	yes
Ip6k2	chr9:108795993-108806333	3.70488	5.2692	0.508157	0.0286947	yes
Chd7	chr4:8690405-8868449	4.48675	6.38081	0.508068	0.00710851	yes
Os9	chr10:127094258-127121160	18.2876	26.0071	0.50804	0.00663679	yes
Ptpre	chr7:135537823-135686294	3.61197	5.13366	0.507202	0.012881	yes
Glrx2	chr1:143739348-143749678	10.5632	15.0124	0.507105	0.0107144	yes
Rab11fip3	chr17:25989035-26069177	2.25523	3.20315	0.506222	0.0361606	yes
Fam129a	chr1:151571372-151719347	37.5331	53.3077	0.506183	0.00984695	yes
Pgd	chr4:149149984-149166707	138.408	196.57	0.506123	0.0123537	yes
Flt1	chr5:147562195-147725988	1.25538	1.78232	0.505628	0.0245514	yes
2310022B05Rik	chr8:124635755-124663369	2.28337	3.24162	0.505552	0.0300114	yes
Nbeal2	chr9:110624788-110654161	4.18048	5.93282	0.505048	0.0138763	yes
Tgds	chr14:118111910-118132765	8.94942	12.6994	0.504899	0.0188827	yes
Pelp1	chr11:70392880-70410031	7.79742	11.0638	0.504785	0.0118007	yes
Gpatch8	chr11:102475915-102556158	5.85989	8.31404	0.504677	0.00766958	yes
Gpr65	chr12:98268634-98276722	16.1037	22.8445	0.504461	0.0135492	yes
Prmt10	chr8:77549396-77581338	14.168	20.0963	0.5043	0.0109347	yes
Hnrnph2	chrX:134601285-134607054	67.1506	95.2177	0.50383	0.0110431	yes
Crkl	chr16:17451984-17487440	12.651	17.9363	0.503633	0.00836289	yes
Enah	chr1:181896385-182019980	2.0652	2.92772	0.503493	0.0184699	yes
Ubxn4	chr1:128244180-128279377	49.9632	70.8203	0.503297	0.0102954	yes
Zfc3h1	chr10:115384958-115432771	14.1553	20.0499	0.502255	0.00904622	yes
Lpcat2	chr8:92855349-92919279	27.5023	38.951	0.502105	0.00915655	yes
Tmbim1	chr1:74285033-74353692	42.7542	60.5472	0.501993	0.00881201	yes
Pggt1b	chr18:46239948-46280850	19.0337	26.9456	0.501498	0.0106073	yes
Dync1li1	chr9:114688830-114723777	35.4296	50.1479	0.501231	0.00733312	yes
Gm20939	chr17:94864917-94878321	4.57477	6.47446	0.501061	0.0226558	yes
B4galt3	chr1:171270327-171276895	9.22868	13.0576	0.500696	0.0172208	yes
Camta1	chr4:151059522-151861768	3.7248	5.26953	0.500513	0.0471404	yes
Smarcc1	chr9:110132023-110240178	32.2288	45.5836	0.500167	0.0125567	yes
Hsp90aa1	chr12:110691035-110696395	167.958	237.536	0.500047	0.0152609	yes
Rad17	chr13:100617163-100651061	24.3795	34.4761	0.499928	0.00996804	yes
Vamp4	chr1:162570827-162599078	26.7544	37.8256	0.499587	0.0119065	yes
Supt7l	chr5:31514568-31526762	9.78683	13.8364	0.499556	0.0169236	yes
Nfrkb	chr9:31386191-31421334	5.5252	7.81115	0.499507	0.0119065	yes
Arcn1	chr9:44742143-44767808	86.789	122.676	0.499266	0.0124526	yes
Appl1	chr14:26918987-26970551	17.4162	24.6163	0.499183	0.0080155	yes
Nrp2	chr1:62703316-62818692	109.803	155.156	0.4988	0.041223	yes
Sc5d	chr9:42254176-42264300	29.6691	41.9226	0.498764	0.00859434	yes
Sfr1	chr19:47731755-47735588	135.752	191.736	0.498143	0.0227508	yes
Wasl	chr6:24613809-24664995	4.95692	7.00112	0.498142	0.0198169	yes

Mkl2	chr16:13256480-13417529	2.49896	3.52898	0.49792	0.0308859	yes
Cript	chr17:87025560-87035808	72.2703	102.054	0.497856	0.00904622	yes
Ubash3b	chr9:41013640-41157494	20.7002	29.2224	0.49743	0.00950212	yes
Copz1	chr15:103272917-103299864	71.1776	100.474	0.497332	0.00687627	yes
Tgm2	chr2:158116404-158146392	1.8131	2.55765	0.496357	0.0306743	yes
Rhbdd2	chr5:135632653-135646376	2.38642	3.36551	0.495975	0.0391038	yes
H2-Q4	chr17:35379616-35384674	15.7357	22.1904	0.495899	0.0152609	yes
Klhl12	chr1:134455554-134490873	6.93438	9.77882	0.495894	0.0189835	yes
Nadk	chr4:155562391-155591001	92.1236	129.884	0.495583	0.0136535	yes
Slc12a6	chr2:112266313-112368027	15.6703	22.0906	0.495402	0.0468864	yes
Pqbp1	chrX:7894518-7899269	53.8684	75.9388	0.495399	0.0167115	yes
Rsf1	chr7:97579895-97692782	8.83202	12.4499	0.49532	0.00789883	yes
Lrp6	chr6:134446477-134566913	12.6858	17.8795	0.495094	0.00904622	yes
Zhx1	chr15:58047002-58076508	20.3721	28.6955	0.494233	0.00881201	yes
Srsf11	chr3:158010492-158036639	93.576	131.764	0.49374	0.0175233	yes
Glg1	chr8:111157557-111259202	29.0848	40.9467	0.493479	0.0103944	yes
Cbll1	chr12:31484828-31499616	4.28363	6.02942	0.493188	0.0247412	yes
Nbeal1	chr1:60180598-60334705	8.82278	12.4166	0.492969	0.0100707	yes
Adam9	chr8:24949610-25016922	26.7588	37.6539	0.492786	0.0108186	yes
Bod1l	chr5:41787539-41844315	13.7839	19.395	0.492702	0.0122489	yes
Psmc4	chr7:28041701-28050092	80.9413	113.864	0.492361	0.0106073	yes
Rnf34	chr5:122850187-122868945	23.6351	33.2381	0.491909	0.011374	yes
Arih2	chr9:108602942-108649380	10.5102	14.7789	0.491759	0.0136535	yes
Mfsd11	chr11:116854014-116875637	3.31144	4.65631	0.491726	0.0340124	yes
Terf2ip	chr8:112011358-112020528	4.05213	5.69777	0.491715	0.0209746	yes
Map3k3	chr11:106084901-106155434	26.9812	37.9243	0.491165	0.0101798	yes
Dynlrb1	chr2:155236532-155250277	150.085	210.949	0.491113	0.0148245	yes
Itch	chr2:155133480-155226855	22.0913	31.0475	0.490996	0.00778355	yes
Vps53	chr11:76046225-76179630	16.0828	22.6015	0.490893	0.0111512	yes
Ap3s2	chr7:79875324-79920640	5.3314	7.49216	0.490867	0.011588	yes
Rap1gds1	chr3:138925896-139075201	23.9181	33.606	0.490612	0.0106073	yes
Rfx7	chr9:72532239-72622949	9.3149	13.0878	0.49061	0.0111512	yes
Ncoa5	chr2:165000356-165034779	13.1311	18.449	0.490548	0.0108186	yes
Pcnxl4	chr12:72536356-72580213	3.0255	4.25055	0.490476	0.0334914	yes
Dld	chr12:31331561-31351471	220.391	309.4	0.489407	0.0205916	yes
Rccd1	chr7:80316615-80324454	4.67233	6.555	0.488454	0.0311881	yes
Mfsd8	chr3:40818171-40846854	3.10837	4.36076	0.488423	0.0296495	yes
Zfp287	chr11:62711485-62729093	2.95938	4.14996	0.487805	0.0211866	yes
Xpnpep1	chr19:52991179-53038654	49.414	69.2911	0.48775	0.00973453	yes
Jun	chr4:95049035-95052222	34.0537	47.7387	0.487349	0.0108186	yes
Otub2	chr12:103376893-103406350	2.30704	3.23344	0.48703	0.0438319	yes
Atrnl1	chr19:57611033-58133340	2.27147	3.18244	0.486512	0.0266354	yes
Entpd5	chr12:84373875-84409029	9.52071	13.3387	0.486479	0.0124526	yes
Abhd4	chr14:54254128-54269169	2.83058	3.96434	0.485982	0.0399787	yes
Micu1	chr10:59702562-59864134	33.324	46.6577	0.485554	0.0101798	yes
Tbl1x	chrX:77511226-77660265	19.6884	27.5637	0.485423	0.0119065	yes
P3h1	chr4:119232914-119248977	3.75565	5.25786	0.485414	0.0250422	yes
Dennd6a	chr14:26573857-26634322	23.8114	33.3355	0.485408	0.0333148	yes
Cstf3	chr2:104590483-104665425	20.8151	29.1394	0.485338	0.023171	yes
Ppp3r1	chr11:17159297-17200380	68.536	95.9325	0.485158	0.0100707	yes
Atp6v1h	chr1:5083172-5162549	106.193	148.62	0.484945	0.0111512	yes
Ctsc	chr7:88278092-88310875	32.1597	45.0061	0.484868	0.0111512	yes
Kif21a	chr15:90933274-91049951	1.06656	1.49247	0.484735	0.0365931	yes
Ppp6r1	chr7:4631494-4658950	41.2529	57.7179	0.484524	0.0106073	yes
Phf12	chr11:77982815-78030535	7.16106	10.0168	0.484176	0.0175233	yes
Ntn3,Tbc1d24	chr17:24175430-24209387	3.23831	4.52783	0.483581	0.0275042	yes
Myo1g	chr11:6506547-6520958	13.0682	18.2678	0.483249	0.0122489	yes
Eif1	chr11:100319995-100322096	23.725	33.1633	0.483181	0.0269153	yes
Atp6v0a2	chr5:124629051-124724455	15.6321	21.8478	0.482978	0.0123537	yes
Asb7	chr7:66644566-66689561	4.63634	6.47827	0.482622	0.0184699	yes
Smurf2	chr11:106820063-106920715	15.6005	21.7916	0.482179	0.0111512	yes
Suv420h1	chr19:3767420-3818303	13.843	19.3359	0.48213	0.0288864	yes
Slc18a2	chr19:59260877-59296012	2.57515	3.59618	0.481811	0.0364231	yes
Asun	chr6:146549631-146577835	35.822	50.0131	0.481459	0.0143075	yes
Zfp119a	chr17:55864891-55878953	4.3154	6.02392	0.481207	0.0433818	yes

Ier3ip1	chr18:76930026-76941614	53.924	75.2608	0.48097	0.0146178	yes
Ccpg1	chr9:72985503-73039699	9.78199	13.6518	0.480895	0.0403132	yes
Hacd3	chr9:64986982-65021717	50.0284	69.807	0.480623	0.0119065	yes
Zfp963	chr8:69741638-69749962	3.85301	5.3751	0.480304	0.0413997	yes
Pigm	chr1:172376530-172384099	11.5836	16.1542	0.479822	0.012881	yes
Phrf1	chr7:141228787-141262751	12.0858	16.8537	0.479757	0.0125567	yes
Cnppd1	chr1:75135214-75142368	9.44424	13.1674	0.479467	0.0218594	yes
Slc30a9	chr5:67306956-67356145	34.6378	48.2885	0.479331	0.011374	yes
Gpatch2	chr1:187215510-187351429	4.42786	6.17278	0.479312	0.0210807	yes
Yipf6	chrX:98937780-98949020	13.6771	19.0635	0.479053	0.0103944	yes
Gtf2f1	chr17:57003401-57011288	76.7774	107.013	0.479029	0.0205916	yes
Cfl2	chr12:54858818-54862877	12.19	16.9888	0.478888	0.0244588	yes
Cndp2	chr18:84667464-84685702	42.4024	59.0911	0.478796	0.012881	yes
Hmgn5	chrX:109004536-109013380	10.2272	14.252	0.478755	0.0188827	yes
Metrnl	chr11:121702426-121717389	4.81978	6.71548	0.478522	0.0344621	yes
Uimc1	chr13:55027879-55100300	19.0818	26.5832	0.478314	0.0252408	yes
Rabgap1	chr2:37443284-37566437	18.2491	25.411	0.47763	0.0140893	yes
Plekhm3	chr1:64789120-64956824	15.5382	21.6315	0.477308	0.011588	yes
Ccnh	chr13:85189476-85213723	58.9108	82.0114	0.477293	0.0124526	yes
Taf11	chr17:27901127-27907724	22.9721	31.9784	0.477216	0.0191808	yes
Cnot6	chr11:49671496-49712722	30.7217	42.7661	0.477209	0.0138763	yes
Alpk1	chr3:127670309-127780527	12.0994	16.8427	0.477185	0.0136535	yes
Ralgapa1	chr12:55602889-55821516	12.6705	17.6338	0.476873	0.0121412	yes
Adprm	chr11:67037879-67052618	10.41	14.4836	0.47645	0.0287934	yes
Hmgcr	chr13:96648961-96670936	59.5216	82.8091	0.476376	0.0148245	yes
Capzb	chr4:139192898-139291820	143.674	199.879	0.47633	0.0156913	yes
Ccp110	chr7:118712610-118737018	6.92312	9.63101	0.476266	0.0200117	yes
Ext1	chr15:53068260-53346183	3.42293	4.76118	0.47609	0.0301829	yes
Arhgap12	chr18:6024449-6136098	16.8004	23.3679	0.476034	0.014404	yes
Slc26a2	chr18:61196853-61211596	9.85335	13.7032	0.475823	0.0171279	yes
Dennd1a	chr2:37776248-38287384	2.948	4.0998	0.47582	0.0234724	yes
Pgap1	chr1:54472999-54557684	5.6421	7.8463	0.475781	0.0147323	yes
Lasp1	chr11:97799671-97838764	27.3215	37.9861	0.475435	0.0126632	yes
Sde2	chr1:180851150-180868114	18.6983	25.991	0.475107	0.015043	yes
Senp5	chr16:31959669-32003287	15.3666	21.3558	0.474832	0.0109347	yes
Zfp654	chr16:64780346-64786321	7.86365	10.9282	0.474782	0.0190803	yes
D1Ertd622e	chr1:97643901-97662018	121.856	169.302	0.474417	0.0164008	yes
Hipk3	chr2:104426481-104494489	25.2454	35.061	0.473846	0.0139859	yes
Gm5801	chr14:56010291-56011480	52.3261	72.6504	0.473439	0.0153646	yes
Arl2bp	chr8:94666599-94674457	10.4365	14.4898	0.473404	0.0275042	yes
Atrn	chr2:130906495-131030326	9.33319	12.9569	0.473282	0.0140893	yes
Atxn2l	chr7:126491707-126503302	6.02877	8.36551	0.472591	0.0206813	yes
Abr	chr11:76416731-76577739	38.4482	53.3056	0.471368	0.0176188	yes
Hypk	chr2:121457087-121458440	157.832	218.811	0.47129	0.0175233	yes
Copb1	chr7:114215558-114254680	86.5771	120.025	0.471279	0.0145094	yes
Unc93b1	chr19:3935185-3949340	115.756	160.444	0.47098	0.0153646	yes
Emc1	chr4:139352586-139378735	11.8946	16.4827	0.47065	0.0129867	yes
Cdipt	chr7:126975913-126980501	26.216	36.3167	0.47019	0.0135492	yes
Dennd4c	chr4:86748554-86850602	10.9132	15.1157	0.469973	0.0141985	yes
Hspa13	chr16:75755190-75766818	24.5449	33.991	0.469733	0.011588	yes
Gtpbp2	chr17:46161031-46169370	7.70747	10.6714	0.469423	0.0216975	yes
Psmc6	chr14:45329823-45349071	215.298	298.031	0.469128	0.0186727	yes
Pdxdc1	chr16:13819276-13903145	55.3137	76.5681	0.469106	0.0294593	yes
Pik3c2a	chr7:116337275-116443458	24.8939	34.4539	0.468878	0.0145094	yes
Plod3	chr5:136982200-136996646	19.3961	26.8436	0.468812	0.0419513	yes
Ankrd11	chr8:122883821-123042284	12.9379	17.9	0.468352	0.0172208	yes
Dock7	chr4:98936658-99120915	14.4689	20.0057	0.467457	0.0162041	yes
Herc4	chr10:63243796-63317881	33.8108	46.7474	0.467402	0.0138763	yes
Slc10a7	chr8:78509327-78734012	12.3294	17.0404	0.46685	0.0159005	yes
Clint1	chr11:45851963-45910625	35.2457	48.6951	0.466328	0.0162041	yes
Acot7	chr4:152178099-152271855	72.4481	100.087	0.46624	0.016003	yes
Khdrbs1	chr4:129703168-129742303	35.3142	48.7862	0.466225	0.0184699	yes
Fam63b	chr9:70599013-70657174	6.56672	9.0692	0.465801	0.0167115	yes
Marf1	chr16:14109165-14159274	13.2081	18.2404	0.465719	0.0133195	yes
Six1	chr12:73041826-73046712	3.65443	5.04281	0.464582	0.0384504	yes

Esd	chr14:74732296-74750765	158.715	218.964	0.464261	0.0176188	yes
Lmbrd1	chr1:24678543-24766301	11.3866	15.7074	0.464117	0.0194933	yes
Nbr1	chr11:101552106-101581951	17.4698	24.0967	0.463974	0.0140893	yes
Hars	chr18:36766527-36783205	96.5322	133.137	0.463833	0.0145094	yes
Atmin	chr8:116943392-116960445	10.0927	13.9197	0.463813	0.0166035	yes
1200014J11Rik	chr11:73047866-73083579	11.16	15.3899	0.463646	0.014404	yes
Mapk9	chr11:49846750-49886421	23.3541	32.1987	0.463323	0.0175233	yes
Ampd2	chr3:108074061-108086666	21.3925	29.4913	0.463186	0.0154682	yes
Dnm1l	chr16:16312227-16359031	58.1682	80.1745	0.462913	0.0245514	yes
Tmem180	chr19:46356879-46375254	4.53385	6.24858	0.46279	0.0486308	yes
Ppif	chr14:25694169-25701282	23.4988	32.3858	0.462776	0.0265349	yes
Zfp608	chr18:54888044-54990180	1.66395	2.29291	0.462568	0.0332263	yes
Sep-08	chr11:53519735-53544096	6.32946	8.72114	0.462434	0.0233736	yes
Kpna1	chr16:35983362-36036162	49.2022	67.7669	0.461859	0.0176188	yes
Ubxn7	chr16:32332251-32393747	8.38787	11.5517	0.461727	0.0134387	yes
Sin3a	chr9:57072039-57128368	20.6606	28.4454	0.461315	0.0208855	yes
Ap4e1	chr2:127008710-127069814	9.01436	12.4093	0.461128	0.0167115	yes
Coa4	chr7:100537099-100539812	14.0492	19.339	0.461026	0.0339376	yes
Sepn1	chr4:134537891-134552166	4.38177	6.03077	0.460827	0.0357258	yes
Sdad1	chr5:92284009-92310024	35.3838	48.6936	0.460643	0.0180585	yes
Hif1an	chr19:44562853-44576274	8.6879	11.9514	0.460105	0.0199123	yes
Amfr	chr8:93971587-94012640	36.5805	50.292	0.459257	0.0226558	yes
Kpna6	chr4:129643978-129672767	16.908	23.2414	0.458989	0.0135492	yes
Csnk2a2	chr8:95446095-95488820	6.98588	9.6007	0.458698	0.0252408	yes
Gtf2h1	chr7:46796093-46823800	64.9986	89.3215	0.458598	0.0172208	yes
Med14	chrX:12675370-12761973	14.9629	20.5508	0.457808	0.0168229	yes
AI987944	chr7:41372929-41393379	21.7307	29.8433	0.457675	0.0217808	yes
Minos1	chr4:139101813-139131113	37.5374	51.5484	0.457599	0.0154682	yes
Cox7a2l	chr17:83501916-83514333	388.352	533.28	0.457527	0.038088	yes
Mllt3	chr4:87769924-88033407	5.13234	7.04533	0.45705	0.0214996	yes
Zfp568	chr7:29983954-30028282	9.11003	12.5025	0.456686	0.0175233	yes
Gtf2e2	chr8:33731913-33777173	47.0356	64.5504	0.456673	0.0153646	yes
Gnaq	chr19:16132830-16387453	17.7825	24.3959	0.456177	0.0198169	yes
Zfp770	chr2:114193460-114201432	9.40283	12.8979	0.455964	0.0239982	yes
Ap1g1	chr8:109778582-109864209	22.8559	31.3433	0.455592	0.0119065	yes
Sltm	chr9:70542777-70592232	38.6039	52.9102	0.4548	0.0241896	yes
Ric8b	chr10:84917612-85018337	5.96705	8.17313	0.453872	0.0263336	yes
Xpnpep3	chr15:81400187-81454888	4.31251	5.90602	0.453658	0.0408538	yes
Slc35b4	chr6:34155878-34177054	13.4505	18.4181	0.453466	0.0241896	yes
Asns	chr6:7675170-7693182	132.665	181.618	0.453115	0.0219511	yes
Akap9	chr5:3928185-4080204	15.4737	21.1808	0.452938	0.0253474	yes
Fam102a	chr2:32535358-32569750	9.56372	13.091	0.452929	0.0220515	yes
Mtfr1	chr3:19187328-19220817	20.2367	27.6994	0.452885	0.0173206	yes
Tlk1	chr2:70712407-70825480	30.4376	41.6569	0.452703	0.0216975	yes
Phc3	chr3:30899294-30969415	3.35248	4.58663	0.452205	0.0235758	yes
Akap11	chr14:78492245-78536860	14.7375	20.1609	0.452065	0.0189835	yes
Fmr1	chrX:68678540-68717961	45.7808	62.6061	0.451562	0.0190803	yes
Ubn2	chr6:38433924-38512763	4.79899	6.55925	0.450802	0.0315437	yes
Fbxw2	chr2:34804363-34826235	38.2666	52.2808	0.450193	0.0248334	yes
Tmem29	chrX:150397772-150459150	9.82256	13.4176	0.449954	0.0445395	yes
Btbd10	chr7:113315643-113369339	15.5921	21.2957	0.449744	0.0225652	yes
Phlpp2	chr8:109868602-109944671	1.7314	2.36439	0.449526	0.0461477	yes
Nol6	chr4:41114426-41124339	10.4913	14.3188	0.448714	0.0203128	yes
Tfg	chr16:56690328-56717450	52.038	71.0141	0.448539	0.0192851	yes
Cog6	chr3:52982122-53017223	9.08521	12.3973	0.448439	0.031351	yes
Xylt2	chr11:94663846-94677493	4.77813	6.51986	0.448395	0.031351	yes
Abca3	chr17:24352022-24414513	6.64777	9.07084	0.448366	0.0226558	yes
Ptpn22	chr3:103859794-103912252	48.6378	66.3495	0.448009	0.0229541	yes
Sbds	chr5:130245731-130255462	33.9657	46.3343	0.447999	0.0185753	yes
Rnps1	chr17:24414674-24425897	15.8507	21.621	0.447887	0.0320425	yes
C1galt1	chr6:7845223-7872042	26.3171	35.8952	0.447792	0.0224699	yes
Ubap1	chr4:41348995-41389766	15.0631	20.5422	0.447574	0.0208855	yes
Arhgef11	chr3:87618750-87748623	6.69492	9.12966	0.447494	0.0213983	yes
Zranb2	chr3:157534396-157548339	126.512	172.52	0.447487	0.0300114	yes
D19Bwg1357e	chr19:27388697-27429908	78.9426	107.589	0.446654	0.026831	yes

Lrpap1	chr5:35091505-35105697	18.3946	25.058	0.445989	0.0225652	yes
Sh3bp2	chr5:34525783-34563639	18.1868	24.7736	0.445914	0.0204073	yes
Snx19	chr9:30427328-30466726	8.51155	11.593	0.445764	0.0218594	yes
Cyb5r3	chr15:83153500-83172208	38.8245	52.8795	0.445742	0.0293726	yes
Eif4e2	chr1:87213913-87240488	162.552	221.382	0.445637	0.0438319	yes
Abhd2	chr7:79273265-79361601	13.0862	17.817	0.445204	0.0243709	yes
Tmed7	chr18:46585927-46597535	109.357	148.879	0.445096	0.020214	yes
Adnp2	chr18:80127134-80151482	10.7131	14.5834	0.444954	0.0252408	yes
Prune2	chr19:16956117-17223932	1.05395	1.43436	0.4446	0.0372338	yes
Epc2	chr2:49451485-49551609	18.775	25.5276	0.443247	0.031351	yes
Usp8	chr2:126707327-126759314	61.6834	83.8591	0.443087	0.0359964	yes
Ppwd1	chr13:104205121-104249622	32.4834	44.1561	0.442913	0.0488815	yes
Tnfrsf1a	chr6:125349722-125362483	23.8946	32.4777	0.442765	0.0239982	yes
Arid1a	chr4:133679007-133753611	6.1444	8.34981	0.442472	0.0235758	yes
Phf20l1	chr15:66577571-66645255	16.1433	21.9365	0.442399	0.020214	yes
Srgap2	chr1:131285250-131527361	12.0905	16.4286	0.442344	0.0216975	yes
BC005561	chr5:104508351-104522383	7.32706	9.95046	0.441528	0.0276926	yes
Dynll1	chr5:115297109-115300990	117.907	160.105	0.441367	0.0205017	yes
Zfp281	chr1:136624900-136630391	16.0898	21.847	0.441288	0.0219511	yes
Ptpn4	chr1:119658092-119837071	29.9122	40.6119	0.441169	0.020214	yes
Rpf2	chr10:40223245-40247039	67.1064	91.1037	0.44106	0.0296495	yes
Vps18	chr2:119288741-119298453	14.0852	19.1212	0.440997	0.0262535	yes
Fbxl20	chr11:98082553-98149616	1.68698	2.28974	0.440738	0.0407726	yes
Cul4b	chrX:38531620-38576196	37.2458	50.5292	0.440041	0.0233736	yes
Hk1	chr10:62268854-62379908	27.4422	37.2268	0.439944	0.028486	yes
S100pbp	chr4:129150824-129189482	12.138	16.4645	0.439829	0.0340124	yes
Ywhab	chr2:163995196-164018587	87.8896	119.21	0.439735	0.0233736	yes
Cul3	chr1:80266817-80340430	94.3422	127.954	0.439652	0.0287934	yes
Rictor	chr15:6708380-6800400	10.9475	14.842	0.439079	0.0205017	yes
Rab3gap1	chr1:127868772-127943876	26.9979	36.6004	0.439008	0.0209746	yes
Zcchc9	chr13:91796532-91807696	15.5153	21.0151	0.437735	0.0353816	yes
Mast3	chr8:70778116-70792433	5.34773	7.24265	0.43759	0.0301829	yes
Abhd5	chr9:122351615-122381523	14.2812	19.3325	0.436913	0.031351	yes
Pias2	chr18:77065207-77155708	27.0909	36.6677	0.436703	0.0408538	yes
Agpat6	chr8:23172945-23208453	43.3872	58.7029	0.436163	0.0246488	yes
Rnf115	chr3:96727610-96791155	48.2189	65.2394	0.436143	0.0205017	yes
Pdap1	chr5:145128769-145140089	99.8622	135.089	0.435901	0.035904	yes
Zscan22	chr7:12897808-12909083	4.06513	5.49783	0.435562	0.0479165	yes
Prkrip1	chr5:136180356-136198954	17.6857	23.9164	0.435417	0.0282881	yes
Kcnq1ot1	chr7:143107253-143427042	2.42617	3.27939	0.434742	0.0333148	yes
Stam	chr2:14074111-14148330	13.2825	17.9521	0.434631	0.027792	yes
Acd	chr8:105690905-105701095	9.88086	13.3526	0.434412	0.0440292	yes
Pctp	chr11:89983416-90002894	10.9294	14.7695	0.434409	0.0409198	yes
Emc2	chr15:43477228-43527777	62.7466	84.7435	0.433564	0.0260568	yes
Trim23	chr13:104179097-104202048	7.9521	10.7367	0.433145	0.0382622	yes
Tmem214	chr5:30869646-30877467	13.9687	18.8574	0.432936	0.0333148	yes
Smarcc2	chr10:128459235-128490174	9.28112	12.5275	0.432722	0.0343814	yes
Il6st	chr13:112464069-112506860	14.4465	19.4991	0.432685	0.0241896	yes
Ptpn2	chr18:67665500-67724621	59.0175	79.6302	0.432174	0.0399787	yes
Med10	chr13:69809881-69816094	38.4968	51.9293	0.431808	0.0292744	yes
Atg2b	chr12:105613539-105685241	7.7021	10.3887	0.431698	0.0237919	yes
Ggps1	chr13:14052444-14063401	14.2703	19.2443	0.43141	0.0334032	yes
Xiap	chrX:42067835-42109664	52.4002	70.6536	0.431192	0.0305714	yes
Tmed4	chr11:6270713-6274837	30.3121	40.8525	0.430531	0.0300114	yes
Magi1	chr6:93675452-94283917	2.01713	2.71745	0.429948	0.045321	yes
Donson	chr16:91679264-91688728	13.5179	18.2055	0.429499	0.0339376	yes
Smc5	chr19:23206440-23273897	31.1989	42.0162	0.429452	0.0285904	yes
Tmem229b	chr12:78961794-79007277	14.0811	18.9609	0.429268	0.0296495	yes
Elavl1	chr8:4284781-4325100	31.9239	42.9842	0.429171	0.0278914	yes
Ogfod1	chr8:94037197-94067922	10.0191	13.487	0.428809	0.0250422	yes
Rreb1	chr13:37826037-37952005	3.53928	4.76292	0.428391	0.0366917	yes
Dnajc13	chr9:104151596-104262930	22.2842	29.9837	0.428154	0.0252408	yes
Dcp2	chr18:44380499-44424969	16.3	21.9287	0.427946	0.0243709	yes
Rab8a	chr8:72161199-72181366	103.464	139.191	0.42794	0.0402316	yes
Stx18	chr5:38039229-38159467	15.4904	20.8373	0.427787	0.0340124	yes

Ing5	chr1:93803964-93822100	21.3407	28.7008	0.427481	0.0242828	yes
Smap1	chr1:23761925-23922381	28.96	38.9349	0.427002	0.0387143	yes
Setd8	chr5:124439929-124462307	54.3499	73.0673	0.426949	0.0381645	yes
Slc39a6	chr18:24579880-24603817	28.9161	38.8729	0.426891	0.0334914	yes
Adcy9	chr16:4284885-4420498	2.13345	2.86734	0.426521	0.0489649	yes
Naa30	chr14:49172226-49191031	14.8973	20.0208	0.426447	0.0300114	yes
Fkbp15	chr4:62300341-62360548	15.3037	20.5602	0.425973	0.0266354	yes
Tmem30a	chr9:79768940-79793430	94.9596	127.568	0.425876	0.0342003	yes
Slc35a3	chr3:116670797-116712280	49.9018	67.0335	0.42579	0.0246488	yes
Chfr	chr5:110135829-110176504	13.997	18.7846	0.424432	0.0328464	yes
S100a13	chr3:90514434-90524581	29.9549	40.1888	0.424002	0.0496183	yes
Stxbp5	chr10:9755546-9901040	9.09367	12.1972	0.423611	0.0281835	yes
Atp6v1c1	chr15:38661903-38692444	120.876	162.118	0.423521	0.0302743	yes
Scfd1	chr12:51377512-51450102	45.2658	60.7059	0.423413	0.0258596	yes
Dis3l2	chr1:86703803-87050097	13.1775	17.6717	0.423368	0.0342003	yes
Ufm1	chr3:53853375-53863807	7.84578	10.5209	0.423275	0.0364231	yes
Ube2q1	chr3:89773608-89783997	71.3463	95.6325	0.422664	0.033762	yes
Msmo1	chr8:64718144-64733578	105.472	141.372	0.422642	0.0273047	yes
Itgb1	chr8:128685653-128733579	103.803	139.095	0.422221	0.0385337	yes
Fhl3	chr4:124700698-124708611	40.4494	54.2006	0.422191	0.0320425	yes
Rragc	chr4:123917432-123936997	44.9687	60.2515	0.422077	0.0241896	yes
Ppp6c	chr2:39196797-39226338	68.4275	91.6073	0.420887	0.0267306	yes
Cdc37	chr9:21138540-21149906	141.973	190.042	0.420696	0.0327512	yes
Prickle4,Tomm6	chr17:47686644-47694736	65.4036	87.513	0.420128	0.0349096	yes
Sart1	chr19:5377522-5390069	16.845	22.536	0.41991	0.0272102	yes
Mtmr1	chrX:71364759-71419196	17.3215	23.1632	0.419268	0.0263336	yes
Gga1	chr15:78877189-78894585	7.04056	9.40907	0.418363	0.0360752	yes
Gpatch1	chr7:35276543-35318440	9.16701	12.2478	0.418	0.0378013	yes
Srp68	chr11:116245165-116274217	45.5596	60.8663	0.41789	0.0309827	yes
Actn1	chr12:80167541-80260371	28.9576	38.6828	0.417748	0.0305714	yes
Saraf	chr8:34154562-34170847	35.94	48.0001	0.417444	0.0331378	yes
Tcerg1	chr18:42511486-42575785	34.4724	46.0263	0.417018	0.0454066	yes
Wipf1	chr2:73429609-73529487	11.554	15.4259	0.416967	0.0344621	yes
BC005537	chr13:24801656-24812899	185.538	247.709	0.416933	0.0397251	yes
Itpr2	chr6:146108298-146502223	5.71039	7.62277	0.416728	0.0275985	yes
Rabggtb	chr3:153907288-153912966	81.7958	109.183	0.41665	0.0347296	yes
Tmem135	chr7:89139720-89338787	27.0942	36.1541	0.416176	0.031965	yes
Tmem33	chr5:67260564-67291461	30.3613	40.5126	0.416138	0.0312726	yes
Ipo13	chr4:117894492-117914999	10.9751	14.6442	0.416088	0.0431522	yes
Uba5	chr9:104046587-104063121	23.8021	31.7575	0.416009	0.032407	yes
Gpatch4	chr3:88043105-88055994	30.6677	40.9145	0.415891	0.041488	yes
Tmem106b	chr6:13069758-13089269	15.7318	20.988	0.415886	0.028985	yes
Dnaja2	chr8:85537639-85555271	140.567	187.445	0.415205	0.0413997	yes
Tgs1	chr4:3574878-3616623	9.97584	13.3008	0.414998	0.0370511	yes
Fbxo38	chr18:62504058-62548743	21.9689	29.2855	0.414725	0.0283815	yes
Eif2s2	chr2:154871409-154892906	77.8432	103.751	0.414484	0.0409198	yes
Ube2g2	chr10:77622320-77645990	55.8465	74.429	0.414398	0.0364231	yes
Hsbp1	chr8:119344537-119348929	84.8048	113.012	0.414257	0.0360752	yes
Tmbim6	chr15:99392946-99410049	91.8071	122.325	0.414042	0.0381645	yes
Atg12	chr18:46732416-46741579	17.9622	23.9306	0.413887	0.039359	yes
Tbc1d5	chr17:50733126-51179352	11.0299	14.6878	0.4132	0.031351	yes
Snx7	chr3:117781496-117868936	29.1308	38.777	0.412655	0.0350095	yes
Paip1	chr13:119428599-119460323	30.8853	41.093	0.411976	0.0288864	yes
Bcor	chrX:12036737-12160355	6.15551	8.18971	0.411932	0.0432236	yes
Ccdc93	chr1:121431066-121506460	10.4768	13.9379	0.411811	0.033674	yes
Clptm1l	chr13:73604001-73620639	85.2128	113.321	0.411273	0.033762	yes
Ddx10	chr9:53098453-53248112	43.8798	58.3498	0.411169	0.037332	yes
Zfp326	chr5:105876567-105915820	11.9859	15.938	0.411136	0.0435636	yes
Tcf25	chr8:123373710-123404174	60.5781	80.5286	0.410705	0.035189	yes
Krit1	chr5:3803164-3844515	12.2548	16.2904	0.410675	0.0365219	yes
Senp6	chr9:80066902-80144780	37.1074	49.3193	0.410447	0.0397999	yes
Capns1	chr7:30186941-30195048	77.6916	103.234	0.410089	0.0354745	yes
Fam32a	chr8:72219729-72223775	66.1339	87.8546	0.409729	0.0350894	yes
Ercc3	chr18:32240330-32270147	27.2028	36.1339	0.409594	0.0341064	yes
Psmc2	chr5:21785282-21803784	100.253	133.156	0.409474	0.0361606	yes

Picalm	chr7:90130231-90209447	139.693	185.482	0.409021	0.0497577	yes
Erlin1	chr19:44034942-44069785	30.8833	40.9963	0.408664	0.0360752	yes
Tprkb	chr6:85915718-85930284	20.0375	26.5949	0.408447	0.0404912	yes
Tpst1	chr5:130073325-130135733	23.1636	30.734	0.407975	0.0397999	yes
Lsm12	chr11:102163488-102185256	23.4188	31.0699	0.407848	0.0469626	yes
Usp31	chr7:121642020-121707253	8.47671	11.2352	0.40645	0.0350894	yes
Trim44	chr2:102300118-102400900	30.6131	40.5752	0.406448	0.0392619	yes
Fam129b	chr2:32876133-32961255	66.8057	88.5321	0.406229	0.0461477	yes
Srek1ip1	chr13:104817238-104838659	29.785	39.4645	0.40597	0.0475883	yes
Strn	chr17:78653963-78736560	15.5798	20.6393	0.405715	0.0327512	yes
Derl1	chr15:57869501-57892418	26.6023	35.232	0.405339	0.0426043	yes
Tnks2	chr19:36834231-36893477	49.1384	65.0613	0.404948	0.0391938	yes
Tia1	chr6:86404218-86433405	15.2516	20.1931	0.4049	0.0385337	yes
Psmd14	chr2:61711693-61800376	179.173	237.178	0.404614	0.040068	yes
Rprd1b	chr2:158028496-158091797	10.2104	13.5125	0.404252	0.0472165	yes
Ptpn11	chr5:121130532-121191397	23.5041	31.105	0.404235	0.0357258	yes
Slc7a6os	chr8:106200437-106210933	20.2355	26.7707	0.403765	0.0409198	yes
Kpna4	chr3:69072220-69127092	32.9488	43.5841	0.403577	0.0334914	yes
Mbd1	chr18:74268287-74282684	16.5985	21.9466	0.402945	0.0402316	yes
Coro1c	chr5:113842438-113908706	66.8158	88.2173	0.400872	0.0396355	yes
Prr14l	chr5:32789206-32854230	12.7016	16.7677	0.400674	0.0360752	yes
Plekha3	chr2:76675314-76697335	33.6901	44.471	0.400538	0.0359041	yes
Ttc7b	chr12:100276608-100520822	10.2904	13.5823	0.400429	0.0444694	yes
Gm15800	chr5:121220218-121368577	2.17614	2.87071	0.399642	0.0451577	yes
Anp32b	chr4:46451116-46472523	66.8257	88.1172	0.399021	0.0404022	yes
Map4k3	chr17:80580512-80728025	12.5654	16.5652	0.398696	0.0424213	yes
Traf6	chr2:101678419-101701668	10.1452	13.3728	0.398502	0.0428589	yes
Ago1	chr4:126435012-126468421	5.73982	7.56435	0.398209	0.0456796	yes
Wbp1l	chr19:46599105-46657389	20.0505	26.4176	0.397861	0.0408538	yes
Gm21992,Rbm14,Rbm4	chr19:4784292-4811634	40.9572	53.905	0.396302	0.0489649	yes
Dcp1a	chr14:30008999-30527056	8.70622	11.4584	0.396287	0.045765	yes
Cabin1	chr10:75646109-75764357	6.98259	9.18583	0.395647	0.0383599	yes
Pank3	chr11:35769494-35791285	53.2225	70.0143	0.395615	0.0365931	yes
Pitpnb	chr5:111330696-111388359	24.2462	31.8735	0.394602	0.0435636	yes
Sri	chr5:8046077-8069314	30.5685	40.178	0.394357	0.0444694	yes
Arid2	chr15:96287521-96405463	10.1027	13.2782	0.394309	0.0403132	yes
Rab5a	chr17:53479233-53539451	31.307	41.1467	0.39429	0.0401573	yes
Prkaca	chr8:83972977-83996442	17.6693	23.2178	0.393986	0.0450719	yes
Ythdf3	chr3:16183182-16217037	35.3571	46.4531	0.393778	0.0433027	yes
Chp1	chr2:119547706-119587022	44.8554	58.7499	0.389304	0.0397999	yes
Cpne1,Rbm12	chr2:156071840-156111965	45.6418	59.7794	0.389292	0.0434767	yes
Fam3c	chr6:22306521-22356081	36.762	48.1323	0.388792	0.0483622	yes
Rin2	chr2:145786115-145887616	10.6778	13.9795	0.388701	0.0464197	yes
Usp12	chr5:146734811-146794956	16.0657	21.0327	0.388656	0.0449001	yes
Rnf216	chr5:142990892-143113020	11.3516	14.8605	0.388582	0.0476469	yes
Aqr	chr2:114101160-114175339	34.8923	45.6448	0.387541	0.048798	yes
Prpf6	chr2:181601318-181655661	30.5349	39.9293	0.38699	0.0480846	yes
Slc39a7	chr17:34028265-34031690	47.4712	62.0564	0.386528	0.0474025	yes
Zcchc17	chr4:130316084-130359943	43.0706	56.2234	0.384466	0.0496925	yes
Hccs	chrX:169311530-169320343	34.8944	45.5396	0.384127	0.0498495	yes
Tug1	chr11:3639784-3648814	24.9152	32.5149	0.384078	0.0499323	yes
Rnf6	chr5:146209193-146221457	27.9934	36.5249	0.383797	0.0463347	yes
Erlin2	chr8:27023798-27039435	20.0038	26.0896	0.383205	0.0449001	yes
Man2a1	chr17:64601648-64755110	17.1313	22.3147	0.381358	0.0487144	yes
Ppp1r21	chr17:88530123-88588367	25.1597	32.7714	0.381325	0.0480846	yes
Cdc6	chr11:98907888-98923942	11.8713	15.4607	0.381127	0.0493334	yes
Vps33a	chr5:123528759-123573015	24.6283	32.0692	0.380868	0.0465981	yes
Jarid2	chr13:44730773-44921643	8.23255	10.7193	0.380795	0.0464197	yes
