# Supplementary material for: RNA Sequencing of Murine Norovirus-Infected Cells Reveals Transcriptional Alteration of Genes Important to Viral Recognition and Antigen Presentation
Source: Front Immunol. 2017 Aug 11;8:959. doi: 10.3389/fimmu.2017.00959 (PMC5554501; doi:10.3389/fimmu.2017.00959)
Supplement: Supplementary file 4 [file Table_4.PDF]

**TABLE S4** Enrichment terms generated from GOrilla (MNV 12 hpi)

| | Upregulated genes | | |
|---|---|---|---|
| **Goterm** | **Description** | **P-value** | **Enrichment (N, B, n, b)** |
| GO:0006950 | response to stress | 1.08E-21 | 4.20 (20634,2194,121,54) |
| GO:0002376 | immune system process | 8.34E-21 | 5.72 (20634,1222,121,41) |
| GO:0006952 | defense response | 2.01E-19 | 6.80 (20634,853,121,34) |
| GO:0043207 | response to external biotic stimulus | 3.07E-18 | 7.86 (20634,629,121,29) |
| GO:0009607 | response to biotic stimulus | 9.47E-18 | 7.54 (20634,656,121,29) |
| GO:0009605 | response to external stimulus | 4.53E-17 | 5.70 (20634,1018,121,34) |
| GO:0002682 | regulation of immune system process | 1.03E-16 | 5.34 (20634,1118,121,35) |
| GO:0050896 | response to stimulus | 7.05E-16 | 2.62 (20634,4234,121,65) |
| GO:0006954 | inflammatory response | 1.18E-15 | 10.29 (20634,348,121,21) |
| GO:0031347 | regulation of defense response | 1.48E-15 | 7.70 (20634,554,121,25) |
| GO:0048583 | regulation of response to stimulus | 1.80E-15 | 3.00 (20634,3123,121,55) |
| GO:0002684 | positive regulation of immune system process | 8.94E-15 | 6.73 (20634,659,121,26) |
| GO:0032496 | response to lipopolysaccharide | 2.49E-13 | 11.02 (20634,263,121,17) |
| GO:0001817 | regulation of cytokine production | 4.88E-13 | 7.08 (20634,530,121,22) |
| GO:0002237 | response to molecule of bacterial origin | 6.50E-13 | 10.39 (20634,279,121,17) |
| GO:0080134 | regulation of response to stress | 7.90E-13 | 4.51 (20634,1173,121,31) |
| GO:0006955 | immune response | 1.00E-12 | 6.07 (20634,674,121,24) |
| GO:0001819 | positive regulation of cytokine production | 1.71E-12 | 8.92 (20634,344,121,18) |
| GO:0048584 | positive regulation of response to stimulus | 1.82E-12 | 3.74 (20634,1643,121,36) |
| GO:0050727 | regulation of inflammatory response | 4.19E-12 | 10.22 (20634,267,121,16) |
| GO:0032101 | regulation of response to external stimulus | 7.61E-12 | 5.25 (20634,812,121,25) |
| GO:0051716 | cellular response to stimulus | 1.01E-11 | 3.27 (20634,2034,121,39) |
| GO:0006935 | chemotaxis | 1.20E-11 | 10.61 (20634,241,121,15) |
| GO:0042330 | taxis | 1.34E-11 | 10.53 (20634,243,121,15) |
| GO:0009966 | regulation of signal transduction | 1.82E-11 | 3.07 (20634,2278,121,41) |
| GO:0009893 | positive regulation of metabolic process | 3.43E-11 | 2.73 (20634,2869,121,46) |
| GO:0060326 | cell chemotaxis | 5.13E-11 | 14.21 (20634,144,121,12) |
| GO:0048518 | positive regulation of biological process | 6.58E-11 | 2.17 (20634,4796,121,61) |
| GO:0010033 | response to organic substance | 7.15E-11 | 3.46 (20634,1674,121,34) |
| GO:0002687 | positive regulation of leukocyte migration | 9.13E-11 | 16.03 (20634,117,121,11) |
| GO:0048522 | positive regulation of cellular process | 9.85E-11 | 2.23 (20634,4439,121,58) |
| GO:0002252 | immune effector process | 1.20E-10 | 8.17 (20634,334,121,16) |
| GO:0031325 | positive regulation of cellular metabolic process | 2.02E-10 | 2.74 (20634,2680,121,43) |
| GO:0030335 | positive regulation of cell migration | 2.43E-10 | 7.14 (20634,406,121,17) |
| GO:0023051 | regulation of signaling | 3.05E-10 | 2.75 (20634,2603,121,42) |
| GO:0042221 | response to chemical | 3.43E-10 | 3.04 (20634,2077,121,37) |
| GO:0051239 | regulation of multicellular organismal process | 3.44E-10 | 2.74 (20634,2613,121,42) |
| GO:0051704 | multi-organism process | 3.74E-10 | 5.31 (20634,675,121,21) |
| GO:2000147 | positive regulation of cell motility | 3.80E-10 | 6.94 (20634,418,121,17) |
| GO:0032268 | regulation of cellular protein metabolic process | 4.02E-10 | 2.95 (20634,2193,121,38) |
| GO:0030595 | leukocyte chemotaxis | 4.04E-10 | 16.88 (20634,101,121,10) |
| GO:0010646 | regulation of cell communication | 5.58E-10 | 2.70 (20634,2654,121,42) |
| GO:0051272 | positive regulation of cellular component movement | 5.65E-10 | 6.76 (20634,429,121,17) |
| GO:0019220 | regulation of phosphate metabolic process | 7.04E-10 | 3.45 (20634,1534,121,31) |
| GO:0051174 | regulation of phosphorus metabolic process | 7.62E-10 | 3.43 (20634,1539,121,31) |
| GO:0040017 | positive regulation of locomotion | 7.75E-10 | 6.62 (20634,438,121,17) |
| GO:0050900 | leukocyte migration | 8.62E-10 | 13.03 (20634,144,121,11) |
| GO:1903034 | regulation of response to wounding | 1.11E-09 | 7.01 (20634,389,121,16) |
| GO:0051246 | regulation of protein metabolic process | 1.12E-09 | 2.79 (20634,2381,121,39) |
| GO:0050789 | regulation of biological process | 1.18E-09 | 1.54 (20634,10328,121,93) |
| GO:0030593 | neutrophil chemotaxis | 1.24E-09 | 24.36 (20634,56,121,8) |
| GO:0032103 | positive regulation of response to external stimulus | 1.29E-09 | 8.41 (20634,284,121,14) |
| GO:0019221 | cytokine-mediated signaling pathway | 1.36E-09 | 9.39 (20634,236,121,13) |
| GO:0009967 | positive regulation of signal transduction | 1.44E-09 | 3.80 (20634,1213,121,27) |
| GO:0071310 | cellular response to organic substance | 1.58E-09 | 4.25 (20634,964,121,24) |
| GO:0042325 | regulation of phosphorylation | 1.90E-09 | 3.62 (20634,1318,121,28) |
| GO:0002685 | regulation of leukocyte migration | 2.02E-09 | 12.02 (20634,156,121,11) |
| GO:0010604 | positive regulation of macromolecule metabolic process | 2.08E-09 | 2.63 (20634,2656,121,41) |
| GO:1990266 | neutrophil migration | 2.19E-09 | 22.74 (20634,60,121,8) |
| GO:0030334 | regulation of cell migration | 2.63E-09 | 5.02 (20634,679,121,20) |
| GO:0009615 | response to virus | 2.82E-09 | 11.65 (20634,161,121,11) |
| GO:0051707 | response to other organism | 3.21E-09 | 6.51 (20634,419,121,16) |
| GO:0071621 | granulocyte chemotaxis | 3.27E-09 | 21.65 (20634,63,121,8) |
| GO:0043408 | regulation of MAPK cascade | 4.02E-09 | 5.19 (20634,624,121,19) |
| GO:0051270 | regulation of cellular component movement | 4.41E-09 | 4.62 (20634,775,121,21) |
| GO:0033993 | response to lipid | 4.92E-09 | 5.46 (20634,562,121,18) |
| GO:0070887 | cellular response to chemical stimulus | 5.42E-09 | 3.69 (20634,1200,121,26) |
| GO:2000145 | regulation of cell motility | 6.03E-09 | 4.78 (20634,713,121,20) |
| GO:0097530 | granulocyte migration | 6.10E-09 | 20.06 (20634,68,121,8) |
| GO:0065007 | biological regulation | 6.91E-09 | 1.48 (20634,10802,121,94) |
| GO:0031349 | positive regulation of defense response | 7.09E-09 | 9.26 (20634,221,121,12) |
| GO:0001932 | regulation of protein phosphorylation | 7.49E-09 | 3.64 (20634,1219,121,26) |
| GO:0065009 | regulation of molecular function | 7.53E-09 | 2.83 (20634,2106,121,35) |
| GO:0071347 | cellular response to interleukin-1 | 7.55E-09 | 26.53 (20634,45,121,7) |
| GO:0070098 | chemokine-mediated signaling pathway | 7.55E-09 | 26.53 (20634,45,121,7) |
| GO:1902531 | regulation of intracellular signal transduction | 8.26E-09 | 3.39 (20634,1409,121,28) |
| GO:0050776 | regulation of immune response | 1.17E-08 | 5.52 (20634,525,121,17) |
| GO:0010562 | positive regulation of phosphorus metabolic process | 1.19E-08 | 3.99 (20634,984,121,23) |

| | | | |
|---|---|---|---|
| GO:0045937 | positive regulation of phosphate metabolic process | 1.19E-08 | 3.99 (20634,984,121,23) |
| GO:0032270 | positive regulation of cellular protein metabolic process | 1.47E-08 | 3.52 (20634,1260,121,26) |
| GO:0002690 | positive regulation of leukocyte chemotaxis | 1.50E-08 | 17.95 (20634,76,121,8) |
| GO:0010574 | regulation of vascular endothelial growth factor production | 1.53E-08 | 35.28 (20634,29,121,6) |
| GO:0070372 | regulation of ERK1 and ERK2 cascade | 1.55E-08 | 8.63 (20634,237,121,12) |
| GO:0034097 | response to cytokine | 1.55E-08 | 7.67 (20634,289,121,13) |
| GO:0051247 | positive regulation of protein metabolic process | 1.55E-08 | 3.40 (20634,1356,121,27) |
| GO:0031399 | regulation of protein modification process | 1.86E-08 | 3.17 (20634,1560,121,29) |
| GO:0023056 | positive regulation of signaling | 2.18E-08 | 3.34 (20634,1378,121,27) |
| GO:0080090 | regulation of primary metabolic process | 2.27E-08 | 1.95 (20634,5085,121,58) |
| GO:0050794 | regulation of cellular process | 2.39E-08 | 1.52 (20634,9889,121,88) |
| GO:0048519 | negative regulation of biological process | 2.68E-08 | 2.07 (20634,4285,121,52) |
| GO:0051240 | positive regulation of multicellular organismal process | 2.68E-08 | 3.21 (20634,1488,121,28) |
| GO:1901700 | response to oxygen-containing compound | 2.82E-08 | 3.80 (20634,1031,121,23) |
| GO:0010647 | positive regulation of cell communication | 2.85E-08 | 3.30 (20634,1396,121,27) |
| GO:0040012 | regulation of locomotion | 2.94E-08 | 4.35 (20634,784,121,20) |
| GO:0042327 | positive regulation of phosphorylation | 3.34E-08 | 4.11 (20634,871,121,21) |
| GO:0031323 | regulation of cellular metabolic process | 3.52E-08 | 1.92 (20634,5144,121,58) |
| GO:0043410 | positive regulation of MAPK cascade | 3.87E-08 | 5.89 (20634,434,121,15) |
| GO:0070374 | positive regulation of ERK1 and ERK2 cascade | 4.80E-08 | 10.34 (20634,165,121,10) |
| GO:0097529 | myeloid leukocyte migration | 4.81E-08 | 15.50 (20634,88,121,8) |
| GO:1902533 | positive regulation of intracellular signal transduction | 5.02E-08 | 4.21 (20634,810,121,20) |
| GO:0035914 | skeletal muscle cell differentiation | 5.30E-08 | 20.23 (20634,59,121,7) |
| GO:0019222 | regulation of metabolic process | 6.24E-08 | 1.86 (20634,5509,121,60) |
| GO:0042127 | regulation of cell proliferation | 6.32E-08 | 3.17 (20634,1451,121,27) |
| GO:0040011 | locomotion | 7.18E-08 | 4.12 (20634,828,121,20) |
| GO:0048585 | negative regulation of response to stimulus | 8.06E-08 | 3.34 (20634,1276,121,25) |
| GO:0002688 | regulation of leukocyte chemotaxis | 8.09E-08 | 14.51 (20634,94,121,8) |
| GO:0070555 | response to interleukin-1 | 8.44E-08 | 18.95 (20634,63,121,7) |
| GO:0071345 | cellular response to cytokine stimulus | 1.03E-07 | 9.53 (20634,179,121,10) |
| GO:0007165 | signal transduction | 1.10E-07 | 2.01 (20634,4330,121,51) |
| GO:0050790 | regulation of catalytic activity | 1.13E-07 | 3.00 (20634,1593,121,28) |
| GO:0043065 | positive regulation of apoptotic process | 1.35E-07 | 4.98 (20634,548,121,16) |
| GO:0098542 | defense response to other organism | 1.36E-07 | 6.37 (20634,348,121,13) |
| GO:1901342 | regulation of vasculature development | 1.41E-07 | 7.98 (20634,235,121,11) |
| GO:0042981 | regulation of apoptotic process | 1.50E-07 | 3.23 (20634,1318,121,25) |
| GO:0043068 | positive regulation of programmed cell death | 1.52E-07 | 4.93 (20634,553,121,16) |
| GO:0001776 | leukocyte homeostasis | 1.60E-07 | 17.30 (20634,69,121,7) |
| GO:0043067 | regulation of programmed cell death | 1.88E-07 | 3.20 (20634,1334,121,25) |
| GO:0010575 | positive regulation of vascular endothelial growth factor production | 1.97E-07 | 37.07 (20634,23,121,5) |
| GO:0050729 | positive regulation of inflammatory response | 2.07E-07 | 12.87 (20634,106,121,8) |
| GO:2000026 | regulation of multicellular organismal development | 2.17E-07 | 2.83 (20634,1748,121,29) |
| GO:0016477 | cell migration | 2.22E-07 | 4.50 (20634,644,121,17) |
| GO:0071222 | cellular response to lipopolysaccharide | 2.52E-07 | 10.23 (20634,150,121,9) |
| GO:0060255 | regulation of macromolecule metabolic process | 2.78E-07 | 1.85 (20634,5150,121,56) |
| GO:0031348 | negative regulation of defense response | 2.82E-07 | 10.10 (20634,152,121,9) |
| GO:0051248 | negative regulation of protein metabolic process | 3.32E-07 | 3.58 (20634,999,121,21) |
| GO:0001934 | positive regulation of protein phosphorylation | 3.48E-07 | 3.91 (20634,828,121,19) |
| GO:0010942 | positive regulation of cell death | 3.72E-07 | 4.62 (20634,591,121,16) |
| GO:0071219 | cellular response to molecule of bacterial origin | 4.35E-07 | 9.59 (20634,160,121,9) |
| GO:0031401 | positive regulation of protein modification process | 4.45E-07 | 3.52 (20634,1017,121,21) |
| GO:0032269 | negative regulation of cellular protein metabolic process | 4.92E-07 | 3.65 (20634,934,121,20) |
| GO:0050921 | positive regulation of chemotaxis | 5.05E-07 | 11.46 (20634,119,121,8) |
| GO:0045765 | regulation of angiogenesis | 5.19E-07 | 8.01 (20634,213,121,10) |
| GO:0032879 | regulation of localization | 5.26E-07 | 2.44 (20634,2381,121,34) |
| GO:0050793 | regulation of developmental process | 5.41E-07 | 2.48 (20634,2269,121,33) |
| GO:0051049 | regulation of transport | 5.57E-07 | 2.77 (20634,1722,121,28) |
| GO:0050778 | positive regulation of immune response | 5.65E-07 | 6.20 (20634,330,121,12) |
| GO:0051607 | defense response to virus | 6.50E-07 | 11.09 (20634,123,121,8) |
| GO:0048247 | lymphocyte chemotaxis | 6.77E-07 | 29.40 (20634,29,121,5) |
| GO:0010941 | regulation of cell death | 8.25E-07 | 2.95 (20634,1444,121,25) |
| GO:0051241 | negative regulation of multicellular organismal process | 9.38E-07 | 3.36 (20634,1065,121,21) |
| GO:0071216 | cellular response to biotic stimulus | 1.07E-06 | 8.62 (20634,178,121,9) |
| GO:0090026 | positive regulation of monocyte chemotaxis | 1.08E-06 | 48.72 (20634,14,121,4) |
| GO:0048523 | negative regulation of cellular process | 1.15E-06 | 1.97 (20634,3972,121,46) |
| GO:0048870 | cell motility | 1.21E-06 | 3.98 (20634,728,121,17) |
| GO:0065008 | regulation of biological quality | 1.29E-06 | 2.23 (20634,2835,121,37) |
| GO:0023057 | negative regulation of signaling | 1.34E-06 | 3.29 (20634,1089,121,21) |
| GO:0009892 | negative regulation of metabolic process | 1.50E-06 | 2.37 (20634,2374,121,33) |
| GO:1904018 | positive regulation of vasculature development | 1.64E-06 | 9.81 (20634,139,121,8) |
| GO:0010648 | negative regulation of cell communication | 1.67E-06 | 3.24 (20634,1104,121,21) |
| GO:0044093 | positive regulation of molecular function | 1.94E-06 | 3.10 (20634,1211,121,22) |
| GO:0001660 | fever generation | 1.95E-06 | 102.32 (20634,5,121,3) |
| GO:1902105 | regulation of leukocyte differentiation | 2.14E-06 | 6.85 (20634,249,121,10) |
| GO:0002683 | negative regulation of immune system process | 2.15E-06 | 5.46 (20634,375,121,12) |
| GO:0045088 | regulation of innate immune response | 2.18E-06 | 7.91 (20634,194,121,9) |
| GO:0010605 | negative regulation of macromolecule metabolic process | 2.73E-06 | 2.40 (20634,2205,121,31) |
| GO:0072676 | lymphocyte migration | 2.74E-06 | 22.44 (20634,38,121,5) |
| GO:1903036 | positive regulation of response to wounding | 3.06E-06 | 9.03 (20634,151,121,8) |
| GO:0034114 | regulation of heterotypic cell-cell adhesion | 3.23E-06 | 37.90 (20634,18,121,4) |
| GO:0033554 | cellular response to stress | 3.40E-06 | 3.22 (20634,1059,121,20) |
| GO:0050728 | negative regulation of inflammatory response | 3.64E-06 | 10.95 (20634,109,121,7) |
| GO:0090025 | regulation of monocyte chemotaxis | 4.07E-06 | 35.90 (20634,19,121,4) |

| | | | |
|---|---|---|---|
| GO:0044092 | negative regulation of molecular function | 4.16E-06 | 3.46 (20634,887,121,18) |
| GO:0010628 | positive regulation of gene expression | 4.39E-06 | 2.62 (20634,1692,121,26) |
| GO:0098586 | cellular response to virus | 5.07E-06 | 34.11 (20634,20,121,4) |
| GO:1903706 | regulation of hemopoiesis | 5.41E-06 | 5.50 (20634,341,121,11) |
| GO:0007166 | cell surface receptor signaling pathway | 5.66E-06 | 2.73 (20634,1501,121,24) |
| GO:0032680 | regulation of tumor necrosis factor production | 5.83E-06 | 10.20 (20634,117,121,7) |
| GO:1903035 | negative regulation of response to wounding | 5.90E-06 | 8.27 (20634,165,121,8) |
| GO:0042326 | negative regulation of phosphorylation | 6.07E-06 | 4.93 (20634,415,121,12) |
| GO:1903555 | regulation of tumor necrosis factor superfamily cytokine production | 6.52E-06 | 10.03 (20634,119,121,7) |
| GO:0032102 | negative regulation of response to external stimulus | 6.66E-06 | 6.03 (20634,283,121,10) |
| GO:0031324 | negative regulation of cellular metabolic process | 6.80E-06 | 2.34 (20634,2187,121,30) |
| GO:0043085 | positive regulation of catalytic activity | 6.88E-06 | 3.34 (20634,920,121,18) |
| GO:0035690 | cellular response to drug | 7.22E-06 | 18.54 (20634,46,121,5) |
| GO:0071346 | cellular response to interferon-gamma | 8.04E-06 | 18.14 (20634,47,121,5) |
| GO:0051336 | regulation of hydrolase activity | 8.20E-06 | 3.45 (20634,840,121,17) |
| GO:0051050 | positive regulation of transport | 8.97E-06 | 3.27 (20634,938,121,18) |
| GO:0045766 | positive regulation of angiogenesis | 9.01E-06 | 9.55 (20634,125,121,7) |
| GO:0048660 | regulation of smooth muscle cell proliferation | 9.01E-06 | 9.55 (20634,125,121,7) |
| GO:0010629 | negative regulation of gene expression | 1.02E-05 | 2.71 (20634,1448,121,23) |
| GO:1901724 | positive regulation of cell proliferation involved in kidney development | 1.08E-05 | 63.95 (20634,8,121,3) |
| GO:0031665 | negative regulation of lipopolysaccharide-mediated signaling pathway | 1.08E-05 | 63.95 (20634,8,121,3) |
| GO:0031649 | heat generation | 1.08E-05 | 63.95 (20634,8,121,3) |
| GO:0009611 | response to wounding | 1.11E-05 | 9.25 (20634,129,121,7) |
| GO:0050920 | regulation of chemotaxis | 1.12E-05 | 7.58 (20634,180,121,8) |
| GO:0045087 | innate immune response | 1.14E-05 | 5.08 (20634,369,121,11) |
| GO:0022603 | regulation of anatomical structure morphogenesis | 1.19E-05 | 3.20 (20634,958,121,18) |
| GO:0009968 | negative regulation of signal transduction | 1.30E-05 | 3.18 (20634,964,121,18) |
| GO:0001933 | negative regulation of protein phosphorylation | 1.35E-05 | 4.99 (20634,376,121,11) |
| GO:0050865 | regulation of cell activation | 1.40E-05 | 4.54 (20634,451,121,12) |
| GO:0010563 | negative regulation of phosphorus metabolic process | 1.43E-05 | 4.18 (20634,530,121,13) |
| GO:0045936 | negative regulation of phosphate metabolic process | 1.43E-05 | 4.18 (20634,530,121,13) |
| GO:0002697 | regulation of immune effector process | 1.61E-05 | 4.90 (20634,383,121,11) |
| GO:1901701 | cellular response to oxygen-containing compound | 1.72E-05 | 3.84 (20634,622,121,14) |
| GO:0043066 | negative regulation of apoptotic process | 1.82E-05 | 3.41 (20634,801,121,16) |
| GO:0060548 | negative regulation of cell death | 1.84E-05 | 3.24 (20634,894,121,17) |
| GO:0071356 | cellular response to tumor necrosis factor | 2.09E-05 | 14.96 (20634,57,121,5) |
| GO:0043086 | negative regulation of catalytic activity | 2.13E-05 | 3.77 (20634,634,121,14) |
| GO:1902107 | positive regulation of leukocyte differentiation | 2.17E-05 | 8.35 (20634,143,121,7) |
| GO:0097190 | apoptotic signaling pathway | 2.17E-05 | 5.95 (20634,258,121,9) |
| GO:0043069 | negative regulation of programmed cell death | 2.25E-05 | 3.35 (20634,815,121,16) |
| GO:0002260 | lymphocyte homeostasis | 2.28E-05 | 14.70 (20634,58,121,5) |
| GO:0002507 | tolerance induction | 2.29E-05 | 51.16 (20634,10,121,3) |
| GO:0051173 | positive regulation of nitrogen compound metabolic process | 2.29E-05 | 2.44 (20634,1744,121,25) |
| GO:0002764 | immune response-regulating signaling pathway | 2.37E-05 | 8.23 (20634,145,121,7) |
| GO:0001659 | temperature homeostasis | 2.39E-05 | 23.52 (20634,29,121,4) |
| GO:0002832 | negative regulation of response to biotic stimulus | 2.39E-05 | 23.52 (20634,29,121,4) |
| GO:0002460 | adaptive immune response based on somatic recombination of immune receptors built from immunoglobulin superfamily domains | 2.48E-05 | 14.45 (20634,59,121,5) |
| GO:0006469 | negative regulation of protein kinase activity | 2.57E-05 | 6.75 (20634,202,121,8) |
| GO:0043547 | positive regulation of GTPase activity | 2.66E-05 | 6.72 (20634,203,121,8) |
| GO:0002831 | regulation of response to biotic stimulus | 2.66E-05 | 6.72 (20634,203,121,8) |
| GO:0051172 | negative regulation of nitrogen compound metabolic process | 2.66E-05 | 2.63 (20634,1429,121,22) |
| GO:0043901 | negative regulation of multi-organism process | 2.70E-05 | 8.07 (20634,148,121,7) |
| GO:0006928 | movement of cell or subcellular component | 2.97E-05 | 2.99 (20634,1026,121,18) |
| GO:0010558 | negative regulation of macromolecule biosynthetic process | 3.02E-05 | 2.68 (20634,1334,121,21) |
| GO:0048872 | homeostasis of number of cells | 3.08E-05 | 7.91 (20634,151,121,7) |
| GO:1902624 | positive regulation of neutrophil migration | 3.13E-05 | 22.00 (20634,31,121,4) |
| GO:0043900 | regulation of multi-organism process | 3.14E-05 | 4.55 (20634,412,121,11) |
| GO:0002694 | regulation of leukocyte activation | 3.29E-05 | 4.53 (20634,414,121,11) |
| GO:0050707 | regulation of cytokine secretion | 3.35E-05 | 7.80 (20634,153,121,7) |
| GO:0072573 | tolerance induction to lipopolysaccharide | 3.41E-05 | 170.53 (20634,2,121,2) |
| GO:0050715 | positive regulation of cytokine secretion | 3.60E-05 | 9.74 (20634,105,121,6) |
| GO:0051171 | regulation of nitrogen compound metabolic process | 3.92E-05 | 1.85 (20634,3683,121,40) |
| GO:0001782 | B cell homeostasis | 4.04E-05 | 20.67 (20634,33,121,4) |
| GO:0010468 | regulation of gene expression | 4.10E-05 | 1.85 (20634,3690,121,40) |
| GO:0019219 | regulation of nucleobase-containing compound metabolic process | 4.32E-05 | 1.89 (20634,3430,121,38) |
| GO:0006953 | acute-phase response | 4.55E-05 | 20.06 (20634,34,121,4) |
| GO:0034341 | response to interferon-gamma | 4.61E-05 | 12.73 (20634,67,121,5) |
| GO:0002443 | leukocyte mediated immunity | 4.61E-05 | 12.73 (20634,67,121,5) |
| GO:0033673 | negative regulation of kinase activity | 4.71E-05 | 6.20 (20634,220,121,8) |
| GO:0044699 | single-organism process | 4.96E-05 | 1.37 (20634,10116,121,81) |
| GO:0035456 | response to interferon-beta | 5.12E-05 | 19.49 (20634,35,121,4) |
| GO:0031327 | negative regulation of cellular biosynthetic process | 5.21E-05 | 2.59 (20634,1385,121,21) |
| GO:2000113 | negative regulation of cellular macromolecule biosynthetic process | 5.26E-05 | 2.67 (20634,1279,121,20) |
| GO:0042592 | homeostatic process | 5.30E-05 | 2.76 (20634,1175,121,19) |
| GO:0002377 | immunoglobulin production | 5.39E-05 | 39.35 (20634,13,121,3) |
| GO:0038034 | signal transduction in absence of ligand | 5.73E-05 | 18.95 (20634,36,121,4) |
| GO:0097192 | extrinsic apoptotic signaling pathway in absence of ligand | 5.73E-05 | 18.95 (20634,36,121,4) |
| GO:0045934 | negative regulation of nucleobase-containing compound metabolic process | 5.73E-05 | 2.65 (20634,1287,121,20) |
| GO:0051345 | positive regulation of hydrolase activity | 5.93E-05 | 4.24 (20634,442,121,11) |
| GO:0051817 | modification of morphology or physiology of other organism involved in symbiotic interaction | 6.10E-05 | 12.01 (20634,71,121,5) |
| GO:1902622 | regulation of neutrophil migration | 6.40E-05 | 18.44 (20634,37,121,4) |
| GO:0035821 | modification of morphology or physiology of other organism | 6.52E-05 | 11.84 (20634,72,121,5) |

| | | | |
|---|---|---|---|
| GO:1901722 | regulation of cell proliferation involved in kidney development | 6.83E-05 | 36.54 (20634,14,121,3) |
| GO:0009890 | negative regulation of biosynthetic process | 6.94E-05 | 2.53 (20634,1413,121,21) |
| GO:0042493 | response to drug | 7.57E-05 | 6.86 (20634,174,121,7) |
| GO:0008284 | positive regulation of cell proliferation | 7.78E-05 | 3.17 (20634,808,121,15) |
| GO:0051223 | regulation of protein transport | 7.82E-05 | 3.34 (20634,715,121,14) |
| GO:0009889 | regulation of biosynthetic process | 7.90E-05 | 1.82 (20634,3660,121,39) |
| GO:0034612 | response to tumor necrosis factor | 7.94E-05 | 11.37 (20634,75,121,5) |
| GO:0002253 | activation of immune response | 8.13E-05 | 6.78 (20634,176,121,7) |
| GO:0007611 | learning or memory | 8.18E-05 | 5.73 (20634,238,121,8) |
| GO:0045935 | positive regulation of nucleobase-containing compound metabolic process | 8.33E-05 | 2.37 (20634,1655,121,23) |
| GO:0050777 | negative regulation of immune response | 8.34E-05 | 8.39 (20634,122,121,6) |
| GO:1903708 | positive regulation of hemopoiesis | 8.43E-05 | 6.74 (20634,177,121,7) |
| GO:0008630 | intrinsic apoptotic signaling pathway in response to DNA damage | 8.46E-05 | 11.22 (20634,76,121,5) |
| GO:0032727 | positive regulation of interferon-alpha production | 8.50E-05 | 34.11 (20634,15,121,3) |
| GO:0031400 | negative regulation of protein modification process | 8.62E-05 | 3.76 (20634,544,121,12) |
| GO:0043618 | regulation of transcription from RNA polymerase II promoter in response to stress | 8.73E-05 | 17.05 (20634,40,121,4) |
| GO:0045824 | negative regulation of innate immune response | 8.73E-05 | 17.05 (20634,40,121,4) |
| GO:1902679 | negative regulation of RNA biosynthetic process | 8.91E-05 | 2.75 (20634,1117,121,18) |
| GO:0071396 | cellular response to lipid | 9.00E-05 | 4.95 (20634,310,121,9) |
| GO:0050708 | regulation of protein secretion | 9.98E-05 | 4.38 (20634,389,121,10) |
| GO:0070487 | monocyte aggregation | 1.02E-04 | 113.69 (20634,3,121,2) |
| GO:0044763 | single-organism cellular process | 1.05E-04 | 1.42 (20634,8409,121,70) |
| GO:0071622 | regulation of granulocyte chemotaxis | 1.06E-04 | 16.24 (20634,42,121,4) |
| GO:0051094 | positive regulation of developmental process | 1.12E-04 | 2.61 (20634,1243,121,19) |
| GO:0051348 | negative regulation of transferase activity | 1.15E-04 | 5.46 (20634,250,121,8) |
| GO:0002819 | regulation of adaptive immune response | 1.18E-04 | 7.87 (20634,130,121,6) |
| GO:0006357 | regulation of transcription from RNA polymerase II promoter | 1.21E-04 | 2.31 (20634,1697,121,23) |
| GO:0048661 | positive regulation of smooth muscle cell proliferation | 1.21E-04 | 10.40 (20634,82,121,5) |
| GO:0045637 | regulation of myeloid cell differentiation | 1.23E-04 | 6.35 (20634,188,121,7) |
| GO:0009719 | response to endogenous stimulus | 1.24E-04 | 3.03 (20634,843,121,15) |
| GO:0031326 | regulation of cellular biosynthetic process | 1.26E-04 | 1.80 (20634,3601,121,38) |
| GO:0043620 | regulation of DNA-templated transcription in response to stress | 1.27E-04 | 15.50 (20634,44,121,4) |
| GO:0002757 | immune response-activating signal transduction | 1.40E-04 | 7.64 (20634,134,121,6) |
| GO:0051253 | negative regulation of RNA metabolic process | 1.43E-04 | 2.65 (20634,1160,121,18) |
| GO:0032647 | regulation of interferon-alpha production | 1.51E-04 | 28.42 (20634,18,121,3) |
| GO:0031664 | regulation of lipopolysaccharide-mediated signaling pathway | 1.51E-04 | 28.42 (20634,18,121,3) |
| GO:0034121 | regulation of toll-like receptor signaling pathway | 1.52E-04 | 14.83 (20634,46,121,4) |
| GO:0070201 | regulation of establishment of protein localization | 1.72E-04 | 3.10 (20634,771,121,14) |
| GO:0050890 | cognition | 1.76E-04 | 5.13 (20634,266,121,8) |
| GO:0042107 | cytokine metabolic process | 1.78E-04 | 26.93 (20634,19,121,3) |
| GO:0043269 | regulation of ion transport | 1.78E-04 | 3.48 (20634,588,121,12) |
| GO:0043405 | regulation of MAP kinase activity | 1.90E-04 | 5.07 (20634,269,121,8) |
| GO:0043087 | regulation of GTPase activity | 1.90E-04 | 5.07 (20634,269,121,8) |
| GO:0009891 | positive regulation of biosynthetic process | 1.93E-04 | 2.24 (20634,1751,121,23) |
| GO:0008285 | negative regulation of cell proliferation | 2.02E-04 | 3.43 (20634,596,121,12) |
| GO:2000562 | negative regulation of CD4-positive, alpha-beta T cell proliferation | 2.03E-04 | 85.26 (20634,4,121,2) |
| GO:0039528 | cytoplasmic pattern recognition receptor signaling pathway in response to virus | 2.03E-04 | 85.26 (20634,4,121,2) |
| GO:0019087 | transformation of host cell by virus | 2.03E-04 | 85.26 (20634,4,121,2) |
| GO:0002449 | lymphocyte mediated immunity | 2.10E-04 | 13.64 (20634,50,121,4) |
| GO:0045892 | negative regulation of transcription, DNA-templated | 2.12E-04 | 2.66 (20634,1091,121,17) |
| GO:0098609 | cell-cell adhesion | 2.16E-04 | 3.66 (20634,512,121,11) |
| GO:0032663 | regulation of interleukin-2 production | 2.27E-04 | 13.37 (20634,51,121,4) |
| GO:1903507 | negative regulation of nucleic acid-templated transcription | 2.44E-04 | 2.63 (20634,1104,121,17) |
| GO:0010556 | regulation of macromolecule biosynthetic process | 2.48E-04 | 1.78 (20634,3444,121,36) |
| GO:0051249 | regulation of lymphocyte activation | 2.49E-04 | 4.32 (20634,355,121,9) |
| GO:0009987 | cellular process | 2.52E-04 | 1.29 (20634,11404,121,86) |
| GO:0051254 | positive regulation of RNA metabolic process | 2.68E-04 | 2.36 (20634,1443,121,20) |
| GO:0019724 | B cell mediated immunity | 2.79E-04 | 23.25 (20634,22,121,3) |
| GO:0002763 | positive regulation of myeloid leukocyte differentiation | 2.84E-04 | 12.63 (20634,54,121,4) |
| GO:0010634 | positive regulation of epithelial cell migration | 2.94E-04 | 8.61 (20634,99,121,5) |
| GO:2001233 | regulation of apoptotic signaling pathway | 2.99E-04 | 4.22 (20634,364,121,9) |
| GO:0032677 | regulation of interleukin-8 production | 3.05E-04 | 12.40 (20634,55,121,4) |
| GO:0010893 | positive regulation of steroid biosynthetic process | 3.20E-04 | 22.24 (20634,23,121,3) |
| GO:0032479 | regulation of type I interferon production | 3.27E-04 | 12.18 (20634,56,121,4) |
| GO:0051222 | positive regulation of protein transport | 3.28E-04 | 3.78 (20634,451,121,10) |
| GO:2000561 | regulation of CD4-positive, alpha-beta T cell proliferation | 3.37E-04 | 68.21 (20634,5,121,2) |
| GO:0045351 | type I interferon biosynthetic process | 3.37E-04 | 68.21 (20634,5,121,2) |
| GO:1990441 | negative regulation of transcription from RNA polymerase II promoter in response to endoplasmic reticulum stress | 3.37E-04 | 68.21 (20634,5,121,2) |
| GO:2000427 | positive regulation of apoptotic cell clearance | 3.37E-04 | 68.21 (20634,5,121,2) |
| GO:0060341 | regulation of cellular localization | 3.40E-04 | 2.47 (20634,1245,121,18) |
| GO:0002526 | acute inflammatory response | 3.50E-04 | 11.97 (20634,57,121,4) |
| GO:0051252 | regulation of RNA metabolic process | 3.57E-04 | 1.81 (20634,3101,121,33) |
| GO:0034122 | negative regulation of toll-like receptor signaling pathway | 3.64E-04 | 21.32 (20634,24,121,3) |
| GO:0031328 | positive regulation of cellular biosynthetic process | 3.84E-04 | 2.18 (20634,1717,121,22) |
| GO:0002703 | regulation of leukocyte mediated immunity | 3.90E-04 | 6.32 (20634,162,121,6) |
| GO:0001775 | cell activation | 3.90E-04 | 3.70 (20634,461,121,10) |
| GO:0010557 | positive regulation of macromolecule biosynthetic process | 3.99E-04 | 2.23 (20634,1604,121,21) |
| GO:0007155 | cell adhesion | 4.11E-04 | 2.84 (20634,840,121,14) |
| GO:0090023 | positive regulation of neutrophil chemotaxis | 4.12E-04 | 20.46 (20634,25,121,3) |
| GO:0071900 | regulation of protein serine/threonine kinase activity | 4.17E-04 | 4.03 (20634,381,121,9) |
| GO:2001141 | regulation of RNA biosynthetic process | 4.29E-04 | 1.82 (20634,2998,121,32) |
| GO:0034112 | positive regulation of homotypic cell-cell adhesion | 4.44E-04 | 6.16 (20634,166,121,6) |

| | | | |
|---|---|---|---|
| GO:0051051 | negative regulation of transport | 4.47E-04 | 3.64 (20634,469,121,10) |
| GO:0022610 | biological adhesion | 4.63E-04 | 2.81 (20634,850,121,14) |
| GO:0002440 | production of molecular mediator of immune response | 4.63E-04 | 19.68 (20634,26,121,3) |
| GO:2000178 | negative regulation of neural precursor cell proliferation | 4.63E-04 | 19.68 (20634,26,121,3) |
| GO:0002761 | regulation of myeloid leukocyte differentiation | 4.78E-04 | 7.75 (20634,110,121,5) |
| GO:1903039 | positive regulation of leukocyte cell-cell adhesion | 5.04E-04 | 6.02 (20634,170,121,6) |
| GO:0072126 | positive regulation of glomerular mesangial cell proliferation | 5.04E-04 | 56.84 (20634,6,121,2) |
| GO:0031915 | positive regulation of synaptic plasticity | 5.04E-04 | 56.84 (20634,6,121,2) |
| GO:0009896 | positive regulation of catabolic process | 5.13E-04 | 4.37 (20634,312,121,8) |
| GO:0071624 | positive regulation of granulocyte chemotaxis | 5.19E-04 | 18.95 (20634,27,121,3) |
| GO:0002548 | monocyte chemotaxis | 5.19E-04 | 18.95 (20634,27,121,3) |
| GO:0002902 | regulation of B cell apoptotic process | 5.19E-04 | 18.95 (20634,27,121,3) |
| GO:0007613 | memory | 5.19E-04 | 7.61 (20634,112,121,5) |
| GO:0051093 | negative regulation of developmental process | 5.25E-04 | 2.77 (20634,861,121,14) |
| GO:0002250 | adaptive immune response | 5.69E-04 | 5.88 (20634,174,121,6) |
| GO:0002675 | positive regulation of acute inflammatory response | 5.79E-04 | 18.27 (20634,28,121,3) |
| GO:0032728 | positive regulation of interferon-beta production | 5.79E-04 | 18.27 (20634,28,121,3) |
| GO:2000403 | positive regulation of lymphocyte migration | 5.79E-04 | 18.27 (20634,28,121,3) |
| GO:0046640 | regulation of alpha-beta T cell proliferation | 5.79E-04 | 18.27 (20634,28,121,3) |
| GO:1904951 | positive regulation of establishment of protein localization | 5.79E-04 | 3.52 (20634,485,121,10) |
| GO:1901698 | response to nitrogen compound | 5.91E-04 | 3.25 (20634,577,121,11) |
| GO:0032675 | regulation of interleukin-6 production | 6.09E-04 | 7.35 (20634,116,121,5) |
| GO:0046330 | positive regulation of JNK cascade | 6.09E-04 | 7.35 (20634,116,121,5) |
| GO:0008219 | cell death | 6.09E-04 | 3.04 (20634,674,121,12) |
| GO:0045859 | regulation of protein kinase activity | 6.17E-04 | 3.23 (20634,580,121,11) |
| GO:0045321 | leukocyte activation | 6.35E-04 | 3.80 (20634,404,121,9) |
| GO:0035458 | cellular response to interferon-beta | 6.43E-04 | 17.64 (20634,29,121,3) |
| GO:0032388 | positive regulation of intracellular transport | 6.43E-04 | 4.22 (20634,323,121,8) |
| GO:2000112 | regulation of cellular macromolecule biosynthetic process | 6.52E-04 | 1.73 (20634,3342,121,34) |
| GO:0032760 | positive regulation of tumor necrosis factor production | 6.86E-04 | 10.03 (20634,68,121,4) |
| GO:0002696 | positive regulation of leukocyte activation | 6.89E-04 | 4.77 (20634,250,121,7) |
| GO:0002759 | regulation of antimicrobial humoral response | 7.03E-04 | 48.72 (20634,7,121,2) |
| GO:0034144 | negative regulation of toll-like receptor 4 signaling pathway | 7.03E-04 | 48.72 (20634,7,121,2) |
| GO:2000425 | regulation of apoptotic cell clearance | 7.03E-04 | 48.72 (20634,7,121,2) |
| GO:2000108 | positive regulation of leukocyte apoptotic process | 7.11E-04 | 17.05 (20634,30,121,3) |
| GO:0090022 | regulation of neutrophil chemotaxis | 7.11E-04 | 17.05 (20634,30,121,3) |
| GO:0016337 | single organismal cell-cell adhesion | 7.17E-04 | 3.73 (20634,411,121,9) |
| GO:0043525 | positive regulation of neuron apoptotic process | 7.25E-04 | 9.89 (20634,69,121,4) |
| GO:0070228 | regulation of lymphocyte apoptotic process | 7.25E-04 | 9.89 (20634,69,121,4) |
| GO:1903557 | positive regulation of tumor necrosis factor superfamily cytokine production | 7.25E-04 | 9.89 (20634,69,121,4) |
| GO:0007159 | leukocyte cell-cell adhesion | 7.57E-04 | 4.70 (20634,254,121,7) |
| GO:0014910 | regulation of smooth muscle cell migration | 7.65E-04 | 9.74 (20634,70,121,4) |
| GO:0031331 | positive regulation of cellular catabolic process | 7.74E-04 | 4.68 (20634,255,121,7) |
| GO:0045940 | positive regulation of steroid metabolic process | 7.84E-04 | 16.50 (20634,31,121,3) |
| GO:0032743 | positive regulation of interleukin-2 production | 7.84E-04 | 16.50 (20634,31,121,3) |
| GO:0032755 | positive regulation of interleukin-6 production | 8.07E-04 | 9.61 (20634,71,121,4) |
| GO:0030155 | regulation of cell adhesion | 8.26E-04 | 3.12 (20634,601,121,11) |
| GO:0006355 | regulation of transcription, DNA-templated | 8.27E-04 | 1.78 (20634,2977,121,31) |
| GO:0043270 | positive regulation of ion transport | 8.29E-04 | 4.63 (20634,258,121,7) |
| GO:0045944 | positive regulation of transcription from RNA polymerase II promoter | 8.32E-04 | 2.54 (20634,1009,121,15) |
| GO:0001562 | response to protozoan | 8.62E-04 | 15.99 (20634,32,121,3) |
| GO:0043122 | regulation of I-kappaB kinase/NF-kappaB signaling | 8.77E-04 | 5.41 (20634,189,121,6) |
| GO:1903506 | regulation of nucleic acid-templated transcription | 8.95E-04 | 1.77 (20634,2991,121,31) |
| GO:0014070 | response to organic cyclic compound | 9.17E-04 | 3.31 (20634,515,121,10) |
| GO:0050867 | positive regulation of cell activation | 9.27E-04 | 4.54 (20634,263,121,7) |
| GO:0002315 | marginal zone B cell differentiation | 9.33E-04 | 42.63 (20634,8,121,2) |
| GO:0034340 | response to type I interferon | 9.33E-04 | 42.63 (20634,8,121,2) |
| GO:0002753 | cytoplasmic pattern recognition receptor signaling pathway | 9.33E-04 | 42.63 (20634,8,121,2) |
| GO:0034115 | negative regulation of heterotypic cell-cell adhesion | 9.33E-04 | 42.63 (20634,8,121,2) |
| GO:0045123 | cellular extravasation | 9.33E-04 | 42.63 (20634,8,121,2) |
| GO:0070587 | regulation of cell-cell adhesion involved in gastrulation | 9.33E-04 | 42.63 (20634,8,121,2) |
| GO:0051547 | regulation of keratinocyte migration | 9.33E-04 | 42.63 (20634,8,121,2) |
| GO:0051549 | positive regulation of keratinocyte migration | 9.33E-04 | 42.63 (20634,8,121,2) |
| GO:0000185 | activation of MAPKKK activity | 9.33E-04 | 42.63 (20634,8,121,2) |
| GO:0046642 | negative regulation of alpha-beta T cell proliferation | 9.33E-04 | 42.63 (20634,8,121,2) |
| GO:0002920 | regulation of humoral immune response | 9.44E-04 | 15.50 (20634,33,121,3) |
| GO:0043271 | negative regulation of ion transport | 9.84E-04 | 6.61 (20634,129,121,5) |

| Downregulated genes | | | |
|---|---|---|---|
| **Goterm** | **Description** | **P-value** | **Enrichment (N, B, n, b)** |
| GO:0006412 | translation | 8.45E-20 | 7.08 (20634,309,330,35) |
| GO:0006334 | nucleosome assembly | 1.39E-19 | 14.33 (20634,96,330,22) |
| GO:0043043 | peptide biosynthetic process | 5.32E-19 | 6.69 (20634,327,330,35) |
| GO:0034728 | nucleosome organization | 1.01E-18 | 12.19 (20634,118,330,23) |
| GO:0065004 | protein-DNA complex assembly | 6.94E-17 | 10.92 (20634,126,330,22) |
| GO:0043604 | amide biosynthetic process | 1.95E-16 | 5.55 (20634,394,330,35) |
| GO:0071824 | protein-DNA complex subunit organization | 1.97E-16 | 9.72 (20634,148,330,23) |
| GO:0006518 | peptide metabolic process | 3.98E-16 | 5.26 (20634,428,330,36) |
| GO:0034622 | cellular macromolecular complex assembly | 4.43E-16 | 4.68 (20634,534,330,40) |
| GO:1901566 | organonitrogen compound biosynthetic process | 3.15E-14 | 3.71 (20634,759,330,45) |
| GO:1901564 | organonitrogen compound metabolic process | 5.70E-13 | 2.79 (20634,1322,330,59) |
| GO:0043603 | cellular amide metabolic process | 9.70E-13 | 4.07 (20634,553,330,36) |

| | | | |
|---|---|---|---|
| GO:0006325 | chromatin organization | 1.09E-12 | 3.95 (20634,585,330,37) |
| GO:0045653 | negative regulation of megakaryocyte differentiation | 1.33E-12 | 33.10 (20634,17,330,9) |
| GO:0034723 | DNA replication-dependent nucleosome organization | 2.16E-11 | 20.84 (20634,30,330,10) |
| GO:0006335 | DNA replication-dependent nucleosome assembly | 2.16E-11 | 20.84 (20634,30,330,10) |
| GO:1901533 | negative regulation of hematopoietic progenitor cell differentiation | 4.12E-11 | 24.47 (20634,23,330,9) |
| GO:0043933 | macromolecular complex subunit organization | 1.17E-10 | 2.28 (20634,1840,330,67) |
| GO:0045652 | regulation of megakaryocyte differentiation | 2.23E-10 | 20.84 (20634,27,330,9) |
| GO:0006333 | chromatin assembly or disassembly | 2.42E-10 | 12.10 (20634,62,330,12) |
| GO:0065003 | macromolecular complex assembly | 2.50E-10 | 2.73 (20634,1101,330,48) |
| GO:0051290 | protein heterotetramerization | 4.03E-10 | 16.03 (20634,39,330,10) |
| GO:0034724 | DNA replication-independent nucleosome organization | 4.78E-09 | 19.24 (20634,26,330,8) |
| GO:0006336 | DNA replication-independent nucleosome assembly | 4.78E-09 | 19.24 (20634,26,330,8) |
| GO:0045638 | negative regulation of myeloid cell differentiation | 1.06E-08 | 8.83 (20634,85,330,12) |
| GO:1901532 | regulation of hematopoietic progenitor cell differentiation | 3.28E-08 | 12.51 (20634,45,330,9) |
| GO:0006461 | protein complex assembly | 1.06E-07 | 2.61 (20634,887,330,37) |
| GO:0000028 | ribosomal small subunit assembly | 1.12E-07 | 23.45 (20634,16,330,6) |
| GO:0022607 | cellular component assembly | 2.51E-07 | 2.07 (20634,1633,330,54) |
| GO:0051262 | protein tetramerization | 3.47E-07 | 5.89 (20634,138,330,13) |
| GO:0071822 | protein complex subunit organization | 4.94E-07 | 2.26 (20634,1190,330,43) |
| GO:1903707 | negative regulation of hemopoiesis | 1.22E-06 | 5.77 (20634,130,330,12) |
| GO:0009205 | purine ribonucleoside triphosphate metabolic process | 1.69E-06 | 6.20 (20634,111,330,11) |
| GO:0009141 | nucleoside triphosphate metabolic process | 1.98E-06 | 5.52 (20634,136,330,12) |
| GO:0009199 | ribonucleoside triphosphate metabolic process | 2.20E-06 | 6.03 (20634,114,330,11) |
| GO:0019693 | ribose phosphate metabolic process | 3.31E-06 | 4.21 (20634,223,330,15) |
| GO:0009144 | purine nucleoside triphosphate metabolic process | 3.36E-06 | 5.78 (20634,119,330,11) |
| GO:0045637 | regulation of myeloid cell differentiation | 1.11E-05 | 4.32 (20634,188,330,13) |
| GO:0051291 | protein heterooligomerization | 1.14E-05 | 5.09 (20634,135,330,11) |
| GO:0046034 | ATP metabolic process | 1.52E-05 | 6.18 (20634,91,330,9) |
| GO:0009142 | nucleoside triphosphate biosynthetic process | 1.81E-05 | 8.42 (20634,52,330,7) |
| GO:0006342 | chromatin silencing | 1.93E-05 | 6.95 (20634,72,330,8) |
| GO:0009117 | nucleotide metabolic process | 1.95E-05 | 3.29 (20634,323,330,17) |
| GO:0046128 | purine ribonucleoside metabolic process | 2.34E-05 | 4.34 (20634,173,330,12) |
| GO:0009150 | purine ribonucleotide metabolic process | 2.64E-05 | 3.98 (20634,204,330,13) |
| GO:0009116 | nucleoside metabolic process | 2.78E-05 | 3.97 (20634,205,330,13) |
| GO:0034641 | cellular nitrogen compound metabolic process | 2.87E-05 | 1.48 (20634,3920,330,93) |
| GO:0045814 | negative regulation of gene expression, epigenetic | 2.88E-05 | 6.58 (20634,76,330,8) |
| GO:0042278 | purine nucleoside metabolic process | 2.93E-05 | 4.24 (20634,177,330,12) |
| GO:0006753 | nucleoside phosphate metabolic process | 3.10E-05 | 3.17 (20634,335,330,17) |
| GO:0009206 | purine ribonucleoside triphosphate biosynthetic process | 3.34E-05 | 9.62 (20634,39,330,6) |
| GO:0009145 | purine nucleoside triphosphate biosynthetic process | 3.88E-05 | 9.38 (20634,40,330,6) |
| GO:0009259 | ribonucleotide metabolic process | 3.95E-05 | 3.83 (20634,212,330,13) |
| GO:0009201 | ribonucleoside triphosphate biosynthetic process | 4.49E-05 | 9.15 (20634,41,330,6) |
| GO:0008152 | metabolic process | 4.62E-05 | 1.28 (20634,7689,330,158) |
| GO:0006163 | purine nucleotide metabolic process | 4.79E-05 | 3.76 (20634,216,330,13) |
| GO:0006807 | nitrogen compound metabolic process | 5.52E-05 | 1.44 (20634,4204,330,97) |
| GO:0022618 | ribonucleoprotein complex assembly | 5.54E-05 | 4.30 (20634,160,330,11) |
| GO:0006364 | rRNA processing | 5.86E-05 | 4.27 (20634,161,330,11) |
| GO:0055086 | nucleobase-containing small molecule metabolic process | 5.87E-05 | 2.90 (20634,388,330,18) |
| GO:0044271 | cellular nitrogen compound biosynthetic process | 6.33E-05 | 1.61 (20634,2523,330,65) |
| GO:1901657 | glycosyl compound metabolic process | 6.34E-05 | 3.66 (20634,222,330,13) |
| GO:0034470 | ncRNA processing | 6.49E-05 | 3.27 (20634,287,330,15) |
| GO:0009119 | ribonucleoside metabolic process | 6.49E-05 | 3.91 (20634,192,330,12) |
| GO:0016072 | rRNA metabolic process | 7.73E-05 | 4.14 (20634,166,330,11) |
| GO:0009167 | purine ribonucleoside monophosphate metabolic process | 8.53E-05 | 4.98 (20634,113,330,9) |
| GO:0071826 | ribonucleoprotein complex subunit organization | 9.07E-05 | 4.07 (20634,169,330,11) |
| GO:0009126 | purine nucleoside monophosphate metabolic process | 9.13E-05 | 4.94 (20634,114,330,9) |
| GO:0044085 | cellular component biogenesis | 9.28E-05 | 4.43 (20634,141,330,10) |
| GO:0006754 | ATP biosynthetic process | 1.04E-04 | 10.42 (20634,30,330,5) |
| GO:0009161 | ribonucleoside monophosphate metabolic process | 1.04E-04 | 4.85 (20634,116,330,9) |
| GO:1901576 | organic substance biosynthetic process | 1.13E-04 | 1.51 (20634,3199,330,77) |
| GO:0015985 | energy coupled proton transport, down electrochemical gradient | 1.30E-04 | 14.71 (20634,17,330,4) |
| GO:0015986 | ATP synthesis coupled proton transport | 1.30E-04 | 14.71 (20634,17,330,4) |
| GO:1903706 | regulation of hemopoiesis | 1.32E-04 | 2.93 (20634,341,330,16) |
| GO:0009123 | nucleoside monophosphate metabolic process | 1.85E-04 | 4.50 (20634,125,330,9) |
| GO:0009058 | biosynthetic process | 2.18E-04 | 1.47 (20634,3265,330,77) |
| GO:0002181 | cytoplasmic translation | 2.54E-04 | 12.51 (20634,20,330,4) |
| GO:0006396 | RNA processing | 2.83E-04 | 2.24 (20634,641,330,23) |
| GO:0044249 | cellular biosynthetic process | 3.76E-04 | 1.47 (20634,3108,330,73) |
| GO:0072521 | purine-containing compound metabolic process | 3.78E-04 | 3.06 (20634,266,330,13) |
| GO:0071840 | cellular component organization or biogenesis | 4.57E-04 | 1.37 (20634,4278,330,94) |
| GO:0055114 | oxidation-reduction process | 7.20E-04 | 2.02 (20634,773,330,25) |
| GO:0032543 | mitochondrial translation | 7.27E-04 | 9.62 (20634,26,330,4) |
| GO:0015992 | proton transport | 8.22E-04 | 5.44 (20634,69,330,6) |
| GO:0006818 | hydrogen transport | 8.87E-04 | 5.36 (20634,70,330,6) |
| GO:0034660 | ncRNA metabolic process | 9.48E-04 | 2.54 (20634,369,330,15) |
| GO:0009132 | nucleoside diphosphate metabolic process | 9.56E-04 | 5.28 (20634,71,330,6) |
| GO:0000462 | maturation of SSU-rRNA from tricistronic rRNA transcript (SSU-rRNA, 5.8S rRNA, LSU-rRNA) | 9.71E-04 | 8.93 (20634,28,330,4) |
| GO:0016043 | cellular component organization | 9.86E-04 | 1.35 (20634,4207,330,91) |

N - is the total number of genes

B - is the total number of genes associated with a specific GO term

n - is the number of genes in the top of the user's input list or in the target set when appropriate

b - is the number of genes in the intersection
