# Supplementary material for: RNA Sequencing of Murine Norovirus-Infected Cells Reveals Transcriptional Alteration of Genes Important to Viral Recognition and Antigen Presentation
Source: Front Immunol. 2017 Aug 11;8:959. doi: 10.3389/fimmu.2017.00959 (PMC5554501; doi:10.3389/fimmu.2017.00959)
Supplement: Supplementary file 5 [file Table_5.PDF]

TABLE S5 Enrichment terms generated from DAVID (MNV 12 hpi)

Upregulated genes				
**Goterm**	**Description**	**No. of genes**	**P-value**	**Fold enrichment**
GO:0006955	immune response	22	7.937885E-12	6.611288
GO:0006954	inflammatory response	16	4.136601E-11	10.065185
GO:0006952	defense response	20	2.134250E-10	6.318824
GO:0009611	response to wounding	18	2.374428E-10	7.342219
GO:0006935	chemotaxis	9	9.020053E-07	11.686927
GO:0042330	taxis	9	9.020053E-07	11.686927
GO:0044421	extracellular region part		2.803911E-06	3.980227
GO:0005615	extracellular space	14	3.038214E-06	4.964860
GO:0008009	chemokine activity	6	4.799136E-06	23.574216
GO:0042379	chemokine receptor binding	6	5.476310E-06	22.969749
GO:0005576	extracellular region	22	1.480373E-04	2.373085
GO:0005125	cytokine activity	8	1.877436E-04	6.635705
GO:0007626	locomotory behavior	9	2.654881E-04	5.330021
GO:0007610	behavior	11	5.233645E-04	3.844342
GO:0045321	leukocyte activation	8	8.537991E-04	5.170472
GO:0001819	positive regulation of cytokine production	5	9.172820E-04	11.414651
GO:0001775	cell activation	8	1.672501E-03	4.602981
GO:0051046	regulation of secretion	6	1.820327E-03	6.794000
GO:0046649	lymphocyte activation	7	2.200320E-03	5.187391
GO:0002526	acute inflammatory response	5	2.473161E-03	8.737140
GO:0001817	regulation of cytokine production	6	2.891078E-03	6.109712
GO:0005149	interleukin-1 receptor binding	3	3.225526E-03	34.454624
GO:0009897	external side of plasma membrane	6	5.200817E-03	5.278176
GO:0060341	regulation of cellular localization	6	5.400475E-03	5.274845
GO:0050727	regulation of inflammatory response	4	7.374607E-03	9.932749
GO:0010033	response to organic substance	10	8.727537E-03	2.802805
GO:0042110	T cell activation	5	8.846889E-03	6.100934
GO:0019724	B cell mediated immunity	4	1.057685E-02	8.710256
GO:0048585	negative regulation of response to stimulus	4	1.057685E-02	8.710256
GO:0002252	immune effector process	5	1.173327E-02	5.616733
GO:0060326	cell chemotaxis	3	1.515412E-02	15.726852
GO:0030595	leukocyte chemotaxis	3	1.515412E-02	15.726852
GO:0002449	lymphocyte mediated immunity	4	1.611348E-02	7.449561
GO:0030217	T cell differentiation	4	1.611348E-02	7.449561
GO:0042113	B cell activation	4	1.726459E-02	7.258547
GO:0006953	acute-phase response	3	1.852828E-02	14.154167

Upregulated genes				
**KEGG pathway**	**Description**	**No. of genes**	**P-value**	**Fold enrichment**
mmu04060	Cytokine-cytokine receptor interaction	12	2.388575E-06	5.130849
mmu04620	Toll-like receptor signaling pathway	9	3.194889E-06	9.484298
mmu04623	Cytosolic DNA-sensing pathway	7	1.063991E-05	13.278017
mmu04062	Chemokine signaling pathway	9	2.619065E-04	5.159041
mmu05332	Graft-versus-host disease	5	2.040880E-03	8.993730
mmu04621	NOD-like receptor signaling pathway	5	2.609993E-03	8.413490
mmu04940	Type I diabetes mellitus	5	2.767687E-03	8.279942
mmu04010	MAPK signaling pathway	9	3.048118E-03	3.543190
mmu04622	RIG-I-like receptor signaling pathway	5	3.655096E-03	7.671123
mmu04640	Hematopoietic cell lineage	5	7.752910E-03	6.209957
mmu05330	Allograft rejection	4	1.688581E-02	7.194984

Downregulated genes				
**Goterm**	**Description**	**No. of genes**	**P-value**	**Fold enrichment**
GO:0003735	structural constituent of ribosome	33	1.699990E-28	14.892308
GO:0005840	ribosome	36	1.270259E-27	11.840909
GO:0030529	ribonucleoprotein complex	44	9.751159E-22	6.014430
GO:0006412	translation	34	4.561589E-19	7.241254
GO:0005739	mitochondrion	67	1.587333E-18	3.200568
GO:0005198	structural molecule activity	35	2.011795E-15	5.300057
GO:0005743	mitochondrial inner membrane	28	1.351474E-13	5.973792
GO:0044429	mitochondrial part	36	2.804701E-13	4.338654
GO:0019866	organelle inner membrane	28	4.818120E-13	5.667444
GO:0005740	mitochondrial envelope	31	5.088110E-13	5.006898
GO:0000786	nucleosome	15	5.471107E-13	15.278592
GO:0031966	mitochondrial membrane	30	6.571546E-13	5.148221
GO:0070469	respiratory chain	15	1.101156E-12	14.573427
GO:0006334	nucleosome assembly	15	2.286625E-12	13.960274
GO:0031497	chromatin assembly	15	3.379725E-12	13.588000
GO:0031967	organelle envelope	35	3.407225E-12	4.093154

GO:0031975	envelope	35	3.777837E-12	4.078050
GO:0034728	nucleosome organization	15	4.090119E-12	13.409211
GO:0065004	protein-DNA complex assembly	15	4.090119E-12	13.409211
GO:0032993	protein-DNA complex	15	8.819590E-12	12.630303
GO:0006323	DNA packaging	16	1.775674E-11	10.762772
GO:0043228	non-membrane-bounded organelle	68	4.834580E-11	2.237782
GO:0043232	intracellular non-membrane-bounded organelle	68	4.834580E-11	2.237782
GO:0006333	chromatin assembly or disassembly	16	5.470010E-11	9.972844
GO:0006091	generation of precursor metabolites and energy	22	2.351921E-10	5.726743
GO:0031090	organelle membrane	38	3.325152E-09	2.966326
GO:0000785	chromatin	17	6.813270E-09	6.506520
GO:0022900	electron transport chain	14	9.190309E-09	8.492500
GO:0034622	cellular macromolecular complex assembly	18	1.969794E-08	5.635576
GO:0033279	ribosomal subunit 11	11	7.351550E-08	10.525253
GO:0034621	cellular macromolecular complex subunit organization	18	1.171634E-07	4.991510
GO:0015078	hydrogen ion transmembrane transporter activity	11	2.998590E-07	9.141213
GO:0015077	monovalent inorganic cation transmembrane transporter activity	11	5.264481E-07	8.615856
GO:0006325	chromatin organization	19	8.758960E-07	4.097968
GO:0006119	oxidative phosphorylation	9	1.430113E-06	10.918929
GO:0051276	chromosome organization	21	2.009683E-06	3.531535
GO:0022890	inorganic cation transmembrane transporter activity	12	2.464849E-06	6.438764
GO:0065003	macromolecular complex assembly	18	9.922945E-06	3.618107
GO:0009201	ribonucleoside triphosphate biosynthetic process	8	2.537519E-05	5.844301
GO:0043933	macromolecular complex subunit organization	18	2.849733E-05	3.332207
GO:0005694	chromosome	19	2.951498E-05	3.174283
GO:0044427	chromosomal part	17	4.293135E-05	3.376024
GO:0015935	small ribosomal subunit	6	8.054992E-05	13.065831
GO:0044455	mitochondrial membrane part	7	1.127618E-04	9.021645
GO:0045259	proton-transporting ATP synthase complex	5	1.924203E-04	16.618820
GO:0004298	threonine-type endopeptidase activity	5	2.169907E-04	16.224664
GO:0070003	threonine-type peptidase activity	5	2.169907E-04	6.224664
GO:0005839	proteasome core complex	5	2.375752E-04	15.787879
GO:0015934	large ribosomal subunit	6	3.437232E-04	0.715618
GO:0019843	rRNA binding	5	3.722801E-04	14.196581
GO:0009206	purine ribonucleoside triphosphate biosynthetic process	8	4.253752E-04	5.844301
GO:0009145	purine nucleoside triphosphate biosynthetic process	8	4.539916E-04	5.782128
GO:0009142	nucleoside triphosphate biosynthetic process	8	4.841242E-04	5.721263
GO:0016469	proton-transporting two-sector ATPase complex	6	4.888027E-04	9.021645
GO:0009205	purine ribonucleoside triphosphate metabolic process	8	7.000656E-04	5.381386
GO:0009199	ribonucleoside triphosphate metabolic process	8	7.424894E-04	5.328627
GO:0009144	purine nucleoside triphosphate metabolic process	8	9.329574E-04	5.127547
GO:0015992	proton transport	6	9.525466E-04	7.839231
GO:0045263	proton-transporting ATP synthase complex, coupling factor	4	9.804001E-04	19.431235
GO:0009152	purine ribonucleotide biosynthetic process	8	9.861106E-04	5.079626
GO:0006818	hydrogen transport	6	1.039430E-03	7.691321
GO:0009260	ribonucleotide biosynthetic process	8	1.223010E-03	4.896577
GO:0006754	ATP biosynthetic process	7	1.272353E-03	5.799756
GO:0009141	nucleoside triphosphate metabolic process	8	1.502358E-03	4.726261
GO:0009150	purine ribonucleotide metabolic process	8	1.829153E-03	4.567395
GO:0015985	energy coupled proton transport	5	2.019645E-03	9.181081
GO:0015986	ATP synthesis coupled proton transport	5	2.019645E-03	9.181081
GO:0055114	oxidation reduction	21	2.049106E-03	2.123125
GO:0046034	ATP metabolic process	7	2.054619E-03	5.284222
GO:0042773	ATP synthesis coupled electron transport	4	2.146681E-03	15.097778
GO:0000502	proteasome complex	6	2.148942E-03	6.532915
GO:0009259	ribonucleotide metabolic process	8	2.419825E-03	4.348160
GO:0022626	cytosolic ribosome	4	2.639554E-03	14.033670
GO:0008121	ubiquinol-cytochrome-c reductase activity	3	3.060383E-03	34.071795
GO:0016679	oxidoreductase activity, diphenols and related substances as donors	3	3.060383E-03	34.071795
GO:0016681	oxidoreductase activity, cytochrome as acceptor	3	3.060383E-03	34.071795
GO:0034220	ion transmembrane transport	5	3.233656E-03	8.088095
GO:0006164	purine nucleotide biosynthetic process	8	4.032204E-03	3.967299
GO:0009165	nucleotide biosynthetic process	9	4.136400E-03	3.514138
GO:0033177	proton-transporting two-sector ATPase complex	4	4.756340E-03	11.482094
GO:0034404	nucleobase, nucleoside and nucleotide biosynthetic process	9	4.901332E-03	3.415978
GO:0034654	nucleobase, nucleoside, nucleotide and nucleic acid biosynthetic process	9	4.901332E-03	3.415978
GO:0005753	mitochondrial proton-transporting ATP synthase complex	3	4.923091E-03	27.064935
GO:0006007	glucose catabolic process	5	6.983620E-03	6.532692
GO:0019320	hexose catabolic process	5	6.983620E-03	6.532692
GO:0022904	respiratory electron transport chain	4	7.741227E-03	9.705714
GO:0046365	monosaccharide catabolic process	5	7.975804E-03	6.290741
GO:0006163	purine nucleotide metabolic process	8	9.290917E-03	3.397000
GO:0044275	cellular carbohydrate catabolic process	5	1.149392E-02	5.661667
GO:0019318	hexose metabolic process	8	1.232187E-02	3.216095
GO:0005829	cytosol	17	1.320928E-02	1.955511
GO:0044271	nitrogen compound biosynthetic process	11	1.360401E-02	2.474636

GO:0046164	alcohol catabolic process	5	1.508629E-02	5.226154
GO:0008565	protein transporter activity	5	1.571061E-02	5.162393
GO:0006006	glucose metabolic process	7	1.703875E-02	3.397000
GO:0042775	mitochondrial ATP synthesis coupled electron transport	3	1.729757E-02	14.558571

**Downregulated genes**				
**KEGG pathway**	**Description**	**No. of genes**	**P-value**	**Fold enrichment**
mmu03010	Ribosome	28	4.419635E-25	14.796832
mmu00190	Oxidative phosphorylation	22	1.445609E-13	7.959395
mmu05012	Parkinson's disease	18	1.658032E-09	6.365340
mmu05322	Systemic lupus erythematosus	16	2.595848E-09	7.306064
mmu05010	Alzheimer's disease	19	3.364557E-08	4.910016
mmu05016	Huntington's disease	19	3.666785E-08	4.883186
mmu04260	Cardiac muscle contraction	8	1.192366E-03	4.823876
mmu03050	Proteasome	6	2.900865E-03	6.004186

The enrichment analysis was completed with DAVID. The GO terms in these lists are ranked by p-value.
Ontology and pathway terms were deemed significant if their p-value was <0.02
GOterms represent a combination of BP (biological processes), CC (cellular compartments) and MF (molecular function)
