# Supplementary material for: RNA Sequencing of Murine Norovirus-Infected Cells Reveals Transcriptional Alteration of Genes Important to Viral Recognition and Antigen Presentation
Source: Front Immunol. 2017 Aug 11;8:959. doi: 10.3389/fimmu.2017.00959 (PMC5554501; doi:10.3389/fimmu.2017.00959)
Supplement: Supplementary file 6 [file Table_6.PDF]

TABLE S6 Significantly expressed genes (MNV 12 hpi vs Lox 12 hpt)

**MNV-only upregulated genes**		
**Gene**	**Gene Name**	**Fold Change**
Lamc2	Laminin, gamma 2	34.77
Flrt3	Fibronectin leucine rich transmembrane protein 3	14.47
Kdm6b	Lysine specific demethylase 6B	7.00
Arc	Activity regulated cytoskeletal-associated protein	6.45
Olr1	Oxidized low density lipoprotein receptor 1	4.94
Il7r	Interleukin 7 receptor	4.77
Gem	GTP binding protein overexpressed in skeletal muscle	4.63
Lars2	Leucyl-tRNA synthetase, mitochondrial	3.59
Rgs16	Regulator of G-protein signaling 16	3.43
C030037D09Rik	Riken cDNA C030037D09 gene	3.37
Gpr35	G protein-coupled receptor 35	3.33
Jun	Jun proto-oncogene(Jun)	3.29
1700012D01Rik	Riken cDNA 1700012D01 gene	3.14
3300002P13Rik	Riken cDNA 3300002P13 gene	3.10
Rgs1	Regulator of G-protein signaling 1	2.96
B230217O12Rik	Riken cDNA B230217O12 gene	2.71
Mmp12	Matrix metallopeptidase 12	2.65
Gapt	Grb2-binding adaptor, transmembrane	2.52
Bzrap1	benzodiazepine receptor associated protein 1(Bzrap1)	2.51
Zbtb10	Zinc finger and BTB domain containing 10	2.51
Rnu12	U12 small nuclear RNA	2.45
Mmp9	Matrix metallopeptidase 9	2.41
Pla2g4c	Phospholipase A2 group IVC	2.41
Gp1bb,Sept5	Glycoprotein Ib, beta polypeptide	2.39
Gm6377	Predicted gene 6377	2.38
Igfals	Insulin-like growth factor binding protein, acid labile subunit	2.33
Cald1	Caldesmon 1	2.32
Rgs8	Regulator of G-protein signaling 8	2.29
Fgr	FGR proto-oncogene, Src family tyrosine kinase	2.28
Jazf1	JAZF zinc finger 1(Jazf1)	2.24
Cyp4f37	Cytochrome P450, family 4, subfamily f, polypeptide 37	2.17
Hnrnpd	Heterogeneous nuclear ribonucleoprotein D	2.16
Spink5	Serine peptidase inhibitor, Kazal type 5	2.15
6820431F20Rik	Cadherin 11 pseudogene	2.11
Slc7a2	Solute carrier family 7 member 2	2.10
Aoah	Acycloxyacyl hydrolase	2.09
Kcnab1	Potassium voltage-gated channel, shaker-related subfamily, beta member 1	2.09
Rnu11	U11 small nuclear RNA	2.08
Olfr934	Olfactory receptor 934	2.04
Tanc2	Tetratricopeptide repeat, ankyrin repeat and coiled-coil containing 2	2.04
Pde3b	Phosphodiesterase 3B	2.02
Atg2a	Autophagy related 2A	2.02
Kazald1	Kazal-type serine peptidase inhibitor domain 1	2.00

**MNV-only downregulated genes**		
**Gene**	**Gene Name**	**Fold Change**
Hist1h2br	Histone cluster 1 H2br	-120.20
H2-Q10	Histocompatibility 2, Q region locus 10	-23.55
Dnaja1	DnaJ heat shock protein family (Hsp40) member A1	-7.79
Gm11127	Predicted gene 11127	-3.77
Dynlt1b	Dynein light chain Tctex-type 1B	-3.71

Shb	Src homology 2 domain-containing transforming protein B	-2.71
Rpl11	Ribosomal protein L11	-2.65
Hmgcl	3-hydroxy-3-methylglutaryl-Coenzyme A lyase	-2.60
Mlycd	Malonyl-CoA decarboxylase	-2.57
Psmb4	Proteasome subunit, beta type 4	-2.53
Psme2	Proteasome activator subunit 2	-2.50
Lst1	Leukocyte specific transcript 1	-2.49
Cebpe	CCAAT/enhancer binding protein epsilon	-2.44
Psmb5	Proteasome subunit, beta type 5	-2.42
Coprs	Coordinator of PRMT5 and differentiation stimulator	-2.40
Snta1	Syntrophin, alpha 1	-2.39
C1qc	Complement component 1, q subcomponent, C chain	-2.37
Lrrc17	Leucine rich repeat containing 17	-2.36
Psmb3	Proteasome subunit, beta type 3	-2.31
Tnfaip8l2	Tumor necrosis factor, alpha-induced protein 8-like 2	-2.31
Fbxw4	F-box and WD-40 domain protein 4	-2.31
Tmem115	Transmembrane protein 115	-2.27
Pdcd5	Programmed cell death 5	-2.25
Gchfr	GTP cyclohydrolase I feedback regulator	-2.24
Ap1s1	Adaptor protein complex AP-1, sigma 1	-2.23
Coro1a	Coronin, actin binding protein 1A	-2.22
Mrpl27	Mitochondrial ribosomal protein L27	-2.21
Ocel1	Occludin/ELL domain containing 1	-2.20
Snx20	Sorting nexin 20	-2.20
Hddc3	HD domain containing 3	-2.19
Ap2s1	Adaptor-related protein complex 2, sigma 1 subunit	-2.19
Slc25a11	Aolute carrier family 25 member 11	-2.19
Ogfod2	2-oxoglutarate and iron-dependent oxygenase domain containing 2	-2.18
Il17rc	Interleukin 17 receptor C	-2.16
Bad	Bcl2-associated agonist of cell death	-2.16
Acbd6	Acyl-Coenzyme A binding domain containing 6	-2.15
Stard3nl	STARD3 N-terminal like	-2.15
Zfand2b	Zinc finger, AN1 type domain 2B	-2.14
Chmp1a	Charged multivesicular body protein 1A	-2.14
Dnajc22	DnaJ heat shock protein family (Hsp40) member C22	-2.12
Dok3	Docking protein 3	-2.11
Timm23	Translocase of inner mitochondrial membrane 23	-2.10
Esrra	Estrogen related receptor, alpha	-2.10
Tmem134	Transmembrane protein 134	-2.10
Snx15	Sorting nexin 15	-2.09
Myeov2	Myeloma overexpressed 2	-2.08
Ccdc142	Coiled-coil domain containing 142	-2.08
Atp13a2	ATPase type 13A2	-2.08
Aacs	Acetoacetyl-CoA synthetase	-2.08
Sec61g	SEC61, gamma subunit(Sec61g)	-2.08
Gabarapl2	Gamma-aminobutyric acid receptor-associated protein-like 2	-2.07
Med28	Mediator complex subunit 28	-2.06
Atp6v1f	ATPase H+ transporting lysosomal V1 subunit F	-2.06
Echs1	Enoyl Coenzyme A hydratase, short chain, 1, mitochondrial	-2.05
Nradd	Neurotrophin receptor associated death domain	-2.05
Rhox5	Reproductive homeobox 5	-2.05
BC022687	Riken cDNA BC022687 gene	-2.04
Mvb12a	Multivesicular body subunit 12A	-2.04
Sash3	SAM and SH3 domain containing 3	-2.03
Pycr2	Pyrroline-5-carboxylate reductase family, member 2	-2.03
Rsph3a	Radial spoke 3A homolog	-2.03
Bst2	Bone marrow stromal cell antigen 2	-2.02

Adck1	AarF domain containing kinase 1	-2.01
Mrps18c	Mitochondrial ribosomal protein S18C	-2.01
Gm561	Predicted gene 561	-2.01
Gtpbp3	GTP binding protein 3	-2.01
Cib1	Calcium and integrin binding 1	-2.00
Mief2	Mitochondrial elongation factor 2	-2.00
Psmb9	Proteasome subunit, beta type 9	-2.00
