# Supplementary material for: RNA Sequencing of Murine Norovirus-Infected Cells Reveals Transcriptional Alteration of Genes Important to Viral Recognition and Antigen Presentation
Source: Front Immunol. 2017 Aug 11;8:959. doi: 10.3389/fimmu.2017.00959 (PMC5554501; doi:10.3389/fimmu.2017.00959)
Supplement: Supplementary file 7 [file Table_7.PDF]

TABLE S7 Enrichment terms generated from DAVID (MNV 12 hpi vs. Lox 12 hpt)

MNV-only upregulated genes				
**GOterm**	**Description**	**No. of genes**	**P-value**	**Fold enrichment**
	positive regulation of exocytosis	2	2.70E-02	69.7
	regulation of secretion	3	2.90E-02	10.9
	response to wounding	4	3.70E-02	5.2
	regulation of cellular localization	3	4.60E-02	8.4
	negative regulation of signal transduction	3	5.10E-02	7.9
	negative regulation of cell communication	3	5.90E-02	7.3
	regulation of exocytosis	2	6.60E-02	28.3
	defense response	4	6.90E-02	4
	leukocyte activation	3	7.90E-02	6.2
	inflammatory response	3	8.30E-02	6
	negative regulation of macromolecule metabolic process	4	9.20E-02	3.6
	cell activation	3	9.60E-02	5.5
	insulin-like growth factor binding	2	4.50E-02	41.7
	syntaxin binding	2	5.10E-02	36.7
	SNARE binding	2	5.70E-02	32.7
	GTPase activator activity	3	6.10E-02	7.2
	enzyme activator activity	3	9.60E-02	5.5

MNV-only downregulated genes				
**GOterm**	**Description**	**No. of genes**	**P-value**	**Fold enrichment**
	threonine-type endopeptidase activity	4	3.50E-05	60.3
	threonine-type peptidase activity	4	3.50E-05	60.3
	protein transporter activity	4	1.10E-03	19.2
	P-P-bond-hydrolysis-driven transmembrane transporter activity	4	4.30E-03	11.8
	primary active transmembrane transporter activity	4	4.40E-03	11.7
	protein transport	8	5.20E-03	3.6
	establishment of protein localization	8	5.40E-03	3.6
	protein localization	8	1.10E-02	3.1
	regulation of lymphocyte activation	4	1.20E-02	8.2
	regulation of leukocyte activation	4	1.40E-02	7.7
	regulation of cell activation	4	1.50E-02	7.6
	programmed cell death	6	1.90E-02	3.7
	positive regulation of T cell activation	3	2.00E-02	13.4
	cell death	6	2.50E-02	3.5
	death	6	2.80E-02	3.4
	regulation of lymphocyte proliferation	3	2.90E-02	11.1
	regulation of mononuclear cell proliferation	3	2.90E-02	11.1
	regulation of leukocyte proliferation	3	3.00E-02	10.8
	P-P-bond-hydrolysis-driven protein transmembrane transporter	2	3.00E-02	63.3
	macromolecule transmembrane transporter activity	2	3.00E-02	63.3
	protein transmembrane transporter activity	2	3.00E-02	63.3
	positive regulation of immune system process	4	3.10E-02	5.7
	antigen processing and presentation	3	3.40E-02	10.2
	positive regulation of lymphocyte activation	3	3.90E-02	9.4
	positive regulation of leukocyte activation	3	4.20E-02	9
	positive regulation of cell activation	3	4.40E-02	8.8
	regulation of T cell activation	3	5.00E-02	8.2
	intracellular transport	5	5.40E-02	3.4
	intracellular protein transport	4	6.30E-02	4.3
	apoptosis	5	6.70E-02	3.2
	homeostasis of number of cells within a tissue	2	6.70E-02	28.1
	tumor necrosis factor receptor binding	2	6.90E-02	27.5
	immune response	5	7.00E-02	3.1
	cellular protein localization	4	7.60E-02	4
	cellular macromolecule localization	4	7.70E-02	3.9
	tumor necrosis factor receptor superfamily binding	2	7.70E-02	24.3
	structural constituent of ribosome	3	7.90E-02	6.3
	lipid binding	4	9.00E-02	3.7
	proteolysis involved in cellular protein catabolic process	5	9.90E-02	2.8
