# Supplementary material for: RNA Sequencing of Murine Norovirus-Infected Cells Reveals Transcriptional Alteration of Genes Important to Viral Recognition and Antigen Presentation
Source: Front Immunol. 2017 Aug 11;8:959. doi: 10.3389/fimmu.2017.00959 (PMC5554501; doi:10.3389/fimmu.2017.00959)
Supplement: Supplementary file 8 [file Table_8.PDF]

TABLE S8 List of qPCR primers used to determine gene expression changes with MNV infection

Gene ID	Forward (5'-3')	Reverse (5'-3-)
ISG15	AAGCAGACTCCTTAATTCCAG	CCTCCATGGGCCTTCCCTCGA
IRF3	GATGGAGAGGTCCACAAGGA	GAGTGTAGCGTGGGGAGTGT
IRF7	CTGGAGCCATGGGTATGCA	AAGCACAAGCCGAGACTGCT
DHX58	GAGACCTGGAGGAACCATCA	CCCTCGAGGTGTTTCCAGTA
TLR3	ACTTGCTATCTTGGATGC	AGTTCTTCACTTCGCAAC
TLR7	TGACTCTCTTCTCCTCCA	GCTTCCAGGTCTAATCTG
DDX58	CAGTACATTCAGGCTGAG	GGCCAGTTTTCCTTGTC
IFIH1	GGCACCATGGGAAGTGATT	ATTTGGTAAGGCCTGAGCTG
STAT-1	GCAAGCGTAATCTCCAGGA	GGCTTCACCTTCTCTGTTCT
TNF-α	CAGCCTCTTCTCATTCCTGC	CAGCCTCTTCTCATTCCTGC
IKBKE	ACAAGGCCCGCAACAAGAAA	TTCACGATGTTCTGGTGATTC
IFNb	TCCCTATGGAGATGACGGAG	ACCCAGTGCTGGAGAAATTG
IFNa2	AGCAGATCCAGAAGGCTCAA	GGAGGGTTGTATTCCAAGCA
IL-1b	AAGGAGAACCAAGCAACGACAAAA	TGGGGAACTCTGCAGACTCAAACT
IL-6	GATGGATGCTACCAAACTGGA	TCTGAAGGACTCTGGCTTTG
CCL4	CTCTCCTCTTGCTCGTGGC	GTACTCAGTGACCCAGGGCTC
NF-kB1	GAAATTCCTGATCCAGACAAAAAC	ATCACTTCAATGGCCTCTGTGTAG
NF-kB2	CTGGTGGACACATACAGGAAGAC	ATAGGCACTGTCTTCTTTCACCTC
c-Fos	GGGGACAGCCTTTCCTACTA	CTGTCACCGTGGGGATAAAG
c-Jun	ACGACCTTCTACGACGATGC	CCAGGTTCAAGGTCATGCTC
GAPDH	AGCTTGTCATCAACGGGAAG	TTTGATGTTAGTGGGGTCTCG
