# Supplementary material for: RNA Sequencing of Murine Norovirus-Infected Cells Reveals Transcriptional Alteration of Genes Important to Viral Recognition and Antigen Presentation
Source: Front Immunol. 2017 Aug 11;8:959. doi: 10.3389/fimmu.2017.00959 (PMC5554501; doi:10.3389/fimmu.2017.00959)
Supplement: Supplementary file 9 [file Table_9.PDF]

TABLE S9 Read mapping to Abelson and Moloney Mu-LV

Sample	Total no. of reads	No. of reads mapped		% of reads mapped		% genome coverage	
		[a]Ab Mu-LV	[b]Mo Mu-LV	Ab Mu-LV	Mo Mu-LV	Ab Mu-LV	Mo Mu-LV
mock	57380304	40	3623	7.0E-05	6.3E-03	3.1	6.3
4 hpi	59221498	50	4110	8.4E-05	6.9E-03	4.9	7.0
8 hpi	61203972	50	3779	8.2E-05	6.2E-03	4.2	7.7
12 hpi	54950136	50	3144	9.1E-05	5.7E-03	4.3	6.9
16 hpi	56463488	40	3000	7.1E-05	5.3E-03	3.0	6.6
20 hpi	57627090	50	3105	8.7E-05	5.4E-03	3.1	7.1

[a] Abelson Murine Leukemia Virus

[b] Moloney Murine Leukemia Virus
